# Samarium(II) folding cascades involving hydrogen atom transfer for the synthesis of complex polycycles

**DOI:** 10.1038/s41467-018-07194-x

**Published:** 2018-11-15

**Authors:** Mateusz P. Plesniak, Monserrat H. Garduño-Castro, Philipp Lenz, Xavier Just-Baringo, David J. Procter

**Affiliations:** School of Chemistry, University of Manchester, Oxford Road, Manchester, M13 9PL UK

## Abstract

The expedient assembly of complex, natural product-like small molecules can deliver new chemical entities with the potential to interact with biological systems and inspire the development of new drugs and probes for biology. Diversity-oriented synthesis is a particularly attractive strategy for the delivery of complex molecules in which the 3-dimensional architecture varies across the collection. Here we describe a folding cascade approach to complex polycyclic systems bearing multiple stereocentres mediated by reductive single electron transfer (SET) from SmI_2_. Simple, linear substrates undergo three different folding pathways triggered by reductive SET. Two of the radical cascade pathways involve the activation and functionalization of otherwise inert secondary alkyl and benzylic groups by 1,5-hydrogen atom transfer (HAT). Combination of SmI_2_, a privileged reagent for cascade reactions, and 1,5-HAT can lead to complexity-generating radical sequences that unlock access to diverse structures not readily accessible by other means.

## Abstract (web summary)

Diversity-oriented synthesis is a valuable strategy to construct complex molecules of medicinal interest. Here, the authors show a folding cascade strategy to convert linear substrates into polycyclic compounds with multiple stereocentres by combining the reductive chemistry of SmI_2_ with 1,5-hydrogen atom transfer.

## Introduction

The synthesis of complex small molecules that can interact with biological systems is of paramount importance in science. Advances in the field can lead to new therapeutic agents and probes for molecular biology that allow intricate biological processes to be unravelled^1,2^. Target-oriented synthesis, medicinal and combinatorial chemistry, and diversity-oriented synthesis are the main strategies used to gain access to collections of complex small molecules^3^. While the first two approaches target defined structure space of known biological relevance, diversity-oriented synthesis attempts to deliver a diverse range of molecular architectures than can give access to unexplored activity and the promise of greater potency^4–9^. These products often possess unique three-dimensional (3D) shapes^10–14^ that can enhance their interaction with biological targets, in contrast to more traditional medicinal chemistry scaffolds that are two dimensional^15–17^.

A main goal of diversity-oriented approaches is to concomitantly access structurally complex molecules and skeletal diversity^18^. In this regard, cascade processes that convert simple starting materials into complex products, in one pot, are ideal^19–22^. Furthermore, approaches in which the presence of a particular group in a substrate can direct the reaction down one of several pathways, the so-called substrate-based approaches, are highly desirable tools for accessing skeletal diversity^23^.

Recent studies in the field of radical chemistry have shown the great potential of open shell intermediates, generated selectively under mild conditions, for use in cascade processes in which complex polycyclic systems bearing multiple stereocentres are formed in a single operation^24–26^. Of particular interest are radical processes that exploit hydrogen atom transfer (HAT) to functionalise remote, inert C–H bonds^27–29^. In the context of complexity generation, HAT processes allow radicals to be relocated to sites at which new bond-forming events can take place, thus extending reaction cascades^30^. While recent reports in the field of diversity-oriented synthesis have utilised radical reactions to access diverse molecular space^31–34^, the exploitation of HAT in such processes remains largely unexplored.

**Fig. 1:**
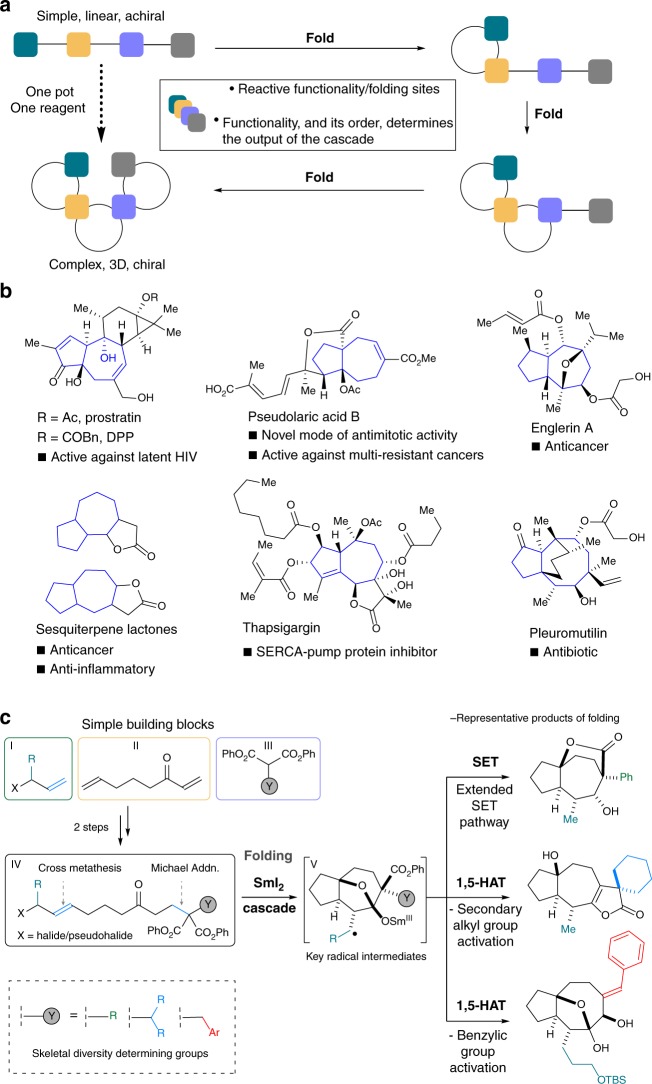
A folding cascade approach that delivers polycyclic architectures and the samarium(II) folding cascade approach. The folding cascade approach, examples of biologically active natural products bearing [5,7] and [5,8] fused bicyclic motifs, and a samarium(II)-mediated, one-pot cascade reaction enabling access to these architectures, are shown. **a** Folding cascade approaches convert simple, linear starting materials to complex 3D polycyclic architectures through the sequential action of a single reagent on functional groups arranged in the substrate to be folded. **b** Biologically relevant natural products possessing multiple stereocentres and sites of oxygenation, and [5,7] or [5,8] ring systems include prostratin, pseudolaric acid B, englerin A, sesquiterpene lactones such as thapsigargin, and pleuromutilin. **c** This work involves the development of a one-pot approach to complex polycyclic architectures from simple starting materials. The approach exploits samarium(II) folding cascades featuring 1,5-HAT functionalisation of tertiary and benzylic C–H bonds

Herein we present a folding cascade approach in which simple, linear starting materials are converted to complex 3D polycyclic architectures through the sequential action of a single reagent on functional groups arranged in the substrate to be folded (Fig. 1a).^18–20^ In particular, using the reductive single electron transfer (SET) reagent samarium diiodide (SmI_2_)^35–38^ in folding cascades, radical (and anionic) character is generated at various points during the one-pot/one-reagent folding process and utilised for selective carbon–carbon bond formation. In our substrate-based approach, skeletal diversity is achieved by the formation of different polycyclic ring systems, quaternary centres, and spirocyclic functionalities, accompanied by the generation of up to five stereocentres. The four distinct scaffolds generated resemble those found in many complex and biologically active natural products (Fig. 1b). The starting materials **IV** to be folded are typically accessed in modular fashion from terminal alkenes **I** bearing allylic functionality X, keto diene building block **II**, and diphenoxy malonates **III**, in two steps (Michael addition and alkene cross-metathesis). In particular, malonates **III** contain groups that determine the course of the folding cascades. Depending on the nature of the group Y, key radical intermediates **V** can follow three distinct pathways to generate four types of complex scaffold. Two of the folding pathways involve 1,5-hydrogen atom transfer (HAT), an important process that has rarely been exploited in the chemistry of SmI_2_^39–41^ or in diversity-oriented synthesis (Fig. 1c).

## Results

### Samarium(II) folding cascades involving an extended SET pathway

**Fig. 2:**
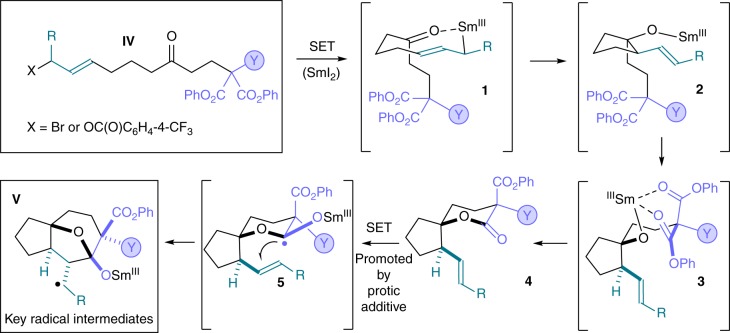
Mechanistic platform for the radical folding cascades. A mechanism for the common stages of the samarium(II) folding cascades and the formation of the key radical intermediates **V** is shown. The sequence commences with an intramolecular, samarium(II)-mediated Barbier reaction to give alkoxides **2** which yield **4** after diastereoselective lactonisation. Addition of water then triggers lactone radical cyclisations involving radicals **5**

The mechanistic platform upon which our SmI_2_-mediated folding radical cascades are built involves a Barbier cyclisation–lactonisation–lactone radical cyclisation sequence to generate key radical intermediates **V** with high diastereocontrol (Fig. 2). Allyl samariums **1** are initially formed by reduction of the allylic halide/pseudohalide moiety (X = Br or OC(O)C_6_H_4_-4-CF_3_) and undergo diastereoselective addition to the ketone carbonyl to give samarium alkoxides **2**. Diastereoselective lactonisation via transition structure **3** establishes a α-all-carbon quaternary stereocentre and gives lactones **4**. The addition of a protic additive to the reaction vessel activates SmI_2_^42,43^ and switches on the next stage of the sequence: SET reduction of the lactone carbonyl in **4** gives ketyl radicals **5** and 5-*exo*-trig radical cyclisation generates the key radicals **V**^44^.

**Fig. 3:**
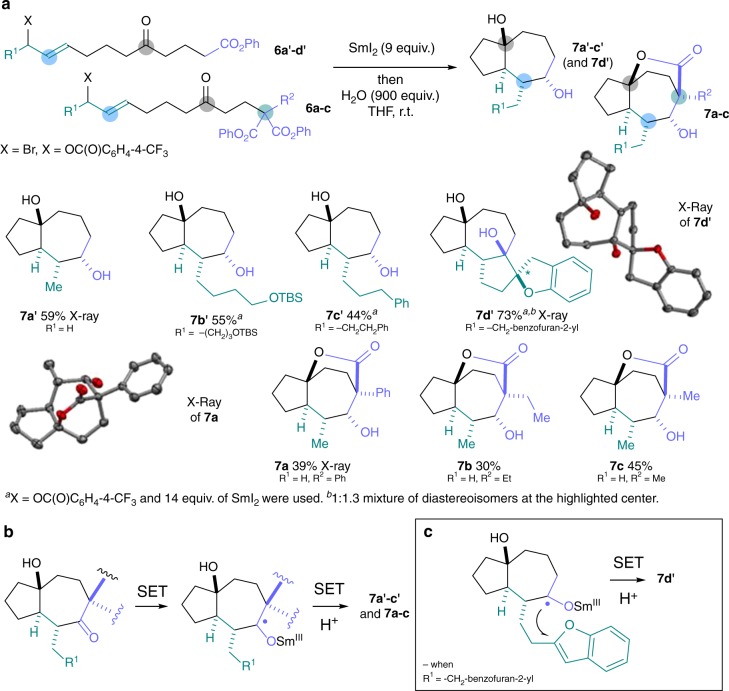
Folding radical cascades following extended SET pathways. Samarium(II) folding cascades involving an extended SET pathway from key radical intermediates **V** are shown. **a** Folding of the substrates **6a’**–**d’** allowed access to diols **7a’**–**d’** bearing up to five new stereocentres. The use of malonate-derived substrates **6a**–**6c** allowed the synthesis of complex lactones **7a**–**7c**. **b** The formation of products involves SET reduction of the carbonyl and diastereoselective protonation to give diols **7a’**–**c’**. In the case of products **7a**–**c**, the alcohol formed in the last step undergoes lactonisation. **c** The formation of product **7d’** involves a dearomatising radical cyclisation of the samarium(II) ketyl radical intermediate formed upon ketone reduction

Initially, substrates were designed to follow an extended SET pathway in which radicals **V** were reduced further and the anions protonated by the H_2_O additive (Fig. 3a). Subsequent opening of the hemiketal in the products and SET reduction of the resulting ketones gave secondary alcohols **7a’–c’** and **7a–7c** from the folding of simple substrates with complete diastereocontrol (Fig. 3b). Alternatively, the ketyl radical intermediates formed in the ketone reduction can be trapped by a pendant heteroarene to forge spirocyclic scaffold **7d’** (Fig. 3c). Notably, linear starting materials **6a**–**6c**, prepared from diphenylmalonate building blocks **III**, underwent lactonisation in the final stage of the cascade process to deliver bridged lactone products **7a–7c**. All products from this extended SET pathway were obtained in moderate to good overall isolated yield (30–73%) and as single diastereoisomers (with the exception of **7d’**). Notably, up to 5 contiguous stereocentres and 3 rings are formed in the folding cascades.

### Samarium(II) folding cascades involving 1,5-HAT from a secondary centre

**Fig. 4:**
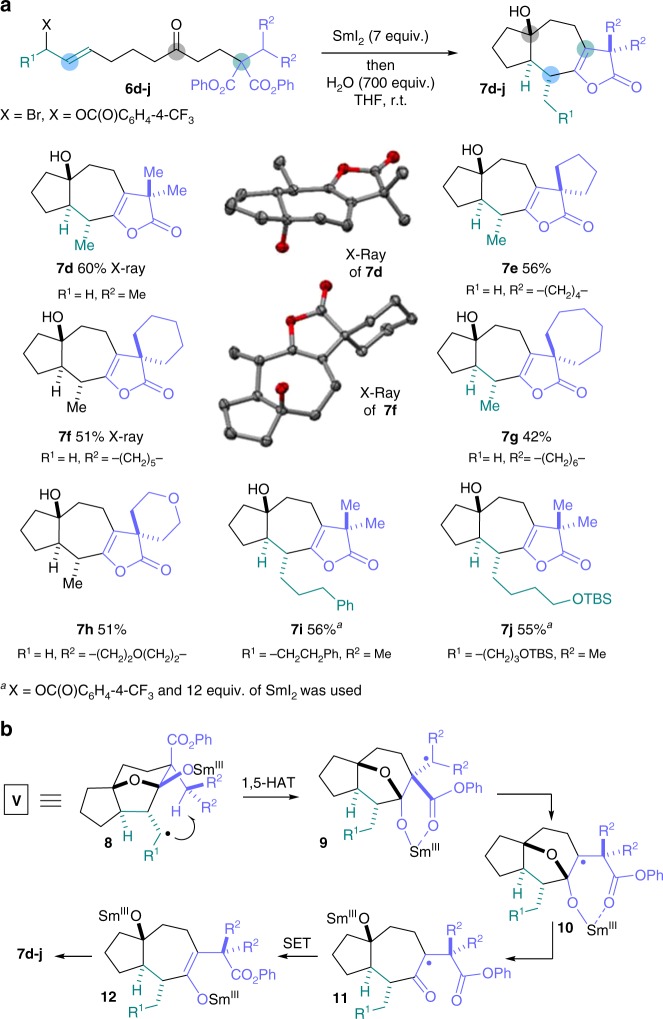
Folding radical cascades involving 1,5-HAT and activation of a secondary alkyl group. A samarium(II) folding cascade involving 1,5-HAT activation of secondary alkyl groups in key radical intermediates **V** is shown. **a** Scope of the samarium(II) folding cascade involving activation of a secondary alkyl group by 1,5-HAT to give products **7d**–**j**. **b** The mechanism of folding involves 1,5-HAT in key radical intermediates **V** to give **9**. A 1,2-ester shift then generates radical intermediates **10** that, after hemiketal opening and SET reduction, give samarium(III) enolates **12**. The final products **7d**–**j** are obtained by lactonisation of enolates **12**

To extend the radical cascades and to functionalise otherwise unreactive substrate sites during folding, we sought to relocate the radicals in **V** using intramolecular HAT, and to trap the new radicals, thus accessing diverse structures. Crucially, HAT processes have little precedent in the chemistry of SmI_2_ as radical intermediates are typically reduced rapidly to the corresponding carbanions^45^. We reasoned that careful substrate design could lead to radical intermediates **V** in which the radical centre was sufficiently close to the site of abstraction for the rate of 1,5-HAT to outcompete the usual radical reduction and protonation that dominates SmI_2_ chemistry. The proposed 1,5-HAT pathway was explored using malonate components **III** bearing secondary alkyl groups possessing tertiary C–H bonds β- to the ester carbonyls. Pleasingly, simple substrates **6d–j** underwent radical folding cascades to give complex tricyclic lactones **7d–j** in good overall yield and as single diastereoisomers (Fig. 4a). Notably, malonate substrates **6e–h** containing carbo- and heterocyclic motifs, conveniently introduced using a suitably substituted malonate component **III**, delivered lactone products bearing carbo- and hetero-spirocyclic rings **7e–h**.

The mechanism of the folding cascades involving 1,5-HAT from a secondary alkyl group is shown in Fig. 4b. After the initial Barbier cyclisation – lactonisation – lactone cyclisation sequence (see Fig. 2), 1,5-HAT in radical **8** activates the secondary alkyl group in the malonate unit and radicals **9** are formed. Crucially, reduction of tertiary radicals **9** to the corresponding anions is slower than intramolecular radical addition and the radicals undergo 1,2-migration of the ester group^46^ to give stabilised radicals **11** after hemiketal collapse. Subsequent SET gives samarium enolates **12**^47^ that undergo lactonisation. As proposed, the facile nature of the 1,5-HAT process, relative to reduction of **8** to the corresponding anions, is likely the result of the proximity of the radicals in **V**, formed from the diastereoselective lactonisation–lactone cyclisation sequence, to the alkyl sidechain located α to the ester (Fig. 4b).

### Samarium(II) folding cascades involving 1,5-HAT from a benzylic centre

Building on the successful use of 1,5-HAT to activate secondary alkyl groups in the folding cascades, we proposed that remote activation of a benzylic position could lead to alternative product architectures. Benzyl-containing substrates **6k**–**q** were prepared using suitably substituted malonates **III** in two steps. Exposure of **6k–q** to SmI_2_ delivered 5,8-carbocylic cascade products **7k**–**q** as single diastereoisomers in moderate yield. Notably, the folding cascades feature five bond-forming events and establish five new contiguous stereocentres. In this case it was found that MeOH, and not H_2_O, was the optimal protic additive for use in conjunction with SmI_2_^48–50^. This may be due to the particular conformation of lactone intermediates **4** (Fig. 2; when Y = CH_2_Ar) and promotion of lactone carbonyl reduction by coordination of SmI_2_ to the alpha ester group, thus negating the need for water activation of the reagent.^51^

**Fig. 5:**
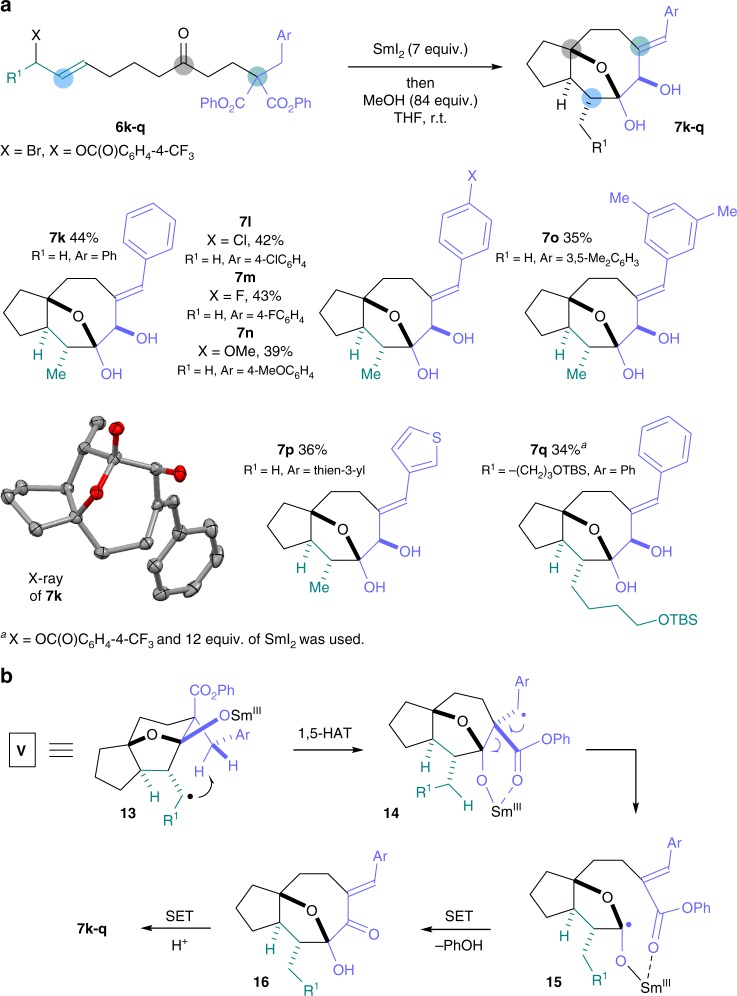
Folding radical cascades involving 1,5-HAT and activation of a benzylic group. A samarium(II) folding cascade involving activation of benzylic groups by 1,5-HAT in key radical intermediates **V** is shown. **a** The scope of the samarium(II) folding cascades involving benzylic group activation by 1,5-HAT to give products **7k**–**q**. **b** The mechanism of folding involves 1,5-HAT in key radical intermediates **V** to give **14** and subsequent fragmentation to give ketyl-type radicals **15**. Intramolecular acyloin reaction then delivers **16** that undergo diastereoselective reduction to give products **7k**–**q**

Importantly, the reductive SET conditions were also shown to be compatible with the presence of halide (formation of **7l** and **7m**), methoxy (formation of **7n**), and heteroaryl substituents (formation of **7p**). The carbon-halogen bonds in **7l** and **7m** provide useful handles for further functionalisation of the cascade products (Fig. 5a). The mechanism of the folding cascade is set out in Fig. 5b. After the initial Barbier cyclisation–lactonisation–lactone cyclisation sequence (see Fig. 2), 1,5-HAT in key radical intermediates **13** allows activation of the benzylic position to give radicals **14**. In contrast to the pathway involving tertiary radicals **9**, the benzylic radicals **14** undergo fragmentation, presumably promoted by the formation of an alkene that is in conjugation with the aromatic ring and the ester carbonyl. The resultant ketyl-type radicals **15** undergo acyloin-type cyclisation to deliver ketones **16** that are then diastereoselectively reduced (Fig. 5b).

### Mechanistic experiments

**Fig. 6:**
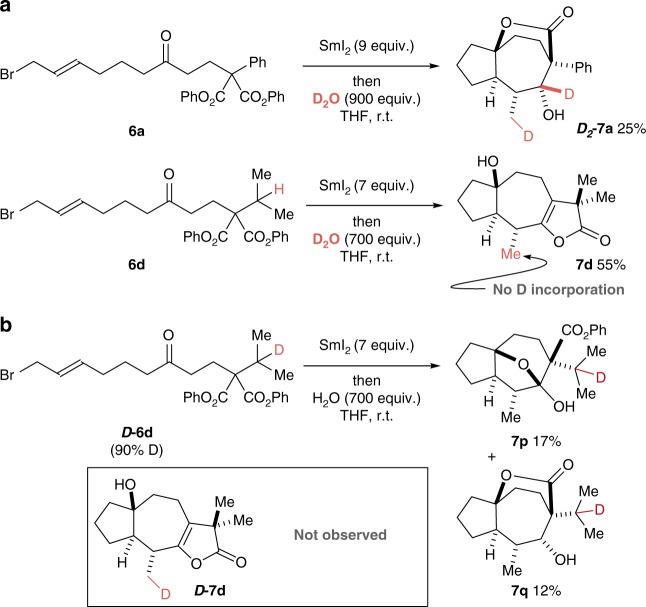
Mechanistic studies. Mechanistic studies involving the use of a labelled additive and a labelled substrate are shown. **a**, Reaction with SmI_2_–D_2_O resulted in the formation of deuterated product ***D***_***2***_-**7a**. This observation supports the operation of the extended SET mechanistic pathway when 1,5-HAT in **V** is not possible. Treatment of substrate **6d** with SmI_2_–D_2_O resulted in the formation of non-deuterated product **7d**. This reaction outcome supports the operation of a mechanism involving activation of a secondary alkyl group by 1,5-HAT. **b**, Use of deuterated substrate ***D***-**6d** in the reaction resulted in the formation of products **7p** and **7q** (product ***D***-**7d** was not formed): The presence of deuterium in ***D***-**6d** blocked 1,5-HAT and redirected folding

Mechanistic experiments involving a labelled additive and substrate have been used to probe the HAT pathways (Fig. 6). We first confirmed that a substrate lacking a substituent capable of HAT, substrate **6a**, undergoes cascade cyclisation to give labelled product, ***D***_***2***_**-7a**, when exposed to SmI_2_ and D_2_O (rather than H_2_O)^52^. This observation is consistent with radicals **V** being reduced and the resulting organosamariums protonated when HAT processes are not in operation. Furthermore, when substrate **6d**, bearing an iso-propyl substituent capable of undergoing HAT, was exposed to identical SmI_2_ and D_2_O conditions, no deuterium incorporation in the methyl group of product **7d** was observed. This suggests that the primary radical intermediate (cf. **V**) is not quenched by reduction/protonation but is instead quenched by intramolecular HAT.

To provide further support for 1,5-HAT, labelled substrate ***D*-6d** was prepared in which the tertiary hydrogen atom implicated in the HAT processes was exchanged for deuterium. Notably, upon treatment with SmI_2_ and H_2_O, cyclisation cascade products **7p** and **7q** (29%), rather than labelled **7d**, were isolated. As 1,5-deuterium transfer is known to be slower than 1,5-HAT^53^, the deuterium atom in ***D*-6d** blocks the final stages of the folding cascade and redirects the process. It is known that deuterium can be used as a ‘protecting group’ to prevent HAT processes and thus the outcome of the reaction of ***D*-6d** lends further support to the importance of such a process in the folding cascades^54^.

## Discussion

In summary, we have developed a folding cascade approach to complex polycyclic architectures bearing multiple stereocentres mediated by reductive SET from SmI_2_. The simple, linear substrates for folding are prepared in a modular, two-step synthesis and straightforward variation of substrate structure leads to three different folding pathways that deliver very different molecular architectures. Two of the folding pathways involve the use of 1,5-HAT to activate and functionalise otherwise inert secondary alkyl and benzylic groups in the substrates. Notably, 1,5-HAT is scarcely seen in the chemistry of SmI_2_ and rarely exploited and our studies suggest that the incorporation of 1,5-HAT in synthetic methods involving the SET reagent SmI_2_ can now be considered. In the context of diversity-oriented synthesis, the incorporation of 1,5-HAT processes in radical carbon–carbon bond-forming cascades has the potential to generate complexity and unlock access to diverse molecular structures in the search for new bioactive natural product-like compounds.

## Methods

### General experimental and characterization

Supplementary Figures 1–82 for the nuclear magnetic resonance spectra, Supplementary Tables 1–6 for X-ray crystallographic data, and Supplementary Methods giving full experimental details and the characterization of compounds are given in the Supplementary Information.

### Cascade involving an extended SET pathway

To a solution of SmI_2_ (9.00 mL, 0.90 mmol, 0.1 M in tetrahydrofuran (THF)), under nitrogen, δ-keto ester **6a’–c’** or **6a–c** (0.11 mmoL) in THF (0.70 mL) was added dropwise and the reaction mixture stirred for 14 h at room temperature. After that time, degassed H_2_O (2.20 mL, 122 mmoL) was added and the reaction was stirred at the same temperature for 24 h before being quenched with air, followed by saturated aqueous Rochelle’s salt and saturated aqueous sodium thiosulphate. The aqueous layer was extracted with Et_2_O (3 × 15 mL) and the combined organic layers were washed with brine (15 mL), dried over MgSO_4_, concentrated in vacuo, and purified by column chromatography eluting with EtOAc/hexane (5:95), to give compound **7a’–c’** or **7a–c**.

### Cascade involving 1,5-HAT and activation of a secondary alkyl group

To a solution of SmI_2_ (7.00 mL, 0.70 mmoL 0.1 M in THF), under nitrogen, malonate **6d–j** (0.10 mmoL) in THF (0.70 mL) was added dropwise and the reaction mixture stirred for 14 h at room temperature. After that time, degassed H_2_O (1.26 mL, 70.0 mmol) was added and the reaction was stirred at the same temperature for 24 h before being quenched with air, followed by saturated aqueous Rochelle’s salt and saturated aqueous sodium thiosulfate. The aqueous layer was extracted with Et_2_O (3 × 15 mL) and the combined organic layers were washed with brine (15 mL), dried over MgSO_4_, concentrated in vacuo, and purified by column chromatography eluting with EtOAc/hexane (5:95), to give compound **7d–j**.

### Cascade involving 1,5-HAT and activation of a benzylic group

To a solution of SmI_2_ (7.00 mL, 0.70 mmoL, 0.1 M in THF), malonate **6k–q** (0.10 mmoL) in THF (0.70 mL) was added dropwise under nitrogen and stirred for 14 h at room temperature. After that time, degassed MeOH (340 µL, 8.4 mmol) was added and the reaction was stirred at the same temperature for 48 h before being quenched with air followed by saturated aqueous Rochelle's salt and saturated aqueous sodium thiosulfate. The aqueous layer was extracted with Et_2_O (3 × 15 mL) and the combined organic layers were washed with brine (15 mL), dried over MgSO_4_, concentrated in vacuo, and purified by column chromatography eluting with Et_2_O/pentane (20:80), to give compound **7k–q**.

## Electronic supplementary material

Supplementary Information

## Data Availability

The X-ray crystallographic coordinates for **7a’**, **7d’**, **7a**, **7d**, **7****f**, and **7k** have been deposited at the Cambridge Crystallographic Data Centre (CCDC) under deposition numbers CCDC 1825583 and 1825585-89. These data can be obtained free of charge from the CCDC via www.ccdc.cam.ac.uk/data_request/cif. The authors declare that all other data supporting the findings of this study are available within the article and its Supplementary Information file.
