# Supplementary material for: Samarium(II) folding cascades involving hydrogen atom transfer for the synthesis of complex polycycles
Source: Nat Commun. 2018 Nov 15;9:4802. doi: 10.1038/s41467-018-07194-x (PMC6237924; doi:10.1038/s41467-018-07194-x)
Supplement: Supplementary file 1 — Supplementary Information [file 41467_2018_7194_MOESM1_ESM.pdf]

Samarium(II) folding cascades involving hydrogen atom transfer for the synthesis of complex polycycles

Plesniak, M. P. *et al.*

Correspondence to: david.j.procter@manchester.ac.uk

Supplementary Methods

All experiments were performed under a positive pressure of nitrogen using dry solvents unless stated otherwise. THF was distilled from sodium/benzophenone and CH_2Cl_2 was distilled from CaH_2. K_2CO_3 was oven-dried for 48 h at 140 °C. All other solvents and reagents were purchased from commercial sources and used as received. Reactions run at cryogenic temperatures for extended periods of time were cooled using a HAAKE® EK90 immersion cooler. ^{1}H NMR spectra were recorded at room temperature on a Bruker 400 or 500 MHz spectrometer. ^{13}C NMR spectra were recorded at 100 or 125 MHz, respectively. All NMR spectra are referenced from the residual nondeuterated solvent peak. Assignments were determined either on the basis of unambiguous chemical shifts or coupling patterns, COSY, HSQC, HMBC, NOESY experiments. Chemical shift values are reported in parts per million (ppm) on the δ scale, with coupling constants (*J*) reported in Hz. Splitting patterns are reported as follows: singlet (s), doublet (d), triplet (t), quartet (q), multiplet (m) and broad (b). Infrared spectra were recorded as evaporated films or neat using a FT/IR spectrometer, and wavelengths of maximum absorbance (ν_{max}) are quoted in wave numbers (cm^{-1}). Mass spectra were obtained from the Mass Spectroscopy Service of the University of Manchester using positive or negative electrospray (ESI) or atmospheric pressure chemical ionisation (APCI), and the parent ions $[M+H]^+$, $[M+Na]^+$ or $[M+NH_4]^+$ are quoted. Melting points were measured on solids as obtained after chromatography or recrystallization when appropriate and are uncorrected. Column chromatography was carried out using 40–63 μ, 60 Å silica gel and the procedure included the evaporation of solvents *in vacuo*. Routine TLC analysis was carried out on silica gel 60 F254 coated aluminium sheets of 0.2 mm thickness. Plates were visualised using a 254 nm ultraviolet lamp and developed by dipping in aqueous potassium permanganate solution or ethanolic phosphomolybdic acid solution followed by heating.

Ethyl 5-oxodec-9-enoate S1

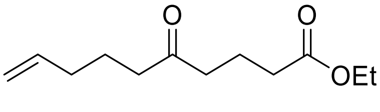

To a stirred solution of glutaric acid monoethyl ester chloride (3.50 mL, 22.0 mmol), and CuI (381 mg, 2.00 mmol) in THF (40 mL), pent-4-en-1-ylmagnesium bromide (46.0 mL, 20.0 mmol, 0.44 M in THF) was added during 1 h at –15 ℃. After addition was complete, reaction was stirred for an additional 1 h at –15 °C and quenched with saturated aqueous NH_4Cl. The phases were separated and the aqueous layer washed with Et_2O (3 × 20 mL). The combined

organic phases were washed with brine (20 mL), dried over $MgSO_4$ and concentrated *in vacuo* to give an orange oil which was purified by flash chromatography eluting with EtOAc/Hexane (3:97 to give the title compound as a yellow oil (3.73 g, 17.6 mmol, 88%). ^{1}H NMR (400 MHz, $CDCl_3$) δ 1.26 (t, *J* = 7.1, 3 H, $OCH_2C\mathit{H}_3$), 1.68 (p, *J* = 7.4 Hz, 2 H, $CH_2=CHCH_2C\mathit{H}_2CH_2$), 1.89 (p, *J* = 7.3 Hz, 2 H, $CH_2C\mathit{H}_2CH_2C(O)OEt$), 2.02 – 2.10 (m, 2 H, $CH_2=CHC\mathit{H}_2CH_2CH_2$), 2.32 (t, *J* = 7.3 Hz, 2 H, $CH_2CH_2C\mathit{H}_2C(O)OEt$), 2.41 (t, *J* = 7.5 Hz, 2 H, $CH_2=CHCH_2CH_2C\mathit{H}_2$), 2.47 (t, *J* = 7.2 Hz, 2 H, $C\mathit{H}_2CH_2CH_2C(O)OEt$), 4.13 (q, *J* = 7.2 Hz, 2 H, $OC\mathit{H}_2CH_3$), 4.93 – 5.06 (m, 2 H, $C\mathit{H}_2=CH$), 5.76 (ddt, *J* = 17.0, 10.2, 6.7 Hz, 1 H, $CH_2=C\mathit{H}$) ppm. ^{13}C NMR (100 MHz, $CDCl_3$) δ 14.2 ($OCH_2\mathit{C}H_3$), 18.9 ($CH_2\mathit{C}H_2CH_2C(O)OEt$), 22.8 ($CH_2=CHCH_2\mathit{C}H_2CH_2$), 33.1 ($CH_2=CH\mathit{C}H_2CH_2CH_2$), 33.3 ($CH_2CH_2\mathit{C}H_2C(O)OEt$), 41.6 ($CH_2=CHCH_2CH_2\mathit{C}H_2$), 41.9 ($\mathit{C}H_2CH_2CH_2C(O)OEt$), 60.4 ($O\mathit{C}H_2CH_3$), 115.2 ($\mathit{C}H_2=CH$), 137.9 ($CH_2=\mathit{C}H$), 173.2 ($\mathit{C}(O)OEt$), 210.1 ($\mathit{C}(O)$) ppm. IR ν_{max} (neat/cm^{-1}): 2938, 1731, 1712, 1640, 1446, 1414, 1373, 1178, 1095, 1029, 998, 911, 856, 775. HRMS calcd for $C_{12}H_{20}O_3Na$ $[M + Na]^+$: 235.1310, found: 235.1309.

Phenyl 5-oxodec-9-enoate S2

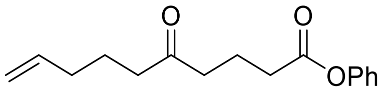

To a flask charged with ethyl 5-oxodec-9-enoate (1 g, 4.7 mmol) was added 2 M NaOH (4.71 mL, 9.4 mmol) and H_2O (2.5 mL) and stirred at room temperature for 2 h. The reaction mixture was diluted with H_2O (5 mL) and washed with hexane (2 × 10 mL). The organic layers were discarded and the aqueous phase acidified with 1 M HCl to approximately pH 1 and extracted with EtOAc (3 × 15 mL). The combined organic phases were washed with brine (15 mL), dried over $MgSO_4$ and concentrated *in vacuo* to give a white solid which was used in the next step without further purification. To a solution of the resulting crude carboxylic acid (0.42 g, 2.25 mmol) and phenol (0.21 g, 2.25 mmol) in CH_2Cl_2 (5 mL) was added EDCI and DMAP. After 16 h the reaction mixture was quenched with saturated aqueous $NaHCO_3$ and diluted with CH_2Cl_2. The layers were separated and the aqueous layer was extracted with CH_2Cl_2 (3 × 10 mL). The combined organic layers were washed with brine (10 mL), dried over $MgSO_4$, concentrated *in vacuo* and purified by column chromatography eluting with EtOAc/Hexane (5:98) to give the title compound as a colorless oil (0.39 g, 1.49 mmol, 67%). ^{1}H NMR (500 MHz, $CDCl_3$) δ 1.65 (p, *J* = 7.4 Hz, 2H, $CH_2=CHCH_2C\mathit{H}_2CH_2$), 2.00 (m, 4 H, $CH_2C\mathit{H}_2CH_2C(O)OPh$, $CH_2=CHC\mathit{H}_2CH_2CH_2$), 2.38 (t, *J* = 7.4 Hz, 2 H, $CH_2=CHCH_2CH_2C\mathit{H}_2$), 2.53 (m, 4 H, $CH_2CH_2C\mathit{H}_2C(O)OPh$, $C\mathit{H}_2CH_2CH_2C(O)OPh$), 4.90 –

5.00 (m, 2H, CH_2=CH), 5.72 (ddt, J = 16.9, 10.1, 6.7 Hz, 1H, CH$_2$=CH), 7.00 – 7.05 (m, 2H, ArCH), 7.15 – 7.21 (m, 1H, ArCH), 7.33 (t, J = 7.9 Hz, 2H, ArCH) ppm. ^{13}C NMR (125 MHz, CDCl$_3$) δ 19.0 (CH$_2$$CH_2CH_2$C(O)OPh), 23.0 (CH$_2$=CHCH$_2$$CH_2CH_2$), 33.2 (CH$_2$=CH$CH_2CH_2CH_2$), 33.5 (CH$_2CH_2$$CH_2$C(O)OPh), 41.5 (CH$_2$=CHCH$_2CH_2$$CH_2$), 42.1 ($CH_2CH_2CH_2$C(O)OPh), 115.4 (CH=$CH_2$), 121.6 (Ar$C$H), 125.9 (Ar$C$H), 129.5 (Ar$C$H), 138.0 (C$H$=CH$_2$), 150.8 (OAr$C$H), 171.8 ($C$(O)OAr), 210.1 ($C$(O)) ppm. IR ν_{max} (neat/cm^{-1}): 2936, 1756, 1711, 1640, 1593, 1493, 1456, 1413, 1374, 1311, 1162, 1131, 999, 915, 749, 690. HRMS calcd for $C_{16}H_{20}O_3Na$ [M + Na]$^+$: 283.1305, found 283.1291.

Octa-1,7-dien-3-one S3

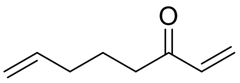

To an oven-dried, 3-neck round bottom flask, cooled under nitrogen, zinc dust (9.80 g, 150 mmol) and iodine (2.50 g, 10.0 mmol) were added and the flask flushed with nitrogen before the addition of dimethylacetamide (100 ml). After the decolourisation of the reaction mixture, 5-bromo-1-pentene (11.8 ml, 100 mmol) was added and the reaction was heated at 80 °C for 5 h. After cooling to room temperature, the reaction mixture was sparged with nitrogen followed by the addition of $Pd(PPh_3)_4$ (1.10 g, 1.00 mmol) and acryloyl chloride (8.89 ml, 110 mmol). After stirring over night at room temperature, the reaction was quenched with 0.05 M HCl (100 ml) and filtered. The aqueous phase was diluted with H_2O (200 mL) and extracted with Et_2O (3 × 100 mL). The combined organic phases were washed with saturated aqueous $NaHCO_3$ (50 mL), 10% aq. LiCl (50 mL) and brine (50 mL). The combined aqueous washes were back extracted with Et_2O (2 × 50 mL). The combined organic phases were dried over $MgSO_4$, concentrated *in vacuo* at room temperature and purified by column chromatography eluting with Et_2O/pentane (0:100 to 5:95) to give the product as a colourless oil (7.09 g, 57.0 mmol, 57%). ^{1}H NMR (400 MHz, CDCl$_3$) δ 1.74 (p, J = 7.4 Hz, 2 H, CH$_2$CH_2CH$_2$C(O)), 2.05 – 2.14 (m, 2 H, CH_2CH$_2$CH$_2$C(O)), 2.60 (t, J = 7.4 Hz, 2 H, CH$_2$CH$_2$CH_2C(O)), 4.95 – 5.07 (m, 2 H, CH_2=CHCH$_2$), 5.73 – 5.86 (m, 2 H, CH$_2$=CHCH$_2$, 1 H from C(O)CH=CH_{cis}H$_{trans}$), 6.22 (dd, J = 17.6, 1.2 Hz, 1 H, C(O)CH=CH$_{cis}$$H_{trans}$), 6.36 (dd, J = 17.6, 10.5 Hz, 1 H, C(O)CH=CH$_{cis}$H$_{trans}$). ^{13}C NMR (100 MHz, CDCl$_3$) δ 22.8 (CH$_2$$CH_2CH_2$C(O)), 33.0 ($CH_2CH_2CH_2$C(O)), 38.6 (CH$_2CH_2$$CH_2$C(O)), 115.3 ($CH_2$=CHCH$_2$), 127.9 (C(O)CH=$CH_2$),

136.5 (C(O)*C*H=CH_2), 137.8 (CH_2=*C*HCH_2), 200.4 (*C*(O)) ppm. Consistent with the literature.[1]

Dimethyl 2-cyclopentylmalonate S4

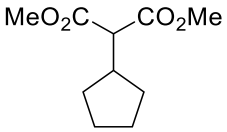

General procedure **A**. To a solution of dimethyl malonate (1.72 mL, 15.0 mmol) and cyclopentyl iodide (2.60 mL, 22.5 mmol) in DMSO (15 mL) was added KO*t*-Bu (2.02 g, 18.0 mmol) and the reaction stirred for 4 h at 100 °C. After cooling, the reaction was quenched with H_2O (10 mL) and acetic acid (4 mL). The organic layer was then extracted with Et_2O (3 × 20 mL). The combined organic phases were dried over $MgSO_4$, concentrated *in vacuo* and purified by column chromatography eluting with EtOAc/Hexane (4:96) to give the product as a colourless oil (2.30 g, 11.5 mmol, 77%). ^{1}H NMR (400 MHz, $CDCl_3$) δ 1.16 – 1.29 (m, 2 H, C*H*$_a$H$_b$$CH_2$$CH_2$*H*$_aH_b$), 1.51 – 1.70 (m, 4 H, CH_2C*H*$_2$C*H*$_2$$CH_2$), 1.79 – 1.91 (m, 2 H, CH$_a$*H*$_b$$CH_2$$CH_2CH_a$*H*$_b$), 2.42 – 2.56 (m, 1 H, C*H*CHC(O)OMe), 3.22 (d, *J* = 10.2 Hz, 1 H, CHC*H*C(O)OMe), 3.73 (s, 6 H, 2 × OC*H*$_3$) ppm. ^{13}C NMR (100 MHz, $CDCl_3$) δ 24.9 ($CH_2$$CH_2$*C*H$_2$$CH_2$), 30.8 ($CH_2$$CH_2$$CH_2$*C*H$_2$), 39.7 (*C*HCHC(O)OMe), 52.3 (O*C*H$_3$), 57.0 (CHC*H*C(O)OMe), 169.5 (C(O)) ppm. IR ν_{max} (neat/cm^{-1}): 2953, 2870, 1732, 1434, 1323, 1197, 1145, 1021, 895. HRMS calcd for $C_{10}H_{16}O_4K$ $[M + K]^+$: 239.0680, found 239.0679.

Dimethyl 2-cycloheptylmalonate S5

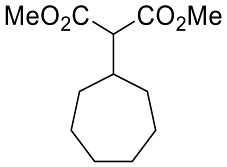

Prepared according to general procedure **A** using dimethylmalonate (800 μL, 7.00 mmol), cycloheptyl iodide (1.21 g, 5.39 mmol), KO*t*-Bu (726 mg, 6.47 mmol) and DMSO (6 mL) to give the title compound as a colourless oil (940 mg, 4.12 mmol, 76%). ^{1}H NMR (400 MHz, $CDCl_3$) δ 1.25 – 1.39 (m, 2 H, C*H*$_2$), 1.41 – 1.54 (m, 4 H, 2 × C*H*$_2$), 1.55 – 1.73 (m, 6 H, 3 × C*H*$_2$), 2.24 – 2.34 (m, 1 H, C*H*(CH_2)$_2$), 3.29 (d, *J* = 8.7 Hz, 1 H, C*H*CH(CH_2)$_2$), 3.73 (s, 6 H, 2 × OC*H*$_3$) ppm. ^{13}C NMR (100 MHz, $CDCl_3$) δ 26.4 (*C*H$_2$), 28.0 (*C*H$_2$), 31.9 (*C*H$_2$), 39.5 (*C*H(CH_2)$_2$), 52.3 (*C*HCH(CH_2)$_2$), 58.4 (O*C*H$_3$), 169.4 (*C*(O)O) ppm. IR ν_{max} (neat/cm^{-1}):

[1] Qian, M.; Covey, D. F. *Adv. Synth. Catal.* **2010**, *352* (11–12), 2057–2061.

2924, 2855, 1732, 1434, 1246, 1194, 1149, 1017. HRMS calcd for $C_{12}H_{20}O_4$ $[M + K]^+$: 267.0993, found 267.0992.

Dimethyl 2-(4-chlorobenzyl)malonate S6

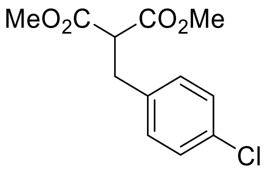

General procedure **B**. NaH (0.40 g, 10.0 mmol) was added to an oven dried flask under nitrogen atmosphere before suspension in THF (50 mL). The reaction mixture was cooled to 0 °C followed by dropwise addition of dimethyl malonate (2.30 mL, 20.0 mmol). The reaction mixture was allowed to warm to room temperature and was stirred for 30 min. After that time, the reaction mixture was cooled to 0 °C followed by addition of 1-(bromomethyl)-4-chlorobenzene (1.25 mL, 10.0 mmol). After stirring at room temperature for 14 h, the reaction mixture was quenched with H_2O (30 ml) and extracted with Et_2O (3 × 50 mL). The combined organic phases were then washed with brine (20 mL), dried with $MgSO_4$, concentrated *in vacuo* and purified by column chromatography eluting with EtOAc/toluene (2:98) to give the title compound as a colourless oil (1.99 g, 7.76 mmol, 78%). ^{1}H NMR (400 MHz, $CDCl_3$) δ 3.19 (d, J = 7.9 Hz, 2 H, CHCH_2), 3.63 (t, J = 7.9 Hz, 1 H, C*H*CH_2), 3,70 (s, 6 H, 2 × OCH_3), 7.11 (d, J = 8.5 Hz, 2 H, ArC*H*), 7.25 (d, J = 8.5 Hz, 2 H, ArC*H*). ^{13}C NMR (100 MHz, $CDCl_3$) δ 34.2 (CH*C*H_2), 52.8 (O*C*H_3), 53.6 (*C*HCH_2), 128.9 (Ar*C*H), 130.3 (Ar*C*H), 132.9 (Ar*C*), 136.3 (Ar*C*), 169.1 (*C*(O)O) ppm. IR ν_{max} (neat/cm^{-1}): 2954, 1733, 1492, 1435, 1410, 1345, 1283, 1230, 1151, 1093, 1065, 1016, 962, 813, 717, 668. HRMS calcd for $C_{12}H_{13}ClO_4K$ $[M+K]^+$: 295.0139, found 295.0134.

Dimethyl 2-(4-fluorobenzyl)malonate S7

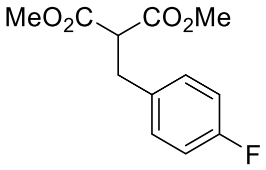

Prepared according to general procedure **B** using dimethyl malonate (2.30 mL, 20.0 mmol), 1-(bromomethyl)-4-fluorobenzene (2.05 g, 10.0 mmol), NaH (0.40 g, 10.0 mmol) and THF (50 mL) to give the title compound as a colourless oil (2.00 g, 8.33 mmol, 83%). ^{1}H NMR (400 MHz, $CDCl_3$) δ 3.19 (d, J = 7.8 Hz, 2 H, CHCH_2), 3.63 (t, J = 7.8 Hz, 1 H, C*H*CH_2), 3,70 (s, 6 H, 2 × OCH_3), 6.93 – 7.00 (m, 2 H, ArC*H*), 7.13 – 7.18 (m, 2 H, ArC*H*). ^{13}C NMR (100

MHz, $CDCl_3$) δ 34.1 (CH*C*H_2), 52.8 (O*C*H_3), 53.8 (*C*HCH_2), 115.6 (d, *J* = 21.2 Hz, Ar*C*H), 130.5 (d, *J* = 8.1 Hz, Ar*C*H), 133.5 (d, *J* = 3.4 Hz, Ar*C*), 161.9 (d, *J* = 244.8 Hz, Ar*C*), 169.2 (*C*(O)O) ppm. ^{19}F NMR (375 MHz, $CDCl_3$) δ -114.95 ppm. IR ν_{max} (neat/cm^{-1}): 2956, 1734, 1602, 1510, 1436, 1345, 1222, 1158, 1097, 1064, 1026, 961, 826, 763, 668. HRMS calcd for $C_{12}H_{13}FO_4K$ [M+K]$^+$: 279.0435, found 279.0429.

Dimethyl 2-(3,5-dimethylbenzyl)malonate S8

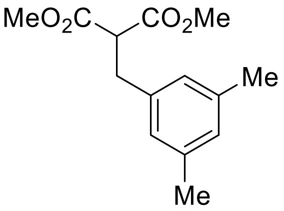

Prepared according to general procedure **B** using dimethyl malonate (2.30 mL, 20.0 mmol), 1-(bromomethyl)-3,5-dimethylbenzene (1.99 g, 10.0 mmol), NaH (0.40 g, 10.0 mmol) and THF (50 mL) to give the title compound as a colourless oil (2.14 g, 8.55 mmol, 86%). ^{1}H NMR (400 MHz, $CDCl_3$) δ 2.27 (s, 6 H, 2 × ArC-C*H_3*), 3.14 (d, *J* = 7.8 Hz, 2 H, CHC*H_2*), 3.65 (t, *J* = 7.8 Hz, 1 H, C*H*CH_2), 3,71 (s, 6 H, 2 × OC*H_3*), 6.80 (s, 2 H, ArC*H*), 6.85 (s, 1 H, ArC*H*). ^{13}C NMR (100 MHz, $CDCl_3$) δ 21.4 (ArC-*C*H_3), 34.8 (CH*C*H_2), 52.7 (O*C*H_3), 53.8 (*C*HCH_2), 126.7 (Ar*C*H), 128.6 (Ar*C*H), 137.8 (Ar*C*), 138.2 (Ar*C*), 169.5 (*C*(O)O) ppm. IR ν_{max} (neat/cm^{-1}): 2953, 1733, 1607, 1435, 1345, 1269, 1227, 1199, 1148, 1067, 1030, 963, 853, 697, 668. HRMS calcd for $C_{14}H_{18}O_4Na$ [M+Na]$^+$: 273.1103, found 273.1096.

Dimethyl 2-(4-methoxybenzyl)malonate (S9)

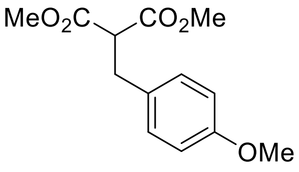

Prepared according to general procedure **B** using dimethyl malonate (4.38 mL, 38.3 mmol), 1-(chloromethyl)-4-methoxybenzene (3 g, 19.2 mmol), NaH (0.46 g, 19.2 mmol) and THF (96 mL) to give the title compound as a colourless oil (0.7519 g, 2.98 mmol, 16%). ^{1}H NMR (500 MHz, $CDCl_3$) δ 3.18 (d, *J* = 7.8 Hz, 2 H, CHC*H_2*), 3.65 (t, *J* = 7.7 Hz, 1 H, C*H*CH_2), 3.72 (s, 6 H, 2 × OC*H_3*), 3.80 (s, 3 H, ArOC*H_3*), 6.84 (d, *J* = 8.2 Hz, 2 H, ArC*H*), 7.13 (d, *J* = 8.2 Hz, 2 H, ArC*H*). ^{13}C NMR (126 MHz, $CDCl_3$) δ 34.1 (CH*C*H_2), 52.7 (O*C*H_3), 54.0 (*C*HCH_2), 55.3 (ArO*C*H_3), 114.1(Ar*C*H), 129.8 (Ar*C*), 129.9 (Ar*C*H), 158.5 (Ar*C*), 169.4 (*C*(O)O). IR

ν_{max} (neat/cm^{-1}): 2954, 2361, 1732, 1435, 1344, 1245, 1177, 1149, 1030, 912, 821, 731. HRMS calcd for $C_{13}H_{17}O_5$[M + H]$^+$: 253.1071, found 253.1065.

Diethyl 2-(propan-2-yl-2-d)malonate S10

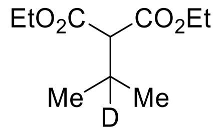

To a suspension of $NaBD_4$ (300 mg, 7.16 mmol) in EtOH (4 mL), diethyl isopropylidenemalonate (1.43 g, 7.16 mmol) was added dropwise at 0 °C. The reaction was allowed to warm to room temperature and stirred for 16 h before being quenched with brine (40 mL) and extracted with Et_2O (4 × 30 mL). The combined organic phases were then dried over $MgSO_4$, concentrated *in vacuo* and purified by column chromatography eluting with Et_2O/Pentane (5:95) to give a colourless oil (752 mg, 4.30 mmol, 60%). ^{1}H NMR (400 MHz, $CDCl_3$) δ 1.00 (s, 6 H, $CD(CH_3)_2$), 1.27 (t, J = 7.1 Hz, 6 H, OCH_2CH_3), 3.11 (s, 1 H, $CHCD(CH_3)_2$), 4.20 (q, J = 7.1 Hz, 4 H, OCH_2CH_3) ppm. ^{13}C NMR (100 MHz, $CDCl_3$) δ 14.1 ($CD(CH_3)_2$), 20.4 (OCH_2CH_3), 28.5 (1:1:1 t, J = 20.1 Hz, ($CD(CH_3)_2$)), 59.3 ($CHCD(CH_3)_2$), 61.1 (OCH_2CH_3), 168.9 (C(O)O) ppm. IR ν_{max} (neat/cm^{-1}): 2965, 2360, 1729, 1465, 1446, 1390, 1368, 1304, 1229, 1184, 1128, 1095, 1034, 860, 668. HRMS calcd for $C_{10}H_{17}DO_4K$ [M + K]$^+$: 242.0899, found 242.0898.

Diphenyl 2-isopropylmalonate S11

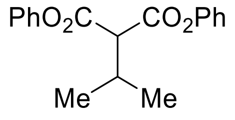

General procedure **C**. To a flask charged with diethyl isopropylmalonate (2.02 g, 10.0 mmol) was added 2 M NaOH (13.5 mL, 27.0 mmol) and the solution heated at reflux for 3 h. After cooling, the reaction mixture was diluted with H_2O (10 mL) and washed with hexane (2 × 20 mL). The organic layers were then discarded and the aqueous phase acidified with 1 M HCl to approximately pH 1 and extracted with EtOAc (3 × 30 mL). The combined organic phases were washed with brine (20 mL), dried over $MgSO_4$ and concentrated *in vacuo* to give a white solid which was used in the next step without further purification. Crude diacid was suspended in dry CH_2Cl_2 (30 mL) under nitrogen and cooled to 0 ℃ followed by the dropwise addition of few drops of DMF and oxalyl chloride (2.57 mL, 30.0 mmol). The reaction was stirred for 3 h at room temperature with venting before being concentrated *in vacuo* to give a yellow oil. Phenol (1.88 g, 20.0 mmol) and DMAP (122 mg, 1 mmol) were then added, the flask flushed

with nitrogen and CH_2Cl_2 (30 mL) added. The reaction mixture was cooled to 0 ℃ followed by the addition of Et_3N (5.58 mL, 40.0 mmol). After stirring at room temperature for 14 h, the reaction mixture was diluted with CH_2Cl_2 (30 mL) and washed with H_2O (2 × 20 ml), sat. $NaHCO_3$ (20 mL) and brine (20 mL). The organic phase was dried over $MgSO_4$, concentrated *in vacuo* and purified by column chromatography eluting with EtOAc/hexane (5:95) to give a white solid (2.15 g, 7.20 mmol, 72% over 3 steps). ^{1}H NMR (400 MHz, $CDCl_3$) δ 1.24 (d, *J* = 6.8 Hz, 6 H, CH(C*H*$_3$)$_2$), 2.62 – 2.72 (m, 1 H, C*H*(CH$_3$)$_2$), 3.65 (d, *J* = 8.0 Hz, 1 H, C*H*CH(CH$_3$)$_2$), 7.12 – 7.18 (m, 4 H, ArC*H*), 7.25 – 7.29 (m, 2 H, ArC*H*), 7.38 – 7.44 (m, 4 H, ArC*H*) ppm. ^{13}C NMR (100 MHz, $CDCl_3$) δ 20.5 (CH(*C*H$_3$)$_2$), 28.9 (*C*H(CH$_3$)$_2$), 58.7 (*C*HCH(CH$_3$)$_2$), 121.4 (Ar*C*H), 126.2 (Ar*C*H), 129.6 (Ar*C*H), 150.5 (Ar*C*), 167.1 (*C*(O)O) ppm. IR ν_{max} (neat/cm^{-1}): 2968, 1774, 1751, 1589, 1484, 1471, 1456, 1342, 1286, 1150, 1107, 1069, 1019, 981, 970, 950, 929, 912, 819, 751. M.p. ($CHCl_3$) = 52 – 53 ˚C. HRMS calcd for $C_{18}H_{18}O_4K$ [M + K]$^+$: 337.0837, found 337.0833.

Diphenyl 2-cyclopentylmalonate S12

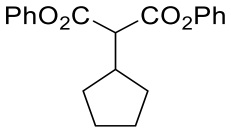

Prepared according to general procedure **C** using dimethyl 2-cyclopentylmalonate (1.00 g, 5.00 mmol), 2 M NaOH (6.80 mL, 13.5 mmol), oxalyl chloride (1.29 mL, 15.0 mmol), phenol (940 mg, 10.0 mmol), DMAP (61 mg, 0.50 mmol), Et_3N (2.79 mL, 20.0 mmol) and CH_2Cl_2 (15 mL) to give the title compound as a white solid (1.33 g, 4.11 mmol, 82% over 3 steps). ^{1}H NMR (400 MHz, $CDCl_3$) δ 1.46 – 1.57 (m, 2 H, C*H*$_2$), 1.61 – 1.82 (m, 4 H, 2 × C*H*$_2$), 2.04 – 2.14 (m, 2 H, C*H*$_2$), 2.70 – 2.81 (m, 1 H C*H*(CH$_2$)$_2$), 3.71 (d, *J* = 10.0 Hz, 1 H, C*H*CH(CH$_2$)$_2$), 7.12 – 7.19 (m, 4 H, ArC*H*), 7.24 – 7.31 (m, 2 H, ArC*H*), 7.38 – 7.45 (m, 4 H, ArC*H*) ppm. ^{13}C NMR (100 MHz, $CDCl_3$) δ 25.1 (*C*H$_2$), 30.9 (*C*H$_2$), 39.6 (*C*H(CH$_2$)$_2$), 57.3 (*C*HCH(CH$_2$)$_2$), 121.4 (Ar*C*H), 126.2 (Ar*C*H), 129.6 (Ar*C*H), 150.5 (Ar*C*), 167.3 (*C*(O)O) ppm. IR ν_{max} (neat/cm^{-1}): 2954, 2869, 1773, 1752, 1591, 1492, 1456, 1323, 1294, 1187, 1161, 1121, 1070, 1024, 1005, 971, 924, 904, 821, 745, 687. M.p. ($CHCl_3$) = 42 – 43 ˚C. HRMS calcd for $C_{20}H_{20}O_4K$ [M + K]$^+$: 363.0993, found 363.0962.

Diphenyl 2-cyclohexylmalonate S13

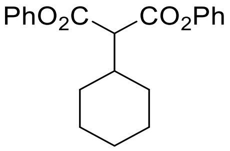

Prepared according to general procedure **C** using diethyl 2-cyclohexylmalonate (1.21 g, 5.00 mmol), 2 M NaOH (6.80 mL, 13.5 mmol), oxalyl chloride (1.29 mL, 15.0 mmol), phenol (940 mg, 10.0 mmol), DMAP (61 mg, 0.50 mmol), Et_3N (2.79 mL, 20.0 mmol) and CH_2Cl_2 (15 mL) to give the title compound as a white solid (1.27 g, 3.75 mmol, 75% over 3 steps). ^{1}H NMR (400 MHz, $CDCl_3$) δ 1.18 – 1.47 (m, 5 H, 1 H from C*H*$_2$, 4 H from 2 × C*H*$_2$), 1.70 – 1.78 (m, 1 H, 1 H from C*H*$_2$), 1.80 – 1.88 (m, 2 H, C*H*$_2$), 1.94 – 2.02 (m, 2 H, C*H*$_2$), 2.32 – 2.43 (m, 1 H, C*H*$(CH_2)_2$), 3.68 (d, *J* = 8.4 Hz, 1 H, C*H*CH$(CH_2)_2$), 7.11 – 7.19 (m, 4 H, ArC*H*), 7.24 – 7.30 (m, 2 H, ArC*H*), 7.37 – 7.45 (m, 4 H, ArC*H*) ppm. ^{13}C NMR (100 MHz, $CDCl_3$) δ 26.0 (*C*H$_2$), 26.0 (*C*H$_2$), 30.8 (*C*H$_2$), 38.1 (*C*H$(CH_2)_2$), 58.1 (*C*HCH$(CH_2)_2$), 121.4 (Ar*C*H), 126.2 (Ar*C*H), 129.6 (Ar*C*H), 150.5 (Ar*C*), 167.0 (*C*(O)O) ppm. IR ν_{max} (neat/cm^{-1}): 2927, 2853, 2358, 1773, 1751, 1591, 1492, 1449, 1292, 1190, 1162, 1116, 1069, 1024, 962, 744, 687. M.p. ($CHCl_3$) = 44 – 46 ˚C. HRMS calcd for $C_{21}H_{22}O_4K$ $[M + K]^+$: 377.1150, found 377.1150.

Diphenyl 2-cycloheptylmalonate S14

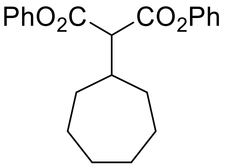

Prepared according to general procedure **C** using dimethyl 2-cycloheptylmalonate (1.14 g, 5.00 mmol), 2 M NaOH (6.80 mL, 13.5 mmol), oxalyl chloride (1.29 mL, 15.0 mmol), phenol (940 mg, 10.0 mmol), DMAP (61 mg, 0.50 mmol), Et_3N (2.79 mL, 20.0 mmol) and CH_2Cl_2 (15 mL) to give the title compound as a white solid (1.33 g, 3.79 mmol, 76% over 3 steps). ^{1}H NMR (400 MHz, $CDCl_3$) δ 1.53 – 1.72 (m, 8 H, 4 × C*H*$_2$), 1.74 – 1.85 (m, 2 H, C*H*$_2$), 1.91 – 2.01 (m, 2 H, C*H*$_2$), 2.51 – 2.61 (m, 1 H, C*H*$(CH_2)_2$), 3.79 (d, *J* = 7.7 Hz, 1 H, C*H*CH$(CH_2)_2$), 7.12 – 7.18 (m, 4 H, ArC*H*), 7.25 – 7.30 (m, 2 H, ArC*H*), 7.38 – 7.45 (m, 4 H, ArC*H*) ppm. ^{13}C NMR (100 MHz, $CDCl_3$) δ 26.7 (*C*H$_2$), 28.0 (*C*H$_2$), 32.1 (*C*H$_2$), 39.6 (*C*H$(CH_2)_2$), 58.5 (*C*HCH$(CH_2)_2$), 121.4 (Ar*C*H), 126.2 (Ar*C*H), 129.6 (Ar*C*H), 150.5 (Ar*C*), 167.3 (*C*(O)O) ppm. IR ν_{max} (neat/cm^{-1}): 2926, 2857, 1774, 1750, 1591, 1492, 1457, 1341, 1294, 1188, 1161, 1118, 1069, 1023, 1005, 963, 828, 743, 687. M.p. ($CHCl_3$) = 38 – 39 ˚C. HRMS calcd for $C_{22}H_{24}O_4Na$ $[M + Na]^+$: 375.1567, found 375.1571.

Diphenyl 2-methylmalonate S15

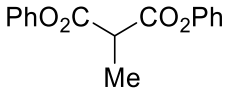

Prepared according to general procedure **C** using diethyl 2-methylmalonate (1.74 g, 10.0 mmol), 2 M NaOH (13.5 mL, 27.0 mmol), oxalyl chloride (2.57 mL, 30.0 mmol), phenol (1.88 g, 20.0 mmol), DMAP (122 mg, 1.00 mmol), Et_3N (5.58 mL, 40.0 mmol) and CH_2Cl_2 (30 mL) to give the title compound as a white solid (1.38 g, 5.10 mmol, 51% over 3 steps). ^{1}H NMR (400 MHz, $CDCl_3$) δ 1.71 (d, *J* = 7.3 Hz, 3 H, $CHCH_3$), 3.94 (q, *J* = 7.3 Hz, 1 H, C*H*CH_3), 7.13 – 7.19 (m, 4 H, ArC*H*), 7.24 – 7.31 (m, 2 H, ArC*H*), 7.37 – 7.46 (m, 4 H, ArC*H*) ppm. ^{13}C NMR (100 MHz, $CDCl_3$) δ 13.6 (CH*C*H_3), 46.4 (*C*HCH_3), 121.3 (Ar*C*H), 126.3 (Ar*C*H), 129.6 (Ar*C*H), 150.5 (Ar*C*), 168.4 (*C*(O)O) ppm. IR ν_{max} (neat/cm^{-1}): 1749, 1591, 1492, 1456, 1330, 1187, 1160, 1134, 1068, 1023, 1005, 927, 745, 687. M.p. ($CHCl_3$) = 40 – 42 ˚C. HRMS calcd for $C_{16}H_{14}O_4K$ [M + K]$^+$: 309.0524, found 309.0520.

Diphenyl 2-ethylmalonate S16

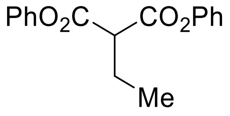

Prepared according to general procedure **C** using diethyl 2-ethylmalonate (1.89 g, 10.0 mmol), 2 M NaOH (13.5 mL, 27.0 mmol), oxalyl chloride (2.57 mL, 30.0 mmol), phenol (1.88 g, 20.0 mmol), DMAP (122 mg, 1.00 mmol), Et_3N (5.58 mL, 40.0 mmol) and CH_2Cl_2 (30 mL) to give the title compound as a white solid (1.70 g, 6.00 mmol, 60% after 3 steps). ^{1}H NMR (400 MHz, $CDCl_3$) δ 1.20 (t, *J* = 7.5 Hz, 3 H, CH_2CH_3), 2.18 – 2.28 (m, 2 H, $CHCH_2CH_3$), 3.78 (t, *J* = 7.4 Hz, 1 H, C*H*CH_2CH_3), 7.11 – 7.19 (m, 4 H, ArC*H*), 7.24 – 7.31 (m, 2 H, ArC*H*), 7.37 – 7.45 (m, 4 H, ArC*H*) ppm. ^{13}C NMR (100 MHz, $CDCl_3$) δ 11.9 (CH_2CH_3), 22.3 ($CHCH_2CH_3$), 53.5 (*C*HCH_2CH_3), 121.4 (Ar*C*H), 126.3 (Ar*C*H), 129.6 (Ar*C*H), 150.5 (Ar*C*), 167.7 (*C*(O)O) ppm. IR ν_{max} (neat/cm^{-1}): 2971, 1770, 1750, 1591, 1492, 1457, 1349, 1286, 1252, 1186, 1160, 1132, 1070, 1023, 1006, 943, 905, 882, 821, 744, 687. M.p. ($CHCl_3$) = 25 – 27 ˚C. HRMS calcd for $C_{17}H_{16}O_4K$ [M + K]$^+$: 323.0680, found 323.0678.

Diphenyl 2-phenylmalonate S17

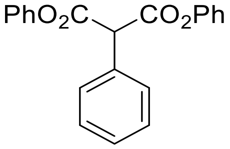

Prepared according to general procedure **C** using diethyl 2-phenylmalonate (2.36 g, 10.0 mmol), 2 M NaOH (13.5 mL, 27.0 mmol), oxalyl chloride (2.57 mL, 30.0 mmol), phenol (1.88 g, 20.0 mmol), DMAP (122 mg, 1.00 mmol), Et_3N (5.58 mL, 40.0 mmol) and CH_2Cl_2 (30 mL) to give the title compound as a white solid (1.97 g, 6.12 mmol, 61% over 3 steps). ^{1}H NMR (400 MHz, $CDCl_3$) δ 5.16 (s, 1 H, C*H*Ph), 7.14 – 7.21 (m, 4 H, ArC*H*), 7.25 – 7.32 (m, 2 H, ArC*H*), 7.39 – 7.53 (m, 7 H, ArC*H*), 7.61 – 7.67 (m, 2 H, ArC*H*) ppm. ^{13}C NMR (100 MHz, $CDCl_3$) δ 58.0 (*C*HPh), 121.3 (Ar*C*H), 126.3 (Ar*C*H), 128.8 (Ar*C*H), 129.0 (Ar*C*H), 129.5 (Ar*C*H), 129.6 (Ar*C*H), 131.8 (Ar*C*), 150.6 (Ar*C*-O), 166.5 (*C*(O)O) ppm. IR ν_{max} (neat/cm^{-1}): 3065, 2358, 1751, 1590, 1491, 1456, 1306, 1186, 1161, 1120, 1070, 1023, 1004, 972, 907, 835, 744, 722, 687. M.p. ($CHCl_3$) = 63 – 65 ˚C. HRMS calcd for $C_{21}H_{16}O_4Na$ [M + Na]$^+$: 355.0941, found 355.0944.

Diphenyl 2-benzylmalonate S18

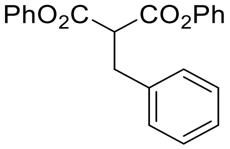

Prepared according to general procedure **C** using dimethyl 2-benzylmalonate (2.22 g, 10.0 mmol), 2 M NaOH (13.5 mL, 27.0 mmol), oxalyl chloride (2.57 mL, 30.0 mmol), phenol (1.88 g, 20.0 mmol), DMAP (122 mg, 1.00 mmol), Et_3N (5.58 mL, 40.0 mmol) and CH_2Cl_2 (30 mL) to give the title compound as a white solid (2.46 g, 7.10 mmol, 71% over 3 steps). ^{1}H NMR (400 MHz, $CDCl_3$) δ 3.51 (d, *J* = 7.9 Hz, 2 H, CHC*H*$_2$Ph), 4.20 (t, *J* = 7.9 Hz, 1 H, C*H*CH$_2$Ph), 7.03 – 7.08 (m, 4 H, ArC*H*), 7.24 – 7.30 (m, 2 H, ArC*H*), 7.30 – 7.35 (m, 1 H, ArC*H*), 7.35 – 7.43 (m, 8 H, ArC*H*) ppm. ^{13}C NMR (100 MHz, $CDCl_3$) δ 34.8 (CH*C*H$_2$Ph), 53.9 (*C*HCH$_2$Ph), 121.3 (Ar*C*H), 126.3 (Ar*C*H), 127.2 (Ar*C*H), 128.8 (Ar*C*H), 129.1 (Ar*C*H), 129.6 (Ar*C*H), 137.2 (Ar*C*), 150.4 (Ar*C*-O), 167.2 (*C*(O)O) ppm. IR ν_{max} (neat/cm^{-1}): 3029, 2358, 1752, 1591, 1491, 1455, 1337, 1272, 1186, 1161, 1126, 1069, 1023, 971, 917, 829, 744, 700, 687, 621. M.p. ($CHCl_3$) = 47 – 49 ˚C. HRMS calcd for $C_{22}H_{18}O_4K$ [M+K]$^+$: 385.0837, found 385.0836.

Diphenyl 2-(4-chlorobenzyl)malonate S19

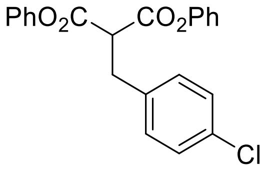

Prepared according to general procedure **C** using dimethyl 2-(4-chlorobenzyl)malonate (1.89 g, 7.35 mmol), 2 M NaOH (9.90 mL, 19.8 mmol), oxalyl chloride (1.80 mL, 21.0 mmol), phenol (1.13 g, 12.0 mmol), DMAP (73 mg, 0.60 mmol), Et_3N (2.10 mL, 15.0 mmol) and CH_2Cl_2 (29 mL) to give the title compound as a white solid (1.44 g, 3.80 mmol, 52% after 3 steps). ^{1}H NMR (400 MHz, $CDCl_3$) δ 3.44 (d, *J* = 7.8 Hz, 2 H, CHC*H*$_2$), 4.12 (t, *J* = 7.8 Hz, 1 H, C*H*CH$_2$), 7.04 (d, *J* = 8.0 Hz, 4 H, ArC*H*), 7.24 – 7.36 (m, 6 H, ArC*H*), 7.39 (t, *J* = 8.0 Hz, 4 H, ArC*H*) ppm. ^{13}C NMR (100 MHz, $CDCl_3$) δ 34.1 (CH*C*H$_2$), 53.8 (*C*HCH$_2$), 121.4 (Ar*C*H), 126.5 (Ar*C*H), 129.1 (Ar*C*H), 129.8 (Ar*C*H), 130.6 (Ar*C*H), 133.2 (Ar*C*), 135.8 (Ar*C*), 150.4 (Ar*C*-O), 167.1 (*C*(O)O) ppm. IR ν_{max} (neat/cm^{-1}): 1750, 1591, 1491, 1270, 1185, 1161, 1126, 1103, 1092, 1070, 1023, 1015, 806, 745, 686, 668. M.p. ($CHCl_3$) = 45 – 46 ˚C. HRMS calcd for $C_{22}H_{17}ClO_4Na$ [M+Na]$^+$: 403.0713, found 403.0708.

Diphenyl 2-(4-fluorobenzyl)malonate S20

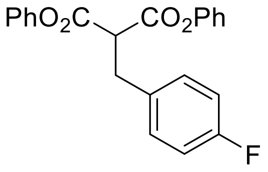

Prepared according to general procedure **C** using dimethyl 2-(4-fluorobenzyl)malonate (1.86 g, 7.72 mmol), 2 M NaOH (10.4 mL, 20.8 mmol), oxalyl chloride (1.97 mL, 22.9 mmol), phenol (1.23 g, 13.1 mmol), DMAP (80 mg, 0.66 mmol), Et_3N (2.30 mL, 16.4 mmol) and CH_2Cl_2 (32 mL) to give the title compound as a white solid (1.69 g, 4.64 mmol, 60% after 3 steps). ^{1}H NMR (400 MHz, $CDCl_3$) δ 3.45 (d, *J* = 7.8 Hz, 2 H, CHC*H*$_2$), 4.12 (t, *J* = 7.8 Hz, 1 H, C*H*CH$_2$), 7.01 – 7.08 (m, 6 H, ArC*H*), 7.24 – 7.29 (m, 2 H, ArC*H*), 7.30 – 7.35 (m, 2 H, ArC*H*), 7.39 (t, *J* = 7.9 Hz, 4 H, ArC*H*) ppm. ^{13}C NMR (100 MHz, $CDCl_3$) δ 34.0 (CH*C*H$_2$), 54.1 (*C*HCH$_2$), 115.8 (d, *J* = 21.2 Hz, Ar*C*H), 121.4 (Ar*C*H), 126.5 (Ar*C*H), 129.7 (Ar*C*H), 130.8 (d, *J* = 8.1 Hz, Ar*C*H), 132.9 (d, *J* = 3.4 Hz, Ar*C*), 150.5 (Ar*C*-O) 162.2 (d, *J* = 246.0 Hz, Ar*C*), 167.2 (*C*(O)O) ppm. ^{19}F NMR (375 MHz, $CDCl_3$) δ -115.44 ppm. IR ν_{max} (neat/cm^{-1}): 1750, 1591, 1510, 1492, 1272, 1220, 1185, 1159, 1125, 1096, 1070, 1024, 826, 745, 687, 668.

M.p. ($CHCl_3$) = 44 – 45 ˚C. HRMS calcd for $C_{22}H_{17}FO_4Na$ $[M+Na]^+$: 387.1009, found 387.1004.

Diphenyl 2-(3,5-dimethylbenzyl)malonate S21

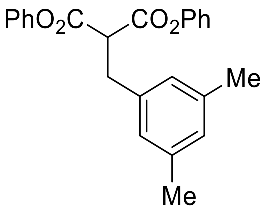

Prepared according to general procedure **C** using dimethyl 2-(3,5-dimethylbenzyl)malonate (1.95 g, 7.80 mmol), 2 M NaOH (10.6 mL, 21.1 mmol), oxalyl chloride (1.70 mL, 20.0 mmol), phenol (1.25 g, 13.3 mmol), DMAP (81 mg, 0.67 mmol), Et_3N (2.34 mL, 16.6 mmol) and CH_2Cl_2 (20 mL) to give the title compound as a white solid (1.62 g, 4.32 mmol, 55% after 3 steps). ^{1}H NMR (400 MHz, $CDCl_3$) δ 2.32 (s, 6 H, 2 × ArC-CH_3), 3.40 (d, J = 7.8 Hz, 2 H, CHCH_2), 4.13 (t, J = 7.8 Hz, 1 H, C*H*CH_2), 6.93 (s, 2 H, ArC*H*), 6.97 (s, 1 H, ArC*H*), 7.03 – 7.05 (d, 4 H, J = 8.0 Hz, ArC*H*), 7.22 – 7.28 (m, 2 H, ArC*H*), 7.39 (t, J = 8.0 Hz, 4 H, ArC*H*) ppm. ^{13}C NMR (100 MHz, $CDCl_3$) δ 21.4 (ArC-*C*H_3), 34.8 (CH*C*H_2), 54.1 (*C*HCH_2), 121.5 (Ar*C*H), 126.4 (Ar*C*H), 127.0 (Ar*C*H), 128.9 (Ar*C*H), 129.7 (Ar*C*H), 137.1 (Ar*C*), 138.4 (Ar*C*), 150.6 (Ar*C*-O), 167.4 (*C*(O)O) ppm. IR ν_{max} (neat/cm^{-1}): 1750, 1591, 1491, 1186, 1160, 1123, 1069, 1024, 849, 745, 687, 668. M.p. ($CHCl_3$) = 56 – 58 ˚C. HRMS calcd for $C_{24}H_{22}O_4Na$ $[M+Na]^+$: 397.1416, found 397.1411.

Diphenyl 2-(4-methoxybenzyl)malonate S22

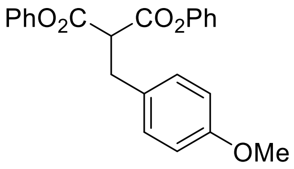

Prepared according to general procedure **C** using dimethyl 2-(4-methoxybenzyl)malonate (0.75 g, 2.98 mmol), 2 M NaOH (4.0 mL, 8.05 mmol), oxalyl chloride (0.66 mL, 7.75 mmol), phenol (0.49 g, 5.17 mmol), DMAP (31 mg, 0.25 mmol), Et_3N (1.10 mL, 7.75 mmol) and CH_2Cl_2 (20 mL) to give the title compound as a colourless oil (0.54 g, 1.43 mmol, 48% after 3 steps). ^{1}H NMR (400 MHz, $CDCl_3$) δ 3.34 (d, J = 7.9 Hz, 2 H, CHCH_2), 3.74 (s, 3 H, 2 × OCH_3), 4.04 (t, J = 7.9 Hz, 1 H, C*H*CH_2), 6.82 (d, J = 8.7 Hz, 2 H, ArC*H*), 6.92 – 7.00 (m, 4 H, ArC*H*), 7.13 – 7.22 (m, 4 H, ArC*H*), 7.26 – 7.34 (m, 4 H, ArC*H*). ^{13}C NMR (101 MHz, $CDCl_3$) δ 34.1 (CH*C*H_2), 54.3 (*C*HCH_2), 55.4 (O*C*H_3), 114.3 (Ar*C*H), 121.4 (Ar*C*H), 126.4 (Ar*C*H), 129.2

(Ar*C*), 129.7 (Ar*C*H), 130.3 (Ar*C*H), 150.5 (Ar*C*), 158.9 (Ar*C*), 167.3 (*C*(O)O). IR ν_{max} (neat/cm^{-1}): 2933, 1750, 1591, 1513, 1492, 1344, 1160, 1125, 1032, 824, 745, 688. HRMS calcd for $C_{23}H_{20}O_5Na$ [M + Na]$^+$: 399.1203, found 399.1200.

Diphenyl 2-(propan-2-yl-2-d)malonate S23

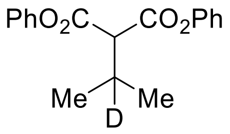

Prepared according to general procedure **C** using diethyl 2-(propan-2-yl-2-d)malonate (700 mg, 3.50 mmol), 2 M NaOH (4.70 mL, 9.45 mmol), oxalyl chloride (0.91 mL, 10.5 mmol), phenol (658 mg, 7.00 mmol), DMAP (43 mg, 0.35 mmol), Et_3N (1.95 mL, 14.0 mmol) and CH_2Cl_2 (11 mL) to give the title compound as a white solid (700 mg, 2.34 mmol, 67% over 3 steps). ^{1}H NMR (400 MHz, $CDCl_3$) δ 1.25 (s, 6 H, CD(C*H*$_3$)$_2$), 3.66 (s, 1 H, C*H*CD(CH$_3$)$_2$), 7.13 – 7.19 (m, 4 H, ArC*H*), 7.24 – 7.30 (m, 2 H, ArC*H*), 7.38 – 7.45 (m, 4 H, ArC*H*) ppm. ^{13}C NMR (100 MHz, $CDCl_3$) δ 20.4 (CD(*C*H$_3$)$_2$), 28.6 (1:1:1 t, *J* = 20.3 Hz, *C*D(CH$_3$)$_2$), 58.6 (*C*HCD(CH$_3$)$_2$), 121.4 (Ar*C*H), 126.3 (Ar*C*H), 129.6 (Ar*C*H), 150.5 (Ar*C*), 167.1 (*C*(O)O) ppm. IR ν_{max} (neat/cm^{-1}): 2976, 1773, 1750, 1589, 1484, 1469, 1457, 1394, 1373, 1306, 1225, 1186, 1175, 1159, 1150, 1117, 1069, 1052, 1019, 980, 948, 922, 912, 895, 835, 798, 751, 733, 692. M.p. ($CHCl_3$) = 52 – 53 ˚C. HRMS calcd for $C_{18}H_{17}DO_4K$ [M + K]$^+$: 338.0899, found 338.0900.

Dimethyl 2-(tetrahydro-4H-pyran-4-ylidene)malonate S24

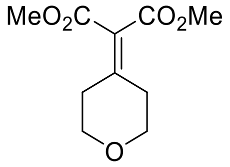

To an oven-dried flask containing THF (28 mL) at 0 °C was slowly added $TiCl_4$ (15.0 mL, 0.015 mmol). To the resulting yellow solution was added dropwise a mixture of tetrahydro-4H-pyran-4-one (500 mg, 5 mmol), dimethyl malonate (1.71 mL, 0.015 mmol) and pyridine (1.21 mL, 0.015 mmol) in THF (9 mL). The reaction was allowed to slowly warm to room temperature over 16 h. The reaction was quenched by slow addition of H_2O while stirring until a homogenous solution was obtained. The resulting aqueous solution was extracted with EtOAc (3 × 30 mL) and the combined organic layers were sequentially washed with 1 M HCl (30 mL) and brine (30 mL), dried over $MgSO_4$, filtered, concentrated *in vacuo* and purified by column chromatography eluting with EtOAc/Hexane (5:98) to give the title compound as a colourless

oil (950 mg, 4.44 mmol, 89%). ^{1}H NMR (400 MHz, $CDCl_3$) δ 2.67 (t, *J* = 5.5 Hz, 4 H, CH_2(C)), 3.76 (d, *J* = 4.2 Hz, 10 H, 4 H from CH_2O, 6 H from 2 × CH_3) ppm. ^{13}C NMR (100 MHz, $CDCl_3$) δ 33.1 (CH_2-C), 52.3 (2 × CH_3), 68.4 (CH_2O), 122.6 ($(CH_2)_2C{=}C$), 156.5 ($C(O)OCH_3$), 165.7 ($(CH_2)_2C{=}C$) ppm. Consistent with the literature.[2]

Dimethyl 2-(tetrahydro-2H-pyran-4-yl)malonate S25

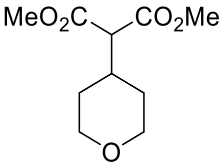

General Procedure **D**. To a solution of dimethyl 2-(tetrahydro-4H-pyran-4-ylidene)malonate (1.65 g, 7.7 mmol) in MeOH (7.7 mL) was added $NaBH_4$ (290 mg, 7.7 mmol) at 0 °C. The reaction was stirred at this temperature for 2 h and at room temperature for 16 h. The reaction mixture was carefully quenched with H_2O (10 mL) and the aqueous layer extracted with EtOAc (3 × 10 mL). The combined organic layers were washed with brine (10 mL), dried over $MgSO_4$, filtered, concentrated *in vacuo* and purified by column chromatography eluting with EtOAc/Hexane (10:90), to give the title compound as a colorless oil (0.97 g, 4.5 mmol, 58%). ^{1}H NMR (400 MHz, $CDCl_3$) δ 1.34 – 1.63 (m, 4 H, CH_2CH), 2.25 – 2.37 (m, 1 H, CH_2CH), 3.21 (d, *J* = 9.2 Hz, 1 H, (CH_2CHCH), 3.40 (td, *J* = 11.8, 2.1 Hz, 2 H, CH_2O), 3.72 (s, 6 H, 2 × CH_3), 3.94 (dtd, *J* = 11.6, 2.5, 1.2 Hz, 2 H, CH_2O) ppm. ^{13}C NMR (100 MHz, $CDCl_3$) δ 30.7 (CH_2CHCH_2), 35.3 (CH_2CHCH_2), 52.6 ($C(O)OCH_3$-CH-$C(O)OCH_3$, 57.6 (CH_2OCH_2), 67.7 (2 x CH_3), 168.7 (2 x $C(O)OCH_3$) ppm. IR ν_{max} (neat/cm^{-1}): 2954, 2844, 1732, 1435, 1258, 1240, 1156, 1133, 1092, 1021, 878. HRMS calcd for $C_{10}H_{16}O_5$ [M^+]: 216.0992, found 216.0992.

[2] Liu W-B., Okamoto., N.; Alexy, E. J.; Hong, A. Y.; Tran, K., Stoltz, B. M. *J. Am. Chem. Soc.*, **2016**, *138* (16), 5234–5237.

Diphenyl 2-(tetrahydro-2H-pyran-4-yl)malonate S26

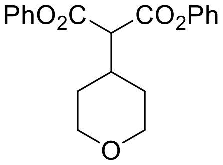

To a mixture of dimethyl 2-(tetrahydro-2H-pyran-4-yl)malonate (990 mg, 5.3 mmol) and phenol (990 mg, 11 mmol) was slowly added $POCl_3$ (0.58 mL, 6.3 mmol) at 0 °C. The reaction mixture was heated at 110 °C for 2 h and, after that time, quenched with H_2O (10 mL). The resulting aqueous solution was extracted with EtOAc (3 × 10 mL) and the combined organic layers were sequentially washed with brine (10 mL), dried over $MgSO_4$, filtered, concentrated *in vacuo* and purified by column chromatography eluting with EtOAc/Hexane (5:95) to give the title compound as a colorless oil (820 mg, 2.42 mmol, 46%). ^{1}H NMR (400 MHz, $CDCl_3$) δ 1.65 – 1.96 (m, 4 H, CH_2CH), 2.62 (tdt, *J* = 12.1, 8.6, 3.7 Hz, 1 H, CH_2C*H*), 3.53 (td, *J* = 11.9, 2.1 Hz, 2 H, CH_2O), 3.74 (d, *J* = 8.6 Hz, 1 H, CH_2CHC*H*), 4.04 – 4.13 (m, 2 H, CH_2O), 7.14 – 7.21 (m, 4 H, ArC*H*), 7.26 – 7.34 (m, 2 H, ArC*H*), 7.40 – 7.48 (m, 4 H, ArC*H*) ppm. ^{13}C NMR (100 MHz, $CDCl_3$) δ 30.7 (CH_2*C*H), 35.3 (*C*H_2CH), 57.5 (CH2CH*C*H), 67.7 (*C*H_2O), 121.3 (Ar*C*H), 126.4 (Ar*C*H), 129.7 (Ar*C*H), 150.4 (Ar*C*), 166.5 (*C*(O)OPh) ppm. IR ν_{max} (neat/cm^{-1}): 2956, 2845, 1750, 1492, 1245, 1186, 1160, 1110, 908, 831, 730, 687. M.p. ($CHCl_3$) = 51 – 53 ˚C. HRMS calcd for $C_{20}H_{20}O_5$ [M - H]$^-$: 339.1238, found 339.1240.

Dimethyl 2-(thiophen-3-ylmethylene)malonate S27

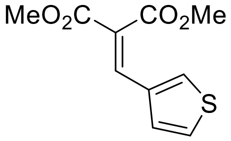

To an oven-dried flask containing thiophene-3-carbaldehyde (2.00 g, 17.9 mmol), dimethyl malonate (2.36 g, 17.9 mmol), piperidine (0.07 mL, 0.71 mmol) and acetic acid (0.2 mL, 3.56 mmol) was added benzene (5 mL) and the mixture was heated at reflux for 4 h. The reaction mixture was quenched with H_2O (5 mL) and the aqueous layer extracted with EtOAc (3 × 10 mL). The combined organic layers were washed with brine (5 mL), dried over $MgSO_4$, filtered, concentrated *in vacuo* and purified by column chromatography eluting with EtOAc/Hexane (10:90), to give the title compound as a yellow oil (3.25 g, 14.4 mmol, 80%). ^{1}H NMR (500 MHz, $CDCl_3$) δ 3.84 (s, 3 H, OCH_3), 3.90 (s, 3 H, OCH_3), 7.15 (d, *J* = 5.1 Hz, 1 H, ArC*H*), 7.34 (dd, *J* = 5.1, 3.0 Hz, 1 H, ArC*H*), 7.61 – 7.64 (m, 1 H, ArC*H*), 7.74 (s, 1 H, C*H*=C)). ^{13}C NMR (126 MHz, $CDCl_3$) δ 52.7 (O*C*H_3), 52.8 (O*C*H_3), 123.5 (Ar*C*), 126.9 (Ar*C*H), 127.1

(Ar*C*H) 131.1 (Ar*C*H), 134.8 (CH=*C*), 136.1 (*C*H=C), 164.8 (*C*(O)O), 167.4 (*C*(O)O). IR ν_{max} (neat/cm^{-1}): 3099, 2952, 1721, 1622, 1435, 1355, 1244, 1160, 1067, 927, 826, 731, 676, 635. HRMS calcd for $C_{10}H_{11}O_4S$ $[M + H]^+$: 227.0373, found 227.0369.

Dimethyl 2-(thiophen-3-ylmethyl)malonate S28

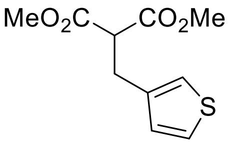

Prepared according to general procedure **D** using dimethyl 2-(thiophen-3-ylmethylene)malonate (1.65 g, 7.7 mmol), $NaBH_4$ (290 mg, 7.7 mmol) and MeOH (7.7 mL) to give the title compound as an oil (1.64 g, 7.18 mmol, 51%). ^{1}H NMR (500 MHz, $CDCl_3$) δ 3.28 (d, *J* = 7.7 Hz, 2 H, CHC*H*2), 3.69 (t, *J* = 7.7 Hz, 1 H, C*H*CH2), 3.74 (s, 6 H, 2 × OC*H*3), 6.95 (d, *J* = 4.9 Hz, 1 H, ArC*H*), 7.04 (s, 1 H, ArC*H*), 7.25 – 7.29 (m, 1 H, ArC*H*). ^{13}C NMR (126 MHz, $CDCl_3$) δ 29.4 (CH*C*H2), 52.7 (O*C*H3), 53.1 (*C*HCH2), 122.1 (Ar*C*H), 126.0 (Ar*C*H), 128.2 (Ar*C*H), 138.0 (Ar*C*), 169.3 (*C*(O)O). IR ν_{max} (neat/cm^{-1}): 2954, 1732, 11435, 1341, 1249, 1221, 1148, 1027, 908, 779, 650, 639. HRMS calcd for $C_{10}H_{13}O_4S$ $[M + H]^+$: 229.0529, found 229.0525.

Diphenyl 2-(thiophen-3-ylmethyl)malonate S29

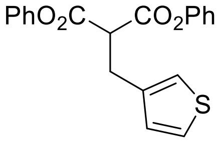

Prepared according to general procedure **C** using dimethyl 2-(thiophen-3-ylmethyl)malonate (1.61 g, 7.07 mmol), 2 M NaOH (9.5 mL, 19.1 mmol), oxalyl chloride (1.8 mL, 21.2 mmol), phenol (1.33 g, 14.0 mmol), DMAP (86 mg, 0. 71 mmol), Et_3N (3.0 mL, 21.2 mmol) and CH_2Cl_2 (25 mL) to give the title compound as a yellow oil (1.33 g, 5.82 mmol, 53% after 3 steps). ^{1}H NMR (400 MHz, $CDCl_3$) δ 3.56 (d, *J* = 7.8 Hz, 2 H, CHC*H*2), 4.22 (t, *J* = 7.8 Hz, 1 H, C*H*CH2), 7.11 (d, *J* = 8.0 Hz, 4 H, ArC*H*), 7.14 (dd, *J* = 5.0, 1.3 Hz, 1 H, ArC*H*), 7.24 (dd, *J* = 3.0, 1.2 Hz, 1 H, ArC*H*), 7.26 – 7.34 (m, 2 H, ArC*H*), 7.38 (dd, *J* = 4.9, 2.9 Hz, 1 H, ArC*H*), 7.43 (dd, *J* = 8.5, 7.3 Hz, 4 H, ArC*H*). ^{13}C NMR (101 MHz, $CDCl_3$) δ 29.3 (CH*C*H2), 53.3 (*C*HCH2), 121.4 (Ar*C*H), 122.8 (Ar*C*H), 126.2 (Ar*C*H), 126.4 (Ar*C*H), 128.4 (Ar*C*H), 129.7 (Ar*C*H), 137.4 (Ar*C*), 150.5 (Ar*C*), 167.2 (*C*(O)O). IR ν_{max} (neat/cm^{-1}): 3098, 1750, 1590,

1491, 1340, 1160, 1123, 913, 832, 744, 687, 639. HRMS calcd for $C_{20}H_{16}O_4SNa$ [M + Na]$^+$: 375.0662, found 375.0655.

4-((*tert*-Butyldimethylsilyl)oxy)butan-1-ol S30

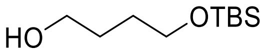

To a cold solution of 1,4-butanediol (0.98 mL, 11 mmol) in CH_2Cl_2 (22 mL) at room temperature, was added imidazole (890 g, 13 mmol) as a solution in CH_2Cl_2 (5 mL). After 30 min, a solution of TBSCl (1. 66 g, 11 mmol) in CH_2Cl_2 (5 mL) was added dropwise and the reaction was stirred at room temperature for 2 h. The reaction was quenched with a H_2O (15 mL) and the aqueous layer extracted with CH_2Cl_2 (3 × 20 mL). The combined organic layers were washed with brine (20 mL), dried over $MgSO_4$, filtered, concentrated *in vacuo* and purified by column chromatography eluting with EtOAc/Hexane (10:90), to give the title compound as a yellow oil (0.90 g, 4.4 mmol, 40%). ^{1}H NMR (500 MHz, $CDCl_3$) δ 0.07 (s, 6 H, Si(C*H*$_3$)$_2$), 0.90 (s, 9 H, Si(C(C*H*$_3$)$_2$)), 1.64 (dq, *J* = 11.3, 6.0 Hz, 4 H, (2 × C*H*$_2$), 2.50 (s, 1 H, O*H*), 3.65 (dt, *J* = 12.7, 5.8 Hz, 4 H, C*H*$_2$OSi, C*H*$_2$OH) ppm. ^{13}C NMR (125 MHz, $CDCl_3$) δ -5.3 (Si(*C*H$_3$)$_2$), 18.4 (Si*C*), 26.0 (SiC(*C*H$_3$)$_3$), 30.0 (*C*H$_2$), 30.4 (*C*H$_2$), 62.9 (*C*H$_2$O), 63.5 (*C*H$_2$O) ppm. Consistent with the literature.[3]

6-((*tert*-Butyldimethylsilyl)oxy)hex-1-en-3-yl 4-(trifluoromethyl)benzoate S31

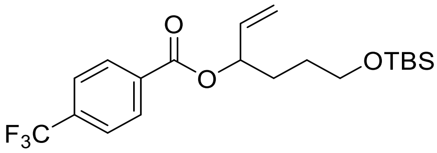

To a solution of oxalyl chloride (0.43 mL, 5.1 mmol) in CH_2Cl_2 (17 mL) was added DMSO (0.56 mL, 7.9 mmol) as a solution in CH_2Cl_2 (14 mL) dropwise, at -78 °C keeping the temperature below -40 °C. After 10 min, a solution of 4-((*tert*-butyldimethylsilyl)oxy)butan-1-ol (700 mg, 3.4 mmol) in CH_2Cl_2 (3 mL) was added dropwise. Aftere 1 h, the solution was treated with Et_3N (1.43 mL, 10 mmol). Upon warming to 0 °C over 1 h and stirring at this temperature for 1 h , the reaction mixture was quenched with H_2O (15 mL). The aqueous phase was extracted with CH_2Cl_2 (3 × 15 mL) and the combined organic layers were washed with brine (15 mL), dried over $MgSO_4$, filtered and concentrated *in vacuo* to give a yellow oil which was used in the next step without further purification. Crude aldehyde (500 mg, 2.5 mmol) was

[3] Peña-López, M.; Martínez, M. M.; Sarandeses, L. A.; Pérez Sestelo, J. *Org. Lett.*, **2010**, *12* (4), 852–854.

dissolved in THF (2.5 mL) and the solution added dropwise to a 1.0 M solution of vinylmagnesium bromide in THF (3.09 mL, 3.1 mmol) at -78 °C. The solution was stirred for 5 min at this temperature, warmed to room temperature and stirred for 30 min. The reaction mixture was quenched with saturated aqueous NH_4Cl (10 mL) and diluted with Et_2O (10) mL. The layers were separated, and the aqueous layer was extracted with Et_2O (3 × 10 mL). The combined organic layers were washed with brine (10 mL), dried over $MgSO_4$, and concentrated *in vacuo* to give the expected alcohol as a yellow oil which was used in the next step without further purification.

General Procedure **E**. To the alcohol (400 mg, 1.74 mmol), DMAP (4.3 mg, 0.02 mmol) and Et_3N (0.48 mL, 3.47 mmol) in CH_2Cl_2 (8.7 mL) at room temperature was added 4-(trifluoromethyl)benzoyl chloride (0.39 mL, 2.61 mmol) dropwise. The solution was stirred at room temperature for 2 h, quenched with H_2O (10 mL) and diluted with CH_2Cl_2 (10 mL). The layers were separated and the aqueous layer was extracted with CH_2Cl_2 (3 × 10 mL). The combined organic layers were washed with brine (10 mL), dried over $MgSO_4$, concentrated *in vacuo* and purified by column chromatography eluting with EtOAc/Hexane (2:98) to give the title compound as a colorless oil (660 mg, 1.64 mmol, 94%). ^{1}H NMR (400 MHz, $CDCl_3$) δ 0.04 (s, 6 H, Si(CH_3)$_2$), 0.89 (s, 9 H, SiC(CH_3)$_3$), 1.54 – 1.70 (m, 2 H, C$H_2$$CH_2$OSi), 1.79 – 1.91 (m, 2 H, C$H_2$$CH_2CH_2$OSi), 3.65 (t, *J* = 6.3 Hz, 2 H, CH_2OSi), 5.24 (dt, *J* = 10.5, 1.2 Hz, 1 H, 1 H from CH_2=CH), 5.34 (dt, *J* = 17.2, 1.2 Hz, 1 H, 1 H from CH_2=CH), 5.54 (q, *J* = 6.5 Hz, 1 H, CH_2=CHC*H*), 5.90 (ddd, *J* = 17.0, 10.5, 6.3 Hz, 1 H, CH_2=C*H*), 7.71 (d, *J* = 8.2 Hz, 2 H, 2 x ArC*H*), 8.17 (d, *J* = 8.1 Hz, 2 H, 2 × ArC*H*) ppm. ^{13}C NMR (100 MHz, $CDCl_3$) δ -5.2 (Si(*C*H_3)$_2$), 18.5 (Si*C*(CH_3)$_3$, 26.1 (SiC(*C*H_3)$_3$), 28.5 (*C*H_2CH_2OSi), 30.8 (*C*$H_2CH_2CH_2$OSi), 62.7 (*C*H_2OSi), 76.0 (CH_2=CH*C*H), 117.4 (*C*H_2=CH), 125.5 (Ar*C*H), 130.1 (Ar*C*H), 133.9 (Ar*C*), 134.4 (Ar*C*), 136.2 (CH_2=*C*HCHOAr), 164.8 (*C*(O)) ppm. *C*F_3 not observed. HRMS calcd for $C_{20}H_{29}F_3O_3SiNa$ $[M + Na]^+$: 425.1730, found 425.1710.

5-Phenylpent-1-en-3-ol S32

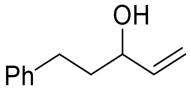

A solution of 3-phenylpropanal (0.66 mL, 5.00 mmol) in THF (15 mL) was added dropwise to a 1 M solution of vinylmagnesium bromide in hexanes (6 mL, 6.00 mmol) at 0 °C. The reaction mixture was allowed to warmed to room temperature, stirred for 45 min, quenched with saturated aqueous NH_4Cl (15 mL) and diluted with Et_2O (10) mL. The layers were separated,

and the aqueous layer was extracted with Et_2O (3 × 15 mL). The combined organic layers were washed with brine (15 mL), dried over $MgSO_4$, concentrated *in vacuo* and purified by column chromatography eluting with EtOAc/Hexane (10:90), to give the title compound as a colourless oil (580 g, 3.6 mmol, 72%). ^{1}H NMR (400 MHz, $CDCl_3$) δ 1.83 – 1.98 (m, 2 H, CHCH_2CH$_2$), 2.68 – 2.86 (m, 2 H, CHCH$_2$CH_2), 4.18 (q, *J* = 6.4 Hz, 1 H, C*H*CH=), 5.19 (dd, *J* = 10.4, 1.4 Hz, 1 H, =CH_aH$_b$), 5.29 (dt, *J* = 17.3, 1.5 Hz, 1 H, =CH$_a$CH_b) 5.95 (ddd, *J* = 16.9, 10.4, 6.2 Hz, 1 H, C*H*=CH$_2$), 7.20 – 7.28 (m, 3 H, ArC*H*), 7.32 (dt, *J* = 7.9, 6.3 Hz, 2 H, ArC*H*) ppm. ^{13}C NMR (100 MHz, $CDCl_3$) δ 31.7 (CH*C*H$_2$CH$_2$), 38.7 (CHCH$_2$*C*H$_2$), 72.5 (*C*HCH$_2$CH$_2$), 115.0 (=*C*H$_2$), 125.8 (Ar*C*H), 128.5 (Ar*C*H), 128.6 (Ar*C*H), 141.1 (*C*H=CH$_2$), 142.2 (Ar*C*) ppm. Consistent with the literature.[4]

5-phenylpent-1-en-3-yl 4-(trifluoromethyl)benzoate S33

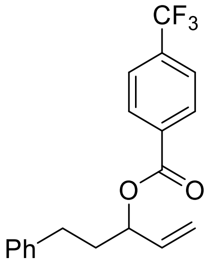

Prepared according to general procedure **E** using 5-phenylpent-1-en-3-ol (41 mg, 2.53 mmol), DMAP (6 mg, 0.05 mmol), 4-(trifluoromethyl) benzoyl chloride (0.56 mL, 3.8 mmol) and Et_3N (0.71 mL, 5.1 mmol) in CH_2Cl_2 (25 mL). The reaction was quenched with H_2O (20 mL) and the aqueous layer extracted with CH_2Cl_2 (3 × 25 mL), combined organic layers were washed with brine (25 mL), dried over $MgSO_4$, filtered, concentrated *in vacuo* and purified by column chromatography eluting with EtOAc/Hexane (10:90), to give the title compound as a colourless oil (0.81 g, 2.4 mmol, 96%). ^{1}H NMR (400 MHz, $CDCl_3$) δ 2.07 – 2.30 (m, 2 H, CHCH_2CH$_2$), 2.81 (t, *J* = 7.9 Hz, 2 H, CHCH$_2$CH_2), 5.30 – 5.47 (m, 2 H, CHCH=CH_2), 5.61 (q, *J* = 6.5 Hz, 1 H, C*H*CH=CH$_2$), 5.99 (ddd, *J* = 17.1, 10.5, 6.3 Hz, 1 H, CHC*H*=CH$_2$), 7.22 – 7.28 (m, 3 H, ArC*H*), 7.30 – 7.39 (m, 2 H, ArC*H*), 7.77 (d, *J* = 8.0 Hz, 2 H, ArC*H*), 8.21 (d, *J* = 8.0 Hz, 2 H, ArC*H*) ppm. ^{13}C NMR (100 MHz, $CDCl_3$) δ 31.6 (CHCH$_2$*C*H$_2$), 35.9 (CH*C*H$_2$CH$_2$), 75.7 (*C*HCH$_2$CH$_2$), 117.7 (CHCH=*C*H$_2$), 125.5 (Ar*C*H), 126.2 (*C*F$_3$), 128.5 (Ar*C*H), 128.6 (Ar*C*H), 130.1 (Ar*C*H), 133.7 (Ar*C*H), 134.4 (Ar*C*), 134.7 (Ar*C*), 135.9 (CH*C*H=CH$_2$), 141.2 (Ar*C*),

[4] Lafrance, M.; Roggen, M.; Carreira, E. M. *Angew. Chem. Int. Ed.* **2012**, *51* (14), 3470 –3473.

164.7 (C(O)O) ppm. IR ν_{max} (neat/cm^{-1}): 2945, 1722, 1411, 1323, 1268, 1167, 1065, 1016, 935, 861, 774, 698. HRMS calcd for $C_{19}H_{17}F_3O_2Na$ [M + Na]$^+$: 357.1073, found 357.1058.

2-(Benzofuran-2-yl) ethan-1-ol S34

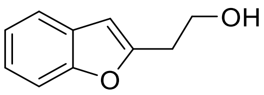

To a stirred mixture of 2-iodophenol (500 mg, 2.27 mmol), palladium acetate (26 mg, 0.11 mmol), CuI (22 mg, 0.11 mmol) and triphenylphosphine (30 mg, 0.11 mmol) in anhydrous Et_3N (5.0 ml) was added 3-butyn-1-ol (0.19 mL, 2.5 mmol). The reaction mixture was stirred for 16 h at room temperature after which it was diluted with EtOAc (20 mL). The EtOAc layer was washed with H_2O (3 × 10 mL) and brine. The combined organic layers were dried over $MgSO_4$, concentrated *in vacuo* and purified by flash column chromatography eluting with EtOAc/Hexane (20:80), to give the title compound as a colorless oil (0.31 g, 1.9 mmol, 83%). ^{1}H NMR (400 MHz, $CDCl_3$) δ 1.72 (bs, 1 H, CH_2H_2OH), 3.09 (t, J = 6.2 Hz, 2 H CH_2CH_2OH), 4.04 (t, J = 6.2 Hz, 2 H, CH_2CH_2OH), 6.56 (s, 1 H, ArCH), 7.20 – 7.31 (m, 2 H, ArCH), 7.45 – 7.50 (m, 1 H, ArCH), 7.52 – 7.57 (m, 1 H, ArCH) ppm. ^{13}C NMR (100 MHz, $CDCl_3$) δ 32.2 (CH_2CH_2OH), 60.9 (CH_2CH_2OH), 103.8 (ArCH), 111.0 (ArCH), 120.6 (ArCH), 122.8 (ArCH), 123.7 (ArCH), 128.8 (ArC), 155.0 (ArC), 156.1 (ArC) ppm. Consistent with the literature.[5]

1-(Benzofuran-2-yl)but-3-en-2-ol S35

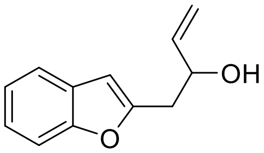

A solution of 1-(benzofuran-2-yl)but-3-en-2-ol (180 mg, 1.1 mmol) in CH_2Cl_2 (2.5 mL) was treated with DMP reagent (494 g, 1.2 mmol) at 0 °C and stirred for 1 h. The reaction mixture was quenched with saturated aqueous $NaHCO_3$ (5 mL) and diluted with CH_2Cl_2 (5 mL). The layers were separated, and the aqueous layer was extracted with CH_2Cl_2 (3 × 5 mL). The combined organic layers were washed with brine (5 mL), dried over $MgSO_4$ and concentrated

[5] Sibasish, P.; Sankha, P.; Surajit, S. *Tetrahedron Lett.* **2011**, *52* (46), 6166-6169.

in vacuo to give the crude aldehyde as a yellow oil which was used in the next step without further purification. Crude aldehyde (200 mg, 1.25 mmol) was dissolved in THF (1.3 mL) and was added dropwise to a 1.0 M solution of vinylmagnesium bromide (1.58 mL, 1.56 mmol) in THF (1 mL) at -78 °C. The solution was stirred for 5 min at this temperature, warmed to room temperature and stirred at this temperature for 30 min. The reaction mixture was quenched with saturated aqueous NH_4Cl (5 mL) and diluted with Et_2O (5 mL). The layers were separated, and the aqueous layer was extracted with Et_2O (3 × 5 mL). The combined organic layers were washed with brine (5 mL), dried over $MgSO_4$, concentrated *in vacuo* and purified by column chromatography eluting with EtOAc/Hexane (10:90), to give the title compound as a colourless oil (0.051 g, 0.27 mmol, 22%). ^{1}H NMR (400 MHz, $CDCl_3$) δ 2.20 (s, 1 H, O*H*), 2.98 – 3.12 (m, 2 H, OHCHC*H*$_2$), 4.60 (q, *J* = 6.2 Hz, 1 H, OHC*H*CH$_2$), 5.21 (d, *J* = 10.5 Hz, 1 H, =C*H*$_2$), 5.36 (d, *J* = 17.2 Hz, 1 H, =C*H*$_2$), 6.00 (ddd, *J* = 16.7, 10.4, 5.8 Hz, 1 H, C*H*=CH$_2$), 6.56 (s, 1 H, ArC*H*), 7.20 – 7.33 (m, 2 H, ArC*H*), 7.44 – 7.60 (m, 2 H, ArC*H*) ppm. ^{13}C NMR (100 MHz, $CDCl_3$) δ 36.8 (*C*H$_2$), 71.3 (OH*C*H), 104.5 (Ar*C*H), 111.0 (Ar*C*H), 115.7 (CH=*C*H$_2$), 120.6 (Ar*C*H), 122.8 (Ar*C*H), 123.7 (Ar*C*), 128.8 (Ar*C*H), 139.6 (*C*H=CH$_2$), 155.00 (Ar*C*), 155.3 (Ar*C*) ppm. IR ν_{max} (neat/cm^{-1}): 3395, 2921, 1603, 1454, 1423, 1253, 1029, 928, 750. HRMS calcd for $C_{12}H_{12}O_2$ $[M]^+$: 188.0832, found 188.0823.

1-(Benzofuran-2-yl)but-3-en-2-yl 4-(trifluoromethyl)benzoate S36

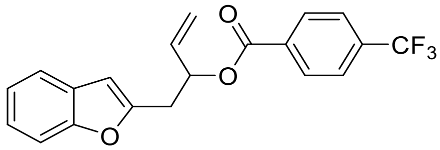

Prepared according to general procedure **E** using 2-(benzofuran-2-yl)ethan-1-ol (50 mg, 2.7 mmol), DMAP (7 mg, 0.006 mmol), 4-(trifluoromethyl)benzoyl chloride (0.06 mL, 0.4 mmol) and Et_3N (0.074 mL, 0.53 mmol) in CH_2Cl_2 (7 mL) to give the title compound as a yellow oil (0.07 g, 0.19 mmol, 72%). ^{1}H NMR (400 MHz, $CDCl_3$) δ 3.22 – 3.34 (m, 2 H, CHC*H*$_2$), 5.29 (dt, *J* = 10.4, 1.1 Hz, 1 H, =C*H*$_2$), 5.41 (dt, *J* = 17.1, 1.1 Hz, 1 H, =C*H*$_2$), 5.87 – 5.94 (m, 1 H, C*H*CH$_2$), 6.00 (ddd, *J* = 16.9, 10.4, 6.3 Hz, 1 H, C*H*=CH$_2$), 6.51 (d, *J* = 1.0 Hz, 1 H, ArC*H*), 7.15 – 7.25 (m, 2 H, ArC*H*), 7.37 – 7.42 (m, 1 H, ArC*H*), 7.45 – 7.51 (m, 1 H, ArC*H*), 7.70 (d, *J* = 8.2 Hz, 2 H, ArC*H*), 8.11 – 8.19 (m, 2 H, ArC*H*) ppm. ^{13}C NMR (100 MHz, $CDCl_3$) δ 33.9 (*C*H$_2$), 73.9 (*C*HCH$_2$), 104.6 (Ar*C*H), 111.0 (Ar*C*H), 118.3 (=*C*H$_2$), 120.7 (Ar*C*H), 122.4 (Ar*C*H), 124.1 (Ar*C*H), 125.3 (Ar*C*H), 128.7 (Ar*C*), 130.2 (Ar*C*H), 133.6 (Ar*C*), 134.5 (Ar*C*), 135.0 (*C*H=CH$_2$), 153.9 (Ar*C*), 155.0 (Ar*C*), 164.5 (*C*(O)O) ppm. IR ν_{max} (neat/cm^{-1}): 2970,

1727, 1455, 1412, 1325, 1272, 1170, 1133, 1101, 1066, 1018, 775, 733. HRMS calcd for $C_{20}H_{15}F_3O_3$ $[M]^+$: 360.0968, found 360.0969.

Diethyl 2-isopropyl-2-(3-oxooct-7-en-1-yl)malonate S37

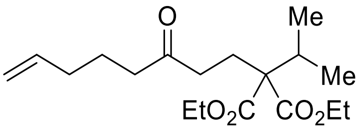

General procedure **F**. In an oven dried flask diethyl 2-isopropylmalonate (404 mg, 2.00 mmol) and *t*-BuOK (22 mg, 0.20 mmol) were dissolved in Et_2O (5 mL)/*t*-BuOH (15 mL) and the solution stirred for 15 min under nitrogen atmosphere at room temperature. Octa-1,7-dien-3-one (273 mg, 2.2 mmol) was added dropwise in Et_2O (1 mL)/t-BuOH (1 mL) over 1 h and the reaction was continued for another 1 h before being quenched with saturated NH_4Cl (20 mL). The aqueous layer was then extracted with Et_2O (3 × 30 mL), the combined organic layers were washed with brine (20 mL), dried over $MgSO_4$, concentrated *in vacuo.* Purification by column chromatography, first column eluting with EtOAc/Toluene (2:98), second column eluting with CH_2Cl_2/Hex (2:1) to (3:1) gave the title compound as a colourless oil (405 mg, 1.24 mmol, 62%). ^{1}H NMR (400 MHz, $CDCl_3$) δ 0.99 (d, *J* = 6.8 Hz, 6 H, $CH(CH_3)_2$), 1.27 (t, *J* = 7.1 Hz, 6 H, 2 × OCH_2CH_3), 1.67 (p, *J* = 7.5 Hz, 2 H, $CH_2CH_2CH_2C(O)$), 2.01 – 2.09 (m, 2 H, $CH_2CH_2CH_2C(O)$), 2.10 – 2.17 (m, 2 H, $C(O)CH_2CH_2C$), 2.30 (hept, *J* = 6.8 Hz, 1 H, $CH(CH_3)_2$), 2.40 (t, *J* = 7.4 Hz, 2 H, $CH_2CH_2CH_2C(O)$), 2.43 – 2.50 (m, 2 H, $C(O)CH_2CH_2C$), 4.14 – 4.25 (m, 4 H, 2 × OCH_2CH_3), 4.94 – 5.06 (m, 2 H, CH_2=CH), 5.70 – 5.82 (m, 1 H, CH_2=C*H*) ppm. ^{13}C NMR (100 MHz, $CDCl_3$) δ 14.1 (OCH_2CH_3), 18.5 ($CH(CH_3)_2$), 22.8 ($CH_2CH_2CH_2C(O)$), 27.0 ($C(O)CH_2CH_2C$), 33.0 ($CH(CH_3)_2$), 33.1 ($CH_2CH_2CH_2C(O)$), 38.5 ($C(O)CH_2CH_2C$), 41.9 ($CH_2CH_2CH_2C(O)$), 60.9 (OCH_2CH_3), 61.0 ($C(O)CH_2CH_2C$), 115.2 (CH_2=$CHCH_2$), 138.0 (CH_2=$CHCH_2$), 170.9 (*C*(O)O), 209.8 (*C*(O)) ppm. IR ν_{max} (neat/cm^{-1}): 2976, 2359, 1720, 1446, 1416, 1391, 1368, 1248, 1194, 1126, 1094, 1026, 1094, 1026, 912, 859. HRMS calcd for $C_{18}H_{31}O_5$ $[M + H]^+$: 327.2166, found 327.2151.

Diphenyl 2-cyclopentyl-2-(3-oxooct-7-en-1-yl)malonate S38

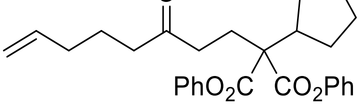

Prepared according to general procedure **F** using diphenyl 2-cyclopentylmalonate (1.30 g, 4.00 mmol), *t*-BuOK (44 mg, 0.40 mmol), octa-1,7-dien-3-one (546 mg, 4.40 mmol) and Et_2O (10

mL)/*t*-BuOH (30 mL) to give the title compound as a colourless oil (458 mg, 1.02 mmol, 25%). ^{1}H NMR (400 MHz, $CDCl_3$) δ 1.57 – 1.81 (m, 8 H, 6 H from 3 × CH_2, 2 H from $CH_2CH_2CH_2C(O)$), 1.94 – 2.11 (m, 4 H, 2 H from CH_2, 2 H from $CH_2CH_2CH_2C(O)$), 2.39 – 2.50 (m, 4 H, 2 H from $CH_2CH_2CH_2C(O)$, 2 H from $C(O)CH_2CH_2C$), 2.65 – 2.80 (m, 3 H, 1 H from $CH(CH_2)_2$, 2 H from $C(O)CH_2CH_2C$), 4.94 – 5.06 (m, 2 H, CH_2=$CHCH_2$), 5.76 (ddt, *J* = 17.0, 10.2, 6.7 Hz, 1 H, CH_2=$CHCH_2$), 7.07 – 7.17 (m, 4 H, Ar*CH*), 7.23 – 7.32 (m, 2 H, Ar*CH*), 7.36 – 7.46 (m, 4 H, Ar*CH*) ppm. ^{13}C NMR (100 MHz, $CDCl_3$) δ 22.8 ($CH_2CH_2CH_2C(O)$), 25.7 (CH_2), 28.0 ($C(O)CH_2CH_2C$), 28.3 (CH_2), 33.1 ($CH_2CH_2CH_2C(O)$), 38.4 ($C(O)CH_2CH_2C$), 42.1 ($CH_2CH_2CH_2C(O)$), 44.5 ($CH(CH_2)_2$), 60.3 ($C(O)CH_2CH_2C$), 115.3 (CH_2=$CHCH_2$), 121.4 (Ar*C*H), 126.3 (Ar*C*H), 129.6 (Ar*C*H), 137.9 (CH_2=$CHCH_2$), 150.5 (Ar*C*), 169.7 (*C*(O)O), 209.4 (*C*(O)) ppm. IR ν_{max} (neat/cm^{-1}): 2950, 2870, 2360, 2342, 1744, 1713, 1591, 1492, 1456, 1369, 1291, 1186, 1161, 1069, 1024, 999, 913, 833, 742, 687. HRMS calcd for $C_{28}H_{32}O_5K$ [M + K]$^+$: 487.1881, found 487.1885.

Diphenyl 2-cyclohexyl-2-(3-oxooct-7-en-1-yl)malonate S39

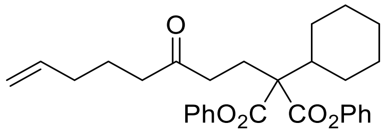

Prepared according to general procedure **F** using diphenyl 2-cyclohexylmalonate (1.35 g, 4.00 mmol), *t*-BuOK (44 mg, 0.40 mmol), octa-1,7-dien-3-one (546 mg, 4.40 mmol) and Et_2O (10 mL)/*t*-BuOH (30 mL) to give the title compound as a colourless oil (408 mg, 0.88 mmol, 22%). ^{1}H NMR (400 MHz, $CDCl_3$) δ 1.16 – 1.31 (m, 1 H, CH_2), 1.32 – 1.45 (m, 4 H, 2 × CH_2), 1.65 – 1.80 (m, 3 H, 1 H from CH_2, 2 H from $CH_2CH_2CH_2C(O)$), 1.83 – 1.93 (m, 2 H, CH_2), 1.99 – 2.10 (m, 4 H, 2 H from CH_2, 2 H from $CH_2CH_2CH_2C(O)$), 2.16 – 2.29 (m, 1 H, $CH(CH_2)_2$), 2.41 – 2.49 (m, 4 H, 2 H from $CH_2CH_2CH_2C(O)$, 2 H from $C(O)CH_2CH_2C$), 2.63 – 2.70 (m, 2 H, $C(O)CH_2CH_2C$), 4.94 – 5.05 (m, 2 H, CH_2=$CHCH_2$), 5.75 (ddt, *J* = 16.9, 10.2, 6.7 Hz, 1 H, CH_2=$CHCH_2$), 7.09 – 7.17 (m, 4 H, Ar*CH*), 7.23 – 7.32 (m, 2 H, Ar*CH*), 7.37 – 7.46 (m, 4 H, Ar*CH*) ppm. ^{13}C NMR (100 MHz, $CDCl_3$) δ 22.8 ($CH_2CH_2CH_2C(O)$), 26.4 (CH_2), 26.9 ($C(O)CH_2CH_2C$), 27.0 (CH_2), 28.8 (CH_2), 33.1 ($CH_2CH_2CH_2C(O)$), 38.4 ($C(O)CH_2CH_2C$), 42.1 ($CH_2CH_2CH_2C(O)$), 43.7 ($CH(CH_2)_2$), 61.3 ($C(O)CH_2CH_2C$), 115.3 (CH_2=$CHCH_2$), 121.4 (Ar*C*H), 126.2 (Ar*C*H), 129.6 (Ar*C*H), 137.9 (CH_2=$CHCH_2$), 150.4 (Ar*C*), 169.3 (*C*(O)O), 209.4 (*C*(O)) ppm. IR ν_{max} (neat/cm^{-1}): 2930, 2853, 1744, 1714, 1591, 1492, 1451, 1185, 1161, 1136, 1069, 1024, 999, 912, 744, 687. HRMS calcd for $C_{29}H_{34}O_5K$ [M + K]$^+$: 501.2038, found 501.2041.

Diphenyl 2-cycloheptyl-2-(3-oxooct-7-en-1-yl)malonate S40

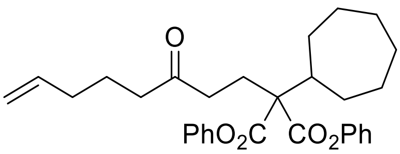

Prepared according to general procedure **F** using 2-cycloheptylmalonate (704 mg, 2.00 mmol), *t*-BuOK (22 mg, 0.20 mmol), octa-1,7-dien-3-one (273 mg, 2.20 mmol) and Et_2O (5 mL)/*t*-BuOH (15 mL) to give the title compound as a colourless oil (408 mg, 0.86 mmol, 43%). ^{1}H NMR (400 MHz, $CDCl_3$) δ 1.51 – 1.73 (m, 10 H, 8 H from 4 × CH_2, 2 H from $CH_2CH_2CH_2$C(O)), 1.75 – 1.91 (m, 2 H, CH_2), 1.95 – 2.10 (m, 4 H, 2 H from CH_2, 2 H from $CH_2CH_2CH_2$C(O)), 2.34 – 2.48 (m, 5 H, 1 H from C*H*$(CH_2)_2$, 2 H from $CH_2CH_2CH_2$C(O), 2 H from C(O)CH_2CH_2C), 2.68 – 2.76 (m, 2 H, C(O)CH_2CH_2C), 4.92 – 5.05 (m, 2 H, CH_2=CHCH_2), 5.75 (ddt, *J* = 17.0, 10.2, 6.7 Hz, 1 H, CH_2=C*H*CH_2), 7.08 – 7.17 (m, 4 H, ArC*H*), 7.24 – 7.31 (m, 2 H, ArC*H*), 7.37 – 7.47 (m, 4 H, ArC*H*) ppm. ^{13}C NMR (100 MHz, $CDCl_3$) δ 22.8 ($CH_2CH_2CH_2$C(O)), 27.5 (C(O)CH_2CH_2C), 27.7 (*C*H_2), 27.9 (*C*H_2), 30.5 (*C*H_2), 33.1 (*C*$H_2CH_2CH_2$C(O)), 38.6 (C(O)*C*H_2CH_2C), 42.1 (CH_2CH_2*C*H_2C(O)), 44.7 (*C*H$(CH_2)_2$), 62.7 (C(O)CH_2CH_2*C*), 115.3 (*C*H_2=CHCH_2), 121.4 (Ar*C*H), 126.2 (Ar*C*H), 129.6 (Ar*C*H), 137.9 (CH_2=*C*HCH_2), 150.4 (Ar*C*), 169.7 (*C*(O)O), 209.6 (*C*(O)) ppm. IR ν_{max} (neat/cm^{-1}): 2922, 2856, 1744, 1713, 1591, 1491, 1456, 1292, 1186, 1160, 1069, 1023, 1000, 912, 831, 743, 687. HRMS calcd for $C_{30}H_{36}O_5K$ [M + K]$^+$: 515.2194, found 515.2201.

Diphenyl 2-(3-oxooct-7-en-1-yl)-2-(tetrahydro-2H-pyran-4-yl)malonate S41

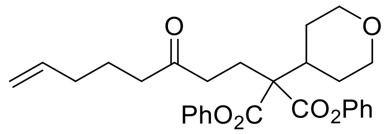

Prepared according to general procedure **F** using diphenyl 2-(tetrahydro-2H-pyran-4-yl)malonate (68 mg, 2.00 mmol), *t*-BuOK (22 mg, 20 mmol), octa-1,7-dien-3-one (27 mg, 2.20 mmol) and Et_2O (5 mL)/*t*-BuOH (15 mL) to give the title compound as a colourless oil (0.16 g, 3.3 mmol, 15%). ^{1}H NMR (400 MHz, $CDCl_3$) δ 1.67 (pd, *J* = 7.6, 1.3 Hz, 2 H, $CH_2CH_2CH_2$C(O)), 1.72 – 1.91 (m, 4 H, CH_2CHCH_2), 1.98 – 2.09 (m, 2 H, $CH_2CH_2CH_2$C(O)), 2.43 (m, 5 H, $CH_2CH_2CH_2$C(O), C(O)$CH_2CH_2C_{quat}$, CH_2C*H*CH_2), 2.65 (dd, *J* = 9.1, 6.3 Hz, 2 H, C(O)$CH_2CH_2C_{quat}$), 3.40 – 4.14 (m, 4 H, CH_2OCH_2), 4.91 – 5.04 (m, 2 H, CH_2=CH), 5.73 (ddtd, *J* = 17.0, 10.3, 6.7, 1.3 Hz, 1 H, CH_2=C*H*CH_2), 7.08 – 7.14 (m, 4 H, ArC*H*), 7.22 – 7.30 (m, 2 H, ArC*H*), 7.40 (td, *J* = 7.9, 1.5 Hz, 4 H, ArC*H*) ppm. ^{13}C NMR (100 MHz, $CDCl_3$) δ

22.9 ($CH_2\mathit{C}H_2CH_2C(O)$), 26.6 ($C(O)CH_2\mathit{C}H_2C_{quat}$), 28.9 ($C\mathit{H_2}CHC\mathit{H_2}$), 33.2 ($C\mathit{H_2}CH_2CH_2C(O)$), 38.2 ($C(O)\mathit{C}H_2CH_2C_{quat}$), 40.8 ($CH_2CH_2\mathit{C}H_2C(O)$), 42.2 ($\mathit{C}H$), 60.6 ($C(O)CH_2CH_2\mathit{C}_{quat}$), 68.4 ($\mathit{C}H_2O\mathit{C}H_2$), 115.5 ($\mathit{C}H_2{=}CH$), 121.4 (Ar*C*H), 126.5 (Ar*C*H), 129.8 (Ar*C*H), 138.0 ($CH_2{=}\mathit{C}H$), 150.4 (Ar*C*), 169.0 (*C*(O)O), 209.2 (*C*(O)) ppm. IR ν_{max} (neat/cm^{-1}): 29523, 2845, 1744, 1715, 1592, 1492, 1371, 1187, 1162, 1122, 1091, 1024, 913, 744, 688. HRMS calcd for $C_{28}H_{33}O_6$ [M + H]$^+$: 465.2272, found 465.2265.

Diphenyl 2-methyl-2-(3-oxooct-7-en-1-yl)malonate S42

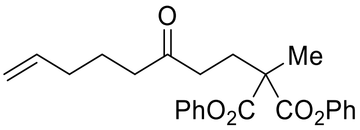

Prepared according to general procedure **F** using diphenyl 2-methylmalonate (540 mg, 2.00 mmol), *t*-BuOK (22 mg, 0.20 mmol), octa-1,7-dien-3-one (273 mg, 2.20 mmol) and Et_2O (5 mL)/*t*-BuOH (15 mL) to give the title compound as a colourless oil (270 mg, 0.68 mmol, 34%). ^{1}H NMR (400 MHz, $CDCl_3$) δ 1.67 – 1.73 (m, 2 H, $CH_2C\mathit{H_2}CH_2C(O)$), 1.72 (s, 3 H, $C\mathit{H_3}$), 2.03 – 2.10 (m, 2 H, $C\mathit{H_2}CH_2CH_2C(O)$), 2.40 – 2.50 (m, 4 H, 2 H from $CH_2CH_2C\mathit{H_2}C(O)$, 2 H from $C(O)CH_2C\mathit{H_2}C$), 2.64 – 2.72 (m, 2 H, $C(O)C\mathit{H_2}CH_2C$), 4.95 – 5.06 (m, 2 H, $C\mathit{H_2}{=}CHCH_2$), 5.76 (ddt, *J* = 17.0, 10.2, 6.7 Hz, 1 H, $CH_2{=}C\mathit{H}CH_2$), 7.09 – 7.17 (m, 4 H, ArC*H*), 7.23 – 7.31 (m, 2 H, ArC*H*), 7.36 – 7.46 (m, 4 H, ArC*H*) ppm. ^{13}C NMR (100 MHz, $CDCl_3$) δ 20.6 ($\mathit{C}H_3$), 22.8 ($CH_2\mathit{C}H_2CH_2C(O)$), 29.4 ($C(O)CH_2\mathit{C}H_2C$), 33.1 ($\mathit{C}H_2CH_2CH_2C(O)$), 37.8 ($C(O)\mathit{C}H_2CH_2C$), 42.1 ($CH_2CH_2\mathit{C}H_2C(O)$), 53.4 ($C(O)CH_2CH_2\mathit{C}$), 115.3 ($\mathit{C}H_2{=}CHCH_2$), 121.2 (Ar*C*H), 126.3 (Ar*C*H), 129.6 (Ar*C*H), 137.9 ($CH_2{=}\mathit{C}HCH_2$), 150.5 (Ar*C*), 170.3 (*C*(O)O), 209.1 (*C*(O)) ppm. IR ν_{max} (neat/cm^{-1}): 2941, 1749, 1713, 1640, 1591, 1492, 1456, 1414, 1380, 1292, 1187, 1161, 1086, 1023, 1004, 917, 830, 744. HRMS calcd for $C_{24}H_{26}O_5Na$ [M + Na]$^+$: 417.1672, found 417.1678.

Diphenyl 2-ethyl-2-(3-oxooct-7-en-1-yl)malonate S43

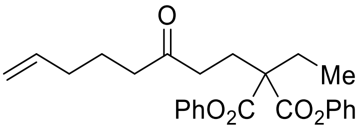

Prepared according to general procedure **F** using diphenyl 2-ethylmalonate (568 mg, 2.00 mmol), *t*-BuOK (22 mg, 0.20 mmol), octa-1,7-dien-3-one (273 mg, 2.20 mmol) and Et_2O (5 mL)/*t*-BuOH (15 mL) to give the title compound as a colourless oil (203 mg, 0.50 mmol, 25%). ^{1}H NMR (400 MHz, $CDCl_3$) δ 1.09 (t, *J* = 7.5 Hz, 3 H, $CH_2C\mathit{H_3}$), 1.70 (p, *J* = 7.4 Hz, 2 H,

CH$_2$C*H*$_2$CH$_2$C(O)), 2.01 – 2.11 (m, 2 H, C*H*$_2$CH$_2$CH$_2$C(O)), 2.22 (q, *J* = 7.5 Hz, 2 H, C*H*$_2$CH$_3$), 2.40 – 2.50 (m, 4 H, 2 H from CH$_2$CH$_2$C*H*$_2$C(O), 2 H from C(O)CH$_2$C*H*$_2$C), 2.57 – 2.66 (m, 2 H, C(O)C*H*$_2$CH$_2$C), 4.94 – 5.06 (m, 2 H, C*H*$_2$=CHCH$_2$), 5.76 (ddt, *J* = 16.9, 10.2, 6.7 Hz, 1 H, CH$_2$=C*H*CH$_2$), 7.09 – 7.16 (m, 4 H, ArC*H*), 7.24 – 7.31 (m, 2 H, ArC*H*), 7.37 – 7.46 (m, 4 H, ArC*H*). ^{13}C NMR (100 MHz, CDCl$_3$) δ 8.7 (CH$_2$*C*H$_3$), 22.8 (CH$_2$*C*H$_2$CH$_2$C(O)), 26.0 (*C*H$_2$CH$_3$), 26.7 (C(O)CH$_2$*C*H$_2$C), 33.1 (*C*H$_2$CH$_2$CH$_2$C(O)), 37.6 (C(O)*C*H$_2$CH$_2$C), 42.1 (CH$_2$CH$_2$*C*H$_2$C(O)), 57.7 (C(O)CH$_2$CH$_2$*C*), 115.3 (*C*H$_2$=CHCH$_2$), 121.3 (Ar*C*H), 126.3 (Ar*C*H), 129.6 (Ar*C*H), 137.9 (CH$_2$=*C*HCH$_2$), 150.5 (Ar*C*), 169.8 (*C*(O)O), 209.1 (*C*(O)) ppm. IR ν_{max} (neat/cm^{-1}): 2942, 2360, 1747, 1714, 1640, 1591, 1492, 1457, 1371, 1293, 1184, 1161, 1104, 1023, 998, 910, 833, 743, 687. HRMS calcd for $C_{25}H_{28}O_5K$ [M + K]$^+$: 447.1568, found 447.1567.

Diphenyl 2-(3-oxooct-7-en-1-yl)-2-phenylmalonate S44

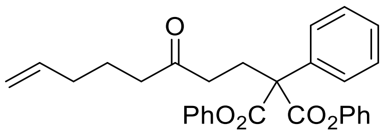

Prepared according to general procedure **F** using diphenyl 2-phenylmalonate (644 mg, 2.00 mmol), *t*-BuOK (22 mg, 0.20 mmol), octa-1,7-dien-3-one (273 mg, 2.20 mmol) and Et$_2$O (5 mL)/*t*-BuOH (15 mL) to give the title compound as a colourless oil (575 mg, 1.26 mmol, 63%). ^{1}H NMR (400 MHz, CDCl$_3$) δ 1.66 (p, *J* = 7.4 Hz, 2 H, CH$_2$C*H*$_2$CH$_2$C(O)), 1.99 – 2.07 (m, 2 H, C*H*$_2$CH$_2$CH$_2$C(O)), 2.34 – 2.41 (m, 2 H, CH$_2$CH$_2$C*H*$_2$C(O)), 2.57 – 2.64 (m, 2 H, C(O)CH$_2$C*H*$_2$C), 2.88 – 2.95 (m, 2 H, C(O)C*H*$_2$CH$_2$C), 4.93 – 5.02 (m, 2 H, C*H*$_2$=CHCH$_2$), 5.74 (ddt, *J* = 17.0, 10.2, 6.7 Hz, 1 H, CH$_2$=C*H*CH$_2$), 7.07 – 7.16 (m, 4 H, ArC*H*), 7.24 – 7.30 (m, 2 H, ArC*H*), 7.37 – 7.43 (m, 5 H, ArC*H*), 7.44 – 7.49 (m, 2 H, ArC*H*), 7.60 – 7.65 (m, 2 H, ArC*H*) ppm. ^{13}C NMR (100 MHz, CDCl$_3$) δ 22.8 (CH$_2$*C*H$_2$CH$_2$C(O)), 29.4 (C(O)CH$_2$*C*H$_2$C), 33.0 (*C*H$_2$CH$_2$CH$_2$C(O)), 38.1 (C(O)*C*H$_2$CH$_2$C), 42.0 (CH$_2$CH$_2$*C*H$_2$C(O)), 62.1 (C(O)CH$_2$CH$_2$*C*), 115.3 (*C*H$_2$=CHCH$_2$), 121.2 (Ar*C*H), 126.4 (Ar*C*H), 128.0 (Ar*C*H), 128.3 (Ar*C*H), 128.7 (Ar*C*H), 129.6 (Ar*C*H), 135.6 (Ar*C*), 137.9 (CH$_2$=*C*HCH$_2$), 150.5 (Ar*C*-O), 168.9 (*C*(O)O), 209.1 (*C*(O)) ppm. IR ν_{max} (neat/cm^{-1}): 2932, 1747, 1713, 1639, 1591, 1491, 1456, 1447, 1414, 1372, 1292, 1183, 1160, 1069, 1023, 1002, 912, 832, 793, 744, 687. HRMS calcd for $C_{29}H_{28}O_5Na$ [M + Na]$^+$: 479.1829, found 479.1825.

Diphenyl 2-benzyl-2-(3-oxooct-7-en-1-yl)malonate S45

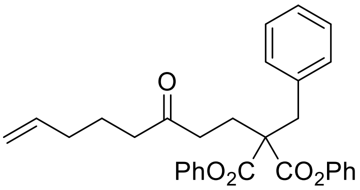

Prepared according to general procedure **F** using diphenyl 2-benzylmalonate (692 mg, 2.00 mmol), *t*-BuOK (22 mg, 0.20 mmol), octa-1,7-dien-3-one (273 mg, 2.20 mmol) and Et_2O (5 mL)/*t*-BuOH (15 mL) to give the title compound as a colourless oil (461 mg, 0.98 mmol, 49%). ^{1}H NMR (400 MHz, $CDCl_3$) δ 1.69 (p, *J* = 7.3 Hz, 2 H, $CH_2CH_2CH_2C(O)$), 2.00 – 2.10 (m, 2 H, $CH_2CH_2CH_2C(O)$), 2.37 – 2.49 (m, 4 H, 2 H from $CH_2CH_2CH_2C(O)$, 2 H from $C(O)CH_2CH_2C$), 2.65 – 2.73 (m, 2 H, $C(O)CH_2CH_2C$), 3.51 (s, 2 H, CH_2Ph), 4.94 – 5.04 (m, 2 H, $CH_2{=}CHCH_2$), 5.75 (ddt, *J* = 17.0, 10.2, 6.7 Hz, 1 H, $CH_2{=}CHCH_2$), 7.06 – 7.11 (m, 4 H, Ar*CH*), 7.25 – 7.38 (m, 7 H, ArC*H*), 7.38 – 7.44 (m, 4 H, ArC*H*) ppm. ^{13}C NMR (100 MHz, $CDCl_3$) δ 22.7 ($CH_2CH_2CH_2C(O)$), 26.9 ($C(O)CH_2CH_2C$), 33.0 ($CH_2CH_2CH_2C(O)$), 37.8 ($C(O)CH_2CH_2C$), 39.6 (CH_2Ph), 42.0 ($CH_2CH_2CH_2C(O)$), 58.6 ($C(O)CH_2CH_2C$), 115.3 ($CH_2{=}CHCH_2$), 121.2 (ArCH), 126.3 (Ar*C*H), 127.5 (Ar*C*H), 128.6 (Ar*C*H), 129.6 (Ar*C*H), 130.3 (Ar*C*H), 135.1 (Ar*C*), 137.9 ($CH_2{=}CHCH_2$), 150.4 (Ar*C*-O), 169.3 (*C*(O)O), 208.9 (*C*(O)) ppm. IR ν_{max} (neat/cm^{-1}): 3062, 2941, 1747, 1714, 1640, 1590, 1492, 1455, 1413, 1371, 1292, 1185, 1161, 1080, 1024, 1002, 914, 834, 736, 701, 688. HRMS calcd for $C_{30}H_{30}O_5Na$ $[M + Na]^+$: 493.1985, found 493.1990.

Diphenyl 2-(4-chlorobenzyl)-2-(3-oxooct-7-en-1-yl)malonate S46

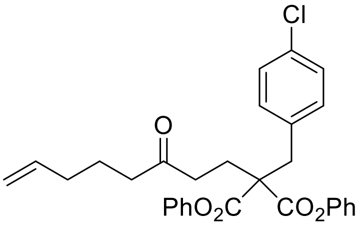

Prepared according to general procedure **F** using diphenyl 2-(4-chlorobenzyl)malonate (762 mg, 2.00 mmol), *t*-BuOK (22 mg, 0.20 mmol), octa-1,7-dien-3-one (273 mg, 2.20 mmol) and Et_2O (5 mL)/*t*-BuOH (15 mL) to give the title compound as a colourless oil (349 mg, 0.69 mmol, 35%). ^{1}H NMR (400 MHz, $CDCl_3$) δ 1.68 (p, *J* = 7.3 Hz, 2 H, $CH_2CH_2CH_2C(O)$), 2.00 – 2.07 (m, 2 H, $CH_2CH_2CH_2C(O)$), 2.35 – 2.47 (m, 4 H, 2 H from $CH_2CH_2CH_2C(O)$, 2 H from $C(O)CH_2CH_2C_{quat}$), 2.64 – 2.71 (m, 2 H, $C(O)CH_2CH_2C_{quat}$), 3.46 (s, 2 H, CH_2Ph), 4.94 – 5.03 (m, 2 H, $CH_2{=}CHCH_2$), 5.68 – 5.80 (m, 1 H, $CH_2{=}CHCH_2$), 7.04 – 7.09 (m, 4 H, ArC*H*), 7.21

– 7.33 (m, 6 H, ArC*H*), 7.37 – 7.44 (m, 4 H, ArC*H*) ppm. ^{13}C NMR (100 MHz, $CDCl_3$) δ 22.8 (CH_2*C*H_2CH_2C(O)), 27.1 (C(O)CH_2*C*H_2C_{quat}), 33.2 (*C*$H_2CH_2CH_2$C(O)), 37.9 (C(O)*C*$H_2CH_2C_{quat}$), 39.3 (*C*H_2Ph), 42.2 (CH_2CH_2*C*H_2C(O)), 58.6 (C(O)CH_2CH_2*C*$_{quat}$), 115.5 (*C*H_2=$CHCH_2$), 121.3 (Ar*C*H), 126.5 (Ar*C*H), 128.9 (Ar*C*H), 129.8 (Ar*C*H), 131.8 (Ar*C*H), 133.7 (Ar*C*), 133.8 (Ar*C*), 138.0 (CH_2=*C*HCH_2), 150.5 (Ar*C*-O), 169.3 (*C*(O)O), 209.0 (*C*(O)) ppm. IR ν_{max} (neat/cm^{-1}): 2924, 2363, 1749, 1492, 1196, 720, 684, 668, 649. HRMS calcd for $C_{30}H_{29}ClO_5K$ [M+K]$^+$: 543.1340, found 543.1340.

Diphenyl 2-(4-fluorobenzyl)-2-(3-oxooct-7-en-1-yl)malonate S47

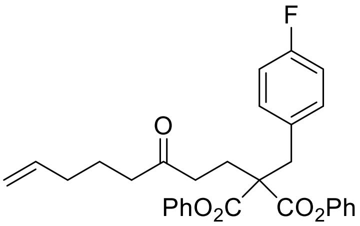

Prepared according to general procedure **F** using diphenyl 2-(4-fluorobenzyl)malonate (729 mg, 2.00 mmol), *t*-BuOK (22 mg, 0.20 mmol), octa-1,7-dien-3-one (273 mg, 2.20 mmol) and Et_2O (5 mL)/*t*-BuOH (15 mL) to give the title compound as a colourless oil (408 mg, 0.84 mmol, 42%). ^{1}H NMR (400 MHz, $CDCl_3$) δ 1.68 (p, J = 7.3 Hz, 2 H, CH_2C*H*$_2CH_2$C(O)), 2.01 – 2.08 (m, 2 H, C*H*$_2CH_2CH_2$C(O)), 2.35 – 2.46 (m, 4 H, 2 H from CH_2CH_2C*H*$_2$C(O), 2 H from C(O)CH_2C*H*$_2C_{quat}$), 2.65 – 2.71 (m, 2 H, C(O)C*H*$_2CH_2C_{quat}$), 3.46 (s, 2 H, C*H*$_2$Ph), 4.94 – 5.03 (m, 2 H, C*H*$_2$=$CHCH_2$), 5.68 – 5.80 (m, 1 H, CH_2=C*H*CH_2), 6.99 – 7.11 (m, 6 H, ArC*H*), 7.23 – 7.31 (m, 4 H, ArC*H*), 7.37 – 7.44 (m, 4 H, ArC*H*) ppm. ^{13}C NMR (100 MHz, $CDCl_3$) δ 22.9 (CH_2*C*H_2CH_2C(O)), 27.2 (C(O)CH_2*C*H_2C_{quat}), 33.2 (*C*$H_2CH_2CH_2$C(O)), 37.9 (C(O)*C*$H_2CH_2C_{quat}$), 39.1 (*C*H_2Ph), 42.2 (CH_2CH_2*C*H_2C(O)), 58.7 (C(O)CH_2CH_2*C*$_{quat}$), 115.5 (*C*H_2=$CHCH_2$), 115.6 (d, J = 21.3 Hz, Ar*C*H), 121.3 (Ar*C*H), 126.5 (Ar*C*H), 129.8 (Ar*C*H), 131.0 (d, J = 3.4 Hz, Ar*C*), 132.0 (d, J = 8.0 Hz, Ar*C*H), 138.0 (CH_2=*C*HCH_2), 150.5 (Ar*C*-O), 162.40 (d, J = 246.2 Hz, Ar*C*), 169.3 (*C*(O)O), 209.0 (*C*(O)) ppm. ^{19}F NMR (375 MHz, $CDCl_3$) δ -114.95 ppm. IR ν_{max} (neat/cm^{-1}): 2927, 1749, 1715, 1591, 1510, 1492, 1456, 1223, 1195, 1160, 1100, 1070, 1024, 913, 843, 746, 688, 668. HRMS calcd for $C_{30}H_{29}FO_5K$ [M+K]$^+$: 527.1636, found 527.1634.

Diphenyl 2-(3,5-dimethylbenzyl)-2-(3-oxooct-7-en-1-yl)malonate S48

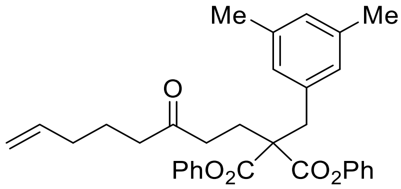

Prepared according to general procedure **F** using diphenyl 2-(3,5-dimethylbenzyl)malonate (749 mg, 2.00 mmol), *t*-BuOK (22 mg, 0.20 mmol), octa-1,7-dien-3-one (273 mg, 2.20 mmol) and Et_2O (5 mL)/*t*-BuOH (15 mL) to give the title compound as a colourless oil (423 mg, 0.85 mmol, 42%). ^1H NMR (400 MHz, $CDCl_3$) δ 1.68 (p, *J* = 7.3 Hz, 2 H, $CH_2CH_2CH_2C(O)$), 2.00 – 2.08 (m, 2 H, $CH_2CH_2CH_2C(O)$), 2.29 (s, 6 H, 2 × ArC-CH_3), 2.36 – 2.47 (m, 4 H, 2 H from $CH_2CH_2CH_2C(O)$, 2 H from $C(O)CH_2CH_2C_{quat}$), 2.65 – 2.70 (m, 2 H, $C(O)CH_2CH_2C_{quat}$), 3.43 (s, 2 H, CH_2Ph), 4.94 – 5.03 (m, 2 H, CH_2=CHCH_2), 5.68 – 5.80 (m, 1 H, CH_2=CHCH_2), 6.90 (s, 2 H, ArC*H*), 6.93 (s, 1 H, ArC*H*), 7.06 – 7.11 (m, 4 H, ArC*H*), 7.24 – 7.29 (m, 2 H, ArC*H*), 7.37 – 7.43 (m, 4 H, ArC*H*) ppm. ^{13}C NMR (100 MHz, $CDCl_3$) δ 21.4 (ArC-CH_3), 22.9 ($CH_2CH_2CH_2C(O)$), 26.9 ($C(O)CH_2CH_2C_{quat}$), 33.2 ($CH_2CH_2CH_2C(O)$), 38.0 ($C(O)CH_2CH_2C_{quat}$), 39.6 (CH_2Ph), 42.2 ($CH_2CH_2CH_2C(O)$), 58.7 ($C(O)CH_2CH_2C_{quat}$), 115.5 (CH_2=CHCH_2), 121.4 (Ar*C*H), 126.4 (Ar*C*H), 128.3 (Ar*C*H), 129.3 (Ar*C*H), 129.7 (Ar*C*H), 135.0 (Ar*C*), 138.0 (Ar*C*), 138.1 (CH_2=CHCH_2), 150.6 (Ar*C*-O), 169.5 (*C*(O)O), 209.2 (*C*(O)) ppm. IR ν_{max} (neat/cm^{-1}): 2923, 2362, 1750, 1716, 1591, 1492, 1456, 1195, 1162, 1070, 744, 688, 668, 649. HRMS calcd for $C_{32}H_{34}O_5Na$ $[M+Na]^+$: 521.2303, found 521.2303.

Diphenyl 2-(4-methoxybenzyl)-2-(3-oxooct-7-en-1-yl)malonate S49

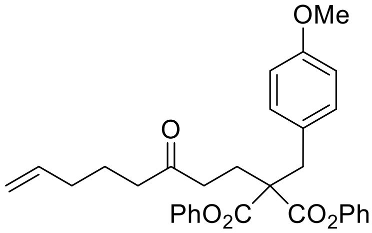

Prepared according to general procedure **F** using diphenyl 2-(4-methoxybenzyl)malonate (0.53 g, 1.43 mmol), *t*-BuOK (15.7 mg, 0.10 mmol), octa-1,7-dien-3-one (0.19 g, 1.54 mmol) and Et_2O (5 mL)/*t*-BuOH (15 mL) to give the title compound as a colourless oil (0.29 g, 0.58 mmol, 41%). ^1H NMR (500 MHz, $CDCl_3$) δ 1.60 (p, *J* = 7.4 Hz, 2 H, $CH_2CH_2CH_2C(O)$), 1.96 (q, *J* = 7.2 Hz, 2 H, $CH_2CH_2CH_2C(O)$), 2.30 (t, *J* = 7.7 Hz, 2 H, $CH_2CH_2CH_2C(O)$, 2.36 (t, *J* = 7.4 Hz, 2 H, $C(O)CH_2CH_2C$), 2.60 (t, *J* = 7.7 Hz, 2 H, $C(O)CH_2CH_2C$), 3.37 (s, 2 H, CH_2Ar), 3.73

(s, 4 H, ArOC*H*$_3$), 4.86 – 4.95 (m, 2 H, C*H*$_2$=CHCH$_2$), 5.66 (ddt, *J* = 16.9, 10.2, 6.7 Hz, 1 H, CH$_2$=C*H*CH$_2$), 6.79 (d, *J* = 8.2 Hz, 2 H, ArC*H*), 7.01 (d, *J* = 8.0 Hz, 4 H, ArC*H*), 7.14 (d, *J* = 8.2 Hz, 2 H, ArC*H*), 7.19 (t, *J* = 7.5 Hz, 2 H, ArC*H*), 7.32 (t, *J* = 7.7 Hz, 4 H, ArC*H*). ^{13}C NMR (126 MHz, CDCl$_3$) δ 22.9 (CH$_2$*C*H$_2$CH$_2$C(O)), 26.9 (C(O)CH$_2$*C*H$_2$C$_{quat}$), 33.1 (*C*H$_2$CH$_2$CH$_2$C(O)), 38.0 (C(O)*C*H$_2$CH$_2$C), 39.0 (*C*H$_2$Ar), 42.1 (CH$_2$CH$_2$*C*H$_2$C(O)), 55.4 (ArO*C*H$_3$), 58.7 (C(O)CH$_2$CH$_2$*C*), 114.1 (Ar*C*H), 115.4 (*C*H$_2$=CHCH$_2$), 121.4 (Ar*C*H), 126.4 (Ar*C*H), 127.1 (ArC), 129.7 (Ar*C*H), 131.4 (Ar*C*H), 138.0 (CH$_2$=*C*HCH$_2$), 150.6 (Ar*C*-O) , 159.1 (Ar*C*-OCH$_3$), 169.5 (*C*(O)O), 209.1 (*C*(O)). IR ν_{max} (neat/cm^{-1}): 2935, 1746, 1714, 1591, 1513, 1492, 1248, 1177, 1161, 1032, 910, 837, 730, 688. HRMS calcd for $C_{31}H_{33}O_6$ [M + H]$^+$: 501.2272, found 501.2268.

Diphenyl 2-(3-oxooct-7-en-1-yl)-2-(thiophen-3-ylmethyl)malonate S50

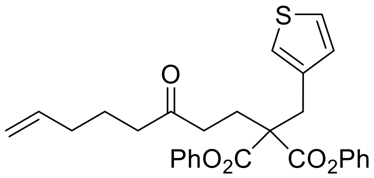

Prepared according to general procedure **F** using diphenyl 2-(thiophen-3-ylmethyl)malonate (0.70 g, 2.00 mmol), *t*-BuOK (0.22 mg, 0.20 mmol), octa-1,7-dien-3-one (0.27 mg, 2.20 mmol) and Et$_2$O (5 mL)/*t*-BuOH (15 mL) to give the title compound as a colourless oil (0.31 mg, 0.66 mmol, 33%). ^{1}H NMR (500 MHz, CDCl$_3$) δ 1.71 (p, *J* = 7.4 Hz, 2 H, CH$_2$C*H*$_2$CH$_2$C(O)), 2.07 (q, *J* = 7.2 Hz, 2 H, C*H*$_2$CH$_2$CH$_2$C(O)), 2.46 (m, 4 H, 2 H from CH$_2$CH$_2$C*H*$_2$C(O), 2 H from C(O)CH$_2$C*H*$_2$C)), 2.70 (t, *J* = 7.7 Hz, 2 H, C(O)C*H*$_2$CH$_2$C), 3.55 (s, 2 H, C*H*$_2$Ar), 4.96 – 5.06 (m, 2 H, C*H*$_2$=CHCH$_2$), 5.77 (ddt, *J* = 16.9, 10.2, 6.7 Hz, 1 H, CH$_2$=C*H*CH$_2$), 7.06 (d, *J* = 4.9 Hz, 1 H, ArC*H*), 7.10 (d, *J* = 8.0 Hz, 4 H, ArC*H*), 7.20 (s, 1 H, ArC*H*), 7.29 (t, *J* = 7.5 Hz, 2 H, ArC*H*), 7.33 (t, *J* = 4.0 Hz, 1 H, ArC*H*), 7.43 (t, *J* = 7.7 Hz, 4 H, ArC*H*). ^{13}C NMR (126 MHz, CDCl$_3$) δ 22.8 (CH$_2$*C*H$_2$CH$_2$C(O)), 27.2 (C(O)CH$_2$*C*H$_2$C$_{quat}$), 33.2 (*C*H$_2$CH$_2$CH$_2$C(O)), 34.3 (*C*H$_2$Ar), 37.9 (C(O)*C*H$_2$CH$_2$C), 42.1 (CH$_2$CH$_2$*C*H$_2$C(O)), 58.3 (C(O)CH$_2$CH$_2$*C*), 115.4 (*C*H$_2$=CHCH$_2$), 121.3 (Ar*C*H), 124.3 (Ar*C*H), 126.0 (Ar*C*H), 126.5 (Ar*C*H), 129.3 (Ar*C*H), 129.8 (Ar*C*H), 135.3 (ArC), 138.0 (CH$_2$=*C*HCH$_2$), 150.5 (Ar*C*-O), 169.5 (*C*(O)O), 209.1 (*C*(O)). IR ν_{max} (neat/cm^{-1}): 2936, 1747, 1714, 1591, 1492, 1155, 1161, 909, 834, 730, 687, 642. HRMS calcd for $C_{28}H_{28}O_5SNa$ [M + Na]$^+$: 477.1730, found 477.1722.

Diphenyl 2-(3-oxooct-7-en-1-yl)-2-(propan-2-yl-2-d)malonate S51

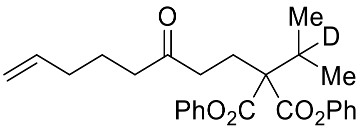

Prepared according to general procedure **F** using diphenyl 2-(propan-2-yl-2-d)malonate (1.20 g, 4.00 mmol), *t*-BuOK (44 mg, 0.20 mmol), octa-1,7-dien-3-one (546 mg, 4.40 mmol) and Et_2O (10 mL)/*t*-BuOH (30 mL) to give the title compound as a colourless oil (440 mg, 1.04 mmol, 26%). ^{1}H NMR (400 MHz, $CDCl_3$) δ 1.24 (s, 6 H, CD(CH_3)$_2$), 1.69 (p, *J* = 7.5 Hz, 2 H, $CH_2CH_2CH_2C(O)$), 2.01 – 2.10 (m, 2 H, C$H_2$$CH_2CH_2C(O)$), 2.40 – 2.48 (m, 4 H, 2 H from CH_2CH_2CH_2C(O), 2 H from C(O)CH_2CH_2C), 2.66 – 2.74 (m, 2 H, C(O)C$H_2$$CH_2$C), 4.95 – 5.04 (m, 2 H, CH_2=$CHCH_2$), 5.75 (ddt, *J* = 16.9, 10.1, 6.7 Hz, 1 H, CH_2=C*H*CH_2), 7.10 – 7.17 (m, 4 H, ArC*H*), 7.24 – 7.31 (m, 2 H, ArC*H*), 7.38 – 7.45 (m, 4 H, ArC*H*) ppm. ^{13}C NMR (100 MHz, $CDCl_3$) δ 18.6 (CD(*C*H_3)$_2$), 22.8 (CH_2*C*$H_2CH_2C(O)$), 27.0 (C(O)CH_2*C*H_2C), 33.1 (*C*$H_2CH_2CH_2C(O)$), 33.3 (1:1:1 t, *J* = 20.1 Hz, *C*D(CH_3)$_2$), 38.3 (C(O)*C*H_2CH_2C), 42.1 (CH_2CH_2*C*H_2C(O)), 61.3 (C(O)CH_2CH_2*C*), 115.3 (*C*H_2=$CHCH_2$), 121.4 (Ar*C*H), 126.3 (Ar*C*H), 129.6 (Ar*C*H), 137.9 (CH_2=*C*HCH_2), 150.4 (Ar*C*), 169.4 (*C*(O)O), 209.4 (*C*(O)) ppm. IR ν_{max} (neat/cm^{-1}): 2940, 1744, 1714, 1640, 1591, 1492, 1456, 1393, 1372, 1292, 1226, 1184, 1161, 1069, 1024, 998, 914, 831, 742, 687. HRMS calcd for $C_{26}H_{29}DO_5K$ [M + K]$^+$: 462.1788, found 462.1784.

Ethyl (*E*)-11-bromo-5-oxoundec-9-enoate 6a'

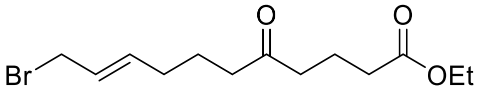

General procedure **G**. To ethyl 5-oxodec-9-enoate (212 mg, 1.00 mmol) in CH_2Cl_2 (1 mL) was added allyl bromide (432 µL, 5.00 mmol) and Hoveyda-Grubbs 2nd generation catalyst (13 mg, 0.020 mmol) in CH_2Cl_2 (1 mL). The reaction was carried out under nitrogen at 40℃ for 4 h after which another portion of HG-II catalyst was added (13 mg, 0.02 mmol) in CH_2Cl_2 (1 mL) and the reaction continued for another 14 h. Volatiles were removed *in vacuo* to give a black oil which was purified by column chromatography eluting with Et_2O/pentane (7:93) to give a yellow oil, which was dissolved in Et_2O, stirred with activated carbon, filtered through a pad of celite and concentrated *in vacuo* to give the title compound as colourless oil and as a mixture of (83:17) E/Z isomers (253 mg, 0.83 mmol, 83%). Spectroscopic data for major (*E*) isomer: ^{1}H NMR (400 MHz, $CDCl_3$) δ 1.26 (t, *J* = 7.1 Hz, 3 H, OCH_2CH_3), 1.68 (p, *J* = 7.4 Hz, 2 H,

$CH{=}CHCH_2C\mathit{H}_2CH_2$), 1.85 – 1.94 (m, 2 H, $CH_2C\mathit{H}_2CH_2C(O)OEt$), 2.03 – 2.11 (m, 2 H, $CH{=}CHC\mathit{H}_2CH_2CH_2$), 2.32 (t, *J* = 7.3 Hz, 2 H, $CH_2CH_2C\mathit{H}_2C(O)OEt$), 2.41 (t, *J* = 7.3 Hz, 2 H, $CH{=}CHCH_2CH_2C\mathit{H}_2$), 2.48 (t, *J* = 7.3 Hz, 2 H, $C\mathit{H}_2CH_2CH_2C(O)OEt$), 3.93 – 3.96 (m, 2 H, $BrC\mathit{H}_2CH{=}CH$), 4.13 (q, *J* = 7.2 Hz, 2 H, $OC\mathit{H}_2CH_3$), 5.63 – 5.79 (m, 2 H, $BrCH_2C\mathit{H}{=}C\mathit{H}$) ppm. ^{13}C NMR (100 MHz, $CDCl_3$) δ 14.3 ($OCH_2\mathit{C}H_3$), 18.9 ($CH_2\mathit{C}H_2CH_2C(O)OEt$), 22.7 ($CH{=}CHCH_2\mathit{C}H_2CH_2$), 31.4 ($CH{=}CH\mathit{C}H_2CH_2CH_2$), 33.2 ($Br\mathit{C}H_2CH{=}CH$), 33.3 ($CH_2CH_2\mathit{C}H_2C(O)OEt$), 41.7 ($CH{=}CHCH_2CH_2\mathit{C}H_2$), 41.8 ($\mathit{C}H_2CH_2CH_2C(O)OEt$), 60.4 ($O\mathit{C}H_2CH_3$), 127.3 ($BrCH_2\mathit{C}H{=}CH$), 135.4 ($BrCH_2CH{=}\mathit{C}H$), 173.2 (*C*(O)OEt), 209.8 (*C*(O)) ppm. IR ν_{max} (neat/cm^{-1}): 2939, 1729, 1711, 1659, 1446, 1412, 1373, 1311, 1203, 1178, 1097, 1030, 965, 858, 754. HRMS calcd for $C_{13}H_{22}BrO_3$ $[M + H]^+$: 305.0752, found: 305.0750.

(*E*)-1-((*tert*-Butyldimethylsilyl)oxy)-10,14-dioxo-14-phenoxytetradec-5-en-4-yl 4-(trifluoromethyl)benzoate 6b’

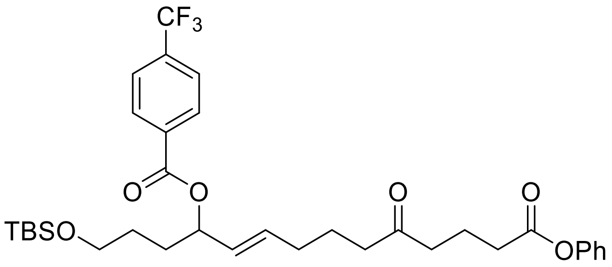

Prepared according to general procedure **G** using diphenyl phenyl 5-oxodec-9-enoate (261 mg, 1.00 mmol), 6-((*tert*-butyldimethylsilyl)oxy)hex-1-en-3-yl 4-(trifluoromethyl)benzoate (1.2 g, 3.00 mmol), Hoveyda-Grubbs 2nd generation catalyst (13 mg, 0.020 mmol) added in two portions and CH_2Cl_2 (5 mL) and purified by column chromatography eluting with EtOAc/Hexane (10:90) to give the title compound as a colourless oil (450 mg, 0.70 mmol, 70%). ^{1}H NMR (400 MHz, $CDCl_3$) δ 0.04 (s, 6 H, $Si(C\mathit{H}_3)_2$), 0.89 (s, 9 H, $SiC(C\mathit{H}_3)_3$), 1.59 (d, *J* = 8.5 Hz, 2 H, $OCH_2C\mathit{H}_2CH_2$), 1.68 (p, *J* = 7.5 Hz, 2 H, $CH_2C\mathit{H}_2CH_2C(O)$), 1.73 – 1.89 (m, 2 H, $OCH_2CH_2C\mathit{H}_2$), 1.94 – 2.11 (m, 4 H, $CH_2C\mathit{H}_2CH_2C(O)OPh$, $CH{=}CHC\mathit{H}_2CH_2CH_2$), 2.41 (t, *J* = 7.4 Hz, 2 H, $CH_2{=}CHCH_2CH_2C\mathit{H}_2$), 2.55 (dt, *J* = 15.6, 7.2 Hz, 4 H, $CH_2CH_2C\mathit{H}_2C(O)OPh$, $C\mathit{H}_2CH_2CH_2C(O)OPh$), 3.64 (t, *J* = 6.3 Hz, 2 H, $OC\mathit{H}_2$), 5.43 – 5.57 (m, 2 H, $C\mathit{H}CH{=}CH$, $CHC\mathit{H}{=}CH$), 5.76 (dt, *J* = 14.6, 6.7 Hz, 1 H, $CHCH{=}C\mathit{H}$), 7.03 – 7.09 (m, 2H, ArC*H*), 7.18 – 7.25 (m, 1 H, ArC*H*), 7.37 (dd, *J* = 8.4, 7.4 Hz, 2 H, ArCH), 7.65 – 7.73 (m, 2 H, ArCH), 8.10 – 8.18 (m, 2 H, ArC*H*) ppm. ^{13}C NMR (100 MHz, $CDCl_3$) δ -5.2 ($Si(\mathit{C}H_3)_2$), 18.5 ($C(O)CH_2\mathit{C}H_2CH_2$), 18.9 ($Si\mathit{C}(CH_3)_3$), 23.0 ($OCH_2CH_2\mathit{C}H_2$), 26.1 ($SiC(\mathit{C}H_3)_3$), 28.6 ($CH_2\mathit{C}H_2CH_2C(O)$), 31.2 ($OCH_2\mathit{C}H_2CH_2$), 31.6 ($\mathit{C}H_2CH_2CH_2C(O)$), 33.4

($C(O)\mathit{C}H_2CH_2CH_2$), 41.5 ($C(O)CH_2CH_2\mathit{C}H_2$), 42.1 ($CH_2CH_2\mathit{C}H_2C(O)$), 62.8 ($O\mathit{C}H_2CH_2CH_2$), 76.1 ($\mathit{C}HCH{=}CH$), 121.6 (Ar*C*H), 125.5 (Ar*C*H), 125.9 (Ar*C*H), 129.0 ($CH\mathit{C}H{=}CH$), 129.5, (Ar*C*H), 130.1 (Ar*C*H), 133.9 ($CHCH{=}\mathit{C}H$), 134.1 ($\mathit{C}F_3$), 134.3 (Ar*C*), 134.6 (Ar*C*) 150.7 (Ar*C*), 164.9 (*C*(O)OAr), 171.8 (*C*(O)OPh), 209.8 (*C*(O)) ppm. IR ν_{max} (neat/cm^{-1}): 2952, 2179, 1721, 1365, 1325, 1200, 1133, 1165, 1101, 909, 733, 616. HRMS calcd for $C_{34}H_{45}F_3O_6Na$ [M + Na]$^+$: 657.2830, found 657.2800.

(*E*)-9,13-dioxo-13-phenoxy-1-phenyltridec-4-en-3-yl 4-(trifluoromethyl)benzoate 6c'

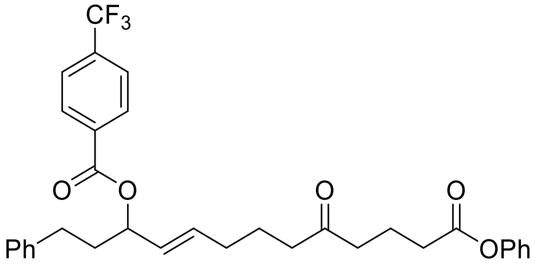

Prepared according to general procedure **G** using phenyl 5-oxodec-9-enoate (0.100 g, 0.38 mmol), 5-phenylpent-1-en-3-yl 4-(trifluoromethyl)benzoate (390 mg, 1.15 mmol), Hoveyda-Grubbs 2nd generation catalyst (5 mg, 0.0076 mmol; added in two portions) and CH_2Cl_2 (2 mL), to give the title compound as a colourless oil (100 mg, 0.18 mmol, 48%). ^{1}H NMR (400 MHz, $CDCl_3$) δ 1.69 (p, *J* = 7.4 Hz, 2 H, $CH_2C\mathit{H_2}CH_2C(O)$), 1.94 – 2.21 (m, 6 H, $C(O)CH_2C\mathit{H_2}CH_2$, $C\mathit{H_2}CH_2CH_2C(O)$, O-$CHC\mathit{H_2}CH_2$), 2.41 (t, *J* = 7.3 Hz, 2 H, $CH_2CH_2C\mathit{H_2}C(O)$), 2.49 – 2.61 (m, 4 H, $C(O)C\mathit{H_2}CH_2CH_2$, O-$CHCH_2C\mathit{H_2}$), 2.71 (t, *J* = 7.9 Hz, 2 H, $C(O)CH_2CH_2C\mathit{H_2}$), 5.44 – 5.59 (m, 2 H, $C\mathit{H}CH_2CH_2$, $CHCH{=}C\mathit{H}$), 5.78 (dt, *J* = 15.1, 6.7 Hz, 1 H, $CHC\mathit{H}{=}CH$), 7.04 – 7.09 (m, 2 H, ArC*H*), 7.14 – 7.21 (m, 3 H, ArC*H*), 7.23 – 7.30 (m, 3 H, ArC*H*), 7.37 (dd, *J* = 8.6, 7.3 Hz, 2 H, ArC*H*), 7.70 (d, *J* = 8.4 Hz, 2 H, ArC*H*), 8.12 (d, *J* = 8.0 Hz, 2 H, ArC*H*) ppm. ^{13}C NMR (100 MHz, $CDCl_3$) δ 18.9 ($C(O)CH_2\mathit{C}H_2CH_2$), 23.0 ($CH_2\mathit{C}H_2CH_2C(O)$), 31.7 ($\mathit{C}H_2CH_2CH_2C(O)$), 31.7 ($C(O)CH_2CH_2\mathit{C}H_2$), 33.4 ($CHCH_2\mathit{C}H_2$), 36.3 ($CH\mathit{C}H_2CH_2$), 41.5 ($C(O)\mathit{C}H_2CH_2CH_2$), 42.1 ($CH_2CH_2\mathit{C}H_2C(O)$), 75.8 ($\mathit{C}HCH_2CH_2$), 121.6 (Ar*C*H), 125.5 (Ar*C*H), 126.0 (Ar*C*H), 126.2 (Ar*C*H), 128.5 (Ar*C*H), 128.6 ($CHCH{=}C\mathit{H}$), 128.7 (Ar*C*H), 129.6 (Ar*C*H), 130.1 (Ar*C*H), 134.0 (ArC), 134.3 ($CH\mathit{C}H{=}CH$), 134.7 (Ar*C*), 141.3 (Ar*C*), 150.7 (Ar*C*), 164.8 (*C*(O)OAr), 171.8 (*C*(O)OPh), 209.8 (*C*(O)) ppm. $\mathit{C}F_3$ not observed. IR ν_{max} (neat/cm^{-1}): 2970, 2170, 1739, 1365, 1325, 1273, 1217, 1132, 909, 732 610. HRMS calcd for $C_{33}H_{33}F_3O5Cl$ [M + Cl]$^-$: 601.1974, found 601.1984.

(*E*)-1-(Benzofuran-2-yl)-8,12-dioxo-12-phenoxydodec-3-en-2-yl 4-(trifluoromethyl)benzoate 6d'

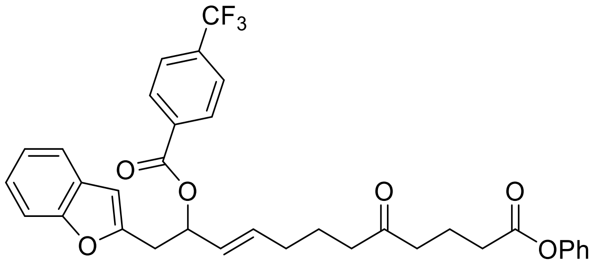

Prepared according to general procedure **G** using phenyl phenyl 5-oxodec-9-enoate (14 mg, 0.054 mmol), 1-(benzofuran-2-yl)but-3-en-2-yl 4-(trifluoromethyl)benzoate (0.7 mg, 0.0016 mmol), Hoveyda-Grubbs 2nd generation catalyst (5 mg, 0.0076 mmol; added in two portions) and CH_2Cl_2 (0.5 mL), to give the title compound as a colourless oil (17 mg, 0.029 mmol, 55%). ^{1}H NMR (400 MHz, $CDCl_3$) δ 1.64 (p, *J* = 8.4, 7.9 Hz, 2 H, $CH_2CH_2CH_2C(O)$ with italic middle H, i.e. CH2C*H*2CH2C(O)), 1.91 – 2.09 (m, 4 H, C(O)CH2C*H*2CH2, C*H*2CH2CH2C(O)), 2.24 – 2.36 (m, 2 H, C(O)C*H*2CH2CH2), 2.45 (t, *J* = 7.1 Hz, 2 H, CH2CH2C*H*2C(O)), 2.56 (t, *J* = 7.2 Hz, 2 H, C(O)CH2CH2C*H*2), 3.22 (dd, *J* = 15.2, 6.0 Hz, 1H, CHC*H*2), 3.29 (dd, *J* = 15.1, 7.0 Hz, 1H, CHC*H*2), 5.50 – 5.67 (m, 1 H, CHC*H*=CH), 5.83 (dq, *J* = 18.3, 6.7 Hz, 2 H, C*H*, CHCH=C*H*), 6.49 (s, 1 H, ArC*H*), 7.03 – 7.11 (m, 2 H, ArC*H*), 7.14 – 7.27 (m, 3 H, ArC*H*), 7.38 (td, *J* = 8.0, 6.4 Hz, 3 H, ArC*H*), 7.48 (dd, *J* = 7.5, 1.6 Hz, 1 H, ArC*H*), 7.69 (d, *J* = 8.2 Hz, 2 H, ArC*H*), 8.14 (d, *J* = 8.1 Hz, 2 H, ArC*H*) ppm. ^{13}C NMR (100 MHz, $CDCl_3$) δ 18.9 (C(O)CH2*C*H2CH2), 22.8 (CH2*C*H2CH2C(O)), 31.6 (*C*H2CH2CH2C(O)), 33.4 (C(O)CH2CH2*C*H2), 34.2 (CH*C*H2), 41.4 (C(O)*C*H2CH2CH2), 41.8 (CH2CH2*C*H2C(O)), 74.0 (C*H*CH2), 104.5 (Ar*C*H), 111.0 (Ar*C*H), 120.6 (Ar*C*H), 121.6 (Ar*C*H), 122.4 (Ar*C*), 122.8 (Ar*C*H), 123.8 (Ar*C*H), 125.5 (Ar*C*H), 126.0 (Ar*C*H), 127.8 (CH*C*H=CH), 128.7 (Ar*C*), 129.6 (Ar*C*H), 130.2 (Ar*C*H), 134.4 (Ar*C*), 135.0 (CHCH=*C*H), 150.7 (Ar*C*), 154.2 (Ar*C*), 154.9 (Ar*C*), 164.6 (*C*(O)OAr), 171.8 (*C*(O)OPh), 209.8 (*C*(O)) ppm *C*F_3 not observed. IR ν_{max} (neat/cm^{-1}): 2970, 1739, 1365, 1217, 688, 571. $C_{34}H_{31}F_3O_6Na$ $[M + Na]^+$: 615.1965, found 615.1968.

Diphenyl (*E*)-2-(9-bromo-3-oxonon-7-en-1-yl)-2-phenylmalonate 6a

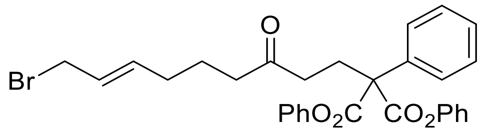

Prepared according to general procedure **G** using diphenyl 2-(3-oxooct-7-en-1-yl)-2-phenylmalonate (200 mg, 0.44 mmol), allyl bromide (194 µL, 2.20 mmol), Hoveyda-Grubbs 2nd generation catalyst (18 mg, 0.02 mmol; added in two portions) and CH_2Cl_2 (2 mL) to give the title compound as a colourless oil and as a mixture of (83:17) E/Z isomers (156 mg, 0.28 mmol, 65%). Spectroscopic data for major (*E*) isomer: ^{1}H NMR (400 MHz, $CDCl_3$) δ 1.65 (p, *J* = 7.4 Hz, 2 H, $CH_2CH_2CH_2C(O)$), 1.99 – 2.08 (m, 2 H, $CH_2CH_2CH_2C(O)$), 2.33 – 2.42 (m, 2 H, $CH_2CH_2CH_2C(O)$), 2.55 – 2.63 (m, 2 H, $C(O)CH_2CH_2C$), 2.86 – 2.94 (m, 2 H, $C(O)CH_2CH_2C$), 3.92 – 3.95 (m, 2 H, $BrCH_2$), 5.62 – 5.73 (m, 2 H, C*H*=C*H*), 7.07 – 7.15 (m, 4 H, ArC*H*), 7.23 – 7.30 (m, 2 H, ArC*H*), 7.36 – 7.49 (m, 7 H, ArC*H*), 7.58 – 7.64 (m, 2 H, ArC*H*) ppm. ^{13}C NMR (100 MHz, $CDCl_3$) δ 22.7 ($CH_2CH_2CH_2C(O)$), 29.4 ($C(O)CH_2CH_2C$), 31.3 ($CH_2CH_2CH_2C(O)$), 33.2 ($BrCH_2$), 38.1 ($C(O)CH_2CH_2C$), 41.9 ($CH_2CH_2CH_2C(O)$), 62.1 ($C(O)CH_2CH_2C$), 121.2 (Ar*C*H), 126.4 (Ar*C*H), 127.3 ($BrCH_2CH$=CH), 128.0 (Ar*C*H), 128.3 (Ar*C*H), 128.7 (Ar*C*H), 129.6 (Ar*C*H), 135.3 ($BrCH_2CH$=*C*H), 135.6 (Ar*C*), 150.5 (Ar*C*-O), 168.9 (*C*(O)O), 208.8 (*C*(O)) ppm. IR ν_{max} (neat/cm^{-1}): 3062, 2935, 1748, 1713, 1640, 1591, 1491, 1456, 1447, 1414, 1371, 1292, 1182, 1160, 1069, 1023, 1002, 911, 832, 794, 744, 686. HRMS calcd for $C_{30}H_{29}BrO_5Na$ [M + Na]$^+$: 571.1091, found 571.1085

Diphenyl (*E*)-2-(9-bromo-3-oxonon-7-en-1-yl)-2-ethylmalonate 6b

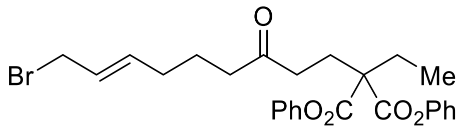

Prepared according to general procedure **G** using diphenyl 2-ethyl-2-(3-oxooct-7-en-1-yl)malonate (195 mg, 0.48 mmol), allyl bromide (206 µL, 2.40 mmol), Hoveyda-Grubbs 2nd generation catalyst (18 mg, 0.020 mmol; added in two portions) and CH_2Cl_2 (2 mL) to give the title compound as a colourless oil and as a mixture of (83:17) E/Z isomers (146 mg, 0.29 mmol, 61%). Spectroscopic data for major (*E*) isomer: ^{1}H NMR (400 MHz, $CDCl_3$) δ 1.09 (t, *J* = 7.5 Hz, 3 H, CH_2CH_3), 1.70 (p, *J* = 7.4 Hz, 2 H, $CH_2CH_2CH_2C(O)$), 2.04 – 2.11 (m, 2 H, $CH_2CH_2CH_2C(O)$), 2.22 (q, *J* = 7.5 Hz, 2 H, CH_2CH_3), 2.39 – 2.52 (m, 4 H, 2 H from $CH_2CH_2CH_2C(O)$, 2 H from $C(O)CH_2CH_2C$), 2.58 – 2.68 (m, 2 H, $C(O)CH_2CH_2C$), 3.91 – 3.95 (m, 2 H, $BrCH_2$), 5.64 – 5.77 (m, 2 H, C*H*=C*H*), 7.09 – 7.16 (m, 4 H, ArC*H*), 7.24 – 7.31

(m, 2 H, ArC*H*), 7.37 – 7.45 (m, 4 H, ArC*H*). ^{13}C NMR (100 MHz, $CDCl_3$) δ 8.7 (CH_2*C*H_3), 22.7 (CH_2*C*H_2CH_2C(O)), 26.1 (*C*H_2CH_3), 26.7 (C(O)CH_2*C*H_2C), 31.3 (*C*$H_2CH_2CH_2$C(O)), 33.2 (Br*C*H_2), 37.7 (C(O)*C*H_2CH_2C), 41.9 (CH_2CH_2*C*H_2C(O)), 57.7 (C(O)CH_2CH_2*C*), 121.3 (Ar*C*H), 126.3 (Ar*C*H), 127.3 (BrCH_2*C*H=CH), 129.6 (Ar*C*H), 135.3 (BrCH_2CH=*C*H), 150.5 (Ar*C*), 169.8 (*C*(O)O), 208.8 (*C*(O)) ppm. IR ν_{max} (neat/cm^{-1}): 2943, 1746, 1713, 1590, 1492, 1456, 1372, 1293, 1183, 1161, 1097, 1069, 1023, 965, 926, 831, 743, 687. HRMS calcd for $C_{26}H_{29}BrO_5K$ [M + K]$^+$: 539.0830, found 539.0832.

Diphenyl (*E*)-2-(9-bromo-3-oxonon-7-en-1-yl)-2-methylmalonate 6c

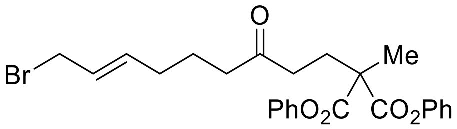

Prepared according to general procedure **G** using diphenyl 2-methyl-2-(3-oxooct-7-en-1-yl)malonate (255 mg, 0.65 mmol), allyl bromide (279 µL, 3.24 mmol), Hoveyda-Grubbs 2nd generation catalyst (16 mg, 0.030 mmol; added in two portions) and CH_2Cl_2 (2 mL), to give the title compound as a colourless oil and as a mixture of (83:17) E/Z isomers (206 mg, 0.42 mmol, 65%). Spectroscopic data for major (*E*) isomer: ^{1}H NMR (400 MHz, $CDCl_3$) δ 1.69 (p, J = 7.3 Hz, 2 H, CH_2C*H*$_2CH_2$C(O)), 1.72 (s, 3 H, C*H*$_3$), 2.05 – 2.11 (m, 2 H, C*H*$_2CH_2CH_2$C(O)), 2.40 – 2.51 (m, 4 H, 2 H from CH_2CH_2C*H*$_2$C(O), 2 H from C(O)CH_2C*H*$_2$C), 2.64 – 2.71 (m, 2 H, C(O)C*H*$_2CH_2$C), 3.92 – 3.95 (m, 2 H, BrC*H*$_2$), 5.65 – 5.77 (m, 2 H, C*H*=C*H*), 7.10 – 7.16 (m, 4 H, ArC*H*), 7.25 – 7.30 (m, 2 H, ArC*H*), 7.37 – 7.45 (m, 4 H, ArC*H*). ^{13}C NMR (100 MHz, $CDCl_3$) δ 20.7 (*C*H_3), 22.7 (CH_2*C*H_2CH_2C(O)), 29.4 (C(O)CH_2*C*H_2C), 31.3 (*C*$H_2CH_2CH_2$C(O)), 33.2 (Br*C*H_2), 37.9 (C(O)*C*H_2CH_2C), 41.9 (CH_2CH_2*C*H_2C(O)), 53.4 (C(O)CH_2CH_2*C*), 121.2 (Ar*C*H), 126.3 (Ar*C*H), 127.3 (BrCH_2*C*H=CH), 129.6 (Ar*C*H), 135.3 (BrCH_2CH=*C*H), 150.5 (Ar*C*), 170.3 (*C*(O)O), 208.8 (*C*(O)) ppm. IR ν_{max} (neat/cm^{-1}): 2941, 1748, 1713, 1590, 1492, 1456, 1412, 1380, 1292, 1185, 1160, 1085, 1069, 1023, 1005, 966, 923, 830, 744, 687. HRMS calcd for $C_{25}H_{27}BrO_5K$ [M + K]$^+$: 525.0673, found 525.0680.

Diphenyl (*E*)-2-(9-bromo-3-oxonon-7-en-1-yl)-2-isopropylmalonate 6d

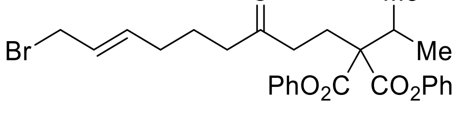

Prepared according to general procedure **G** using diphenyl 2-isopropyl-2-(3-oxooct-7-en-1-yl)malonate (410 mg, 0.97 mmol), allyl bromide (432 µL, 5.00 mmol), Hoveyda-Grubbs 2nd

generation catalyst (26 mg, 0.040 mmol; added in two portions) and CH_2Cl_2 (3 mL), to give the title compound as a colourless oil and as a mixture of (83:17) E/Z isomers (333 mg, 0.65 mmol, 67%). Spectroscopic data for major (*E*) isomer: ^{1}H NMR (400 MHz, $CDCl_3$) δ 1.24 (d, *J* = 6.8 Hz, 6 H, CH(C*H*$_3$)$_2$), 1.69 (p, *J* = 7.3 Hz, 2 H, CH_2C*H*$_2$$CH_2$C(O)), 2.02 – 2.10 (m, 2 H, C*H*$_2$$CH_2CH_2$C(O)), 2.39 – 2.49 (m, 4 H, 2 H from CH_2CH_2C*H*$_2$C(O), 2 H from C(O)CH_2C*H*$_2$C), 2.60 (hept, *J* = 6.9 Hz, 1 H, C*H*(CH_3)$_2$), 2.67 – 2.74 (m, 2 H, C(O)C*H*$_2$$CH_2$C), 3.90 – 3.95 (m, 2 H, BrC*H*$_2$), 5.64 – 5.73 (m, 2 H, C*H*=C*H*), 7.10 – 7.17 (m, 4 H, ArC*H*), 7.24 – 7.31 (m, 2 H, ArC*H*), 7.38 – 7.46 (m, 4 H, ArC*H*) ppm. ^{13}C NMR (100 MHz, $CDCl_3$) δ 18.7 (CH(*C*H$_3$)$_2$), 22.7 (CH_2*C*H$_2$$CH_2$C(O)), 27.0 (C(O)$CH_2$*C*H$_2$C), 31.3 (*C*H$_2$$CH_2CH_2$C(O)), 33.2 (Br*C*H$_2$), 33.4 (*C*H($CH_3$)$_2$), 38.4 (C(O)*C*H$_2$$CH_2$C), 41.9 ($CH_2CH_2$*C*H$_2$C(O)), 61.4 (C(O)$CH_2CH_2$*C*), 121.4 (Ar*C*H), 126.3 (Ar*C*H), 127.3 (BrCH_2*C*H=CH), 129.6 (Ar*C*H), 135.4 (BrCH_2CH=*C*H), 150.4 (Ar*C*), 169.4 (*C*(O)O), 209.1 (*C*(O)) ppm. IR ν_{max} (neat/cm^{-1}): 2941, 1743, 1713, 1591, 1491, 1456, 1392, 1372, 1291, 1184, 1160, 1111, 1069, 1023, 965, 916, 831, 741, 687. HRMS calcd for $C_{27}H_{31}BrO_5K$ [M + K]$^+$: 553.0986, found 553.0990.

Diphenyl (*E*)-2-(9-bromo-3-oxonon-7-en-1-yl)-2-cyclopentylmalonate 6e

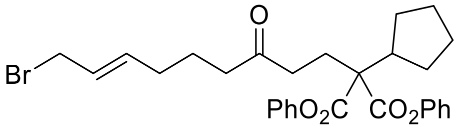

Prepared according to general procedure **G** using diphenyl 2-cyclopentyl-2-(3-oxooct-7-en-1-yl)malonate (405 mg, 0.91 mmol), allyl bromide (400 µL, 4.54 mmol), Hoveyda-Grubbs 2nd generation catalyst (25 mg, 0.04 mmol; added in two portions) and CH_2Cl_2 (3 mL), to give the title compound as a colourless oil and as a mixture of (83:17) E/Z isomers (356 mg, 0.66 mmol, 72%). Spectroscopic data for major (*E*) isomer: ^{1}H NMR (400 MHz, $CDCl_3$) δ 1.59 – 1.79 (m, 8 H, 6 H from 3 × C*H*$_2$, 2 H from CH_2C*H*$_2$$CH_2$C(O)), 1.95 – 2.11 (m, 4 H, 2 H from C*H*$_2$, 2 H from C*H*$_2$$CH_2CH_2$C(O)), 2.38 – 2.50 (m, 4 H, 2 H from CH_2CH_2C*H*$_2$C(O), 2 H from C(O)CH_2C*H*$_2$C), 2.66 – 2.78 (m, 3 H, 1 H from C*H*(CH_2)$_2$, 2 H from C(O)C*H*$_2$$CH_2$C), 3.90 – 3.95 (m, 2 H, C*H*$_2$Br), 5.63 – 5.77 (m, 2 H, C*H*=C*H*), 7.09 – 7.17 (m, 4 H, ArC*H*), 7.23 – 7.31 (m, 2 H, ArC*H*), 7.36 – 7.45 (m, 4 H, ArC*H*) ppm. ^{13}C NMR (100 MHz, $CDCl_3$) δ 22.7 (CH_2*C*H$_2$$CH_2$C(O)), 25.7 (*C*H$_2$), 27.9 (C(O)$CH_2$*C*H$_2$C), 28.3 (*C*H$_2$), 31.3 (*C*H$_2$$CH_2CH_2$C(O)), 33.2 (Br*C*H$_2$), 38.4 (C(O)*C*H$_2$$CH_2$C), 41.9 ($CH_2CH_2$*C*H$_2$C(O)), 44.5 (*C*H($CH_2$)$_2$), 60.2 (C(O)$CH_2CH_2$*C*), 121.4 (Ar*C*H), 126.3 (Ar*C*H), 127.3 (BrCH_2*C*H=CH), 129.6 (Ar*C*H), 135.4 (BrCH_2CH=*C*H), 150.4 (Ar*C*), 169.6 (*C*(O)O), 209.2 (*C*(O)) ppm. IR ν_{max} (neat/cm^{-1}): 2952,

1744, 1713, 1591, 1491, 1456, 1291, 1187, 1161, 1069, 1024, 966, 743, 688. HRMS calcd for $C_{29}H_{33}BrO_5Na$ [M + Na]$^+$: 563.1404, found 563.1411.

Diphenyl (*E*)-2-(9-bromo-3-oxonon-7-en-1-yl)-2-cyclohexylmalonate 6f

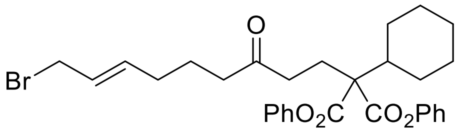

Prepared according to general procedure **G** using diphenyl 2-cyclohexyl-2-(3-oxooct-7-en-1-yl)malonate (300 mg, 0.65 mmol), allyl bromide (287 µL, 3.25 mmol), Hoveyda-Grubbs 2nd generation catalyst (16 mg, 0.03 mmol; added in two portions) and CH_2Cl_2 (2 mL), to give the title compound as a colourless oil and as a mixture of (83:17) E/Z isomers (219 mg, 0.39 mmol, 61%). Spectroscopic data for major (*E*) isomer: ^{1}H NMR (400 MHz, $CDCl_3$) δ 1.15 – 1.33 (m, 1 H, C*H*$_2$), 1.33 – 1.45 (m, 4 H, 2 × C*H*$_2$), 1.63 – 1.79 (m, 3 H, 1 H from C*H*$_2$, 2 H from CH$_2$C*H*$_2$CH$_2$C(O)), 1.82 – 1.92 (m, 2 H, C*H*$_2$), 1.98 – 2.10 (m, 4 H, 2 H from C*H*$_2$, 2 H from C*H*$_2$CH$_2$CH$_2$C(O)), 2.16 – 2.28 (m, 1 H, C*H*(CH$_2$)$_2$), 2.40 – 2.50 (m, 4 H, 2 H from CH$_2$CH$_2$C*H*$_2$C(O), 2 H from C(O)C*H*$_2$CH$_2$C), 2.64 – 2.71 (m, 2 H, C(O)CH$_2$C*H*$_2$C), 3.90 – 3.95 (m, 2 H, BrC*H*$_2$), 5.62 – 5.77 (m, 2 H, C*H*=C*H*), 7.09 – 7.17 (m, 4 H, ArC*H*), 7.24 – 7.31 (m, 2 H, ArC*H*), 7.38 – 7.46 (m, 4 H, ArC*H*). ^{13}C NMR (100 MHz, $CDCl_3$) δ 22.7 (CH$_2$*C*H$_2$CH$_2$C(O)), 26.4 (*C*H$_2$), 26.9 (C(O)CH$_2$*C*H$_2$C), 27.0 (*C*H$_2$), 28.8 (*C*H$_2$), 31.3 (*C*H$_2$CH$_2$CH$_2$C(O)), 33.2 (Br*C*H$_2$), 38.4 (C(O)*C*H$_2$CH$_2$C), 41.9 (CH$_2$CH$_2$*C*H$_2$C(O)), 43.8 (*C*H(CH$_2$)$_2$), 61.3 (C(O)CH$_2$CH$_2$*C*), 121.4 (Ar*C*H), 126.3 (Ar*C*H), 127.3 (BrCH$_2$*C*H=CH), 129.6 (Ar*C*H), 135.4 (BrCH$_2$CH=*C*H), 150.4 (Ar*C*), 169.3 (*C*(O)O), 209.1 (*C*(O)) ppm. IR ν_{max} (neat/cm^{-1}): 2930, 2853, 1743, 1714, 1591, 1491, 1450, 1184, 1160, 1135, 1069, 1023, 1003, 965, 831, 744, 687. HRMS calcd for $C_{30}H_{35}BrO_5K$ [M + K]$^+$: 593.1299, found 593.1308.

Diphenyl (*E*)-2-(9-bromo-3-oxonon-7-en-1-yl)-2-cycloheptylmalonate 6g

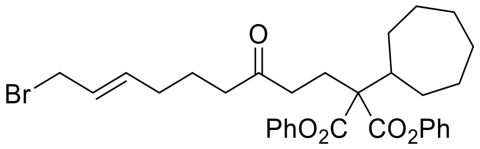

Prepared according to general procedure **G** using diphenyl 2-cycloheptyl-2-(3-oxooct-7-en-1-yl)malonate (400 mg, 0.84 mmol), allyl bromide (371 µL, 4.20 mmol), Hoveyda-Grubbs 2nd generation catalyst (26 mg, 0.040 mmol; added in two portions) and CH_2Cl_2 (3 mL), to give the title compound as a colourless oil and as a mixture of (83:17) E/Z isomers (352 mg, 0.62 mmol, 74%). Spectroscopic data for major (*E*) isomer: ^{1}H NMR (400 MHz, $CDCl_3$) δ 1.52 –

1.72 (m, 10 H, 8 H from 4 × C*H*$_2$, 2 H from CH$_2$C*H*$_2$CH$_2$C(O)), 1.76 – 1.90 (m, 2 H, C*H*$_2$), 1.95 – 2.11 (m, 4 H, 2 H from C*H*$_2$, 2 H from C*H*$_2$CH$_2$CH$_2$C(O)), 2.34 – 2.51 (m, 5 H, 1 H from C*H*(CH$_2$)$_2$, 2 H from CH$_2$CH$_2$C*H*$_2$C(O), 2 H from C(O)CH$_2$C*H*$_2$C), 2.67 – 2.76 (m, 2 H, C(O)C*H*$_2$CH$_2$C), 3.90 – 3.95 (m, 2 H, BrC*H*$_2$), 5.63 – 5.77 (m, 2 H, C*H*=C*H*), 7.09 – 7.16 (m, 4 H, ArC*H*), 7.23 – 7.31 (m, 2 H, ArC*H*), 7.37 – 7.46 (m, 4 H, ArC*H*) ppm. ^{13}C NMR (100 MHz, CDCl$_3$) δ 22.7 (CH$_2$*C*H$_2$CH$_2$C(O)), 27.4 (C(O)CH$_2$*C*H$_2$C), 27.7 (*C*H$_2$), 27.9 (*C*H$_2$), 30.5 (*C*H$_2$), 31.3 (*C*H$_2$CH$_2$CH$_2$C(O)), 33.2 (Br*C*H$_2$), 38.6 (C(O)*C*H$_2$CH$_2$C), 41.9 (CH$_2$CH$_2$*C*H$_2$C(O)), 44.7 (*C*H(CH$_2$)$_2$), 62.7 (C(O)CH$_2$CH$_2$*C*), 121.4 (Ar*C*H), 126.3 (Ar*C*H), 127.3 (BrCH$_2$*C*H=CH), 129.6 (Ar*C*H), 135.4 (BrCH$_2$CH=*C*H), 150.4 (Ar*C*), 169.7 (*C*(O)O), 209.3 (*C*(O)) ppm. IR ν_{max} (neat/cm^{-1}): 2921, 2854, 1743, 1713, 1591, 1491, 1456, 1292, 1186, 1160, 1068, 1023, 965, 918, 830, 743, 687. HRMS calcd for $C_{31}H_{37}BrO_5K$ [M + K]$^+$: 607.1456, found 607.1465.

Diphenyl (*E*)-2-(9-bromo-3-oxonon-7-en-1-yl)-2-(tetrahydro-2H-pyran-4-yl)malonate 6h

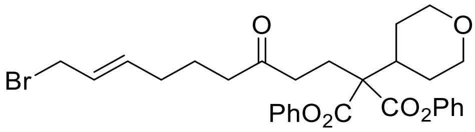

Prepared according to general procedure **G** using diphenyl 2-cyclohexyl-2-(3-oxooct-7-en-1-yl)malonate (0.13 g, 2.67 mmol), allyl bromide (0.12 mL, 1.34 mmol), Hoveyda-Grubbs 2nd generation catalyst (0.003 g, 0.02 mmol) added in two portions and CH$_2$Cl$_2$ (1.34 mL) to give the title compound as a colourless oil and as a mixture of (79:21) E/Z isomers (0.072 g, 1.29 mmol, 48%). Spectroscopic data for major (*E*) isomer: ^{1}H NMR (500 MHz, CDCl$_3$) δ 1.69 (p, *J* = 7.4 Hz, 2 H, CH$_2$C*H*$_2$CH$_2$C(O)), 1.75 – 1.91 (m, 4 H, C*H*$_2$CHC*H*$_2$), 2.07 (q, *J* = 6.7 Hz, 2 H, C*H*$_2$CH$_2$CH$_2$C(O)), 2.41 – 2.51 (m, 5 H, CH$_2$CH$_2$C*H*$_2$C(O), C(O)CH$_2$C*H*$_2$C$_{quat}$, CH$_2$C*H*CH$_2$), 2.68 (t, *J* = 7.5 Hz, 2 H, C(O)C*H*$_2$CH$_2$C$_{quat}$), 3.50 (t, *J* = 11.5 Hz, 2 H, C*H*$_2$O), 3.93 (d, *J* = 5.6 Hz, 2 H, C*H*$_2$Br), 4.09 (dd, *J* = 11.7, 4.1 Hz, 2 H, C*H*$_2$O), 5.50 – 5.80 (m, 2 H, C*H*=C*H*), 7.13 (d, *J* = 7.9 Hz, 4 H, ArC*H*), 7.25 – 7.31 (m, 2 H, ArC*H*), 7.42 (t, *J* = 7.7 Hz, 4 H, ArC*H*) ppm. ^{13}C NMR (125 MHz, CDCl$_3$) δ 22.8 (CH$_2$*C*H$_2$CH$_2$C(O)), 26.6 (C(O)CH$_2$*C*H$_2$C$_{quat}$), 28.9 (C*H*$_2$CHC*H*$_2$), 31.4 (C*H*$_2$CH$_2$CH$_2$C(O)), 33.3 (*C*H$_2$Br), 38.2 (C(O)*C*H$_2$CH$_2$C$_{quat}$), 40.9 (CH$_2$CH$_2$*C*H$_2$C(O)), 42.0 (CH$_2$*C*HCH$_2$), 60.7 (C(O)CH$_2$CH$_2$*C*$_{quat}$), 68.4 (*C*H$_2$O*C*H$_2$), 121.4 (Ar*C*H), 126.5 (Ar*C*H), 127.5 (*C*H=CH), 129.8 (Ar*C*H), 135.4 (CH=*C*H), 150.4 (Ar*C*), 169.0 (*C*(O)O), 208.9 (C(O)) ppm. IR ν_{max} (neat/cm^{-1}): 2969, 1743, 1366, 1200, 688, 616. HRMS calcd for $C_{29}H_{34}BrO_6$ [M + H]$^+$: 557.1533, found 557.1521.

Diphenyl (*E*)-2-isopropyl-2-(3-oxo-11-phenyl-9-((4-(trifluoromethyl)benzoyl)oxy)undec-7-en-1-yl)malonate 6i

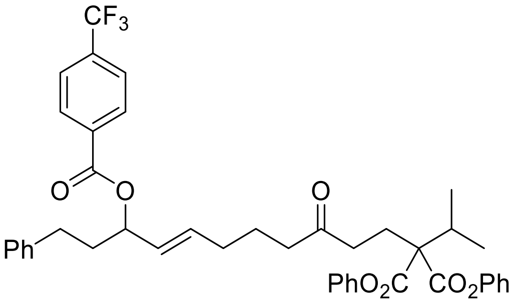

Prepared according to general procedure **G** using diphenyl 2-isopropyl-2-(3-oxooct-7-en-1-yl)malonate (16 mg, 0.38 mmol)**,** 5-phenylpent-1-en-3-yl 4-(trifluoromethyl)benzoate (390 mg, 1.15 mmol), Hoveyda-Grubbs 2nd generation catalyst (5 mg, 0.02 mmol) and CH_2Cl_2 (2 mL) to give the title compound as a colourless oil (170 mg, 0.24 mmol, 62%). ^{1}H NMR (400 MHz, $CDCl_3$) 1.21 (d, *J* = 6.9 Hz, 6 H, CH(C*H*$_3$)$_2$), 1.66 (p, *J* = 7.4 Hz, 2 H, CH$_2$C*H*$_2$CH$_2$C(O)), 1.95 – 2.18 (m, 4 H, C*H*$_2$CH$_2$CH$_2$C(O), O-CHC*H*$_2$CH$_2$), 2.36 – 2.45 (m, 4 H, C(O)CH$_2$C*H*$_2$C$_{quat}$, CH$_2$CH$_2$C*H*$_2$C(O)), 2.56 (p, *J* = 6.9 Hz, 1 H, C*H*(CH$_3$)$_2$), 2.62 – 2.73 (m, 4 H, C(O)C*H*$_2$CH$_2$C$_{quat}$, CHCH$_2$C*H*$_2$), 5.42 – 5.55 (m, 2 H, C*H*CH$_2$CH$_2$, CHCH=C*H*), 5.75 (dt, *J* = 14.9, 6.7 Hz, 1 H, CHC*H*=CH), 7.07 – 7.12 (m, 4 H, ArC*H*), 7.13 – 7.19 (m, 3 H, ArC*H*), 7.24 (qt, *J* = 6.9, 1.1 Hz, 4 H, ArC*H*), 7.33 – 7.41 (m, 4 H, ArC*H*), 7.66 (d, *J* = 8.2 Hz, 2 H, ArC*H*), 8.07 – 8.13 (m, 2 H, ArC*H*) ppm. ^{13}C NMR (100 MHz, $CDCl_3$) δ 18.7 (CH(*C*H$_3$)$_2$), 23.0 (CH$_2$*C*H$_2$CH$_2$C(O)), 27.0 (C(O)CH$_2$*C*H$_2$C$_{quat}$), 31.6 (C(O)*C*H$_2$CH$_2$C$_{quat}$), 31.7 (*C*H$_2$CH$_2$CH$_2$C(O)), 33.4 (*C*H(CH$_3$)$_2$, 36.2 (CH*C*H$_2$CH$_2$), 38.4 (CHCH$_2$*C*H$_2$), 42.1 (CH$_2$CH$_2$*C*H$_2$C(O)), 61.4 (C(O)CH$_2$CH$_2$*C*$_{quat}$), 75.7 (*C*HCH$_2$CH$_2$), 121.5 (Ar*C*H), 125.1 (*C*F$_3$), 125.4 (Ar*C*H), 126.1 (Ar*C*H), 126.3 (Ar*C*H), 128.4 (Ar*C*H), 128.6 (CH*C*H=CH), 128.7 (Ar*C*H), 129.7 (Ar*C*H), 130.1 (Ar*C*H), 133.9 (Ar*C*), 134.2 (CHCH=*C*H), 134.5 (Ar*C*), 141.2 (Ar*C*) 150.5 (Ar*C*), 164.7 (*C*(O)OAr), 169.4 (*C*(O)OPh), 209.2 (*C*(O)) ppm. IR ν_{max} (neat/cm^{-1}): 2941, 1745, 1716, 1592, 1411, 1324, 1271, 1184, 1162, 1113, 1065, 1017, 909, 86, 737, 688. HRMS calcd for $C_{43}H_{43}F_3O_7K$ [M + K]$^+$: 767.2592, found 767.2563.

Diphenyl (*E*)-2-(12-((*tert*-butyldimethylsilyl)oxy)-3-oxo-9-((4-(trifluoromethyl)benzoyl)oxy)dodec-7-en-1-yl)-2-isopropylmalonate 6j

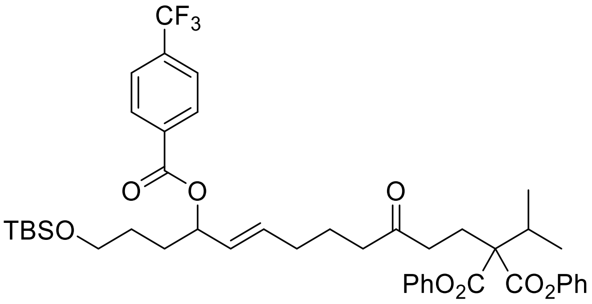

Prepared according to general procedure **G** using diphenyl 2-isopropyl-2-(3-oxooct-7-en-1-yl)malonate (100 mg, 0.24 mmol)**,** 6-((*tert*-butyldimethylsilyl)oxy)hex-1-en-3-yl 4-(trifluoromethyl)benzoate (290 mg, 0.71 mmol), Hoveyda-Grubbs 2nd generation catalyst (3 mg, 0.02 mmol) and CH_2Cl_2 (1.2 mL) to give the title compound as a colourless oil (58 mg, 0.07 mmol, 31%). ^{1}H NMR (400 MHz, $CDCl_3$) δ 0.04 (s, 6 H, Si(C*H*$_3$)$_2$), 0.88 (s, 9 H, SiC(C*H*$_3$)$_3$), 1.22 (d, *J* = 6.9 Hz, 6 H, (C*H*$_3$)$_2$), 1.54 – 1.71 (m, 4 H, OCH$_2$C*H*$_2$CH$_2$, CH$_2$C*H*$_2$CH$_2$C(O)), 1.71 – 1.88 (m, 2 H, OCH$_2$CH$_2$C*H*$_2$), 2.03 (q, *J* = 7.2 Hz, 2 H, C*H*$_2$CH$_2$CH$_2$C(O)), 2.37 – 2.44 (m, 4 H, CH$_2$CH$_2$C*H*$_2$C(O), C(O)CH$_2$C*H*$_2$C$_{quat}$), 2.57 (p, *J* = 6.9 Hz, 1 H, C*H*(CH$_3$)$_2$), 2.66 (dd, *J* = 8.9, 6.7 Hz, 2 H, C(O)C*H*$_2$CH$_2$C$_{quat}$), 3.63 (t, *J* = 6.3 Hz, 2 H, OC*H*$_2$CH$_2$CH$_2$), 5.42 – 5.54 (m, 2 H, C*H*CH=CH, CHC*H*=CH), 5.74 (dt, *J* = 14.1, 6.7 Hz, 1 H, CHCH=C*H*), 7.08 – 7.14 (m, 4 H, ArC*H*), 7.23 – 7.29 (m, 2 H, ArC*H*), 7.39 (dd, *J* = 8.4, 7.4 Hz, 4 H, ArC*H*), 7.68 (d, *J* = 8.2 Hz, 2 H, ArC*H*), 8.11 – 8.17 (m, 2 H, ArC*H*) ppm. ^{13}C NMR (100 MHz, $CDCl_3$) δ -5.1 (Si(*C*H$_3$)$_2$), 18.5 (Si*C*(CH$_3$)$_3$), 18.8 (CH(*C*H$_3$)$_2$), 23.0 (CH$_2$*C*H$_2$CH$_2$C(O)), 26.1 (SiC(*C*H$_3$)$_3$), 27.1 (C(O)CH$_2$*C*H$_2$C$_{quat}$), 28.6 (OCH$_2$*C*H$_2$CH$_2$), 31.2 (OCH$_2$CH$_2$*C*H$_2$), 31.7 (*C*H$_2$CH$_2$CH$_2$C(O)), 33.4 (*C*H(CH$_3$)$_2$), 38.4 (C(O)*C*H$_2$CH$_2$C$_{quat}$), 42.2 (CH$_2$CH$_2$*C*H$_2$C(O)), 61.4 (C(O)CH$_2$CH$_2$*C*$_{quat}$), 62.8 (O*C*H$_2$CH$_2$CH$_2$), 76.1 (*C*HCH=CH), 121.5 (Ar*C*H), 125.4 (*C*F$_3$), 125.5 (Ar*C*H), 125.5 (Ar*C*H), 126.4 (Ar*C*H), 128.9 (O-CHCH=*C*H), 129.7 (Ar*C*H), 130.1 (Ar*C*), 133.9 (CH*C*H=CH), 146.5 (Ar*C*), 150.5 (Ar*C*), 164.8 (*C*(O)OAr), 169.0 (*C*(O)OPh), 209.3 (*C*(O)) ppm *C*F$_3$ not observed. IR ν_{max} (neat/cm^{-1}): 2929, 1747, 1720, 1493, 1325, 1272, 1198, 1187, 1164, 1132, 1101, 1066, 2028, 835, 776, 688. HRMS calcd for $C_{44}H_{55}F_3O_8SiNa$ [M + Na]$^+$: 819.3511, found 819.3475.

Diphenyl (*E*)-2-benzyl-2-(9-bromo-3-oxonon-7-en-1-yl)malonate 6k

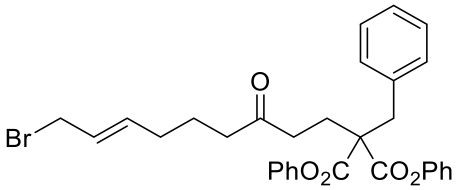

Prepared according to general procedure **G** using diphenyl 2-benzyl-2-(3-oxooct-7-en-1-yl)malonate (443 mg, 0.94 mmol), allyl bromide (406 µL, 4.70 mmol), Hoveyda-Grubbs 2nd generation catalyst (26 mg, 0.040 mmol; added in two portions) and CH_2Cl_2 (3 mL), to give the title compound as a colourless oil and as a mixture of (83:17) E/Z isomers (396 mg, 0.70 mmol, 75%). Spectroscopic data for major (*E*) isomer: ^{1}H NMR (400 MHz, $CDCl_3$) δ 1.69 (p, *J* = 7.4 Hz, 2 H, $CH_2CH_2CH_2C(O)$), 2.02 – 2.11 (m, 2 H, $CH_2CH_2CH_2C(O)$), 2.38 – 2.51 (m, 4 H, 2 H from $CH_2CH_2CH_2C(O)$, 2 H from $C(O)CH_2CH_2C$), 2.67 – 2.74 (m, 2 H, $C(O)CH_2CH_2C$), 3.53 (s, 2 H, CH_2Ar), 3.90 – 3.95 (m, 2 H, BrCH_2), 5.63 – 5.79 (m, 2 H, C*H*=C*H*), 7.06 – 7.12 (m, 4 H, ArC*H*), 7.24 – 7.37 (m, 7 H, ArC*H*), 7.39 – 7.46 (m, 4 H, ArC*H*) ppm. ^{13}C NMR (100 MHz, $CDCl_3$) δ 22.6 ($CH_2CH_2CH_2C(O)$), 26.9 ($C(O)CH_2CH_2C$), 31.3 ($CH_2CH_2CH_2C(O)$), 33.2 (BrCH_2), 37.9 ($C(O)CH_2CH_2C$), 39.7 (CH_2Ar), 41.9 ($CH_2CH_2CH_2C(O)$), 58.5 ($C(O)CH_2CH_2C$), 121.2 (Ar*C*H), 126.3 (Ar*C*H), 127.3 (BrCH_2*C*H=CH), 127.5 (Ar*C*H), 128.6 (Ar*C*H), 129.6 (Ar*C*H), 130.3 (Ar*C*H), 135.1 (Ar*C*), 135.3 (BrCH_2CH=*C*H), 150.4 (Ar*C*-O), 169.3 (*C*(O)O), 208.7 (*C*(O)) ppm. IR ν_{max} (neat/cm^{-1}): 2942, 2360, 1747, 1713, 1590, 1491, 1455, 1411, 1372, 1292, 1192, 1161, 1082, 1069, 1024, 1002, 965, 917, 831, 736, 701, 688. HRMS calcd for $C_{31}H_{31}BrO_5K$ [M + K]$^+$: 601.0986, found 601.0993.

Diphenyl (*E*)-2-(9-bromo-3-oxonon-7-en-1-yl)-2-(4-chlorobenzyl)malonate 6l

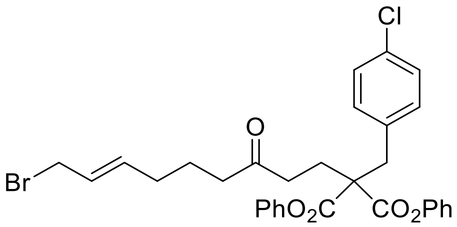

Prepared according to general procedure **G** using diphenyl 2-(4-chlorobenzyl)-2-(3-oxooct-7-en-1-yl)malonate (334 mg, 0.660 mmol), allyl bromide (285 µL, 3.3 mmol), Hoveyda-Grubbs 2nd generation catalyst (16 mg, 0.026 mmol; added in two portions) and CH_2Cl_2 (2 mL), to give the title compound as a colourless oil and as a mixture of (83:17) E/Z isomers (284 mg, 0.475 mmol, 73%). Spectroscopic data for major (*E*) isomer: ^{1}H NMR (400 MHz, $CDCl_3$) δ

1.67 (p, *J* = 7.4 Hz, 2 H, $CH_2CH_2CH_2C(O)$), 2.02 – 2.09 (m, 2 H, $CH_2CH_2CH_2C(O)$), 2.35 – 2.48 (m, 4 H, 2 H from $CH_2CH_2CH_2C(O)$, 2 H from $C(O)CH_2CH_2C_{quat}$), 2.65 – 2.71 (m, 2 H, $C(O)CH_2CH_2C_{quat}$), 3.46 (s, 2 H, CH_2Ar), 3.90 – 3.94 (m, 2 H, $BrCH_2$), 5.63 – 5.77 (m, 2 H, C*H*=C*H*), 7.04 – 7.10 (m, 4 H, Ar*C*H), 7.22 – 7.34 (m, 6 H, Ar*C*H), 7.37 – 7.44 (m, 4 H, Ar*C*H) ppm. ^{13}C NMR (100 MHz, $CDCl_3$) δ 22.8 ($CH_2CH_2CH_2C(O)$), 27.2 ($C(O)CH_2CH_2C_{quat}$), 31.4 ($CH_2CH_2CH_2C(O)$), 33.3 ($BrCH_2$), 37.9 ($C(O)CH_2CH_2C_{quat}$), 39.4 (CH_2Ar), 42.0 ($CH_2CH_2CH_2C(O)$), 58.6 ($C(O)CH_2CH_2C_{quat}$), 121.3 (Ar*C*H), 126.5 (Ar*C*H), 127.5 ($BrCH_2CH$=CH), 128.9 (Ar*C*H), 129.8 (Ar*C*H), 131.8 (Ar*C*H), 133.7 (Ar*C*), 133.8 (Ar*C*), 135.4 ($BrCH_2CH$=*C*H), 150.5 (Ar*C*-O), 169.3 (*C*(O)O), 208.7 (*C*(O)) ppm. IR ν_{max} (neat/cm^{-1}): 2933, 1746, 1714, 1591, 1492, 1456, 1409, 1373, 1192, 1161, 1094, 1069, 1024, 1016, 965, 914, 847, 831, 743, 715, 686, 668, 657. HRMS calcd for $C_{31}H_{30}BrClO_5K$ $[M+K]^+$: 635.0602, found 635.0602.

Diphenyl (*E*)-2-(9-bromo-3-oxonon-7-en-1-yl)-2-(4-fluorobenzyl)malonate 6m

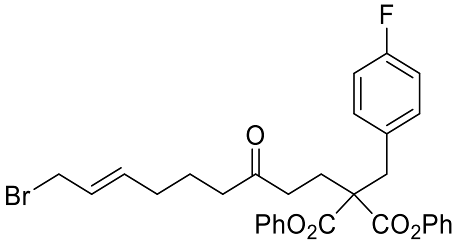

Prepared according to general procedure **G** using diphenyl 2-(4-fluorobenzyl)-2-(3-oxooct-7-en-1-yl)malonate (395 mg, 0.810 mmol), allyl bromide (345 µL, 4.05 mmol), Hoveyda-Grubbs 2nd generation catalyst (20 mg, 0.032 mmol; added in two portions) and CH_2Cl_2 (2 mL) to give the title compound as a colourless oil and as a mixture of (83:17) E/Z isomers (329 mg, 0.566 mmol, 70%). Spectroscopic data for major (*E*) isomer: ^1H NMR (400 MHz, $CDCl_3$) δ 1.67 (p, *J* = 7.3 Hz, 2 H, $CH_2CH_2CH_2C(O)$), 2.02 – 2.08 (m, 2 H, $CH_2CH_2CH_2C(O)$), 2.35 – 2.49 (m, 4 H, 2 H from $CH_2CH_2CH_2C(O)$, 2 H from $C(O)CH_2CH_2C_{quat}$), 2.65 – 2.72 (m, 2 H, $C(O)CH_2CH_2C_{quat}$), 3.47 (s, 2 H, CH_2Ar), 3.89 – 3.94 (m, 2 H, $BrCH_2$), 5.63 – 5.79 (m, 2 H, C*H*=C*H*), 6.99 – 7.10 (m, 6 H, Ar*C*H), 7.25 – 7.31 (m, 4 H, Ar*C*H), 7.37 – 7.44 (m, 4 H, Ar*C*H) ppm. ^{13}C NMR (100 MHz, $CDCl_3$) δ 22.8 ($CH_2CH_2CH_2C(O)$), 27.2 ($C(O)CH_2CH_2C_{quat}$), 31.4 ($CH_2CH_2CH_2C(O)$), 33.3 ($BrCH_2$), 38.0 ($C(O)CH_2CH_2C_{quat}$), 39.2 (CH_2Ar), 42.0 ($CH_2CH_2CH_2C(O)$), 58.7 ($C(O)CH_2CH_2C_{quat}$), 115.6 (d, *J* = 21.2 Hz, Ar*C*H), 121.3 (Ar*C*H), 126.5 (Ar*C*H), 127.5 ($BrCH_2CH$=CH), 129.8 (Ar*C*H), 131.0 (d, *J* = 3.5 Hz, Ar*C*), 132.0 (d, *J* = 8.0 Hz, Ar*C*H), 135.4 ($BrCH_2CH$=*C*H), 150.5 (Ar*C*-O), 162.4 (d, *J* = 246.2 Hz, Ar*C*), 169.3 (*C*(O)O), 208.7 (*C*(O)) ppm. ^{19}F NMR (375 MHz, $CDCl_3$) δ -114.92 ppm. IR ν_{max} (neat/cm^{-1}):

2934, 1746, 1715, 1590, 1510, 1492, 1456, 1418, 1372, 1222, 1186, 1159, 1098, 1069, 1024, 1000, 966, 915, 842, 745, 688, 668, 658, 602. HRMS calcd for $C_{31}H_{30}BrFO_5K$ $[M+K]^+$: 619.0897, found 619.0897.

Diphenyl (*E*)-2-(9-bromo-3-oxonon-7-en-1-yl)-2-(4-methoxybenzyl)malonate 6n

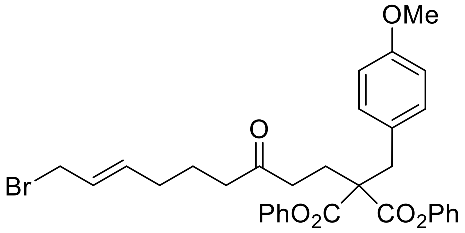

Prepared according to general procedure **G** using diphenyl 2-(4-methoxybenzyl)-2-(3-oxooct-7-en-1-yl)malonate (0.26g, 0.52 mmol), allyl bromide (0.23 mL, 2.62 mmol), Hoveyda-Grubbs 2nd generation catalyst (6.6 mg, 0.01 mmol; added in two portions) and CH_2Cl_2 (2 mL), to give the title compound as a colourless oil and as a mixture of (83:17) *E*/*Z* isomers (0.17 g, 0.28 mmol, 54%). ^{1}H NMR (500 MHz, $CDCl_3$) δ 1.60 (p, *J* = 7.2 Hz, 2 H, $CH_2CH_2CH_2C(O)$), 1.98 (q, *J* = 6.7 Hz, 2 H, $CH_2CH_2CH_2C(O)$), 2.26 – 2.40 (m, 4 H, 2 H from $CH_2CH_2CH_2C(O)$, 2 H from $C(O)CH_2CH_2C$), 2.60 (t, *J* = 7.6 Hz, 2 H, $C(O)CH_2CH_2C$), 3.38 (s, 2 H, CH_2Ar), 3.73 (s, 3 H, $ArOCH_3$), 3.86 (m, 2 H, $BrCH_2$), 5.41 – 5.71 (m, 2 H, C*H*=C*H*), 6.79 (d, *J* = 8.1 Hz, 2 H, ArC*H*), 7.02 (d, *J* = 7.9 Hz, 4H, ArC*H*), 7.14 (d, *J* = 8.2 Hz, 2 H, ArC*H*), 7.17 – 7.22 (m, 2 H, ArC*H*), 7.33 (t, *J* = 7.7 Hz, 4 H, ArC*H*). ^{13}C NMR (126 MHz, $CDCl_3$) δ 22.8 ($CH_2CH_2CH_2C(O)$), 26.9 ($C(O)CH_2CH_2C$), 31.4 ($CH_2CH_2CH_2C(O)$), 33.3 ($BrCH_2$), 38.0 ($C(O)CH_2CH_2C$), 39.0 (CH_2Ar), 42.0 ($CH_2CH_2CH_2C(O)$), 55.4 ($ArOCH_3$), 58.7 ($C(O)CH_2CH_2C$), 114.1 (Ar*C*H), 121.4 (Ar*C*H), 126.4 (Ar*C*H), 127.1 ($BrCH_2CH$=CH), 127.4 (Ar*C*), 129.8 (Ar*C*H), 131.4 (Ar*C*H), 135.4 ($BrCH_2CH$=*C*H), 150.6 (Ar*C*-O), 159.1 (Ar*C*-OCH_3), 169.5 (*C*(O)O), 208.9 (*C*(O)). IR ν_{max} (neat/cm^{-1}): 2936, 1746, 1714, 1513, 1492, 1248, 1160, 1032, 909, 837, 606. HRMS calcd for $C_{32}H_{34}O_6Br$ $[M + H]^+$: 593.1533, found 593.1531.

Diphenyl (*E*)-2-(9-bromo-3-oxonon-7-en-1-yl)-2-(3,5-dimethylbenzyl)malonate 6o

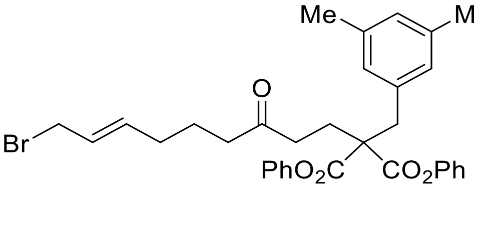

Prepared according to general procedure **G** using diphenyl 2-(3,5-dimethylbenzyl)-2-(3-oxooct-7-en-1-yl)malonate (416 mg, 0.830 mmol), allyl bromide (360 µL, 4.17 mmol),

Hoveyda-Grubbs 2nd generation catalyst (20 mg, 0.032 mmol; added in two portions) and CH_2Cl_2 (2 mL), to give the title compound as a colourless oil and as a mixture of (83:17) E/Z isomers (302 mg, 0.51 mmol, 61%). Spectroscopic data for major (*E*) isomer: ^{1}H NMR (400 MHz, $CDCl_3$) δ 1.67 (p, *J* = 7.3 Hz, 2 H, $CH_2CH_2CH_2C(O)$), 2.01 – 2.09 (m, 2 H, $CH_2CH_2CH_2C(O)$), 2.30 (s, 6 H, 2 × ArC-CH_3), 2.35– 2.49 (m, 4 H, 2 H from $CH_2CH_2CH_2C(O)$, 2 H from $C(O)CH_2CH_2C_{quat}$), 2.65 – 2.72 (m, 2 H, $C(O)CH_2CH_2C_{quat}$), 3.43 (s, 2 H, CH_2Ar), 3.89 – 3.94 (m, 2 H, $BrCH_2$), 5.63 – 5.79 (m, 2 H, C*H*=C*H*), 6.90 (s, 2 H, ArC*H*), 6.93 (s, 1 H, ArC*H*), 7.07 – 7.11 (m, 4 H, ArC*H*), 7.24 – 7.29 (m, 2 H, ArC*H*), 7.37 – 7.43 (m, 4 H, ArC*H*) ppm.^{13}C NMR (100 MHz, $CDCl_3$) δ 21.5 (ArC-CH_3), 22.8 ($CH_2CH_2CH_2C(O)$), 26.9 ($C(O)CH_2CH_2C_{quat}$), 31.4 ($CH_2CH_2CH_2C(O)$), 33.3 ($BrCH_2$), 38.1 ($C(O)CH_2CH_2C_{quat}$), 39.6 (CH_2Ar), 42.0 ($CH_2CH_2CH_2C(O)$), 58.6 ($C(O)CH_2CH_2C_{quat}$), 121.4 (Ar*C*H), 126.4 (Ar*C*H), 127.5 ($BrCH_2$*C*H=CH), 128.3 (Ar*C*H), 129.3 (Ar*C*H), 129.7 (Ar*C*H), 135.0 (Ar*C*), 135.4 ($BrCH_2$CH=*C*H), 138.1 (Ar*C*), 150.6 (Ar*C*-O), 169.5 (*C*(O)O), 208.9 (*C*(O)) ppm. IR ν_{max} (neat/cm^{-1}): 2933, 1750, 1716, 1591, 1492, 1456, 1375, 1195, 1162, 1069, 1026, 966, 853, 745, 688, 668, 657. HRMS calcd for $C_{33}H_{35}BrO_5K$ $[M+K]^+$: 629.1305, found 629.1303.

Diphenyl (*E*)-2-(9-bromo-3-oxonon-7-en-1-yl)-2-(thiophen-3-ylmethyl)malonate 6p

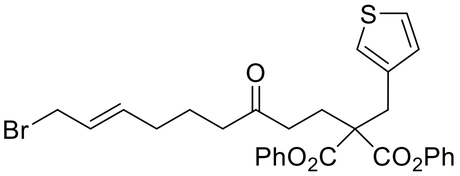

Prepared according to general procedure **G** using diphenyl 2-(3-oxooct-7-en-1-yl)-2-(thiophen-3-ylmethyl)malonate (0.28 g, 0.59 mmol), allyl bromide (0.26 mL, 3.00 mmol), Hoveyda-Grubbs 2nd generation catalyst (7.4 mg, 0.01 mmol; added in two portions) and CH_2Cl_2 (2 mL), to give the title compound as a colourless oil and as a mixture of (83:17) *E*/*Z* isomers (0.19 mg, 0.33 mmol, 56%). ^{1}H NMR (400 MHz, $CDCl_3$) δ 1.65 (p, *J* = 7.4 Hz, 2 H, $CH_2CH_2CH_2C(O)$), 2.03 (tdd, *J* = 6.4, 4.9, 2.1 Hz, 2 H, $CH_2CH_2CH_2C(O)$), 2.40 (td, *J* = 7.2, 3.3 Hz, 4 H, 2 H from $CH_2CH_2CH_2C(O)$, 2 H from $C(O)CH_2CH_2C$), 2.65 (dd, *J* = 8.7, 6.7 Hz, 2 H, $C(O)CH_2CH_2C$), 3.50 (s, 2 H, CH_2Ar), 3.89 (d, *J* = 6.0 Hz, 2 H, $BrCH_2$), 5.44 – 5.81 (m, 2 H, C*H*=C*H*), 7.01 (dd, *J* = 4.9, 1.4 Hz, 1 H, ArC*H*), 7.03 – 7.09 (m, 4 H, ArC*H*), 7.14 (dd, *J* = 3.0, 1.3 Hz, 1 H, ArC*H*), 7.23 (t, *J* = 3.7 Hz, 2 H, ArC*H*), 7.28 (dd, *J* = 4.9, 2.9 Hz, 1 H, ArC*H*), 7.38 (dd, *J* = 8.7, 7.1 Hz, 4 H, ArC*H*). ^{13}C NMR (101 MHz, $CDCl_3$) δ 22.7 ($CH_2CH_2CH_2C(O)$), 27.2 ($C(O)CH_2CH_2C$), 31.4 ($CH_2CH_2CH_2C(O)$), 33.3 ($BrCH_2$), 34.4 (CH_2Ar), 37.9

(C(O)*C*$H_2$$CH_2$C), 41.9 ($CH_2$$CH_2$*C*$H_2$C(O)), 58.3 (C(O)$CH_2$$CH_2$*C*), 121.3 (Ar*C*H), 124.3 (Ar*C*H), 126.0 (Ar*C*H), 126.5 (Ar*C*H), 127.5 (BrCH_2*C*H=CH), 129.3 (Ar*C*H), 129.8 (Ar*C*H), 135.3 (Ar*C*), 135.4 (BrCH_2CH=*C*H), 150.5 (Ar*C*-O), 169.5 (*C*(O)O), 208.8 (*C*(O)). IR ν_{max} (neat/cm^{-1}): 2935, 1748, 1715, 1591, 1492, 1194, 1162, 905, 680, 688, 648. HRMS calcd for $C_{29}H_{29}O_5BrSNa$ [M + Na]$^+$: 591.0811, found 591.0813.

Diphenyl-(*E*)-2-benzyl-2-(12-((*tert*-butyldimethylsilyl)oxy)-3-oxo-9-((4-(trifluoromethyl) benzoyl)oxy)dodec-7-en-1-yl)malonate 6q

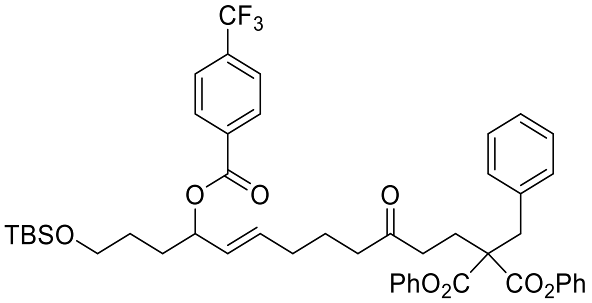

Prepared according to general procedure **G** using diphenyl 2-benzyl-2-(3-oxooct-7-en-1-yl)malonate (211 mg, 0.45 mmol)**,** 6-((*tert*-butyldimethylsilyl)oxy)hex-1-en-3-yl 4-(trifluoromethyl)benzoate (543 mg, 1.35 mmol), Hoveyda-Grubbs 2nd generation catalyst (8 mg, 0.013 mmol) and CH_2Cl_2 (1 mL) to give the title compound a colourless oil (183 mg, 0.22 mmol, 48%). ^{1}H NMR (400 MHz, $CDCl_3$) δ 0.03 (s, 6 H, 2 × Si-C*H_3*), 0.88 (s, 9 H, 3 × Si-C(C*H_3*)), 1.52 – 1.62 (m, 2 H, OCH_2C*H_2*CH_2), 1.66 (p, *J* = 7.3 Hz, 2 H, CH_2C*H_2*CH_2C(O)), 1.71 – 1.89 (m, 2 H, O$CH_2$$CH_2$C*$H_2$*), 1.99 – 2.07 (m, 2 H, C*H_2*$CH_2$$CH_2$C(O)), 2.35 – 2.44 (m, 4 H, $CH_2$$CH_2$C*$H_2$*C(O), C(O)$CH_2$C*$H_2$*$C_{quat}$), 2.63 – 2.69 (m, 2 H, C(O)C*H_2*$CH_2$$C_{quat}$), 3.49 (s, 2 H, C*$H_2$*Ph), 3.63 (t, *J* = 6.5 Hz, 2 H, OC*H_2*$CH_2$$CH_2$), 5.43 – 5.53 (m, 2 H, C*H*CH=CH, 1 H, CHC*H*=CH), 5.70 – 5.78 (m, 1 H, CHCH=C*H*), 7.07 (d, *J* = 7.8 Hz, 4 H, ArC*H*), 7.22 – 7.36 (m, 7 H, ArC*H*), 7.39 (t, *J* = 7.8 Hz, 4 H, ArC*H*), 7.68 (d, *J* = 7.9 Hz, 2 H, ArC*H*), 8.14 (d, *J* = 7.9 Hz, 2 H, ArC*H*) ppm. ^{13}C NMR (100 MHz, $CDCl_3$) δ -5.2 (Si-*C*H_3), 18.5 (Si-*C*(CH_3)$_3$), 23.0 (CH_2*C*$H_2$$CH_2$C(O)), 26.1 (Si-C(*C*$H_3$)$_3$), 27.0 (C(O)$CH_2$*C*$H_2$$C_{quat}$), 28.6 (O$CH_2$*C*$H_2$$CH_2$), 31.2 (O$CH_2$$CH_2$*C*$H_2$), 31.6 (*C*$H_2$$CH_2$$CH_2$C(O)), 37.9 (C(O)*C*$H_2$$CH_2$$C_{quat}$), 39.8 (*C*$H_2$Ph), 42.2 ($CH_2$$CH_2$*C*$H_2$C(O)), 58.6 (C(O)$CH_2$$CH_2$*C*$_{quat}$), 62.8 (O*C*$H_2$$CH_2$$CH_2$), 76.1 (*C*HCH=CH), 121.4 (Ar*C*H), 123.8 (d, *J* = 272.6 Hz, Ar-*C*F_3), 125.5 (q, *J* = 3.6 Hz, Ar*C*H), 126.4 (Ar*C*H), 127.6 (Ar*C*H), 128.8 (Ar*C*H), 129.0 (CH*C*H=CH), 129.8 (Ar*C*H), 130.1 (Ar*C*H), 130.4 (Ar*C*H), 133.9 (CHCH=*C*H), 134.1 (Ar*C*), 134.4 (q, *J* = 32.7 Hz, Ar*C*), 135.2 (Ar*C*), 150.6 (Ar*C*), 164.8 (*C*(O)OAr), 169.4 (*C*(O)OPh), 208.9 (*C*(O)) ppm. IR ν_{max} (neat/cm^{-1}): 2929,

1750, 1719, 1493, 1325, 1272, 1165, 1132, 1100, 1065, 1017, 835, 776, 737, 703, 688, 668, 657. HRMS calcd for $C_{48}H_{55}F_3O_8SiNa$ $[M+Na]^+$: 867.3516, found 867.3510.

Diphenyl (*E*)-2-(9-bromo-3-oxonon-7-en-1-yl)-2-(propan-2-yl-2-*d*)malonate *D*-6e

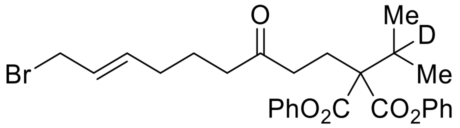

Prepared according to general procedure **G** using diphenyl 2-(3-oxooct-7-en-1-yl)-2-(propan-2-yl-2-d)malonate (310 mg, 0.73 mmol), allyl bromide (323 µL, 3.66 mmol), Hoveyda-Grubbs 2nd generation catalyst (26 mg, 0.040 mmol; added in two portions) and CH_2Cl_2 (3 mL), to give the title compound as a colourless oil and as a mixture of (83:17) E/Z isomers (278 mg, 0.54 mmol, 74%). Spectroscopic data for major (*E*) isomer: ^{1}H NMR (400 MHz, $CDCl_3$) δ 1.23 (s, 6 H, CD(C*H*$_3$)$_2$), 1.68 (p, *J* = 7.4 Hz, 2 H, $CH_2CH_2CH_2$C(O)) with the middle *H*, 2.03 – 2.10 (m, 2 H, C*H*$_2$CH$_2$CH$_2$C(O)), 2.38 – 2.50 (m, 4 H, 2 H from CH$_2$CH$_2$C*H*$_2$C(O), 2 H from C(O)CH$_2$C*H*$_2$C), 2.66 – 2.73 (m, 2 H, C(O)C*H*$_2$CH$_2$C), 3.92 – 3.95 (m, 2 H, C*H*$_2$Br), 5.65 – 5.74 (m, 2 H, C*H*=C*H*), 7.09 – 7.16 (m, 4 H, ArC*H*), 7.25 – 7.31 (m, 2 H, ArC*H*), 7.37 – 7.46 (m, 4 H, ArC*H*) ppm. ^{13}C NMR (100 MHz, $CDCl_3$) δ 18.6 (CD(*C*H$_3$)$_2$), 22.7 (CH$_2$*C*H$_2$CH$_2$C(O)), 26.9 (C(O)CH$_2$*C*H$_2$C), 31.3 (*C*H$_2$CH$_2$CH$_2$C(O)), 33.2 (*C*H$_2$Br), 33.4 (1:1:1 t, *J* = 20.1 Hz, *C*D(CH$_3$)$_2$), 38.4 (C(O)*C*H$_2$CH$_2$C), 41.9 (CH$_2$CH$_2$*C*H$_2$C(O)), 61.3 (C(O)CH$_2$CH$_2$*C*), 121.4 (Ar*C*H), 126.3 (Ar*C*H), 127.3 (BrCH$_2$*C*H=CH), 129.6 (Ar*C*H), 135.4 (BrCH$_2$CH=*C*H), 150.4 (Ar*C*), 169.4 (*C*(O)O), 209.1 (*C*(O)) ppm. IR ν_{max} (neat/cm^{-1}): 2940, 1742, 1713, 1591, 1491, 1456, 1392, 1372, 1292, 1225, 1183, 1160, 1069, 1023, 999, 965, 917, 831, 741, 687. HRMS calcd for $C_{27}H_{30}DBrO_5K$ $[M + K]^+$: 554.1049, found 554.1055.

Preparation of SmI_2

An oven-dried (140 ℃) round bottom flask, equipped with a stirrer bar, was cooled down under a positive strong flow of nitrogen for 30 minutes and loaded with samarium metal (-40 mesh, 1.4 equiv.) and washed (sodium thiosulfate) diiodoethane (1 equiv.). The flask was flushed for another 30 minutes under a positive flow of nitrogen, after which, freshly distilled THF (0.1 M) was added. The mixture was then stirred vigorously under a positive pressure of nitrogen for 16 hours at room temperature. The solid residues were allowed to settle for one hour and the mixture was titrated before use.[6]

[6] Szostak, M.; Spain, M.; Procter, D. J. *Nat. Protoc.* **2012**, *7* (5), 970–977.

***rac*-(3a*R*,7*R*,8*S*,8a*S*)-8-Methyloctahydroazulene-3a,7(1H)-diol 7a'**

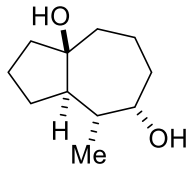

General procedure **H**. To a solution of SmI_2 (9.00 mL, 0.90 mmol, 0.1 M in THF), under nitrogen, ethyl (*E*)-11-bromo-5-oxoundec-9-enoate (34.0 mg, 0.11 mmol) in THF (0.70 mL) was added dropwise and the reaction mixture stirred for 14 h at room temperature. After that time, degassed H_2O (2.20 mL, 122 mmol) was added and reaction was continued at the same temperature for 24 h before being quenched with air, followed by saturated aqueous Rochelle's salt (10 mL) and a few drops of saturated aqueous sodium thiosulfate. The aqueous layer was extracted with Et_2O (3 × 15 mL) and the combined organic layers were washed with brine (15 mL), dried over $MgSO_4$, concentrated *in vacuo* and purified by column chromatography eluting with EtOAc/Hexane (5:95), to give title compound as a white solid (12 mg, 0.065 mmol, 59%). ^{1}H NMR (400 MHz, $CDCl_3$) δ 1.00 (d, *J* = 6.8 Hz, 3 H, $CHCH_3$), 1.05 (bs, 1 H, CO*H*), 1.39 (bs, 1 H, CHO*H*), 1.44–1.82 (m, 10 H, $CHCH_2CH_2CH_2COH$ + $CH(OH)CH_2CH_2CH_2COH$ + $CH(OH)CH_aH_bCH_2CH_2COH$ + $CHCH_aH_bCH_2CH_2COH$ + $CHCH_2CH_2CH_aH_bCOH$ + $CH(OH)CH_2CH_2CH_2COH$ + C*H*COH), 1.84–2.02 (m, 4 H, $CH(OH)CH_aH_bCH_2CH_2COH$ + $CHCH_aH_bCH_2CH_2COH$ + $CHCH_3$ + $CHCH_2CH_2CH_aH_bCOH$), 4.00–4.08 (m, 1 H, C*H*OH) ppm. ^{13}C NMR (100 MHz, $CDCl_3$) δ 16.0 ($CHCH_3$), 20.3 ($CHCH_2CH_2CH_2COH$), 20.7 ($CH(OH)CH_2CH_2CH_2COH$), 30.8 ($CH(OH)CH_2CH_2CH_2COH$), 33.9 ($CHCH_2CH_2CH_2COH$), 37.5 ($CHCH_3$), 40.6 ($CHCH_2CH_2CH_2COH$), 43.8 ($CH(OH)CH_2CH_2CH_2COH$), 48.9 (*C*HCOH), 74.4 (*C*HOH), 82.1 (*C*OH) ppm. M.p. (CH_2Cl_2) 118–121 ºC. IR ν_{max} (neat/cm^{-1}): 3329 (O-H), 2934, 1035. HRMS calcd for $C_{11}H_{19}O$ $[M–OH]^+$: 167.1430, found 167.1430.

(3a*S*,7*S*,8*R*,8a*R*)-8-(4-((*tert*-Butyldimethylsilyl)oxy)butyl)octahydroazulene-3a,7(1H)-diol 7b'

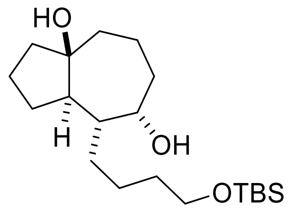

Prepared according to general procedure **H** using SmI_2 (10.0 mL, 0.1 mmol, 0.1 M in THF), (*E*)-1-((*tert*-butyldimethylsilyl)oxy)-10,14-dioxo-14-phenoxytetradec-5-en-4-yl 4-

(trifluoromethyl)benzoate (45 mg, 0.07 mmol) and H_2O (1.8 mL, 100 mmol) to give the title compound as a colourless oil (14.1 mg, 0.039 mmol, 55%). ^{1}H NMR (400 MHz, $CDCl_3$) δ 0.05 (s, 6 H, $Si(CH_3)_2$), 0.89 (s, 9 H, $SiC(CH_3)_3$), 1.21 – 1.41 (m, 6 H, $OCH_2CH_2CH_2CH_2CH$, $OCH_2CH_2CH_2CH_2CH$, $CHCH_2CH_2CH_2COH$), 1.42 – 1.64 (m, 10 H, 1 H from $CHCH_2CH_aH_bCH_2COH$ + $CH(OH)CH_2CH_2CH_2COH$ + 1 H from $CH(OH)CH_aH_bCH_2CH_2COH$ + 1 H from $CHCH_2CH_2CH_aH_bCOH$ + $CH(OH)CH_2CH_2CH_2COH$ + $OCH_2CH_2CH_2CH_2CH$, $OCH_2CH_2CH_2CH_2CH$), 1.65 – 1.77 (m, 4 H, 1 H from $CHCH_2CH_aH_bCH_2COH$, 1 H from $CH(OH)CH_aH_bCH_2CH_2COH$, 1 H from $CHCH_2CH_2CH_aH_bCOH$, CHC-OH), 1.91 – 2.08 (m, 2 H, COH, CHOH), 3.62 (t, J = 6.3 Hz, 2 H, $OCH_2CH_2CH_2CH_2CH$), 4.08 – 4.15 (m, 1 H, CHOH) ppm. ^{13}C NMR (100 MHz, $CDCl_3$) δ -5.1 ($Si(CH_3)_2$), 18.5 ($CHCH_2CH_2CH_2COH$), 19.3 ($CH(OH)CH_2CH_2CH_2COH$), 20.7 ($CHCH_2CH_2CH_2COH$), 24.3 ($OCH_2CH_2CH_2CH_2CH$), 26.1 ($SiC(CH_3)_3$), 30.1 ($SiC(CH_3)_3$), 30.2 ($OCH_2CH_2CH_2CH_2CH$), 33.3 ($OCH_2CH_2CH_2CH_2CH$), 34.8 ($CH(OH)CH_2CH_2CH_2COH$), 40.5 ($OCH_2CH_2CH_2CH_2CH$), 41.8 ($CHCH_2CH_2CH_2COH$), 43.5 ($CH(OH)CH_2CH_2CH_2COH$), 47.7 ($CHCH_2CH_2CH_2COH$), 63.2 ($OCH_2CH_2CH_2CH_2CH$), 70.9 (CH(OH)), 82.1 ($CHCH_2CH_2CH_2COH$) ppm. IR ν_{max} (neat/cm^{-1}): 3432, 2928, 2857, 1738, 1462, 1386, 1361, 1254, 1098, 908, 834, 774, 733, 662. HRMS calcd for $C_{20}H_{40}O_3SiNa$ [M + Na]$^+$: 379.2626, found 379.2634.

(3a*S*,7*S*,8*R*,8a*R*)-8-(3-Phenylpropyl)octahydroazulene-3a,7(1H)-diol 7c'

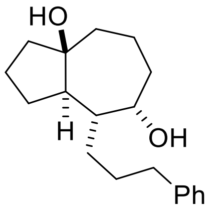

Prepared according to general procedure **H** using SmI_2 (14.0 mL, 1.4 mmol, 0.1 M in THF), (*E*)-9,13-dioxo-13-phenoxy-1-phenyltridec-4-en-3-yl 4-(trifluoromethyl)benzoate (57.0 mg, 0.1 mmol) and H_2O (2.52 mL, 140 mmol) to give the title compound as a colourless oil (12.7 mg, 0.044 mmol, 44%). ^{1}H NMR (400 MHz, $CDCl_3$) δ 1.42 (bs, 2 H, COH, CHOH), 1.46 – 2.08 (m, 15 H, $CHCH_2CH_2CH_2COH$, $CH_2CH_2CH_2Ph$, $CH_2CH_2CH_2Ph$, $CH(OH)CH_2CH_2CH_2OH$, $CH(OH)CH_2CH_2CH_2COH$, CHCOH, $CHCH_2CH_2CH_2Ph$, 1 H from $CH(OH)CH_aH_bCH_2CH_2COH$, 1 H from $CH_aCH_bCH_2CH_2COH$, 1 H from $CHCH_2CH_2CH_aCH_bCOH$), 2.18 – 2.74 (m, 5 H, 1 H from $CH(OH)CH_aH_bCH_2CH_2COH$, 1 H from $CHCH_aH_bCH_2CH_2COH$, 1 H from $CHCH_2CH_2CH_aH_bCOH$, $CH_2CH_2CH_2Ph$), 4.12 (ddd,

J = 7.8, 3.5, 2.9 Hz, 1 H, C*H*OH), 7.11 (ddd, J = 9.7, 5.9, 3.7 Hz, 2 H, ArC*H*), 7.17 – 7.23 (m, 3 H, ArC*H*). ppm. ^{13}C NMR (100 MHz, $CDCl_3$) δ 19.3 ($CHCH_2$*C*H_2CH_2COH), 20.7 ($CH(OH)CH_2$*C*H_2CH_2COH), 22.8 (*C*$H_2CH_2CH_2Ph$), 29.9 (CH_2*C*H_2CH_2Ph), 30.2 ($CH(OH)$*C*$H_2CH_2CH_2CH_2COH$), 34.9 (CH*C*$H_2CH_2CH_2CH_2COH$), 36.6 (*C*$HCH_2CH_2CH_2Ph$), 40.5 ($CH(OH)CH_2CH_2$*C*H_2COH), 41.8 ($CHCH_2CH_2$*C*H_2Ph), 43.6 ($CHCH_2CH_2$*C*H_2COH), 47.7 (*C*HCOH), 71.0 (*C*HOH), 82.1 (*C*OH), 125.8 (Ar*C*H), 128.4 (Ar*C*H), 128.5 (Ar*C*H), 142.8 (Ar*C*) ppm. IR ν_{max} (neat/cm^{-1}): 3452, 2925, 1738, 1454, 1365, 1217, 908, 668, 615. HRMS calcd for $C_{19}H_{28}O_2Na$ [M + Na]$^+$: 311.1982, found 311.1973.

(6a'*R*,9a'*S*,9b'*S*)-1',5',6',7',8',9',9a',9b'-Octahydro-3H-spiro[benzofuran-2,3'-cyclopenta[e]azulene]-3a',6a'(2'H,4'H)-diol S45 and 7d'

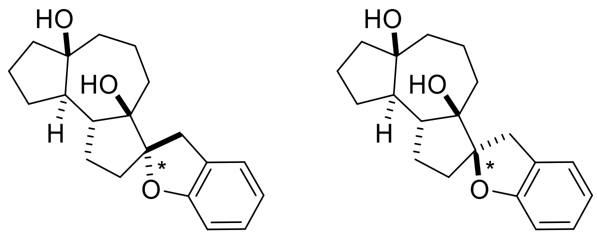

S45 **7d'**

Prepared according to general procedure **H** using SmI_2 (3.54 mL, 0.35 mmol, 0.1 M in THF), diphenyl (*E*)-1-(benzofuran-2-yl)-8,12-dioxo-12-phenoxydodec-3-en-2-yl 4-(trifluoromethyl)benzoate (15.0 mg, 0.026 mmol) and H_2O (0.63 mL, 35.4 mmol) to give **7d'** as a yellow solid (2.5 mg, 0.008 mmol, 32%) and **S45** as colourless oil (3.3 mg, 0.010 mmol, 41%).

Spectroscopic data for major diastereoisomer **S45**: ^{1}H NMR (500 MHz, $CDCl_3$) δ 1.37 – 1.69 (m, 7 H, 1 H from $CHCH_2C$*H_a*H_bCH_2C-OH, 1 H from CH_2C*H_a*$H_bCH_2C_{quat}$-OHC_{quat}-OAr, 1H from CHC*H_a*$H_bCH_2CH_2C$-OH, 1 H from C*H_a*$H_bCH_2C_{quat}$-OAr, 1 H from CH_2CH_2C*H_a*H_bC_{quat}-OHC_{quat}-OAr, 1 H from CH_2C*H_a*H_bC_{quat}-OAr, 1 H from $CHCH_2CH_2C$*H_a*H_bC-OH), 1.71 – 2.10 (m, 11 H, 1 H from $CHCH_2CH_a$*H_b*CH_2C-OH, 1 H from CH_2CH_a*H_b*CH_2C_{quat}-OHC_{quat}-OAr, 1H from $CHCH_a$*H_b*CH_2CH_2C-OH, 1 H from CH_a*H_b*CH_2C_{quat}-OAr, 1 H from $CH_2CH_2CH_a$*H_b*C_{quat}-OHC_{quat}-OAr, 1 H from CH_2CH_a*H_b*C_{quat}-OAr, 1 H from $CHCH_2CH_2CH_a$*H_b*C-OH, C*H_2*$CH_2CH_2C_{quat}$-OHC_{quat}-OAr, C*H*$CH_2CH_2C_{quat}$-OAr, C*H*$CH_2CH_2CH_2C$-OH), 2.92 (d, J = 16.5 Hz, 1 H, 1 H from C*H_a*H_b-Ar), 3.15 (s, 1 H, CHC-O*H*), 3.42 – 3.50 (m, 1 H, CH_a*H_b*-Ar), 3.65 (s, 1 H, C_{quat}-O*H*), 6.70 (d, J = 7.9 Hz, 1 H, ArC*H*), 6.80 (td, J = 7.4, 1.0 Hz, 1 H, ArC*H*), 7.04 – 7.10 (m, 1 H, ArC*H*), 7.14 (dd, J = 7.4,

1.3 Hz, 1 H, ArC*H*) ppm. ^{13}C NMR (125 MHz, $CDCl_3$) δ 17.5 ($CHCH_2$*C*H_2CH_2C-OH), 22.3 (CH_2*C*$H_2CH_2C_{quat}$-OHC_{quat}-OAr), 28.7 (CH*C*$H_2CH_2CH_2$C-OH), 31.5 (*C*$H_2CH_2C_{quat}$-OAr), 32.1 (CH_2CH_2*C*H_2C_{quat}-OHC_{quat}-OAr), 34.7 (C_{quat}*C*H_2Ar), 37.3 (CH_2*C*H_2C_{quat}-OAr), 42.4 ($CHCH_2CH_2$*C*H_2C-OH), 43.8 (*C*$H_2CH_2CH_2C_{quat}$-OHC_{quat}-OAr), 52.0 (*C*$HCH_2CH_2C_{quat}$-OAr), 52.6 (*C*$HCH_2CH_2CH_2$C-OH), 82.4 ($CHCH_2CH_2CH_2$*C*-OH), 83.8 (*C*$_{quat}CH_2$Ar), 100.0 (*C*$_{quat}$-OHCquat-OAr), 109.1 (Ar*C*H), 120.0 (Ar*C*H), 125.0 (Ar*C*H), 127.2 (Ar*C*H), 127.8 (Ar*C*), 159.2 (Ar*C*) ppm. IR ν_{max} (neat/cm^{-1}): 3349, 2923, 1531, 1480, 1391, 1307, 1151, 1033, 799. HRMS calcd for $C_{20}H_{26}O_3Na$ $[M + Na]^+$: 337.1774, found 337.1760.

Spectroscopic data for minor diastereoisomer **7d'**: ^{1}H NMR (400 MHz, $CDCl_3$) δ 1.38 – 1.45 (m, 2 H, C*H*$_2CH_2C_{quat}$-OAr), 1.60 – 1.95 (m, 8 H, $CHCH_2$C*H*$_2CH_2$C-OH, CH_2C*H*$_2CH_2C_{quat}$-OHC_{quat}-OAr, 1 H from CH_2CH_2C*H*$_aH_bC_{quat}$-OHC_{quat}-OAr, 1 H from $CHCH_2CH_2$C*H*$_aH_b$C-OH, C*H*$_2CH_2CH_2C_{quat}$-OHC_{quat}-OAr), 2.01 – 2.36 (m, 7 H, 1 H from CHC*H*$_2CH_2CH_2$C-OH, 1 H from $CHCH_2CH_2CH_a$*H*$_b$C-OH, C*H*COH, 1 H from C*H*$_aH_bC_{quat}$-OAr, 1 H from $CH_2CH_2CH_a$*H*$_bC_{quat}$-OHC_{quat}-OAr, C*H*$CH_2CH_2C_{quat}$C-OAr), 2.50 (dt, *J* = 10.7, 7.6 Hz, 1 H, CH_a*H*$_bC_{quat}$-OAr), 2.79 (d, *J* = 15.6 Hz, 1 H, *CH*$_aH_b$-Ar), 2.95 (bs, 1 H, CO*H*), 3.32 (d, *J* = 15.6 Hz, 1 H, *C*H_a*H*$_b$-Ar), 6.76 – 6.89 (m, 2 H, ArC*H*), 7.09 – 7.17 (m, 2 H, ArC*H*) ppm. ^{13}C NMR (100 MHz, $CDCl_3$) δ 18.4 ($CHCH_2$*C*H_2CH_2C-OH) 23.1 (CH_2*C*$H_2CH_2C_{quat}$OH-C-OAr), 28.2 (CH*C*$H_2CH_2CH_2$C-OH), 29.8 (*C*$H_2CH_2C_{quat}$-OAr), 31.0 (CH_2*C*H_2C_{quat}-OAr), 33.6 (CH_2CH_2*C*H_2C_{quat}OH-C-OAr), 34.0 (*C*H_2-Ar), 42.4 ($CHCH_2CH_2$*C*H_2C-OH), 42.7 (*C*$H_2CH_2CH_2C_{quat}$OH-C-OAr), 50.2 (*C*$HCH_2CH_2C_{quat}$-OAr), 55.8 (*C*$HCH_2CH_2CH_2$C-OH), 82.3 ($CHCH_2CH_2CH_2$*C*-OH), 82.5 (*C*$_{quat}$-OH-C_{quat}-OAr), 101.6 (*C*$_{quat}$-OAr), 110.2 (Ar*C*H), 121.0 (Ar*C*H), 125.0 (Ar*C*H), 127.2 (Ar*C*H), 128.1 (Ar*C*), 157.8 (Ar*C*) ppm. IR ν_{max} (neat/cm^{-1}): 3399, 2928, 1741, 1613, 1480, 1461, 1280, 1100, 949, 880, 748. M.p. ($CHCl_3$) = 85 – 88 ˚C HRMS calcd for $C_{20}H_{26}O_3Na$ $[M + Na]^+$: 337.1747, found 337.1762.

***rac*-(3a*R*,6*R*,7*S*,8*S*,8a*S*)-7-Hydroxy-8-methyl-6-phenyloctahydro-1*H*-3a,6-(epoxymethano)azulen-9-one 7a**

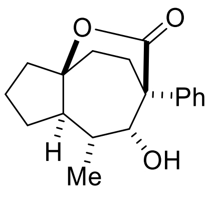

Prepared according to general procedure **H** using SmI_2 (9.00 mL, 0.90 mmol, 0.1 M in THF), diphenyl (*E*)-2-(9-bromo-3-oxononon-7-en-1-yl)-2-phenylmalonate (54.8 mg, 0.10 mmol) and H_2O (1.62 mL, 90 mmol) to give the title compound as a white solid (11.2 mg, 0.039 mmol,

39%). ^{1}H NMR (400 MHz, $CDCl_3$) δ 1.11 (d, *J* = 6.6 Hz, 3 H, $CHC\mathit{H}_3$), 1.46 – 1.68 (m, 2 H, 1 H from $CH_2C\mathit{H}_aH_bCH_2C$-O, 1 H from $CH_2CH_2C\mathit{H}_aH_bC$-O), 1.74 – 1.83 (m, 1 H, $C\mathit{H}CH_3$), 1.85 – 1.94 (m, 3 H, 1 H from $CH_2CH_a\mathit{H}_bCH_2C$-O, 1 H from $C\mathit{H}_aH_bCH_2C$, 1 H from $CH_2C\mathit{H}C$-O), 1.96 – 2.09 (m, 3 H, 1 H from $CH_2CH_2CH_a\mathit{H}_bC$-O, 1 H from $CH_2C\mathit{H}_aH_bC$, 1 H from $C\mathit{H}_aH_bCH_2CH_2C$-O), 2.13 – 2.31 (m, 2 H, 1 H from $CH_a\mathit{H}_bCH_2C$, 1 H from $CH_a\mathit{H}_bCH_2CH_2C$-O), 2.79 – 2.89 (m, 1 H, $CH_2CH_a\mathit{H}_bC$), 4.25 – 4.29 (m, 1 H, $C\mathit{H}OH$), 7.24 – 7.30 (m, 1 H, ArC*H*), 7.31 – 7.42 (m, 2 H, ArC*H*), 7.48 (dd, *J* = 7.4, 1.7 Hz, 2 H, ArC*H*) ppm. ^{13}C NMR (100 MHz, $CDCl_3$) δ 16.7 ($CH\mathit{C}H_3$), 22.9 ($CH_2\mathit{C}H_2CH_2C$-O), 24.6 ($CH_2\mathit{C}H_2C$), 32.1 ($\mathit{C}H_2CH_2CH_2C$-O), 32.6 ($CH_2CH_2\mathit{C}H_2C$-O), 39.1 ($\mathit{C}HCH_3$), 41.9 ($\mathit{C}H_2CH_2C$), 49.1 ($CH_2\mathit{C}HC$-O), 55.1 ($CH_2CH_2\mathit{C}$), 77.7 (*C*HOH), 90.7 ($CH_2CH_2CH_2\mathit{C}$-O), 126.9 (Ar*C*H), 127.9 (Ar*C*H), 128.1 (Ar*C*H), 143.2 (Ar*C*), 174.5 (*C*(O)O) ppm. IR ν_{max} (neat/cm^{-1}): 3442, 2958, 2359, 2342, 1711, 1496, 1445, 1345, 1231, 1192, 1176, 1138, 1122, 1075, 1054, 1013, 964, 937, 757, 731, 699. M.p. ($CHCl_3$) = 82 – 84 ˚C. HRMS calcd for $C_{18}H_{22}O_3K$ $[M + K]^+$: 325.1201, found 325.1198.

***rac*-(3a*R*,6*S*,7*S*,8*S*,8a*S*)-6-Ethyl-7-hydroxy-8-methyloctahydro-1*H*-3a,6-(epoxymethano)azulen-9-one 7b**

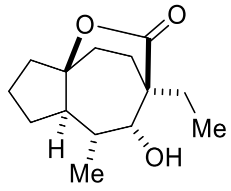

Prepared according to general procedure **H** using SmI_2 (9.00 mL, 0.90 mmol, 0.1 M in THF), diphenyl (*E*)-2-(9-bromo-3-oxononon-7-en-1-yl)-2-ethylmalonate (50.0 mg, 0.1 mmol) and H_2O (1.62 mL, 90 mmol) to give the title compound as a white solid (7.1 mg, 0.030 mmol, 30%). ^{1}H NMR (400 MHz, $CDCl_3$) δ 0.97 (t, *J* = 7.4 Hz, 3 H, $CCH_2C\mathit{H}_3$), 1.04 (d, *J* = 6.8 Hz, 3 H, $CHC\mathit{H}_3$), 1.37 – 1.56 (m, 4 H, 1 H from $CH_2C\mathit{H}_aH_bCH_2C$-O, 1 H from $CH_2CH_2C\mathit{H}_aH_bC$-O, 1 H from $CH_2C\mathit{H}_aH_bC$, 1 H from $C\mathit{H}CH_3$), 1.65 – 1.90 (m, 6 H, 1 H from $CH_2CH_a\mathit{H}_bCH_2C$-O, 1 H from $CH_2C\mathit{H}C$-O, 1 H from $C\mathit{H}_aH_bCH_2C$, 1 H from $C\mathit{H}_aH_bCH_2CH_2C$-O, 2 H from $CC\mathit{H}_2CH_3$), 1.92 – 2.01 (m, 1 H, $CH_2CH_2CH_a\mathit{H}_bC$-O), 2.03 – 2.14 (m, 2 H, 1 H from $CH_a\mathit{H}_bCH_2CH_2C$-O, 1 H from $CH_a\mathit{H}_bCH_2C$), 2.35 (ddd, *J* = 14.2, 10.6, 5.5 Hz, 1 H, $CH_2CH_a\mathit{H}_bC$), 3.59 – 3.65 (m, 1 H, $C\mathit{H}OH$) ppm. ^{13}C NMR (100 MHz, $CDCl_3$) δ 9.1 ($CCH_2\mathit{C}H_3$), 16.4 ($CH\mathit{C}H_3$), 20.1 ($CH_2\mathit{C}H_2C$), 22.9 ($CH_2\mathit{C}H_2CH_2C$-O), 30.5 ($C\mathit{C}H_2CH_3$), 32.1 ($\mathit{C}H_2CH_2CH_2C$-O), 32.5 ($CH_2CH_2\mathit{C}H_2C$-O), 38.9 ($\mathit{C}HCH_3$), 41.7 ($\mathit{C}H_2CH_2C$), 49.0 ($CH_2\mathit{C}HC$-O), 50.3 ($CH_2CH_2\mathit{C}$), 76.8 (*C*HOH), 90.3 ($CH_2CH_2CH_2\mathit{C}$-O), 176.2 (C(O)O) ppm.

IR ν_{max} (neat/cm^{-1}): 3457, 2961, 2877, 1709, 1460, 1352, 1232, 1180, 1155, 1125, 1093, 1032, 1001, 961. M.p. ($CHCl_3$) = 65 – 66 ˚C. HRMS calcd for $C_{14}H_{22}O_3Na$ [M + Na]$^+$: 261.1461, found 261.1452.

***rac*-(3a*R*,6*S*,7*S*,8*S*,8a*S*)-7-Hydroxy-6,8-dimethyloctahydro-1*H*-3a,6-(epoxymethano)azulen-9-one 7c**

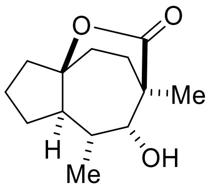

Prepared according to general procedure **H** using SmI_2 (9.00 mL, 0.90 mmol, 0.1 M in THF), diphenyl (*E*)-2-(9-bromo-3-oxonon-7-en-1-yl)-2-methylmalonate (48.6 mg, 0.10 mmol) and H_2O (1.62 mL, 90 mmol) to give the title compound as a white solid (10.1 mg, 0.045 mmol, 45%). ^{1}H NMR (400 MHz, $CDCl_3$) δ 1.03 (d, *J* = 6.7 Hz, 3 H, CHCH_3), 1.18 – 1.58 (m, 7 H, 3 H from CCH_3, 1 H from $CH_2C\mathit{H}_aH_bCH_2C$-O, 1 H from $CH_2CH_2C\mathit{H}_aH_bC$-O, 1 H from $CH_2C\mathit{H}_aH_bC$, 1 H from C*H*CH_3), 1.71 – 1.89 (m, 4 H, 1 H from CH_2C*H*C-O, 1 H from $CH_2CH_aH_bCH_2C$-O, 1 H from $C\mathit{H}_aH_bCH_2CH_2C$-O, 1 H from $C\mathit{H}_aH_bCH_2C$), 1.90 – 2.01 (m, 1 H, $CH_2CH_2CH_a\mathit{H}_bC$-O), 2.01 – 2.14 (m, 2 H, 1 H from $CH_a\mathit{H}_bCH_2CH_2C$-O, 1 H from $CH_a\mathit{H}_bCH_2C$), 2.50 (ddd, *J* = 13.8, 10.6, 6.0 Hz, 1 H, $CH_2CH_a\mathit{H}_bC$), 3.58 – 3.63 (m, 1 H, C*H*OH) ppm. ^{13}C NMR (100 MHz, $CDCl_3$) δ 16.1 (CH*C*H_3), 23.1 ($CH_2\mathit{C}H_2CH_2C$-O), 24.0 ($CH_2\mathit{C}H_2C$), 25.0 (C*C*H_3), 32.5 ($\mathit{C}H_2CH_2CH_2C$-O), 32.7 ($CH_2CH_2\mathit{C}H_2C$-O), 38.6 (*C*HCH_3), 41.8 ($\mathit{C}H_2CH_2C$), 46.8 ($CH_2CH_2\mathit{C}$), 48.4 (CH_2*C*HC-O), 77.2 (*C*HOH), 90.7 ($CH_2CH_2CH_2\mathit{C}$-O), 177.1 (C(O)O) ppm. IR ν_{max} (neat/cm^{-1}): 3459, 2963, 2359, 2343, 1710, 1457, 1381, 1350, 1234, 1181, 1156, 1121, 1092, 1008, 959, 938, 911, 731. M.p. ($CHCl_3$) = 65 – 67 ˚C. HRMS calcd for $C_{13}H_{20}O_3K$ [M + K]$^+$: 263.1044, found 263.1043.

***rac*-(5a*R*,8a*S*,9*S*)-5a-Hydroxy-3,3,9-trimethyl-4,5,5a,6,7,8,8a,9-octahydroazuleno[5,6-b]furan-2(3*H*)-one 7d**

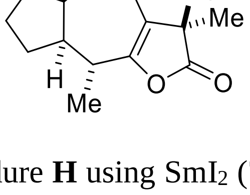

Prepared according to general procedure **H** using SmI_2 (7.00 mL, 0.70 mmol, 0.1 M in THF), diphenyl (*E*)-2-(9-bromo-3-oxonon-7-en-1-yl)-2-isopropylmalonate (51.4 mg, 0.10 mmol) and H_2O (1.26 mL, 70.0 mmol) to give the title compound as a white solid (15.1 mg, 0.060 mmol,

60%). ^{1}H NMR (400 MHz, $\mathrm{CDCl_3}$) δ 1.22 (d, *J* = 7.6 Hz, 3 H, $\mathrm{CHC}H_3$), 1.23 (s, 3 H, C=C-$\mathrm{C(C}H_3\mathrm{)_a(CH_3)_b}$), 1.25 (s, 3 H, $\mathrm{C(CH_3)_a(C}H_3\mathrm{)_b}$), 1.49 – 1.68 (m, 4 H, 1 H from C*H*C-OH, 1 H from $\mathrm{CH_2C}H_a\mathrm{H_bCH_2C}$-OH, 1 H from $\mathrm{C}H_a\mathrm{H_bCH_2CH_2C}$-OH, 1 H from $\mathrm{C}H_a\mathrm{H_bCH_2C}$=C-O), 1.72 – 1.84 (m, 3 H, 1 H from $\mathrm{CH_2CH_a}H_b\mathrm{CH_2C}$-OH, 2 H from $\mathrm{CH_2CH_2C}H_2\mathrm{C}$-OH), 2.00 (dt, *J* = 16.5, 4.1 Hz, 1 H, $\mathrm{CH_2C}H_a\mathrm{H_bC}$=C-O), 2.03 – 2.14 (m, 2 H, 1 H from $\mathrm{CH_a}H_b\mathrm{CH_2CH_2C}$-OH, 1 H from $\mathrm{CH_a}H_b\mathrm{CH_2C}$=C-O), 2.33 (ddt, *J* = 16.5, 12.4, 4.1 Hz, 1 H, $\mathrm{CH_2CH_a}H_b\mathrm{C}$=C-O), 2.56 – 2.66 (m, 1 H, $\mathrm{C}H\mathrm{CH_3}$) ppm. ^{13}C NMR (100 MHz, $\mathrm{CDCl_3}$) δ 16.7 ($\mathrm{CH}C\mathrm{H_3}$), 18.0 ($\mathrm{CH_2}C\mathrm{H_2C}$=C-O), 20.0 ($\mathrm{CH_2}C\mathrm{H_2CH_2C}$-OH), 22.6 ($\mathrm{C(}C\mathrm{H_3)_a(CH_3)_b}$), 23.1 ($\mathrm{C(CH_3)_a(}C\mathrm{H_3)_b}$), 30.8 ($C\mathrm{H_2CH_2CH_2C}$-OH), 33.1 ($C\mathrm{HCH_3}$), 37.6 ($C\mathrm{H_2CH_2C}$=C-O), 43.5 ($\mathrm{CH_2CH_2}C\mathrm{H_2C}$-OH), 46.8 (C=C-$C\mathrm{(CH_3)_2}$), 52.3 (*C*HC-OH), 81.0 (*C*-OH), 121.5 (*C*=C-O), 149.3 (C=*C*-O), 182.6 (*C*(O)O) ppm. IR ν_{max} (neat/cm^{-1}): 3499, 2964, 2930, 2360, 2342, 1789, 1463, 1380, 1280, 1098, 1066, 1021, 996, 933. M.p. ($\mathrm{CHCl_3}$) = 65 – 67 ˚C. HRMS calcd for $\mathrm{C_{15}H_{23}O_3}$ [M + H]$^+$: 251.1642, found 251.1638.

***rac*-(5a*R*,8a*S*,9*S*)-5a-Hydroxy-9-methyl-4,5,5a,6,7,8,8a,9-octahydro-2*H*-spiro[azuleno[5,6-b]furan-3,1'-cyclopentan]-2-one 7e**

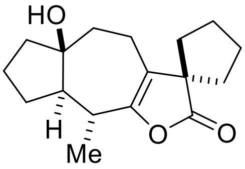

Prepared according to general procedure **H** using $\mathrm{SmI_2}$ (7.00 mL, 0.70 mmol, 0.1 M in THF), diphenyl (*E*)-2-(9-bromo-3-oxonon-7-en-1-yl)-2-cyclopentylmalonate (54.0 mg, 0.10 mmol) and $\mathrm{H_2O}$ (1.26 mL, 70.0 mmol) to give the title compound as a white solid (15.4 mg, 0.056 mmol, 56%). ^{1}H NMR (500 MHz, $\mathrm{CDCl_3}$) δ 1.21 (d, *J* = 7.0 Hz, 3 H, $\mathrm{CHC}H_3$), 1.47 – 1.67 (m, 4 H, 1 H from $\mathrm{C}H_a\mathrm{H_bCH_2C}$=C-O, 1 H from C*H*C-OH, 1 H from $\mathrm{C}H_a\mathrm{H_bCH_2CH_2C}$-OH, 1 H from $\mathrm{CH_2C}H_a\mathrm{H_bCH_2C}$-OH), 1.70 – 1.83 (m, 7 H, 1 H from $\mathrm{CH_2CH_a}H_b\mathrm{CH_2C}$-OH, 1 H from $\mathrm{CH_2CH_2C}H_a\mathrm{H_bCH_2C}$, 1 H from $\mathrm{CH_2C}H_a\mathrm{H_bCH_2CH_2C}$, 1 H from $\mathrm{CH_2CH_2CH_2C}H_a\mathrm{H_bC}$, 1 H from $\mathrm{C}H_a\mathrm{H_bCH_2CH_2CH_2C}$, 2 H from $\mathrm{CH_2CH_2C}H_2\mathrm{C}$-OH), 1.86 – 2.01 (m, 5 H, 1 H from $\mathrm{CH_2CH_2CH_a}H_b\mathrm{CH_2C}$, 1 H from $\mathrm{CH_2CH_a}H_b\mathrm{CH_2CH_2C}$, 1 H from $\mathrm{CH_2CH_2CH_2CH_a}H_b\mathrm{C}$, 1 H from $\mathrm{CH_a}H_b\mathrm{CH_2CH_2CH_2C}$, 1 H from $\mathrm{CH_2C}H_a\mathrm{H_bC}$=C-O), 2.02 – 2.14 (m, 2 H, 1 H from $\mathrm{CH_a}H_b\mathrm{CH_2CH_2C}$-OH, 1 H from $\mathrm{CH_a}H_b\mathrm{CH_2C}$=C-O), 2.30 – 2.41 (m, 1 H, $\mathrm{CH_2CH_a}H_b\mathrm{C}$=C-O), 2.59 (dqd, *J* = 9.6, 7.0, 3.0 Hz, 1 H, $\mathrm{C}H\mathrm{CH_3}$) ppm. ^{13}C NMR (125 MHz, $\mathrm{CDCl_3}$) δ 16.7 ($\mathrm{CH}C\mathrm{H_3}$), 18.3 ($\mathrm{CH_2}C\mathrm{H_2C}$=C-O), 19.9 ($\mathrm{CH_2}C\mathrm{H_2CH_2C}$-OH), 26.9 ($\mathrm{CH_2CH_2}C\mathrm{H_2CH_2C}$), 27.0 ($\mathrm{CH_2}C\mathrm{H_2CH_2CH_2C}$), 30.7 ($C\mathrm{H_2CH_2CH_2C}$-OH), 33.1 ($C\mathrm{HCH_3}$), 35.4 ($C\mathrm{H_2CH_2CH_2CH_2C}$),

35.9 ($CH_2CH_2CH_2\mathit{C}H_2C$), 37.7 ($\mathit{C}H_2CH_2C$=C-O), 43.4 ($CH_2CH_2\mathit{C}H_2C$-OH), 52.3 (*C*HC-OH), 55.9 ($CH_2CH_2CH_2CH_2\mathit{C}$), 80.9 (*C*-OH), 120.0 (*C*=C-O), 149.4 (C=*C*-O), 184.2 (*C*(O)O) ppm. IR ν_{max} (neat/cm^{-1}): 3496, 2956, 2870, 2359, 2342, 1763, 1683, 1447, 1379, 1323, 1292, 1263, 1223, 1162, 1120, 1064, 1011, 991, 937, 853, 748. M.p. ($CHCl_3$) = 78 – 80 ˚C. HRMS calcd for $C_{17}H_{24}O_3Na$ [M + Na]$^+$: 299.1618, found 299.1607.

***rac*-(5a*R*,8a*S*,9*S*)-5a-Hydroxy-9-methyl-4,5,5a,6,7,8,8a,9-octahydro-2*H*-spiro[azuleno[5,6-b]furan-3,1'-cyclohexan]-2-one 7f**

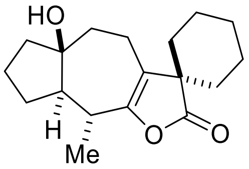

Prepared according to general procedure **H** using SmI_2 (7.00 mL, 0.70 mmol, 0.1 M in THF), diphenyl (*E*)-2-(9-bromo-3-oxonon-7-en-1-yl)-2-cyclohexylmalonate (55.4 mg, 0.10 mmol) and H_2O (1.26 mL, 70.0 mmol) to give the title compound as a white solid (14.8 mg, 0.051 mmol, 51%). ^{1}H NMR (400 MHz, $CDCl_3$) δ 1.14 – 1.19 (m, 1 H, $CH_2CH_2C\mathit{H}_aH_bCH_2CH_2C$), 1.20 (d, *J* = 7.1 Hz, 3 H, $CHC\mathit{H}_3$), 1.46 – 1.66 (m, 10 H, 2 H from $C\mathit{H}_2CH_2CH_2CH_2CH_2C$, 2 H from $CH_2CH_2CH_2CH_2C\mathit{H}_2C$, 1 H from $CH_2C\mathit{H}_aH_bCH_2CH_2CH_2C$, 1 H from $CH_2CH_2CH_2C\mathit{H}_aH_bCH_2C$, 1 H from $C\mathit{H}_aH_bCH_2CH_2C$-OH, 1 H from $CH_2C\mathit{H}_aH_bCH_2C$-OH, 1 H from $C\mathit{H}_aH_bCH_2C$=C-O, 1 H from C*H*C-OH), 1.71 – 1.83 (m, 4 H, 1 H from $CH_2CH_2CH_a\mathit{H}_bCH_2CH_2C$, 1 H from $CH_2CH_a\mathit{H}_bCH_2C$-OH, 2 H from $CH_2CH_2C\mathit{H}_2C$-OH), 1.93 – 2.12 (m, 5 H, 1 H from $CH_2CH_a\mathit{H}_bCH_2CH_2CH_2C$, 1 H from $CH_2CH_2CH_2CH_a\mathit{H}_bCH_2C$, 1 H from $CH_a\mathit{H}_bCH_2CH_2C$-OH, 1 H from $CH_a\mathit{H}_bCH_2C$=C-O, 1 H from $CH_2C\mathit{H}_aH_bC$=C-O), 2.19 – 2.31 (m, 1 H, $CH_2CH_a\mathit{H}_bC$=C-O), 2.61 (dqd, *J* = 9.9, 7.1, 2.7 Hz, 1 H, $C\mathit{H}CH_3$) ppm. ^{13}C NMR (100 MHz, $CDCl_3$) δ 16.5 ($CH\mathit{C}H_3$), 17.7 ($CH_2\mathit{C}H_2C$=C-O), 19.8 ($CH_2\mathit{C}H_2CH_2C$-OH), 20.0 ($CH_2\mathit{C}H_2CH_2CH_2CH_2C$), 20.1 ($CH_2CH_2CH_2\mathit{C}H_2CH_2C$), 25.0 ($CH_2CH_2\mathit{C}H_2CH_2CH_2C$), 30.5 ($\mathit{C}H_2CH_2CH_2C$-OH), 30.5 ($\mathit{C}H_2CH_2CH_2CH_2CH_2C$), 31.0 ($CH_2CH_2CH_2CH_2\mathit{C}H_2C$), 32.9 ($\mathit{C}HCH_3$), 37.9 ($\mathit{C}H_2CH_2C$=C-O), 43.4 ($CH_2CH_2\mathit{C}H_2C$-OH), 49.0 ($CH_2CH_2CH_2CH_2CH_2\mathit{C}$), 52.1 (*C*HC-OH), 80.9 (*C*-OH), 121.3 (*C*=C-O), 149.8 (C=*C*-O), 180.5 (*C*(O)O) ppm. IR ν_{max} (neat/cm^{-1}): 3489, 2929, 2851, 2358, 2343, 1778, 1764, 1683, 1448, 1380, 1357, 1312, 1263, 1209, 1120, 1065, 1019, 993, 976, 953, 930, 877, 850, 753. M.p. ($CHCl_3$) = 79 – 82 ˚C. HRMS calcd for $C_{18}H_{26}O_3Na$ [M + Na]$^+$: 313.1774, found 313.1765.

***r*ac-(5a*R*,8a*S*,9*S*)-5a-Hydroxy-9-methyl-4,5,5a,6,7,8,8a,9-octahydro-2*H*-spiro[azuleno[5,6-b]furan-3,1'-cycloheptan]-2-one 7g**

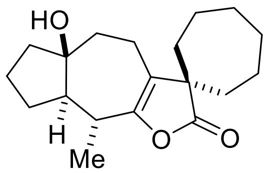

Prepared according to general procedure **H** using SmI_2 (7.00 mL, 0.70 mmol, 0.1 M in THF), diphenyl (*E*)-2-(9-bromo-3-oxonon-7-en-1-yl)-2-cycloheptylmalonate (56.8 mg, 0.10 mmol) and H_2O (1.26 mL, 70.0 mmol) to give the title compound as a colourless oil (12.8 mg, 0.042 mmol, 42%). ^{1}H NMR (400 MHz, $CDCl_3$) δ 1.19 (d, *J* = 7.0 Hz, 3 H, $CHCH_3$), 1.46 – 1.71 (m, 10 H, 2 H from $CH_2CH_2CH_2CH_2CH_2CH_2C$, 2 H from $CH_2CH_2CH_2CH_2CH_2CH_2C$, 1 H from CHC-OH, 1 H from $CH_aH_bCH_2CH_2C$-OH, 1 H from $CH_2CH_aH_bCH_2C$-OH, 1 H from $CH_aH_bCH_2C$=C-O, 1 H from $CH_2CH_2CH_aH_bCH_2CH_2CH_2C$, 1 H from $CH_2CH_2CH_2CH_aH_bCH_2CH_2C$), 1.72 – 1.84 (m, 7 H, 1 H from $CH_2CH_aH_bCH_2C$-OH, 2 H from $CH_2CH_2CH_2CH_2CH_2CH_2C$, 2 H from $CH_2CH_2CH_2CH_2CH_2CH_2C$, 2 H from $CH_2CH_2CH_2C$-OH), 1.85 – 2.00 (m, 2 H, 1 H from $CH_2CH_2CH_aH_bCH_2CH_2CH_2C$, 1 H from $CH_2CH_2CH_2CH_aH_bCH_2CH_2C$), 2.01 – 2.19 (m, 3 H, 1 H from $CH_aH_bCH_2CH_2C$-OH, 1 H from $CH_aH_bCH_2C$=C-O, 1 H from $CH_2CH_aH_bC$=C-O), 2.31 – 2.43 (m, 1 H, $CH_2CH_aH_bC$=C-O), 2.51 – 2.63 (m, 1 H, $CHCH_3$) ppm. ^{13}C NMR (100 MHz, $CDCl_3$) δ 16.8 ($CHCH_3$), 18.4 (CH_2CH_2C=C-O), 20.0 ($CH_2CH_2CH_2C$-OH), 23.4 ($CH_2CH_2CH_2CH_2CH_2CH_2C$), 23.4 ($CH_2CH_2CH_2CH_2CH_2CH_2C$), 30.7 ($CH_2CH_2CH_2C$-OH), 31.2 ($CH_2CH_2CH_2CH_2CH_2CH_2C$), 31.3 ($CH_2CH_2CH_2CH_2CH_2CH_2C$), 33.1 ($CHCH_3$), 35.3 ($CH_2CH_2CH_2CH_2CH_2CH_2C$), 35.5 ($CH_2CH_2CH_2CH_2CH_2CH_2C$), 37.9 ($CH_2CH_2C$=C-O), 43.4 ($CH_2CH_2CH_2C$-OH), 52.2 (CHC-OH), 56.5 ($CH_2CH_2CH_2CH_2CH_2CH_2C$), 81.0 (*C*-OH), 123.2 (*C*=C-O), 148.6 (C=*C*-O), 182.9 (*C*(O)O) ppm. IR ν_{max} (neat/cm^{-1}): 3499, 2924, 2856, 2359, 2342, 1761, 1457, 1293, 1260, 1218, 1169, 1066, 1018, 993, 945. HRMS calcd for $C_{19}H_{28}O_3K$ $[M + K]^+$: 343.1670, found 343.1671.

(5a*R*,8a*S*,9*S*)-5a-Hydroxy-9-methyl-2',3',4,5,5a,5',6,6',7,8,8a,9-dodecahydro-2H-spiro[azuleno[5,6-b]furan-3,4'-pyran]-2-one 7h

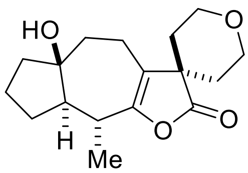

Prepared according to general procedure **H** using SmI_2 (7.00 mL, 0.70 mmol, 0.1 M in THF), diphenyl (*E*)-2-(9-bromo-3-oxononon-7-en-1-yl)-2-cyclohexylmalonate (55.7 mg, 0.10 mmol) and H_2O (1.26 mL, 70.0 mmol) to give the title compound as a white solid (15.1 mg, 0.051 mmol, 51%). ^{1}H NMR (400 MHz, $CDCl_3$) δ 1.19 (d, *J* = 7.0 Hz, 3 H, CHCH_3), 1.38 – 1.65 (m, 5 H, C*H*CH_3, C$H_2$$CH_2$-O, C$H_2$$CH_2$-O), 1.69 – 1.83 (m, 4 H, CH_2C$H_2$$CH_2$C-OH, $CH_2$$CH_2CH_2$C-OH), 1.81 – 2.14 (m, 6 H, CH_2CH_2C=, C$H_2$$CH_2$C=C, C$H_2$$CH_2$$CH_2$C-OH), 2.64 (pd, *J* = 7.2, 3.6 Hz, 1 H, C*H*C-OH), 3.81 (ddd, *J* = 11.9, 5.6, 2.5 Hz, 2 H, CH_2CH_2-O), 4.16 (tt, *J* = 12.3, 2.3 Hz, 2 H, CH_2CH_2-O) ppm. ^{13}C NMR (100 MHz, $CDCl_3$) δ 16.5 (CH*C*H_3), 17.7 (CH_2*C*H_2C=C), 19.8 (*C*$H_2$$CH_2$$CH_2$C-OH), 29.7 ($CH_2$*C*$H_2$$CH_2$C-OH), 30.3 (*C*$H_2$$CH_2$-O), 30.6 (*C*$H_2$$CH_2$-O), 33.0 (*C*H$CH_3$), 37.8 (*C*$H_2$$CH_2$C=C), 43.7 ($CH_2$$CH_2$*C*$H_2$C-OH), 46.6 (*C*), 52.1 (*C*HC-OH), 62.0 (CH_2*C*H_2-O), 62.2 (CH_2*C*H_2-O), 80.9 (*C*-OH), 120.1 (*C*=C-O), 150.8 (C=*C*-O), 180.4 (*C*(O)O) ppm. IR ν_{max} (neat/cm^{-1}): 3465, 2948, 1775, 1217, 1099, 1020. M.p. ($CHCl_3$) = 149 – 151 ˚C. HRMS calcd for $C_{17}H_{25}O_4$ [M + H]$^+$: 293.1747, found 293.1739.

(5a*R*,8a*S*,9*S*)-5a-Hydroxy-3,3-dimethyl-9-(3-phenylpropyl)-4,5,5a,6,7,8,8a,9-octahydroazuleno[5,6-b]furan-2(3H)-one 7i

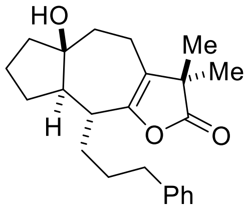

Prepared according to general procedure **H** using SmI_2 (12.00 mL, 1.20 mmol, 0.1 M in THF), diphenyl (*E*)-2-isopropyl-2-(3-oxo-11-phenyl-9-((4-(trifluoromethyl)benzoyl)oxy)undec-7-en-1-yl)malonate (72.8 mg, 0.1 mmol) and H_2O (2.16 mL, 120 mmol) to give the title compound as a colourless oil (19.8 mg, 0.055 mmol, 56%). ^{1}H NMR (400 MHz, $CDCl_3$) δ 1.22 (s, 3 H, C=C-C(C*H*$_3$)$_a$(CH_3)$_b$), 1.23 (s, 3 H, C=C-C(CH_3)$_a$(C*H*$_3$)$_b$), 1.46 – 1.65 (m, 7 H, 1 H from CH_2C*H*$_a$H$_b$$CH_2$C-OH, C$H_2$$CH_2$$CH_2Ph, CH_2CH_2$$CH_2Ph, CH_2$$CH_2$$CH_2$C-OH), 1.66 – 1.81 (m, 4 H, C*H*C-OH CH_2CH$_a$*H*$_b$$CH_2$C-OH, C$H_2$$CH_2$C=C), 1.86 – 2.14 (m, 4 H, $CH_2$$CH_2CH_2$C-

OH, 1 H from $CH_2CH_aH_bC$=C, CHC-O*H*), 2.25 (dddd, *J* = 16.6, 12.0, 4.7, 3.0 Hz, 1 H, $CH_2CH_aH_bC$=C), 2.46 – 2.60 (m, 1 H, 1 H from C*H*$CH_2CH_2CH_2$Ph), 2.60 – 2.74 (m, 2 H, $CH_2CH_2CH_2$Ph), 7.14 – 7.20 (m, 2 H, ArC*H*), 7.27 (d, *J* = 5.7 Hz, 3 H, ArC*H*) ppm. ^{13}C NMR (100 MHz, $CDCl_3$) δ 18.3 (CH_2CH_2C=C), 20.3 ($CH_2CH_2CH_2$C-OH), 22.6 (C(CH_3)$_2$), 23.5 (C(CH_3)$_2$), 27.2 ($CH_2CH_2CH_2$Ph), 29.3 ($CH_2CH_2CH_2$C-OH), 29.9 (CH_2CH_2C=C), 30.6 ($CH_2CH_2CH_2$Ph), 36.5 (*C*H$CH_2CH_2CH_2$Ph), 37.9 ($CH_2CH_2CH_2$Ph), 43.6 ($CH_2CH_2CH_2$C-OH), 46.8 (*C*(CH_3)$_2$), 48.4 (*C*HC-OH), 81.2 (*C*-OH), 123.0 (*C*=C-O), 125.9 (Ar*C*H), 128.4 (Ar*C*H), 128.5 (Ar*C*H), 142.7 (C=*C*-O), 148.2 (Ar*C*), 182.8 (C(O)O) ppm. IR ν_{max} (neat/cm^{-1}): 3496, 2929, 1787, 1453, 1279, 1074, 699. HRMS calcd for $C_{23}H_{31}O_3$ [M + H]$^+$: 355.2195, found 355.2190.

(5a*R*,8a*S*,9*S*)-9-(4-((*tert*-Butyldimethylsilyl)oxy)butyl)-5a-hydroxy-3,3-dimethyl-4,5,5a,6,7,8,8a,9-octahydroazuleno[5,6-b]furan-2(3H)-one 7j

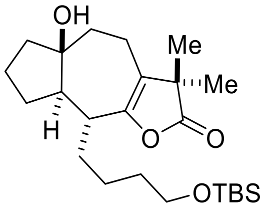

Prepared according to general procedure **H** using SmI_2 (6.02 mL, 0.60 mmol, 0.1 M in THF), diphenyl (*E*)-2-(12-((*tert*-butyldimethylsilyl)oxy)-3-oxo-9-((4-(trifluoromethyl)benzoyl)oxy)dodec-7-en-1-yl)-2-isopropylmalonate (40.0 mg, 0.05 mmol) and H_2O (1.08 mL, 60.0 mmol) to give the title compound as a colourless oil (11.6 mg, 0.027 mmol, 55%). ^{1}H NMR (400 MHz, $CDCl_3$) δ -0.02 – 0.10 (m, 6 H, Si(CH_3)$_2$), 0.81 – 0.96 (m, 9 H, SiC(CH_3)$_3$), 1.22 (s, 3 H, C=C-C(CH_3)$_a$(CH_3)$_b$), 1.24 (s, 3 H, C=C-C(CH_3)$_a$(CH_3)$_b$), 1.33 – 1.67 (m, 9 H, $CH_2CH_2CH_2CH_2$O-Si, $CH_2CH_2CH_2CH_2$O-Si, $CH_2CH_2CH_2CH_2$O-Si, $CH_2CH_2CH_2$C-OH, 1 H from $CH_2CH_aH_bCH_2$C-OH), 1.68 – 1.97 (m, 5 H, C*H*C-OH, CH_2CH_2C=C, 1 H from $CH_2CH_aH_bCH_2$C-OH, 1 H from $CH_2CH_2CH_aH_b$C-OH), 1.98 – 2.52 (m, 4 H, 1 H from $CH_2CH_2CH_aH_b$C-OH, CH_2CH_2C=C, CHC-O*H*), 2.63 (m, 1 H, C*H*$CH_2CH_2CH_2CH_2$O-Si), 3.55 – 3.68 (m, 2 H, $CH_2CH_2CH_2CH_2$O-Si) ppm. ^{13}C NMR (100 MHz, $CDCl_3$) δ -5.1 (Si-CH_3), 18.3(CH_2CH_2C=C), 18.5 (Si*C*(CH_3)$_3$), 20.4 ($CH_2CH_2CH_2$C-OH), 21.2 ($CH_2CH_2CH_2CH_2$O-Si), 22.6 (C(CH_3)$_2$), 23.5 (C(CH_3)$_2$), 26.1 (SiC(CH_3)$_3$) 29.3 ($CH_2CH_2CH_2$C-OH), 30.8 ($CH_2CH_2CH_2CH_2$O-Si), 33.4 ($CH_2CH_2CH_2CH_2$O-Si), 38.0 (*C*H$CH_2CH_2CH_2CH_2$O-Si), 38.0 (CH_2CH_2C=C) 43.6 ($CH_2CH_2CH_2$C-OH), 46.8 (*C*(CH_3)$_2$), 48.4 (*C*HC-OH), 63.2 ($CH_2CH_2CH_2CH_2$O-Si), 81.3 (*C*-OH), 122.8 (*C*=C-O), 148.3 (C=*C*-O),

182.7 (C(O)O) ppm. IR ν_{max} (neat/cm^{-1}): 3481, 2928, 1790, 1693, 1462, 1279, 1256, 1098, 836, 775, 668. . HRMS calcd for $C_{24}H_{42}O_4SiNa$ [M + Na]$^+$: 445.2745, found 445.2748.

***rac*-(3a*R*,7*S*,8*R*,9*S*,9a*S*)-6-((*E*)-Benzylidene)-9-methyloctahydro-3a,8-epoxycyclopenta[8]annulene-7,8(1*H*)-diol 7k**

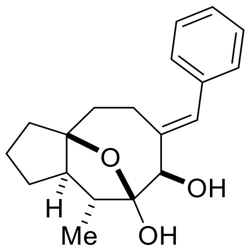

General procedure **I**. To a solution of SmI_2 (7.00 mL, 0.70 mmol, 0.1 M in THF), diphenyl (*E*)-2-benzyl-2-(9-bromo-3-oxonon-7-en-1-yl)malonate (56.2 mg, 0.10 mmol) in THF (0.70 mL) was added dropwise under nitrogen and stirred for 14 h at room temperature. After that time degassed MeOH (340 µL, 8.4 mmol) was added and the reaction was continued at the same temperature for 48 h before being quenched with air followed by saturated aqueous Rochelle salt (10 mL) and a few drops of saturated aqueous sodium thiosulfate. The aqueous layer was extracted with Et_2O (3 × 15 mL) and the combined organic layers were washed with brine (15 mL), dried over $MgSO_4$ and concentrated *in vacuo.* Purification by column chromatography eluting with Et_2O/Pentane (20:80) gave the title compound as a white solid (13.2 mg, 0.044 mmol, 44%). ^{1}H NMR (400 MHz, $CDCl_3$) δ 1.11 (d, J = 7.1 Hz, 3 H, CHCH_3), 1.35 (td, J = 13.3, 6.5 Hz, 1 H, $CH_2CH_2CH_aH_bC$-O), 1.52 – 1.59 (m, 2 H, $CH_2CH_2CH_2C$-O), 1.60 – 1.68 (m, 1 H, $CH_2CH_aH_bCH_2C$-O), 1.69 – 1.81 (m, 2 H, 1 H from $CH_2CH_aH_bCH_2C$-O, 1 H from $CH_aH_bCH_2C$=CH), 1.90 (dq, J = 10.1, 7.2 Hz, 1 H, CHCH$_3$), 1.99 – 2.07 (m, 2 H, 1 H from $CH_2CH_2CH_aH_bC$-O, 1 H from CHC-O), 2.21 (ddd, J = 13.6, 7.9, 1.4 Hz, 1 H from $CH_aH_bCH_2C$=CH), 2.49 (d, J = 7.0 Hz, 1 H, CHOH), 2.47 – 2.54 (m, 1 H, $CH_2CH_aH_bC$=CH), 2.60 (ddd, J = 13.8, 11.3, 7.9 Hz, 1 H, $CH_2CH_aH_bC$=CH), 4.20 (s, 1 H, OCOH), 4.27 (d, J = 6.9 Hz, 1 H, CHOH), 6.68 (s, 1 H, CH_2CH_2C=CH), 7.29 – 7.34 (m, 3 H, ArCH), 7.35 – 7.42 (m, 2 H, ArCH) ppm. ^{13}C NMR (100 MHz, $CDCl_3$) δ 12.3 (CHCH_3), 21.0 (CH_2CH_2C=CH), 23.0 ($CH_2CH_2CH_2C$-O), 31.9 ($CH_2CH_2CH_2C$-O), 39.5 (CH_2CH_2C=CH), 41.3 ($CH_2CH_2CH_2C$-O), 47.2 (CHCH$_3$), 55.0 (CHC-O), 79.4 (CHOH), 89.8 (C-O), 106.3 (OCO), 127.3 (ArCH), 128.4 (ArCH), 128.5 (ArCH), 132.6 (CH_2CH_2C=CH), 136.6 (ArC), 138.7 (CH_2CH_2C=CH) ppm. IR ν_{max} (neat/cm^{-1}): 3456, 2935, 2369, 1265, 1494, 1457, 1338, 1261, 1261, 1155, 1096, 1060, 1040, 1018, 966, 952, 876, 750. M.p. ($CHCl_3$) = 80 – 82 ˚C. HRMS calcd for $C_{19}H_{24}O_3Na$ [M + Na]$^+$: 323.1618, found 323.1610.

***rac*-(3a*R*,7*S*,8*R*,9*S*,9a*S*)-6-((*E*)-4-Chlorobenzylidene)-9-methyloctahydro-3a,8-epoxycyclopenta[8]annulene-7,8(1*H*)-diol 7l**

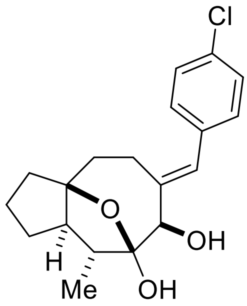

Prepared according to general procedure **I** using SmI_2 (7.00 mL, 0.70 mmol, 0.1 M in THF), diphenyl (*E*)-2-(9-bromo-3-oxonon-7-en-1-yl)-2-(4-chlorobenzyl)malonate (60 mg, 0.10 mmol) and MeOH (340 µL, 8.4 mmol) to give the title compound as a white solid (14.0 mg, 0.042 mmol, 42%). ^{1}H NMR (400 MHz, $CDCl_3$) δ 1.09 (d, *J* = 6.8 Hz, 3 H, $CHCH_3$), 1.24 - 1.38 (m, 1 H, $CH_2CH_2CH_aH_bC$-O), 1.51 – 1.57 (m, 2 H, $CH_2CH_2CH_2C$-O), 1.59 – 1.65 (m, 1 H, $CH_2CH_aH_bCH_2C$-O), 1.65 – 1.79 (m, 2 H, 1 H from $CH_2CH_aH_bCH_2C$-O, 1 H from $CH_aH_bCH_2C$=CH), 1.88 (dq, *J* = 10.1, 7.2 Hz, 1 H, $CHCH_3$), 1.94 – 2.05 (m, 2 H, 1 H from $CH_2CH_2CH_aH_bC$-O, 1 H from CHC-O), 2.16 – 2.23 (m, 1 H, $CH_aH_bCH_2C$=CH), 2.38 – 2.45 (m, 1 H, $CH_2CH_aH_bC$=CH), 2.57 (d, *J* = 6.8 Hz, 1 H, CHO*H*), 2.58 – 2.66 (m, 1 H, $CH_2CH_aH_bC$=CH), 4.18 (s, 1 H, OCO*H*), 4.24 (d, *J* = 6.8 Hz, 1 H, C*H*OH), 6.60 (s, 1 H, CH_2CH_2C=C*H*), 7.21 (d, *J* = 8.5 Hz, 2 H, ArC*H*), 7.33 (d, *J* = 8.5 Hz, 2 H, ArC*H*) ppm. ^{13}C NMR (100 MHz, $CDCl_3$) δ 12.5 ($CHCH_3$), 21.1 (CH_2CH_2C=CH), 23.1 ($CH_2CH_2CH_2C$-O), 32.1 ($CH_2CH_2CH_2C$-O), 39.6 (CH_2CH_2C=CH), 41.4 ($CH_2CH_2CH_2C$-O), 47.4 ($CHCH_3$), 55.2 (*C*HC-O), 79.4 (*C*HOH), 89.9 (*C*-O), 106.4 (O*C*O), 128.8 (Ar*C*H), 129.9 (Ar*C*H), 131.4 (CH_2CH_2C=*C*H), 133.3 (Ar*C*), 135.2 (Ar*C*), 139.7 (CH_2CH_2*C*=CH) ppm. IR ν_{max} (neat/cm^{-1}): 3402, 2933, 1489, 1457, 1392, 1338, 1259, 1155, 1118, 1095, 1061, 1040, 1014, 967, 953, 940, 926, 908, 876, 820, 790, 732, 668. M.p. ($CHCl_3$) = 87 – 89 ˚C. HRMS calcd for $C_{19}H_{23}ClO_3Na$ $[M + Na]^+$: 357.1233, found 357.1229.

***rac*-(3a*R*,7*S*,8*R*,9*S*,9a*S*)-6-((*E*)-4-Fluorobenzylidene)-9-methyloctahydro-3a,8-epoxycyclopenta[8]annulene-7,8(1*H*)-diol 7m**

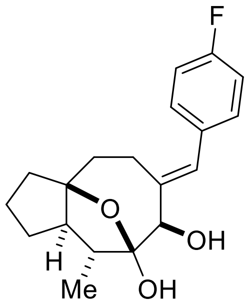

Prepared according to general procedure **I** using SmI_2 (7.00 mL, 0.70 mmol, 0.1 M in THF), diphenyl (*E*)-2-(9-bromo-3-oxonon-7-en-1-yl)-2-(4-fluorobenzyl)malonate (58 mg, 0.10 mmol) and MeOH (340 µL, 8.4 mmol) to give the title compound as a white solid (13.8 mg, 0.043 mmol, 43%). ^{1}H NMR (400 MHz, $CDCl_3$) δ 1.10 (d, *J* = 7.1 Hz, 3 H, $CHCH_3$), 1.24 – 1.38 (m, 1 H, $CH_2CH_2CH_aH_bC$-O), 1.51 – 1.66 (m, 3 H, 2 H from $CH_2CH_2CH_2C$-O, 1 H from $CH_2CH_aH_bCH_2C$-O), 1.66 – 1.80 (m, 2 H, 1 H from $CH_2CH_aH_bCH_2C$-O, 1 H from $CH_aH_bCH_2C$=CH), 1.88 (dq, *J* = 10.1, 7.0 Hz, 1 H, $CHCH_3$), 1.94 – 2.06 (m, 2 H, 1 H from $CH_2CH_2CH_aH_bC$-O, 1 H from C*H*C-O), 2.15 – 2.24 (m, 1 H, $CH_aH_bCH_2C$=CH), 2.43 (dd, *J* = 13.8, 7.0 Hz, 1 H, $CH_2CH_aH_bC$=CH), 2.50 (d, *J* = 6.9 Hz, 1 H, CHO*H*), 2.60 (ddd, *J* = 13.7, 11.5, 8.0 Hz, 1 H, $CH_2CH_aH_bC$=CH), 4.16 (s, 1 H, OCO*H*), 4.24 (d, *J* = 6.8 Hz, 1 H, C*H*OH), 6.62 (s, 1 H, CH_2CH_2C=C*H*), 7.03 – 7.09 (m, 2 H, ArC*H*), 7.23 – 7.29 (m, 2 H, ArC*H*) ppm. ^{13}C NMR (100 MHz, $CDCl_3$) δ 12.5 ($CHCH_3$), 21.1 (CH_2CH_2C=CH), 23.1 ($CH_2CH_2CH_2C$-O), 32.1 ($CH_2CH_2CH_2C$-O), 39.6 (CH_2CH_2C=CH), 41.4 ($CH_2CH_2CH_2C$-O), 47.4 ($CHCH_3$), 55.2 (*C*HC-O), 79.4 (*C*HOH), 89.9 (*C*-O), 106.4 (O*C*O), 115.5 (d, *J* = 21.4 Hz, Ar*C*H), 130.3 (d, *J* = 8.0 Hz, Ar*C*H), 131.6 (CH_2CH_2C=*C*H), 132.8 (d, *J* = 3.3 Hz, Ar*C*), 138.84 (d, *J* = 1.3 Hz, CH_2CH_2C=CH), 162.10 (d, *J* = 247.3 Hz, Ar*C*) ppm. ^{19}F NMR (375 MHz, $CDCl_3$) δ -114.37 ppm. IR ν_{max} (neat/cm^{-1}): 3415, 2936, 2363, 1600, 1508, 1456, 1393, 1225, 1154, 1095, 1041, 1018, 953, 872, 829, 720, 679, 668, 649, 618, 589. M.p. ($CHCl_3$) = 102 – 103 ˚C. HRMS calcd for $C_{19}H_{22}FO_3$ $[M - H]^-$: 317.1553, found 317.1552.

***rac*-(3a*S*,7*R*,8*S*,9*R*,9a*R*)-6-((*E*)-4-Methoxybenzylidene)-9-methyloctahydro-3a,8-epoxycyclopenta[8]annulene-7,8(1H)-diol 7n**

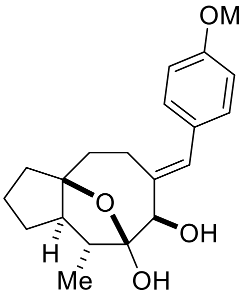

Prepared according to general procedure **I** using SmI_2 (7.00 mL, 0.70 mmol, 0.1 M in THF), diphenyl (*E*)-2-(9-bromo-3-oxonon-7-en-1-yl)-2-(4-methoxybenzyl)malonate (59 mg, 0.10 mmol) and MeOH (340 µL, 8.4 mmol) to give the title compound as an oil (12.9 mg, 0.039 mmol, 39%). ^{1}H NMR (400 MHz, $CDCl_3$) δ 1.09 (d, *J* = 7.1 Hz, 3 H, $CHCH_3$), 1.33 (td, *J* = 12.8, 12.4, 6.0 Hz, 1 H, $CH_2CH_2CH_aH_bC$-O), 1.48 – 1.56 (m, 2 H, $CH_2CH_2CH_2C$-O), 1.62 (dd, *J* = 6.1, 3.2 Hz, 1 H, $CH_2CH_aH_bCH_2C$-O), 1.67 – 1.79 (m, 2 H, 1 H from $CH_2CH_aH_bCH_2C$-O, 1 H from $CH_aH_bCH_2C$=CH), 1.88 (dq, *J* = 10.0, 7.1 Hz, 1 H, $CHCH_3$), 1.94 – 2.07 (m, 2 H, 1 H from $CH_2CH_2CH_aH_bC$-O, 1 H from CHC-O), 2.20 (ddd, *J* = 13.6, 7.8, 1.3 Hz, 1 H, $CH_aH_bCH_2C$=CH), 2.49 (q, *J* = 6.9, 6.4 Hz, 2 H, 1H from CHO*H*, 1 H from $CH_2CH_aH_bC$=CH), 2.59 (ddd, *J* = 13.9, 11.4, 7.8 Hz, 1 H, $CH_2CH_aH_bC$=CH), 3.82 (s, 3 H, O-CH_3), 4.23 (d, *J* = 7.6 Hz, 2 H, 1 H from OCO*H*, 1 H from C*H*OH), 6.59 (s, 1 H, CH_2CH_2C=C*H*), 6.87 – 6.93 (m, 2 H, ArC*H*), 7.20 – 7.29 (m, 2 H, ArC*H*). ^{13}C NMR (101 MHz, $CDCl_3$) δ 12.4 ($CHCH_3$), 21.1 (CH_2CH_2C=CH), 23.1 ($CH_2CH_2CH_2C$-O), 32.0 ($CH_2CH_2CH_2C$-O), 39.6 (CH_2CH_2C=CH), 41.4 ($CH_2CH_2CH_2C$-O), 47.3 ($CHCH_3$), 55.1 (*C*HC-O), 55.4 (O-CH_3), 79.6 (*C*HOH), 89.9 (*C*-O), 106.5 (O*C*O), 114.0 (Ar*C*H), 129.3 (Ar*C*H), 130.0 (Ar*C*), 132.2 (CH_2CH_2C=*C*H), 137.0 (CH_2CH_2C=CH), 159.0 (Ar*C*-OCH_3). IR ν_{max} (neat/cm^{-1}): 3411, 2935, 1749, 1606, 1190, 1154, 1059, 951, 869, 729, 647. HRMS calcd for $C_{20}H_{26}O_4Na$ $[M + Na]^+$: 353.1723, found 353.1716.

***rac*-(3a*R*,7*S*,8*R*,9*S*,9a*S*)-6-((*E*)-3,5-Dimethylbenzylidene)-9-methyloctahydro-3a,8-epoxycyclopenta[8]annulene-7,8(1*H*)-diol 7o**

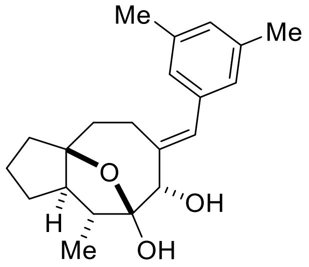

Prepared according to general procedure **I** using SmI_2 (7.00 mL, 0.70 mmol, 0.1 M in THF), diphenyl (*E*)-2-(9-bromo-3-oxonon-7-en-1-yl)-2-(3,5-dimethylbenzyl)malonate (59 mg, 0.10 mmol) and MeOH (340 µL, 8.4 mmol) to give the title compound as a white solid (11.4 mg, 0.035 mmol, 35%). ^{1}H NMR (400 MHz, $CDCl_3$) δ 1.09 (d, *J* = 7.3 Hz, 3 H, $CHCH_3$), 1.25 – 1.35 (m, 1 H, $CH_2CH_2CH_aH_bC$-O), 1.43 – 1.58 (m, 3 H, 1 H from $CH_2CH_aH_bCH_2C$-O, 2 H from $CH_2CH_2CH_2C$-O), 1.59 – 1.74 (m, 2 H, 1 H from $CH_aH_bCH_2C$=CH, 1 H from $CH_2CH_aH_bCH_2C$-O), 1.88 (dq, *J* = 10.1, 7.2 Hz, 1 H, $CHCH_3$), 1.90 – 1.99 (m, 2 H, 1 H from $CH_2CH_2CH_aH_bC$-O, 1 H from CHC-O), 2.08 – 2.16 (m, 1 H, $CH_aH_bCH_2C$=CH), 2.33 (s, 6 H, 2x ArC-CH_3), 2.39 – 2.55 (m, 2 H, CH_2CH_2C=CH), 4.17 – 4.26 (m, 2 H, 1 H from OCO*H*, 1 H from C*H*OH), 6.60 (s, 1 H, CH_2CH_2C=C*H*), 6.91 (s, 2 H, ArC*H*), 6.93 (s, 1 H, ArC*H*) ppm. ^{13}C NMR (100 MHz, $CDCl_3$) δ 12.3 ($CHCH_3$), 21.2 (CH_2CH_2C=CH), 21.6 (ArC-CH_3), 23.1 ($CH_2CH_2CH_2C$-O), 32.1 ($CH_2CH_2CH_2C$-O), 39.7 (CH_2CH_2C=CH), 41.4 ($CH_2CH_2CH_2C$-O), 47.3 ($CHCH_3$), 55.1 (*C*HC-O), 79.6 (*C*HOH), 89.9 (*C*-O), 106.5 (O*C*O), 126.4 (Ar*C*H), 129.2 (Ar*C*H), 133.0 (CH_2CH_2C=*C*H), 136.7 (Ar*C*), 138.0 (Ar*C*), 138.3 (CH_2CH_2C=CH) ppm. IR ν_{max} (neat/cm^{-1}): 3445, 2924, 2853, 1749, 1598, 1493, 1455, 1393, 1258, 1195, 1157, 1098, 1048, 967, 948, 927, 903, 845, 821, 742, 720, 679, 668, 649. M.p. ($CHCl_3$) = 106 – 107 ˚C. HRMS calcd for $C_{21}H_{28}O_3K$ $[M + K]^+$: 367.1675, found 367.1672.

***rac*-(3a*S*,7*R*,8*S*,9*R*,9a*R*,*E*)-9-Methyl-6-(thiophen-3-ylmethylene)octahydro-3a,8-epoxycyclopenta[8]annulene-7,8(1H)-diol 7p**

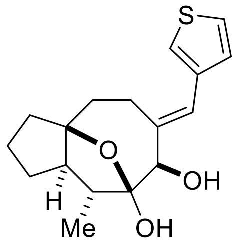

Prepared according to general procedure **I** using SmI_2 (7.00 mL, 0.70 mmol, 0.1 M in THF), diphenyl (*E*)-2-(9-bromo-3-oxononon-7-en-1-yl)-2-(thiophen-3-ylmethyl)malonate (57 mg, 0.10 mmol) and MeOH (340 µL, 8.4 mmol) to give the title compound as an oil (10.9 mg, 0.036 mmol, 36%). ^{1}H NMR (400 MHz, $CDCl_3$) δ 1.08 (d, *J* = 6.9 Hz, 3 H, $CHCH_3$), 1.32 (td, *J* = 13.3, 6.6 Hz, 2 H, $CH_2CH_2CH_2$C-O), 1.44 – 1.54 (m, 2 H, $CH_2CH_2CH_2$C-O), 1.65 – 1.79 (m, 2 H, $CH_2CH_2CH_2$C-O), 1.81 – 1.96 (m, 2 H, 1 H from $CH_aH_bCH_2$C=CH, 1 H from $CHCH_3$), 2.01 (dd, *J* = 13.6, 6.1 Hz, 1 H, C*H*C-O), 2.18 (ddd, *J* = 13.5, 7.5, 1.6 Hz, 1 H, $CH_aH_bCH_2$C=CH), 2.49 – 2.73 (m, 3 H, CHO*H*, CH_2CH_2C=CH), 4.17 – 4.28 (m, 2 H, C*H*OH, OCO*H*), 6.59 (s, 1 H, CH_2CH_2C=C*H*), 7.12 (dd, *J* = 5.0, 1.3 Hz, 1 H, ArC*H*), 7.23 (d, *J* = 2.9 Hz, 1 H, ArC*H*), 7.33 (dd, *J* = 5.0, 2.9 Hz, 1 H, ArC*H*). ^{13}C NMR (101 MHz, $CDCl_3$) δ 12.5 ($CHCH_3$), 21.5 (CH_2CH_2C=CH), 23.1 ($CH_2CH_2CH_2$C-O), 32.0 ($CH_2CH_2CH_2$C-O), 39.0 (CH_2CH_2C=CH), 41.4 ($CH_2CH_2CH_2$C-O), 47.4 ($CHCH_3$), 55.3 (*C*HC-O), 79.2 (*C*HOH), 89.9 (*C*-O), 106.5 (O*C*O), 123.5 (Ar*C*H), 125.8 (Ar*C*H), 126.4 (CH_2CH_2C=*C*H), 128.5 (Ar*C*H), 137.8 (Ar*C*), 138.0 (CH_2CH_2*C*=CH). IR ν_{max} (neat/cm^{-1}): 3439, 2927, 1738, 1455, 1392, 1258, 1147, 906, 729, 647, 592. HRMS calcd for $C_{17}H_{22}O_3SNa$ [M + Na]$^+$: 329.1182, found 329.1179.

***rac*-(3a*R*,7*S*,8*R*,9*S*,9a*S*)-6-((*E*)-Benzylidene)-9-(3-((*tert*-butyldimethylsilyl)oxy)propyl) octahydro-3a,8-epoxycyclopenta[8]annulene-7,8(1*H*)-diol 7q**

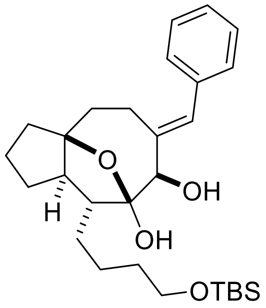

Prepared according to general procedure **I** using SmI_2 (12.0 mL, 1.20 mmol, 0.1 M in THF), diphenyl (*E*)-2-benzyl-2-(12 -((*tert*-butyldimethylsilyl)oxy)-3 -oxo-9 -((4 -(trifluoromethyl) benzoyl)oxy)dodec-7-en-1-yl)malonate (84 mg, 0.10 mmol) and MeOH (340 µL, 8.4 mmol) to give the title compound as a colourless oil (15.9 mg, 0.035 mmol, 34%). ^{1}H NMR (400 MHz, $CDCl_3$) δ 0.02 (s, 3 H, Si-C*H*$_3$), 0.03 (s, 3 H, Si-C*H*$_3$), 0.87 (s, 9 H, 3 × Si-C(C*H*$_3$)), 1.31 – 1.38 (m, 1 H, CH_2CH_2C*H*$_a$H$_b$C-O), 1.38 – 1.69 (m, 9 H, C*H*$_2$$CH_2CH_2CH_2$O-Si, CH_2C*H*$_2$$CH_2CH_2$O-Si, CH_2CH_2C*H*$_2$$CH_2$O-Si, C*H*$_2$$CH_2CH_2$C-O, 1 H from CH_2C*H*$_aH_b$$CH_2$C-O), 1.70 – 1.85 (m, 3 H, C*H*COO, 1 H from C*H*$_a$H$_b$$CH_2$C=CH, 1 H from CH_2CH$_a$*H*$_b$$CH_2$C-O), 1.97 – 2.05 (m, 2 H, 1 H from CH_2CH_2CH$_a$*H*$_b$C-O, C*H*C-O), 2.16 – 2.23 (m, 1 H from CH$_a$*H*$_b$$CH_2$C=CH), 2.44 – 2.51 (m, 2 H, 1 H from CH_2C*H*$_a$H$_b$C=CH, 1 H from CHO*H*), 2.54 – 2.62 (m, 1 H, CH_2CH$_a$*H*$_b$C=CH), 3.59 (t, *J* = 6.3 Hz, 2 H, $CH_2CH_2CH_2$C*H*$_2$O-Si), 4.17 (s, 1 H, OCO*H*), 4.24 (d, *J* = 6.9 Hz, 1 H, C*H*OH), 6.66 (s, 1 H, CH_2CH_2C=C*H*), 7.28 – 7.31 (m, 3 H, ArC*H*), 7.35 – 7.40 (m, 2 H, ArC*H*) ppm. ^{13}C NMR (100 MHz, $CDCl_3$) δ -5.1 (Si-*C*H$_3$), -5.1 (Si*C*H$_3$), 18.5 (Si-*C*(CH$_3$)$_3$), 21.1 (CH_2*C*H$_2$C=CH), 23.1 (CH_2*C*H$_2$$CH_2$C-O), 24.7 ($CH_2$*C*H$_2$$CH_2CH_2$O-Si), 26.1 (Si-C(*C*H$_3$)$_3$), 28.8 (*C*H$_2$$CH_2CH_2$C-O), 33.3 ($CH_2CH_2$*C*H$_2$$CH_2$O-Si), 33.3 (*C*H$_2$$CH_2CH_2CH_2$O-Si), 39.9 (*C*H$_2$$CH_2$C=CH), 41.6 ($CH_2CH_2$*C*H$_2$C-O), 52.3 (*C*HCOO), 54.6 (*C*HC-O), 63.1 ($CH_2CH_2CH_2$*C*H$_2$O-Si), 79.6 (*C*HOH), 90.1 (*C*-O), 106.5 (O*C*O), 127.5 (Ar*C*H), 128.6 (Ar*C*H), 128.7 (Ar*C*H), 133.0 (CH_2CH_2C=*C*H), 136.8 (Ar*C*), 139.0 (*C*=CH) ppm. IR ν_{max} (neat/cm^{-1}): 2927, 2857, 2361, 1253, 1101, 835, 720, 679, 668, 649. HRMS calcd for $C_{28}H_{44}O_4SiNa$ [M + Na]$^+$: 495.2907, found 495.2903.

***rac*-(3a*R*,6*R*,8*S*,8a*S*)-8-(Methyl-d)-6-phenylhexahydro-1*H*-3a,6-(epoxymethano)azulene-7,9(4*H*)-dione D_2-7a**

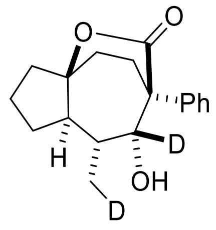

Prepared according to general procedure **H** using SmI_2 (9.00 mL, 0.90 mmol, 0.1 M in THF), diphenyl (*E*)-2-(9-bromo-3-oxonon-7-en-1-yl)-2-phenylmalonate (54.8 mg, 0.10 mmol) and D_2O (1.80 mL, 90.0 mmol) to give the title compound as a white solid (7.2 mg, 0.025 mmol, 25%). ^{1}H NMR (400 MHz, $CDCl_3$) δ 1.10 (dt, *J* = 6.7, 1.8 Hz, 2 H, CHCH_2D), 1.45 – 1.65 (m, 2 H, 1 H from CH_aH$_b$CH$_2$CH$_2$C-O, 1 H from CH$_2$CH_aH$_b$CH$_2$C-O), 1.71 – 1.82 (m, 1 H, C*H*CH$_2$D), 1.83 – 1.95 (m, 3 H, 1 H from CH$_2$CH$_a$$H_bCH_2$C-O, 1 H from C*H*C-O, 1 H from CH$_2$CH$_2$CH_aH$_b$C-O), 1.96 – 2.09 (m, 3 H, 1 H from CH$_a$$H_bCH_2CH_2$C-O, 1 H from CH$_2CH_aH_b$C, 1 H from C$H_aH_bCH_2$C), 2.13 – 2.31 (m, 2 H, 1 H from CH$_2$CH$_2$CH$_a$$H_b$C-O, 1 H from CH$_a$$H_bCH_2$C), 2.78 – 2.89 (m, 1 H, CH$_2$CH$_a$$H_b$C), 7.25 – 7.30 (m, 1 H, ArC*H*), 7.34 – 7.43 (m, 2 H, ArC*H*), 7.47 – 7.50 (m, 2 H, ArC*H*) ppm. ^{13}C NMR (100 MHz, $CDCl_3$) δ 22.9 (CH$_2$*C*H$_2$CH$_2$C-O), 24.5 (CH$_2$*C*H$_2$C), 32.1 (*C*H$_2$CH$_2$C), 32.6 (*C*H$_2$CH$_2$CH$_2$C-O), 38.9 (*C*HCH$_2$D), 41.9 (CH$_2$CH$_2$*C*H$_2$C-O), 49.1 (*C*HC-O), 55.0 (CH$_2$CH$_2$*C*), 90.7 (CH*C*-O), 126.9 (Ar*C*H), 128.9 (Ar*C*H), 128.1 (Ar*C*H), 143.2 (Ar*C*), 174.4 (*C*(O)O) ppm. Signals for CH*C*H$_2$D and *C*DOH are not visible. IR ν_{max} (neat/cm^{-1}): 2929, 1817, 1713, 1496, 1445, 1339, 1262, 1195, 1141, 1069, 1022, 960, 756, 699. M.p. ($CHCl_3$) = 82 – 84 ˚C. HRMS calcd for $C_{18}H_{20}D_2O_3Na$ $[M + Na]^+$: 311.1587, found 311.1587.

***rac*-Phenyl (3a*R*,6*R*,7*R*,8*S*,8a*S*)-7-hydroxy-8-methyl-6-(propan-2-yl-2-d)octahydro-1*H*-3a,7-epoxyazulene-6-carboxylate 7p and *rac*-(3a*R*,6*R*,7*S*,8*S*,8a*S*)-7-Hydroxy-8-methyl-6-(propan-2-yl-2-d)octahydro-1*H*-3a,6-(epoxymethano)azulen-9-one 7q**

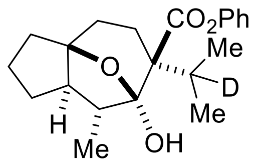

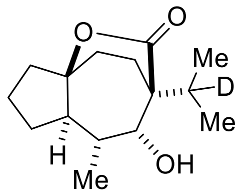

7p **7q**

Prepared according to general procedure **H** using SmI_2 (7.00 mL, 0.70 mmol, 0.1 M in THF), diphenyl (*E*)-2-(9-bromo-3-oxononon-7-en-1-yl)-2-(propan-2-yl-2-*d*)malonate (51.5 mg, 0.10 mmol) and H_2O (1.26 mL, 70.0 mmol) to give the title compound **7p** as a colourless oil (5.9 mg, 0.017 mmol, 17%) and **7q** as a white solid (3.0 mg, 0.012 mmol, 12%).

Spectroscopic data for **7p**. ^{1}H NMR (400 MHz, $CDCl_3$) δ 1.01 (d, J = 7.5 Hz, 3 H, $CHCH_3$), 1.08 (s, 3 H, $(CD(CH_3)_a(CH_3)_b)$, 1.14 (s, 3 H, $(CD(CH_3)_a(CH_3)_b)$, 1.46 – 1.55 (m, 2 H, 1 H from $CH_2CH_2CH_aH_bC$-O, 1 H from $CH_aH_bCH_2C$), 1.57 – 1.70 (m, 3 H, 2 H from $CH_2CH_2CH_2C$-O, 1 H from $CH_2CH_aH_bCH_2C$-O), 1.72 – 1.81 (m, 1 H, $CH_2CH_aH_bC$), 1.83 – 1.97 (m, 2 H, 1 H from $CH_2CH_aH_bCH_2C$-O, 1 H from $CH_2CH_2CH_aH_bC$-O), 2.00 – 2.12 (m, 1 H, $CH_aH_bCH_2C$), 2.22 – 2.33 (m, 1 H, CHC-O), 2.52 – 2.66 (m, 2 H, 1 H from $CH_2CH_aH_bC$, 1 H from $CHCH_3$), 5.99 (s, 1 H, OCO*H*), 7.06 – 7.16 (m, 2 H, ArC*H*), 7.22 – 7.30 (m, 1 H, ArC*H*), 7.35 – 7.46 (m, 2 H, ArC*H*) ppm. ^{13}C NMR (100 MHz, $CDCl_3$) δ 12.2 ($CHCH_3$), 18.9 ($CD(CH_3)_a(CH_3)_b$), 20.2 ($CD(CH_3)_a(CH_3)_b$), 26.2 ($CH_2CH_2CH_2C$-O), 27.1 (CH_2CH_2C), 27.2 ($CH_2CH_2CH_2C$-O), 32.5 (CH_2CH_2C), 34.0 (1:1:1 t, J = 20.1 Hz, $CD(CH_3)_2$), 36.8 ($CH_2CH_2CH_2C$-O), 38.8 ($CHCH_3$), 50.6 (CHC-O), 57.4 (CH_2CH_2C), 91.3 ($CH_2CH_2CH_2C$-O), 108.7 (O*C*OH), 121.7 (Ar*C*H), 126.3 (Ar*C*H), 129.5 (Ar*C*H), 150.4 (Ar*C*), 175.2 (*C*(O)O) ppm. IR ν_{max} (neat/cm^{-1}): 3444, 2958, 1714, 1492, 1456, 1277, 1188, 1161, 1103, 1059, 946, 739, 689. HRMS calcd for $C_{21}H_{27}DO_4Na$ [M + Na]$^+$: 368.1943, found 368.1946.

Spectroscopic data for **7q.** ^{1}H NMR (400 MHz, $CDCl_3$) δ 0.94 (s, 3 H, $(CD(CH_3)_a(CH_3)_b)$), 0.99 (s, 3 H, $(CD(CH_3)_a(CH_3)_b)$, 1.04 (d, J = 6.7 Hz, 3 H, $CHCH_3$), 1.40 – 1.57 (m, 4 H, 1 H from $CHCH_3$, 1 H from $CH_2CH_aH_bC$, 1 H from $CH_aH_bCH_2CH_2C$-O, 1 H from $CH_2CH_aH_bCH_2C$-O), 1.70 – 1.92 (m, 4 H, 1 H from CHC-O, 1 H from $CH_2CH_aH_bCH_2C$-O, 1 H from $CH_2CH_2CH_aH_bC$-O, 1 H from $CH_aH_bCH_2C$), 1.93 – 2.03 (m, 1 H, $CH_aH_bCH_2CH_2C$-O), 2.04 – 2.14 (m, 2 H, 1 H from $CH_aH_bCH_2C$, 1 H from $CH_2CH_2CH_aH_bC$-O), 2.14 – 2.24 (m, 1 H, $CH_2CH_aH_bC$), 3.69 (dd, J = 4.3, 2.7 Hz, 1 H, C*H*OH) ppm. ^{13}C NMR (100 MHz,

$CDCl_3$) δ 16.1 ($CH_2\mathit{C}H_2C$), 17.3 ($CH\mathit{C}H_3$), 17.3 ($CD(\mathit{C}H_3)_a(CH_3)_b$), 19.5 ($CD(CH_3)_a(\mathit{C}H_3)_b$), 22.6 ($CH_2\mathit{C}H_2CH_2C$-O), 31.0 ($\mathit{C}H_2CH_2C$), 32.3 ($\mathit{C}H_2CH_2CH_2C$-O), 39.5 ($\mathit{C}HCH_3$), 41.7 ($CH_2CH_2\mathit{C}H_2C$-O), 50.1 (*C*HC-O), 53.2 ($CH_2CH_2\mathit{C}$), 76.1 (*C*HOH), 89.8 (CH*C*-O), 176.1 (*C*(O)O) ppm. Signal for $\mathit{C}D(CH_3)_2$ not visible. IR ν_{max} (neat/cm^{-1}): 3444, 2958, 2875, 1708, 1456, 1390, 1371, 1345, 1226, 1189, 1175, 1078, 1032, 965. M.p. ($CHCl_3$) = 60 – 62 ˚C. HRMS calcd for $C_{15}H_{23}DO_3$ $[M - H]^-$: 252.1715, found 252.1713.

Supplementary Figure 1. ^{1}H NMR (400 MHz, $CDCl_3$) spectrum of **S1**

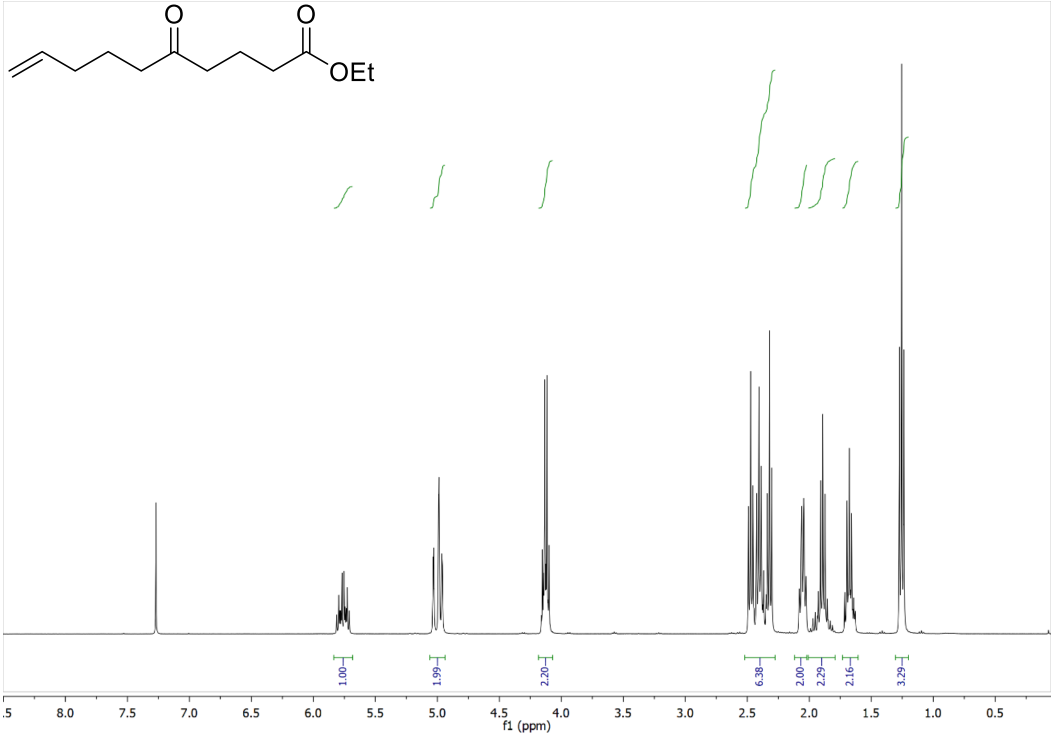

Supplementary Figure 2 . ^{13}C NMR (100 MHz, $CDCl_3$) spectrum of **S1**

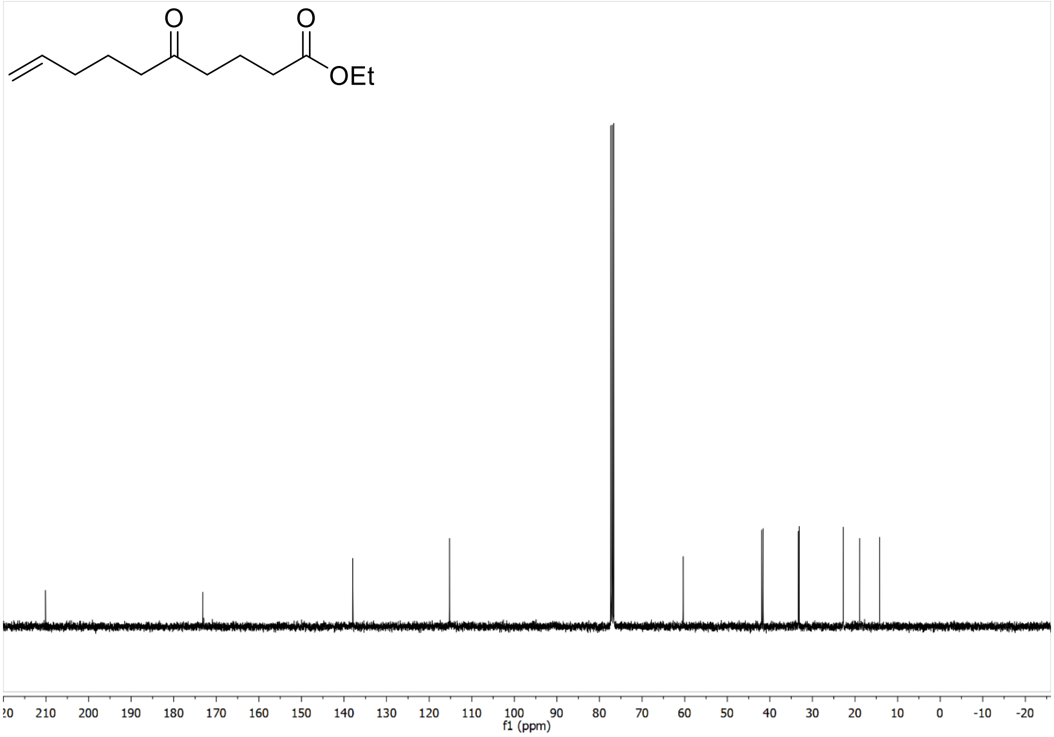

Supplementary Figure 3. ^{1}H NMR (500 MHz, $CDCl_3$) spectrum of **S2**

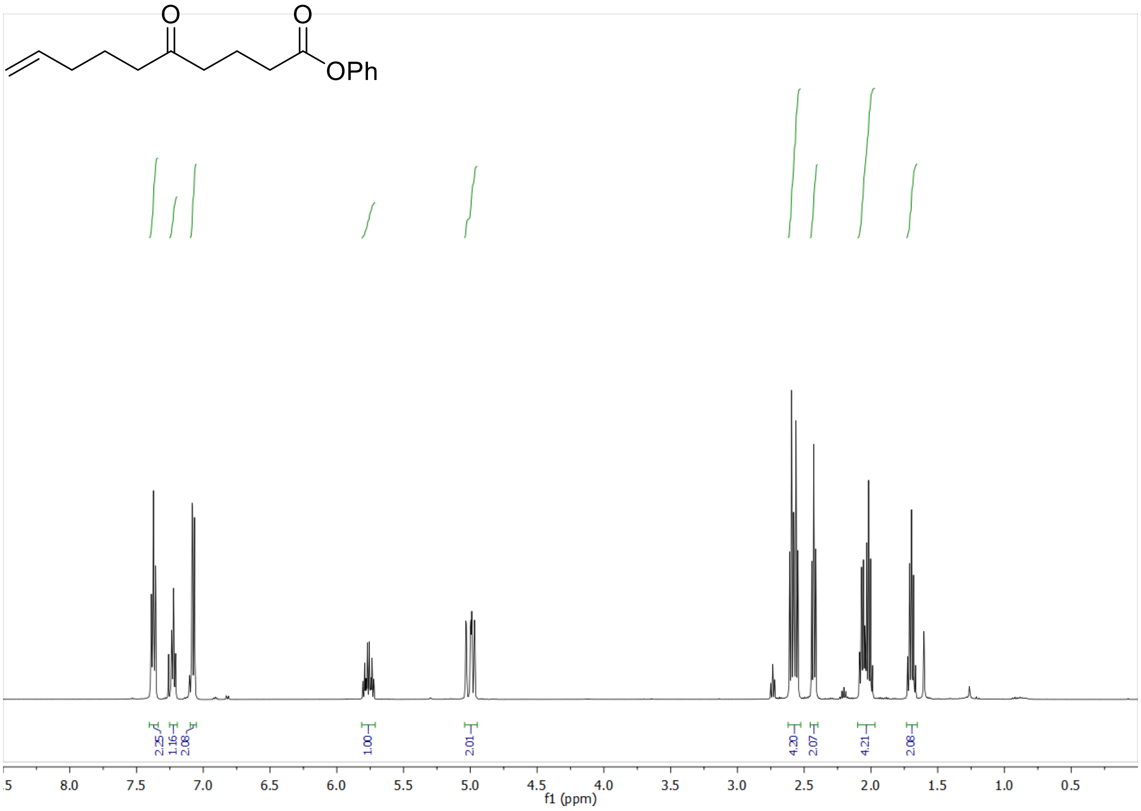

Supplementary Figure 4. ^{13}C NMR (125 MHz, $CDCl_3$) spectrum of **S2**

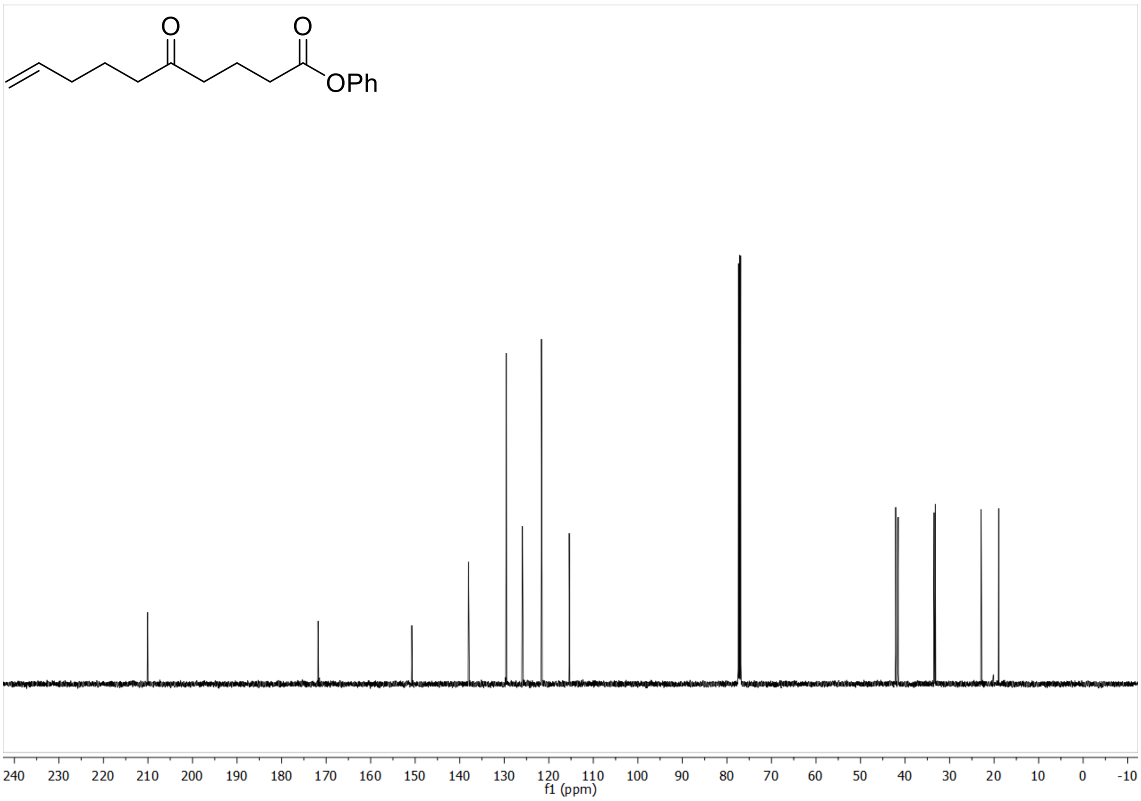

Supplementary Figure 5. ^{1}H NMR (400 MHz, $CDCl_3$) spectrum of **S4**

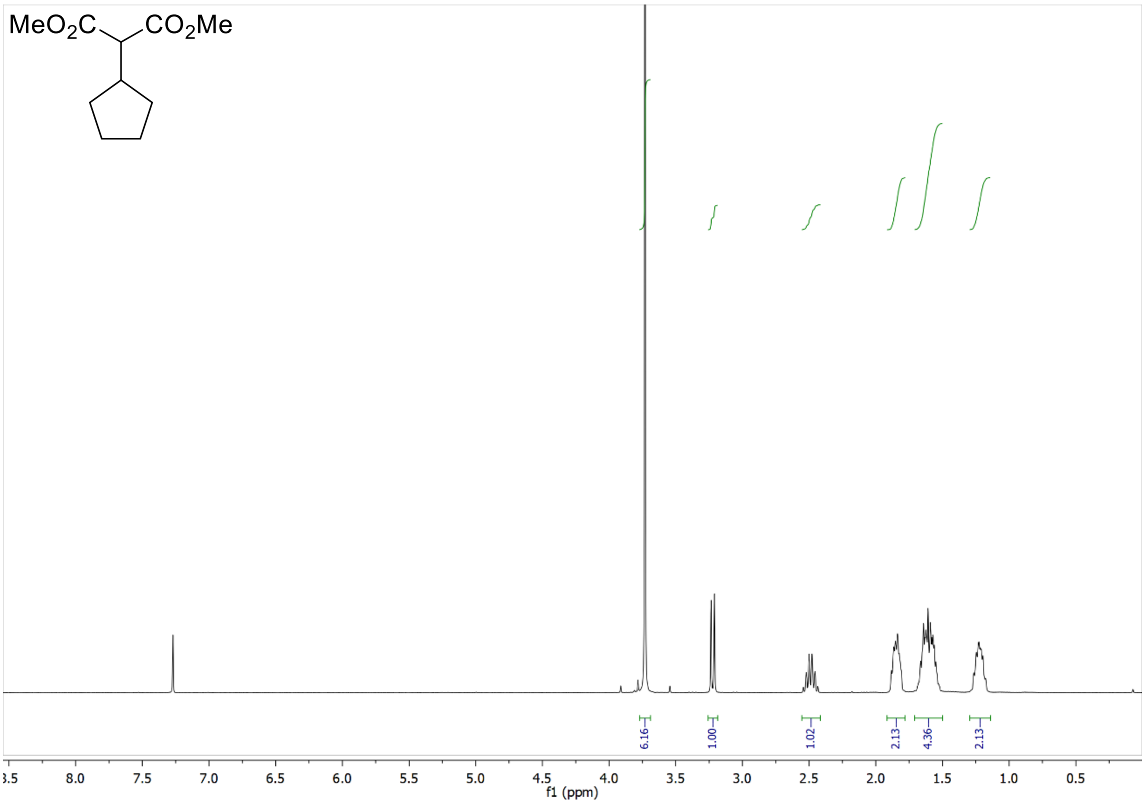

Supplementary Figure 6. ^{13}C NMR (100 MHz, $CDCl_3$) spectrum of **S4**

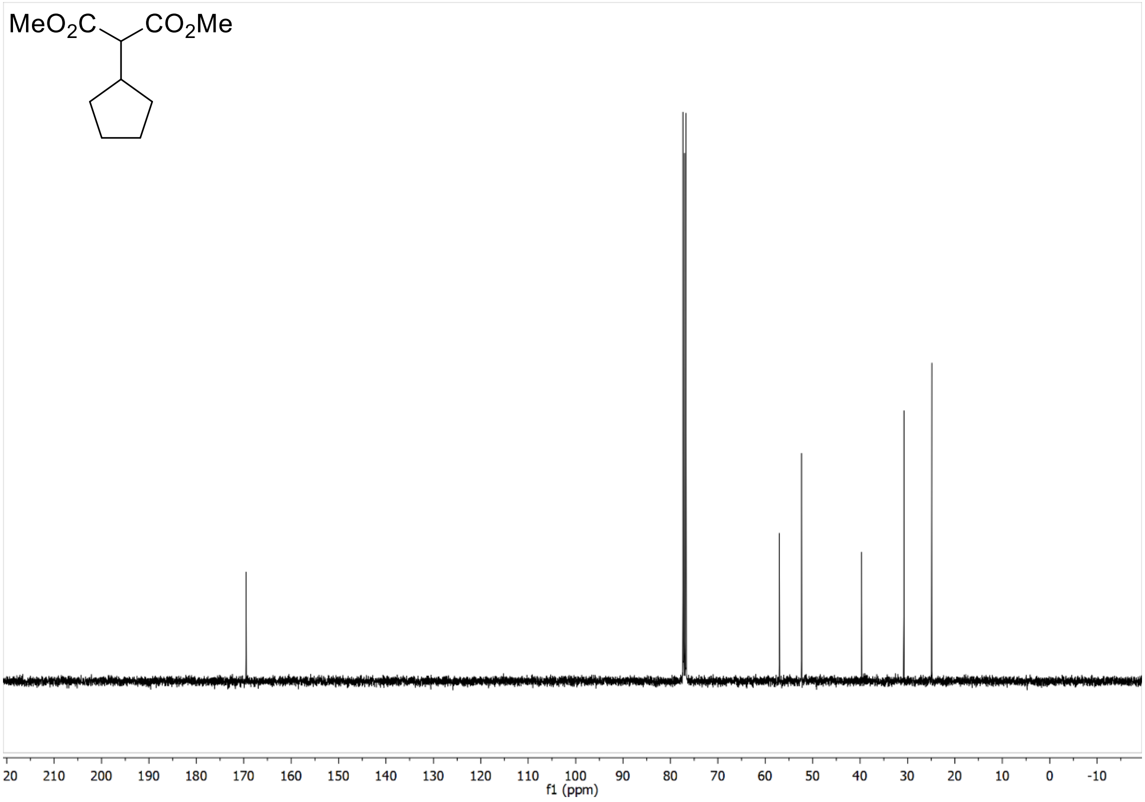

Supplementary Figure 7. ^{1}H NMR (400 MHz, $CDCl_3$) spectrum of **S5**

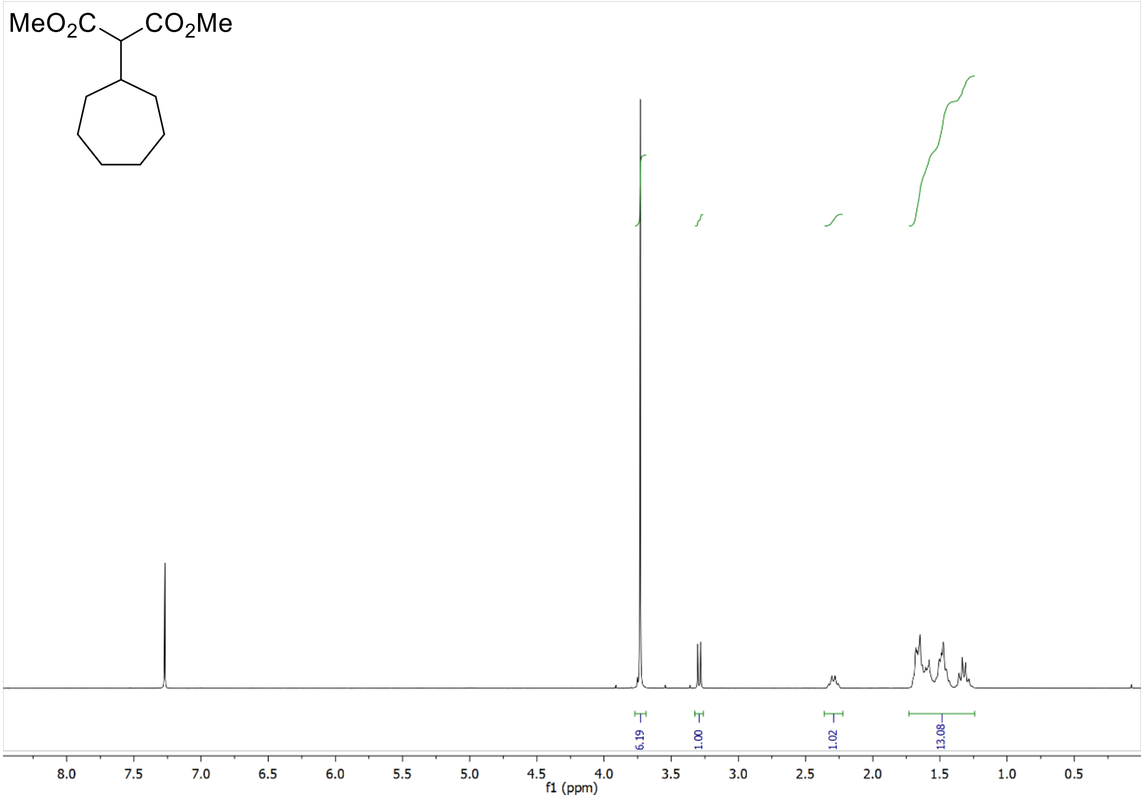

Supplementary Figure 8. ^{13}C NMR (100 MHz, $CDCl_3$) spectrum of **S5**

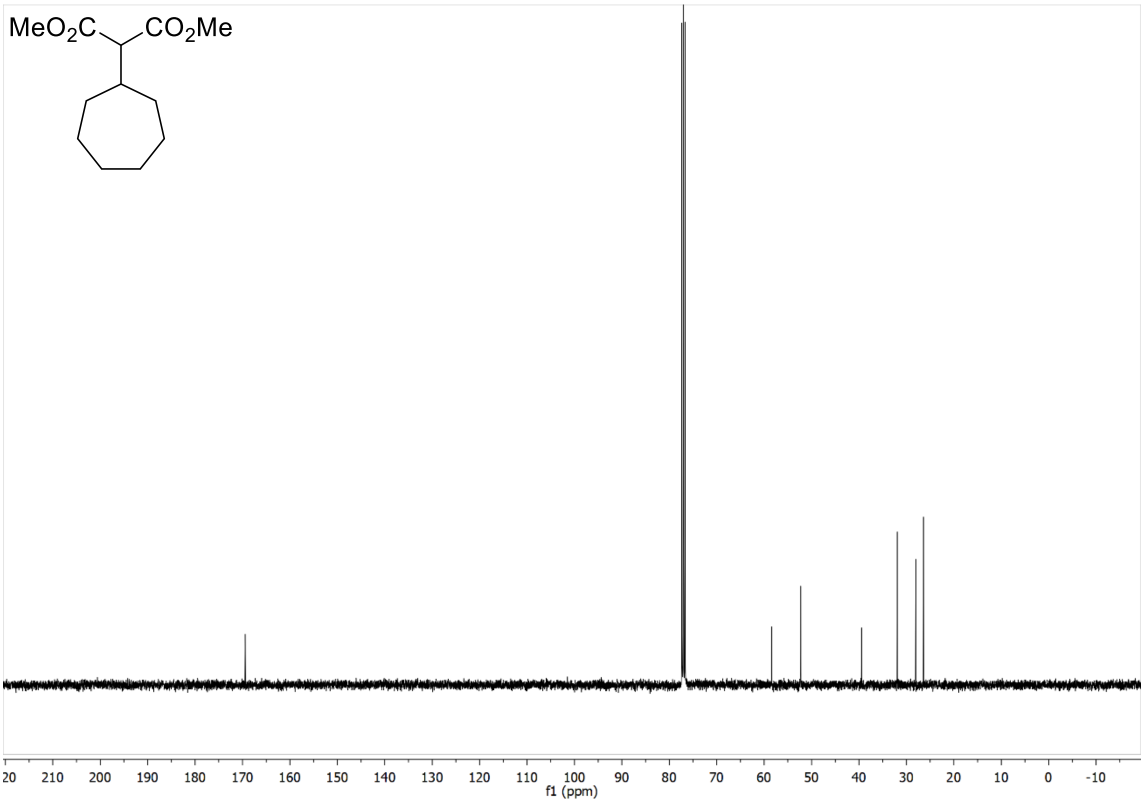

Supplementary Figure 9. ^{1}H NMR (400 MHz, $CDCl_3$) spectrum of **S6**

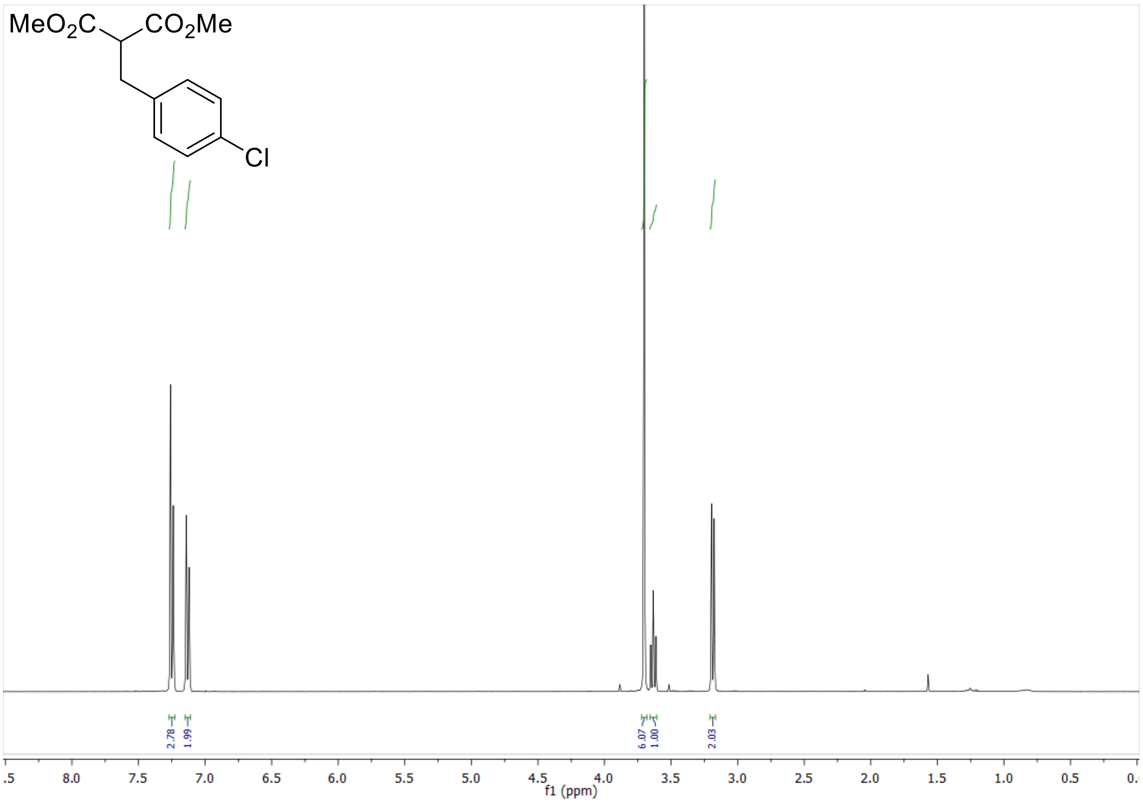

Supplementary Figure 10. ^{13}C NMR (100 MHz, $CDCl_3$) spectrum of **S6**

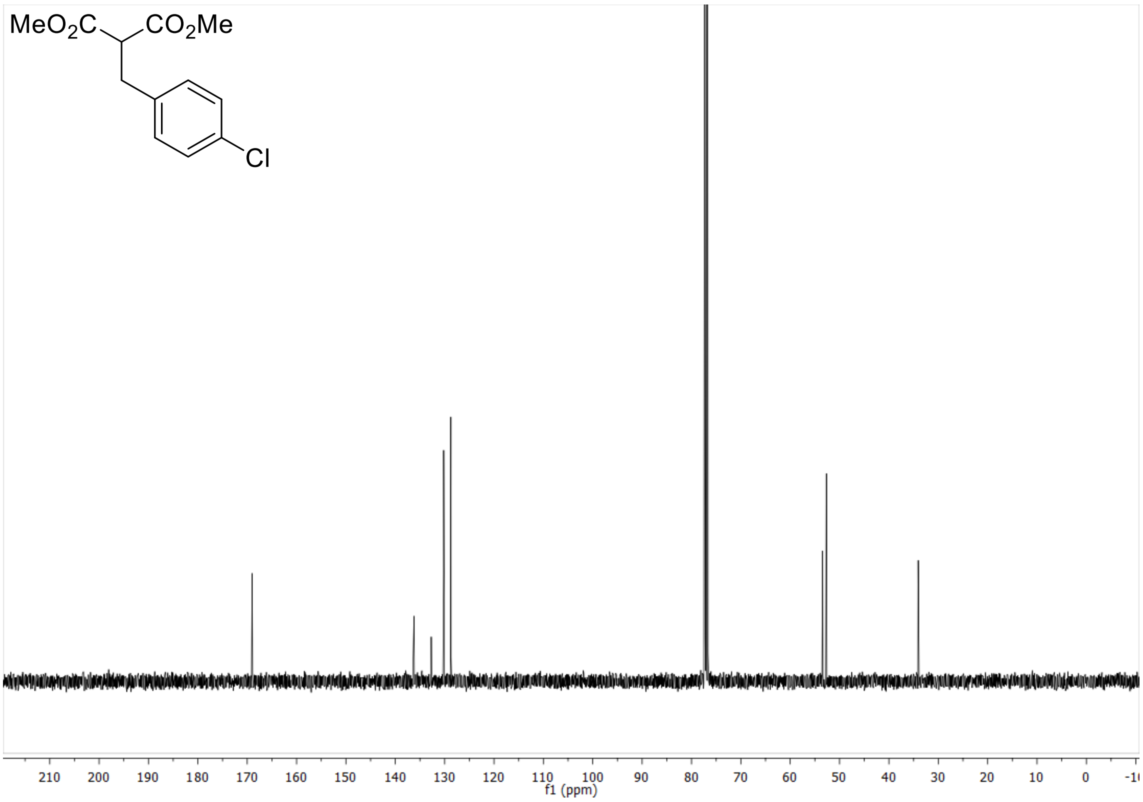

Supplementary Figure 11. ^{1}H NMR (400 MHz, $CDCl_3$) spectrum of **S7**

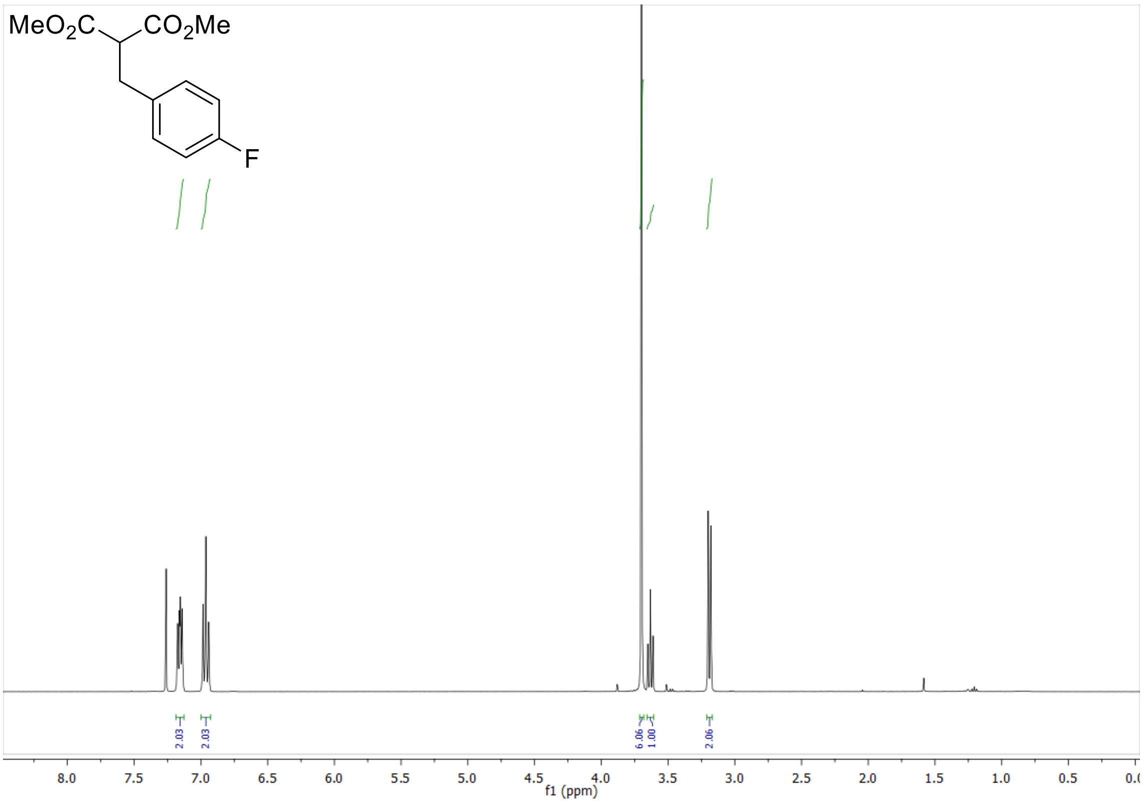

Supplementary Figure 12. ^{13}C NMR (100 MHz, $CDCl_3$) spectrum of **S7**

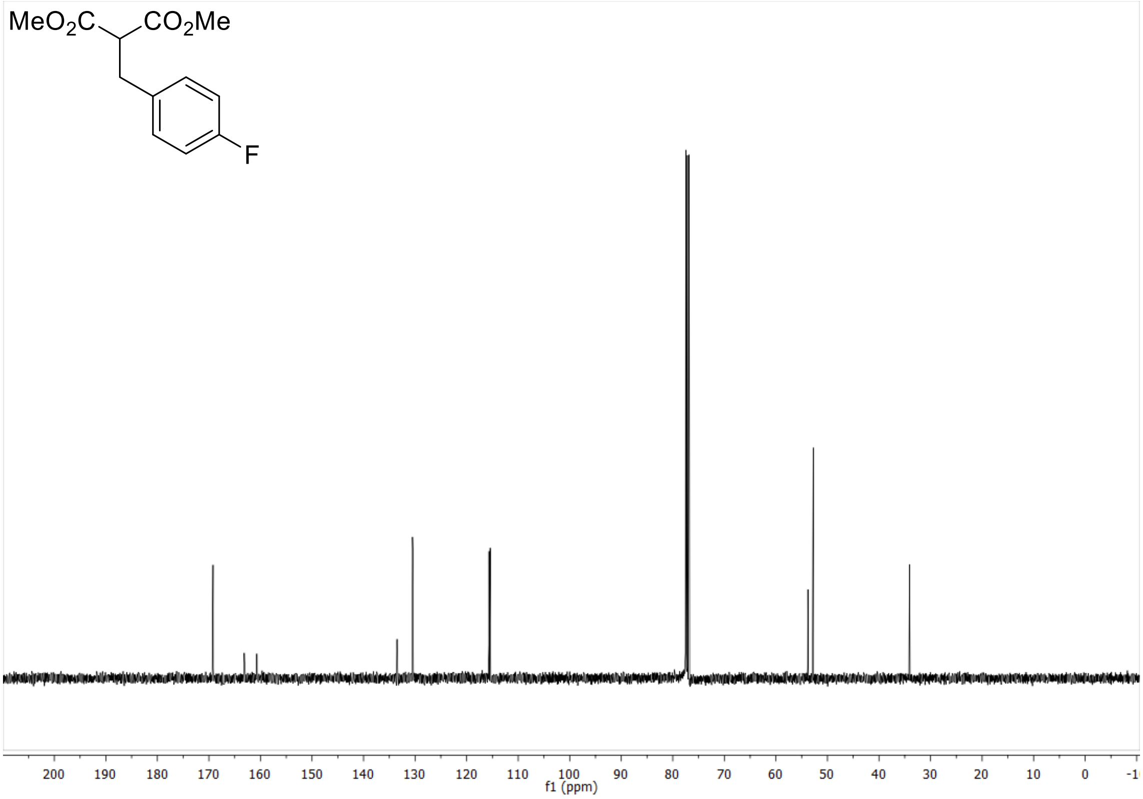

Supplementary Figure 13. ^{1}H NMR (500 MHz, $CDCl_3$) spectrum of **S8**

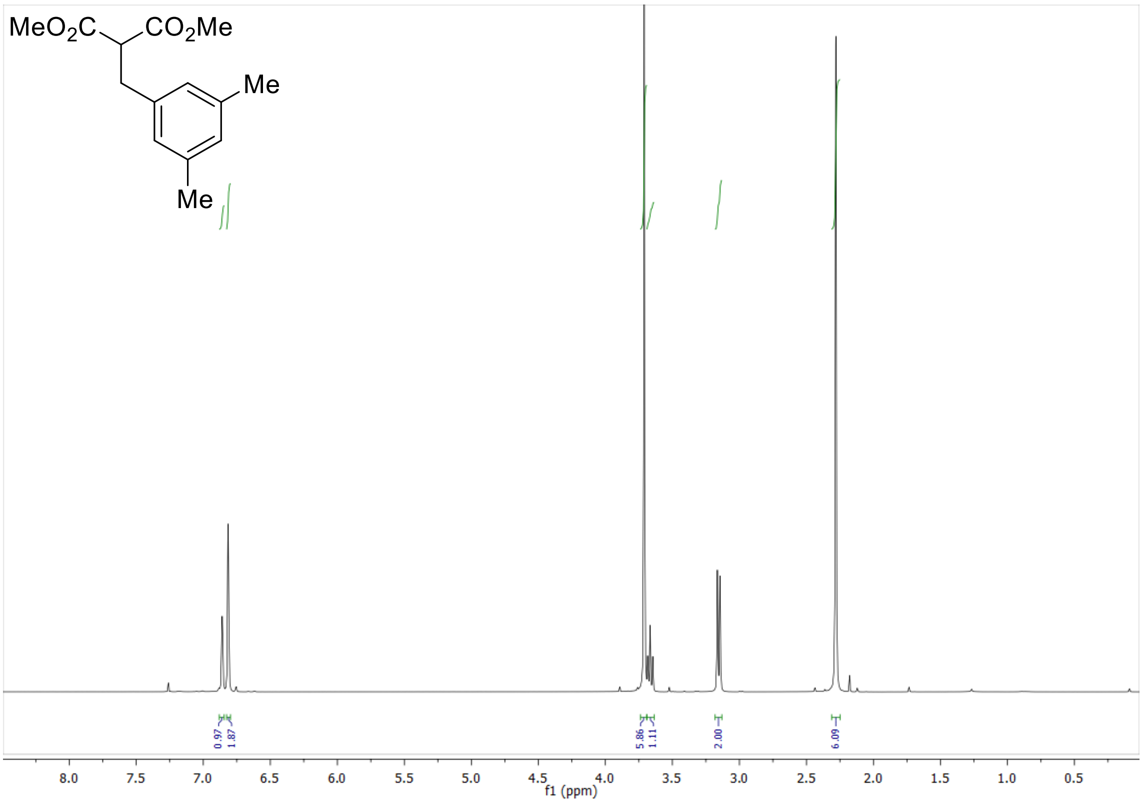

Supplementary Figure 14. ^{13}C NMR (126 MHz, $CDCl_3$) spectrum of **S8**

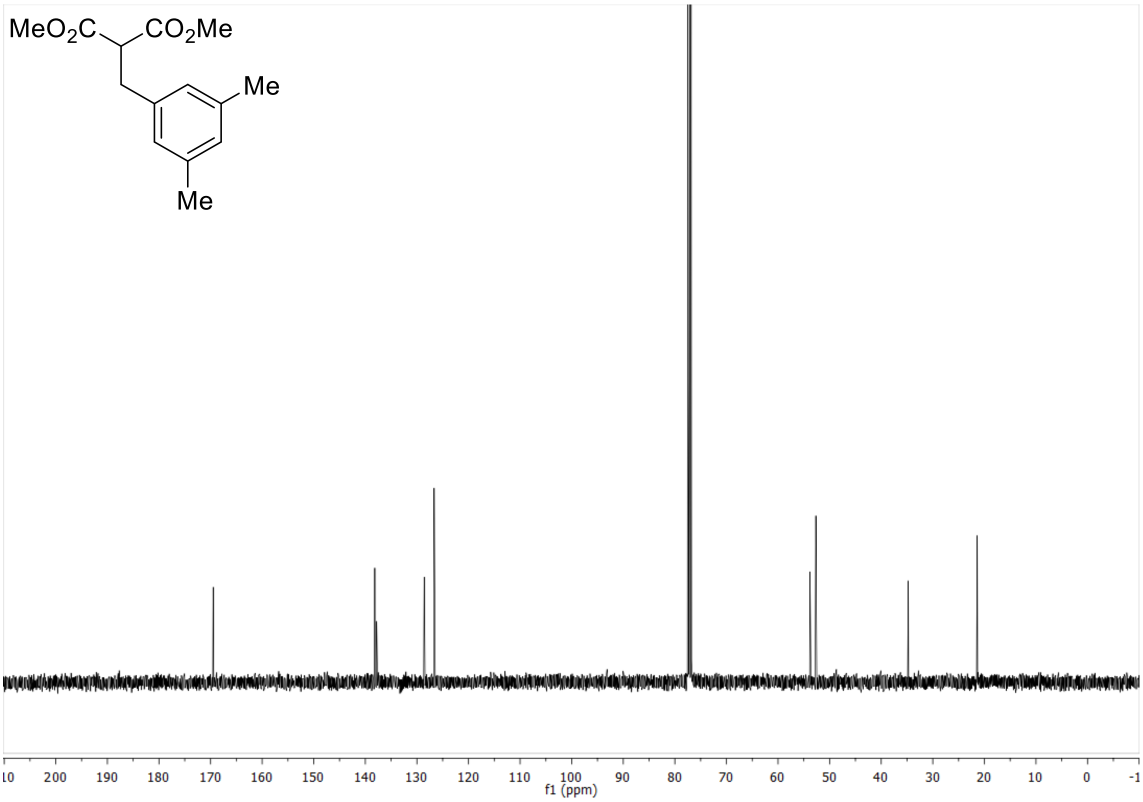

Supplementary Figure 15. ^1H NMR (400 MHz, $CDCl_3$) spectrum of **S9**

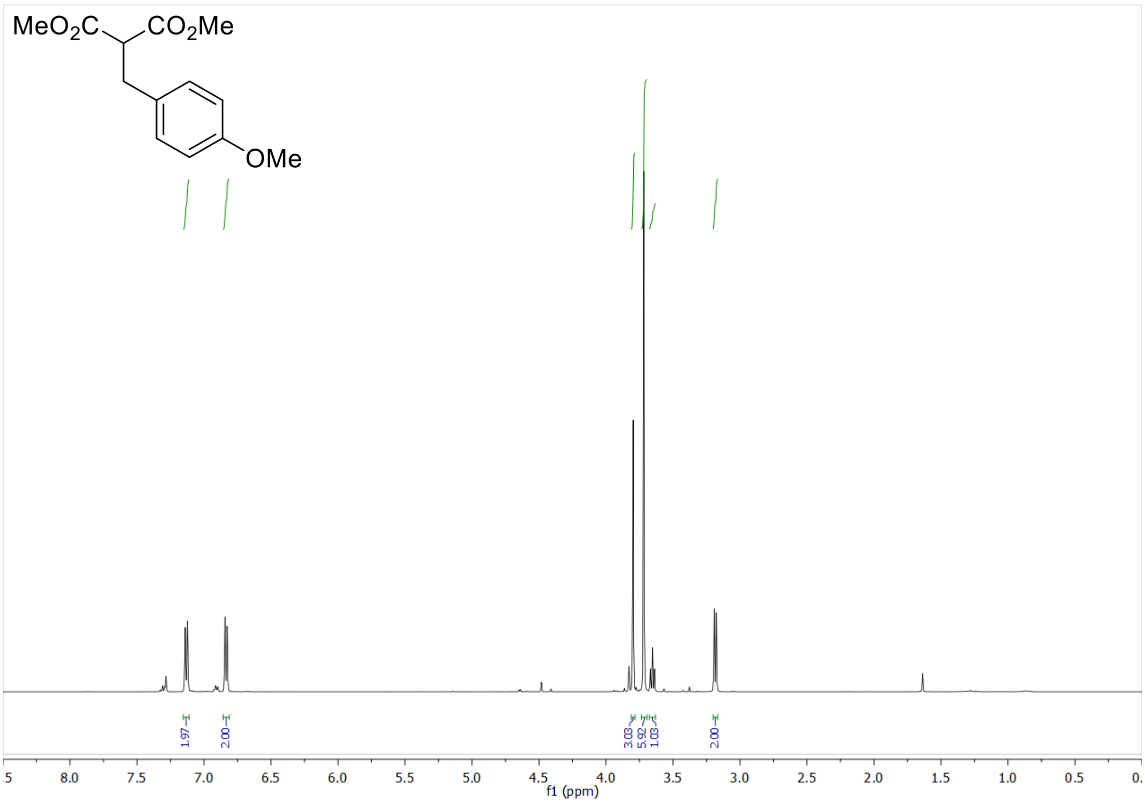

Supplementary Figure 16. ^{13}C NMR (100 MHz, $CDCl_3$) spectrum of **S9**

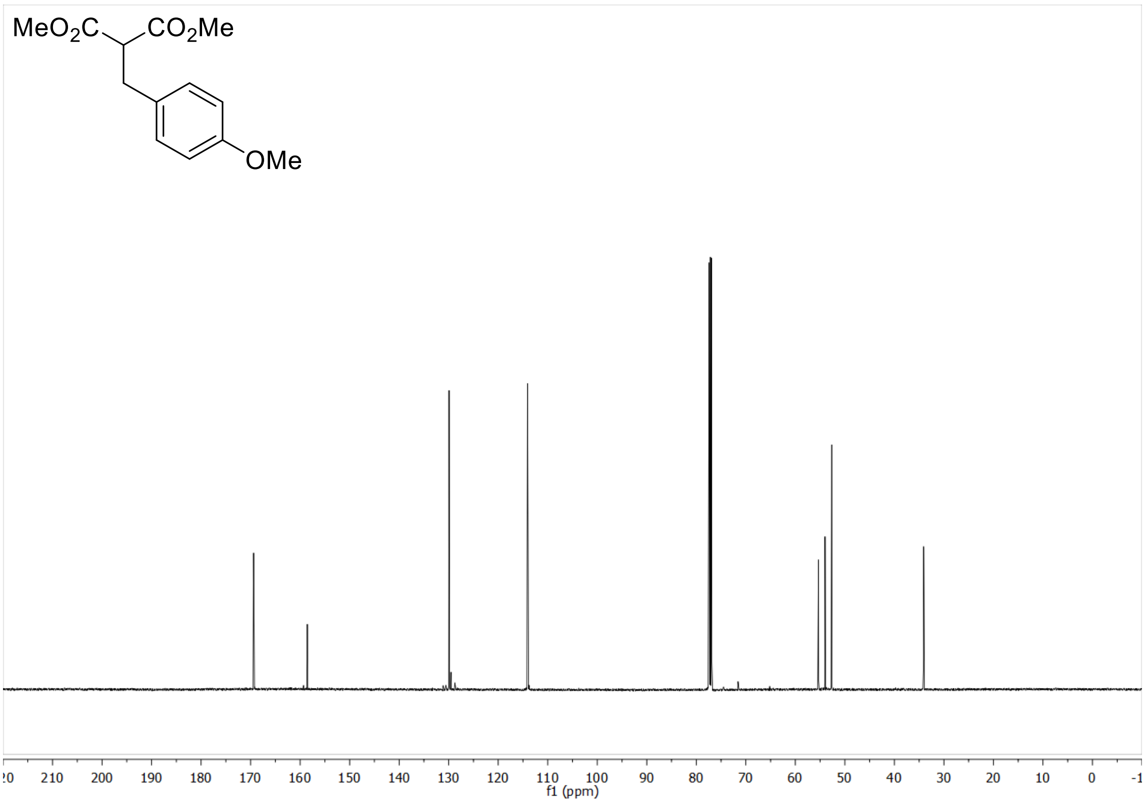

Supplementary Figure 17. ^{1}H NMR (400 MHz, $CDCl_3$) spectrum of **S10**

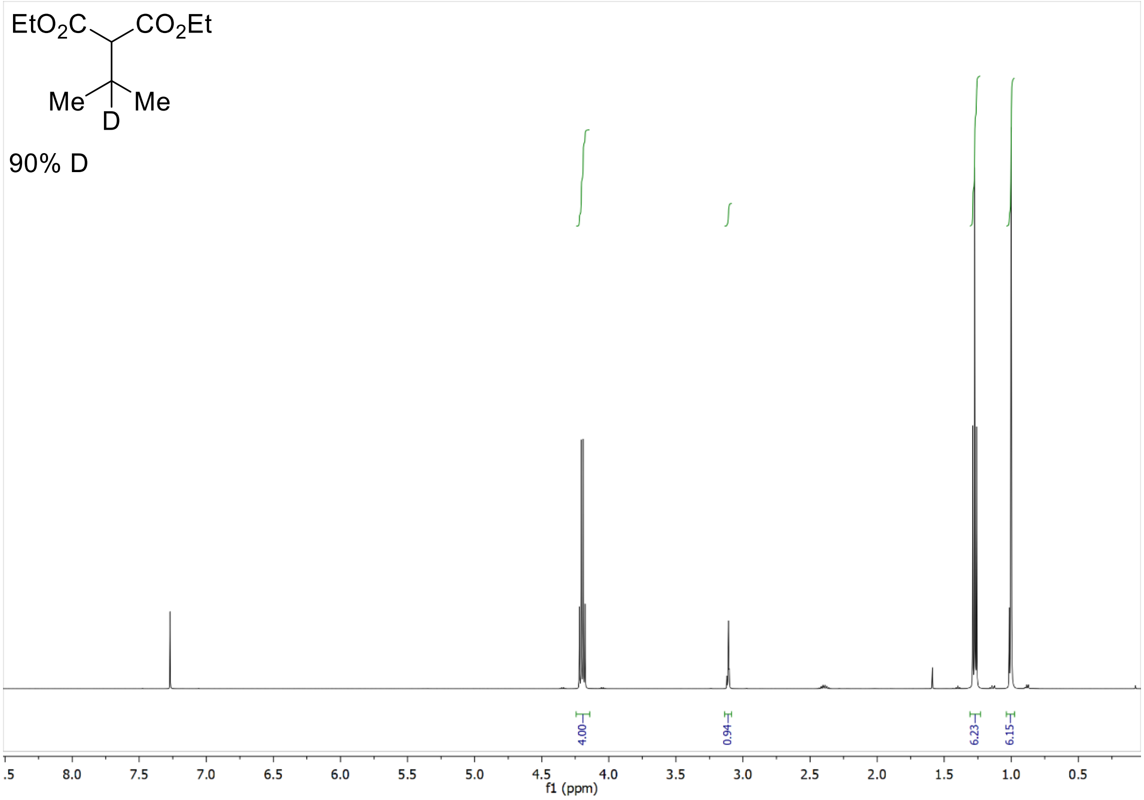

Supplementary Figure 18. ^{13}C NMR (100 MHz, $CDCl_3$) spectrum of **S10**

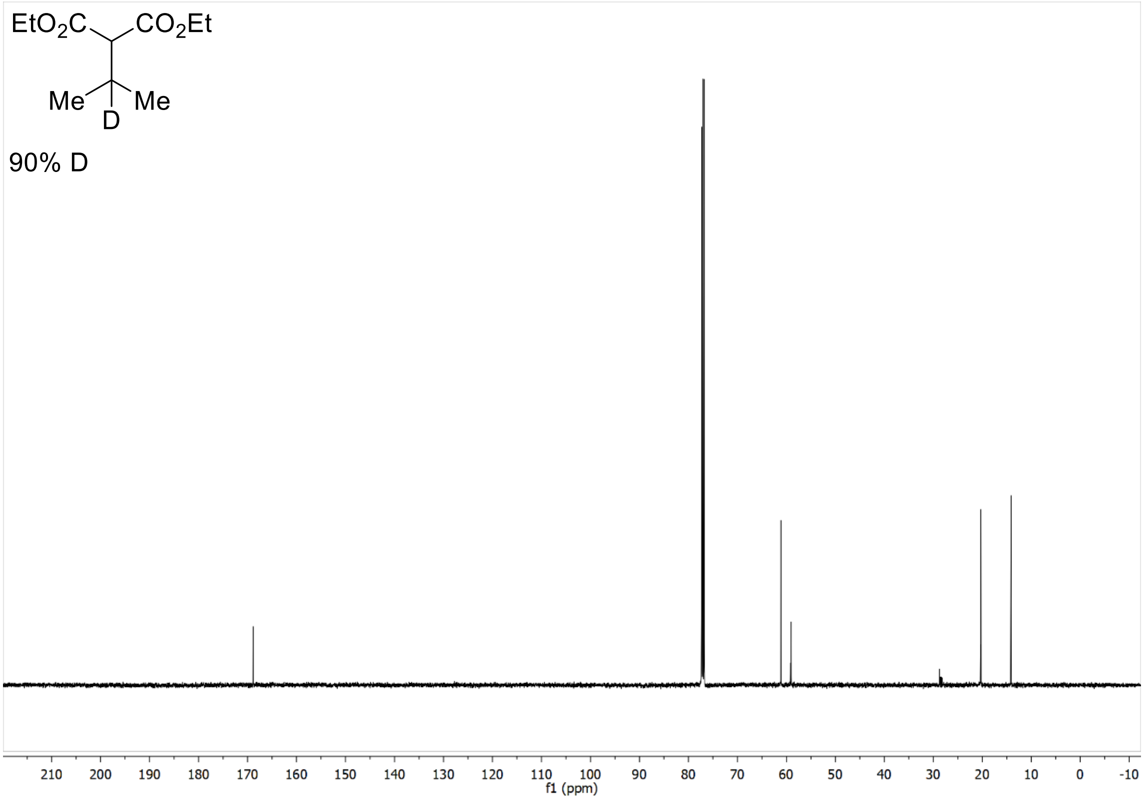

Supplementary Figure 19. ^{1}H NMR (500 MHz, $CDCl_3$) spectrum of **S11**

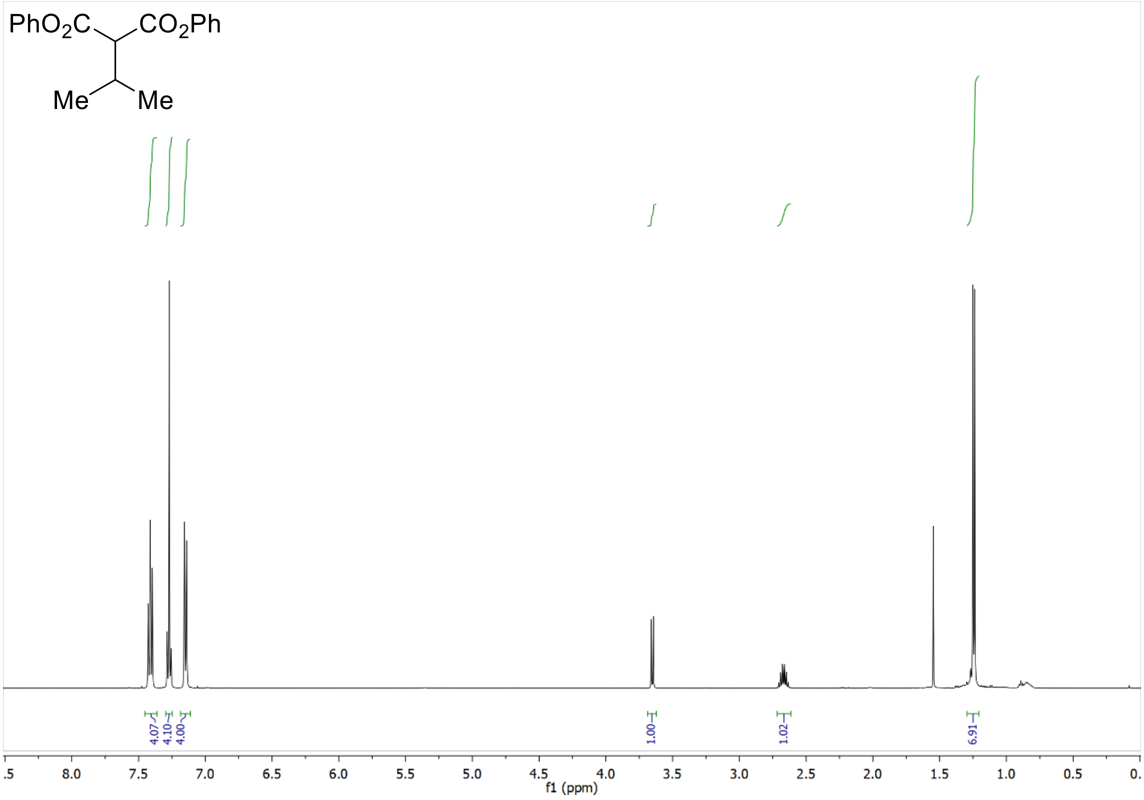

Supplementary Figure 20. ^{13}C NMR (125 MHz, $CDCl_3$) spectrum of **S11**

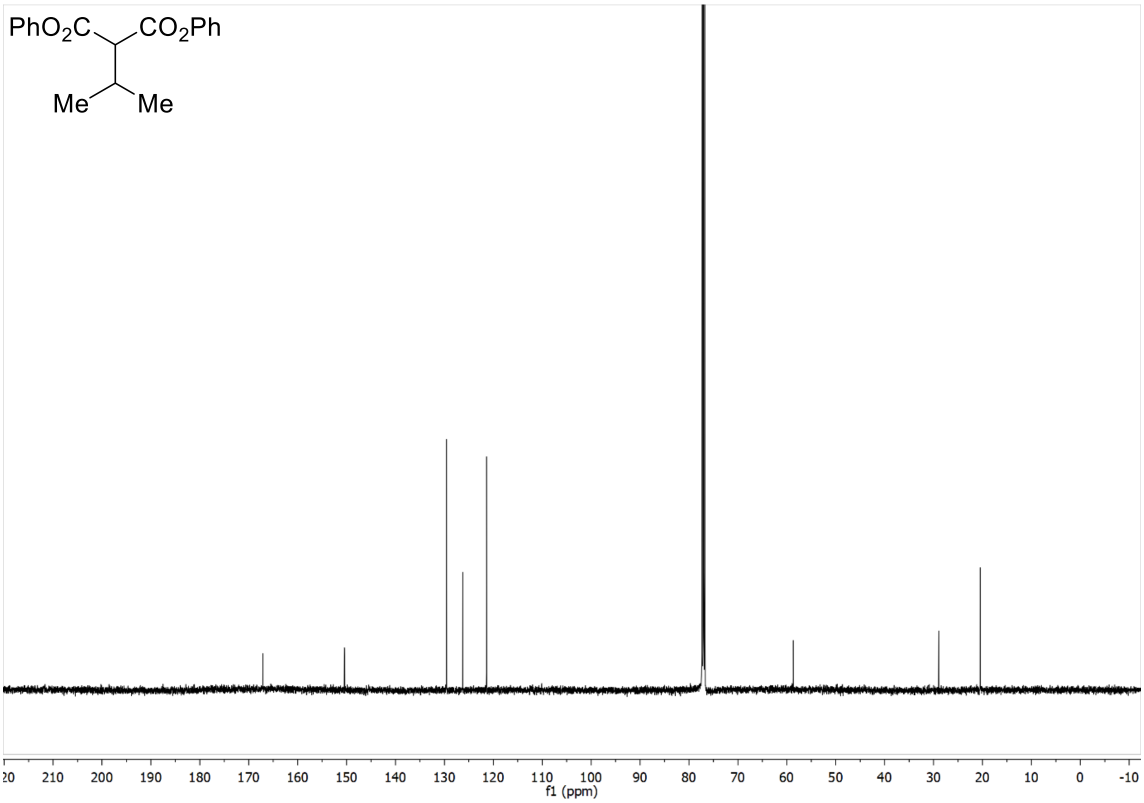

Supplementary Figure 21. ^{1}H NMR (500 MHz, $CDCl_3$) spectrum of **S12**

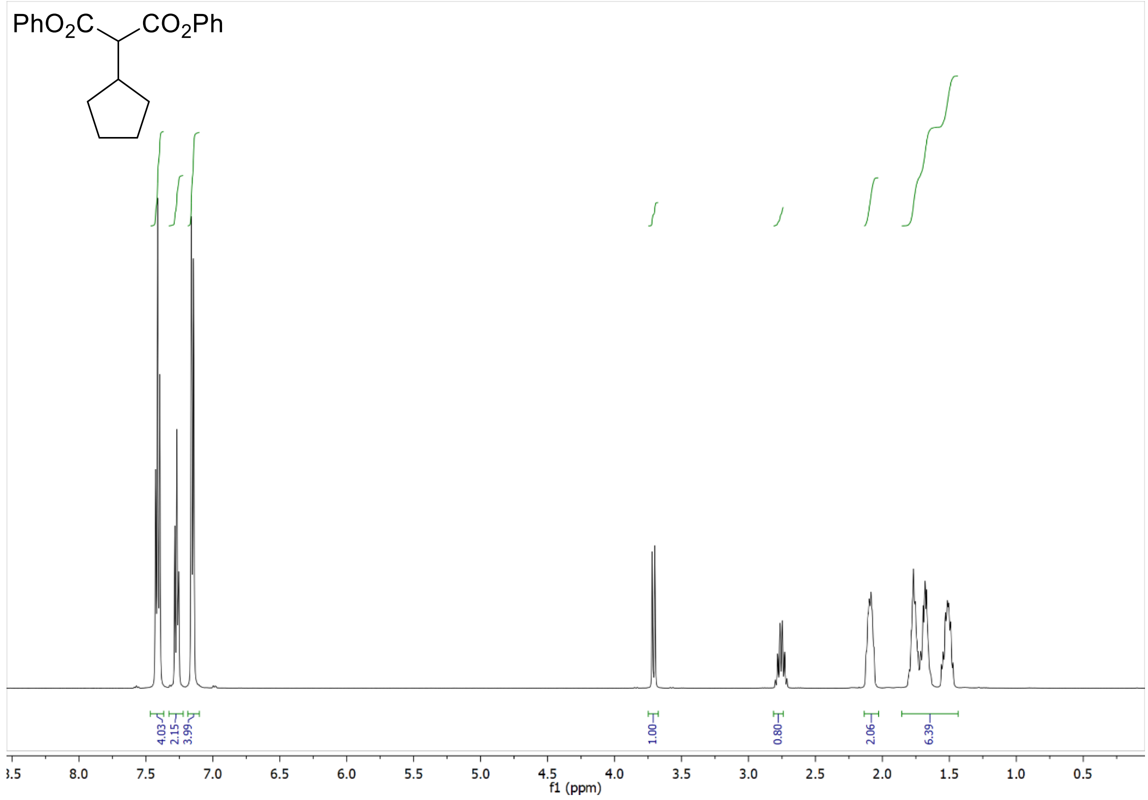

Supplementary Figure 22. ^{13}C NMR (125 MHz, $CDCl_3$) spectrum of **S12**

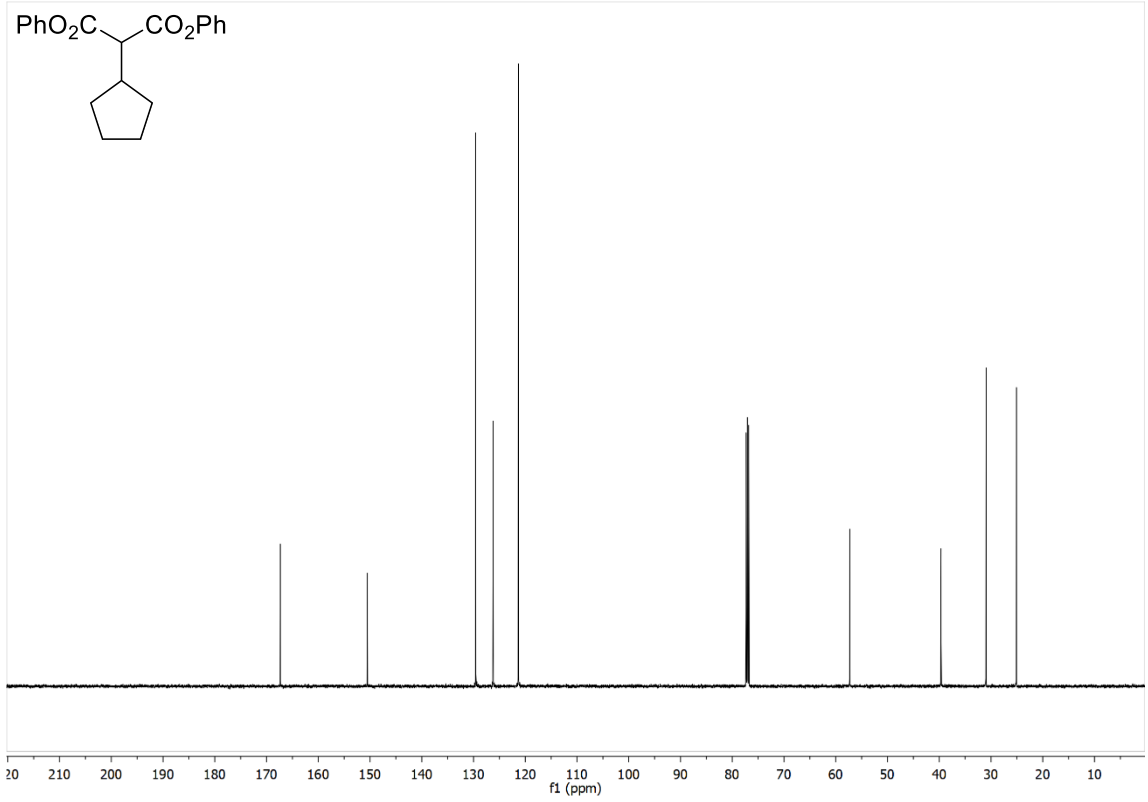

Supplementary Figure 23. ^{1}H NMR (500 MHz, $CDCl_3$) spectrum of **S13**

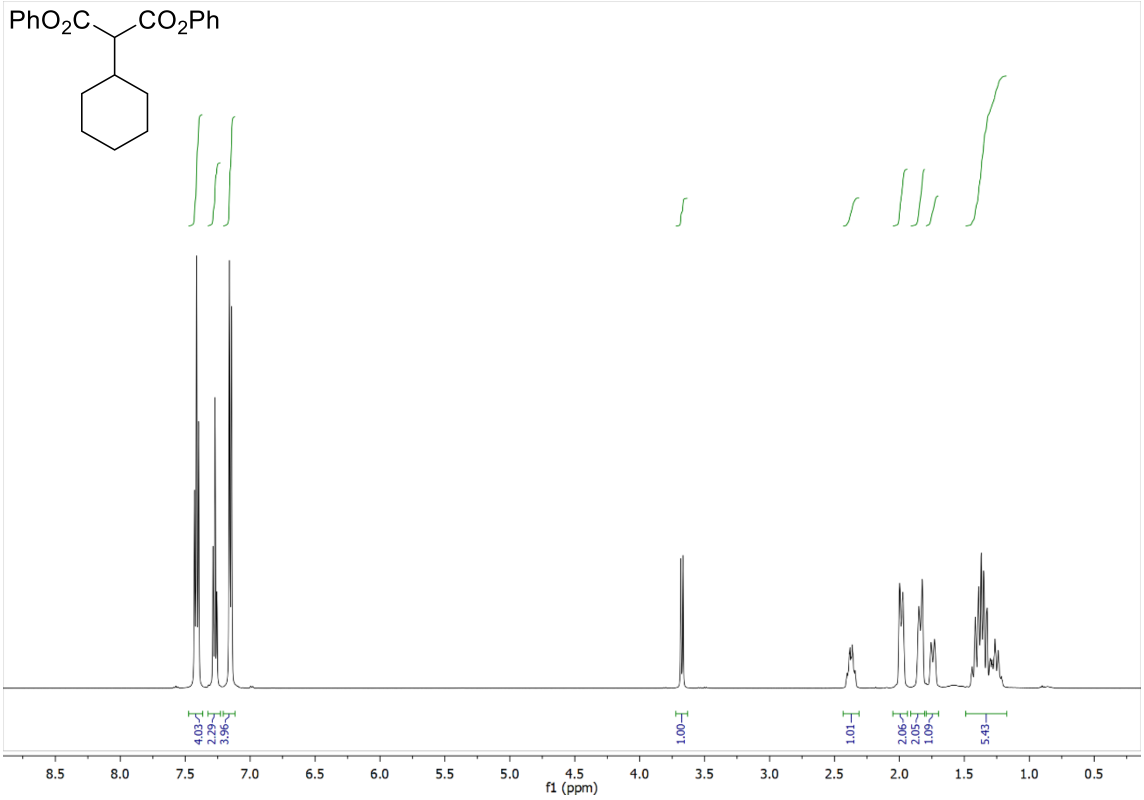

Supplementary Figure 24. ^{13}C NMR (125 MHz, $CDCl_3$) spectrum of **S13**

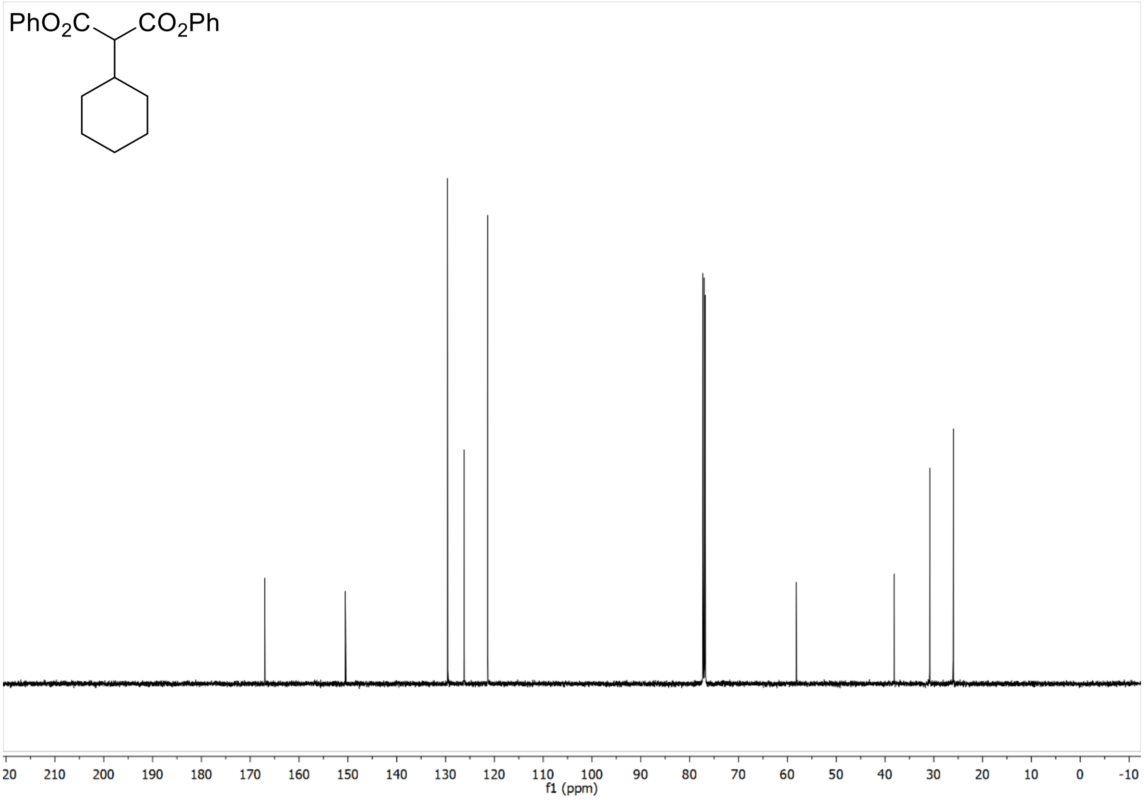

Supplementary Figure 25. ^{1}H NMR (500 MHz, $CDCl_3$) spectrum of **S14**

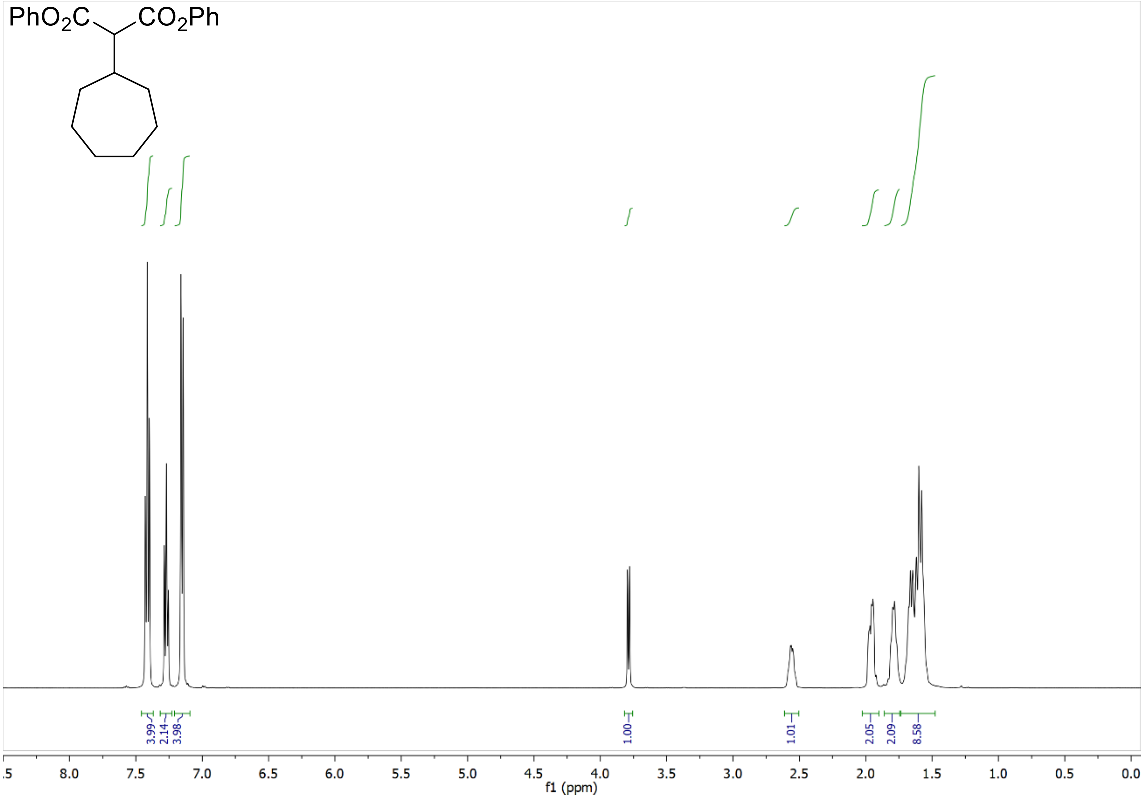

Supplementary Figure 26. ^{13}C NMR (125 MHz, $CDCl_3$) spectrum of **S14**

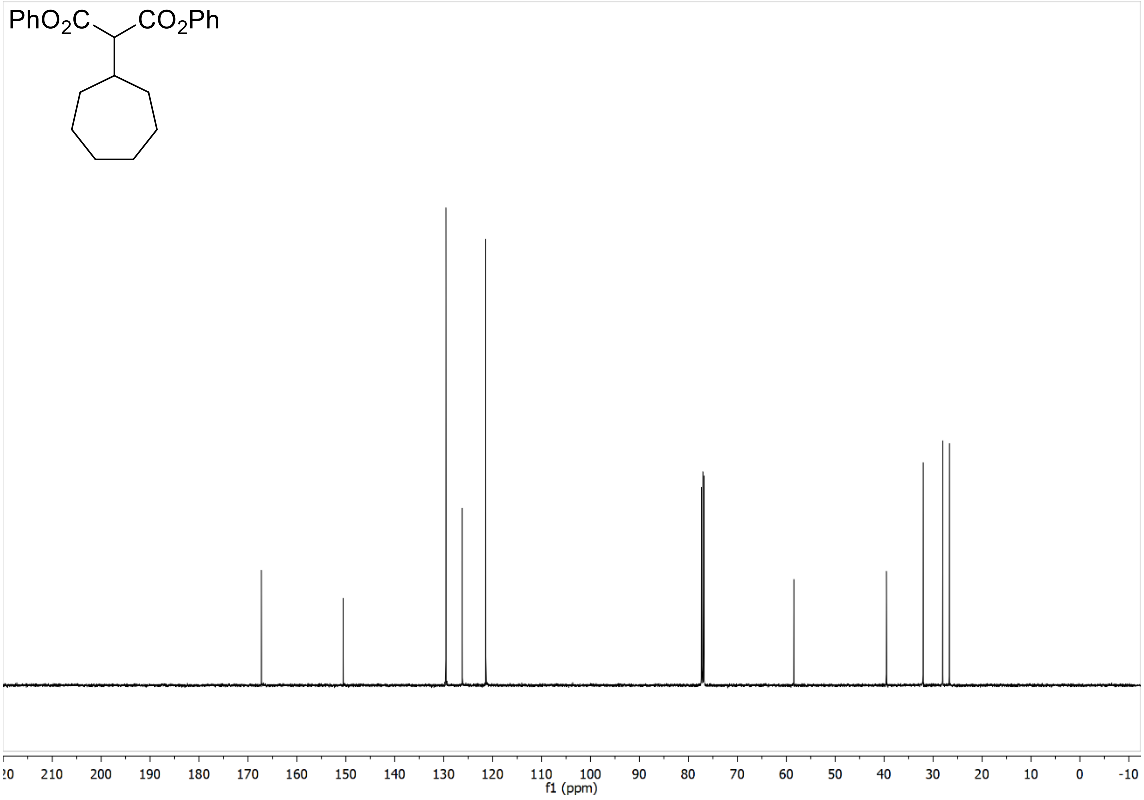

Supplementary Figure 27. ^{1}H NMR (400 MHz, $CDCl_3$) spectrum of **S15**

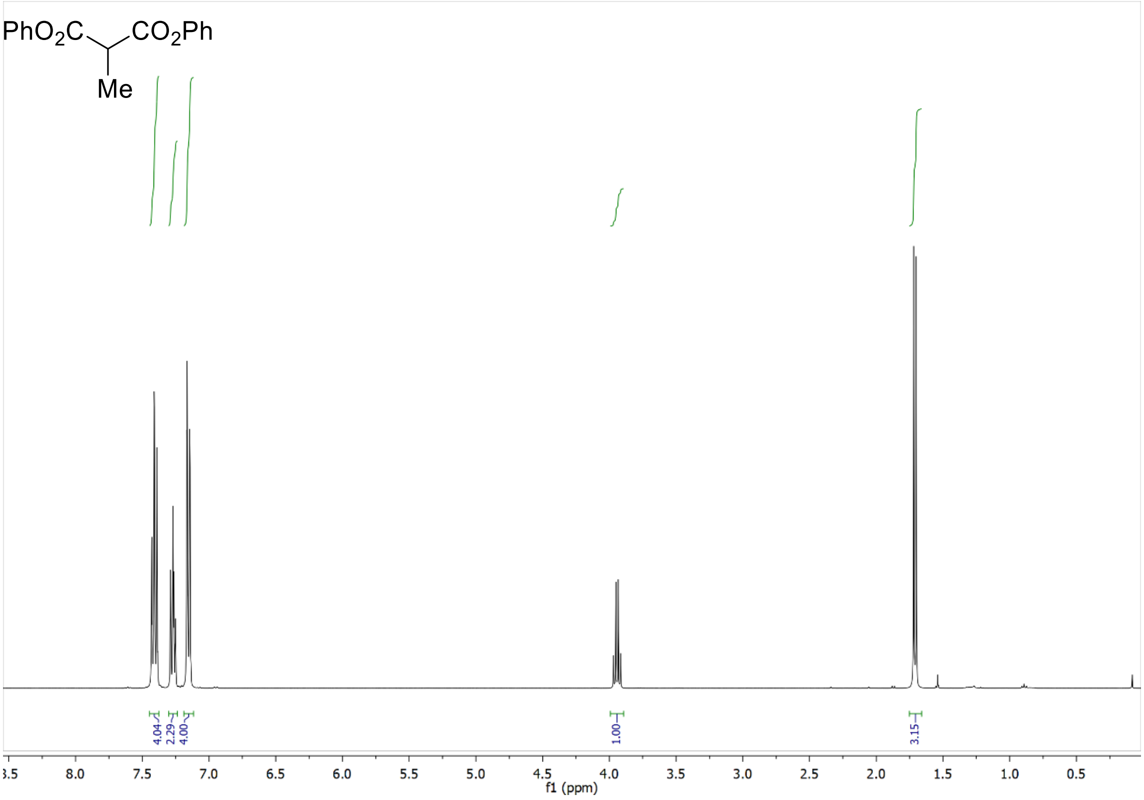

Supplementary Figure 28. ^{13}C NMR (100 MHz, $CDCl_3$) spectrum of **S15**

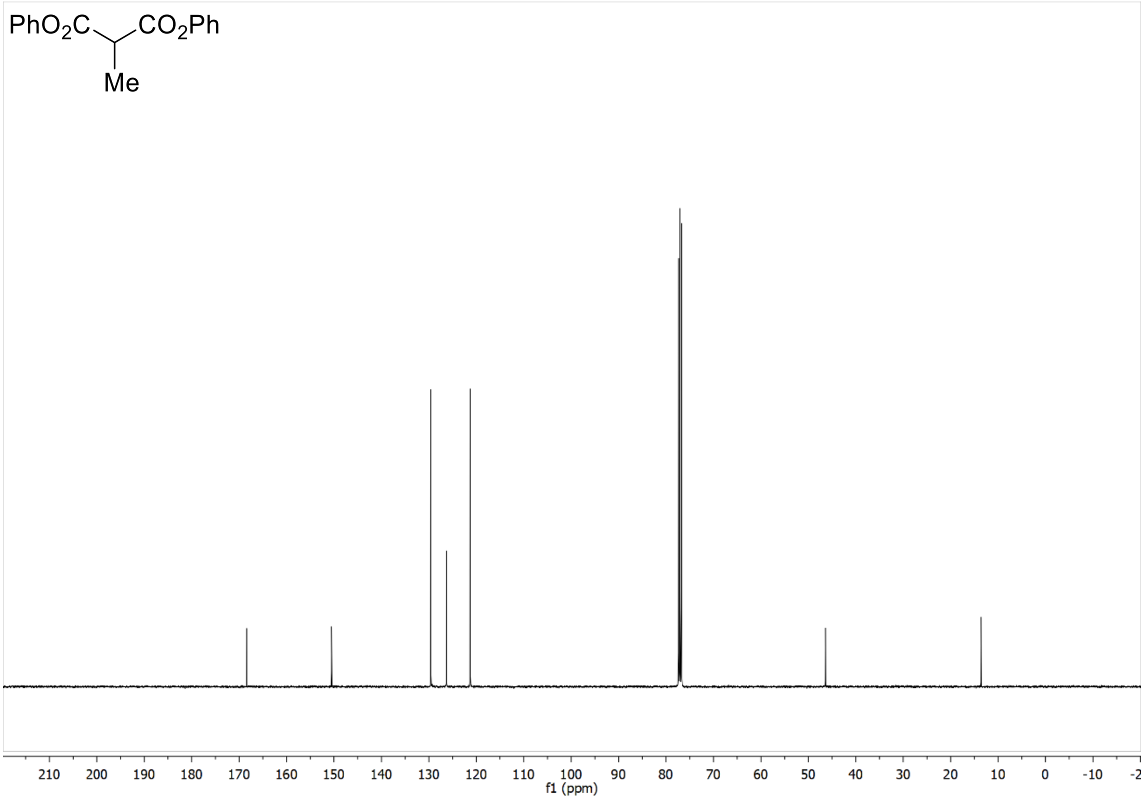

Supplementary Figure 29. ^{1}H NMR (500 MHz, $CDCl_3$) spectrum of **S16**

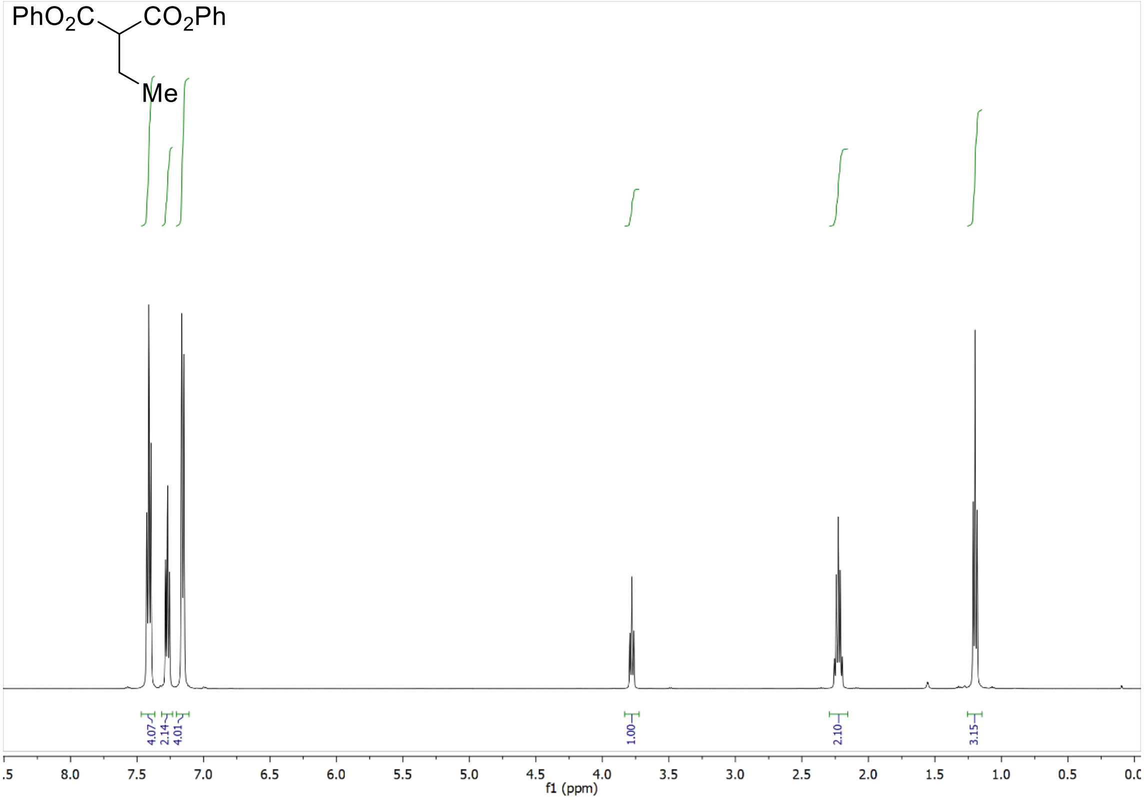

Supplementary Figure 30. ^{13}C NMR (125 MHz, $CDCl_3$) spectrum of **S16**

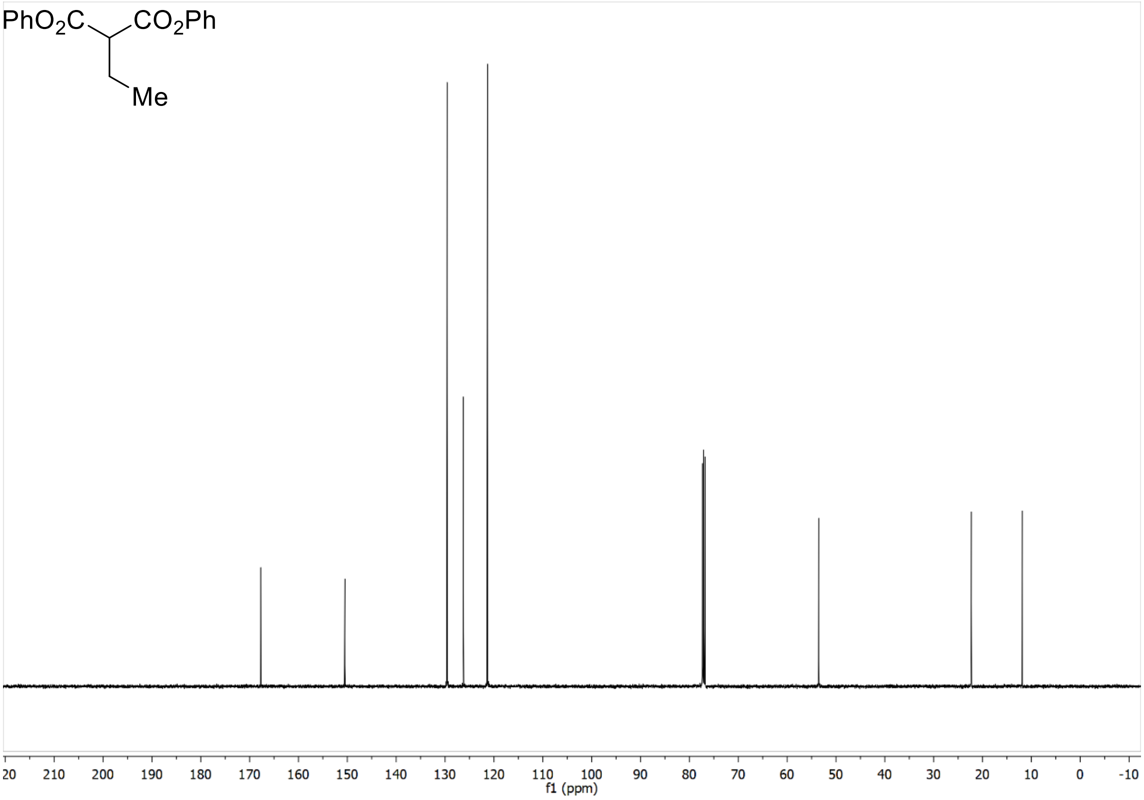

Supplementary Figure 31. ^{1}H NMR (500 MHz, $CDCl_3$) spectrum of **S17**

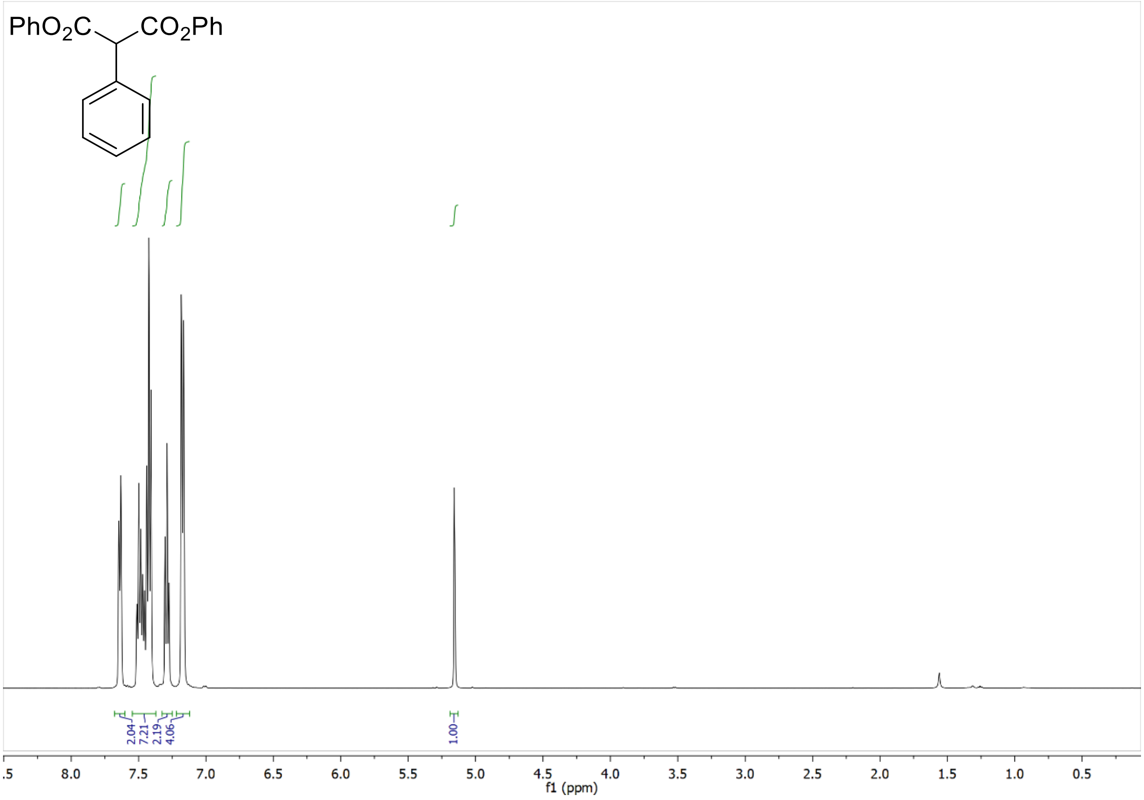

Supplementary Figure 32. ^{13}C NMR (125 MHz, $CDCl_3$) spectrum of **S17**

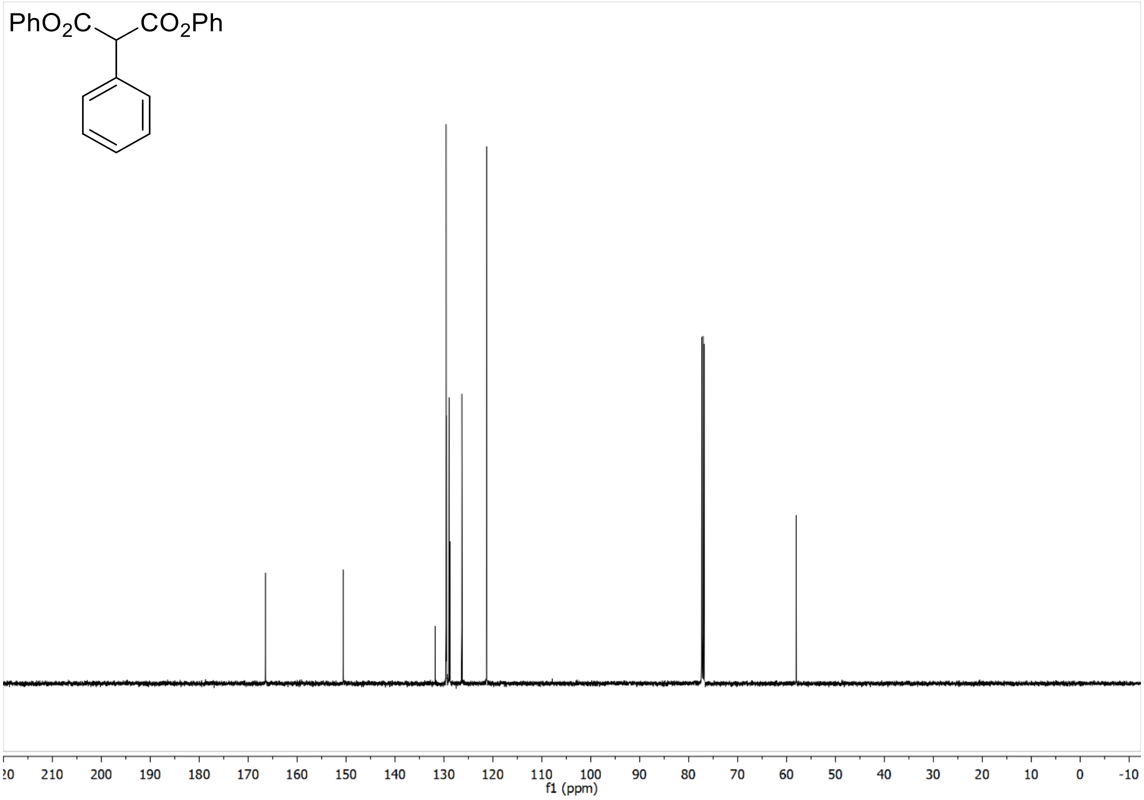

Supplementary Figure 33. ^{1}H NMR (500 MHz, $CDCl_3$) spectrum of **S18**

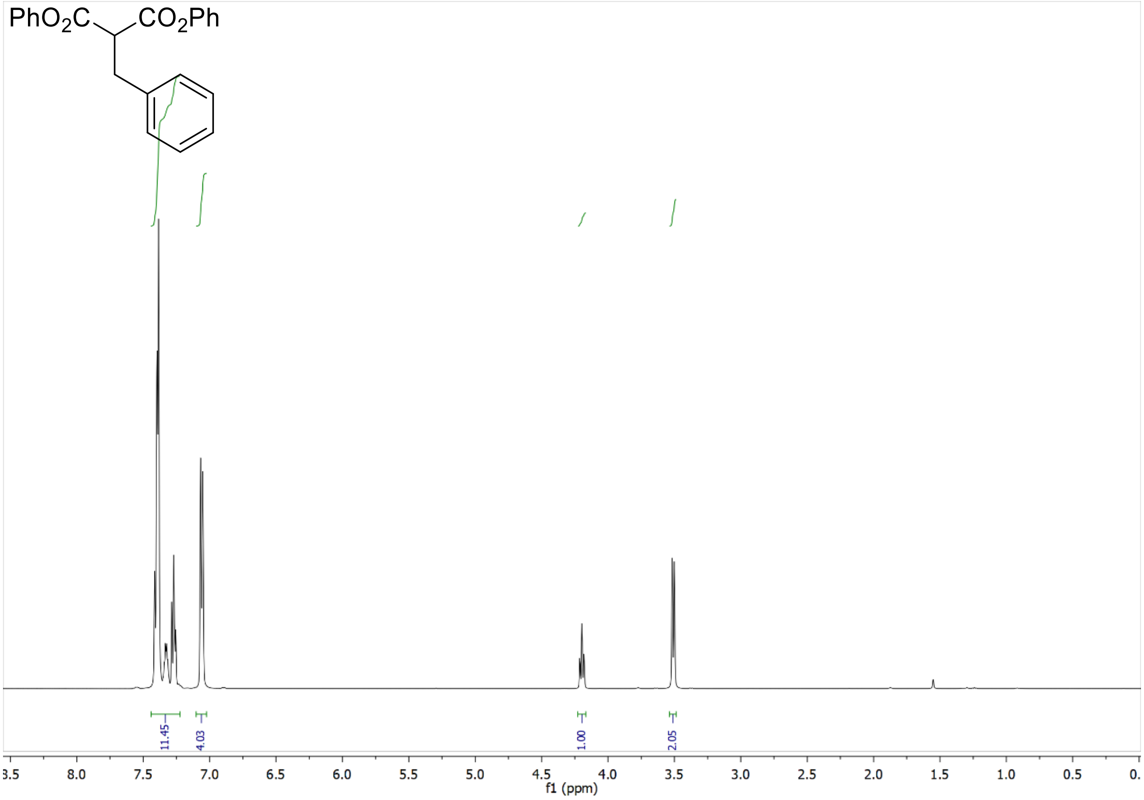

Supplementary Figure 34. ^{13}C NMR (125 MHz, $CDCl_3$) spectrum of **S18**

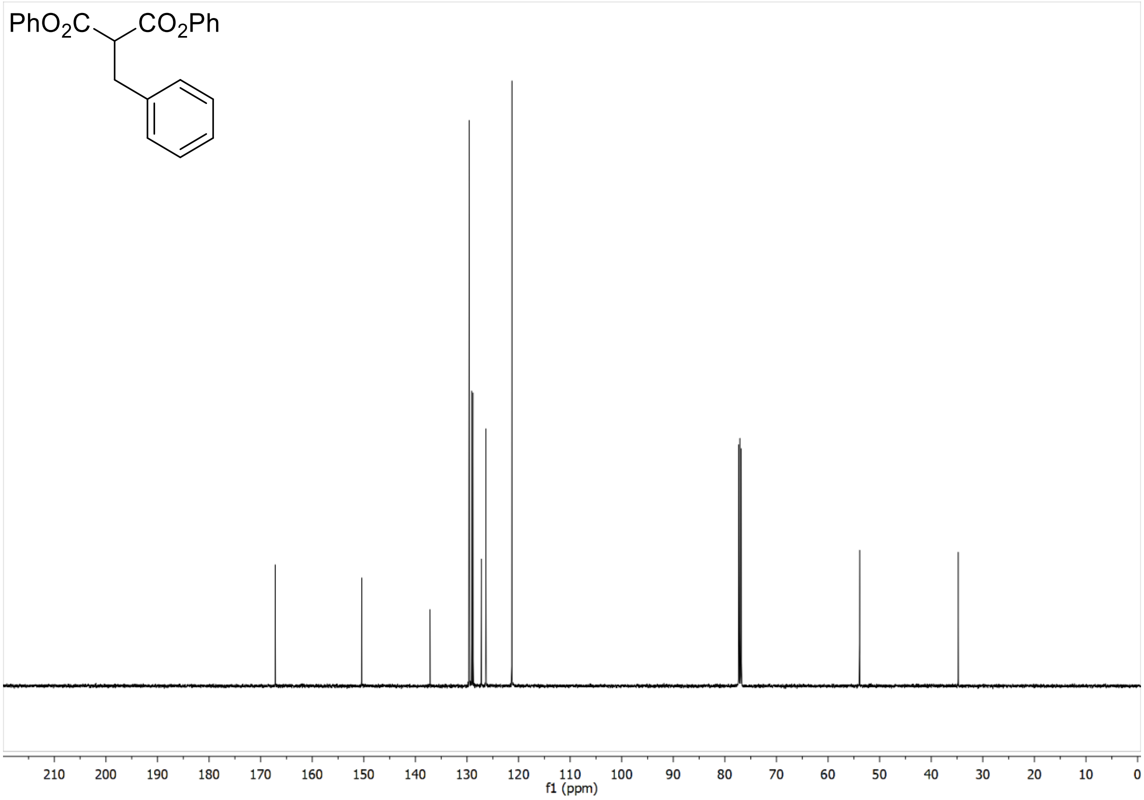

Supplementary Figure 35. ^{1}H NMR (400 MHz, $CDCl_3$) spectrum of **S19**

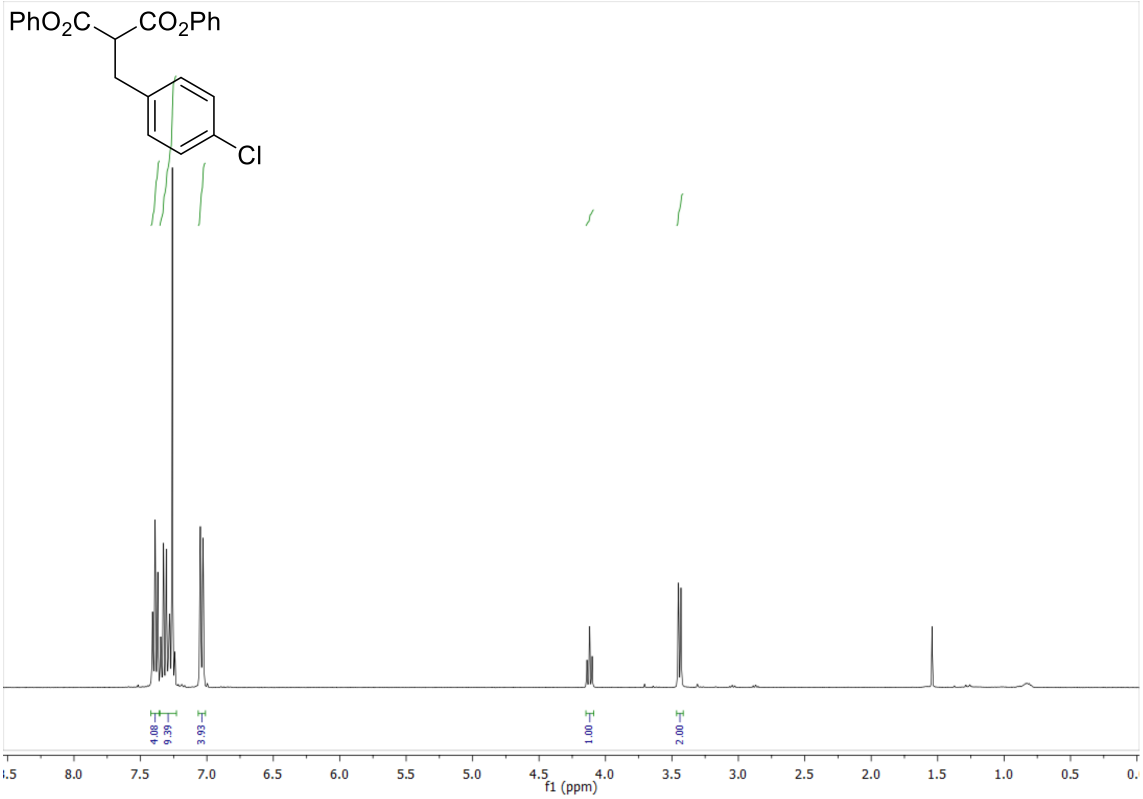

Supplementary Figure 36. ^{13}C NMR (100 MHz, $CDCl_3$) spectrum of **S19**

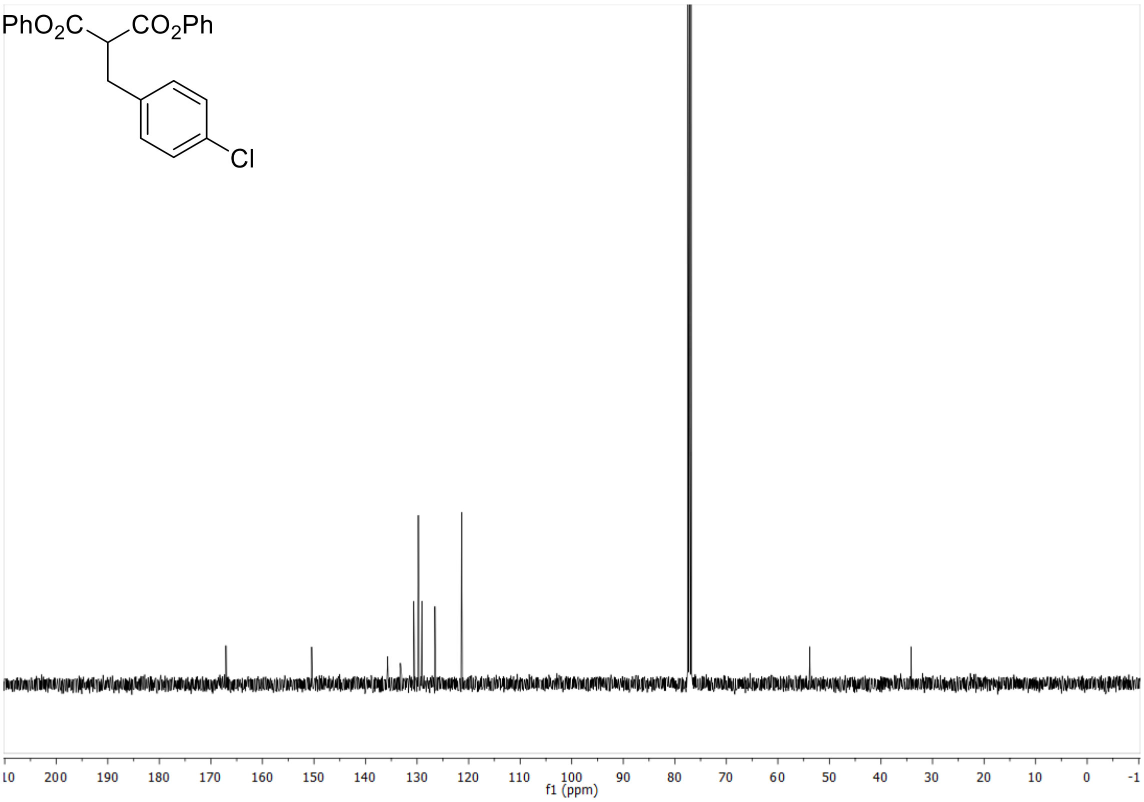

Supplementary Figure 37. ^{1}H NMR (400 MHz, $CDCl_3$) spectrum of **S20**

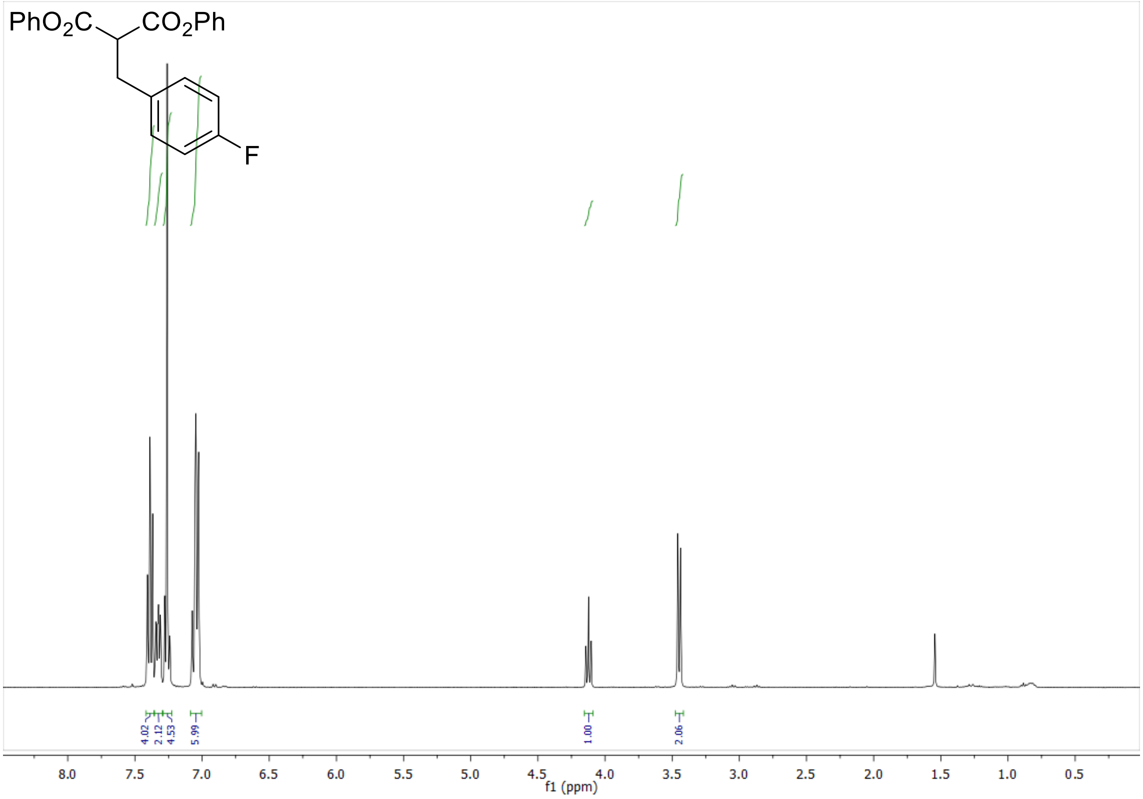

Supplementary Figure 38. ^{13}C NMR (100 MHz, $CDCl_3$) spectrum of **S20**

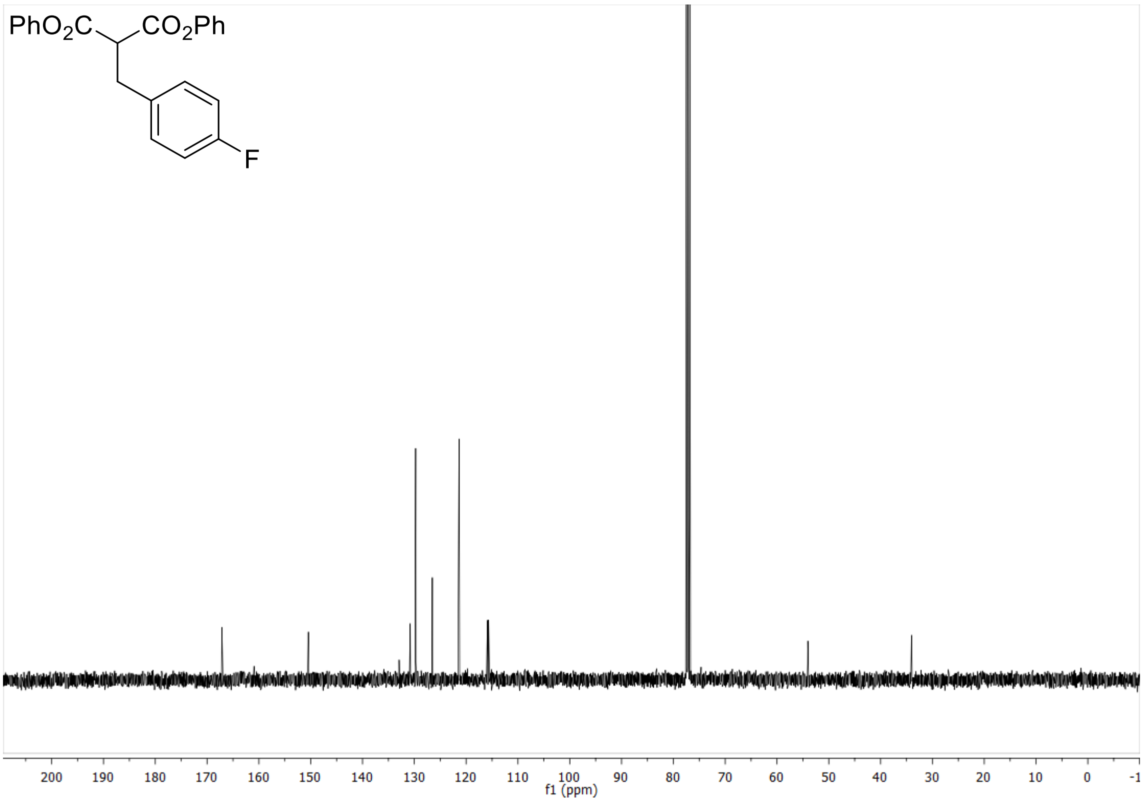

Supplementary Figure 39. ^{1}H NMR (400 MHz, $CDCl_3$) spectrum of **S21**

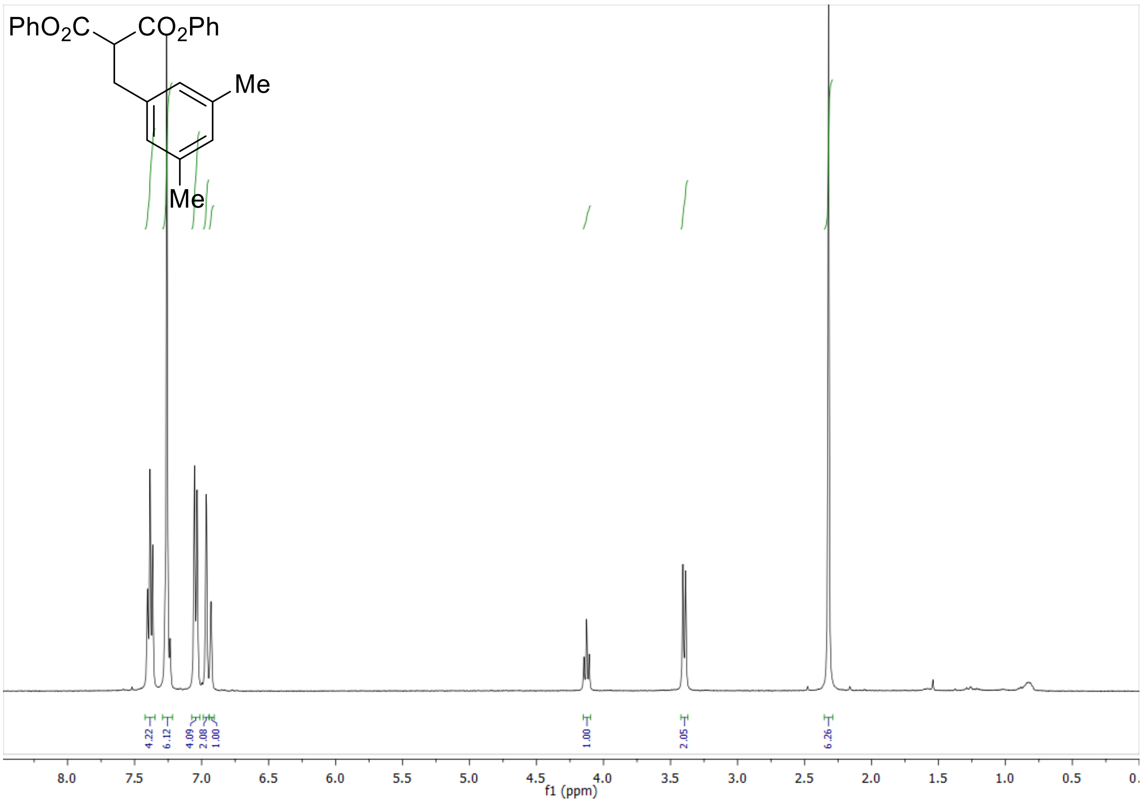

Supplementary Figure 40. ^{13}C NMR (125 MHz, $CDCl_3$) spectrum of **S21**

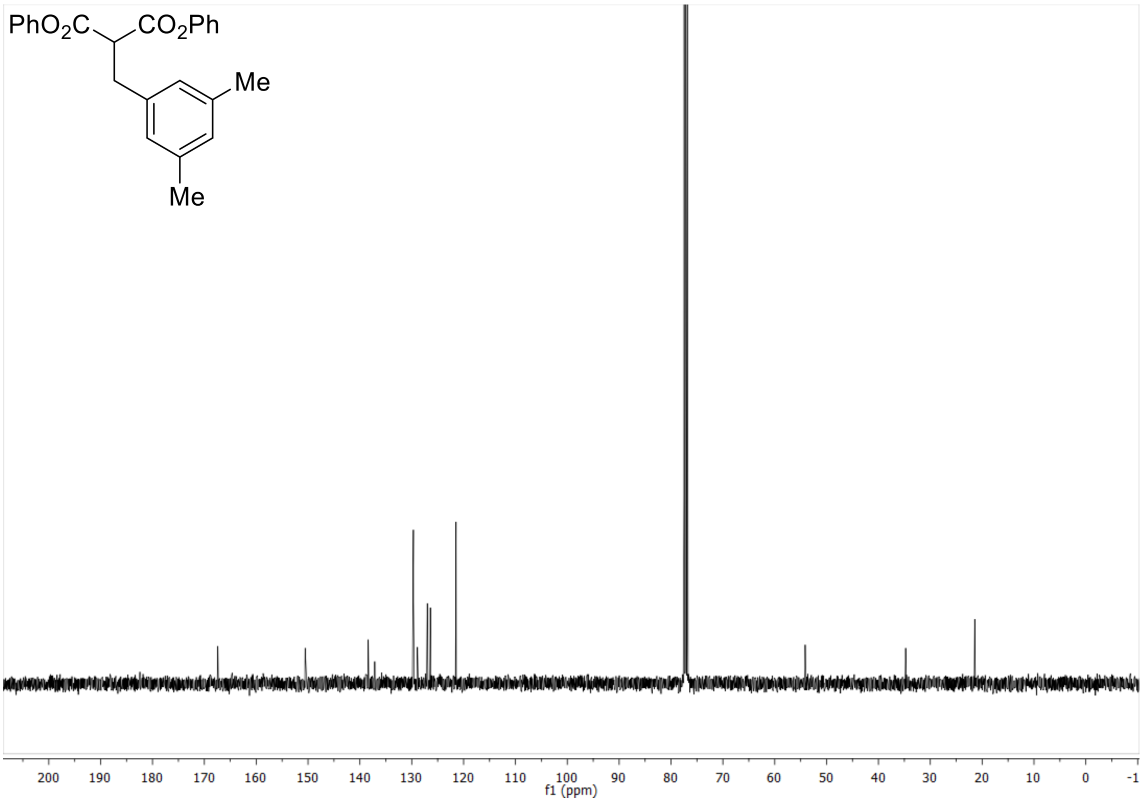

Supplementary Figure 41. ^{1}H NMR (400 MHz, $CDCl_3$) spectrum of **S22**

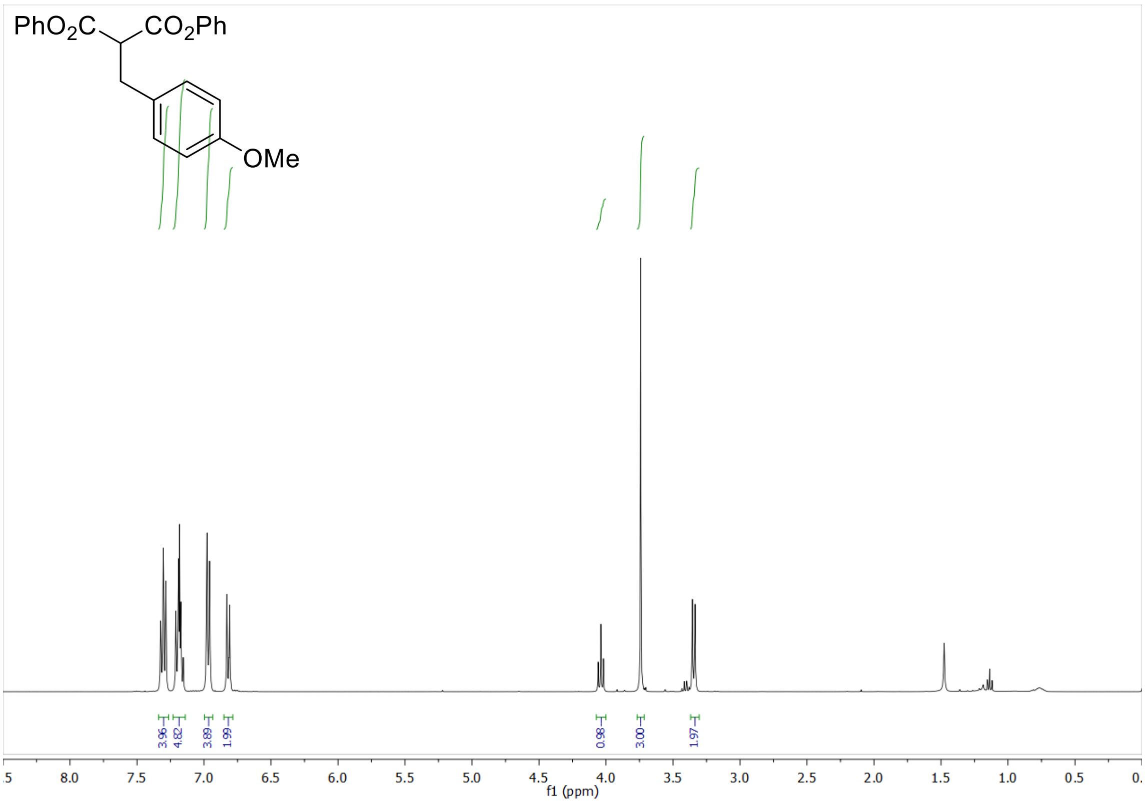

Supplementary Figure 42. ^{13}C NMR (100 MHz, $CDCl_3$) spectrum of **S22**

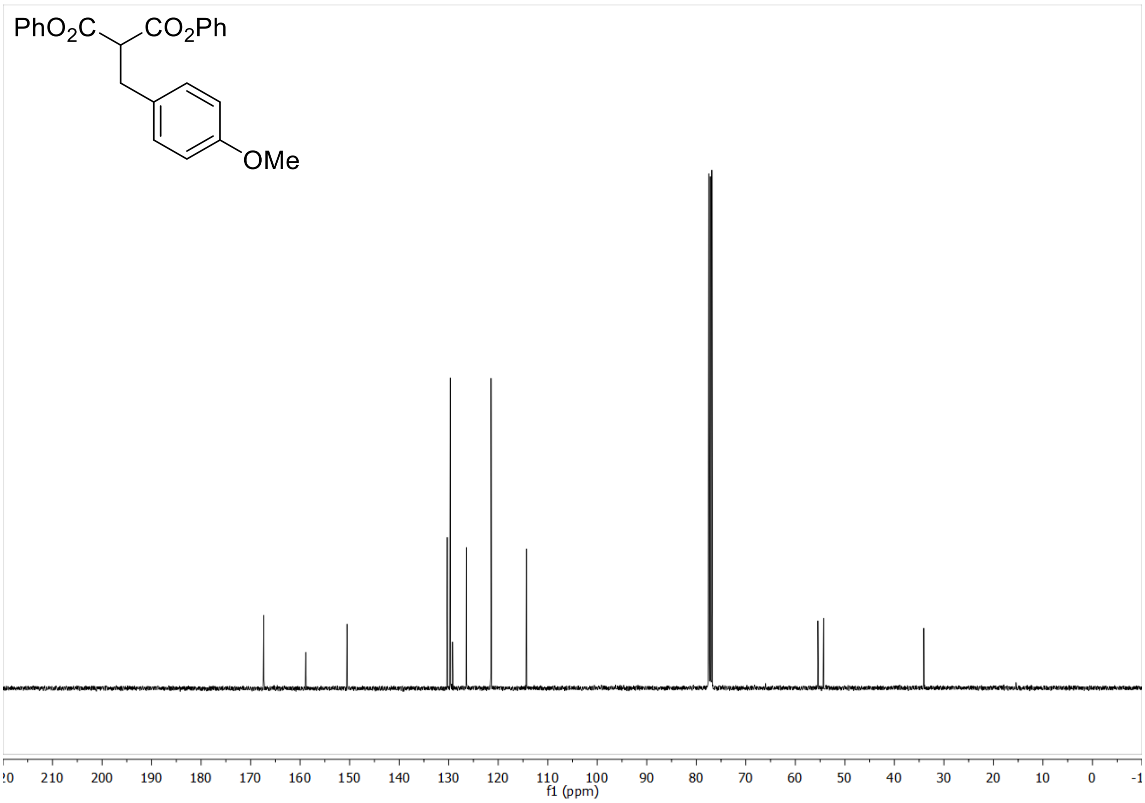

Supplementary Figure 43. ^{1}H NMR (500 MHz, $CDCl_3$) spectrum of **S23**

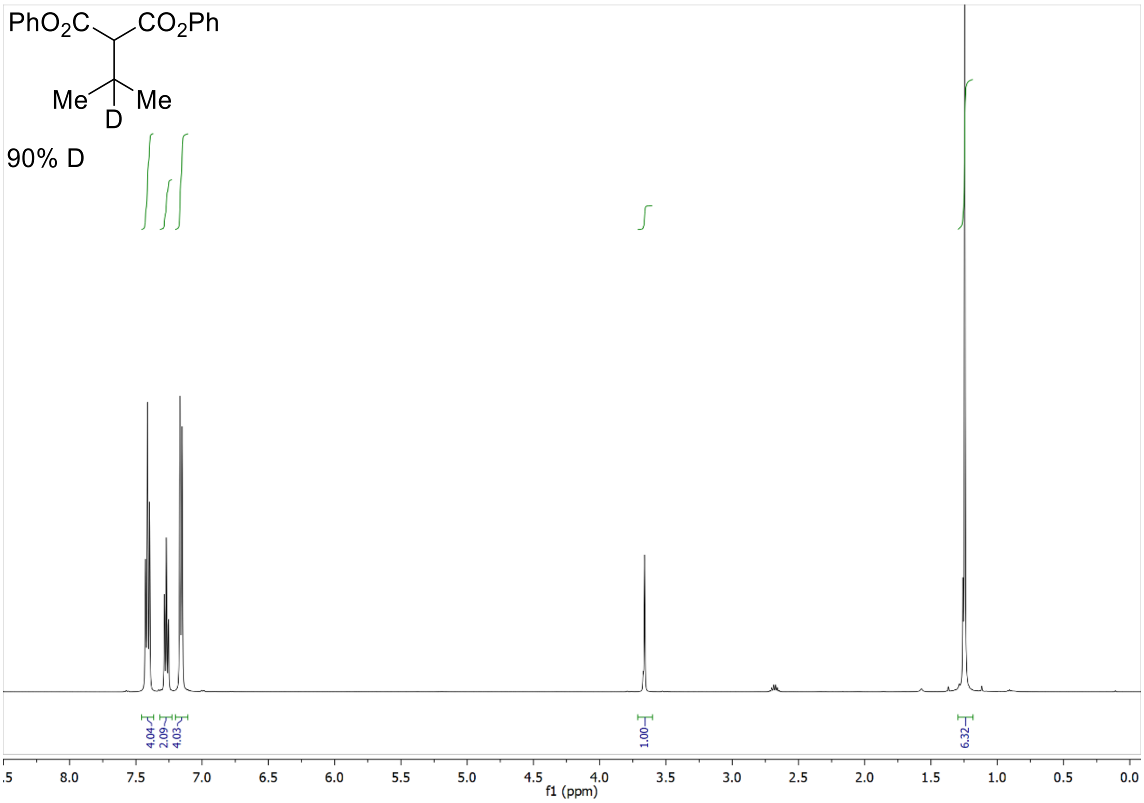

Supplementary Figure 44. ^{13}C NMR (125 MHz, $CDCl_3$) spectrum of **S23**

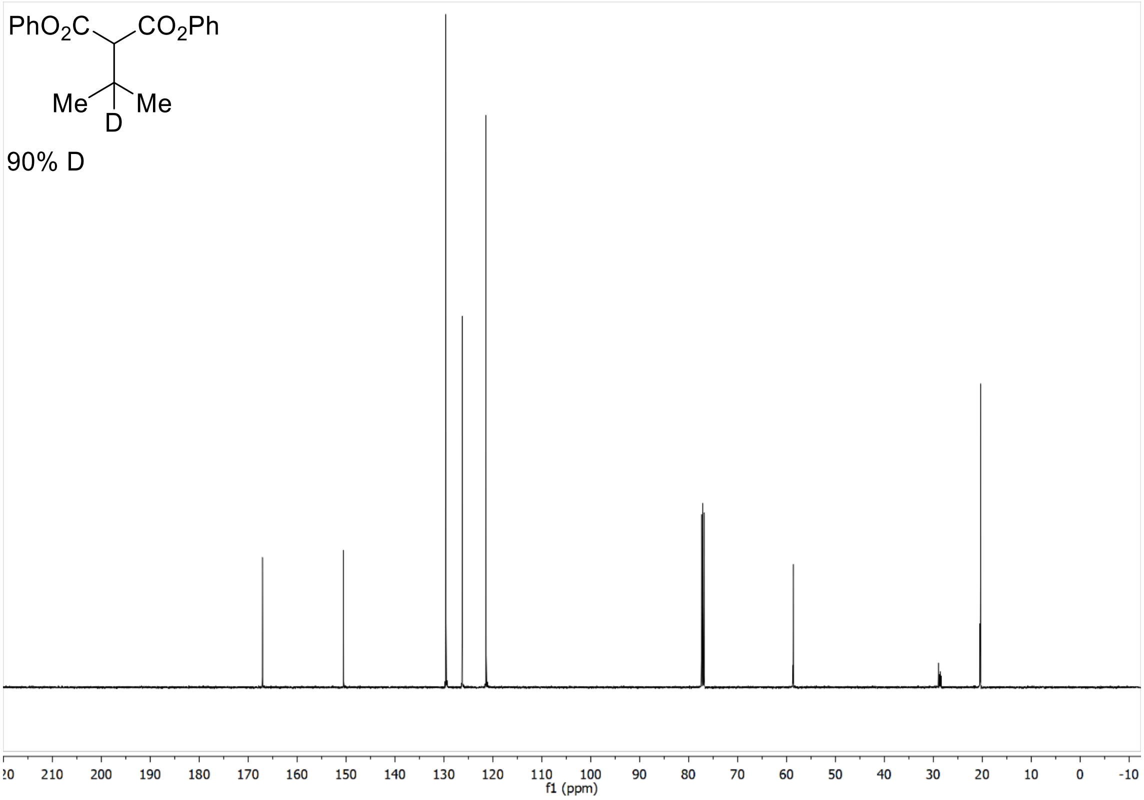

Supplementary Figure 45. ^{1}H NMR (400 MHz, $CDCl_3$) spectrum of **S25**

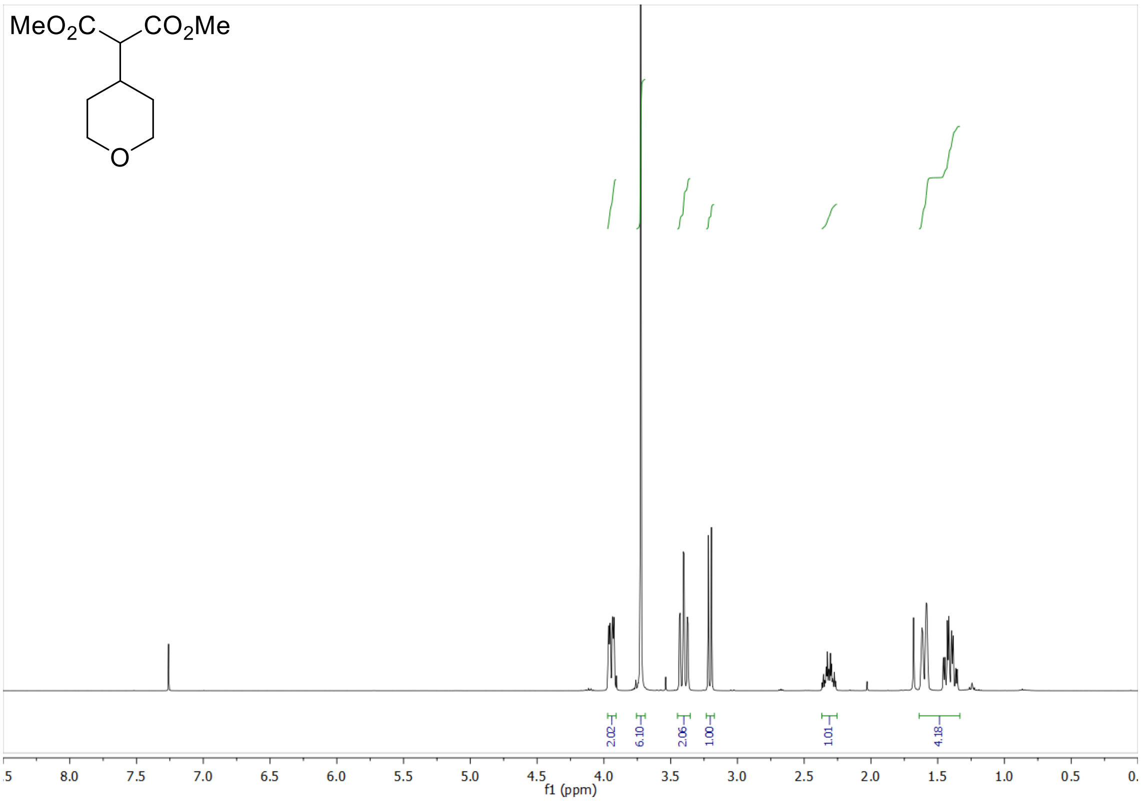

Supplementary Figure 46. ^{13}C NMR (100 MHz, $CDCl_3$) spectrum of **S25**

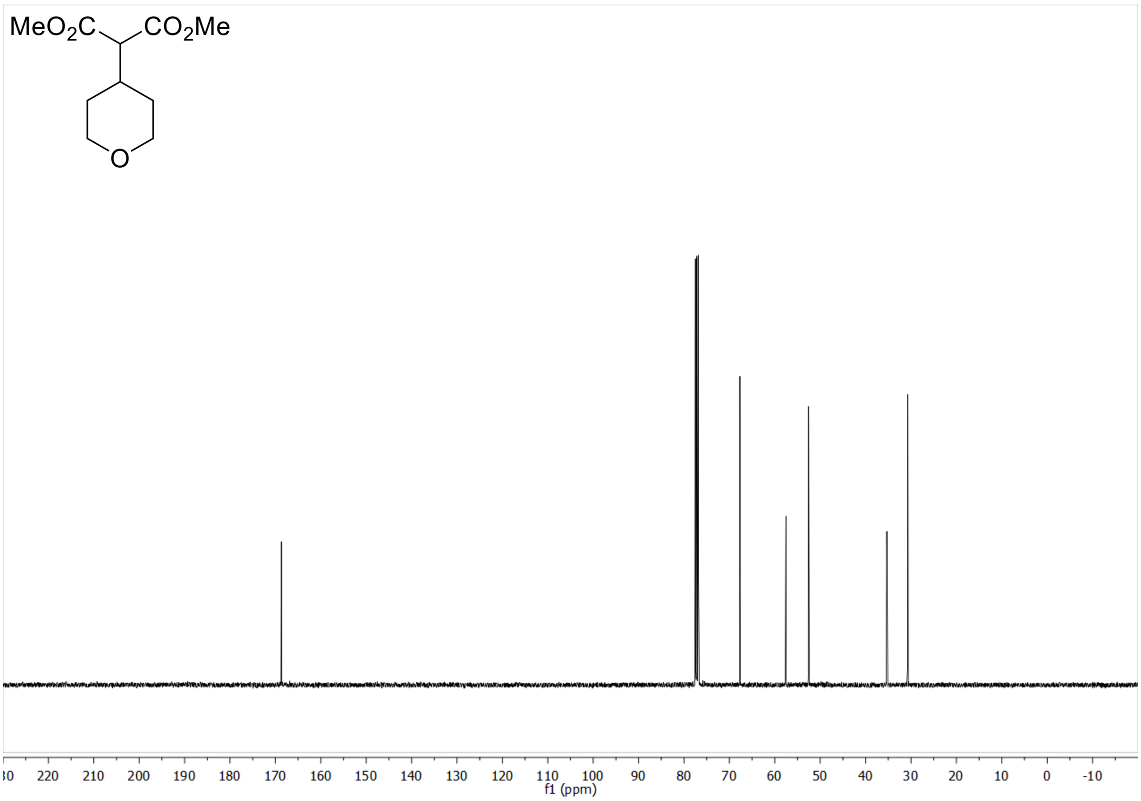

Supplementary Figure 47. ^{1}H NMR (400 MHz, $CDCl_3$) spectrum of **S26**

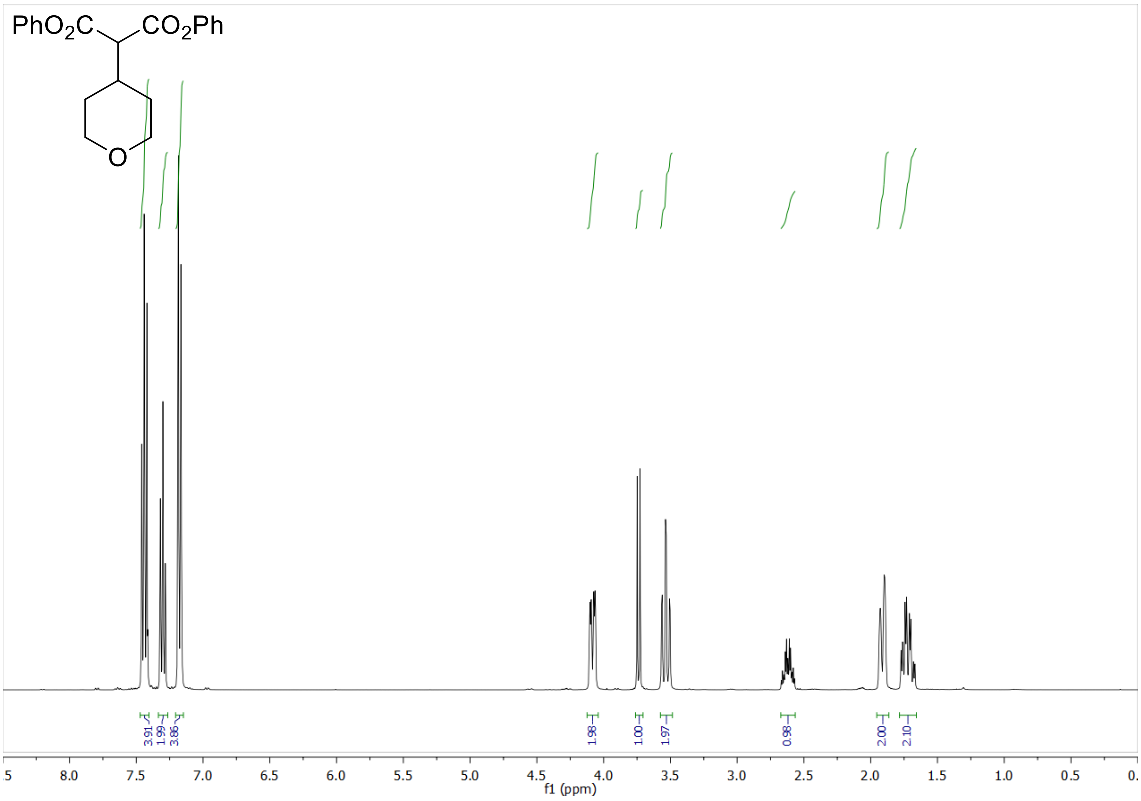

Supplementary Figure 48. ^{13}C NMR (100 MHz, $CDCl_3$) spectrum of **S26**

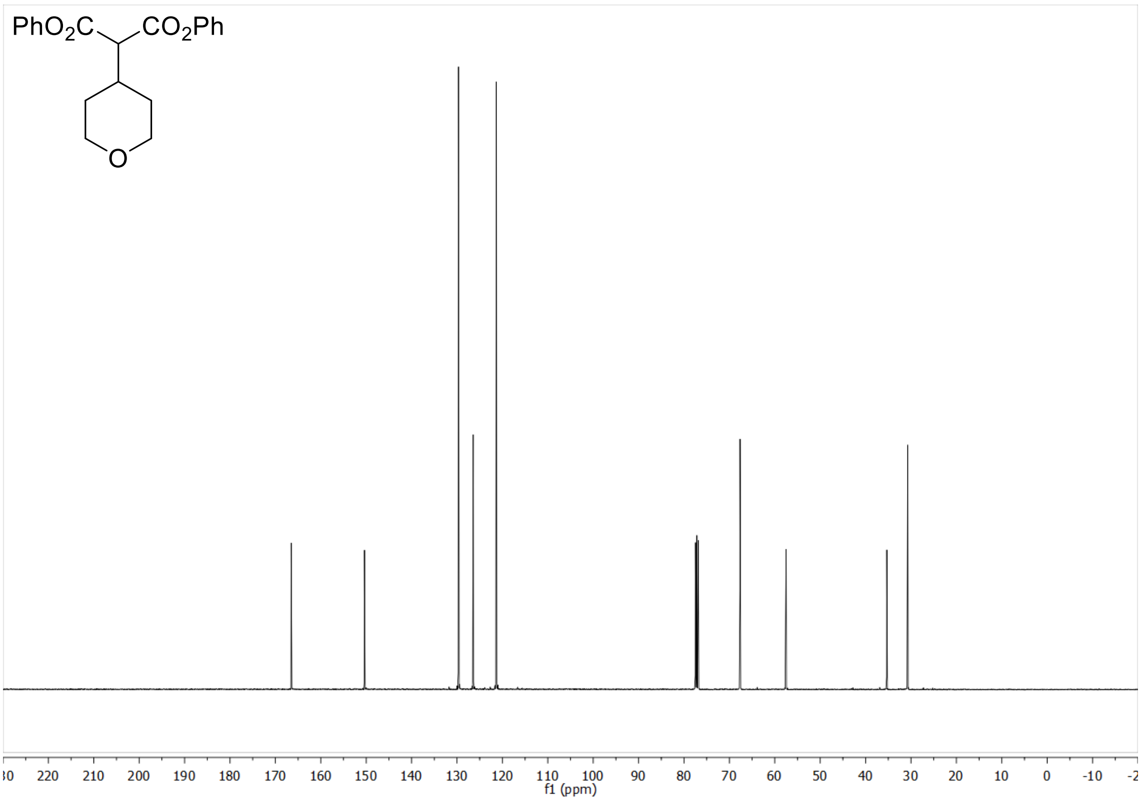

Supplementary Figure 49. ^{1}H NMR (400 MHz, $CDCl_3$) spectrum of **S27**

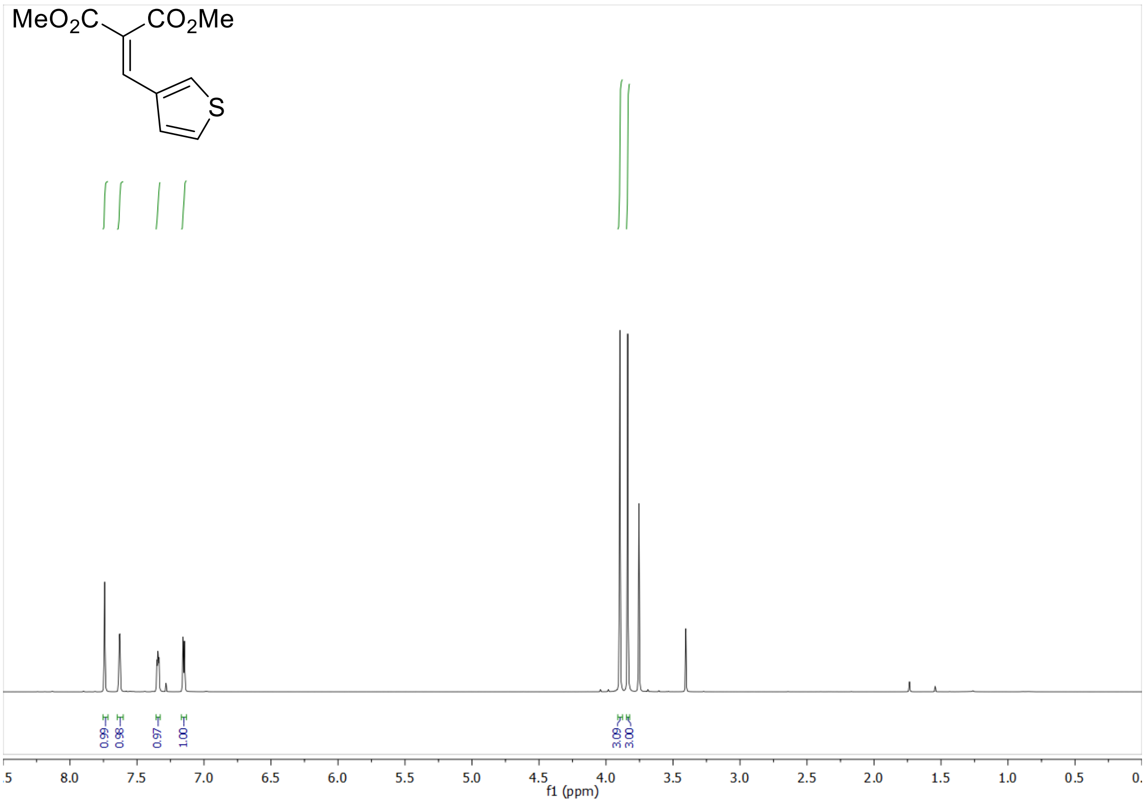

Supplementary Figure 50. ^{13}C NMR (100 MHz, $CDCl_3$) spectrum of **S27**

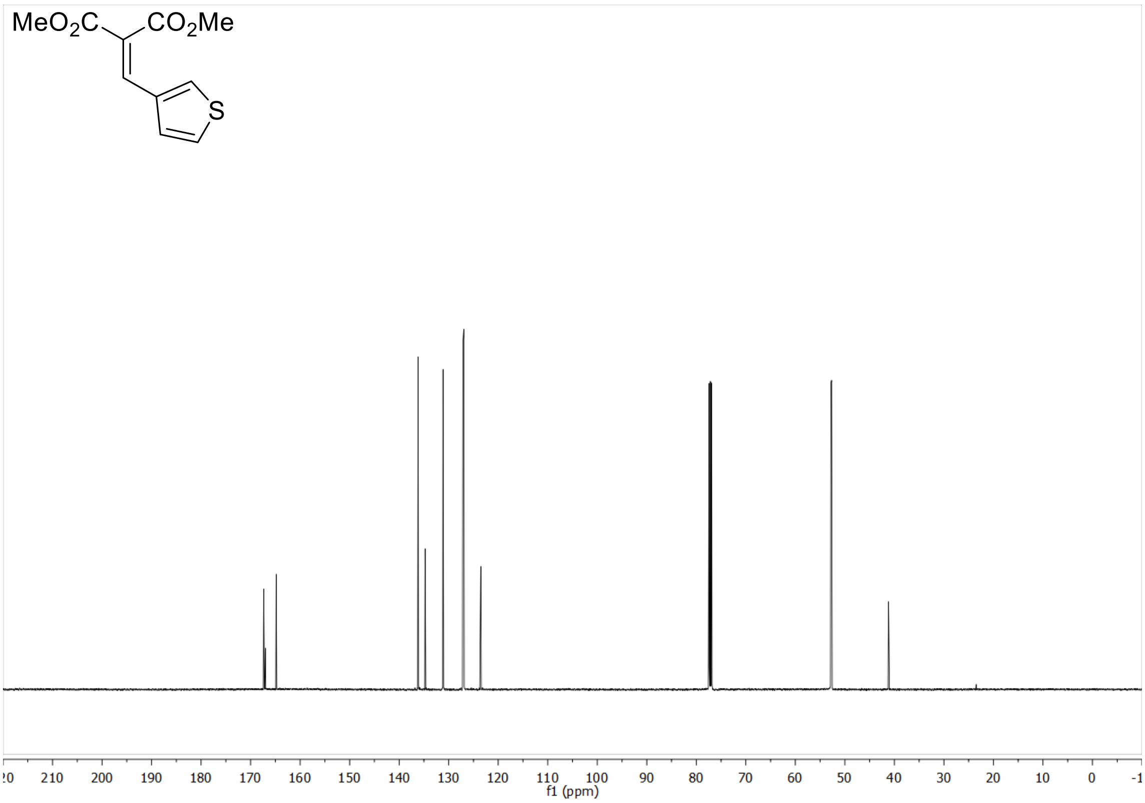

Supplementary Figure 51. ^{1}H NMR (500 MHz, $CDCl_3$) spectrum of **S28**

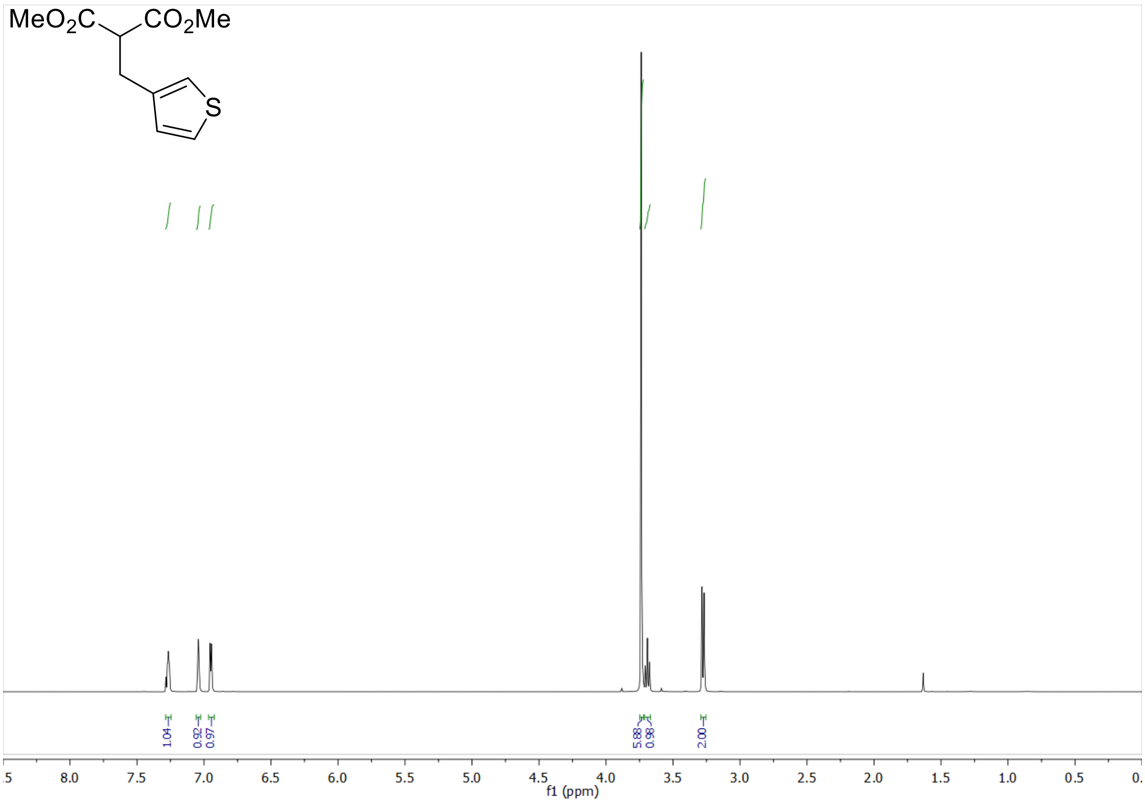

Supplementary Figure 52. ^{13}C NMR (125 MHz, $CDCl_3$) spectrum of **S28**

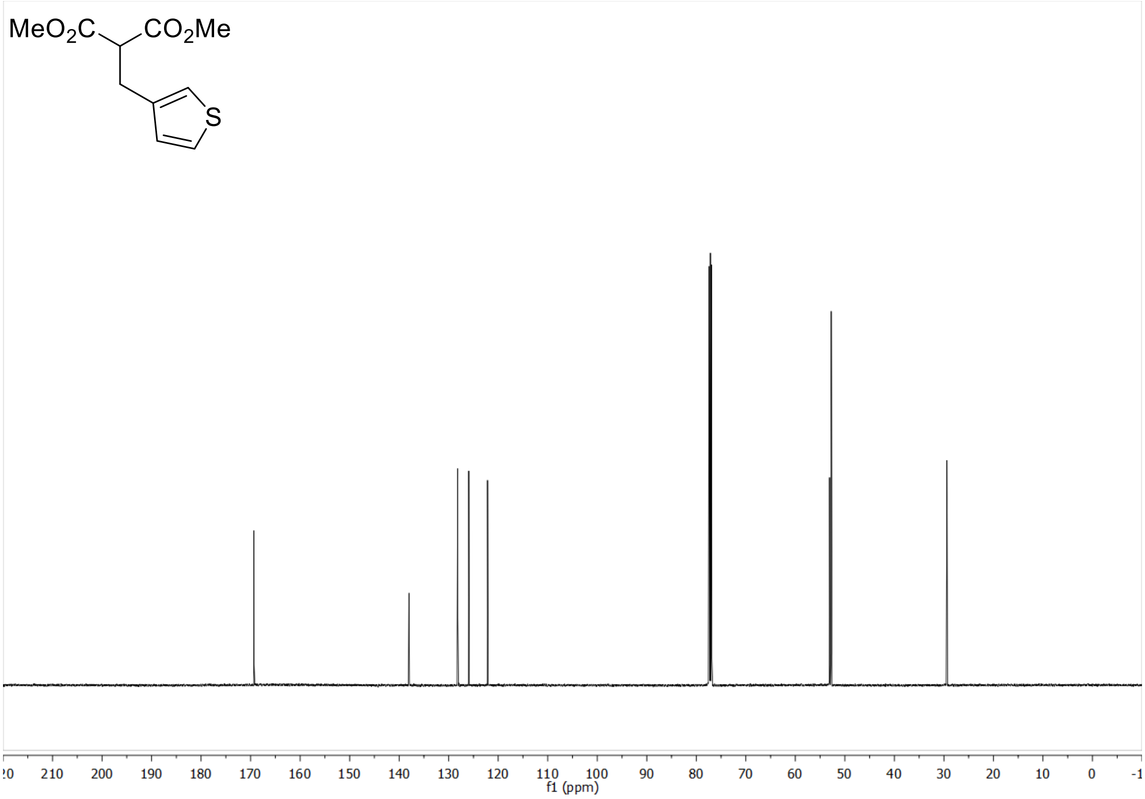

Supplementary Figure 53. ^{1}H NMR (400 MHz, $CDCl_3$) spectrum of **S29**

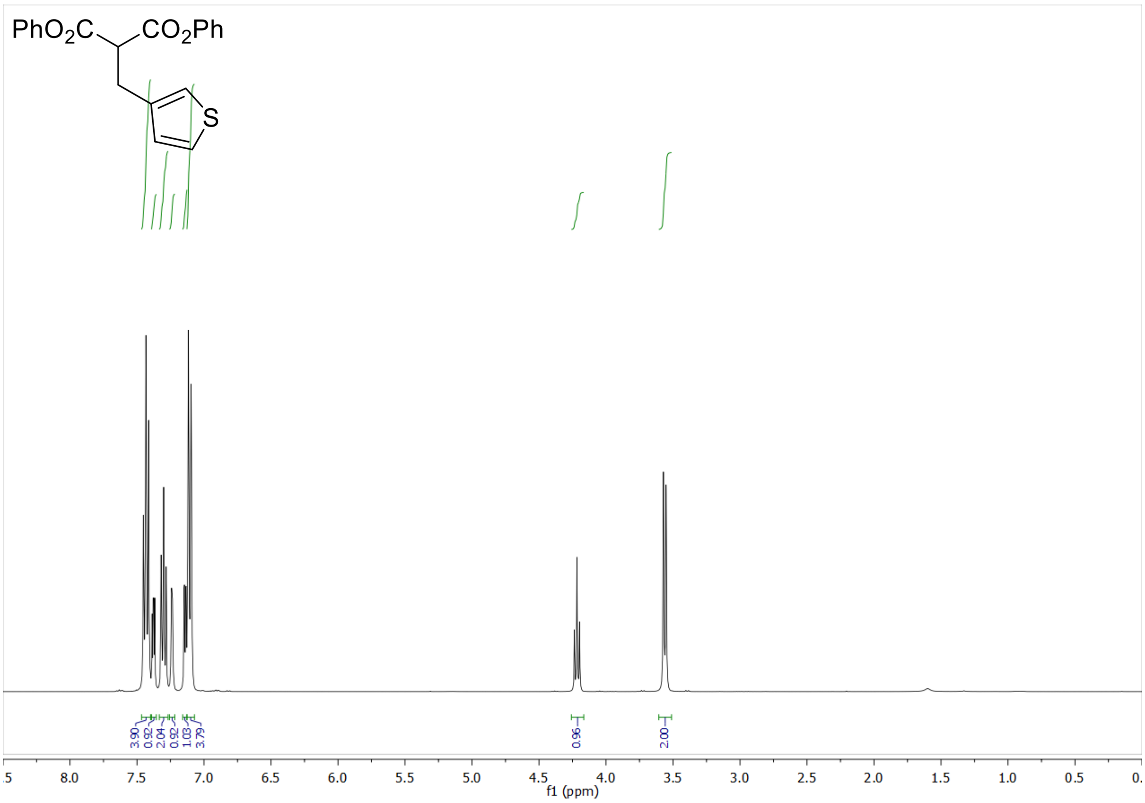

Supplementary Figure 54. ^{13}C NMR (100 MHz, $CDCl_3$) spectrum of **S29**

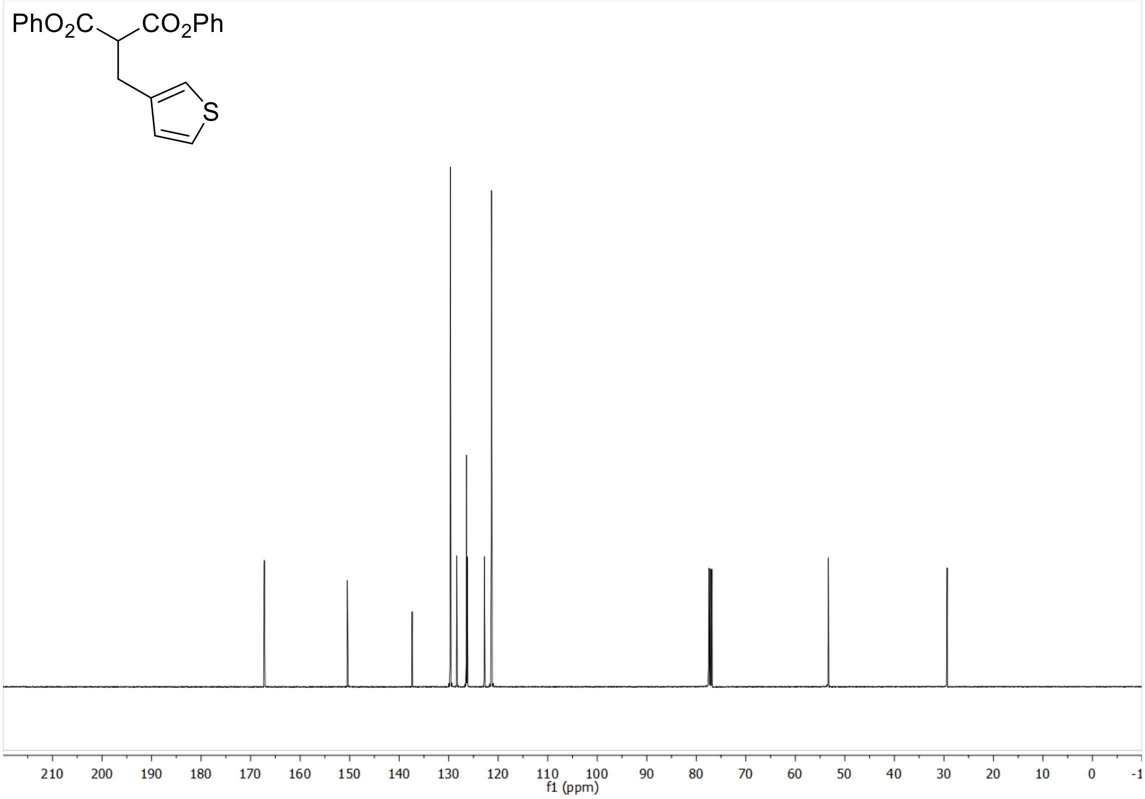

Supplementary Figure 55. ^{1}H NMR (400 MHz, $CDCl_3$) spectrum of **S31**

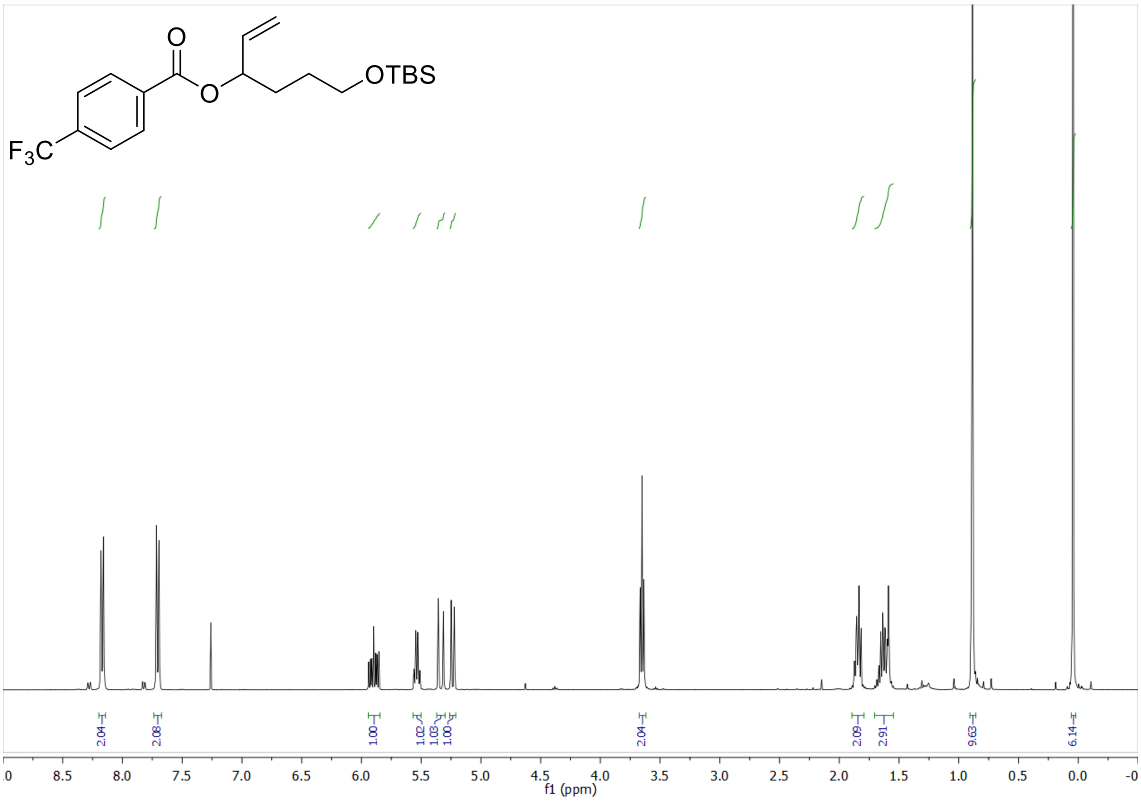

Supplementary Figure 56. ^{13}C NMR (100 MHz, $CDCl_3$) spectrum of **S31**

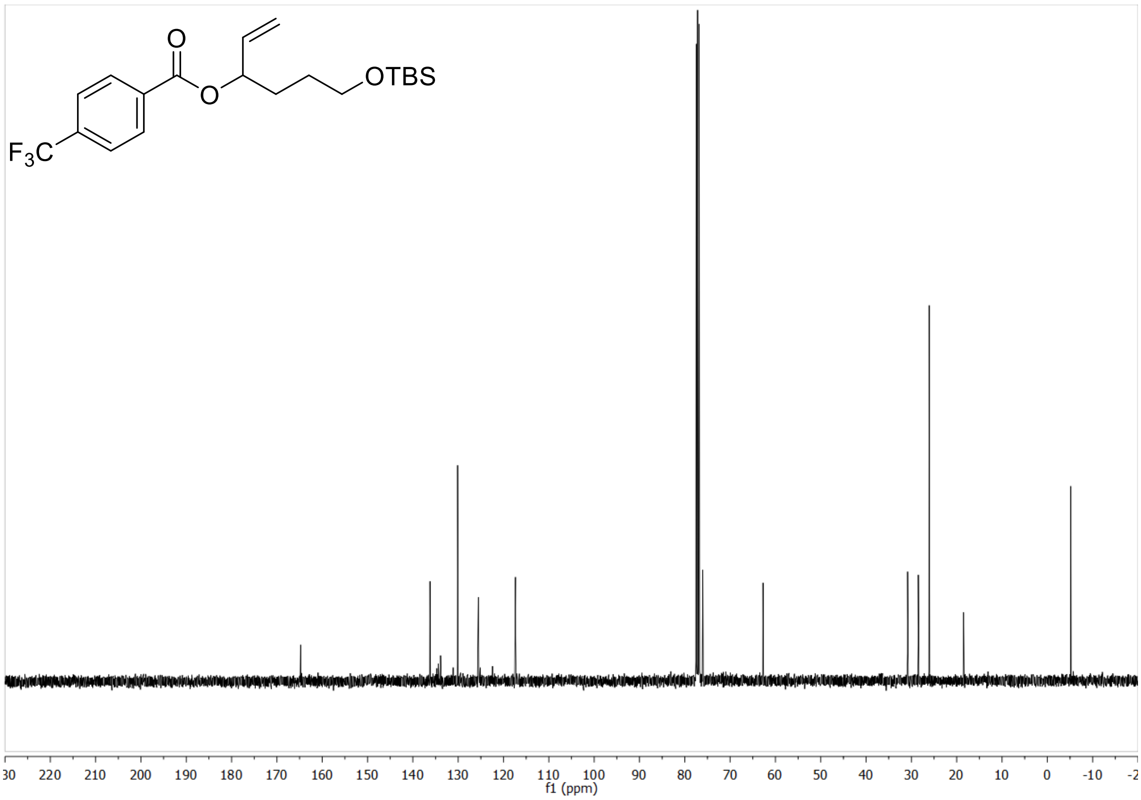

Supplementary Figure 57. ^{1}H NMR (400 MHz, $CDCl_3$) spectrum of **S33**

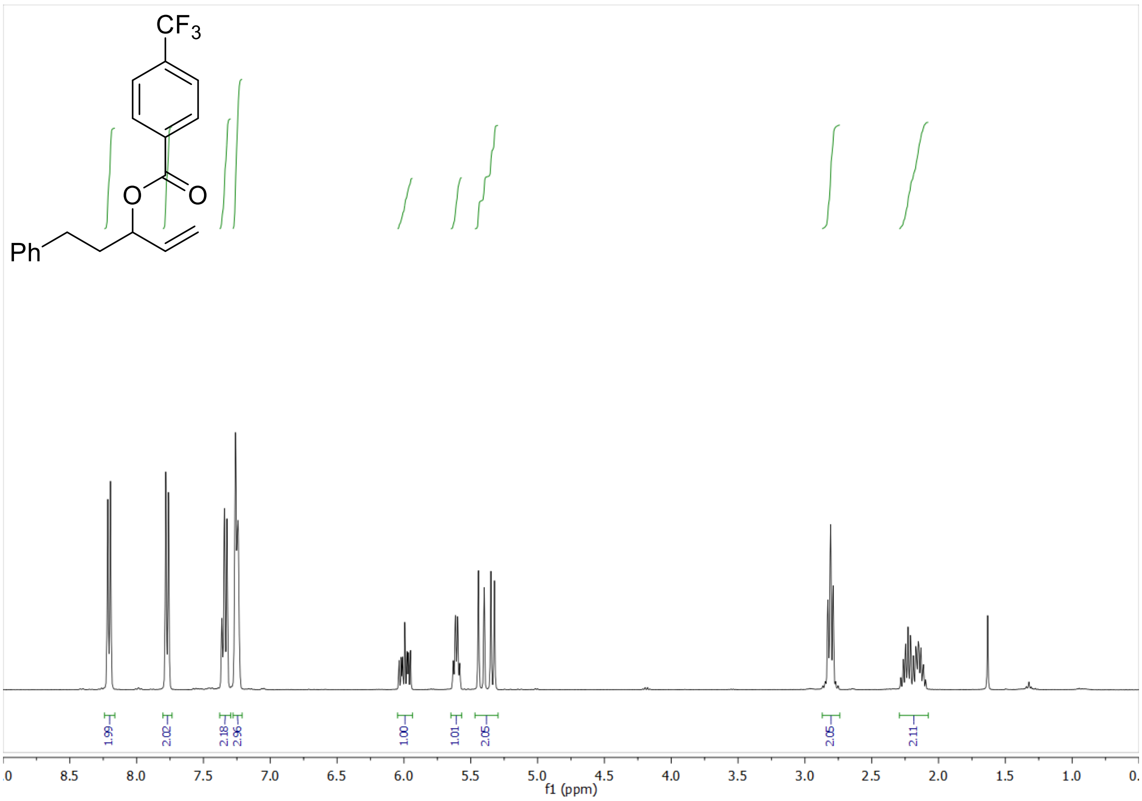

Supplementary Figure 58. ^{13}C NMR (100 MHz, $CDCl_3$) spectrum of **S33**

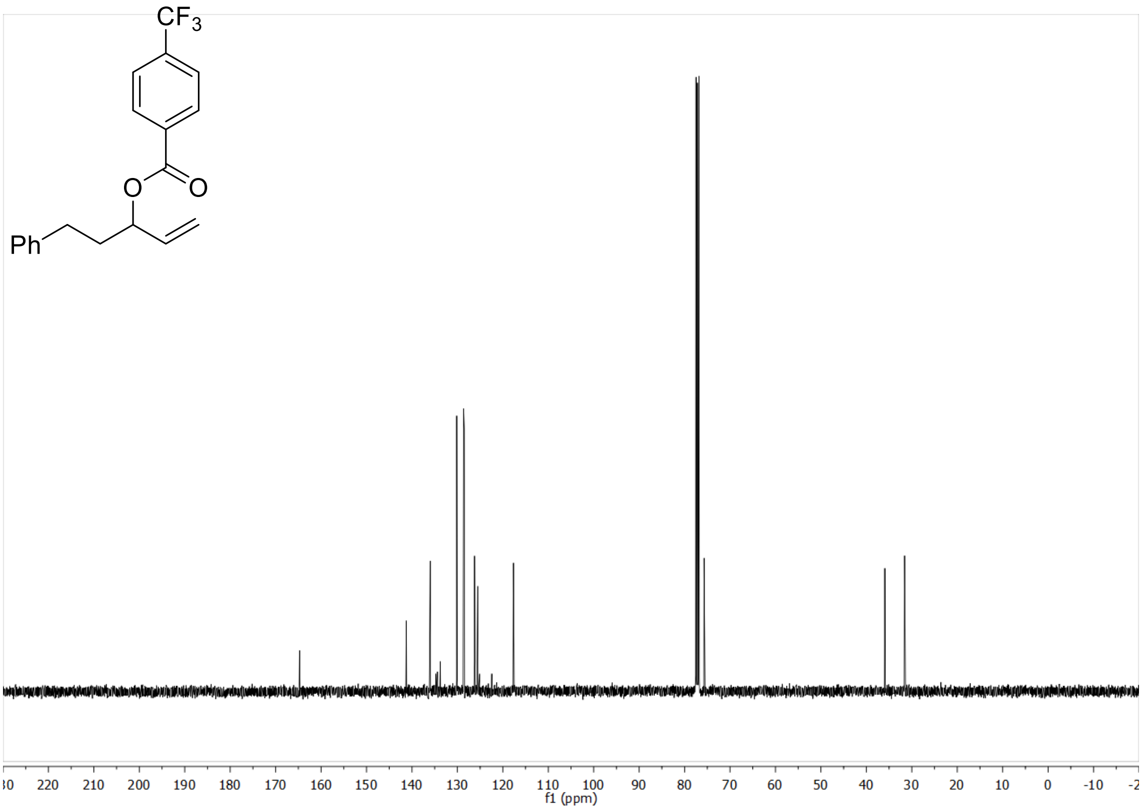

Supplementary Figure 59. ^{1}H NMR (400 MHz, $CDCl_3$) spectrum of **S35**

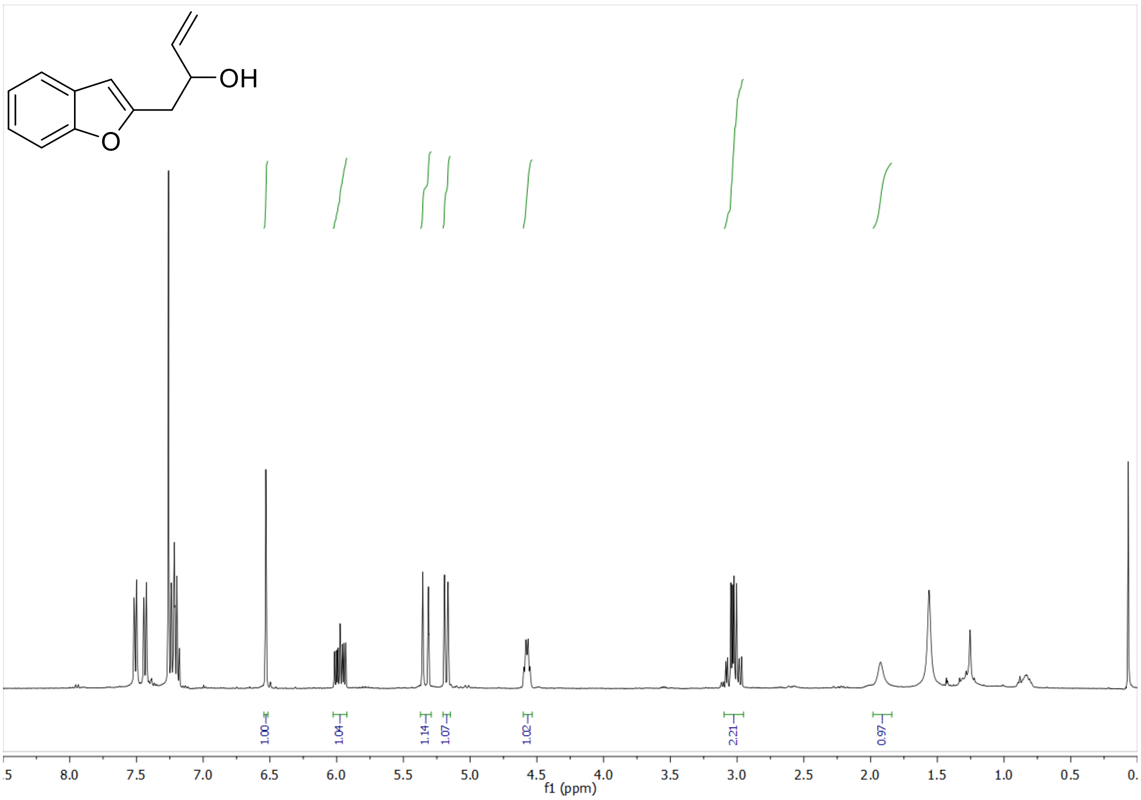

Supplementary Figure 60. ^{13}C NMR (100 MHz, $CDCl_3$) spectrum of **S35**

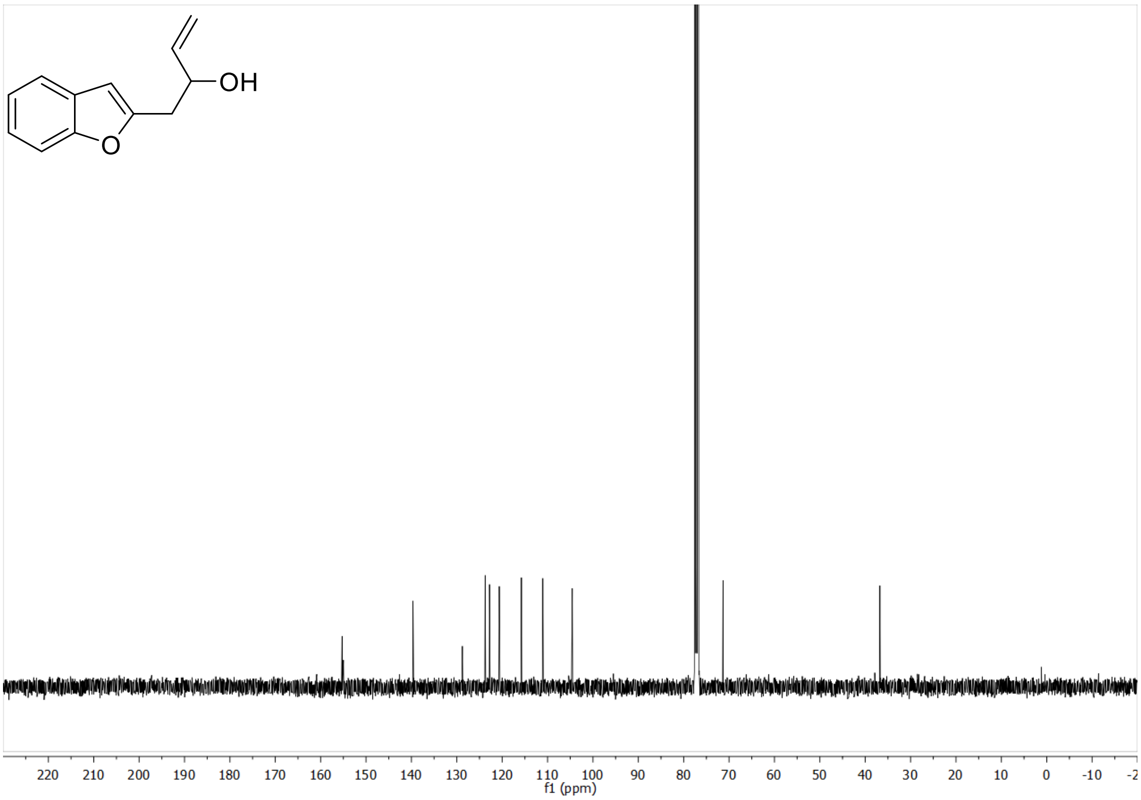

Supplementary Figure 61. ^{1}H NMR (400 MHz, $CDCl_3$) spectrum of **S36**

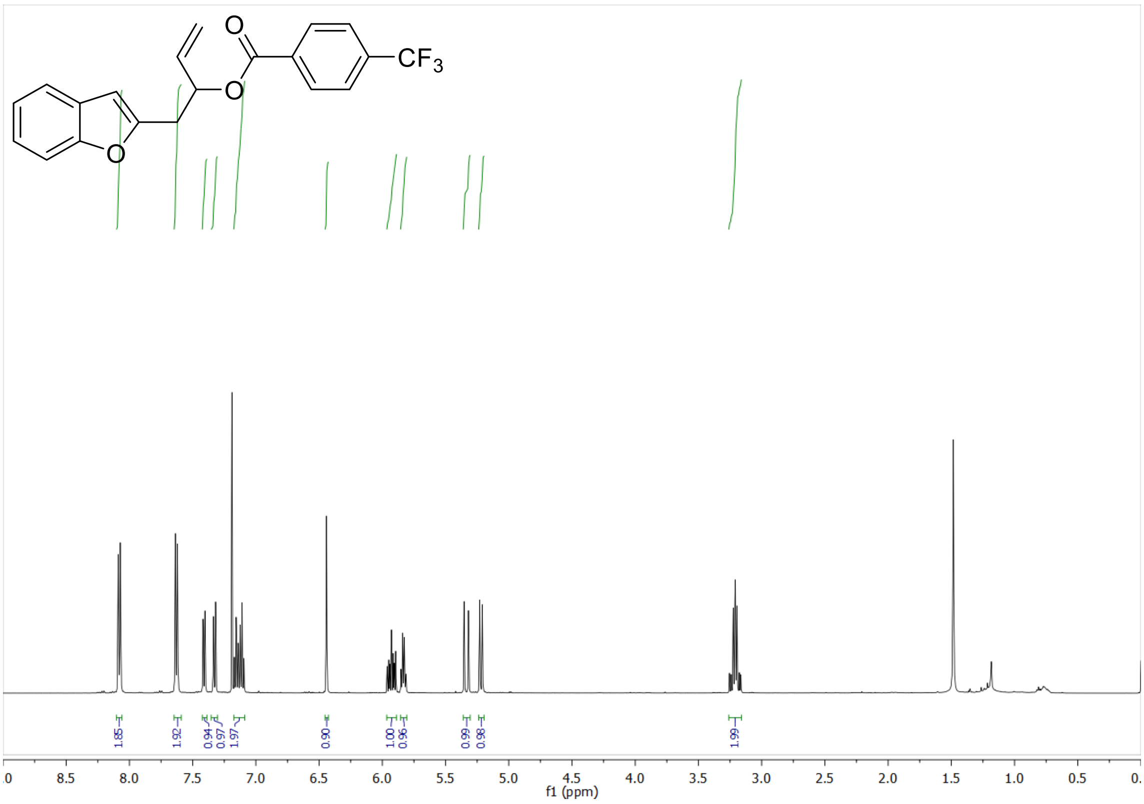

Supplementary Figure 62. ^{13}C NMR (100 MHz, $CDCl_3$) spectrum of **S36**

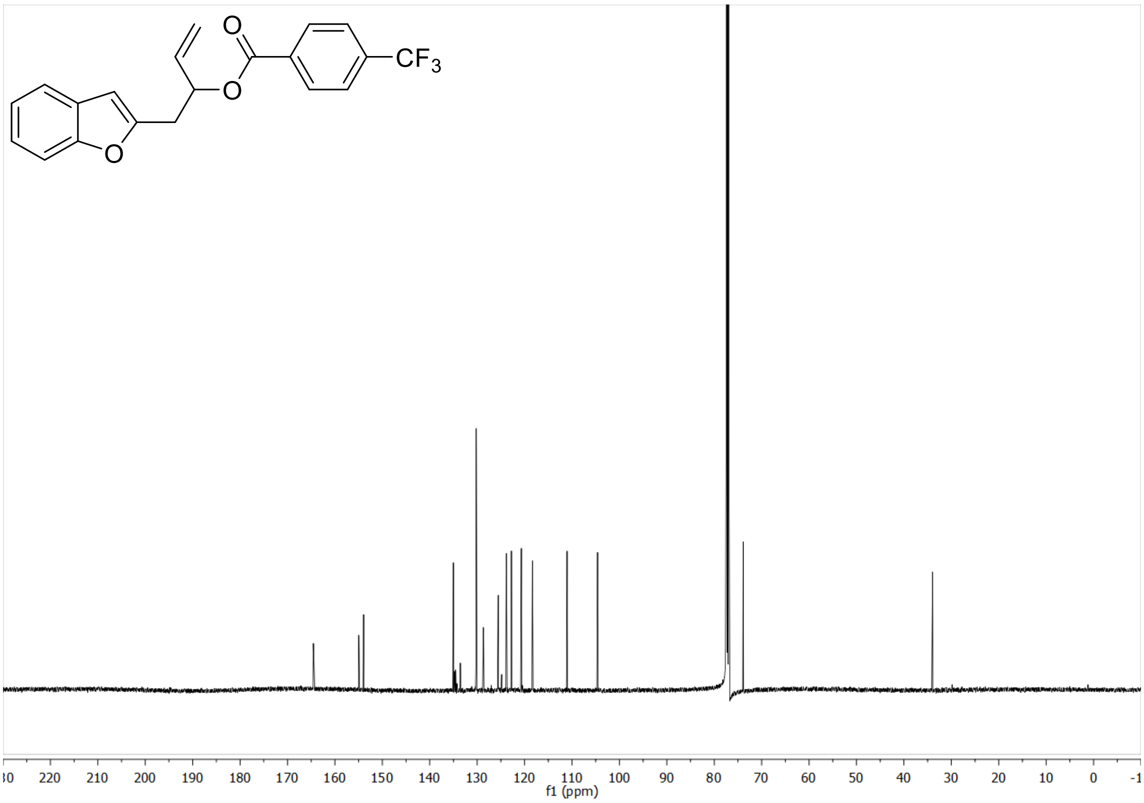

Supplementary Figure 63. ^{1}H NMR (400 MHz, CDCl$_3$) spectrum of **S37**

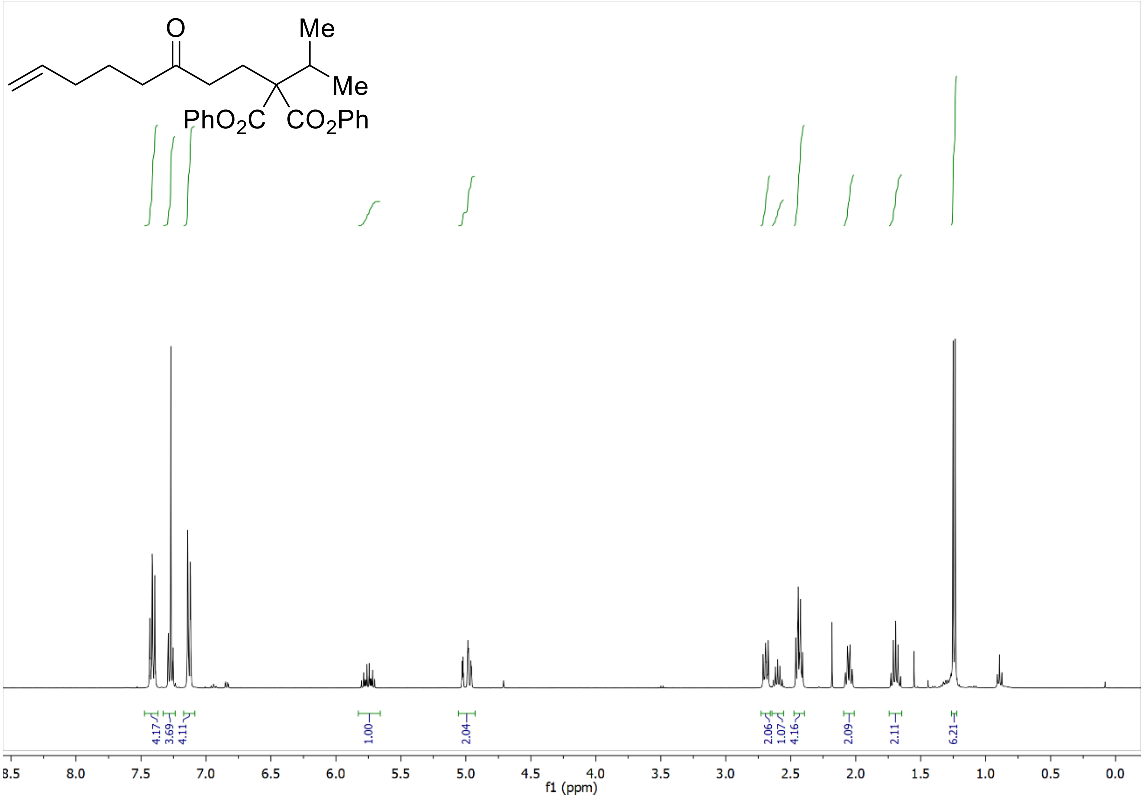

Supplementary Figure 64. ^{13}C NMR (100 MHz, CDCl$_3$) spectrum of **S37**

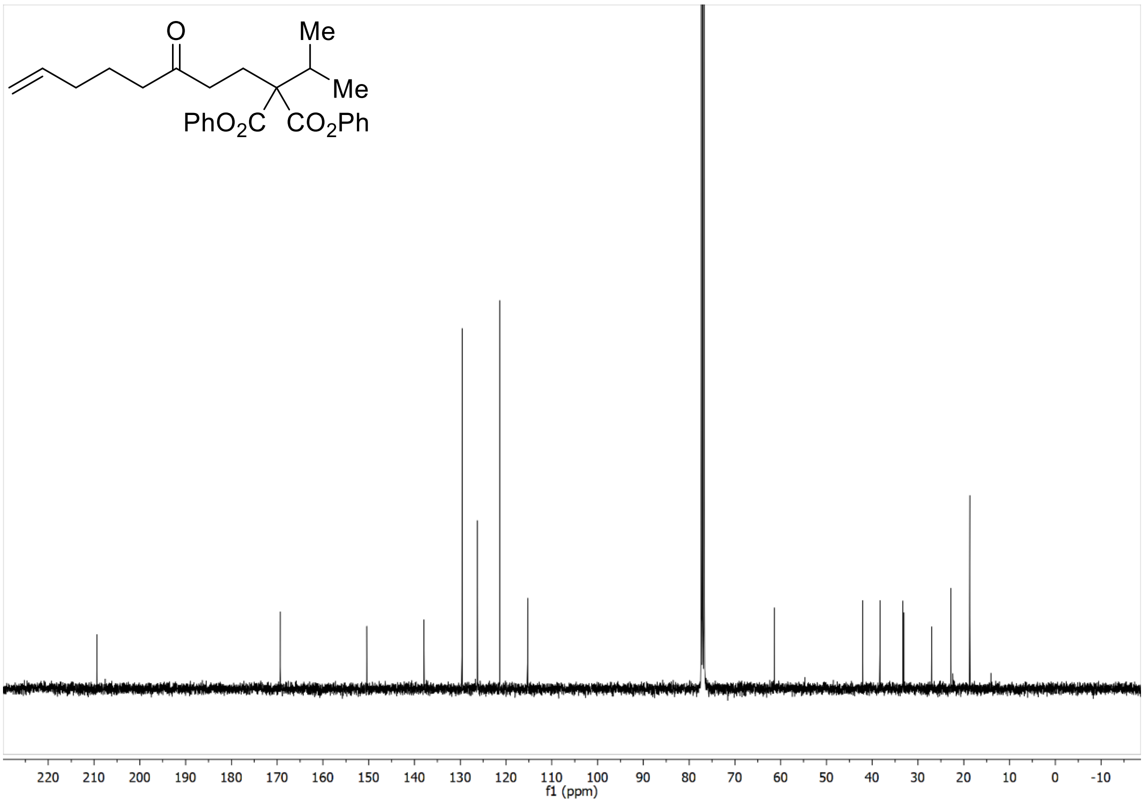

Supplementary Figure 65. ^{1}H NMR (400 MHz, $CDCl_3$) spectrum of **S38**

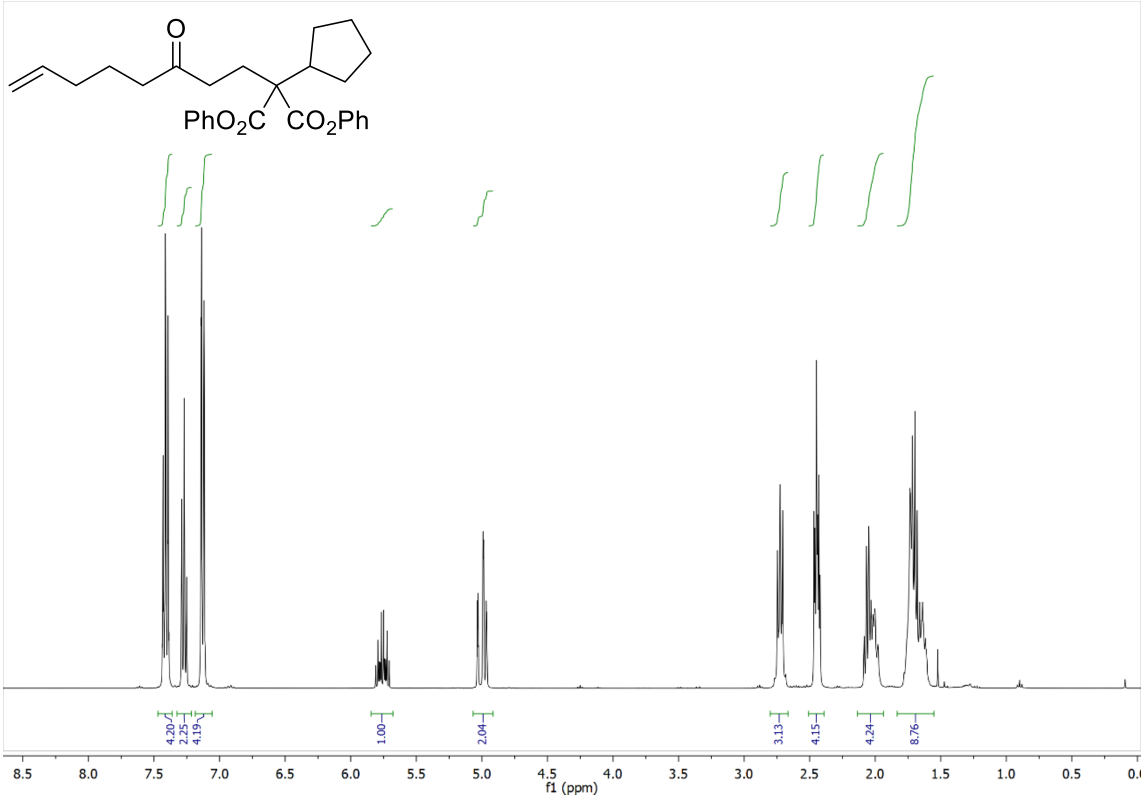

Supplementary Figure 66. ^{13}C NMR (100 MHz, $CDCl_3$) spectrum of **S38**

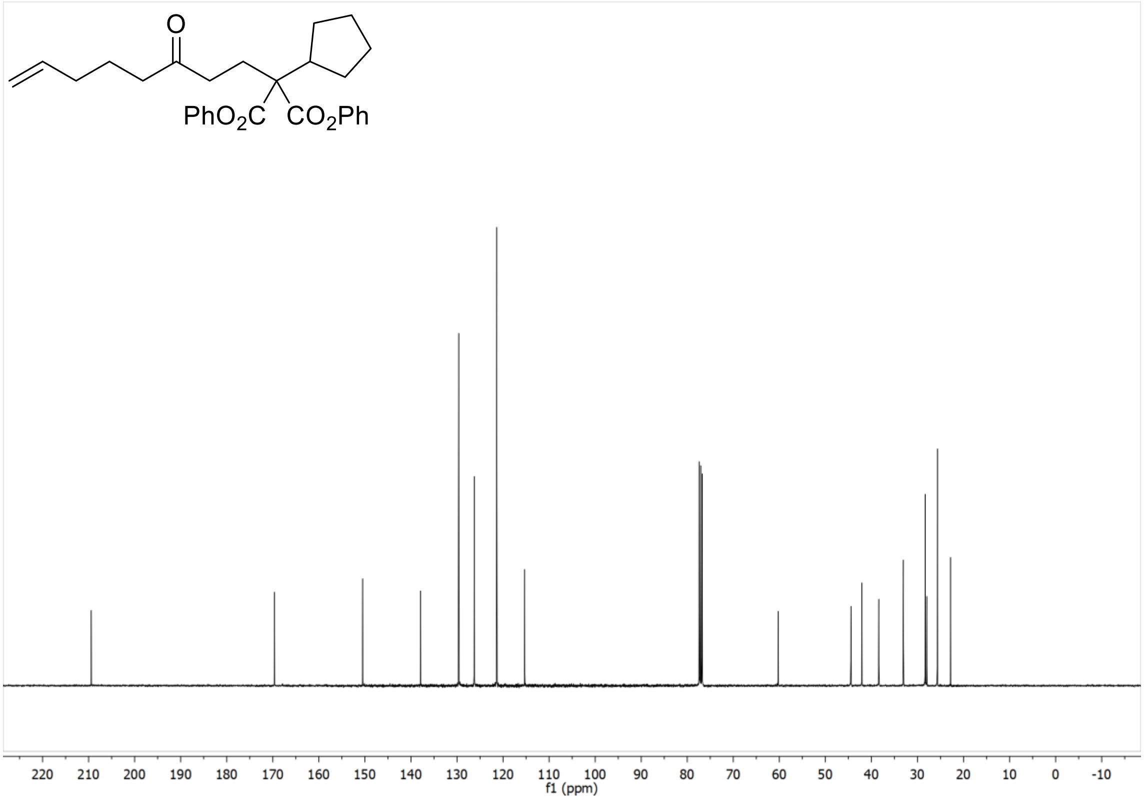

Supplementary Figure 67. ^{1}H NMR (400 MHz, $CDCl_3$) spectrum of **S39**

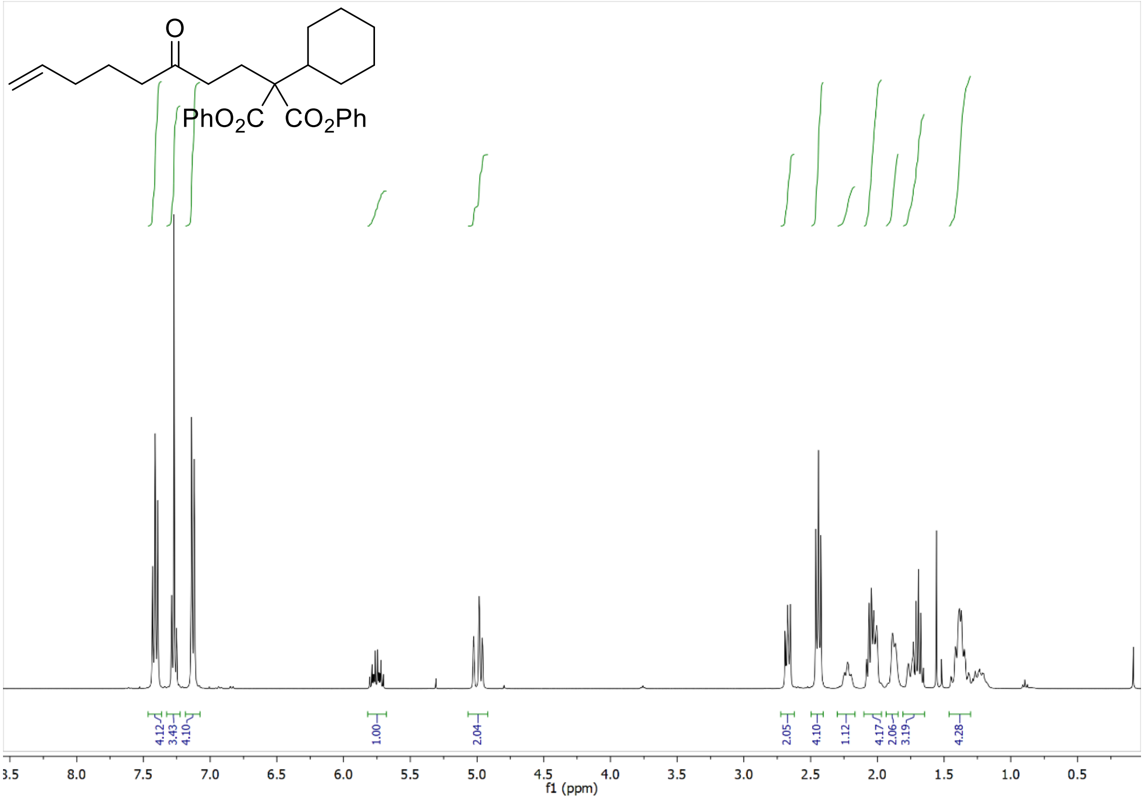

Supplementary Figure 68. ^{13}C NMR (100 MHz, $CDCl_3$) spectrum of **S39**

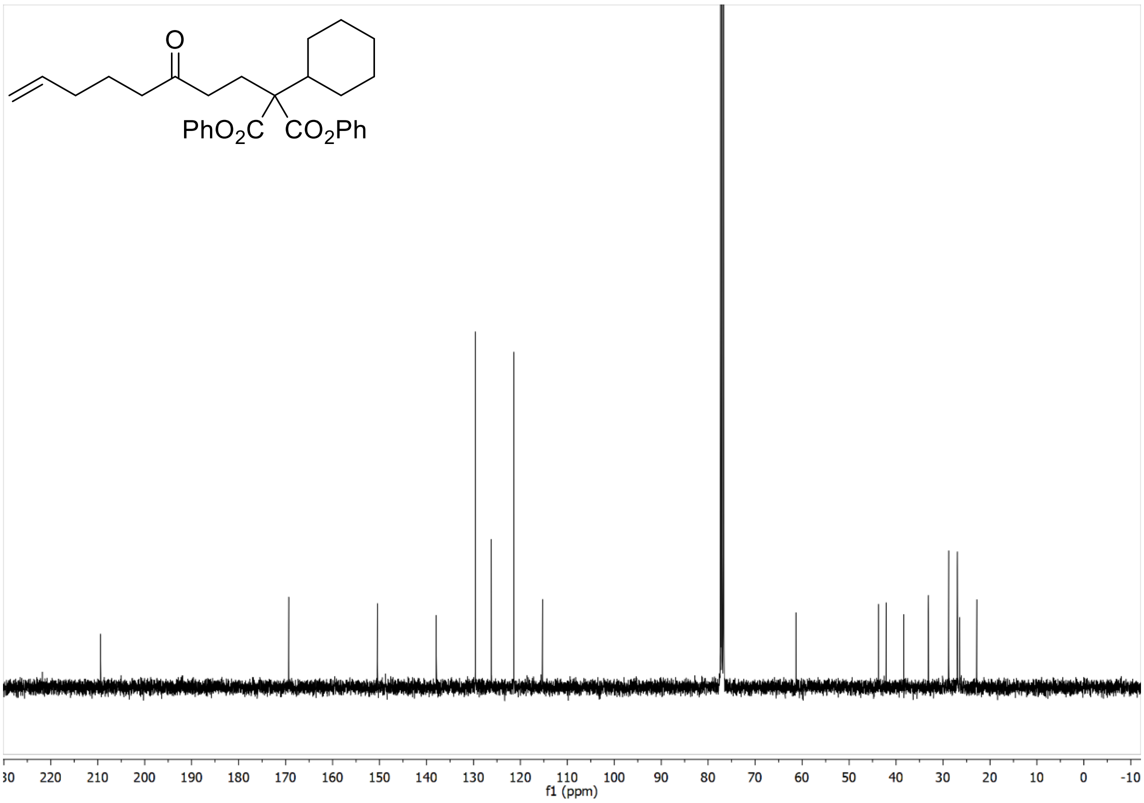

Supplementary Figure 69. ^{1}H NMR (400 MHz, $CDCl_3$) spectrum of **S40**

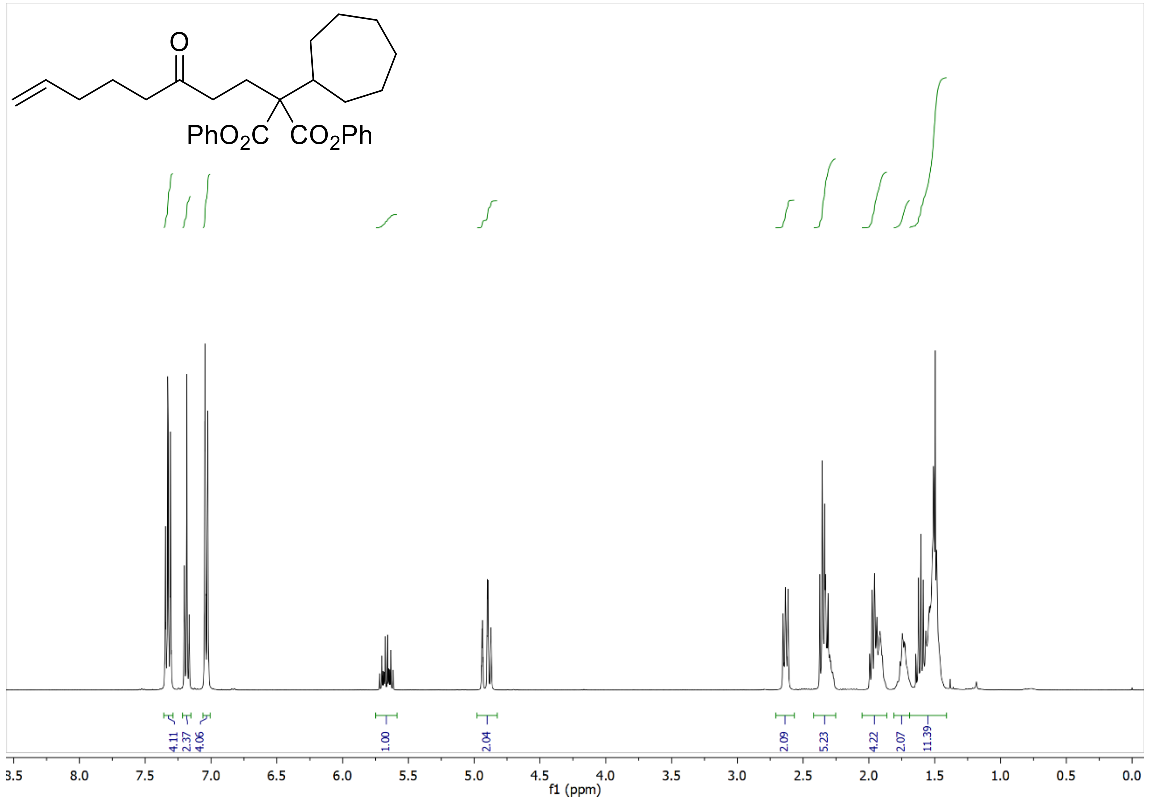

Supplementary Figure 70. ^{13}C NMR (100 MHz, $CDCl_3$) spectrum of **S40**

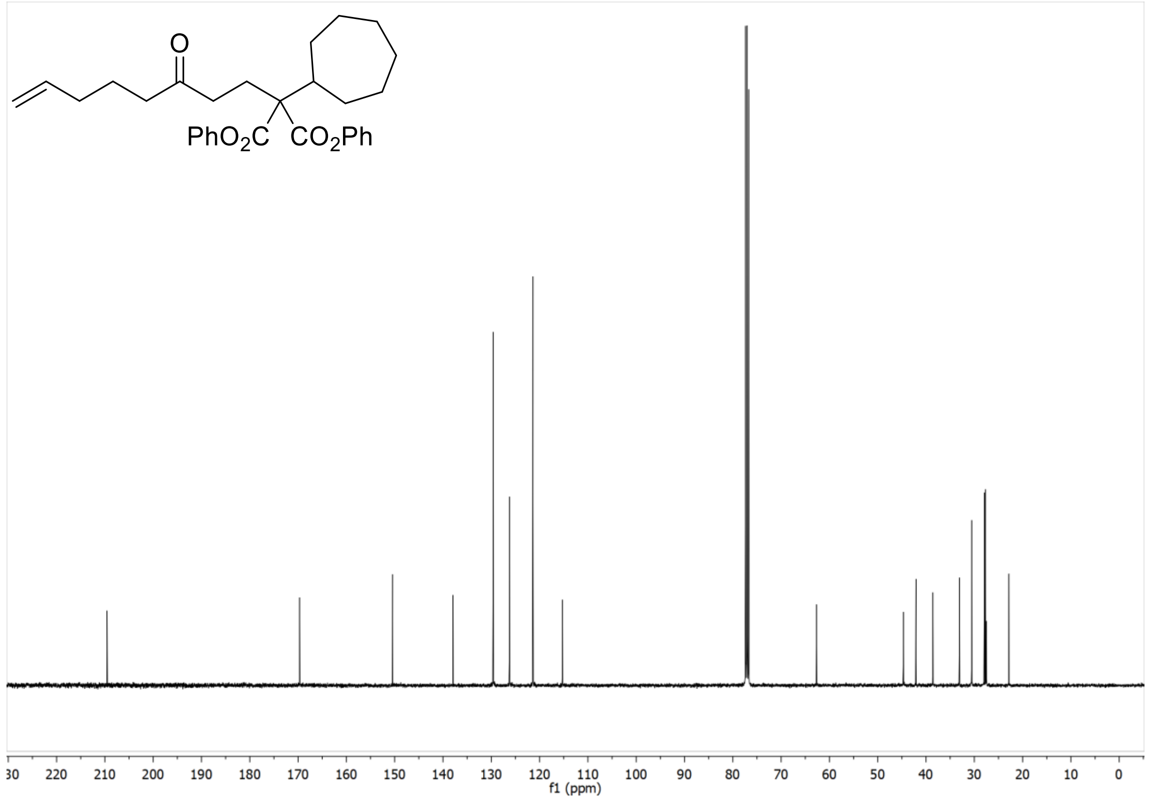

Supplementary Figure 71. ^{1}H NMR (400 MHz, $CDCl_3$) spectrum of **S41**

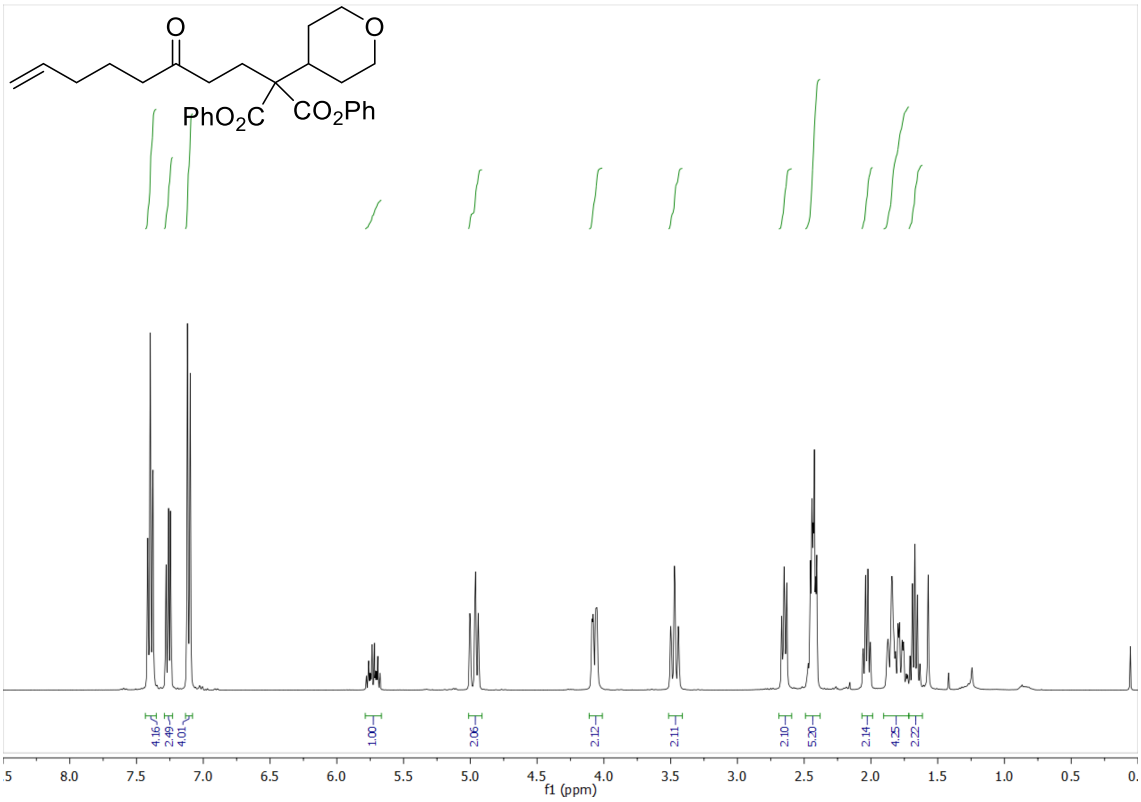

Supplementary Figure 72. ^{13}C NMR (100 MHz, $CDCl_3$) spectrum of **S41**

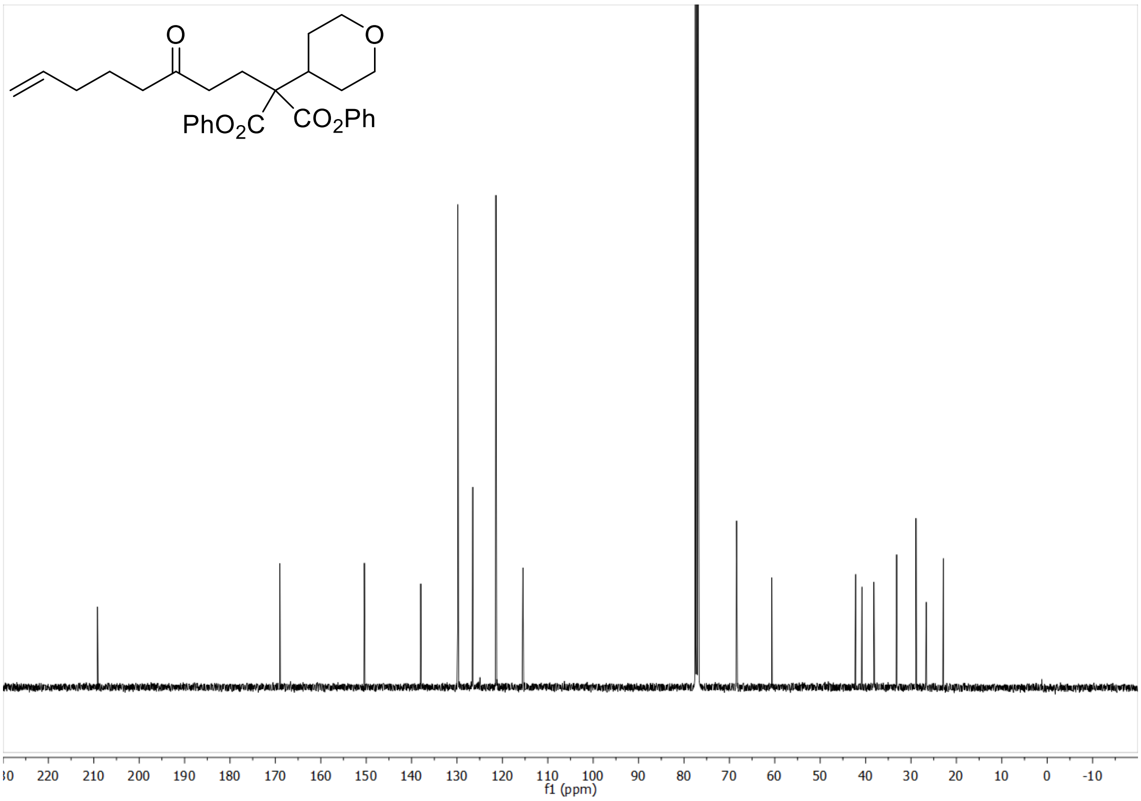

Supplementary Figure 73. ^{1}H NMR (400 MHz, $CDCl_3$) spectrum of **S42**

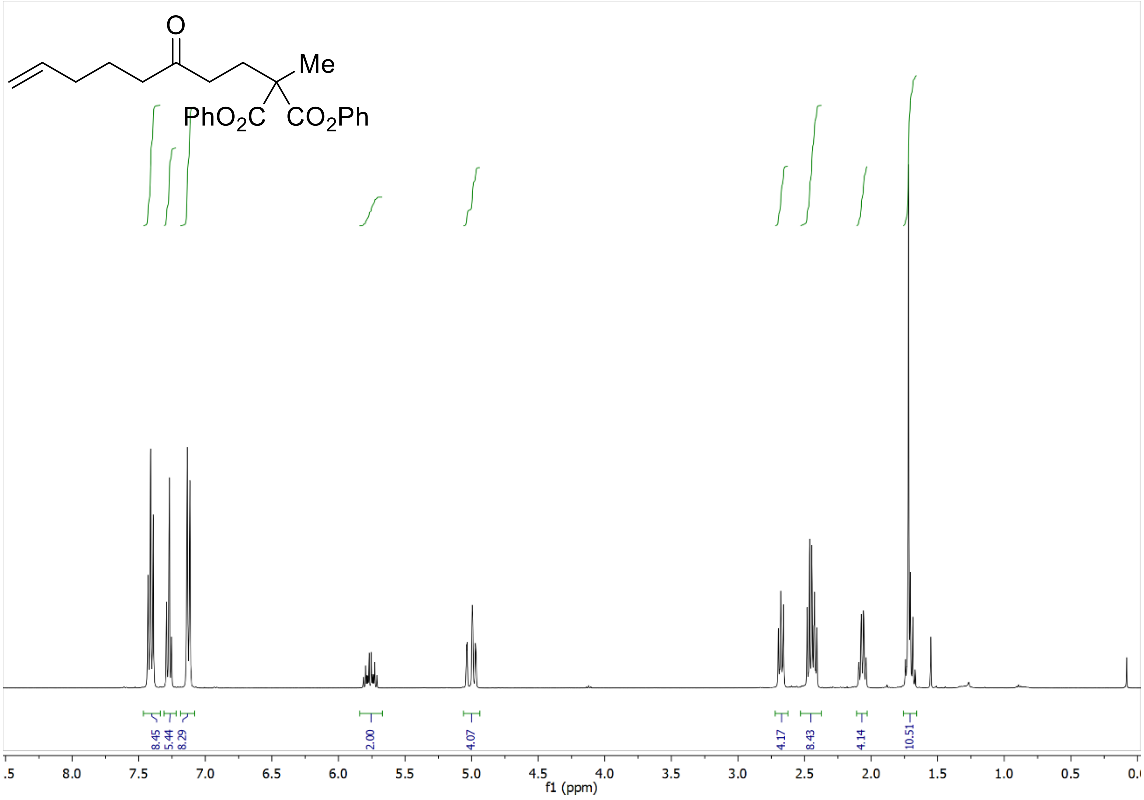

Supplementary Figure 74. ^{13}C NMR (100 MHz, $CDCl_3$) spectrum of **S42**

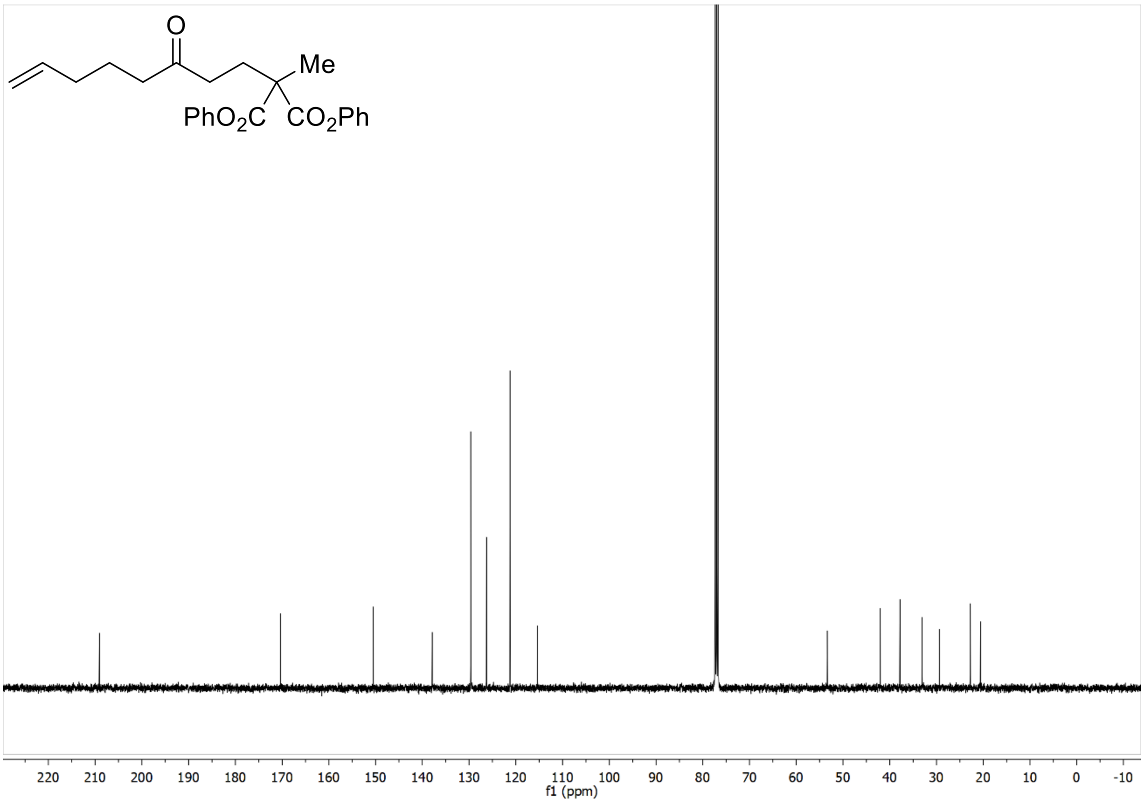

Supplementary Figure 75. ^{1}H NMR (400 MHz, $CDCl_3$) spectrum of **S43**

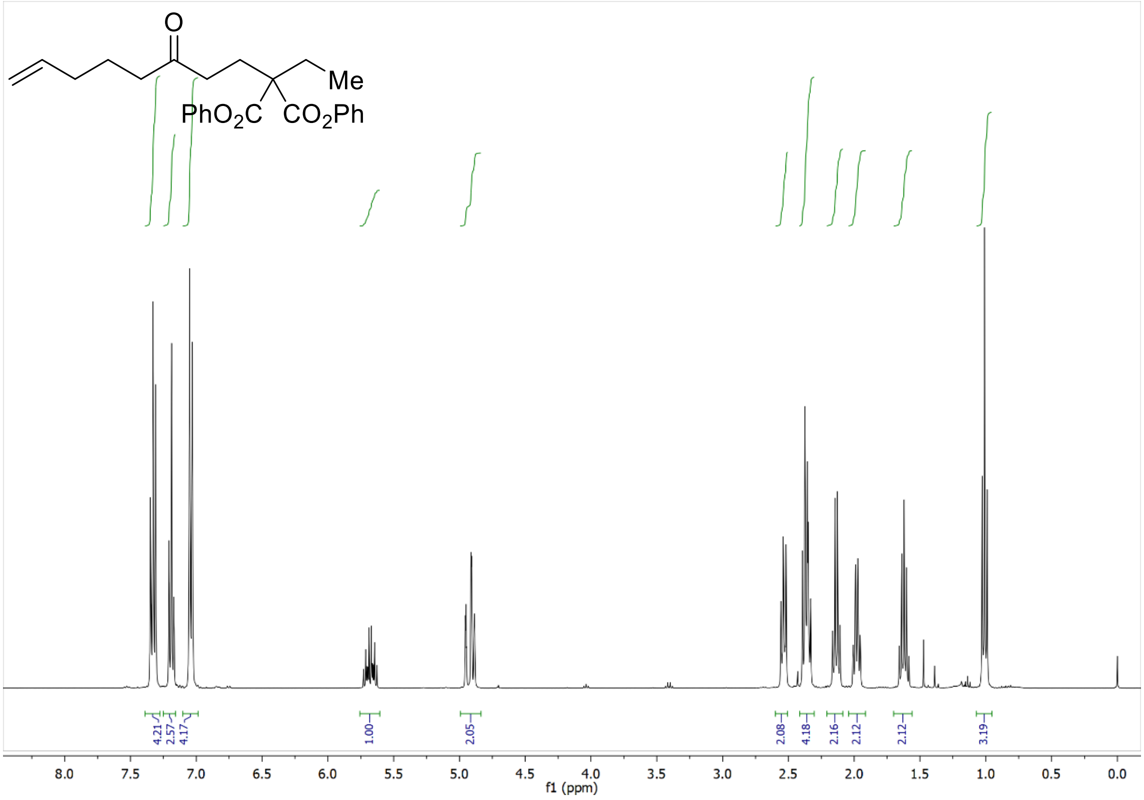

Supplementary Figure 76. ^{13}C NMR (100 MHz, $CDCl_3$) spectrum of **S43**

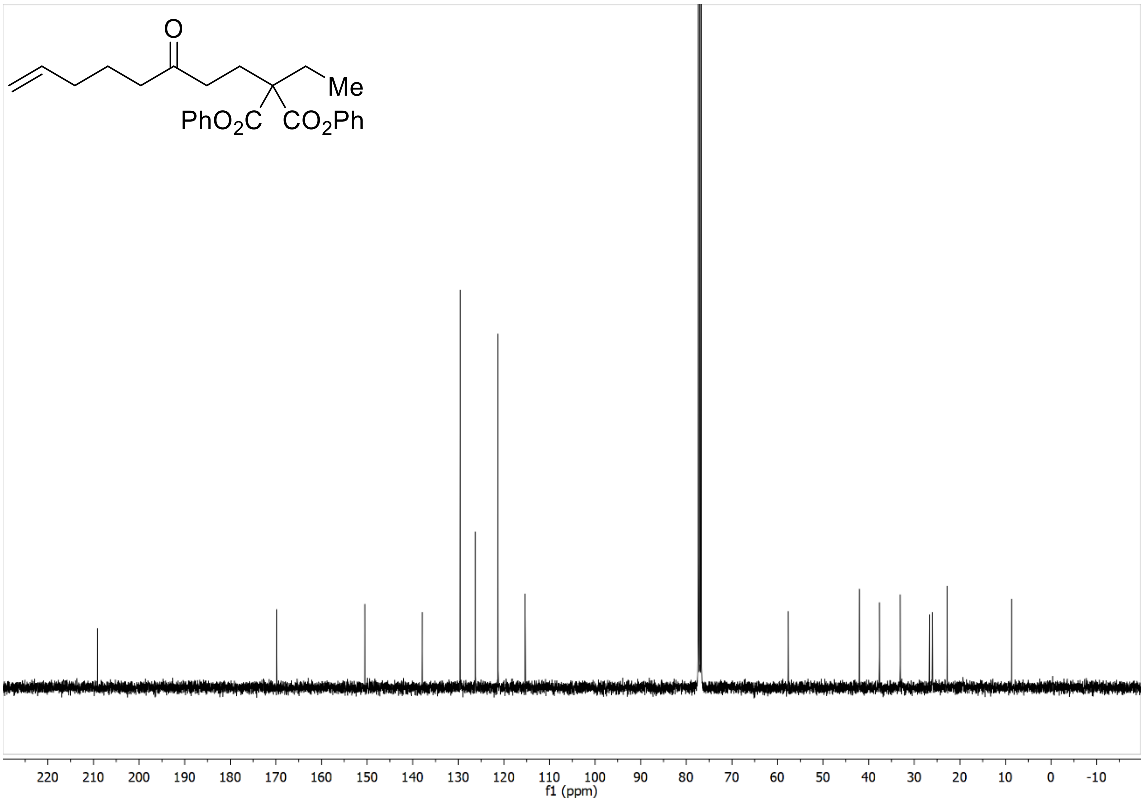

Supplementary Figure 77. ^{1}H NMR (400 MHz, $CDCl_3$) spectrum of **S44**

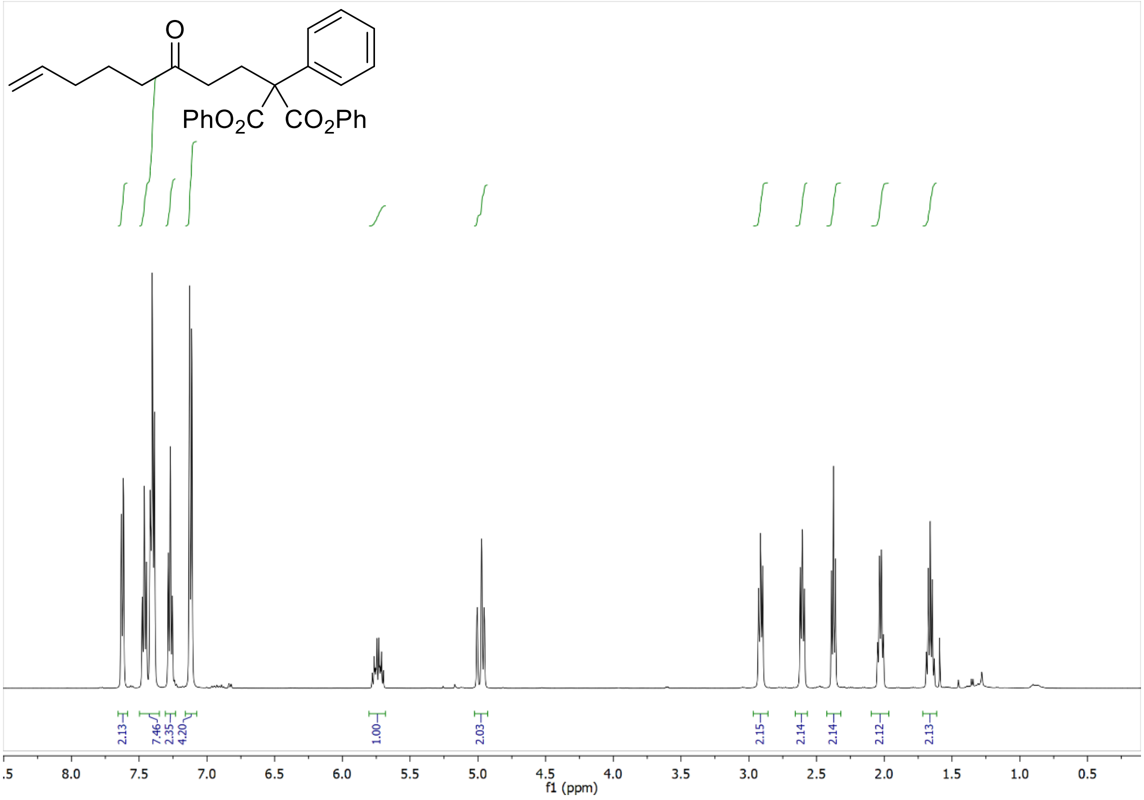

Supplementary Figure 78. ^{13}C NMR (100 MHz, $CDCl_3$) spectrum of **S44**

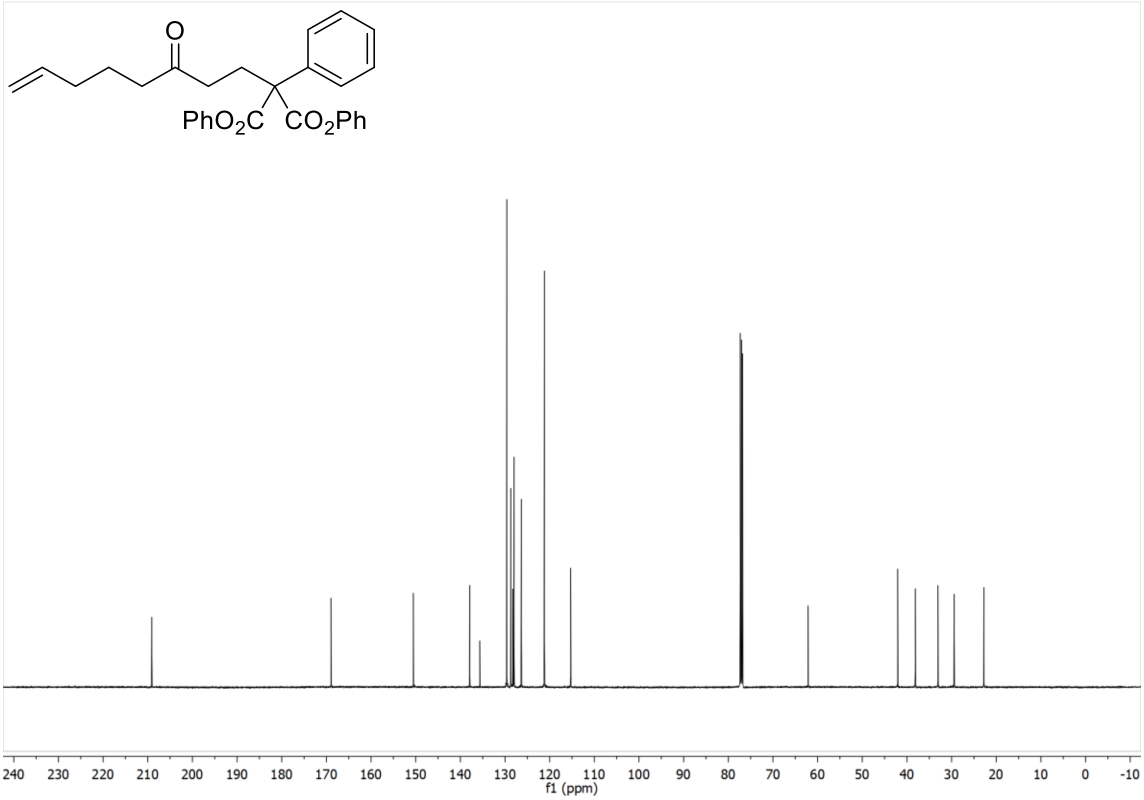

Supplementary Figure 79. ^{1}H NMR (400 MHz, $CDCl_3$) spectrum of **S45**

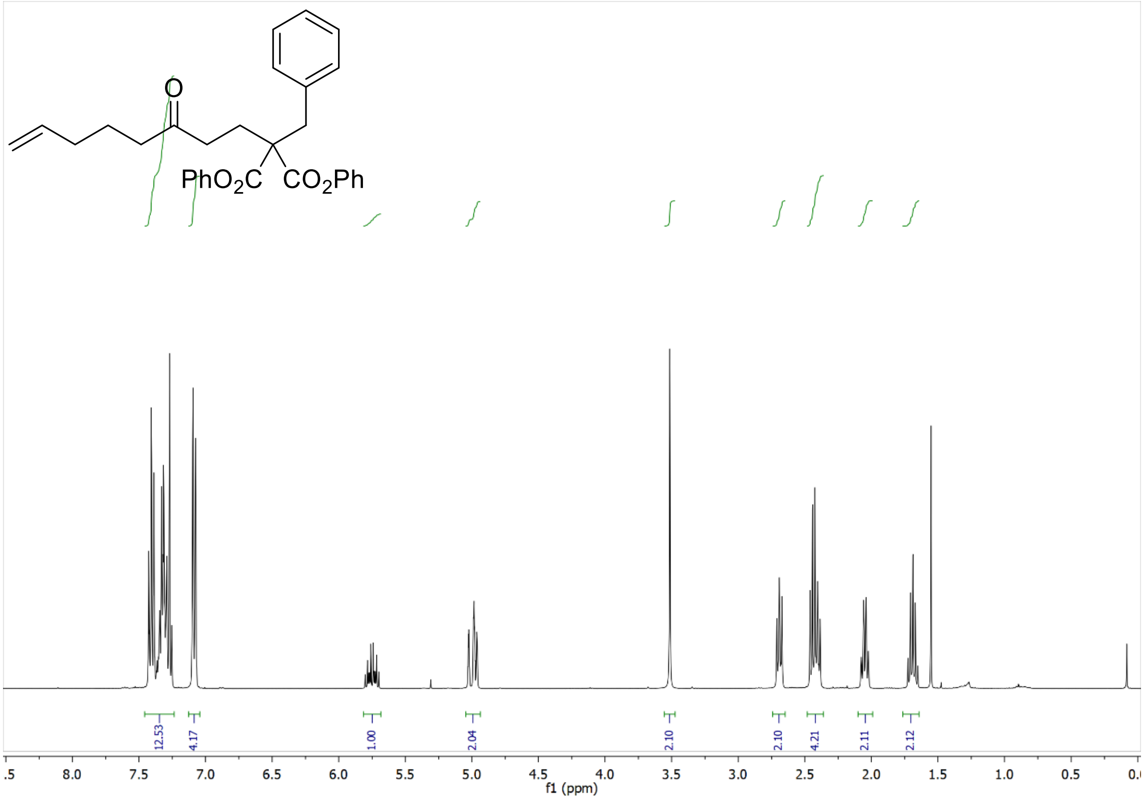

Supplementary Figure 80. ^{13}C NMR (100 MHz, $CDCl_3$) spectrum of **S45**

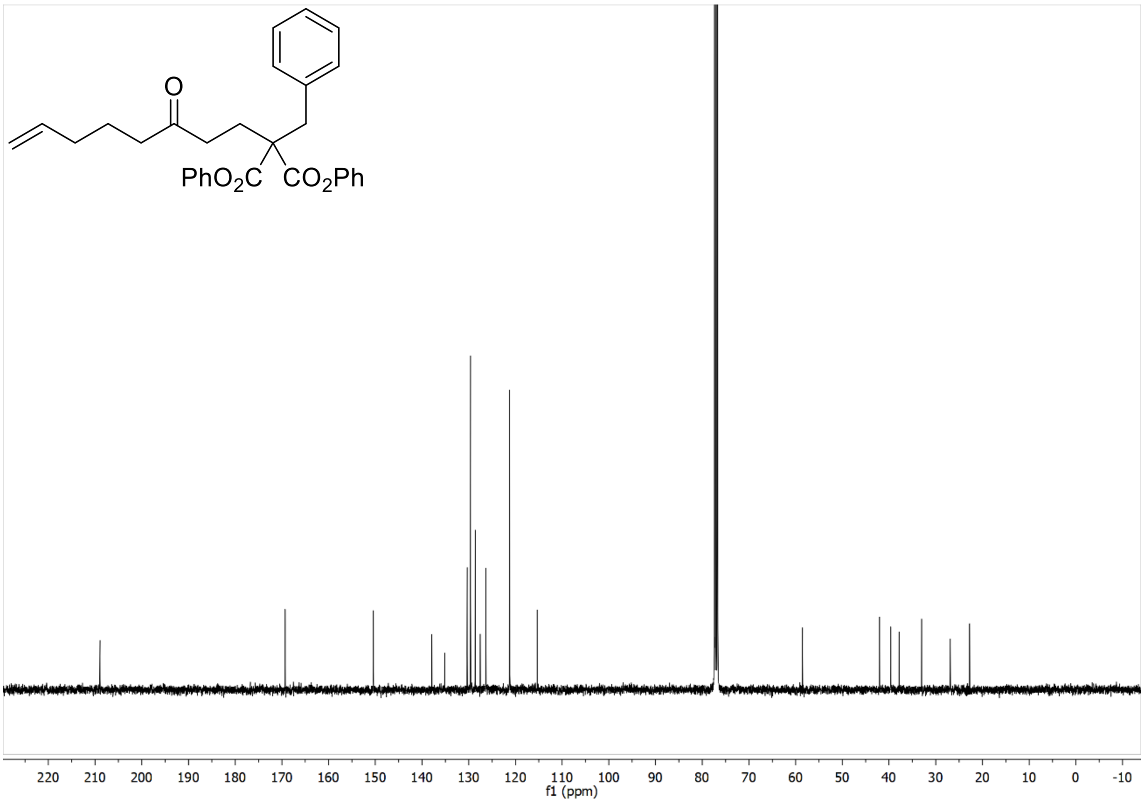

Supplementary Figure 81. ^{1}H NMR (400 MHz, $CDCl_3$) spectrum of **S46**

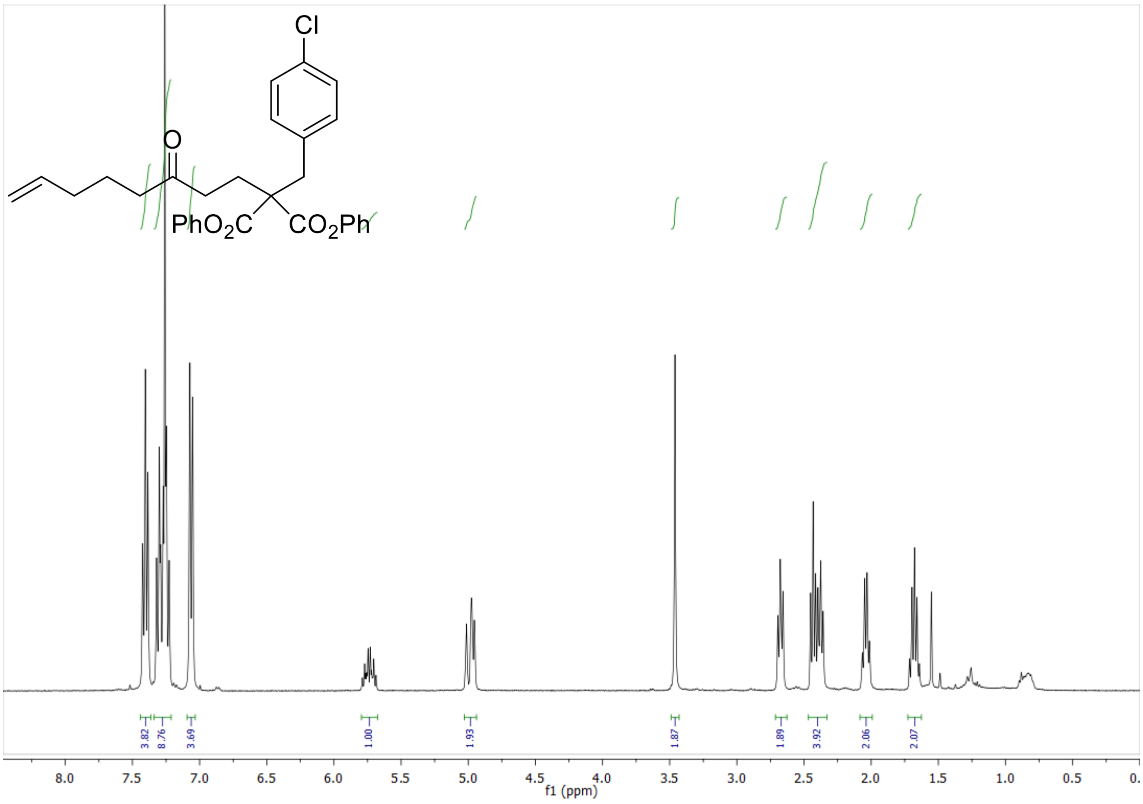

Supplementary Figure 82. ^{13}C NMR (100 MHz, $CDCl_3$) spectrum of **S46**

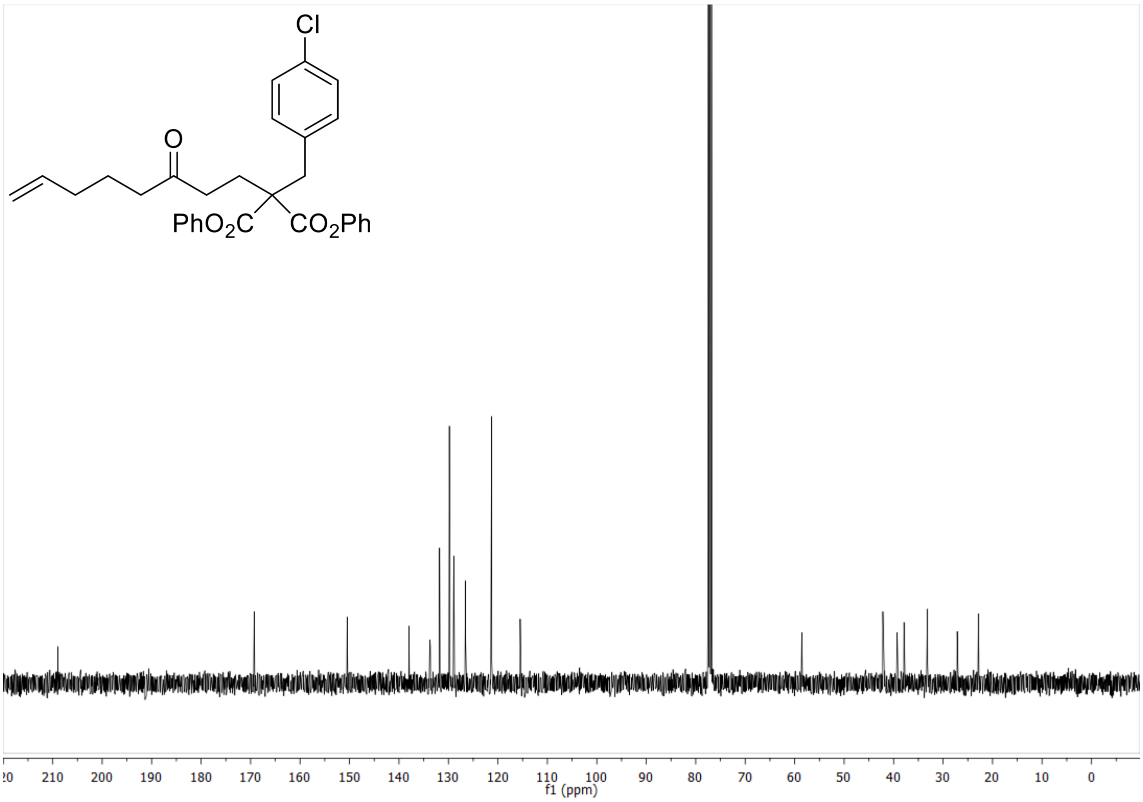

Supplementary Figure 83. ^{1}H NMR (500 MHz, $CDCl_3$) spectrum of **S47**

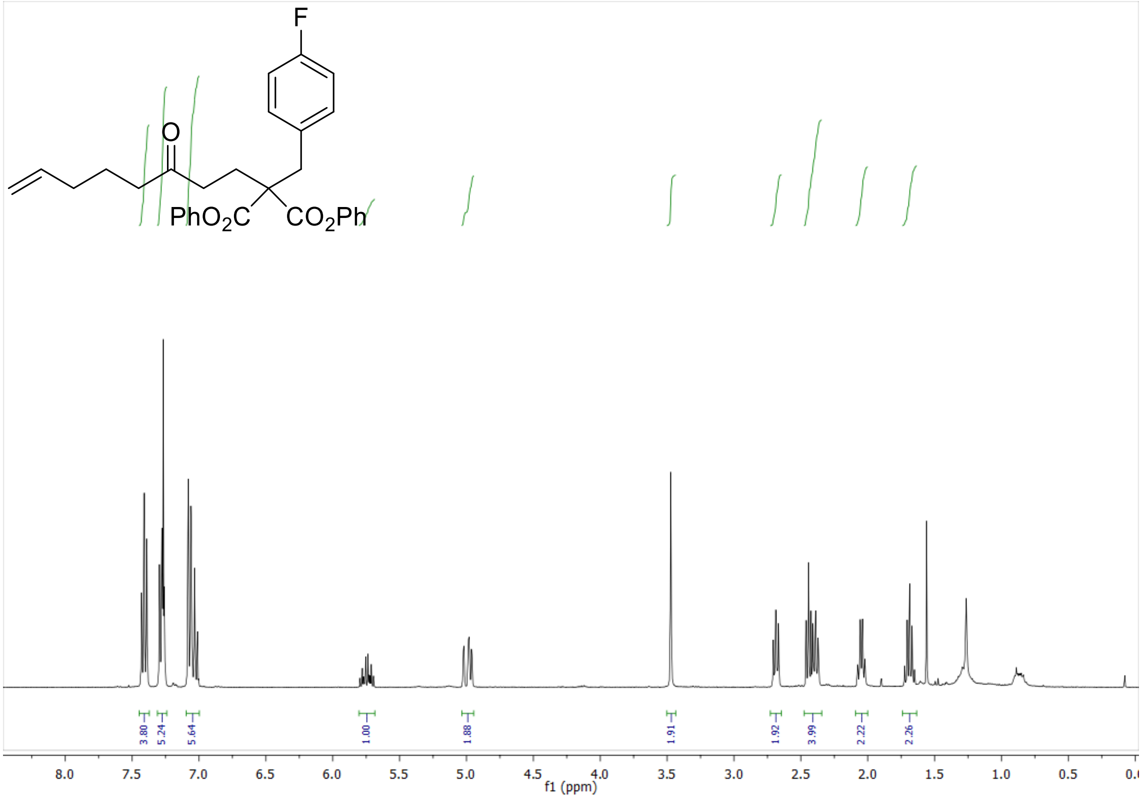

Supplementary Figure 84. ^{13}C NMR (125 MHz, $CDCl_3$) spectrum of **S47**

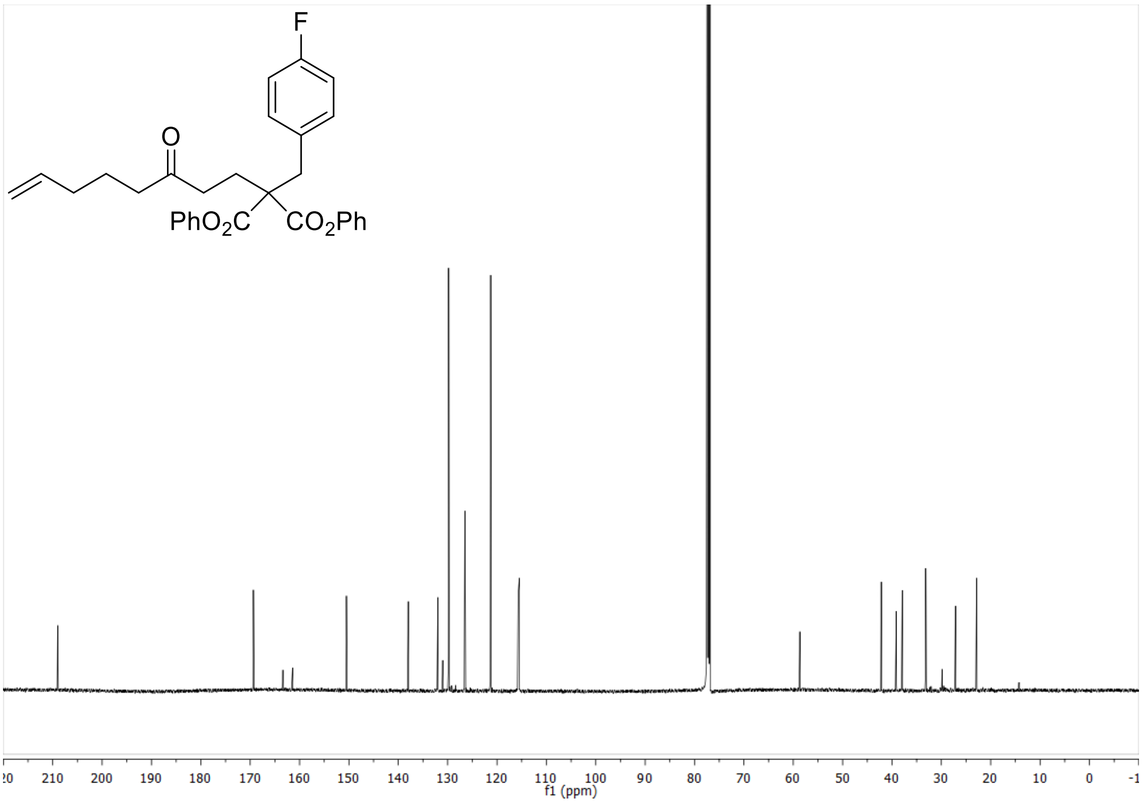

Supplementary Figure 85. ^{1}H NMR (500 MHz, $CDCl_3$) spectrum of **S48**

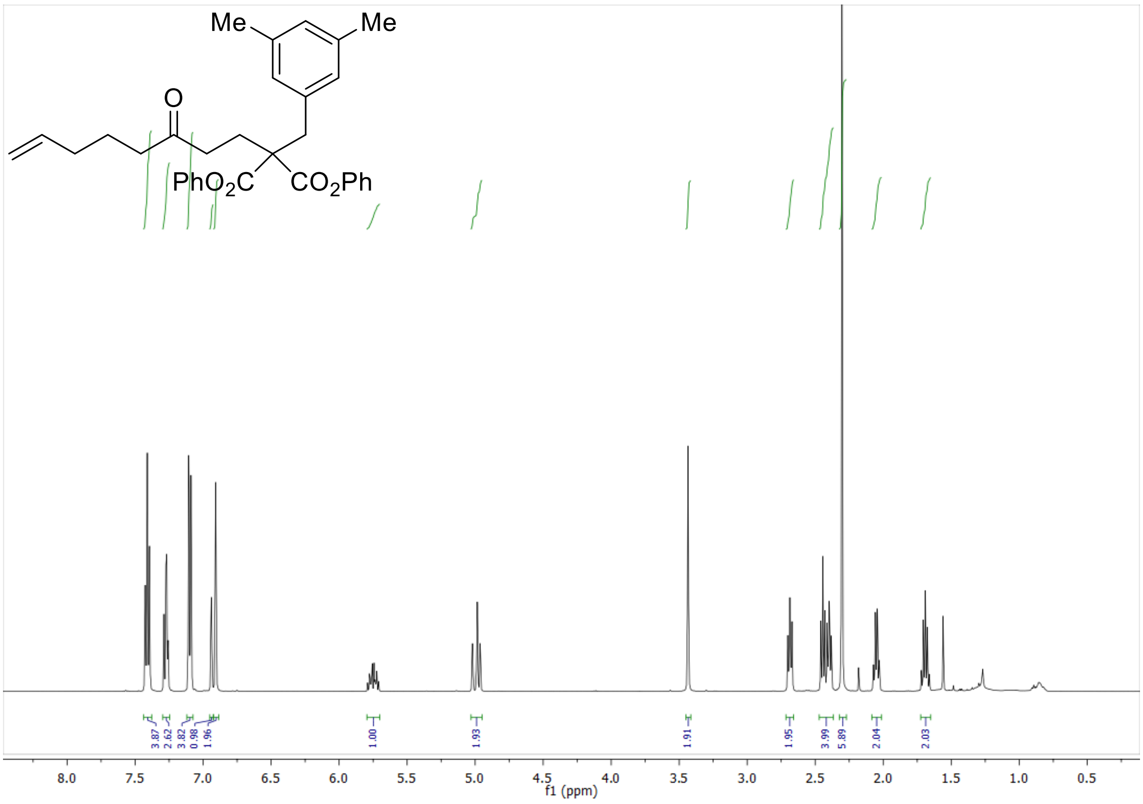

Supplementary Figure 86. ^{13}C NMR (125 MHz, $CDCl_3$) spectrum of **S48**

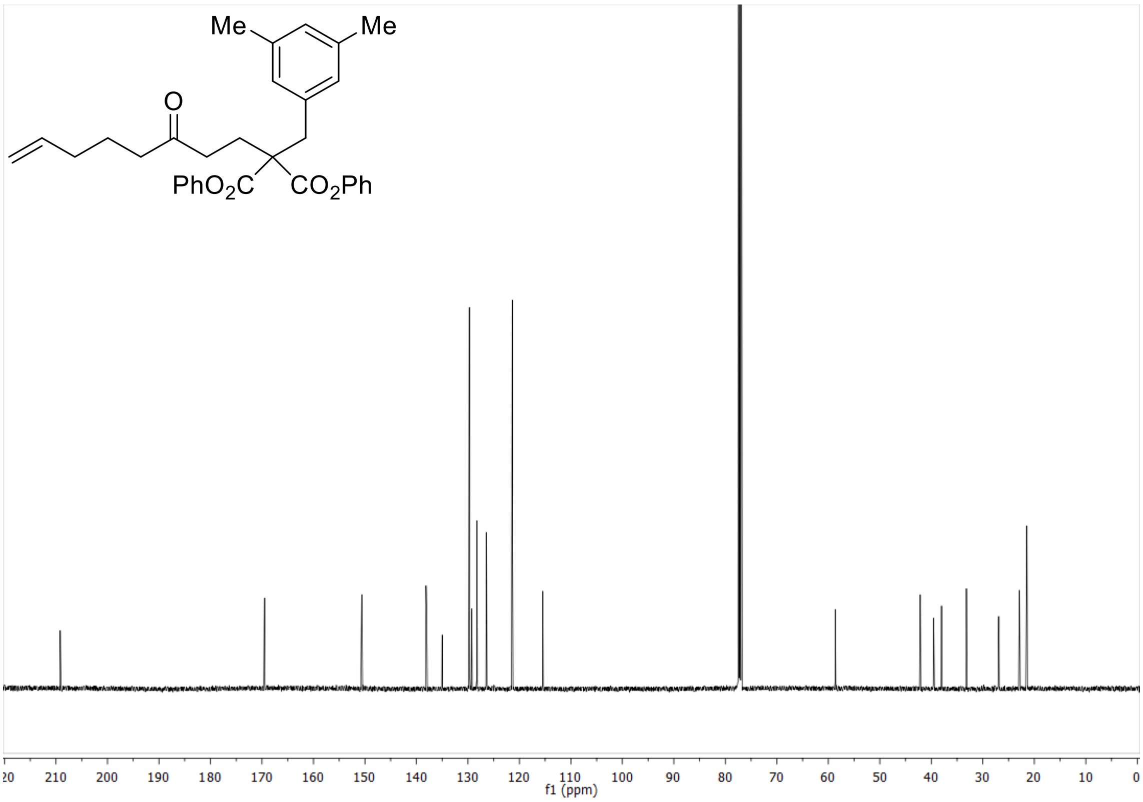

Supplementary Figure 87. ^{1}H NMR (500 MHz, $CDCl_3$) spectrum of **S49**

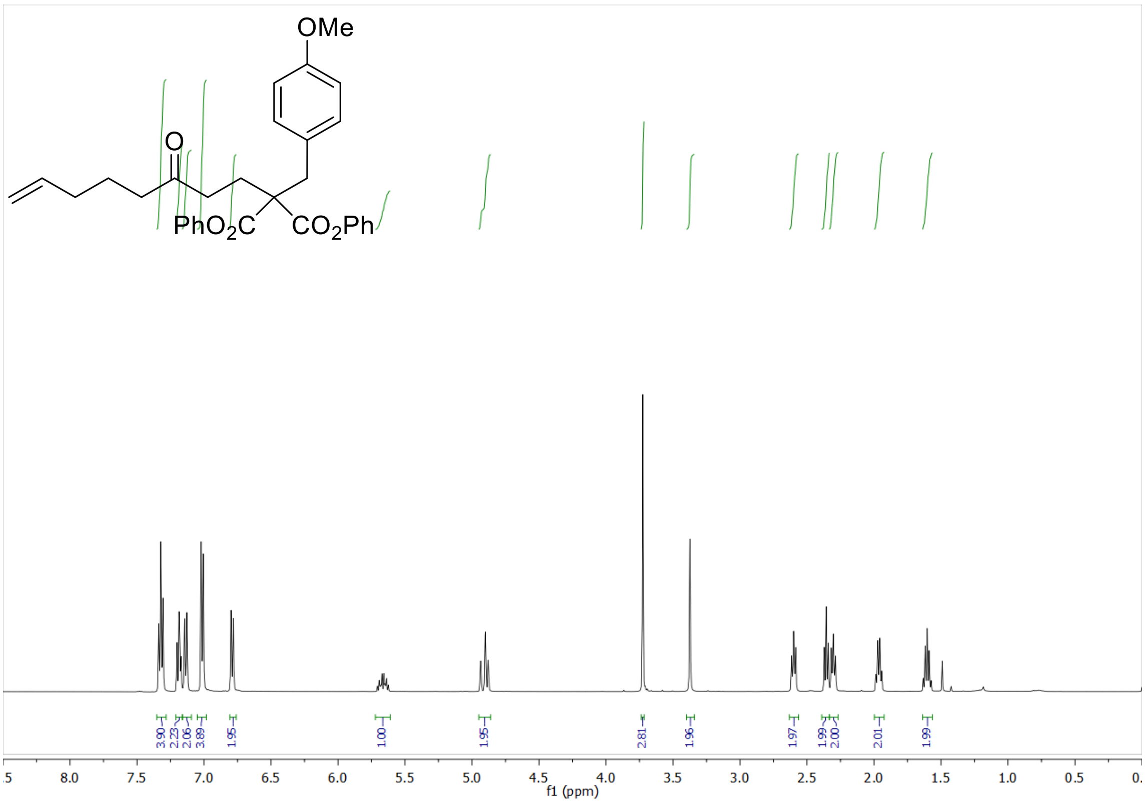

Supplementary Figure 88. ^{13}C NMR (125 MHz, $CDCl_3$) spectrum of **S49**

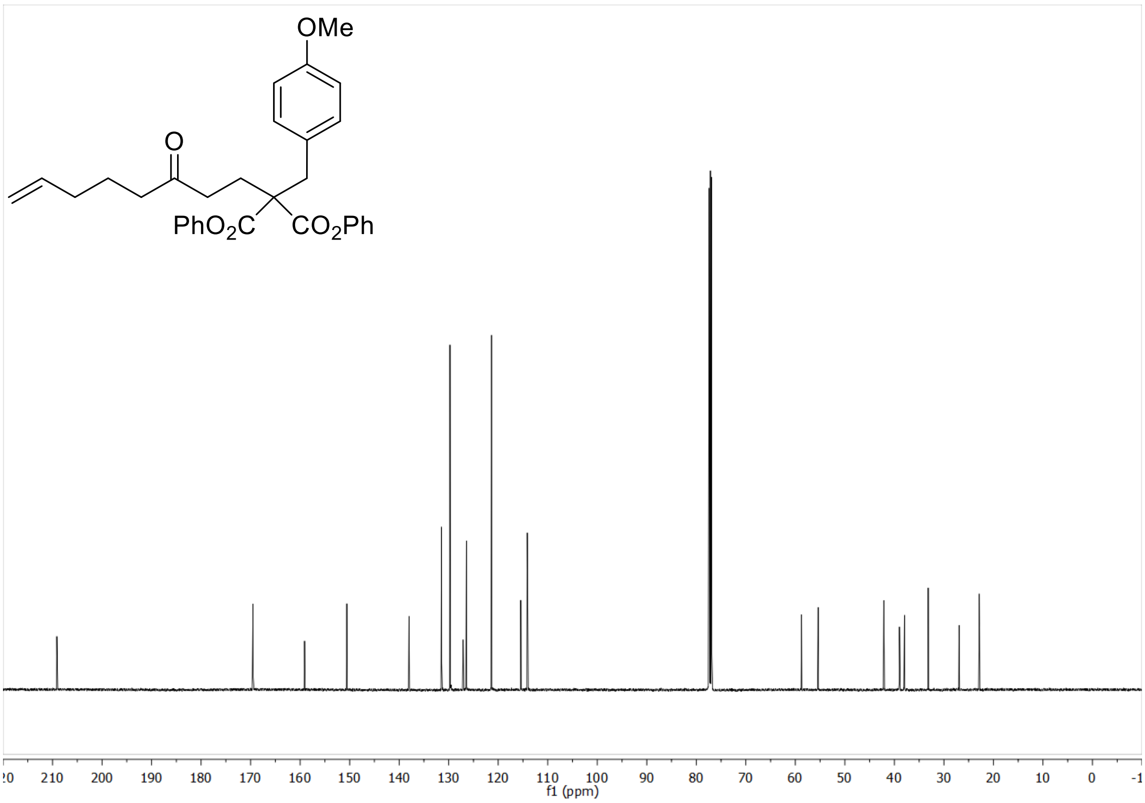

Supplementary Figure 89. ^{1}H NMR (500 MHz, $CDCl_3$) of **S50**

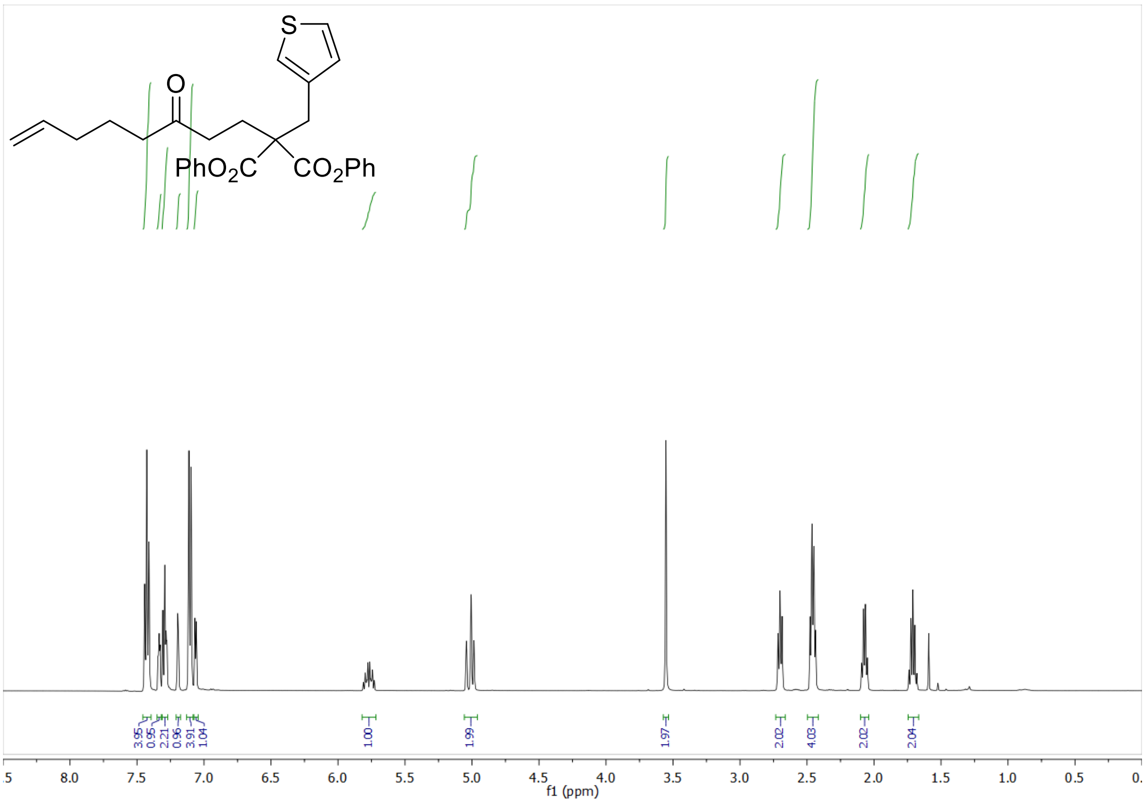

Supplementary Figure 90. ^{13}C NMR (125 MHz, $CDCl_3$) spectrum of **S50**

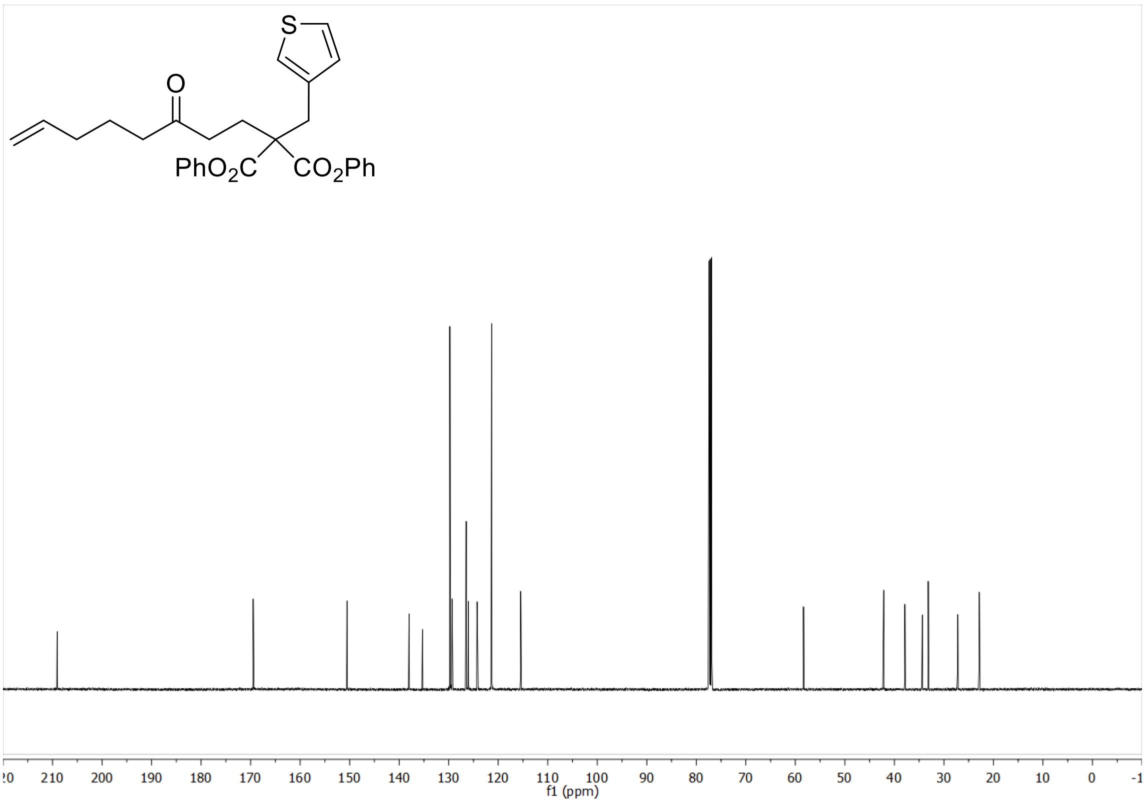

Supplementary Figure 91. ^{1}H NMR (400 MHz, $CDCl_3$) spectrum of **S51**

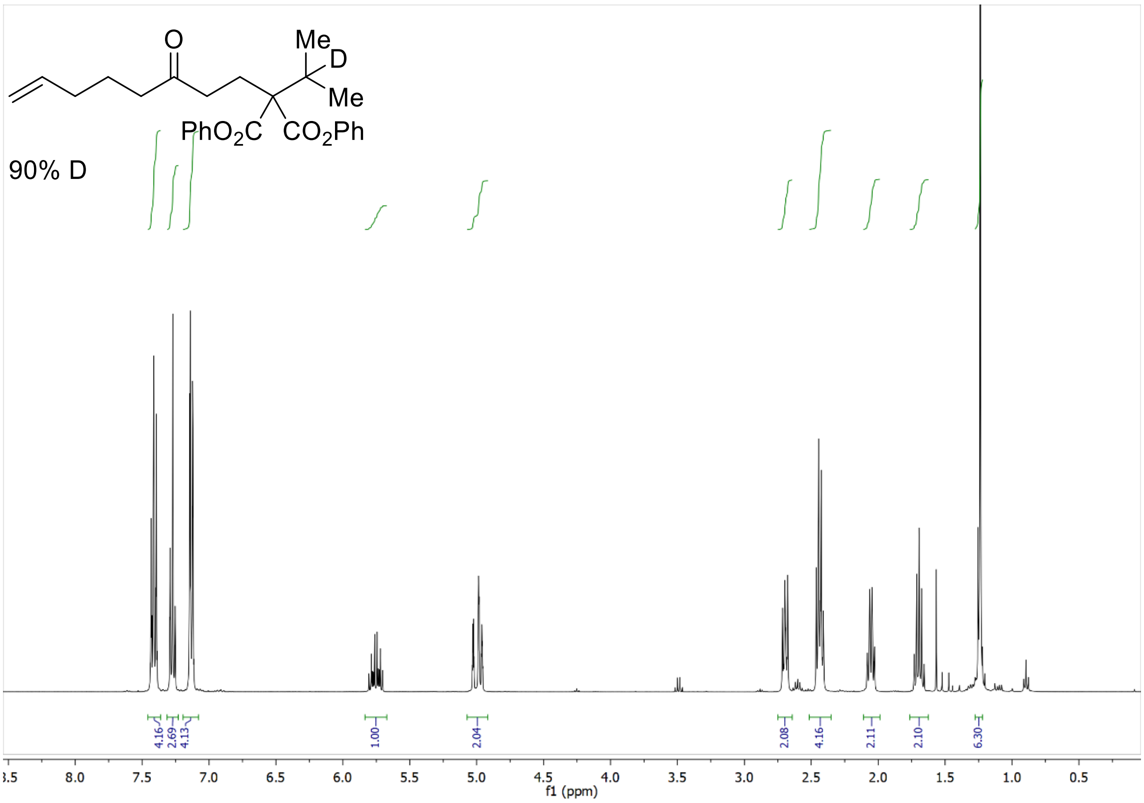

Supplementary Figure 92. ^{13}C NMR (100 MHz, $CDCl_3$) spectrum of **S51**

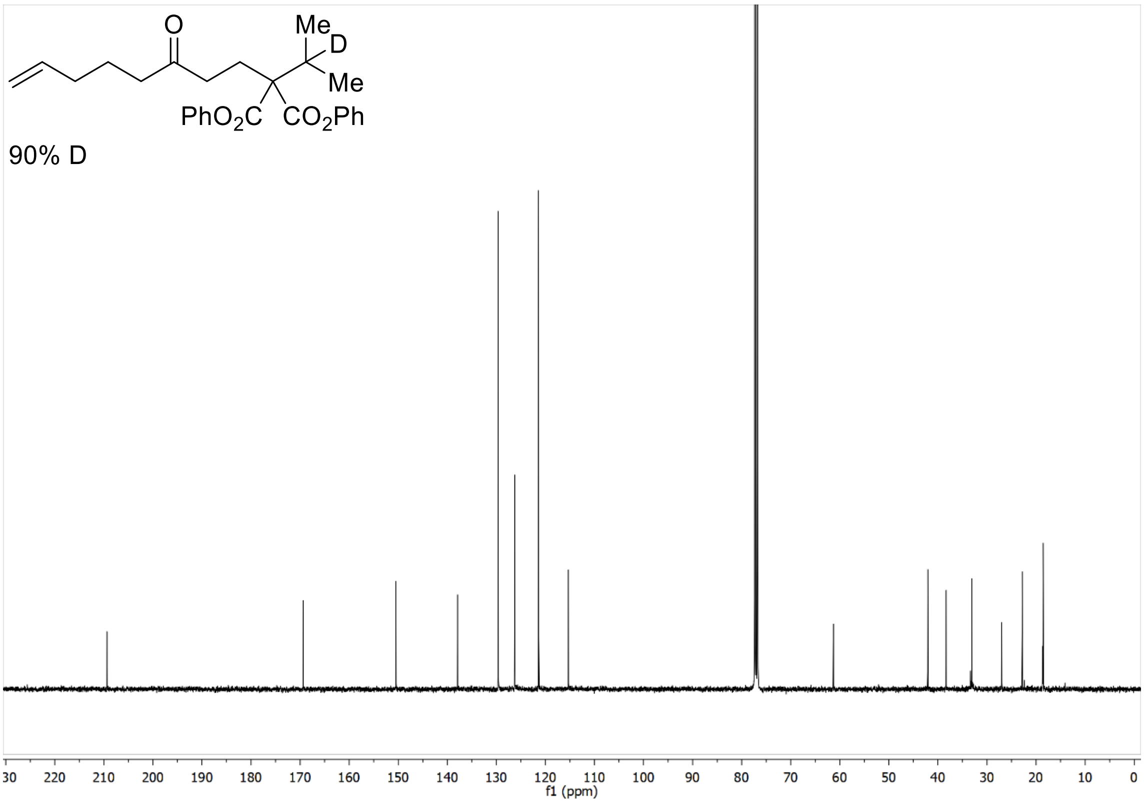

Supplementary Figure 93. ^{1}H NMR (400 MHz, $CDCl_3$) spectrum of **6a'**

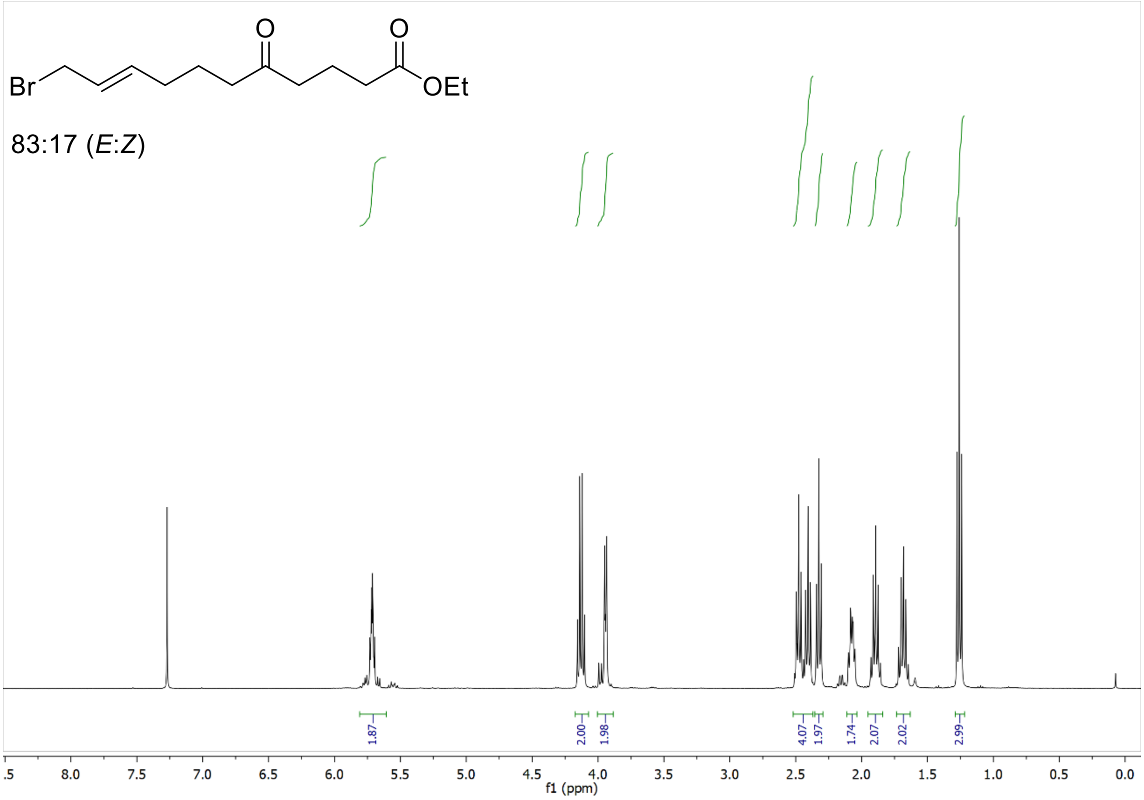

Supplementary Figure 94. ^{13}C NMR (100 MHz, $CDCl_3$) spectrum of **6a'**

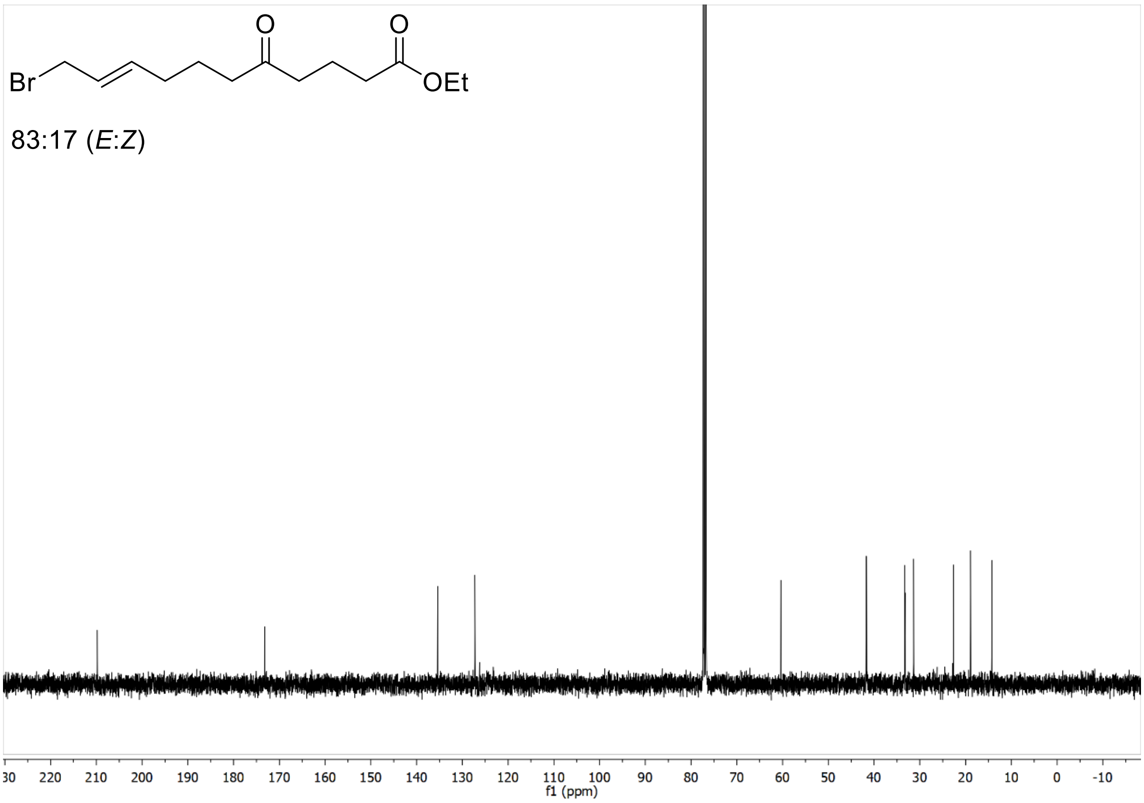

Supplementary Figure 95. ^{1}H NMR (400 MHz, $CDCl_3$) spectrum of **6b'**

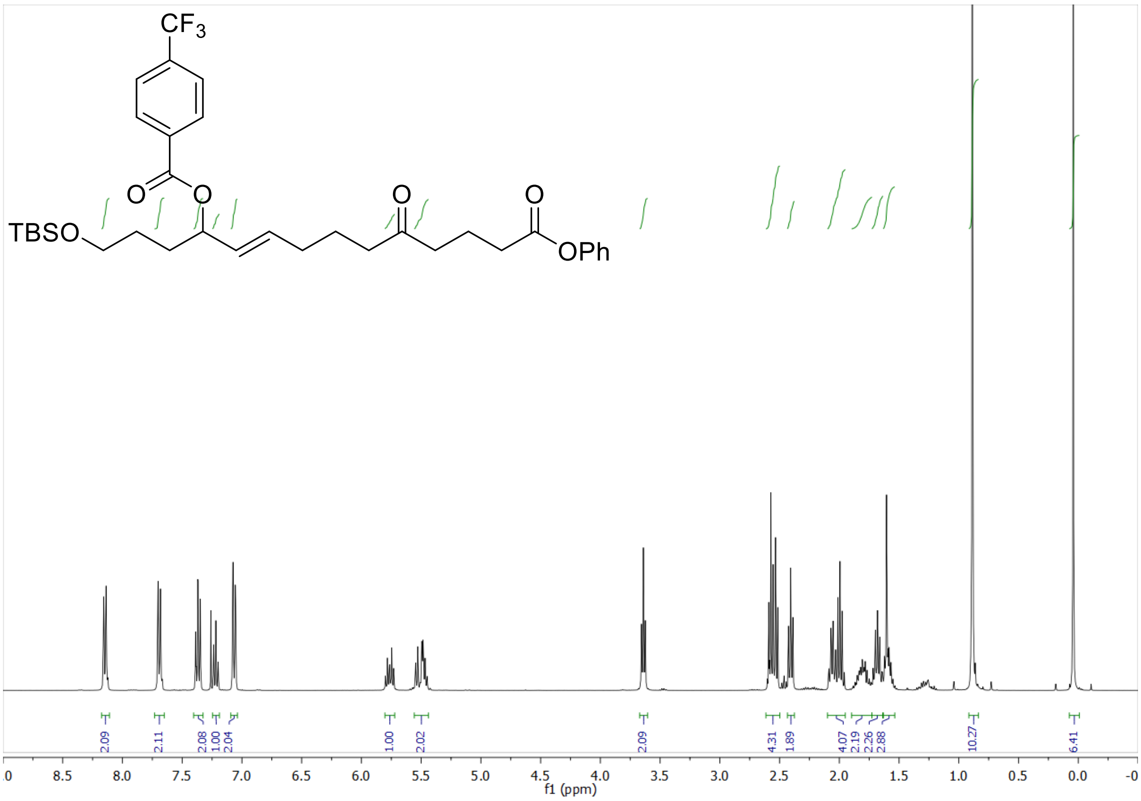

Supplementary Figure 96. ^{13}C NMR (100 MHz, $CDCl_3$) spectrum of **6b'**

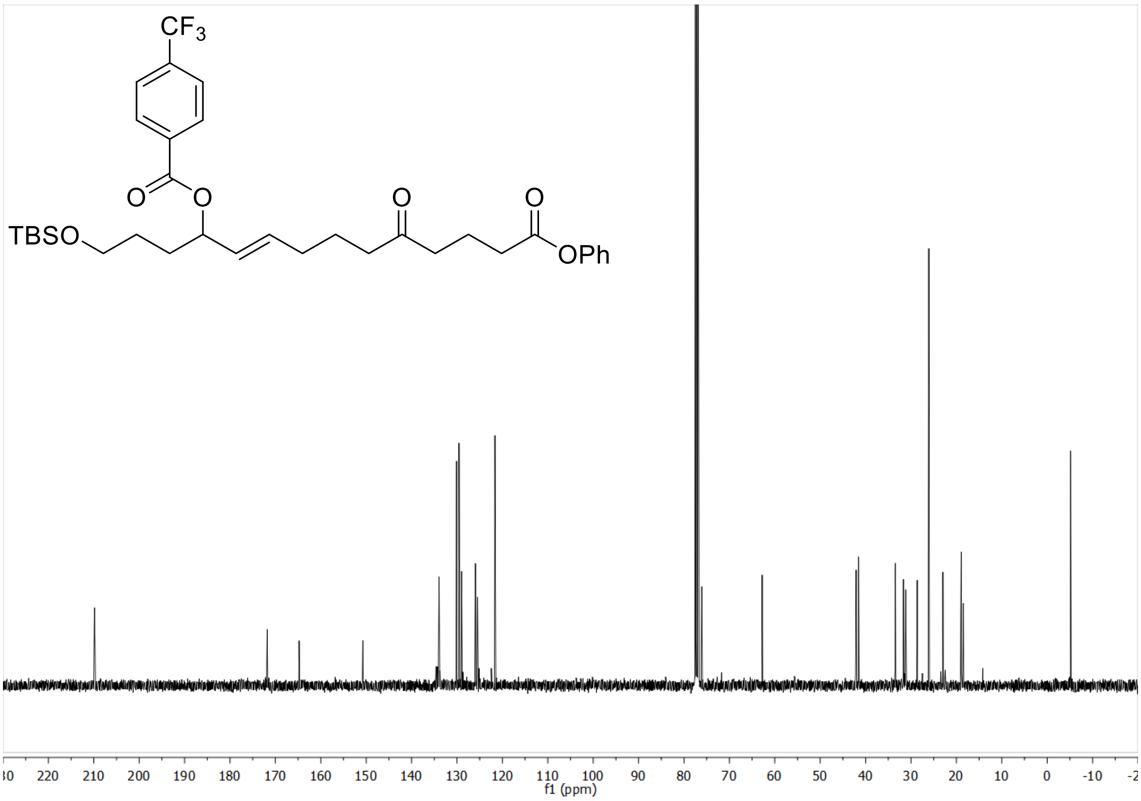

Supplementary Figure 97. ^{1}H NMR (400 MHz, $CDCl_3$) spectrum of **6c'**

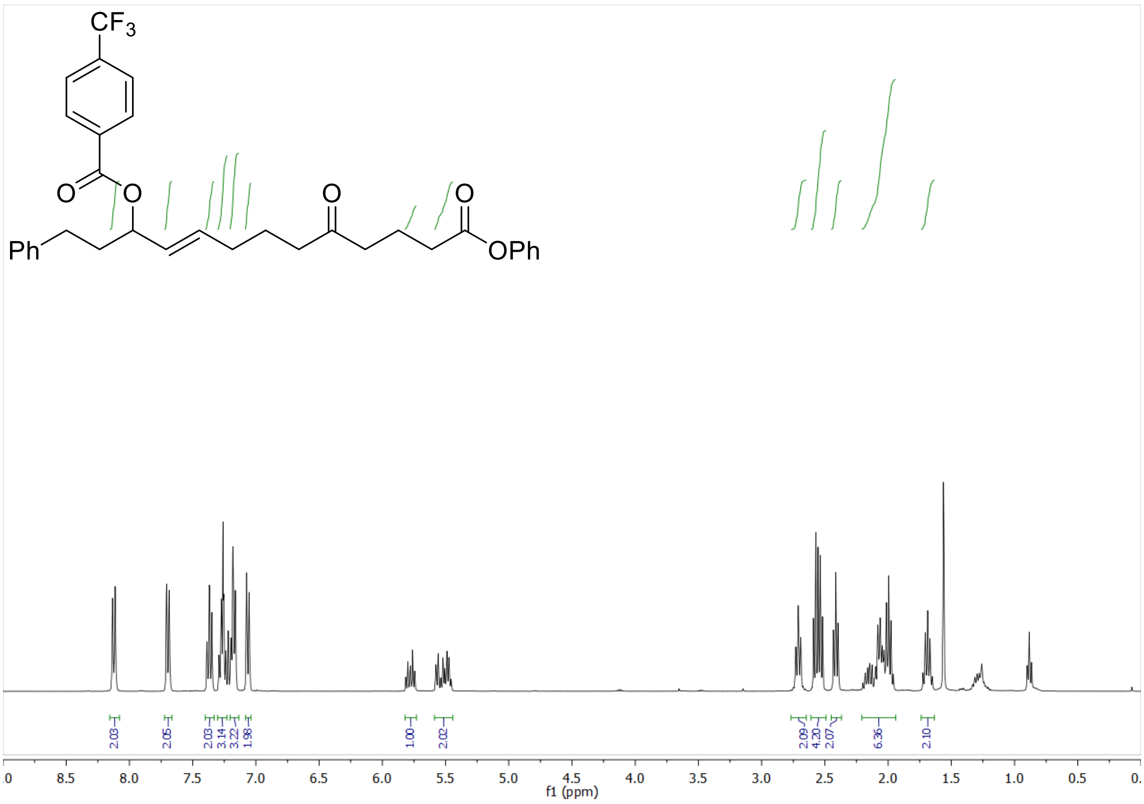

Supplementary Figure 98. ^{13}C NMR (100 MHz, $CDCl_3$) spectrum of **6c'**

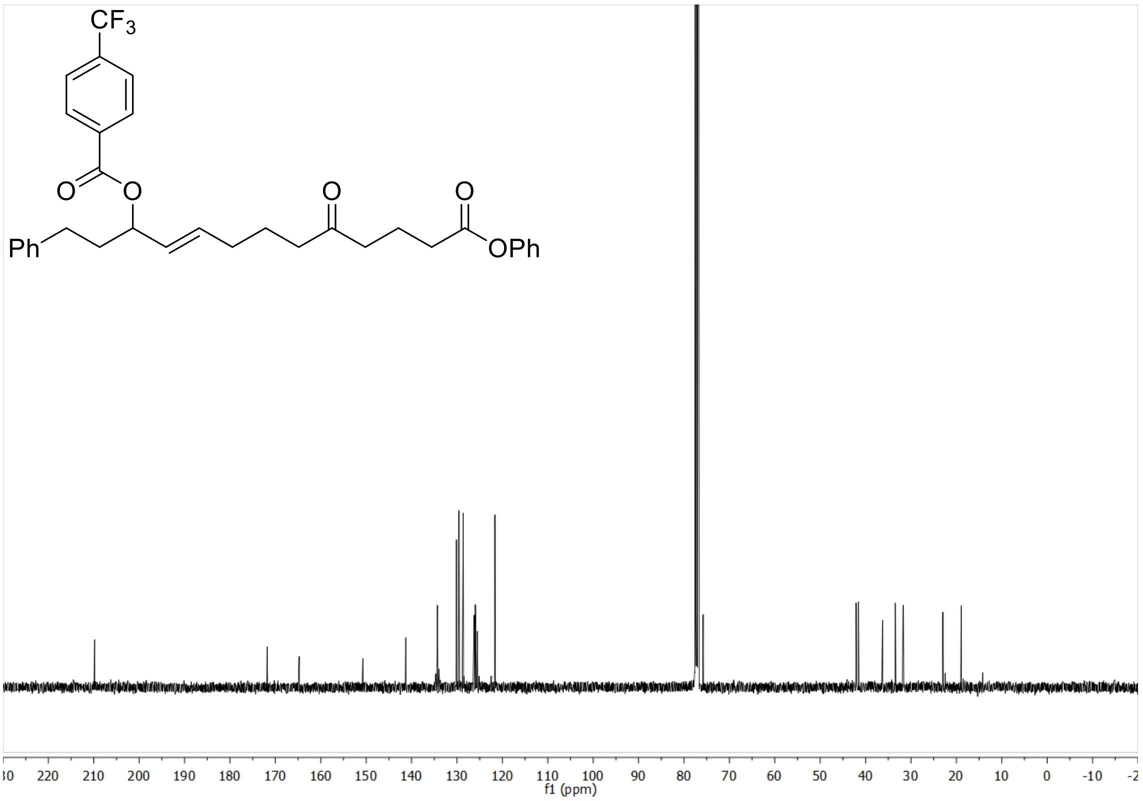

Supplementary Figure 99. ^{1}H NMR (400 MHz, $CDCl_3$) spectrum of **6d'**

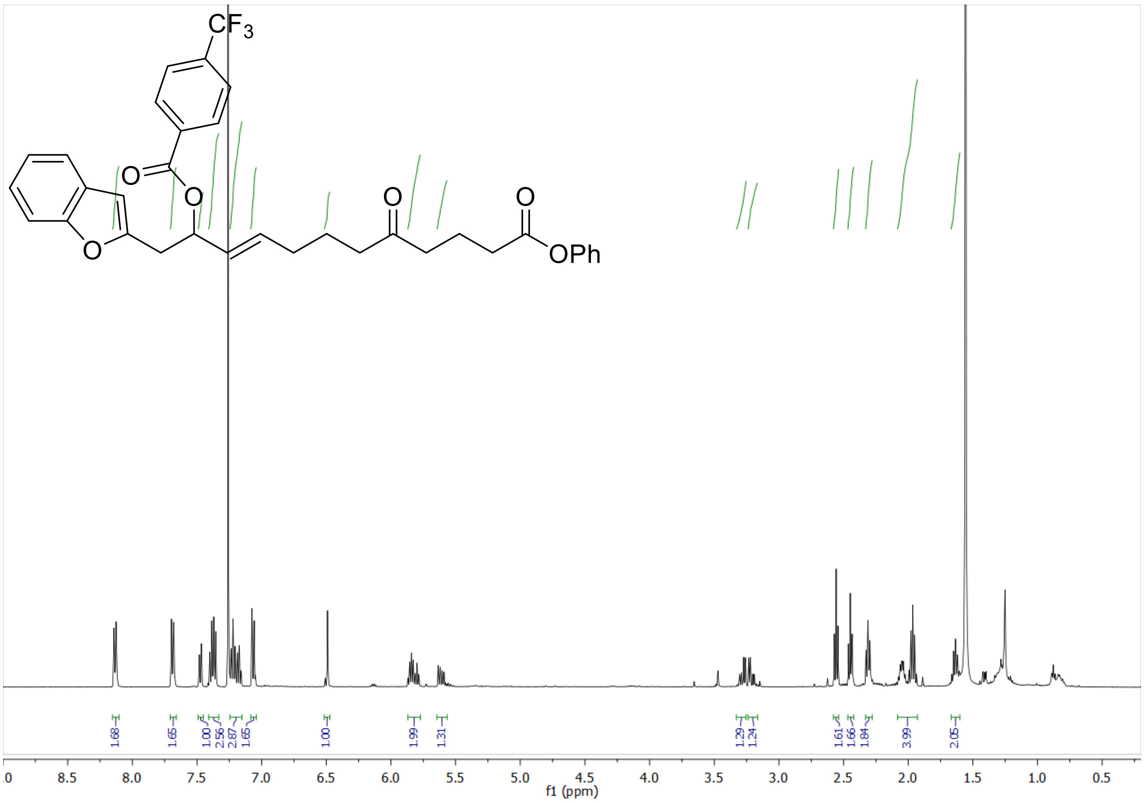

Supplementary Figure 100. ^{13}C NMR (100 MHz, $CDCl_3$) spectrum of **6d'**

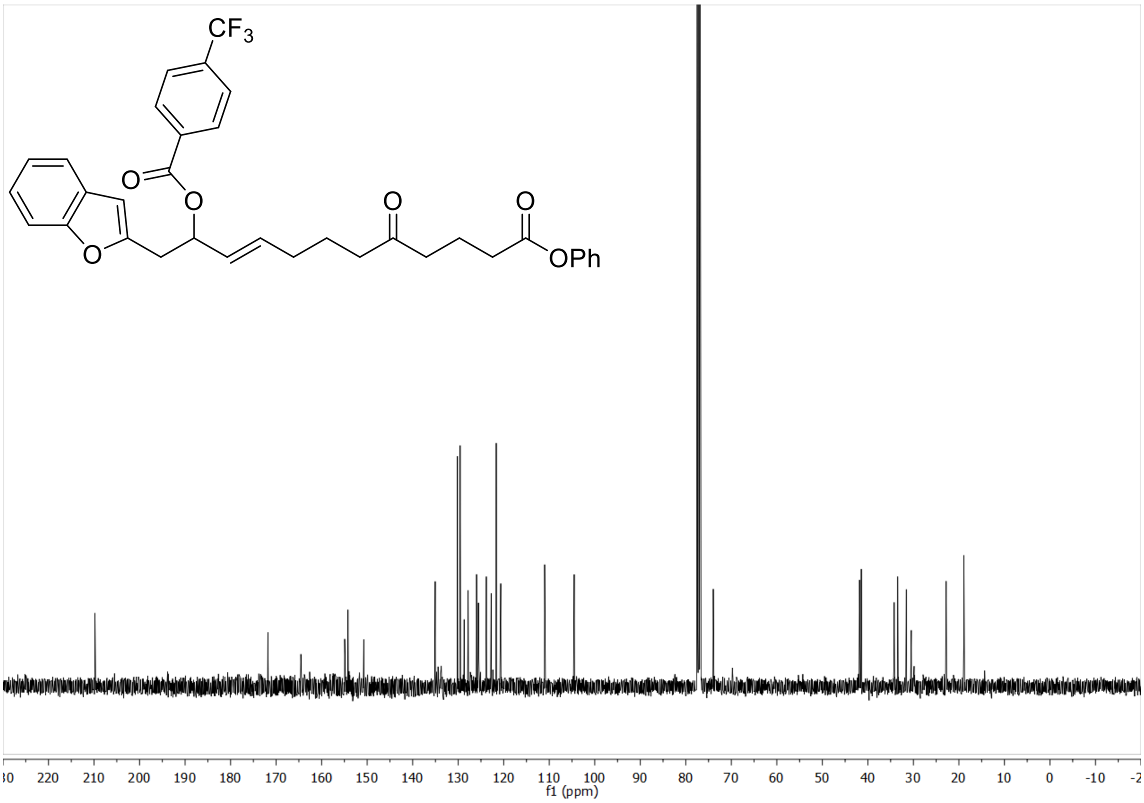

Supplementary Figure 101. ^{1}H NMR (400 MHz, $CDCl_3$) spectrum of **6a**

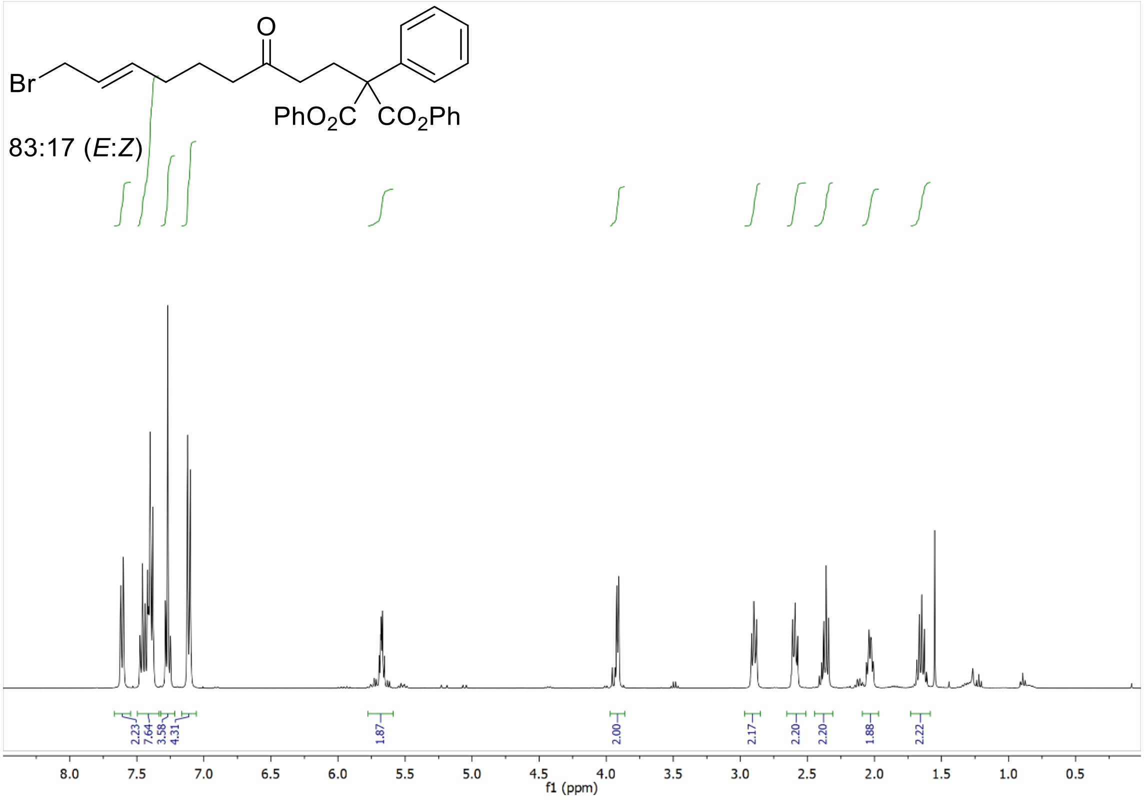

Supplementary Figure 102. ^{13}C NMR (100 MHz, $CDCl_3$) spectrum of **6a**

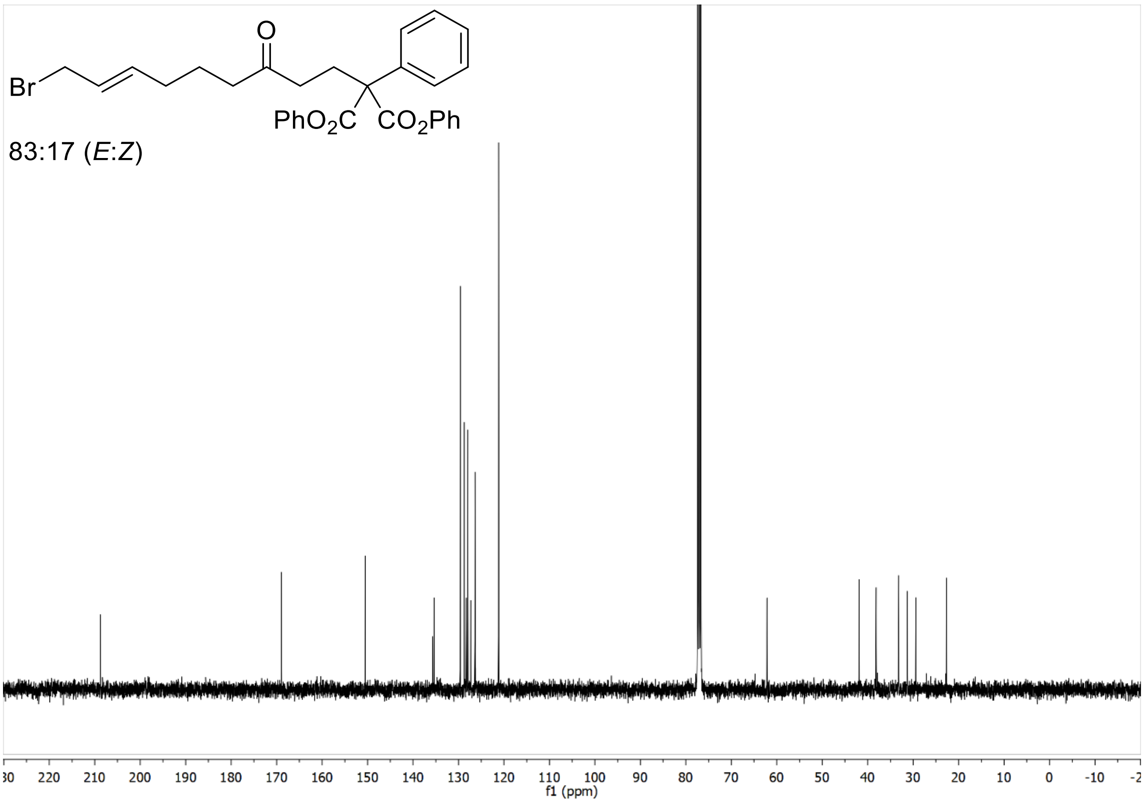

Supplementary Figure 103. ^1H NMR (400 MHz, $CDCl_3$) spectrum of **6b**

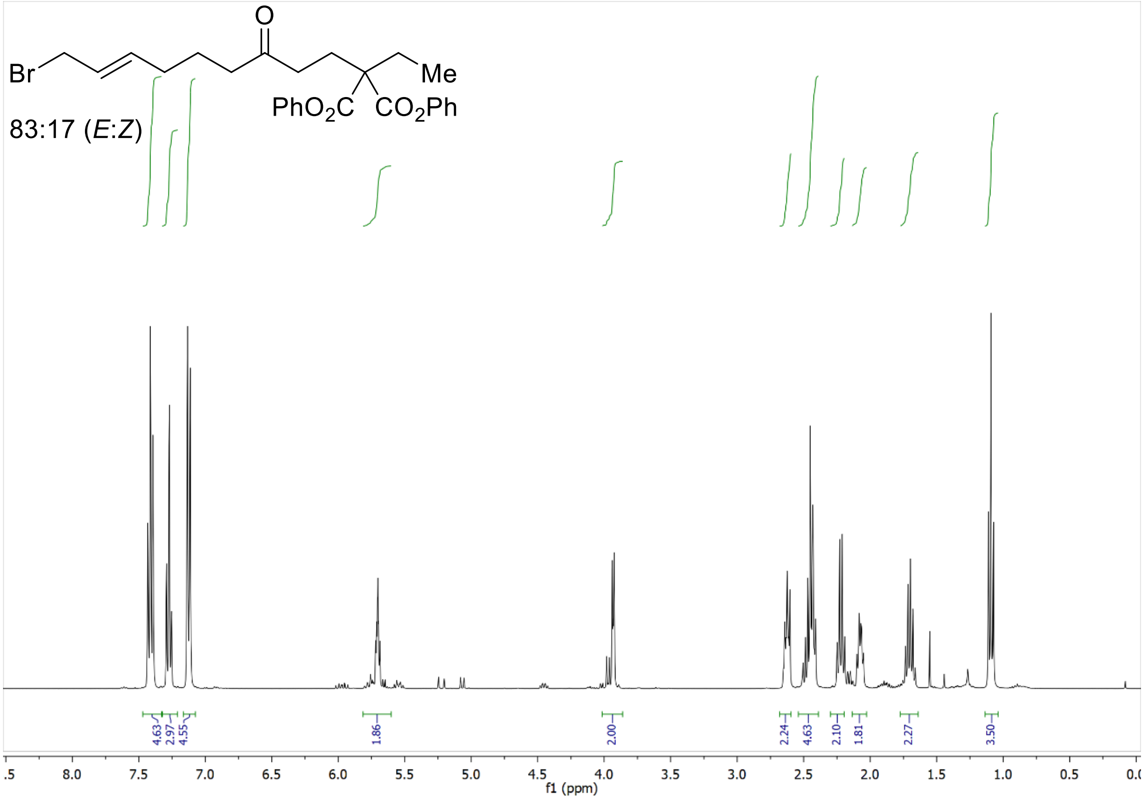

Supplementary Figure 104. ^{13}C NMR (100 MHz, $CDCl_3$) spectrum of **6b**

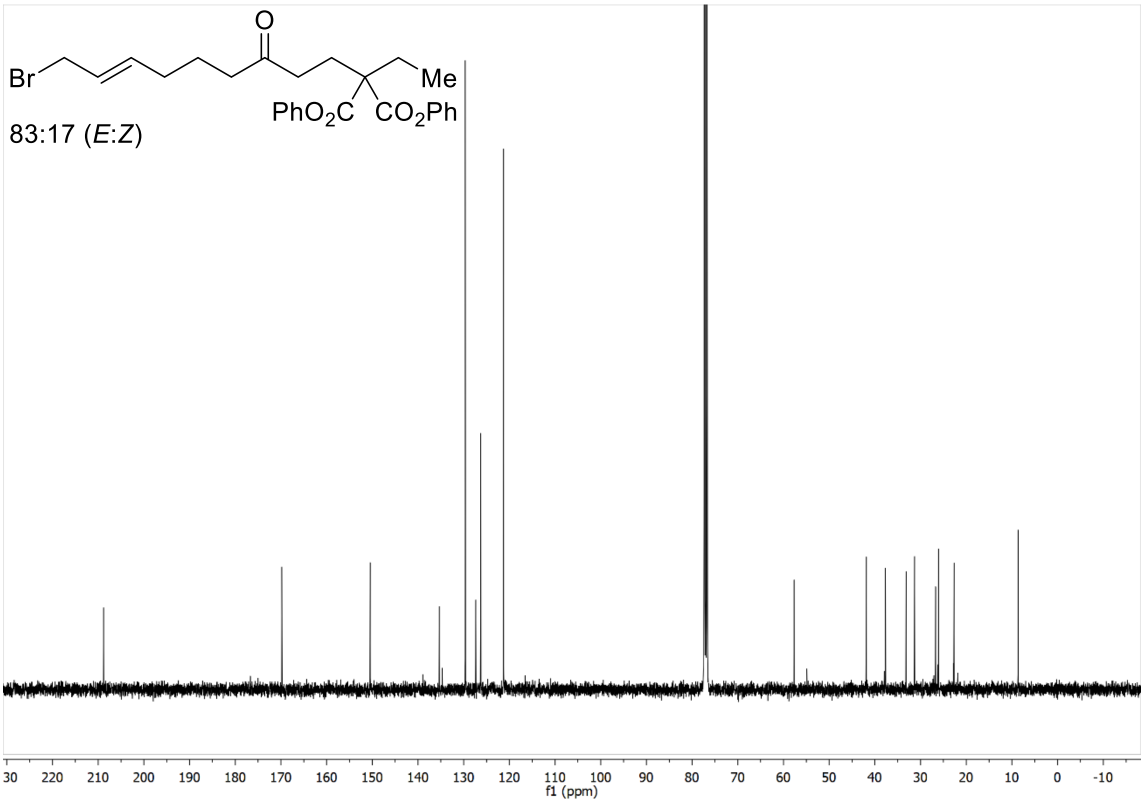

Supplementary Figure 105. ^{1}H NMR (400 MHz, $CDCl_3$) spectrum of **6c**

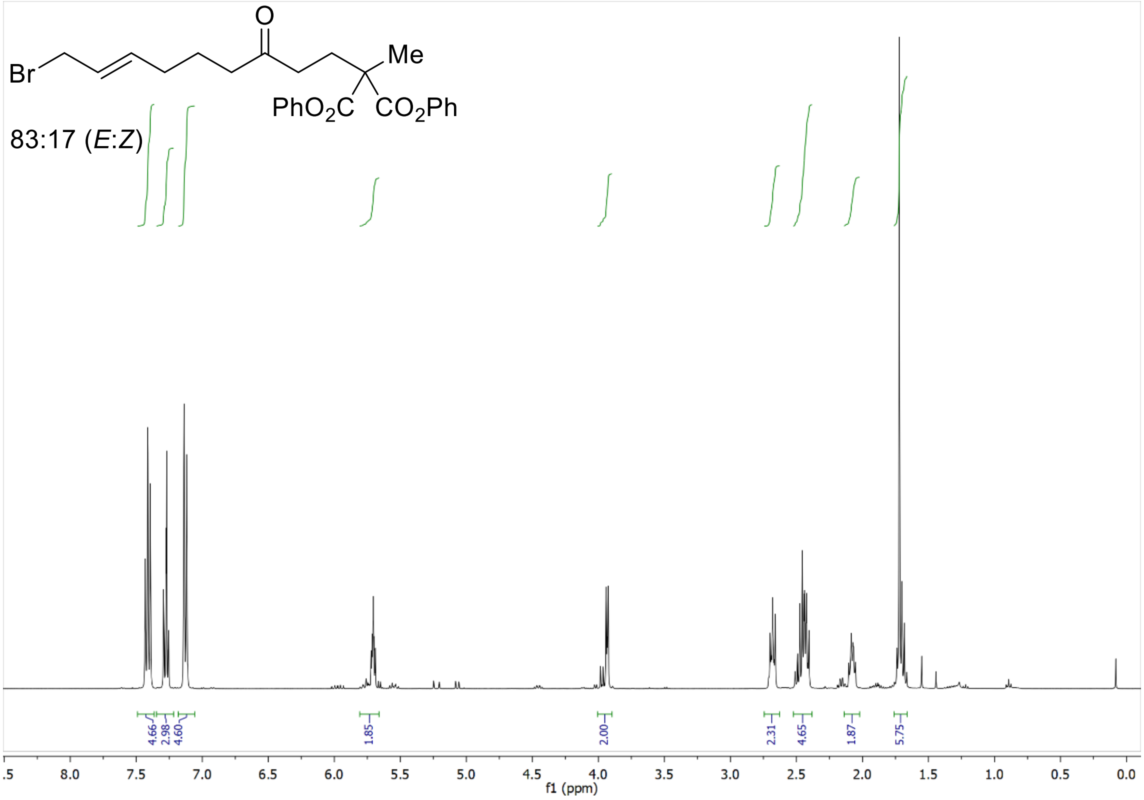

Supplementary Figure 106. ^{13}C NMR (100 MHz, $CDCl_3$) spectrum of **6c**

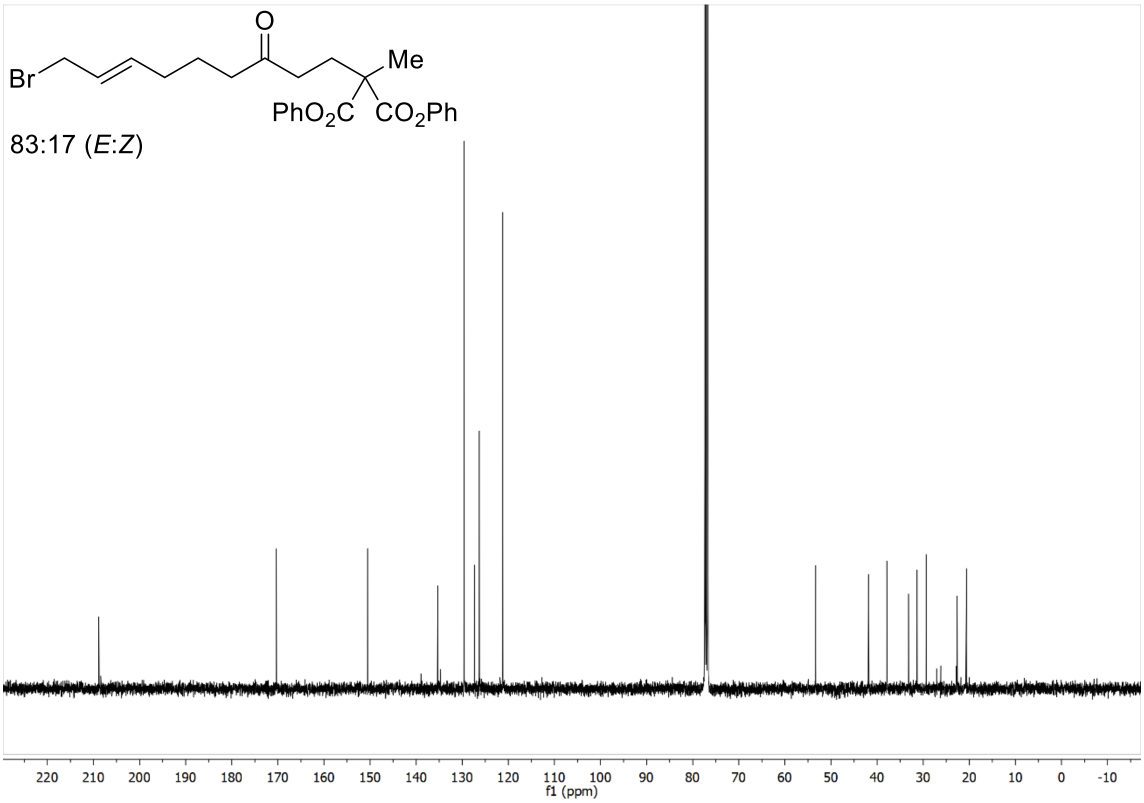

Supplementary Figure 107. ^{1}H NMR (400 MHz, $CDCl_3$) spectrum of **6d**

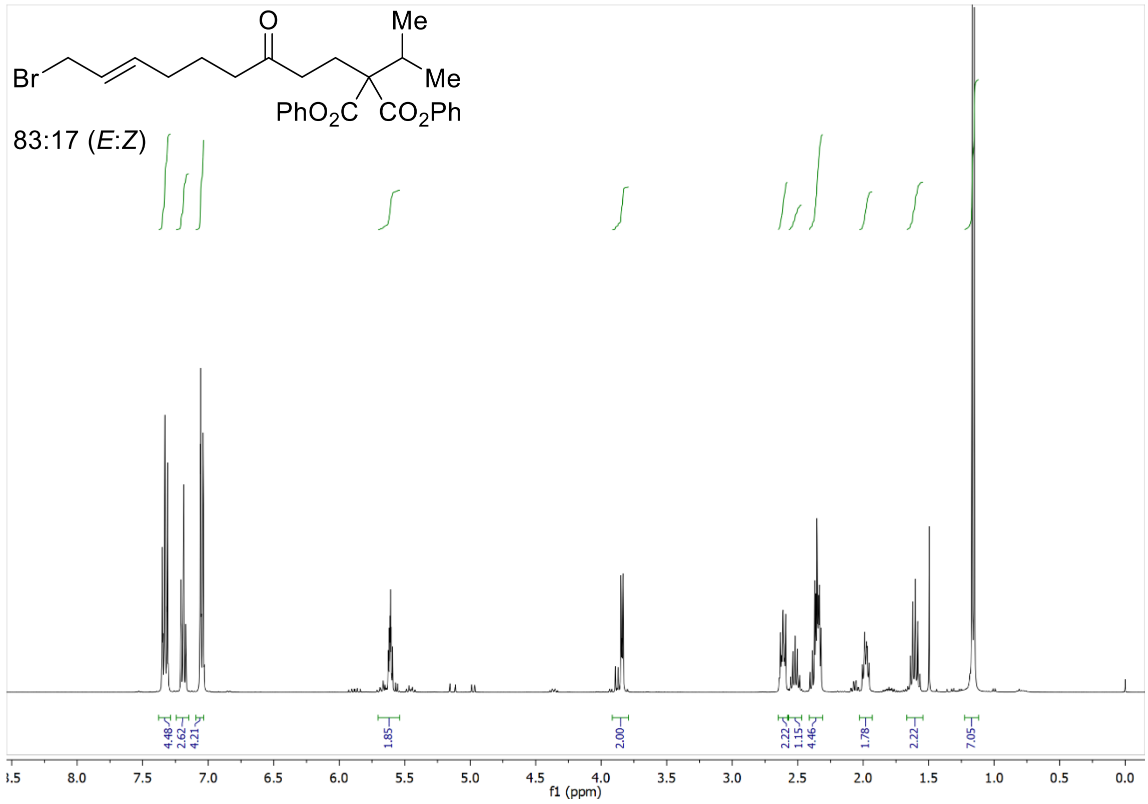

Supplementary Figure 108. ^{13}C NMR (100 MHz, $CDCl_3$) spectrum of **6d**

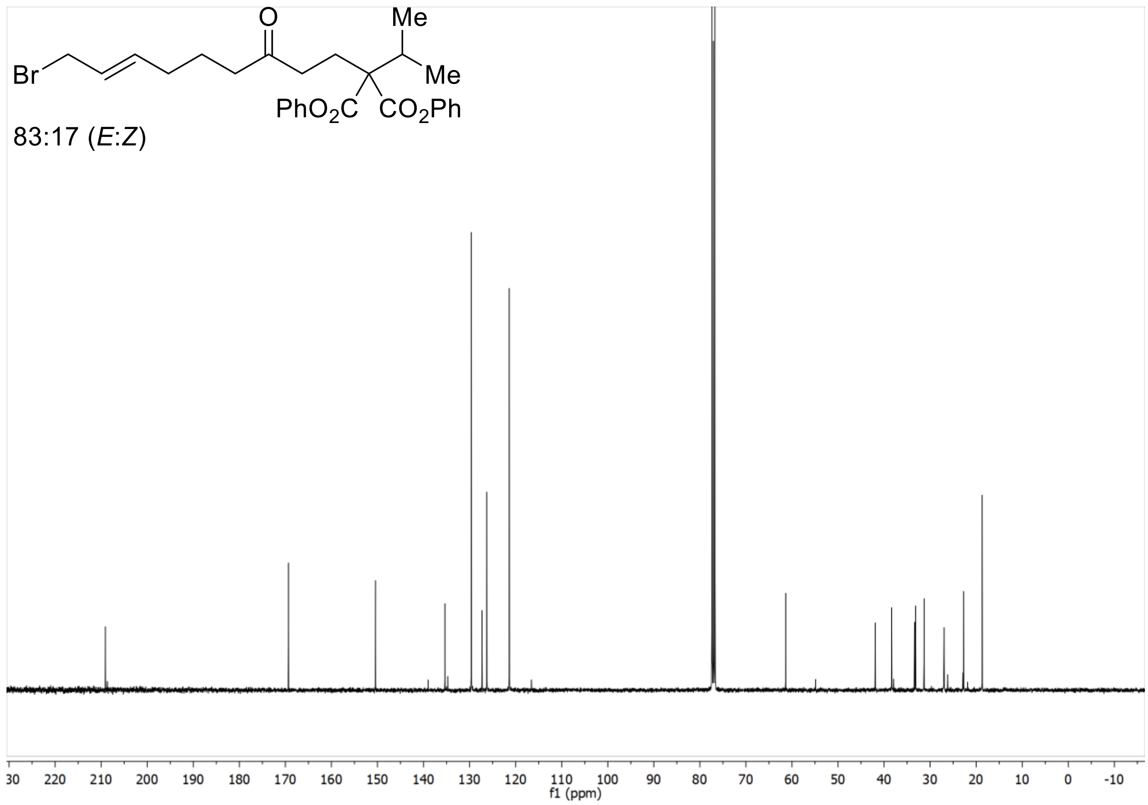

Supplementary Figure 109. ^{1}H NMR (400 MHz, $CDCl_3$) spectrum of **6e**

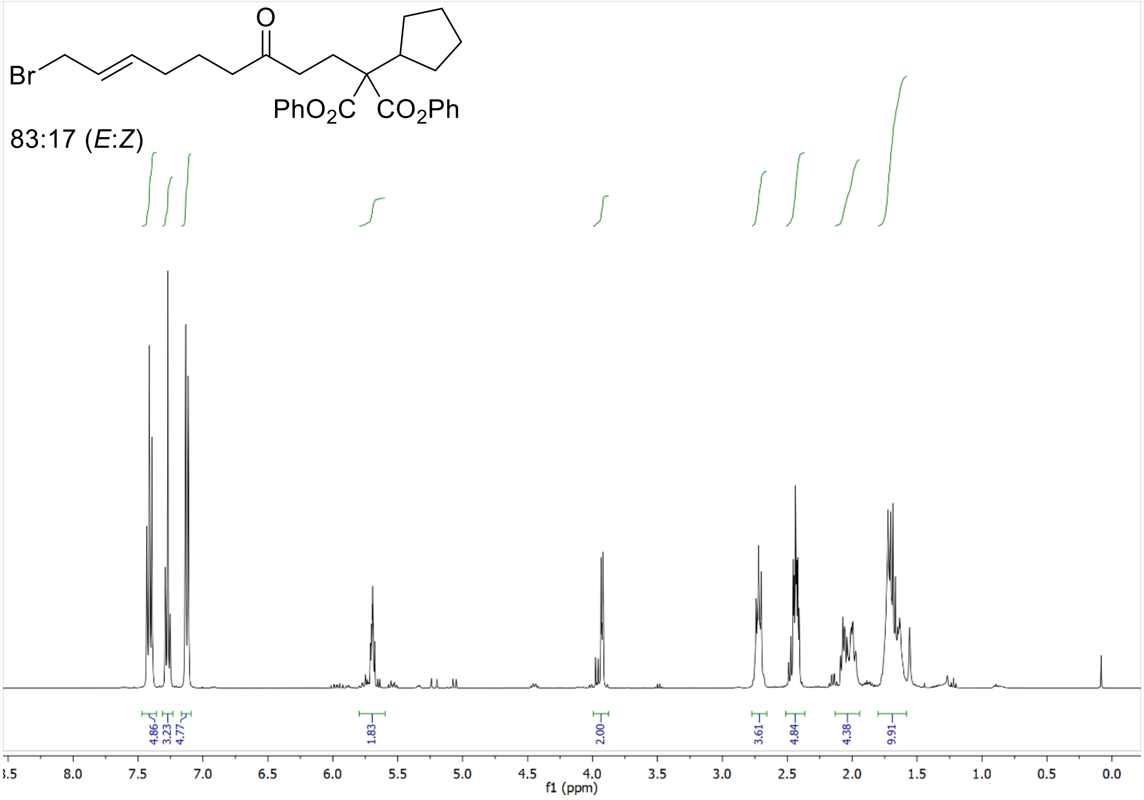

Supplementary Figure 110. ^{13}C NMR (100 MHz, $CDCl_3$) spectrum of **6e**

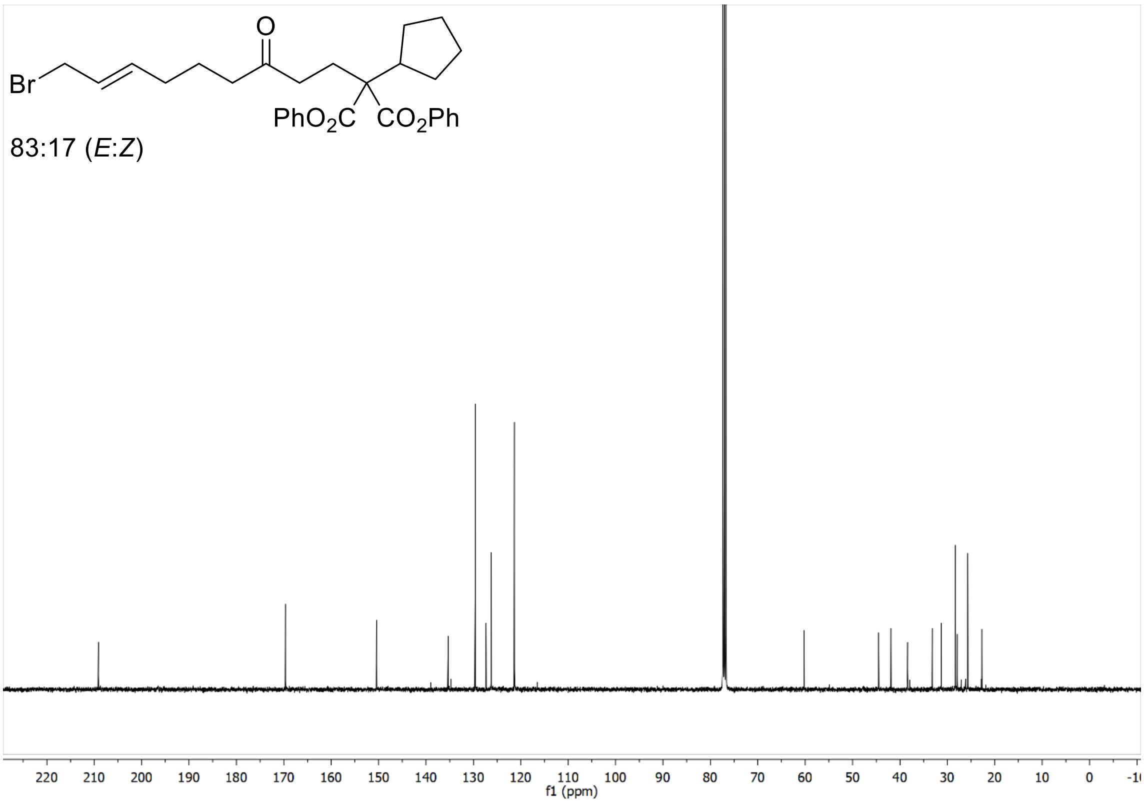

Supplementary Figure 111. ^{1}H NMR (400 MHz, $CDCl_3$) spectrum of **6f**

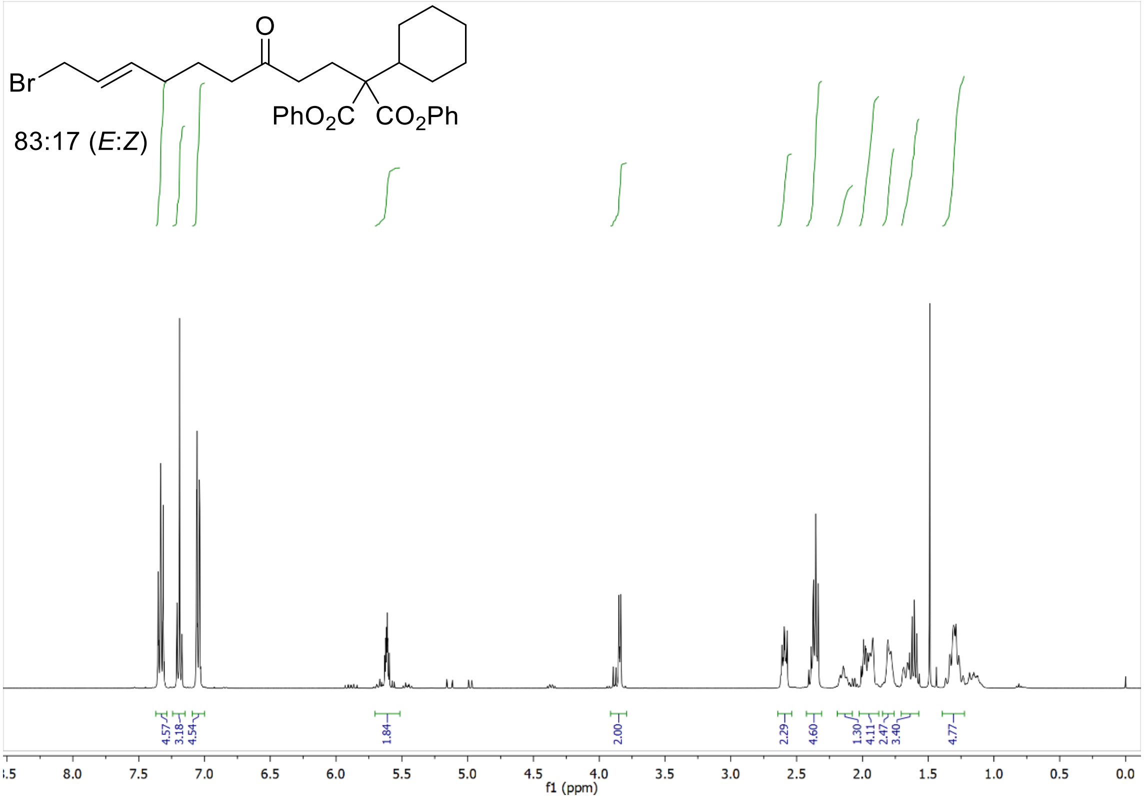

Supplementary Figure 112. ^{13}C NMR (100 MHz, $CDCl_3$) spectrum of **6f**

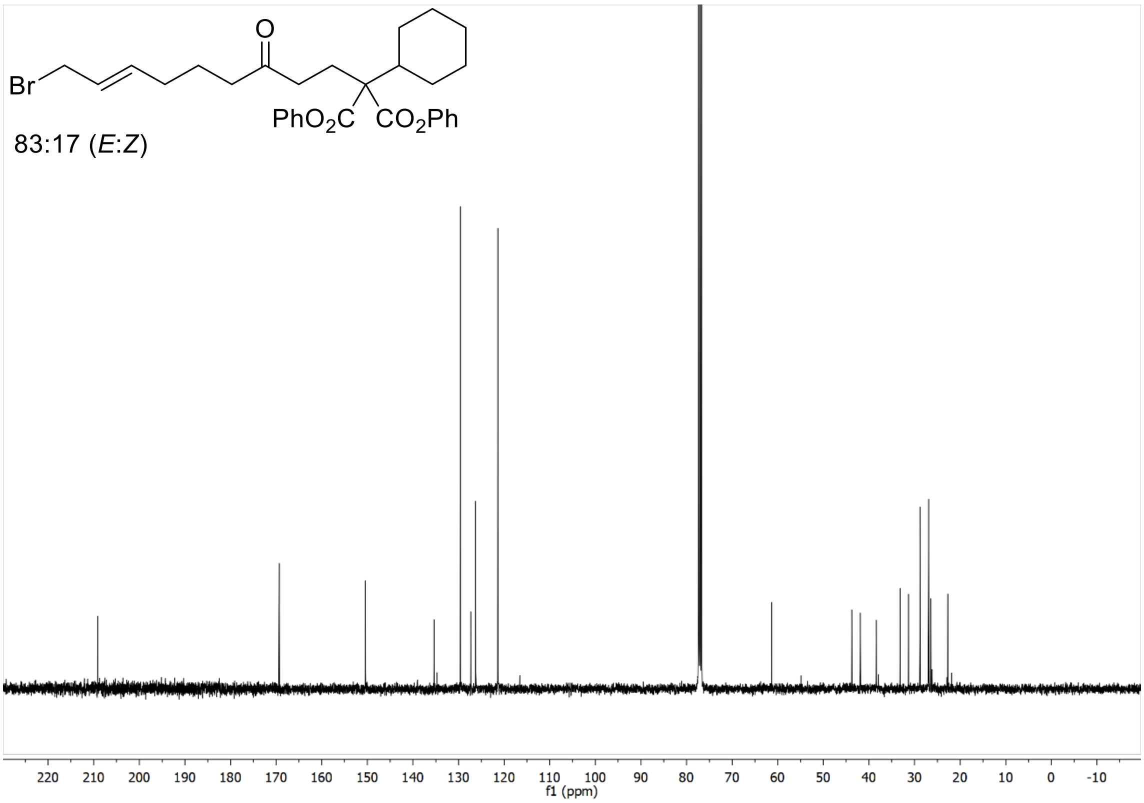

Supplementary Figure 113. ^{1}H NMR (400 MHz, $CDCl_3$) spectrum of **6g**

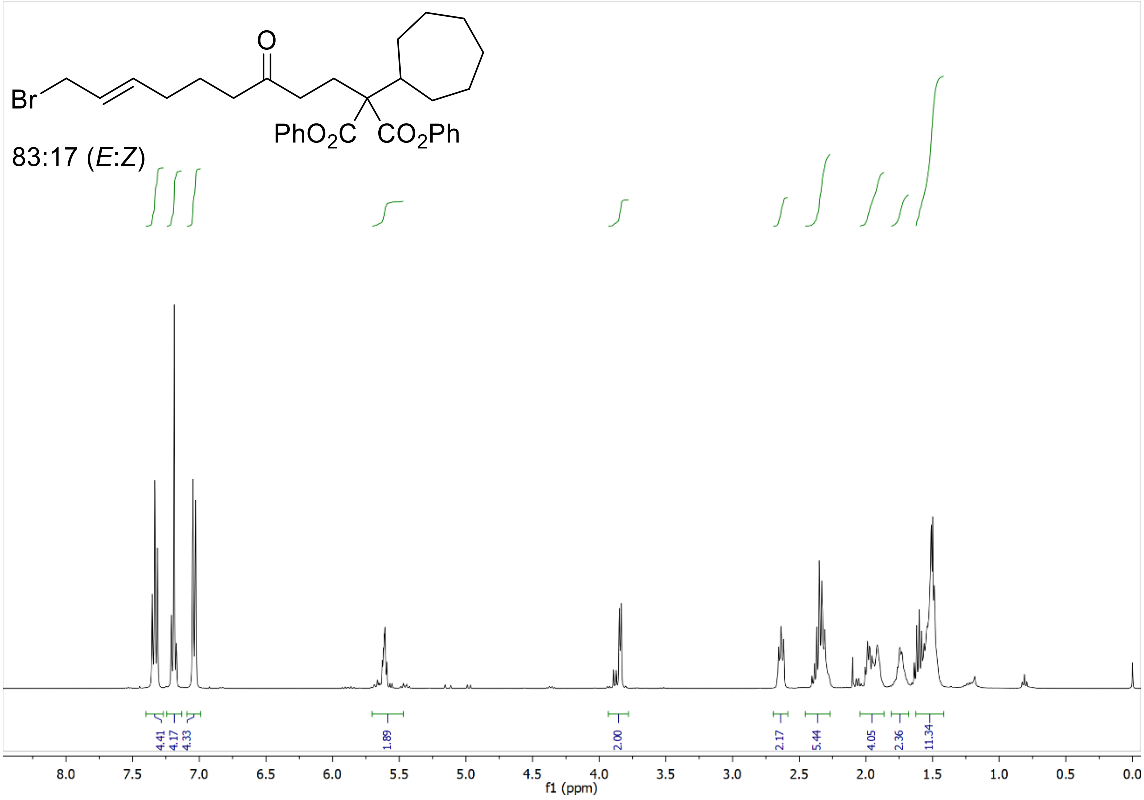

Supplementary Figure 114. ^{13}C NMR (100 MHz, $CDCl_3$) spectrum of **6g**

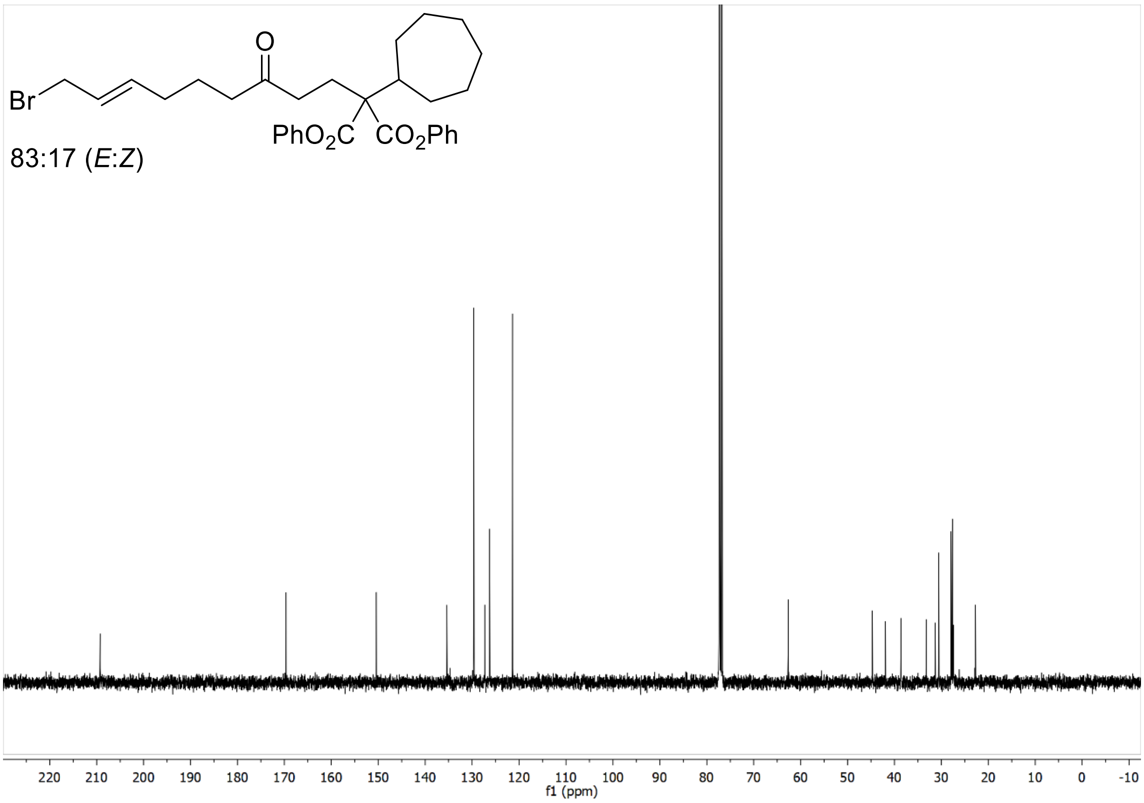

Supplementary Figure 115. ^{1}H NMR (400 MHz, $CDCl_3$) spectrum of **6h**

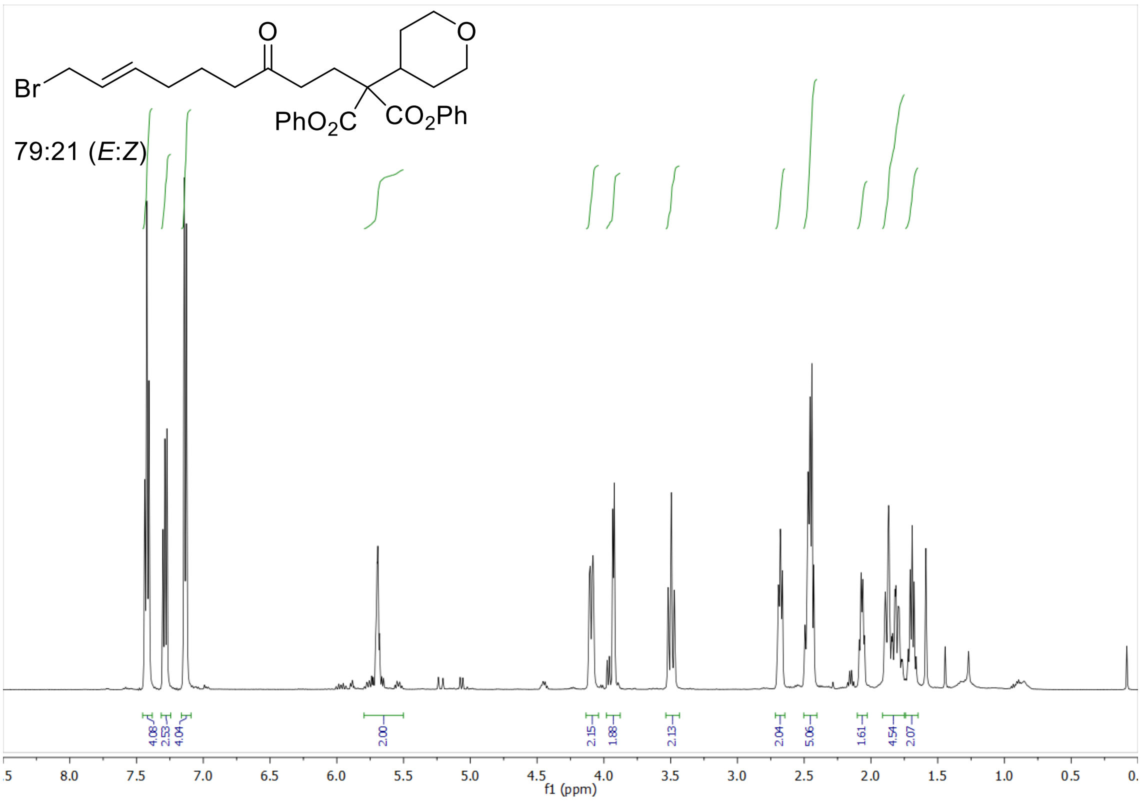

Supplementary Figure 116. ^{13}C NMR (100 MHz, $CDCl_3$) spectrum of **6h**

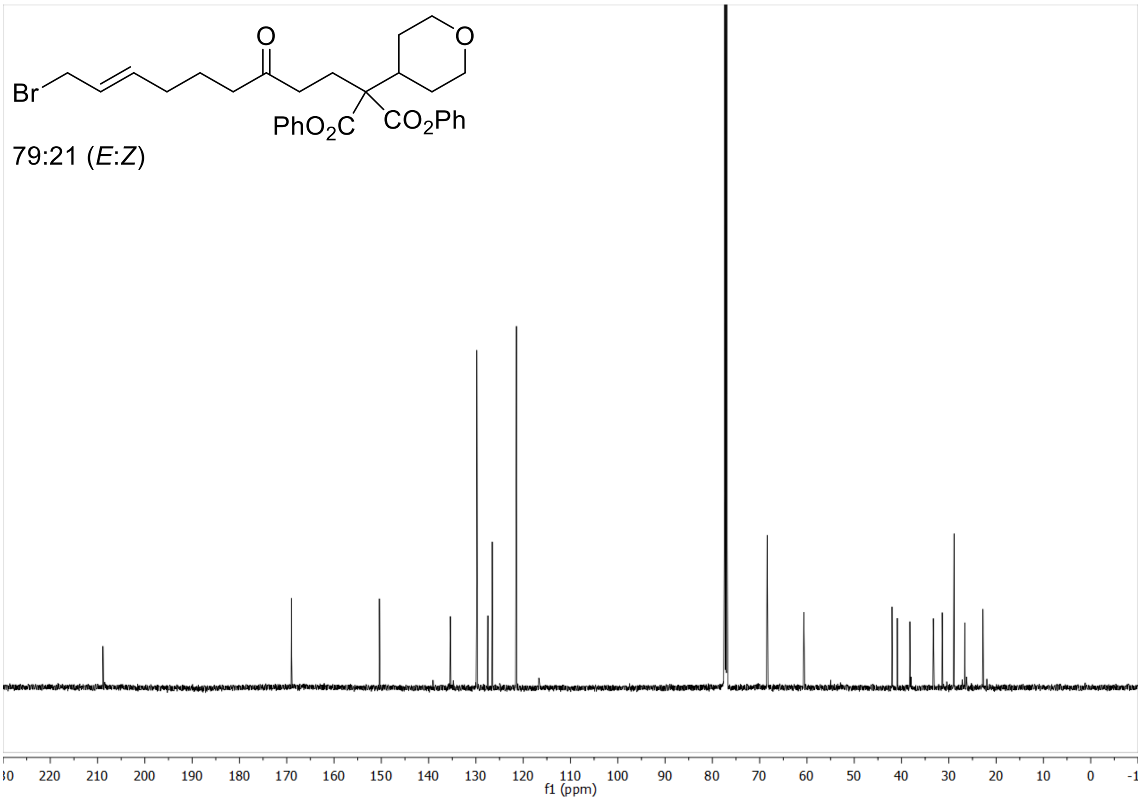

Supplementary Figure 117. ^{1}H NMR (400 MHz, $CDCl_3$) spectrum of **6i**

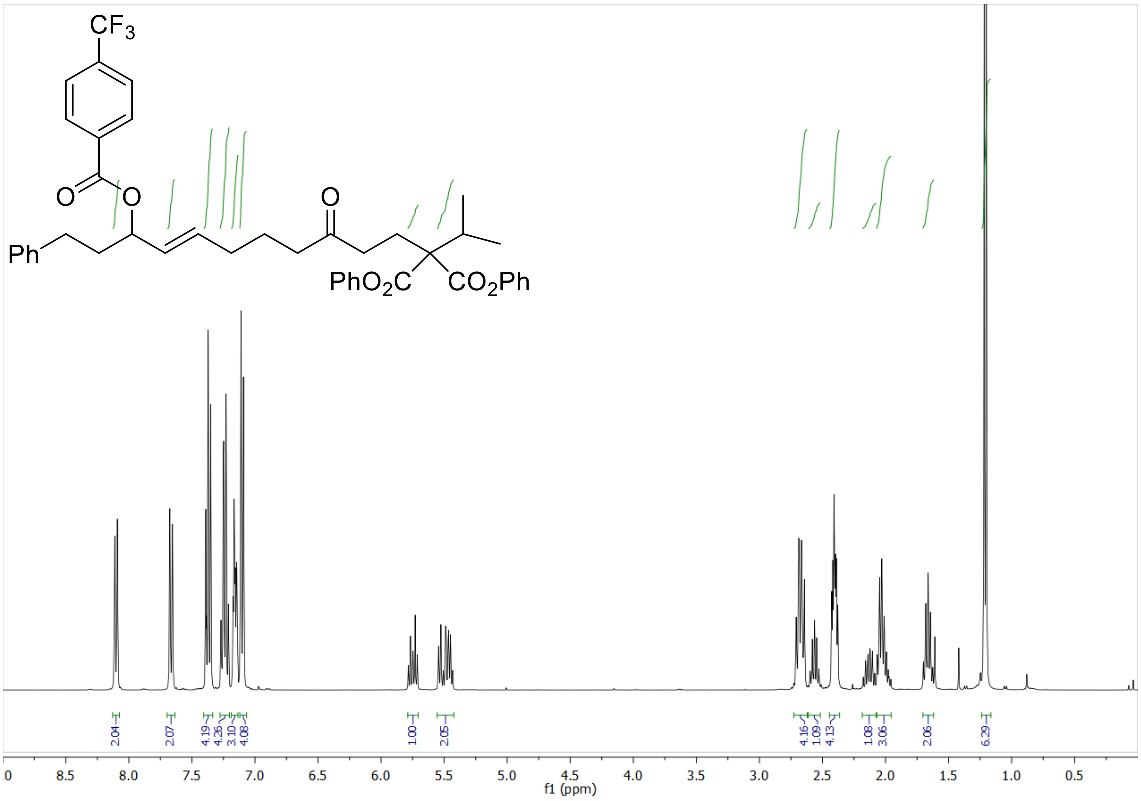

Supplementary Figure 118. ^{13}C NMR (100 MHz, $CDCl_3$) spectrum of **6i**

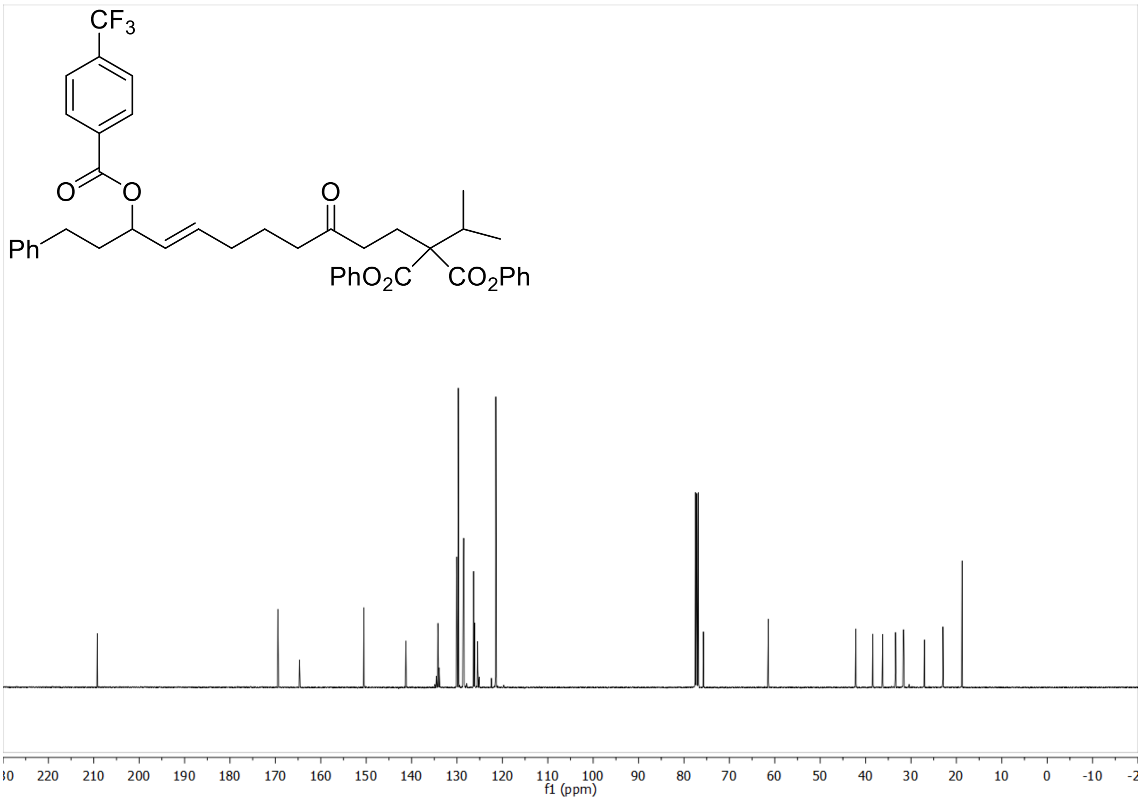

Supplementary Figure 119. ^{1}H NMR (400 MHz, $CDCl_3$) spectrum of **6j**

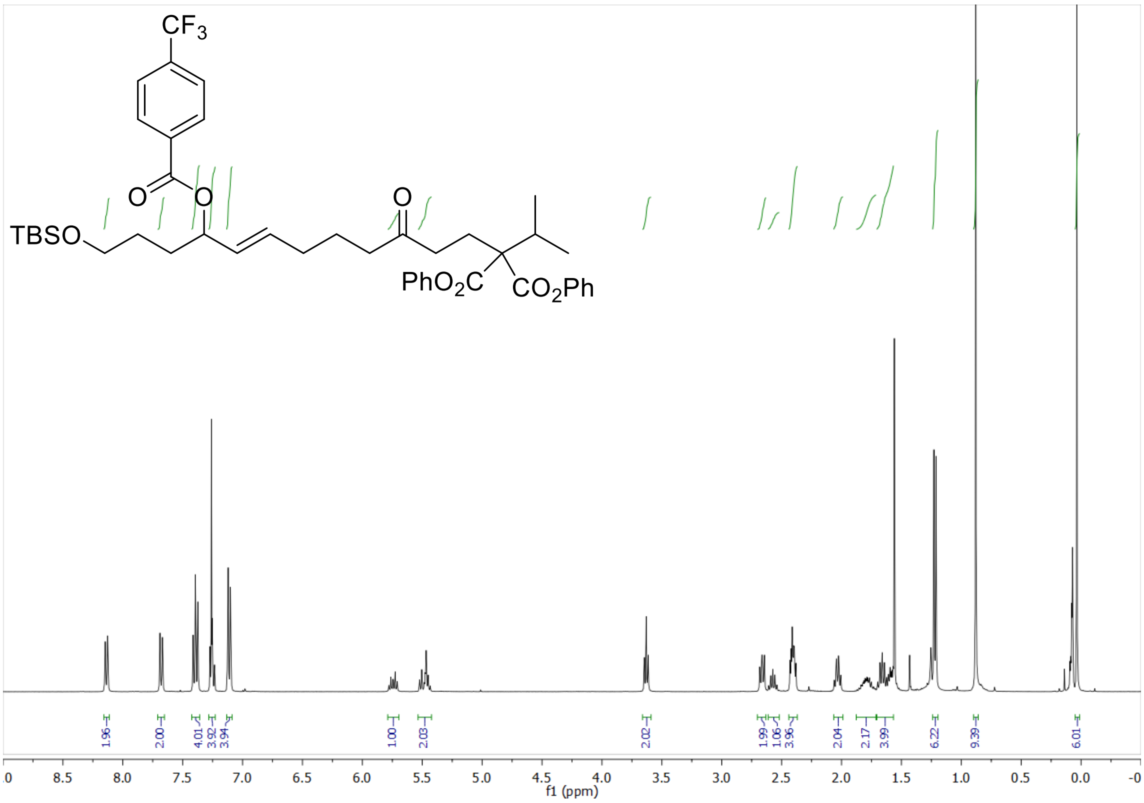

Supplementary Figure 120. ^{13}C NMR (100 MHz, $CDCl_3$) spectrum of **6j**

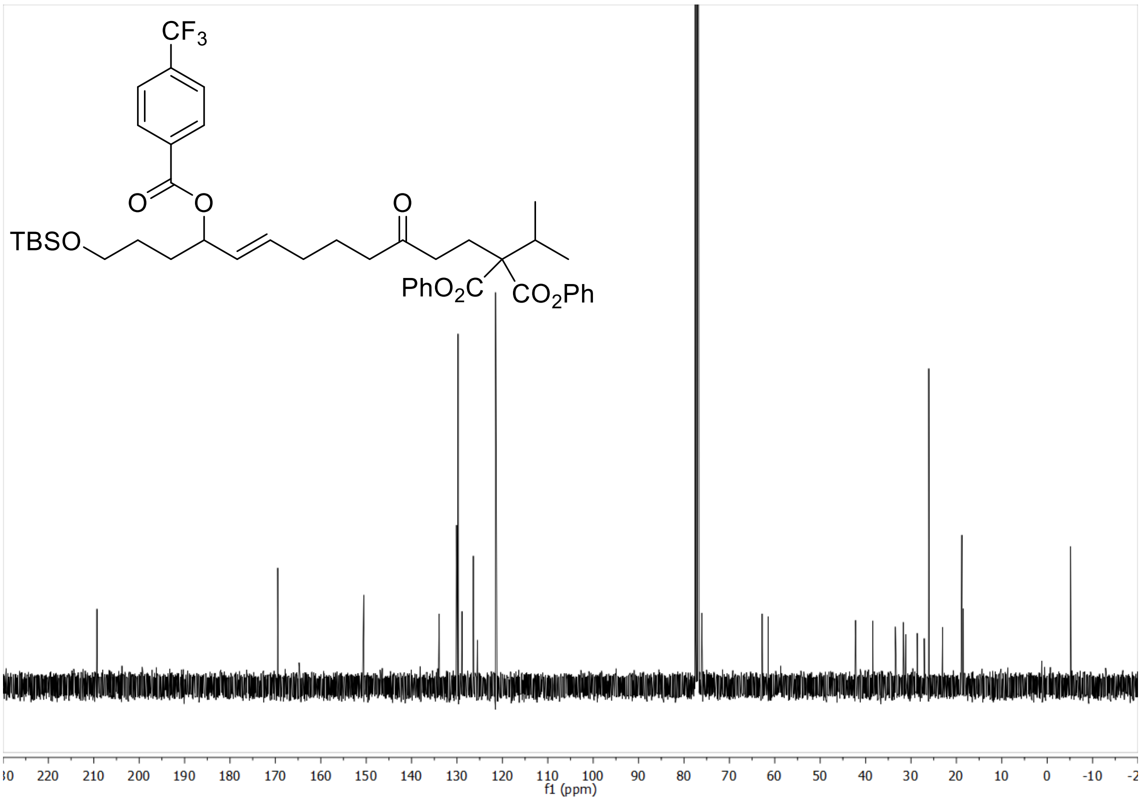

Supplementary Figure 121. ^{1}H NMR (400 MHz, $CDCl_3$) spectrum of **6k**

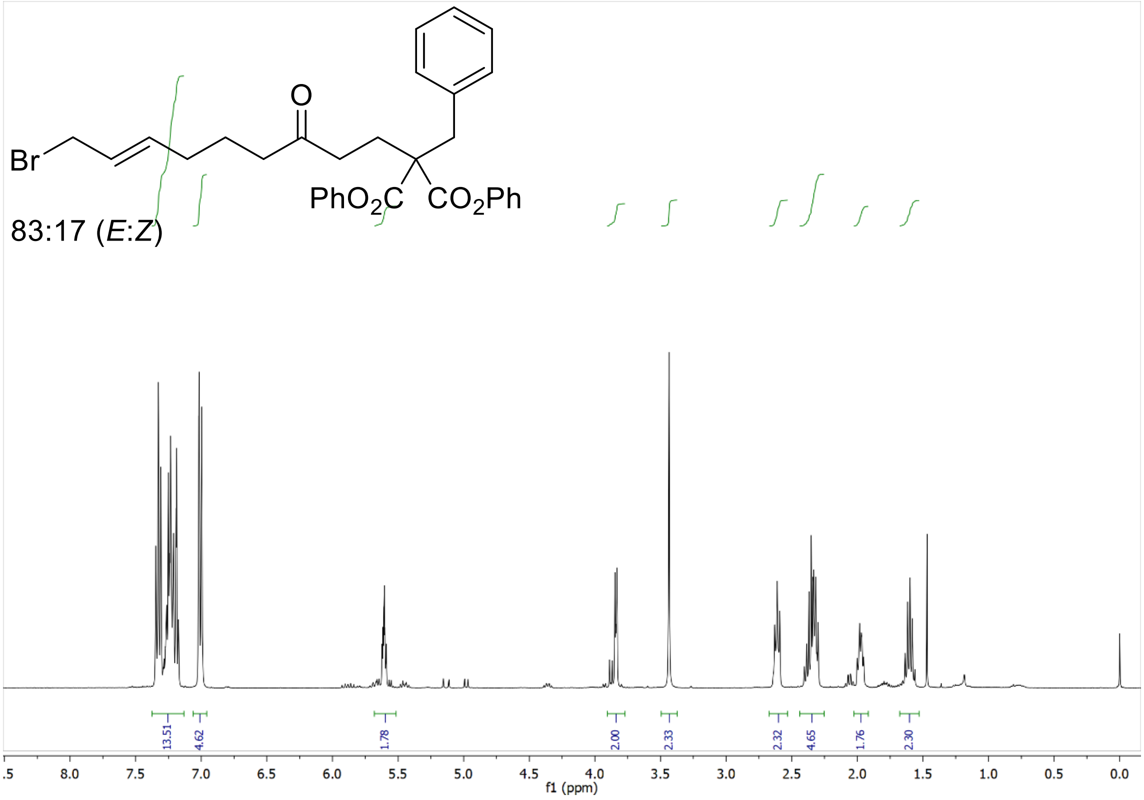

Supplementary Figure 122. ^{13}C NMR (100 MHz, $CDCl_3$) spectrum of **6k**

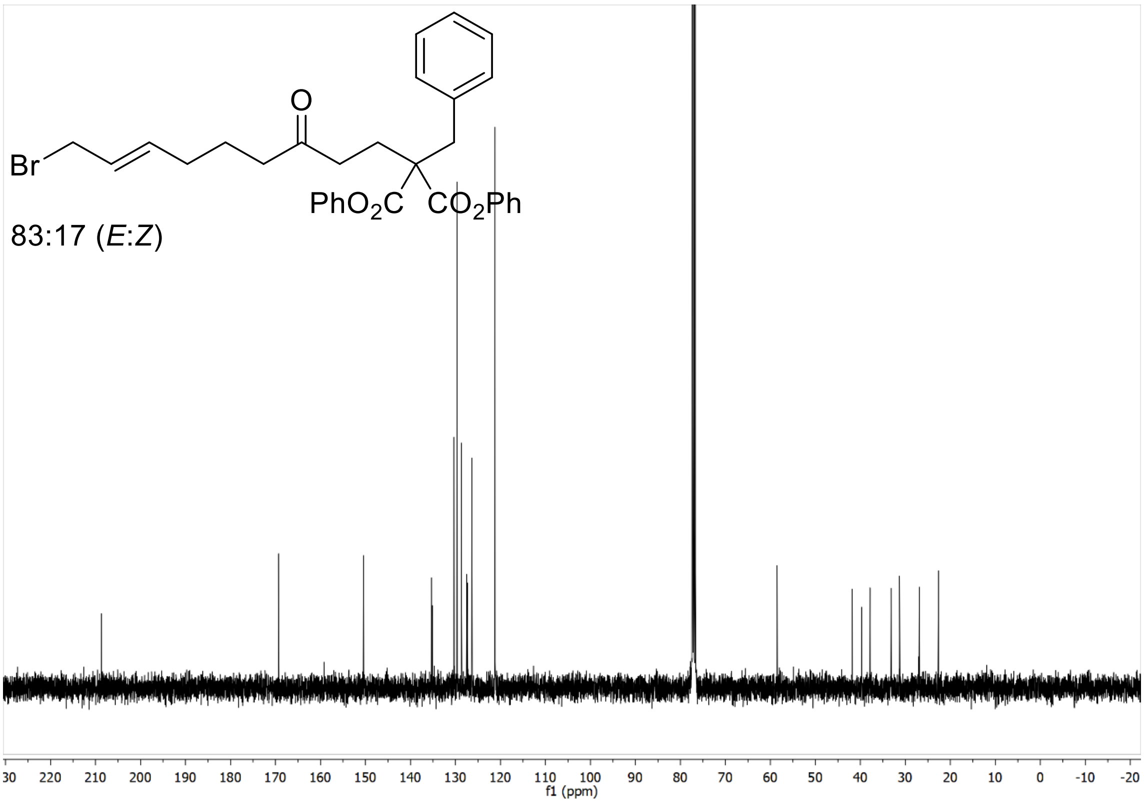

Supplementary Figure 123. ^{1}H NMR (500 MHz, $CDCl_3$) spectrum of **6l**

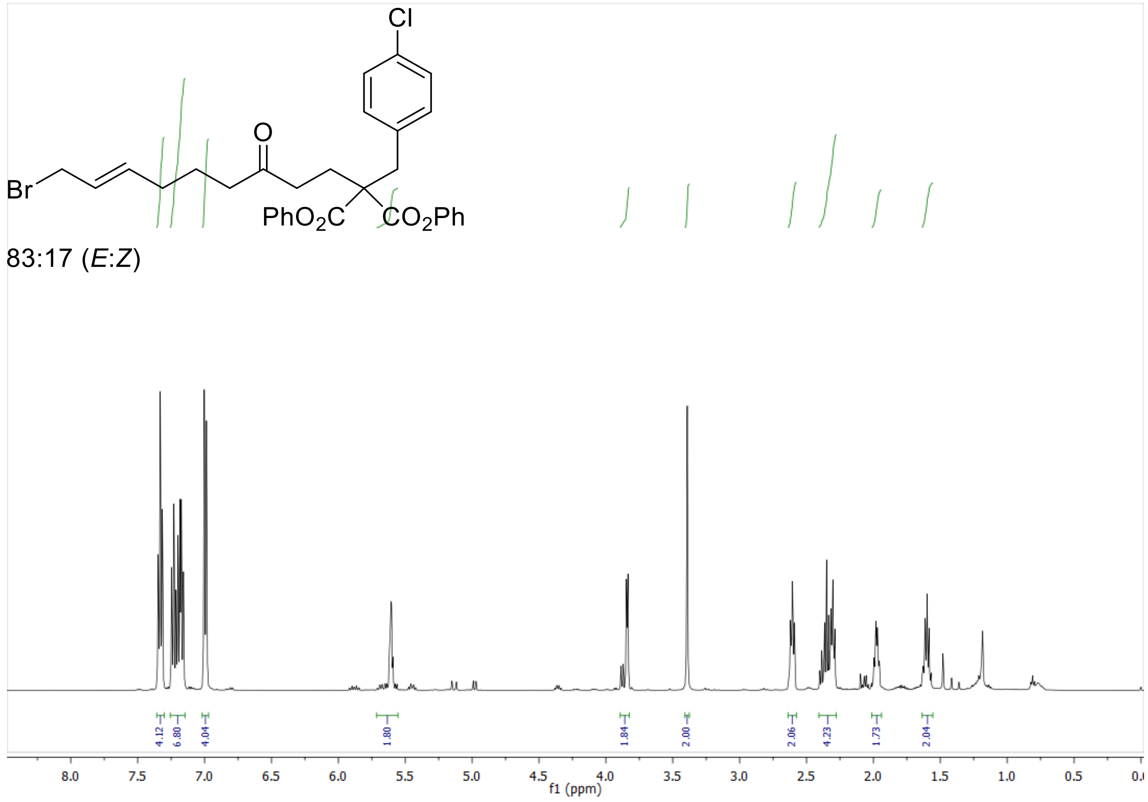

Supplementary Figure 124. ^{13}C NMR (125 MHz, $CDCl_3$) spectrum of **6l**

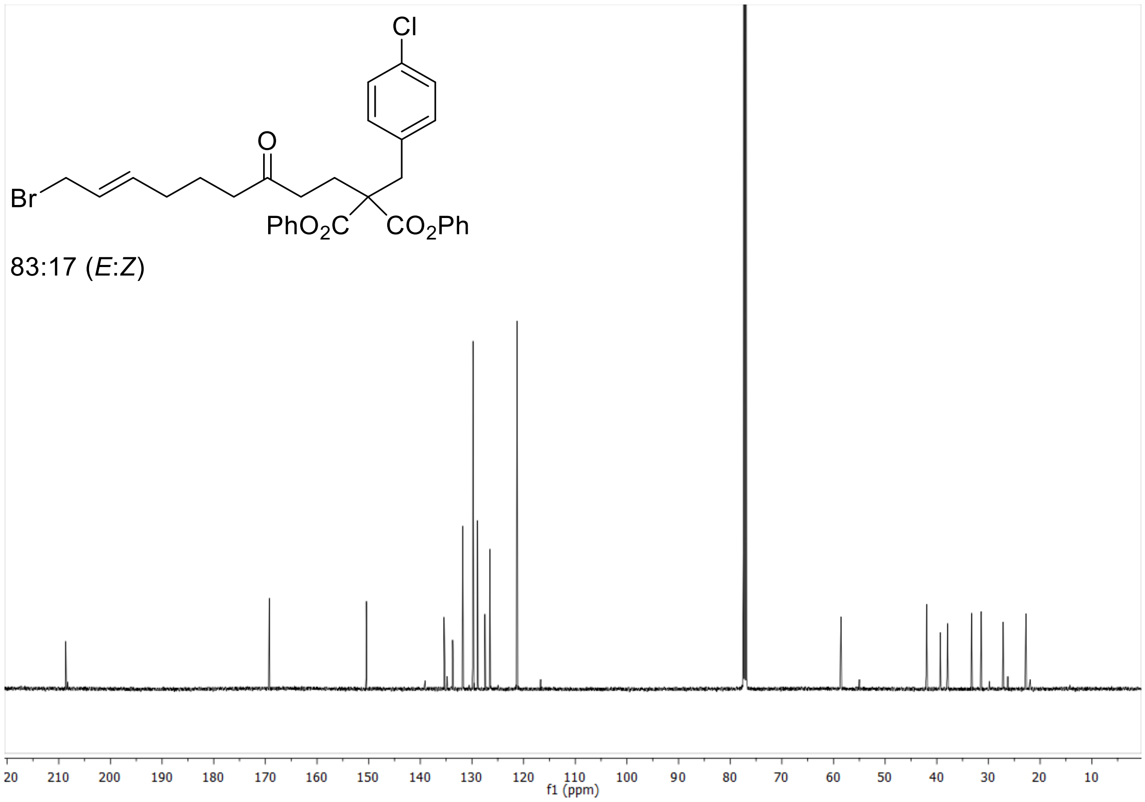

Supplementary Figure 125. ^{1}H NMR (400 MHz, $CDCl_3$) spectrum of **6m**

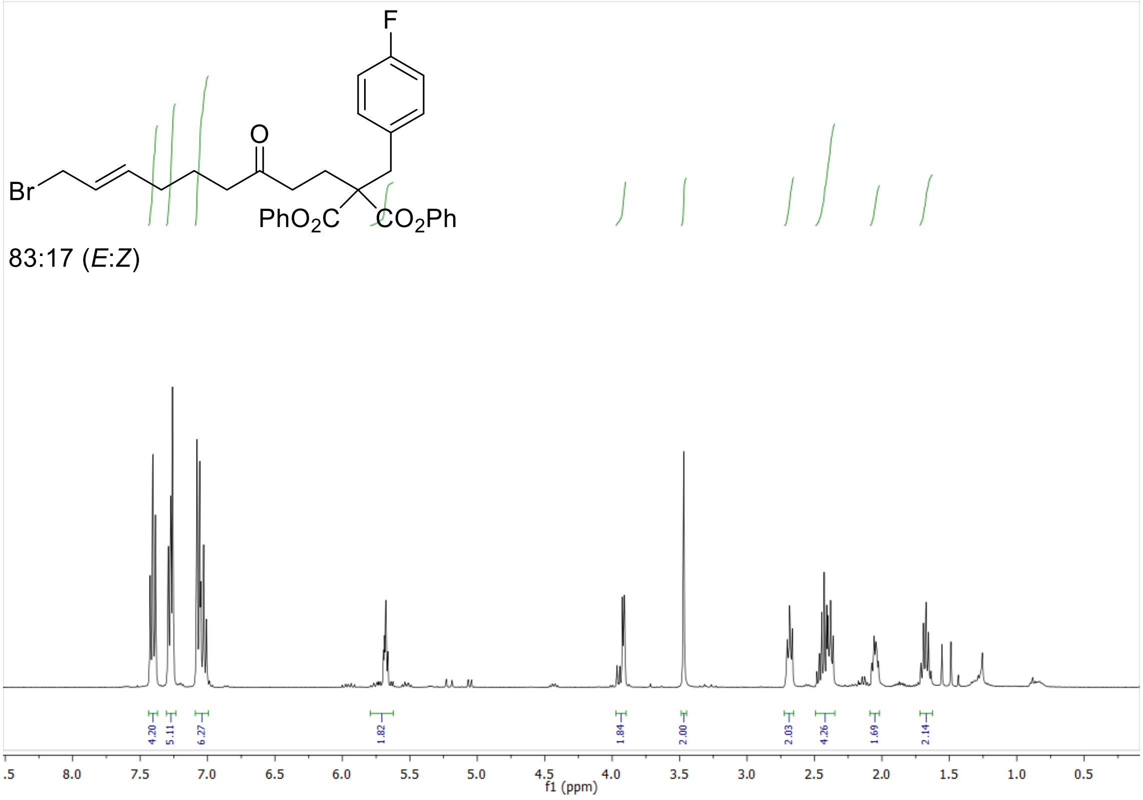

Supplementary Figure 126. ^{13}C NMR (125 MHz, $CDCl_3$) spectrum of **6m**

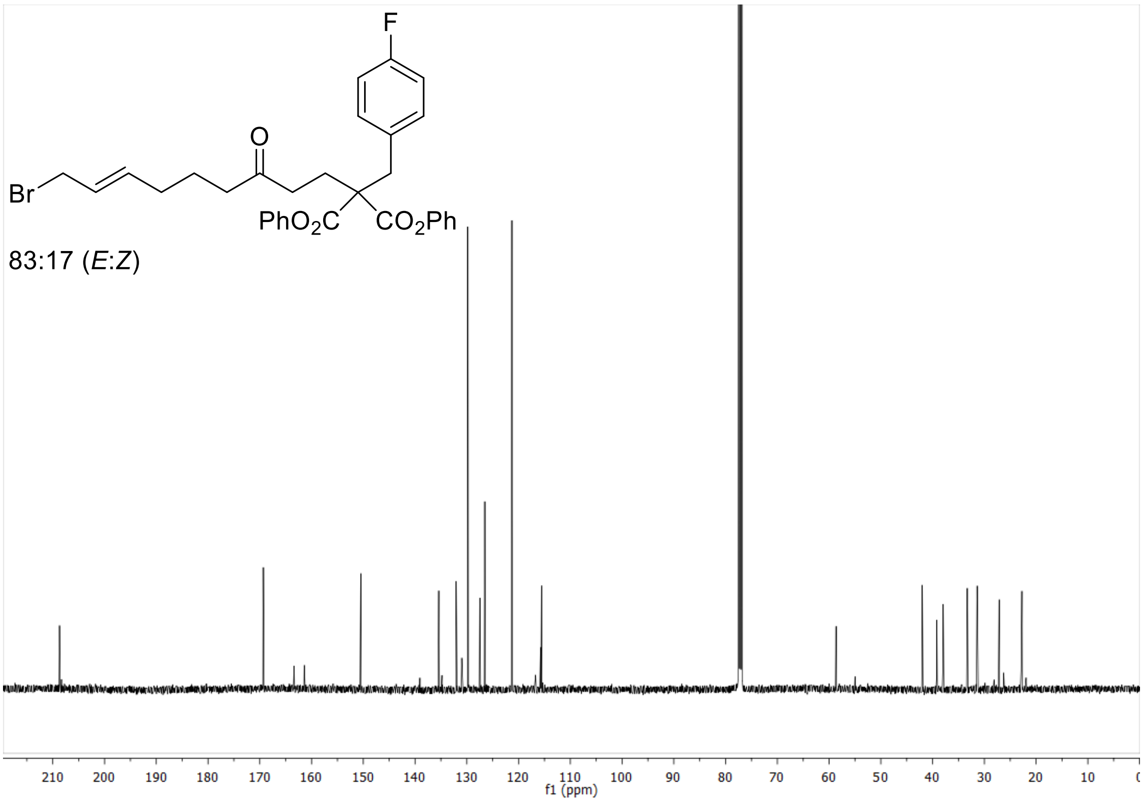

Supplementary Figure 127. ^{1}H NMR (500 MHz, $CDCl_3$) spectrum of **6n**

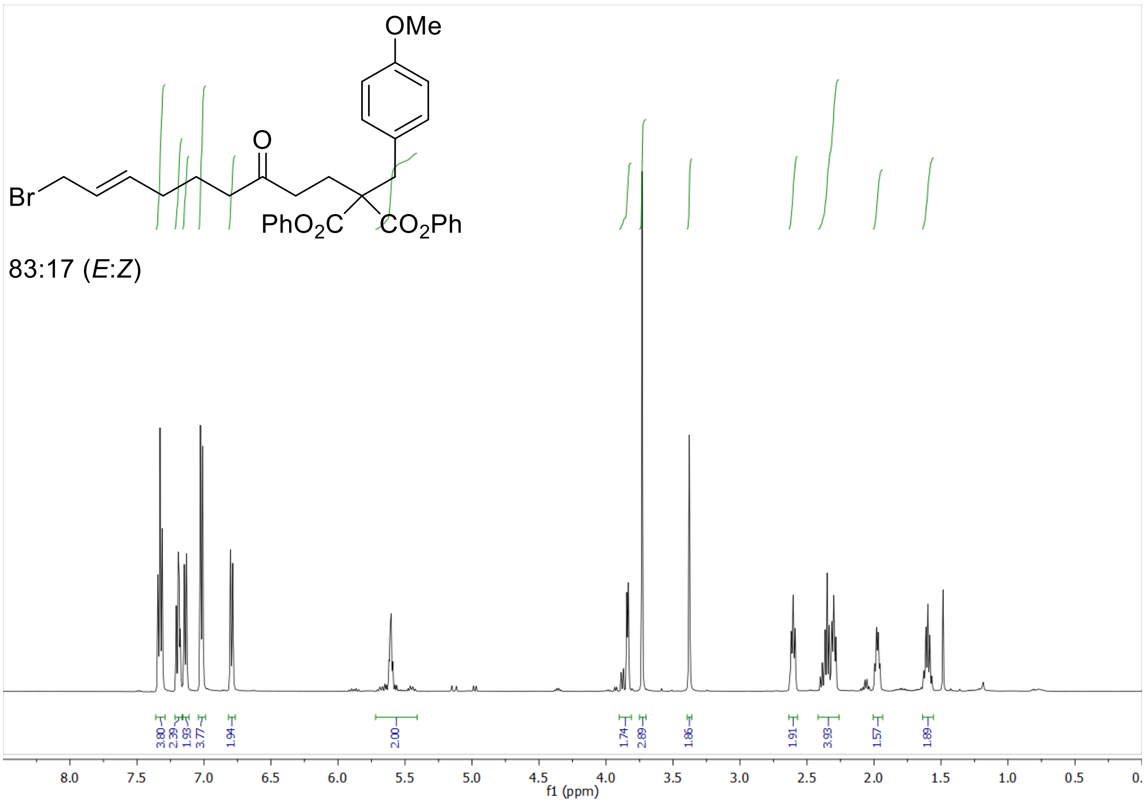

Supplementary Figure 128. ^{13}C NMR (125 MHz, $CDCl_3$) spectrum of **6n**

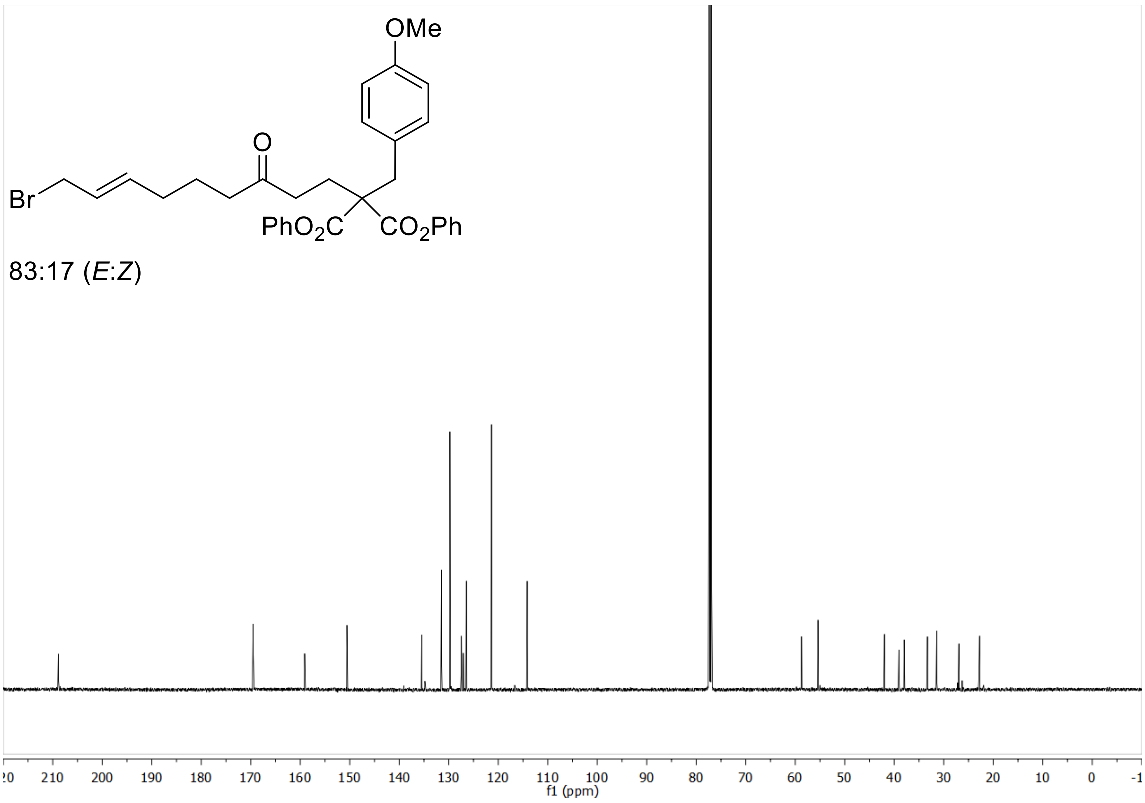

Supplementary Figure 129. ^{1}H NMR (500 MHz, $CDCl_3$) spectrum of **6o**

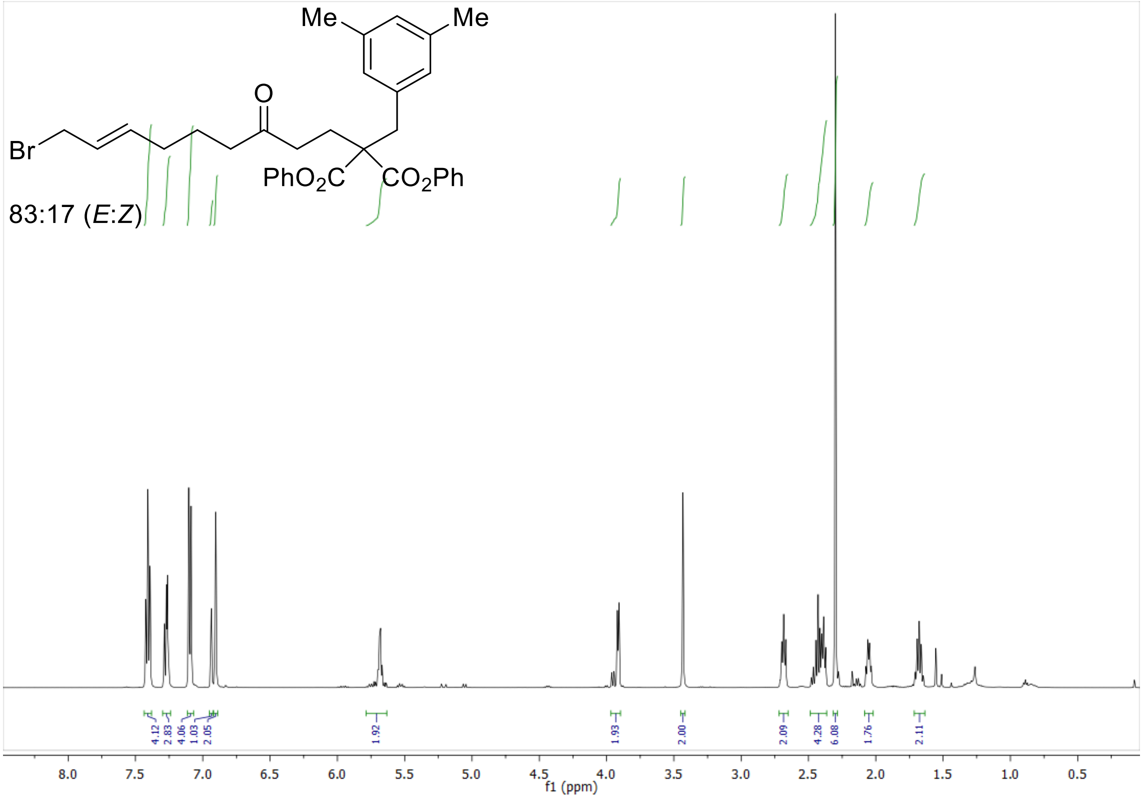

Supplementary Figure 130. ^{13}C NMR (125 MHz, $CDCl_3$) spectrum of **6o**

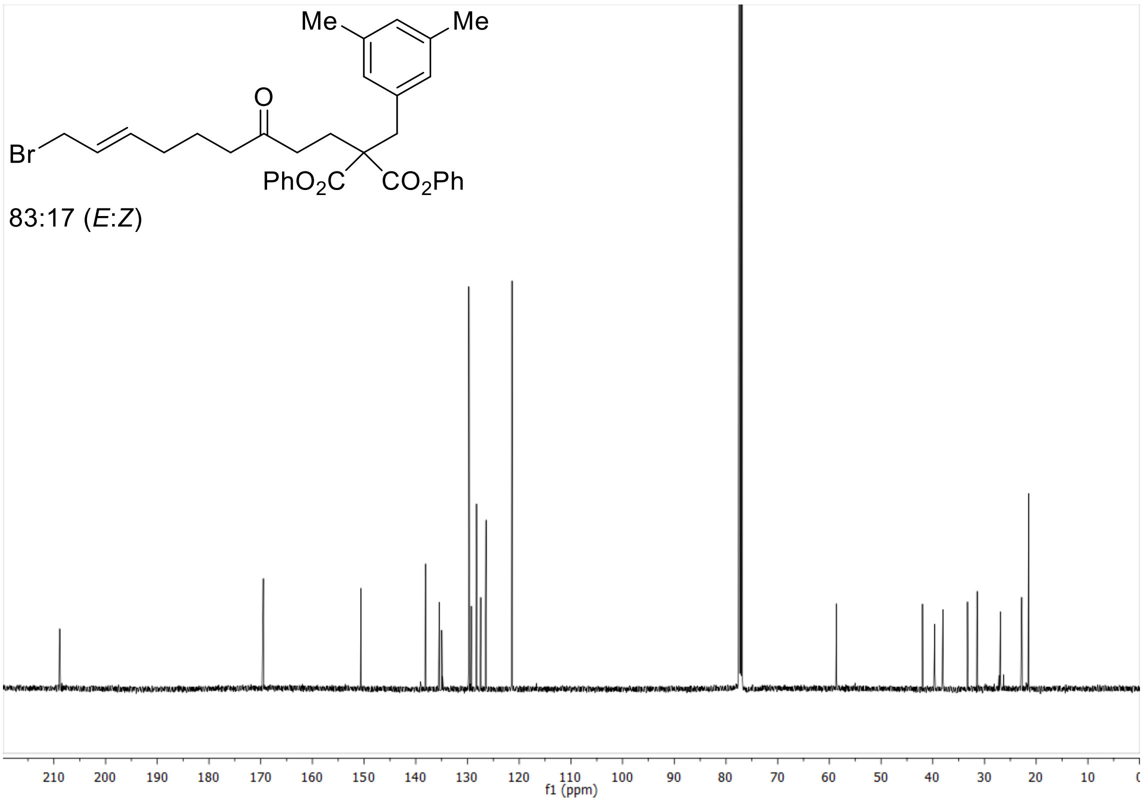

Supplementary Figure 131. ^{1}H NMR (400 MHz, $CDCl_3$) spectrum of **6p**

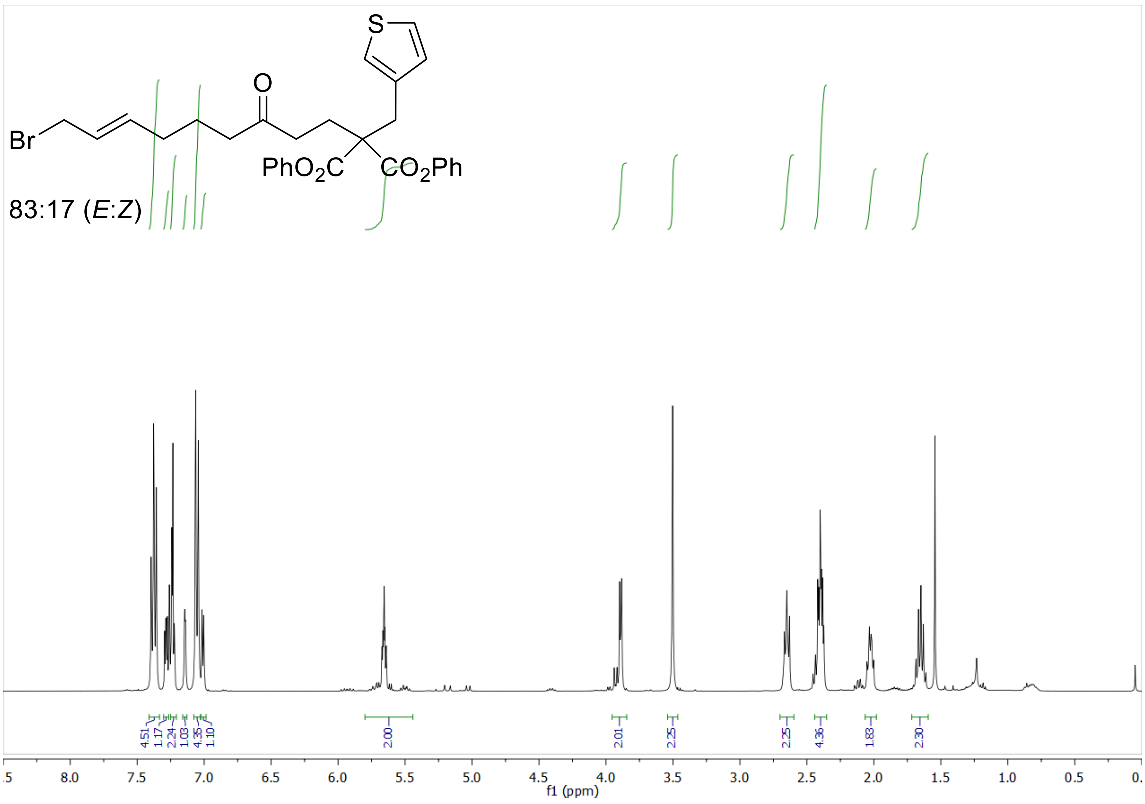

Supplementary Figure 132. ^{13}C NMR (100 MHz, $CDCl_3$) spectrum of **6p**

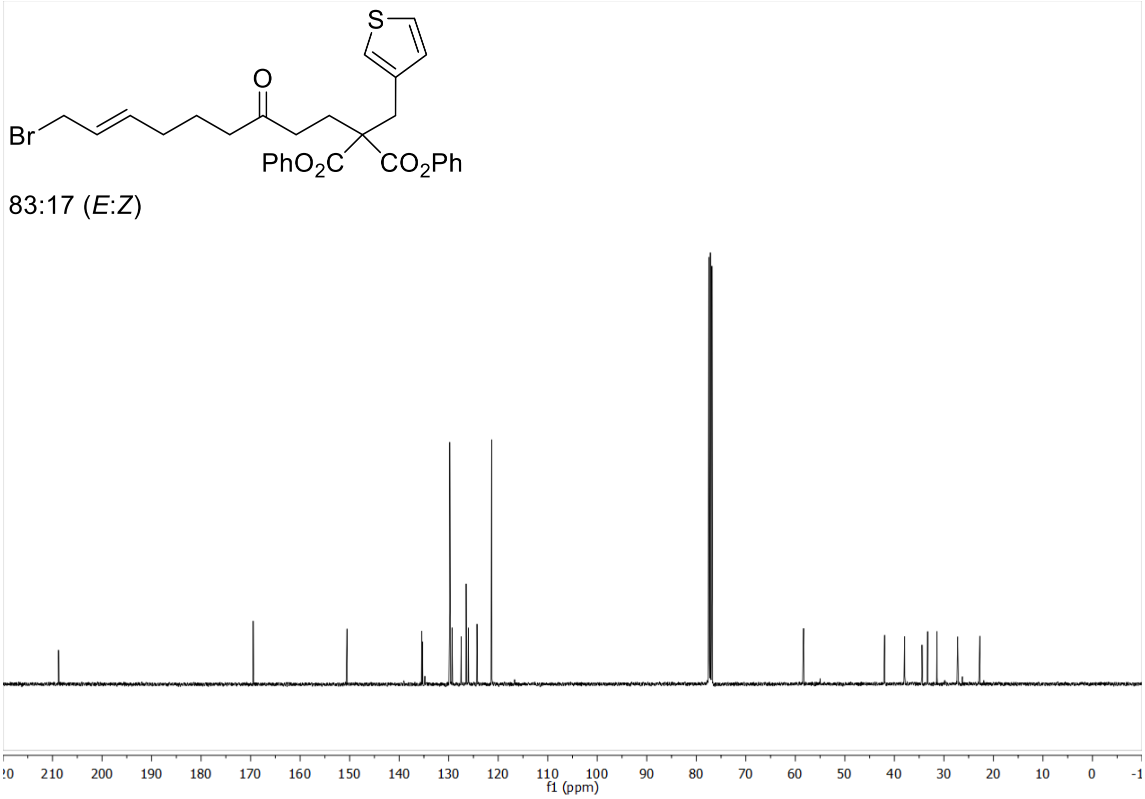

Supplementary Figure 133. ^{1}H NMR (500 MHz, $CDCl_3$) spectrum of **6q**

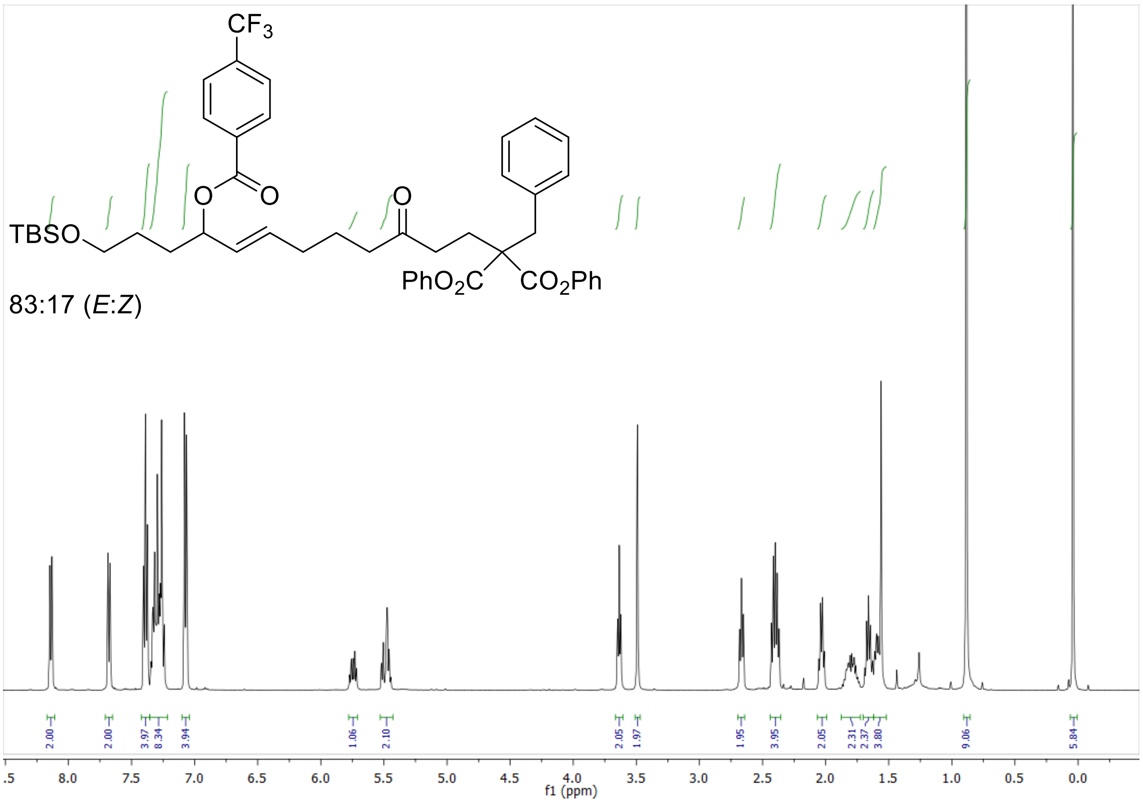

Supplementary Figure 134. ^{13}C NMR (125 MHz, $CDCl_3$) spectrum of **6q**

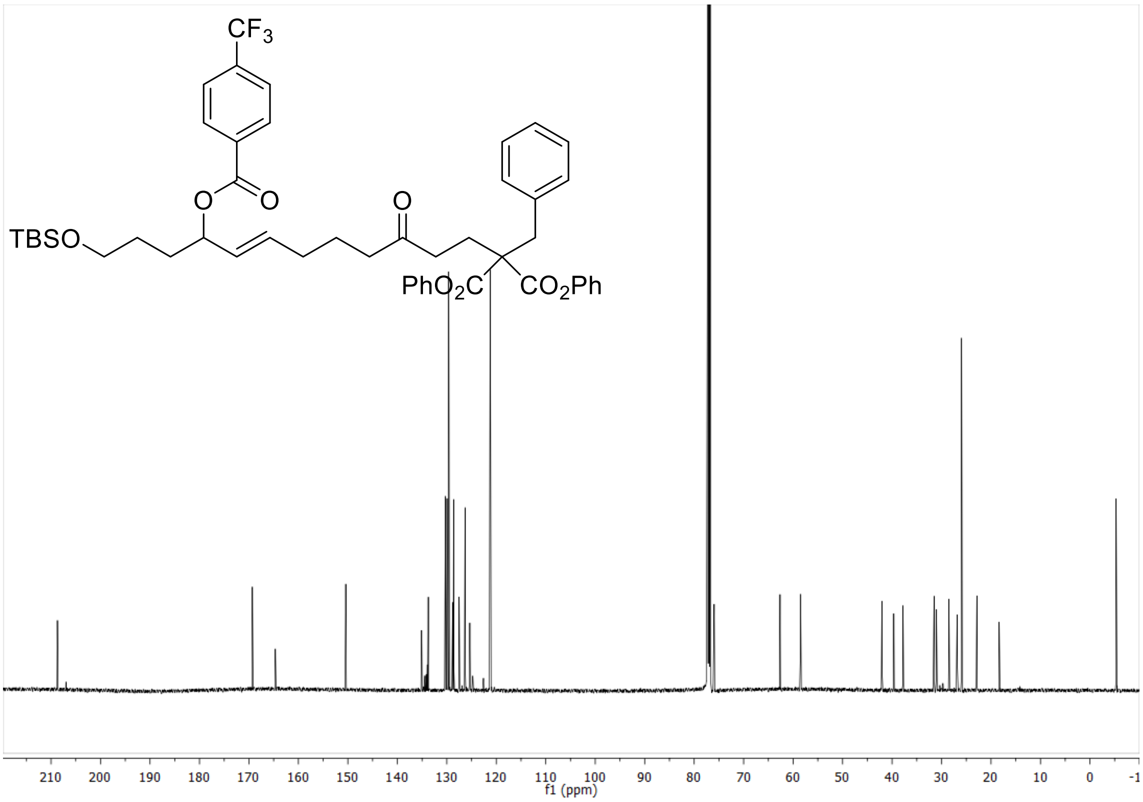

Supplementary Figure 135. ^{1}H NMR (400 MHz, $CDCl_3$) spectrum of ***D*-6e**

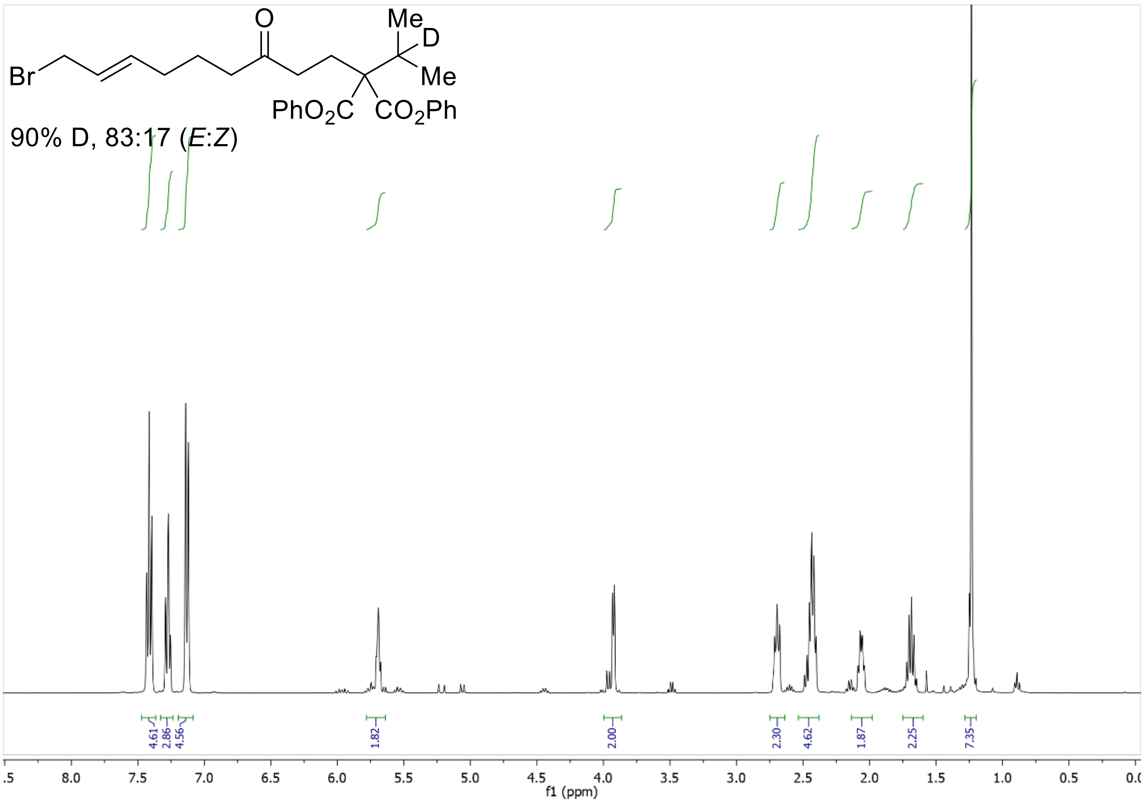

Supplementary Figure 136. ^{13}C NMR (100 MHz, $CDCl_3$) spectrum of ***D*-6e**

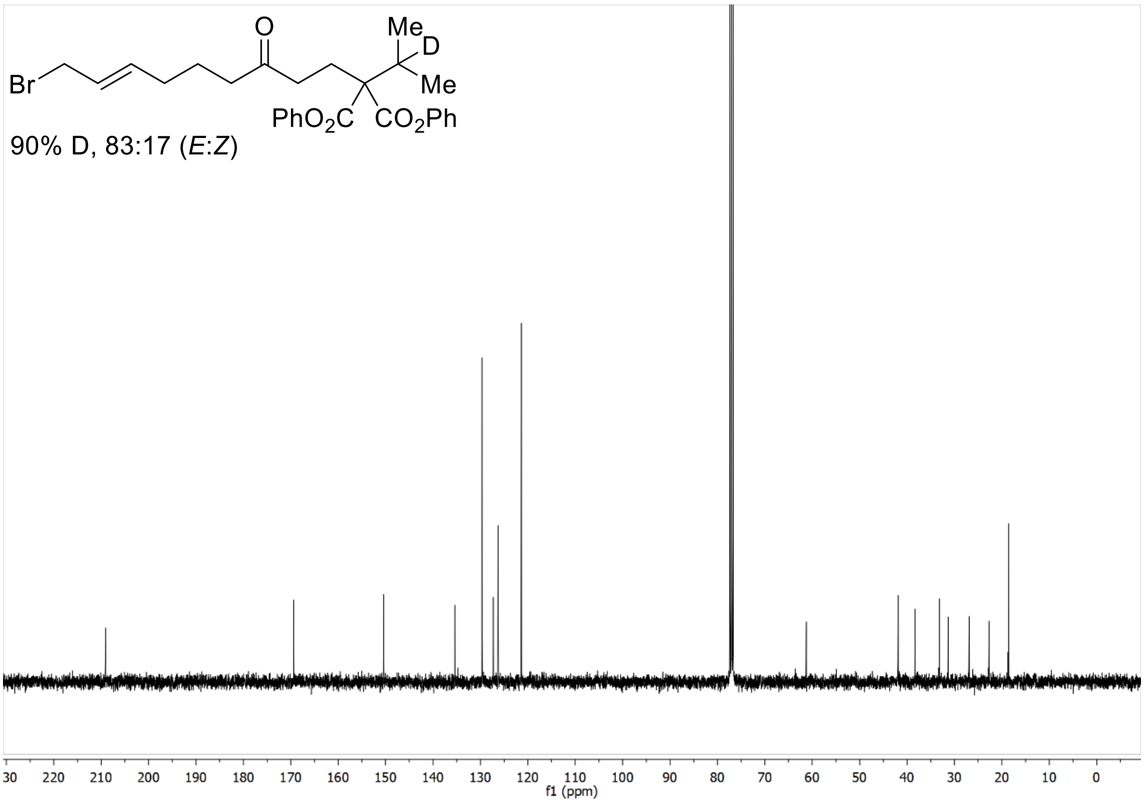

Supplementary Figure 137. ^{1}H NMR (400 MHz, $CDCl_3$) spectrum of **7a'**

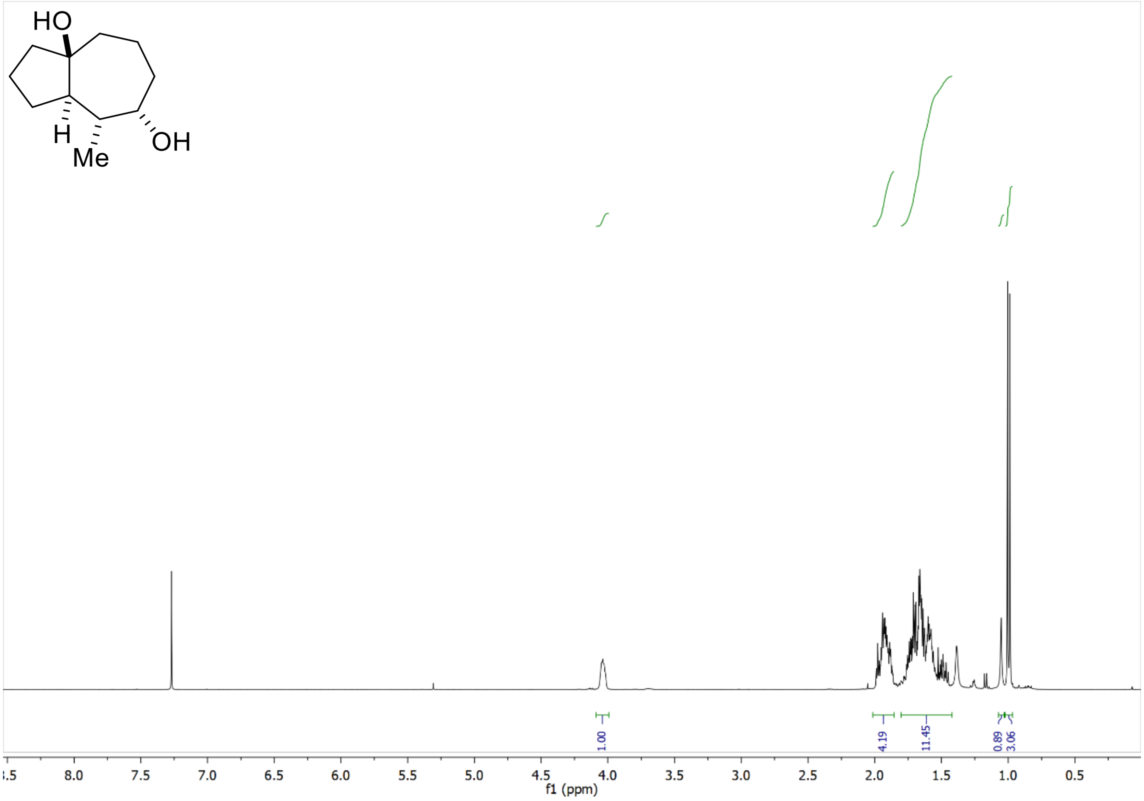

Supplementary Figure 138. ^{13}C NMR (100 MHz, $CDCl_3$) spectrum of **7a'**

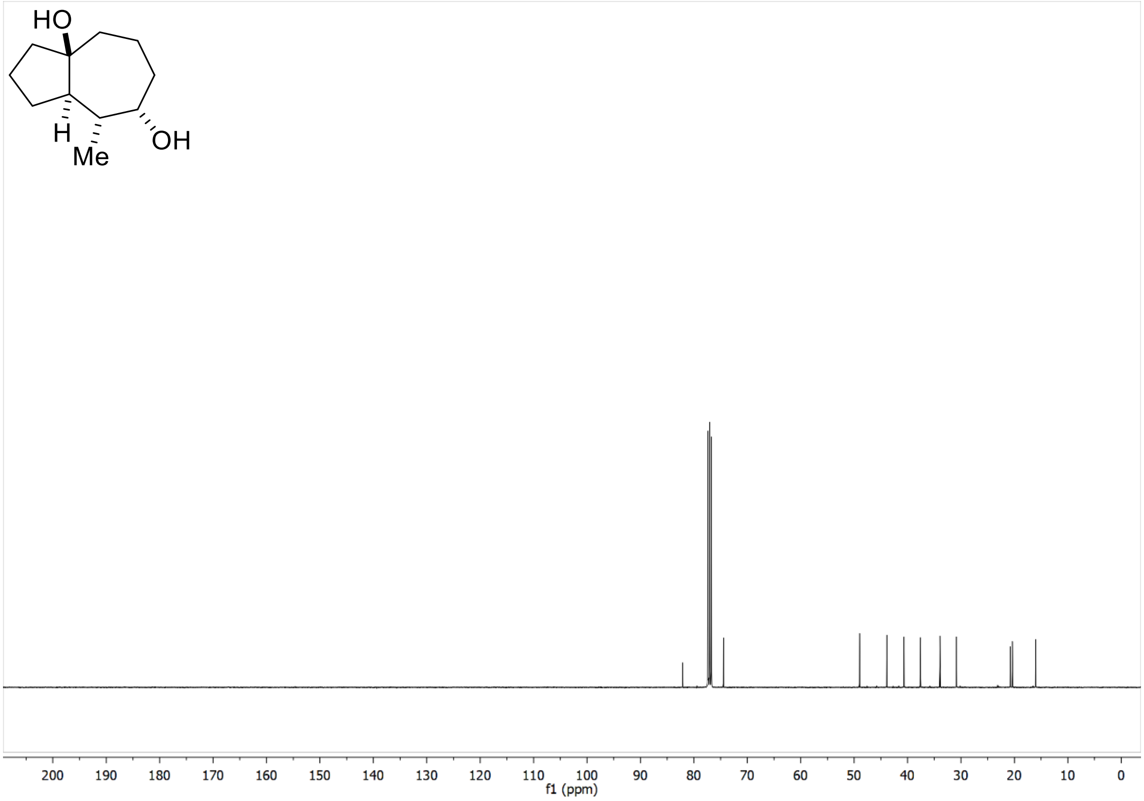

Supplementary Figure 139. ^{1}H NMR (400 MHz, $CDCl_3$) spectrum of **7b'**

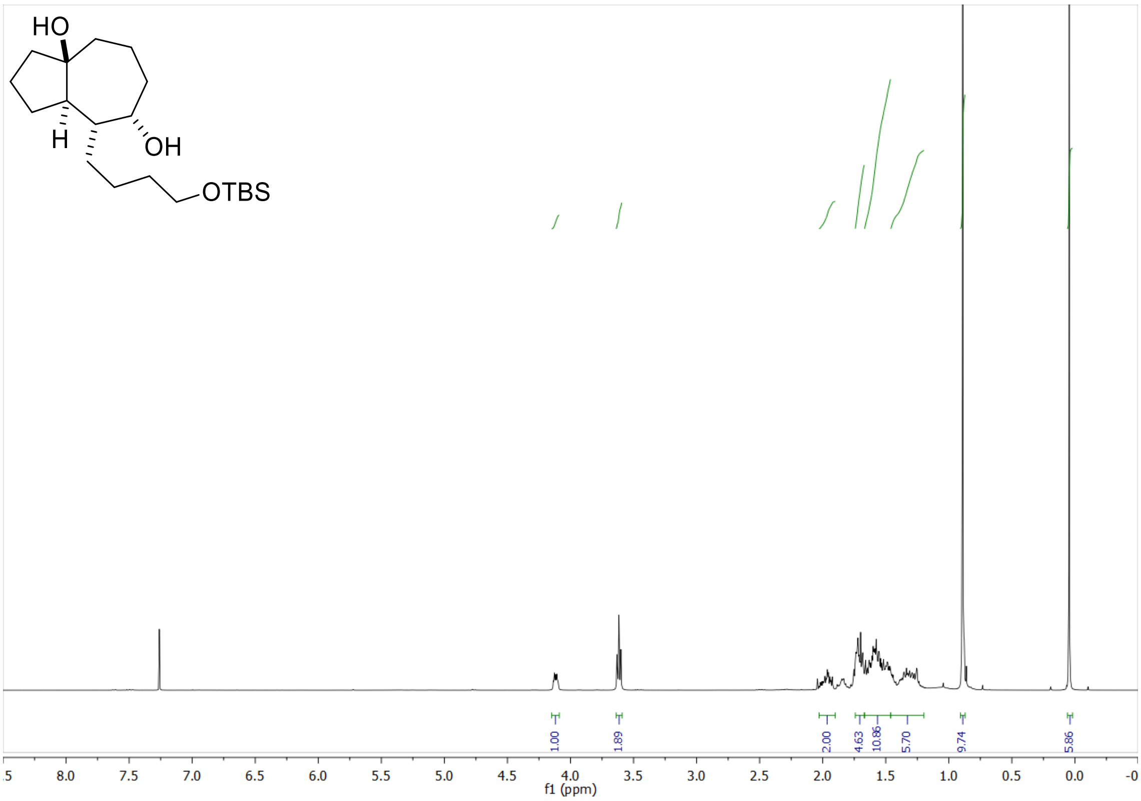

Supplementary Figure 140. ^{13}C NMR (100 MHz, $CDCl_3$) spectrum of **7b'**

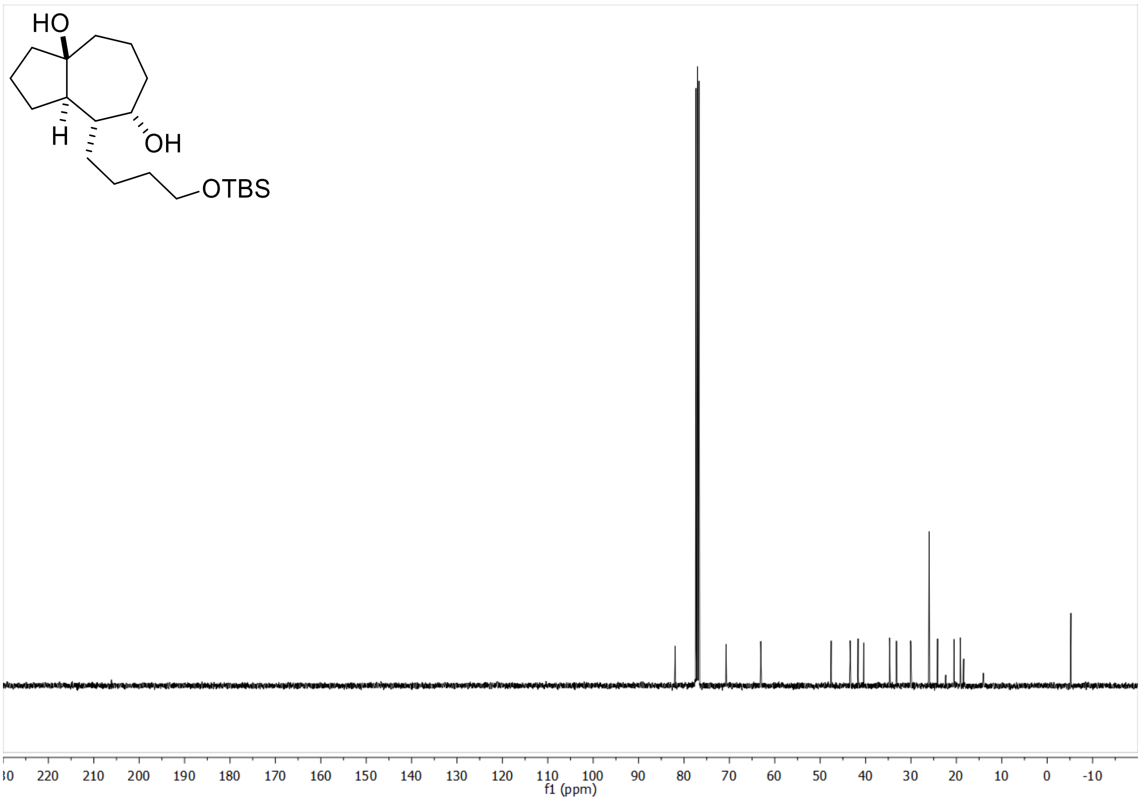

Supplementary Figure 141. ^{1}H NMR (400 MHz, $CDCl_3$) spectrum of **7c'**

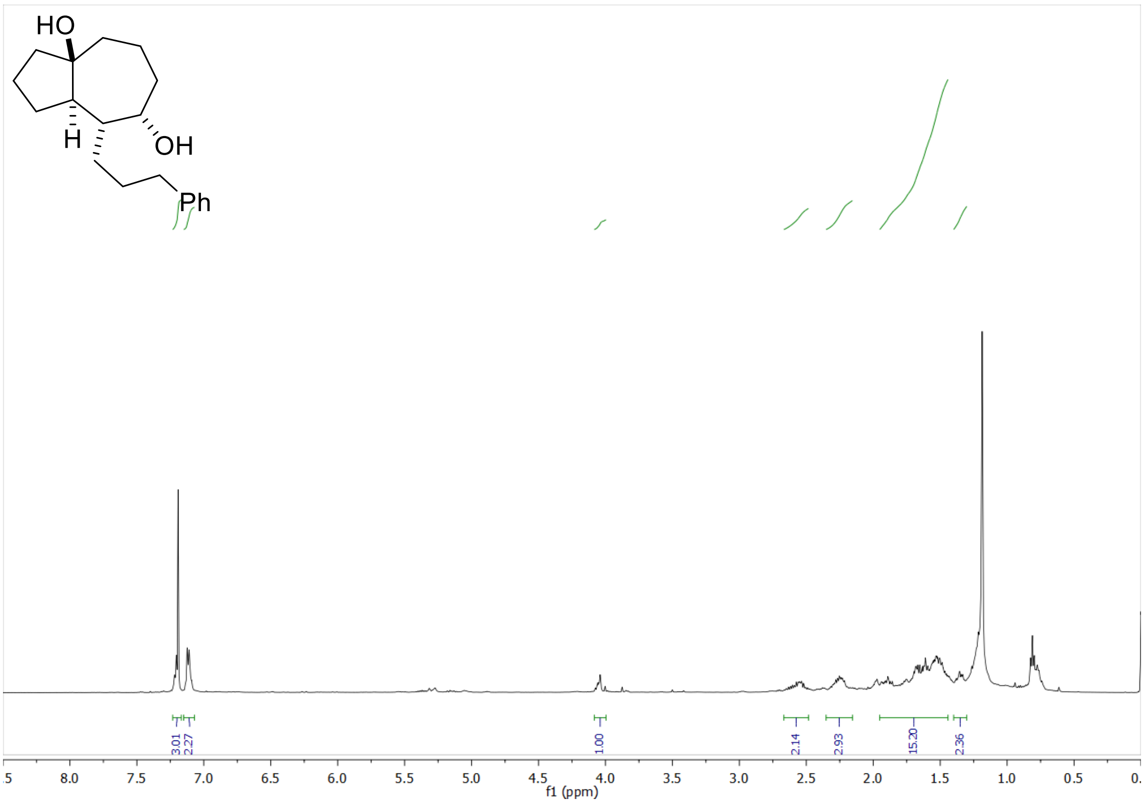

Supplementary Figure 142. ^{13}C NMR (100 MHz, $CDCl_3$) spectrum of **7c'**

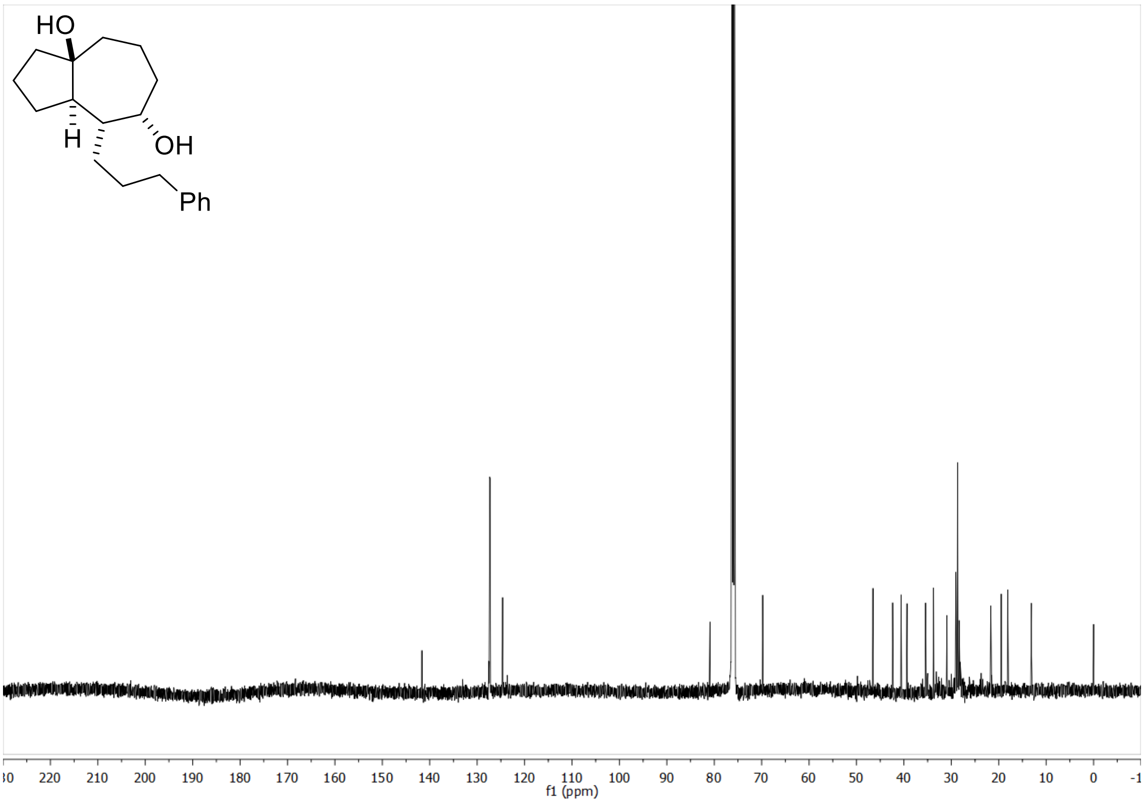

Supplementary Figure 143. ^{1}H NMR (400 MHz, $CDCl_3$) spectrum of **7d'**

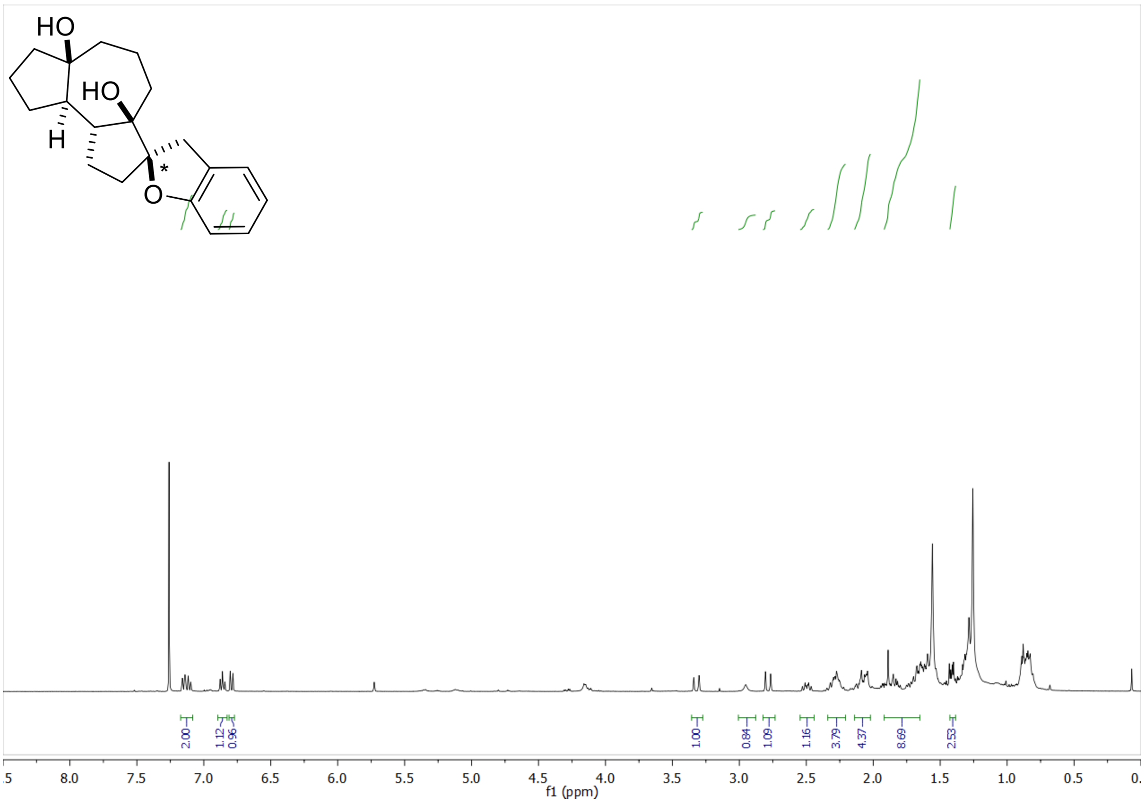

Supplementary Figure 144. ^{13}C NMR (100 MHz, $CDCl_3$) spectrum of **7d'**

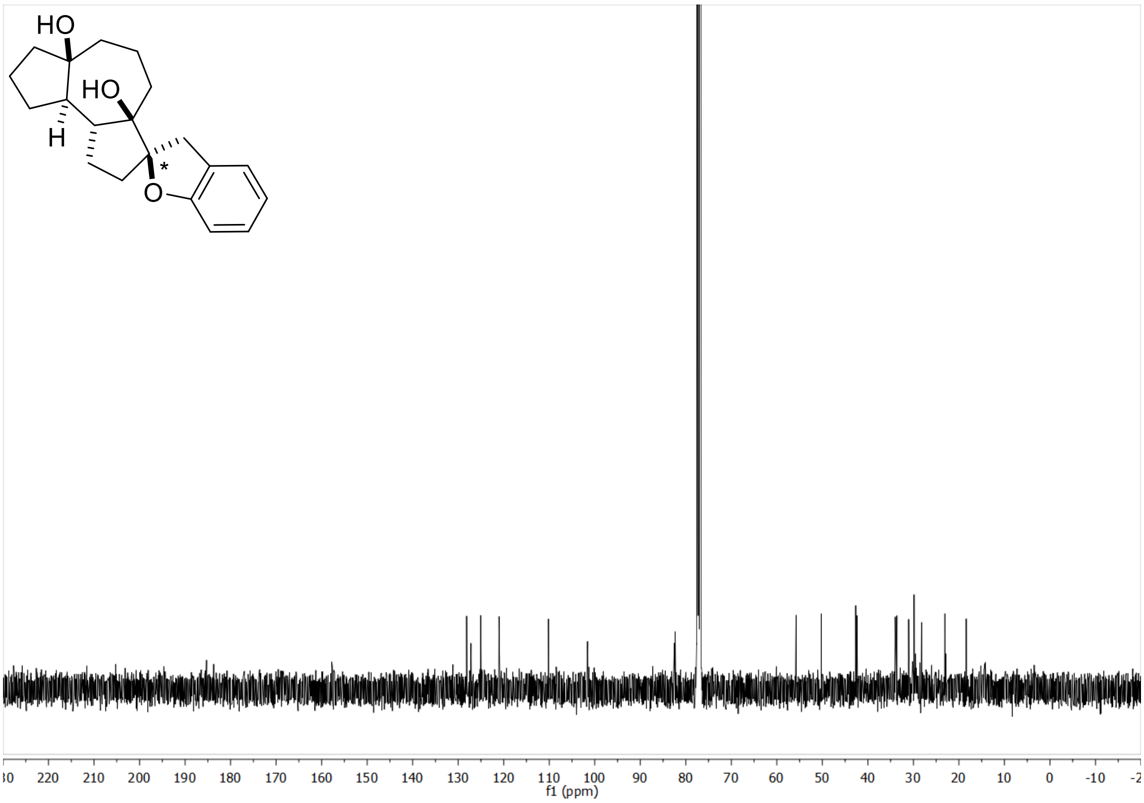

Supplementary Figure 145. ^{1}H NMR (400 MHz, $CDCl_3$) spectrum of **S45**

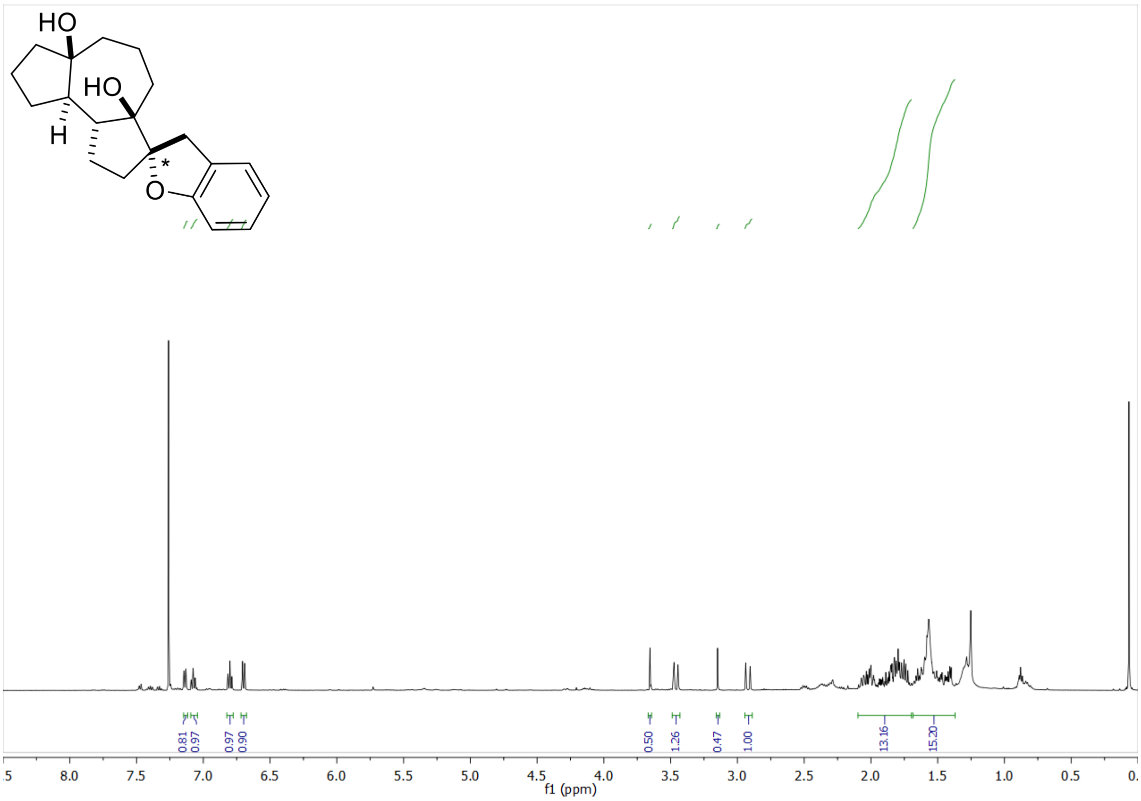

Supplementary Figure 146. ^{13}C NMR (100 MHz, $CDCl_3$) spectrum of **S45**

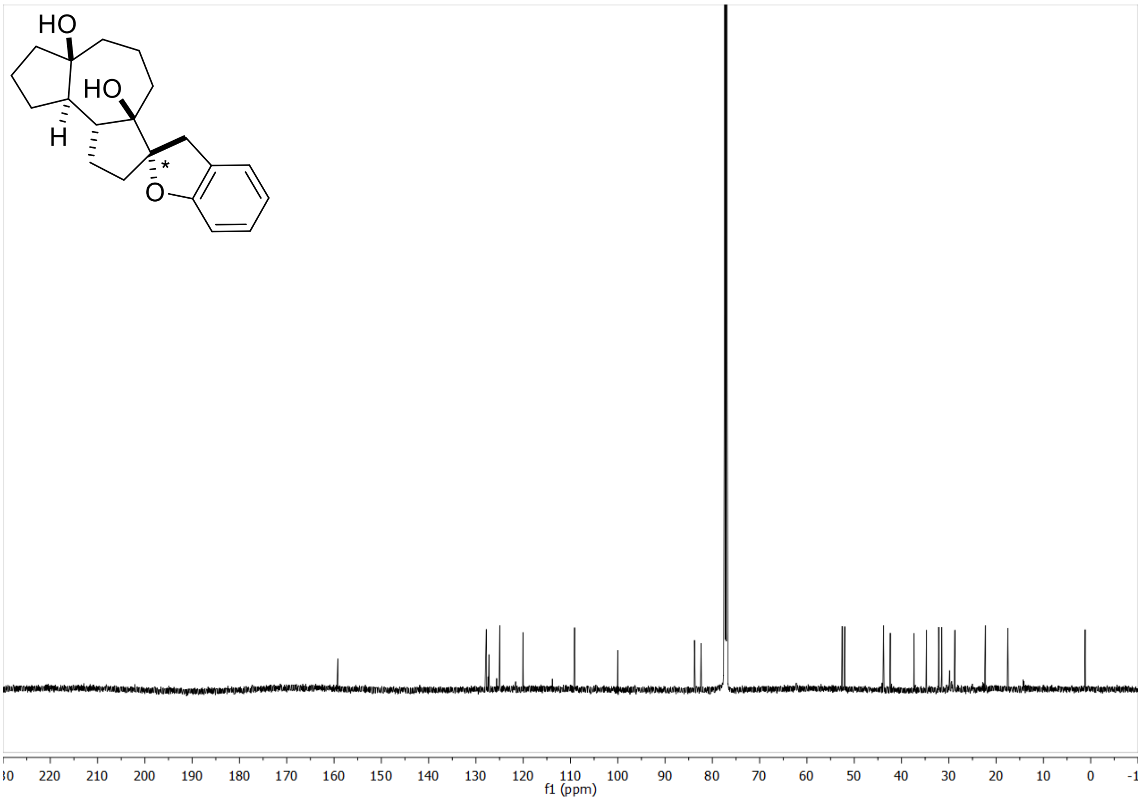

Supplementary Figure 147. ^{1}H NMR (400 MHz, $CDCl_3$) spectrum of **7a**

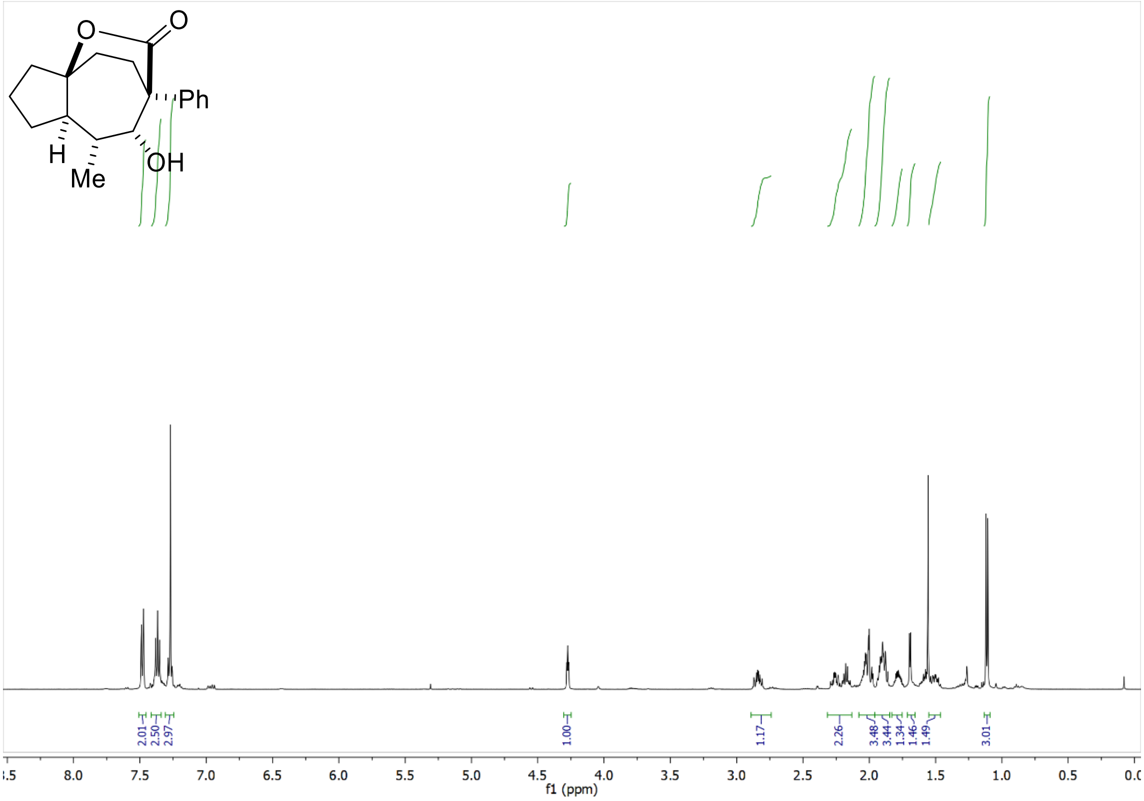

Supplementary Figure 148. ^{13}C NMR (100 MHz, $CDCl_3$) spectrum of **7a**

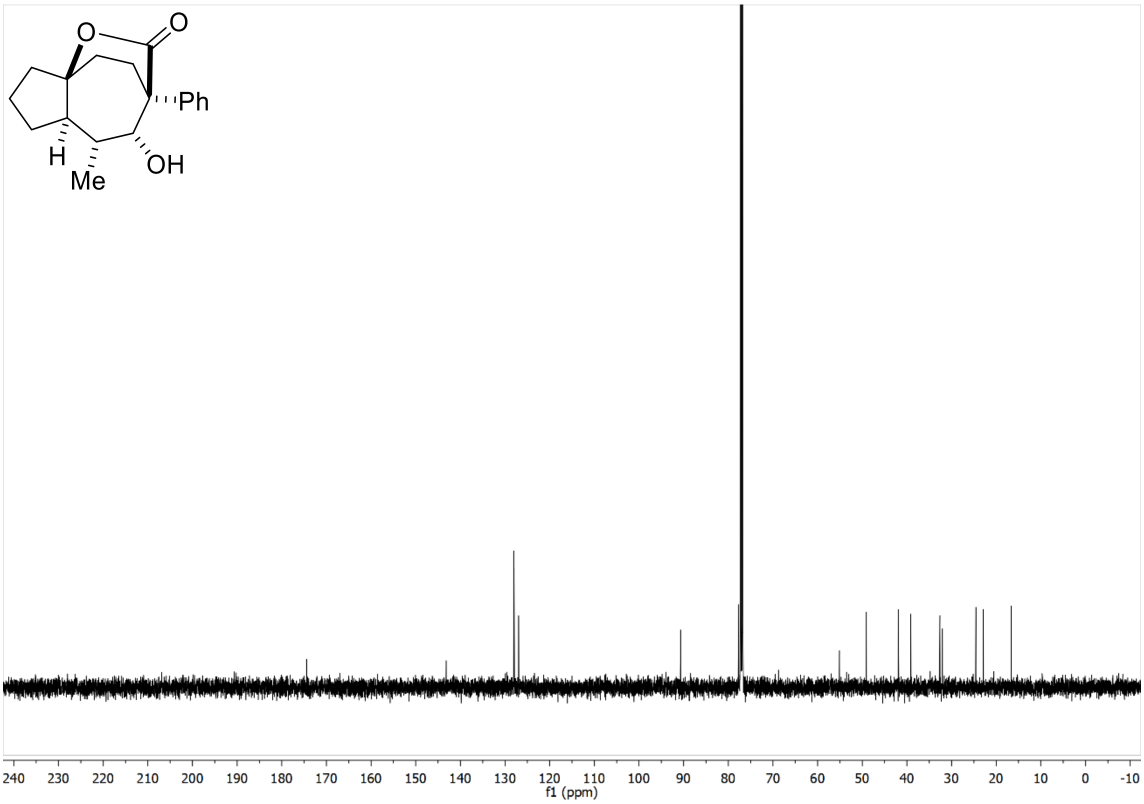

Supplementary Figure 149. ^{1}H NMR (400 MHz, $CDCl_3$) spectrum of **7b**

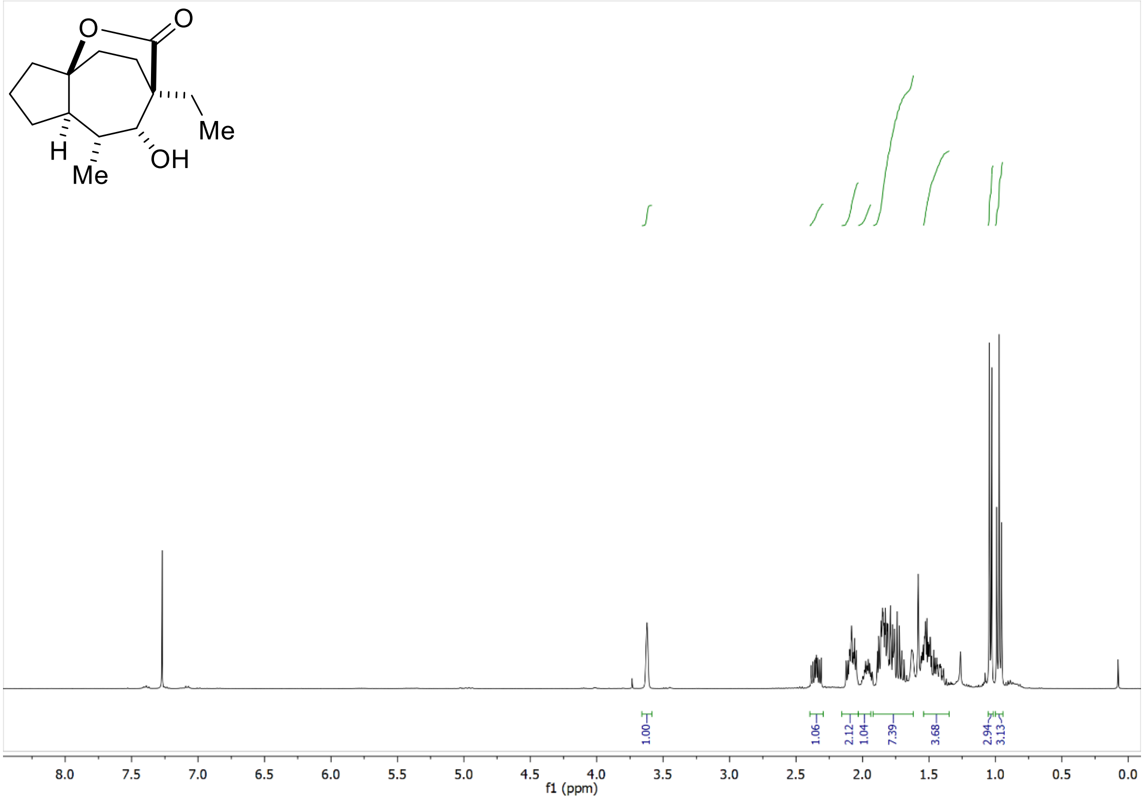

Supplementary Figure 150. ^{13}C NMR (100 MHz, $CDCl_3$) spectrum of **7b**

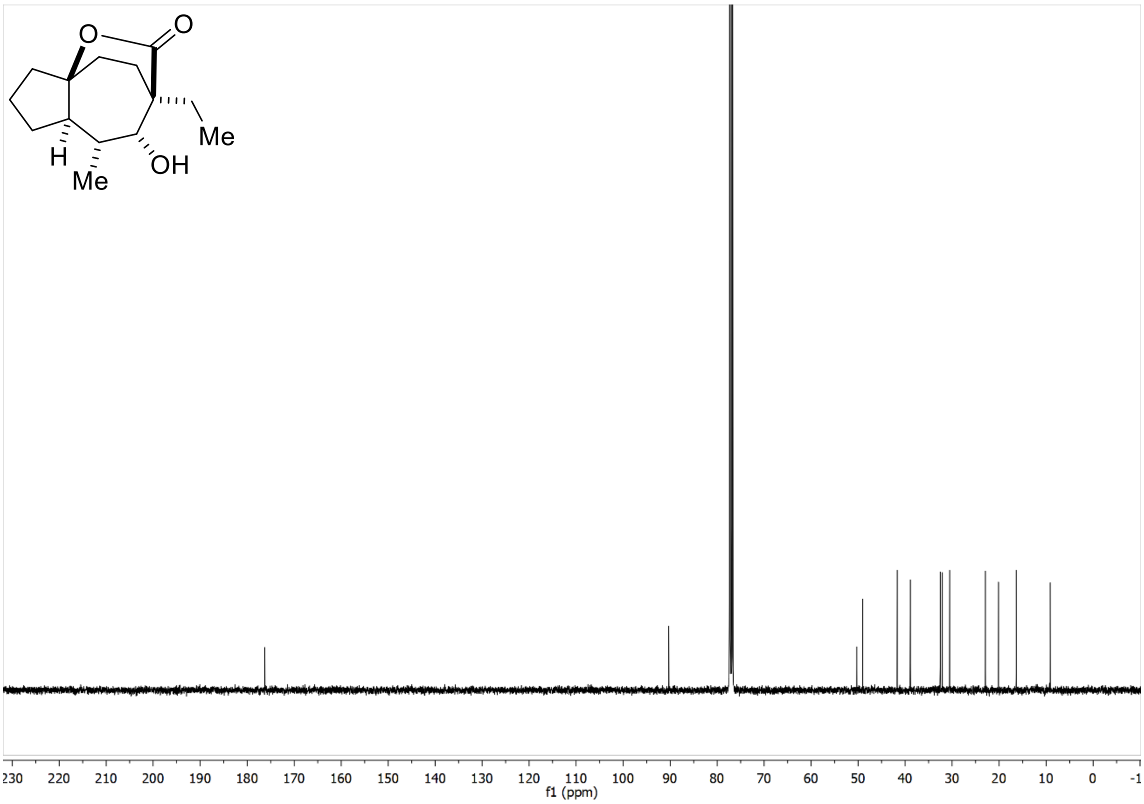

Supplementary Figure 151. ^{1}H NMR (500 MHz, $CDCl_3$) spectrum of **7c**

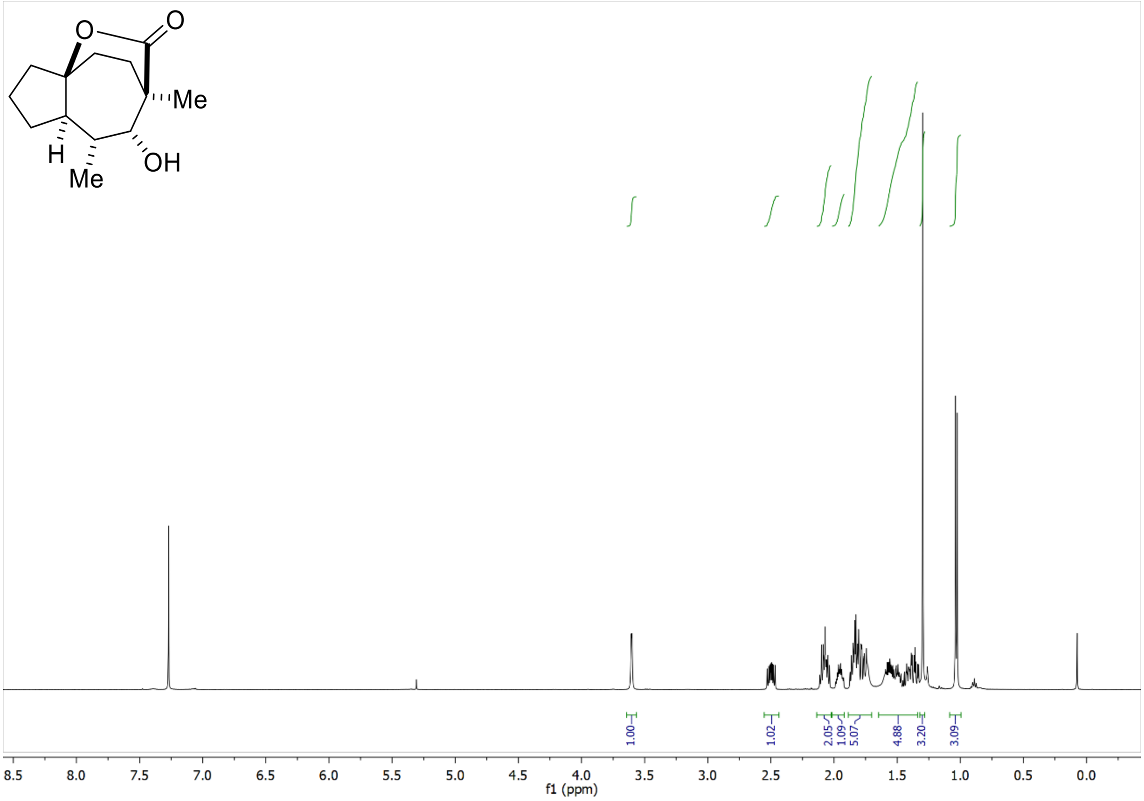

Supplementary Figure 152. ^{13}C NMR (125 MHz, $CDCl_3$) spectrum of **7c**

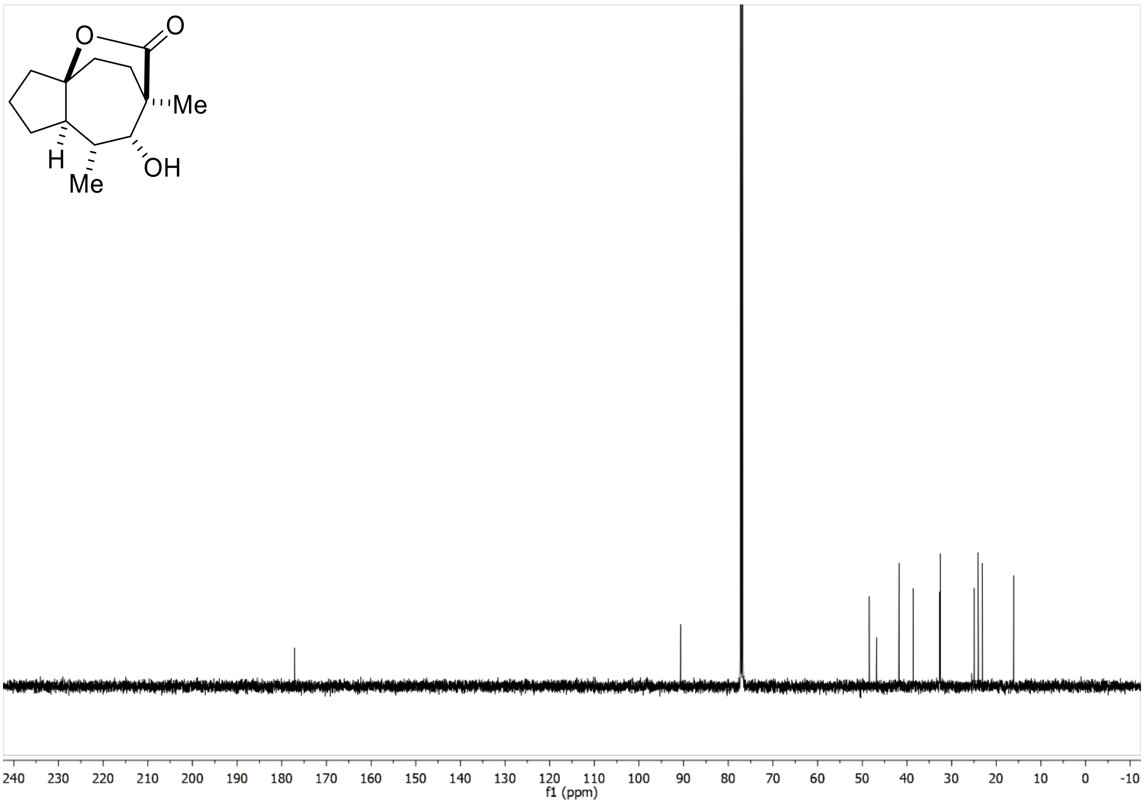

Supplementary Figure 153. ^{1}H NMR (400 MHz, CDCl$_3$) spectrum of **7d**

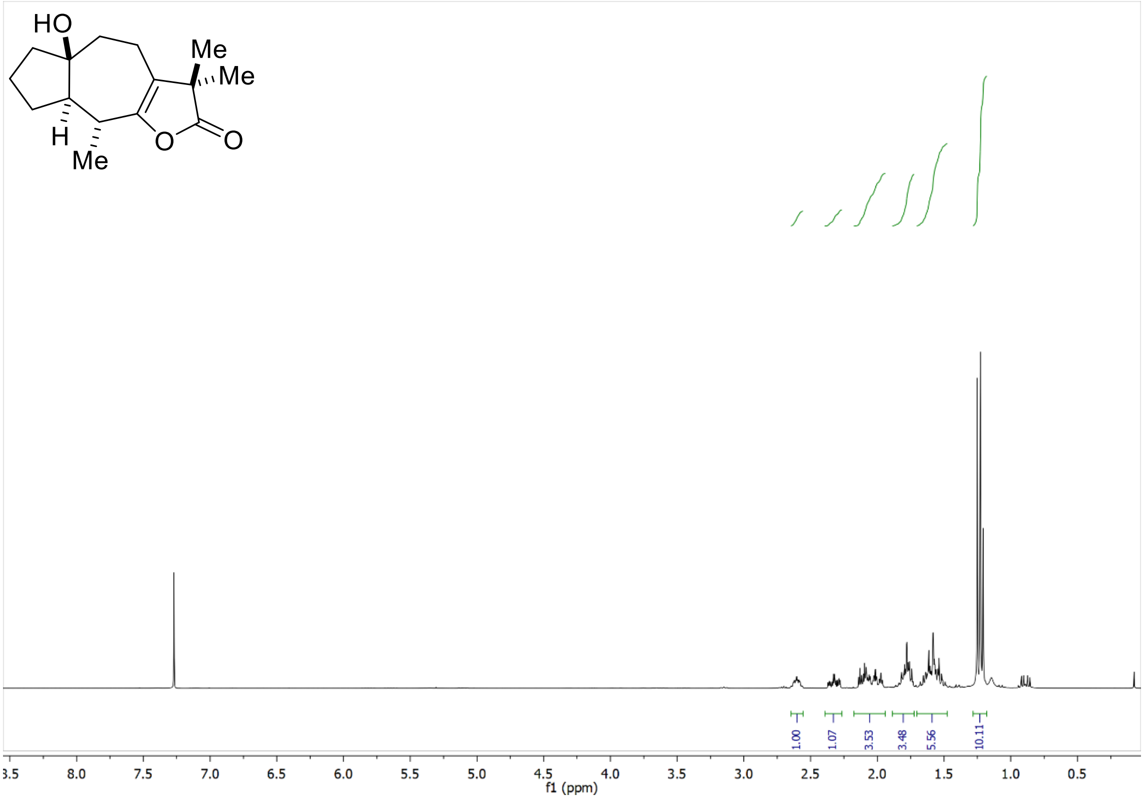

Supplementary Figure 154. ^{13}C NMR (100 MHz, CDCl$_3$) spectrum of **7d**

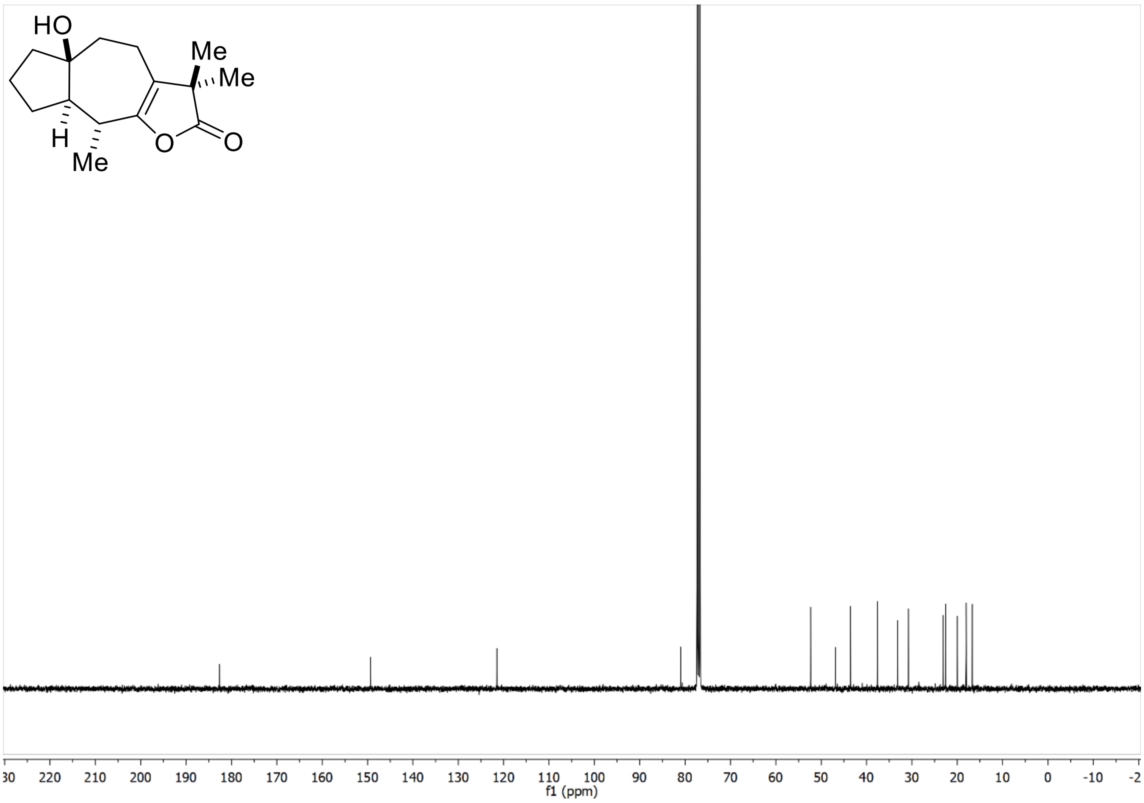

Supplementary Figure 155. ^{1}H NMR (500 MHz, $CDCl_3$) spectrum of **7e**

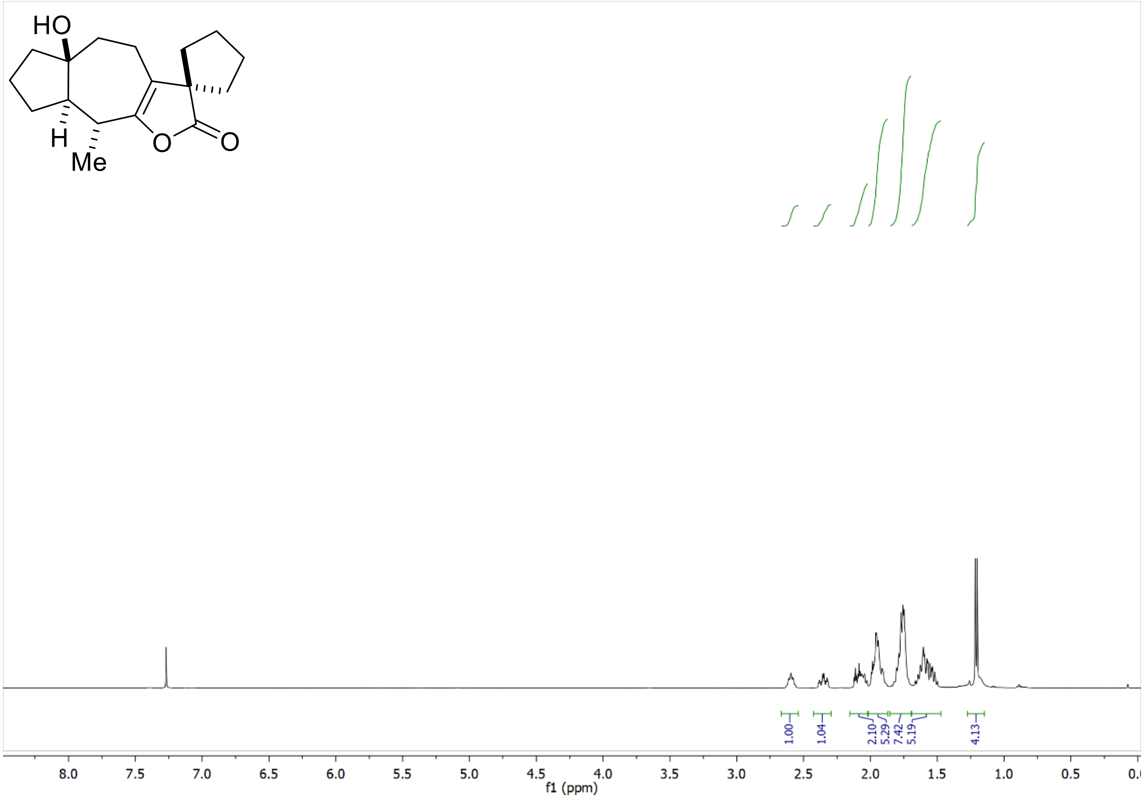

Supplementary Figure 156. ^{13}C NMR (125 MHz, $CDCl_3$) spectrum of **7e**

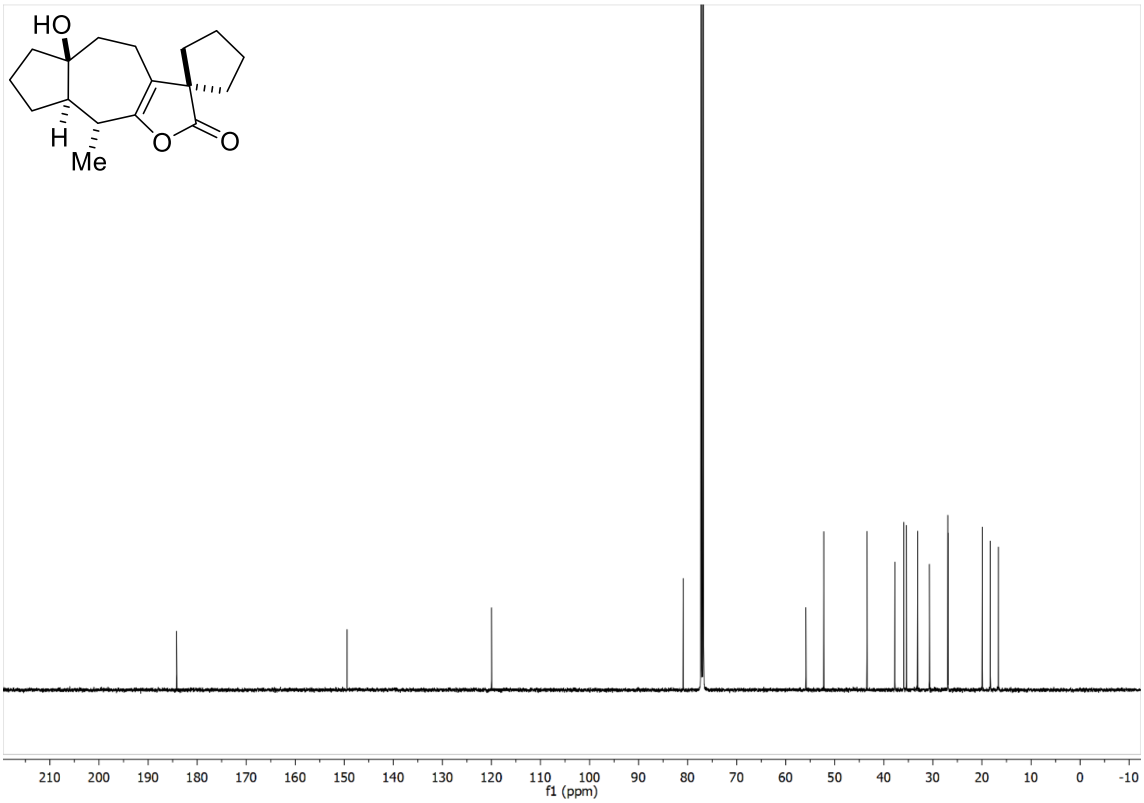

Supplementary Figure 157. ^{1}H NMR (400 MHz, $CDCl_3$) spectrum **7f**

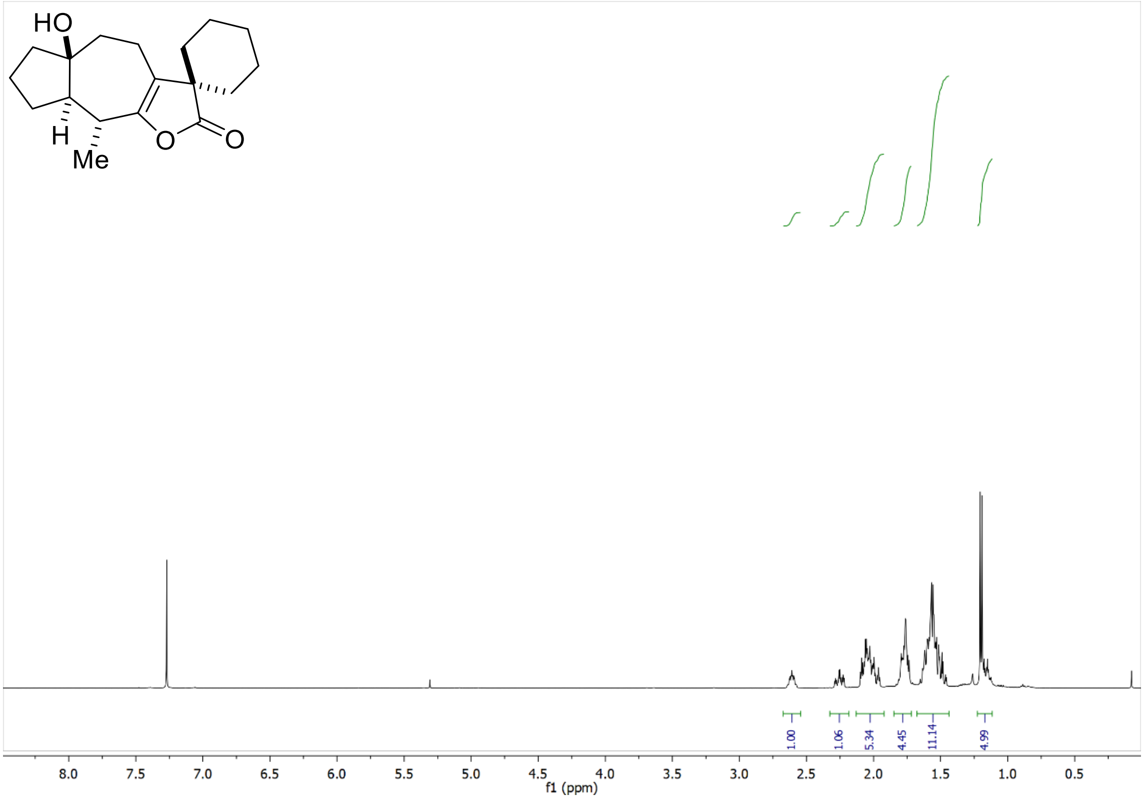

Supplementary Figure 158. ^{13}C NMR (100 MHz, $CDCl_3$) spectrum of **7f**

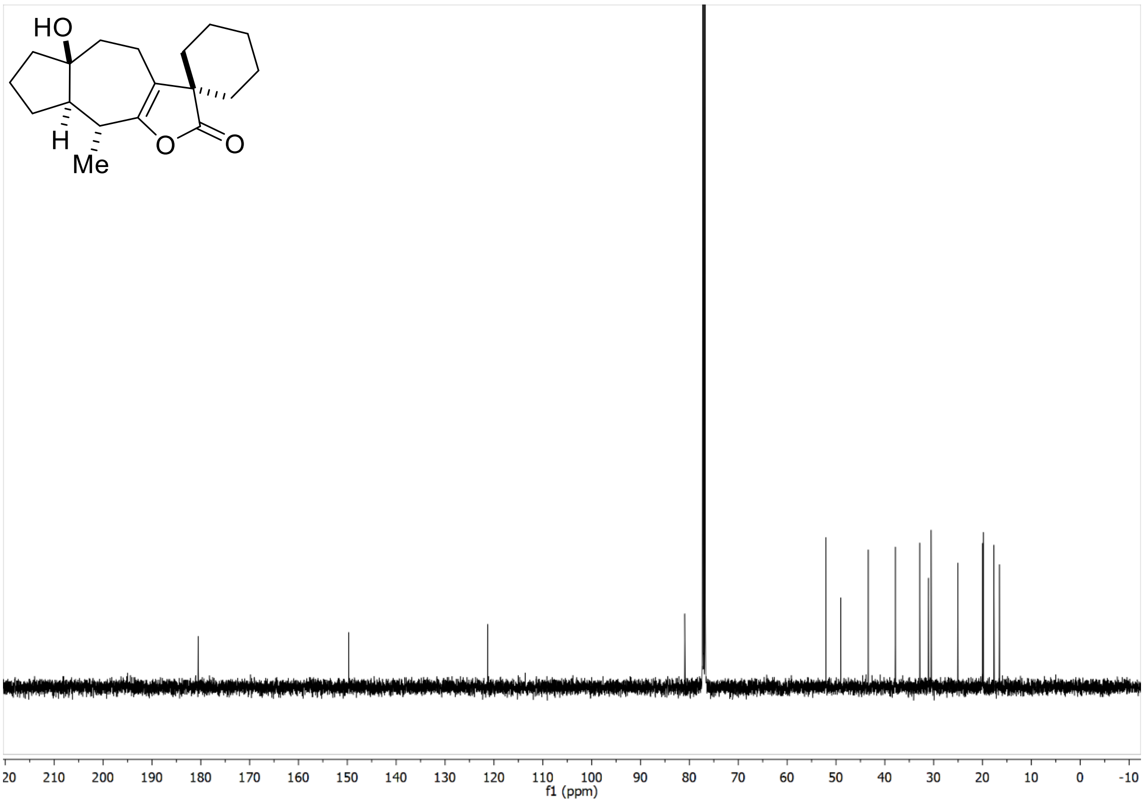

Supplementary Figure 159. ^{1}H NMR (400 MHz, $CDCl_3$) spectrum of **7g**

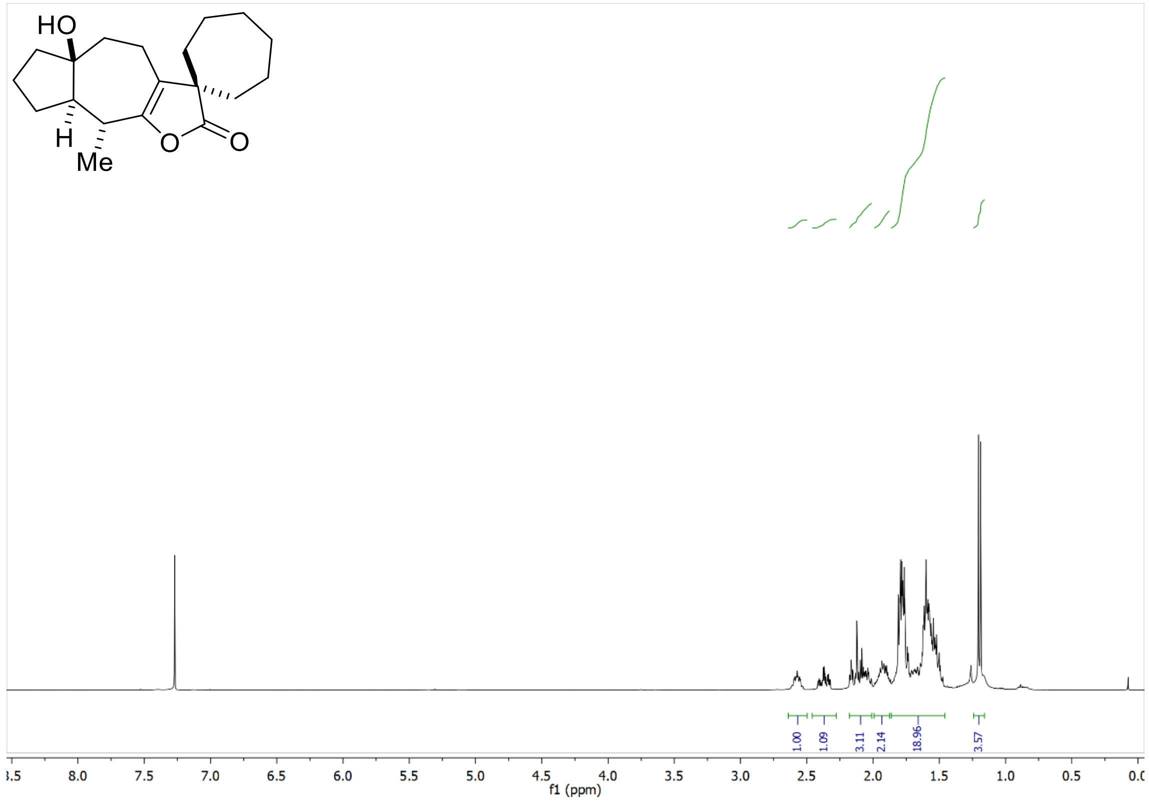

Supplementary Figure 160. ^{13}C NMR (100 MHz, $CDCl_3$) spectrum of **7g**

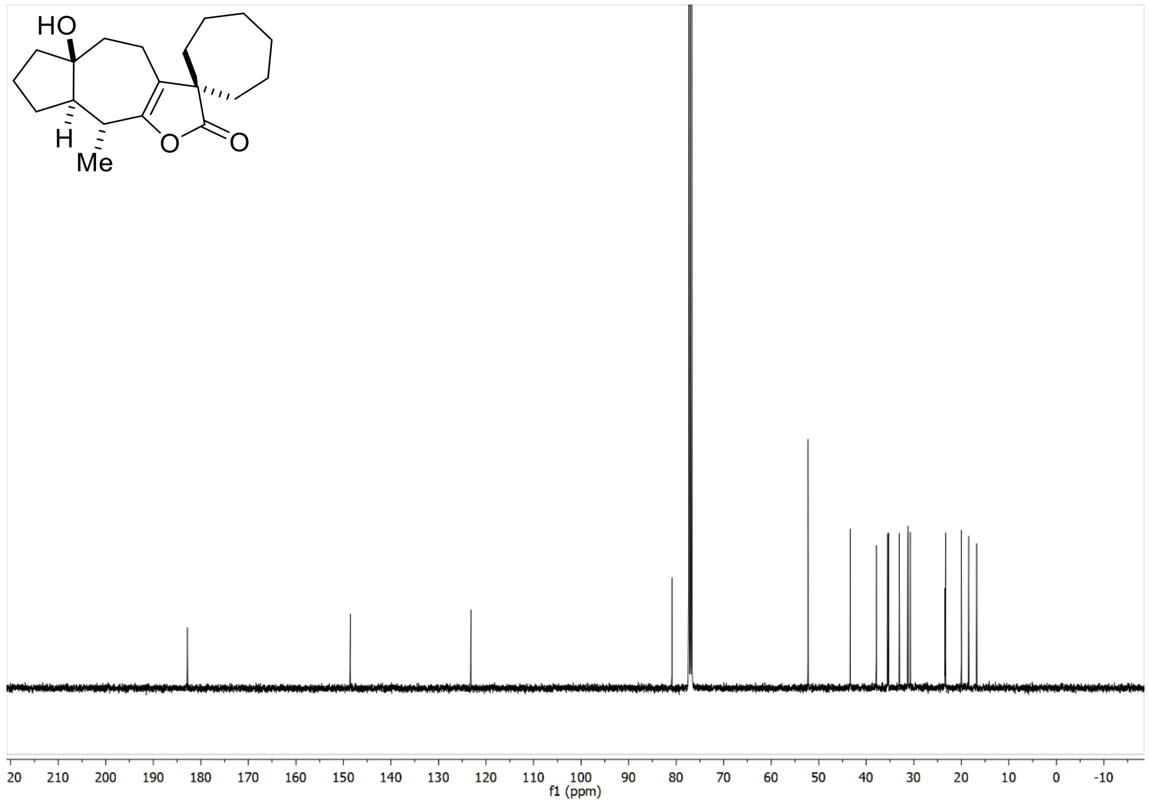

Supplementary Figure 161. ^{1}H NMR (400 MHz, $CDCl_3$) spectrum of **7h**

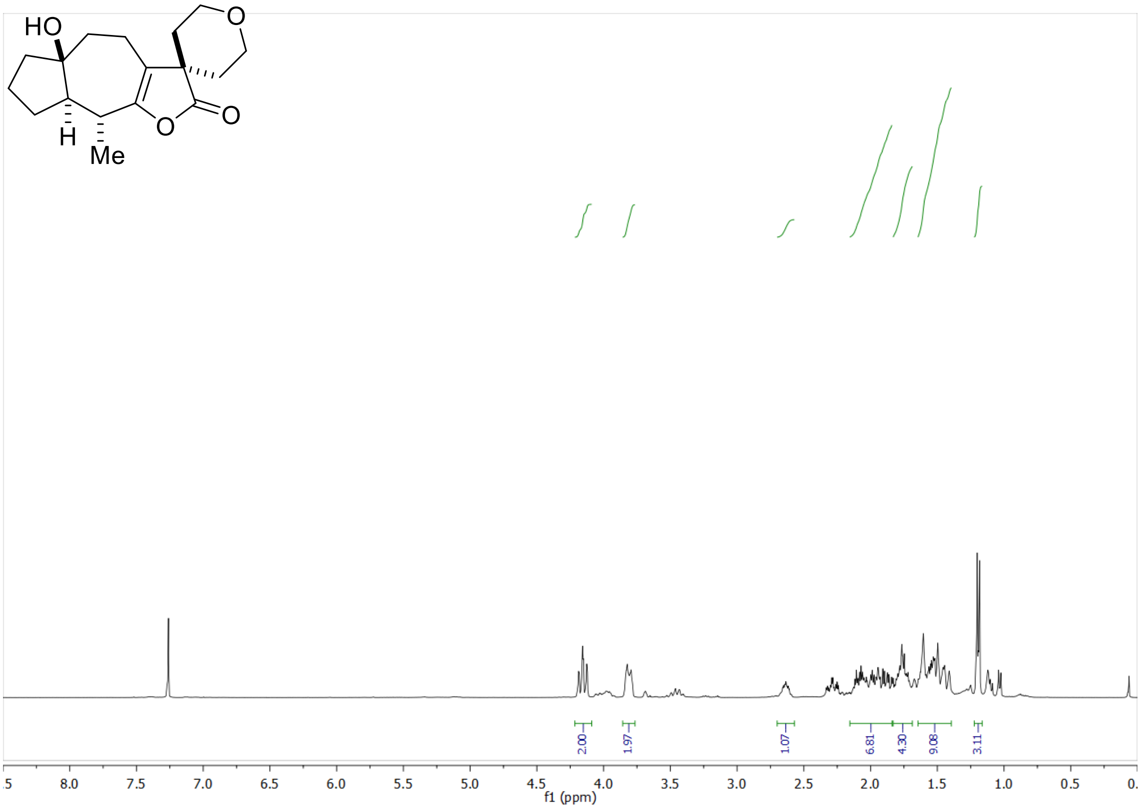

Supplementary Figure 162. ^{13}C NMR (100 MHz, $CDCl_3$) spectrum of **7h**

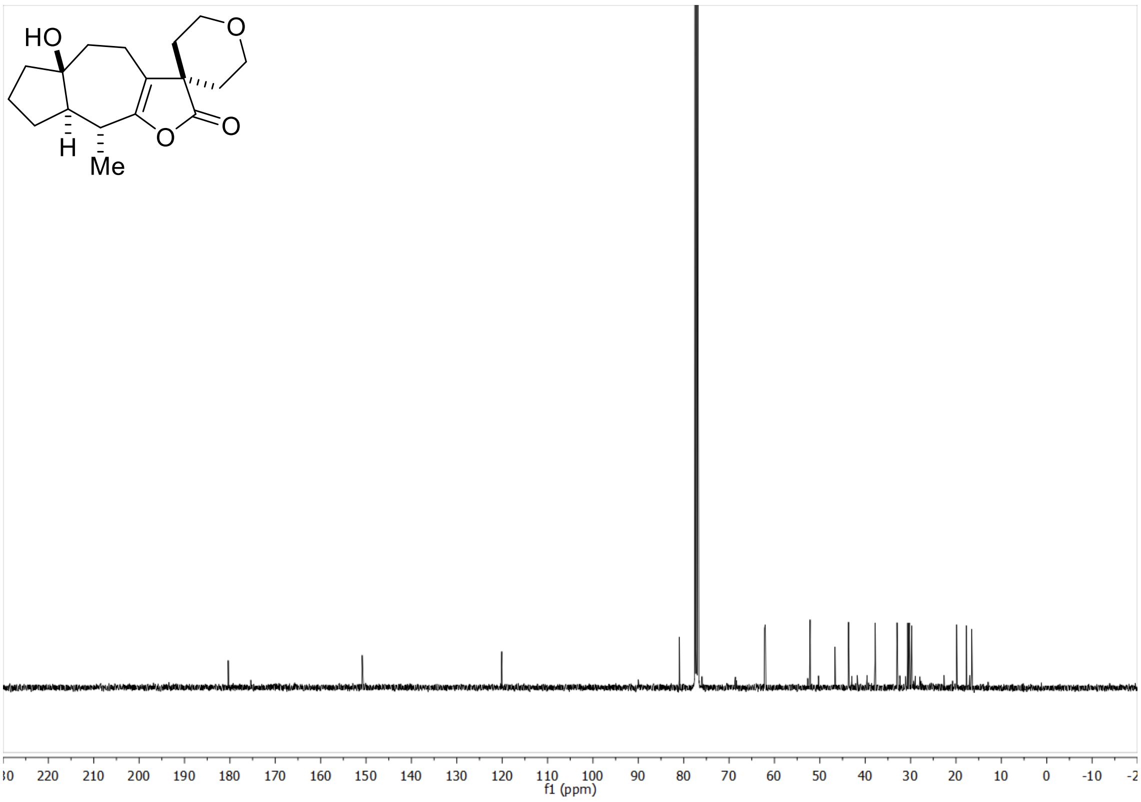

Supplementary Figure 163. ^{1}H NMR (400 MHz, $CDCl_3$) spectrum of **7i**

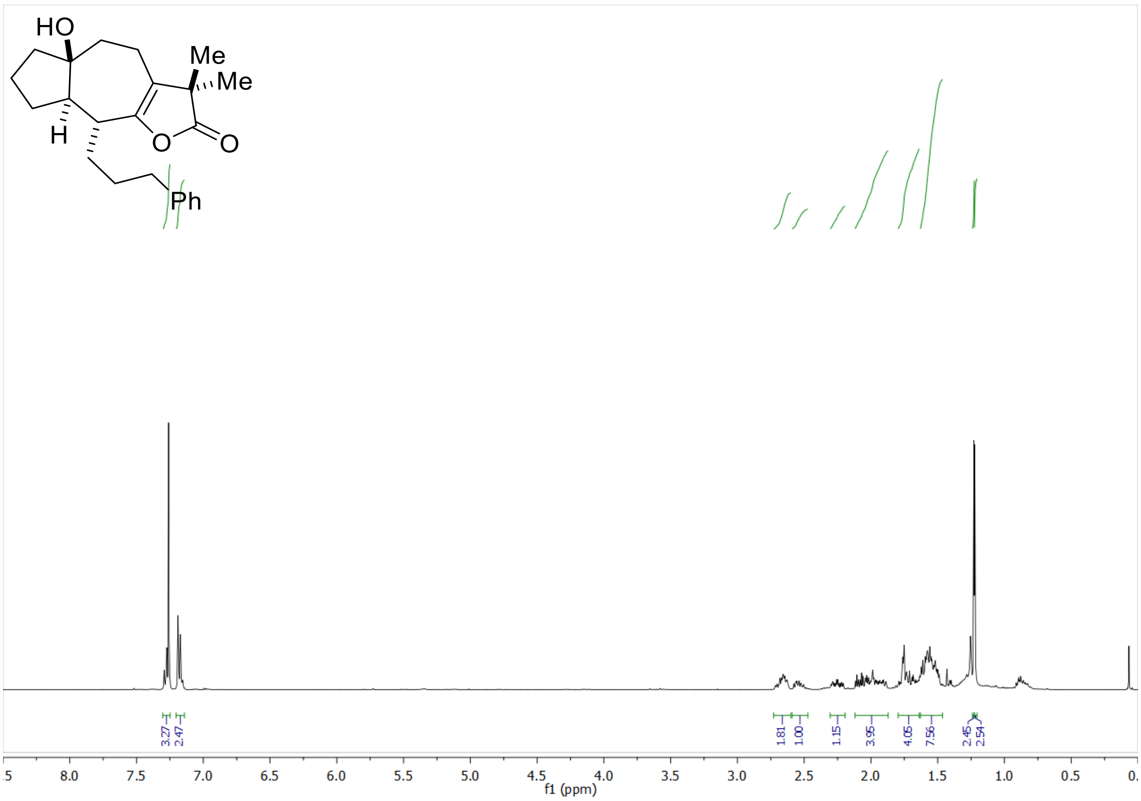

Supplementary Figure 164. ^{13}C NMR (100 MHz, $CDCl_3$) spectrum of **7i**

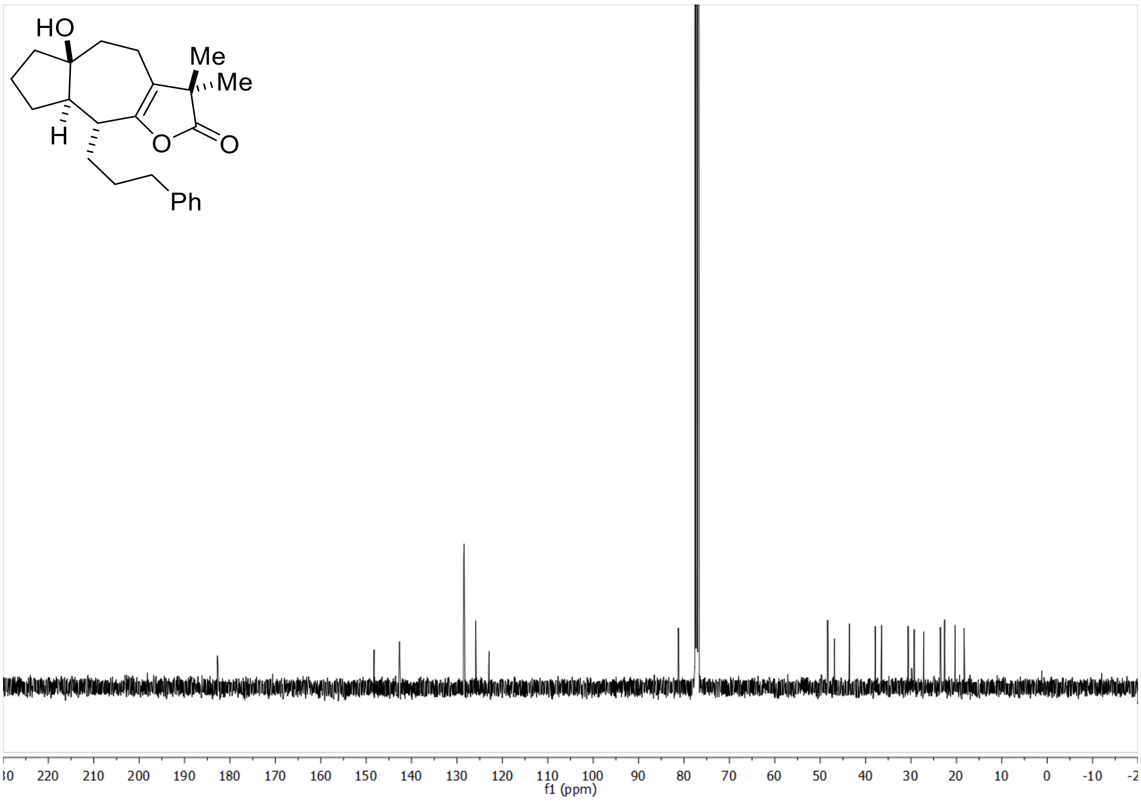

Supplementary Figure 165. ^{1}H NMR (400 MHz, $CDCl_3$) spectrum of **7j**

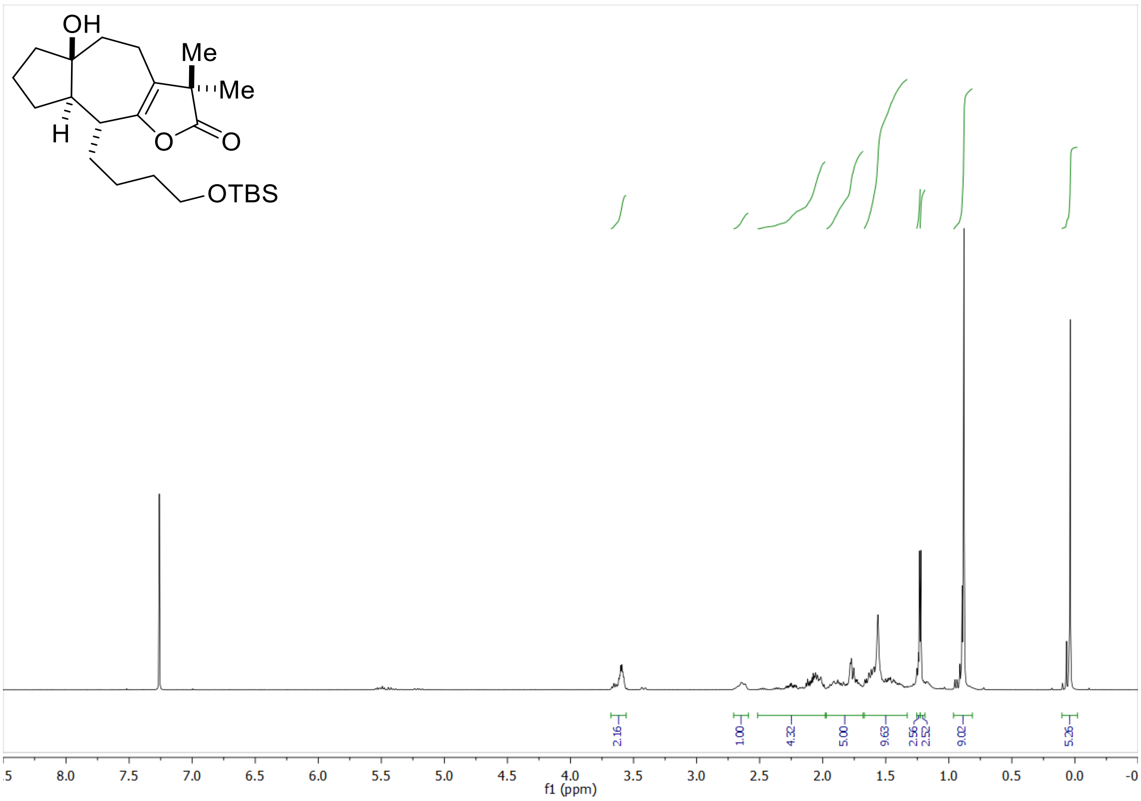

Supplementary Figure 166. ^{13}C NMR (100 MHz, $CDCl_3$) spectrum of **7j**

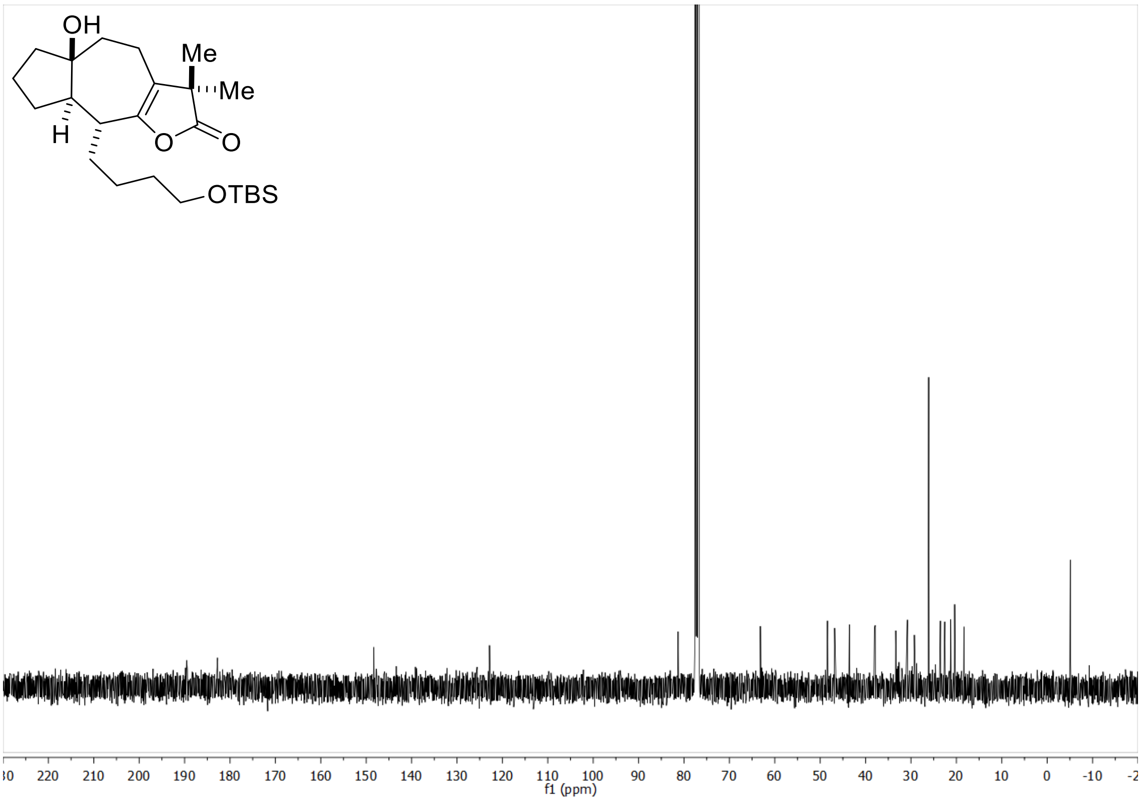

Supplementary Figure 167. ^{1}H NMR (400 MHz, $CDCl_3$) spectrum of **7k**

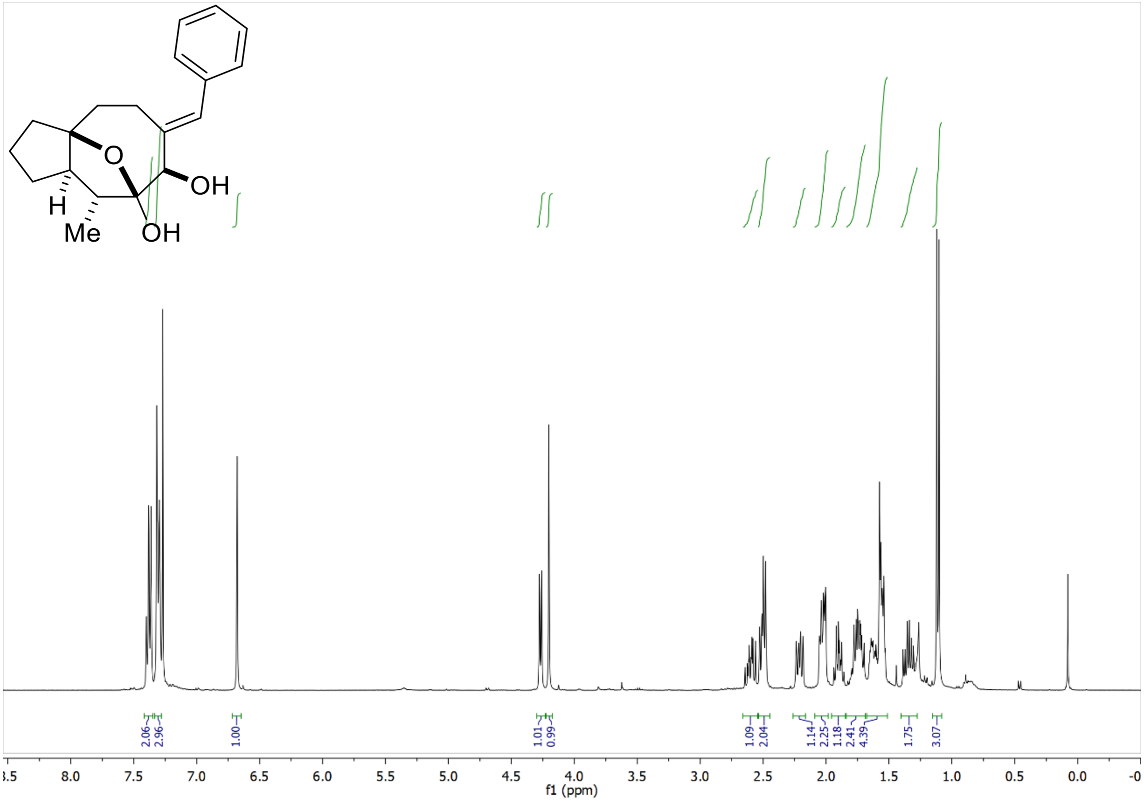

Supplementary Figure 168. ^{13}C NMR (100 MHz, $CDCl_3$) spectrum of **7k**

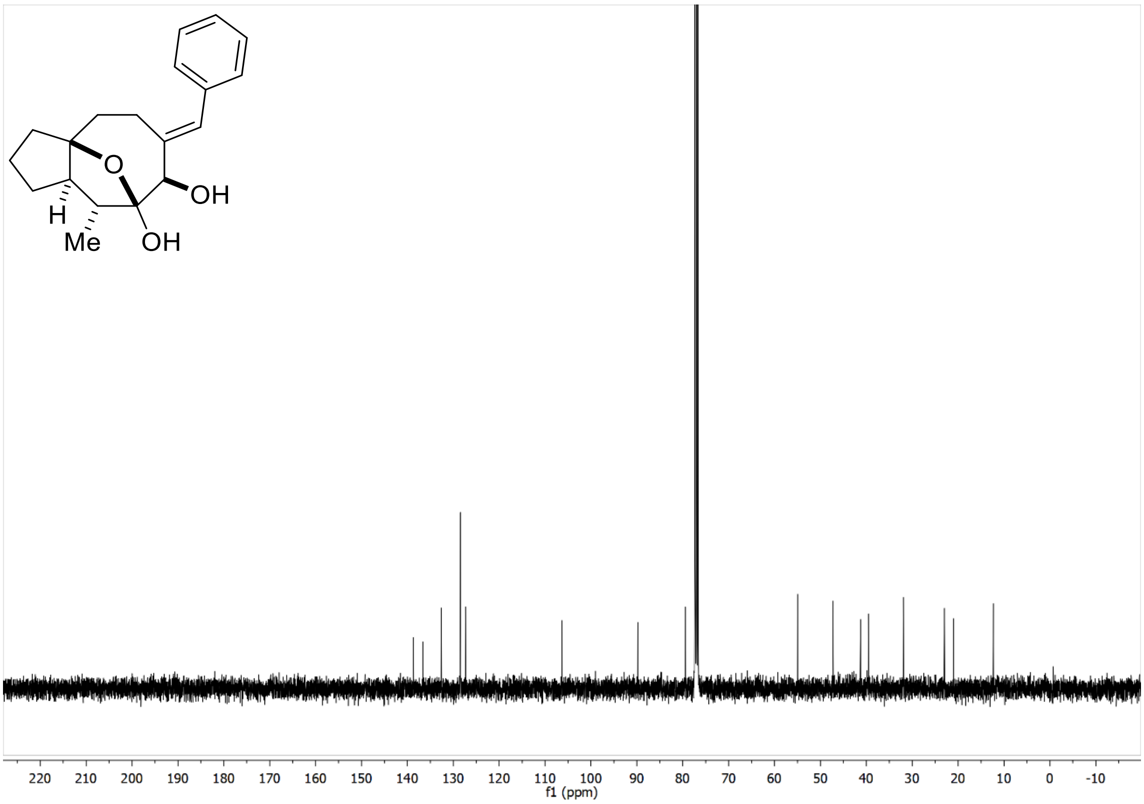

Supplementary Figure 169. ^{1}H NMR (400 MHz, $CDCl_3$) spectrum of **7l**

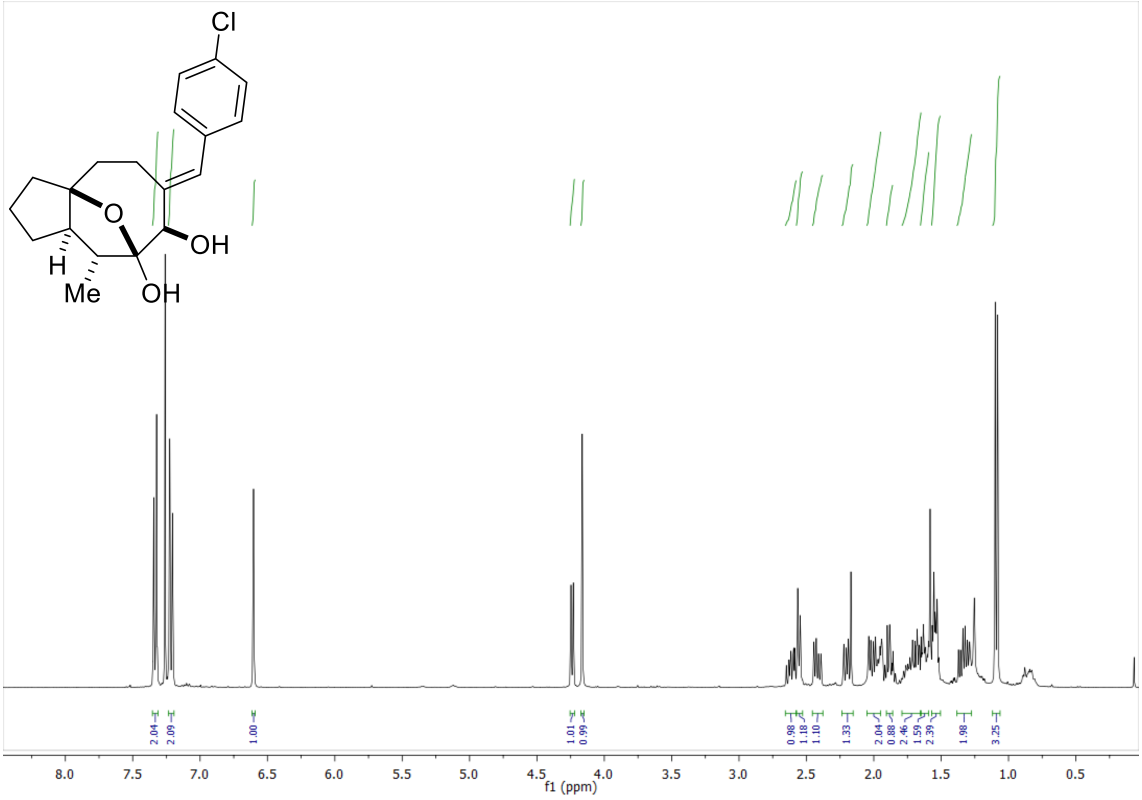

Supplementary Figure 170. ^{13}C NMR (100 MHz, $CDCl_3$) spectrum of **7l**

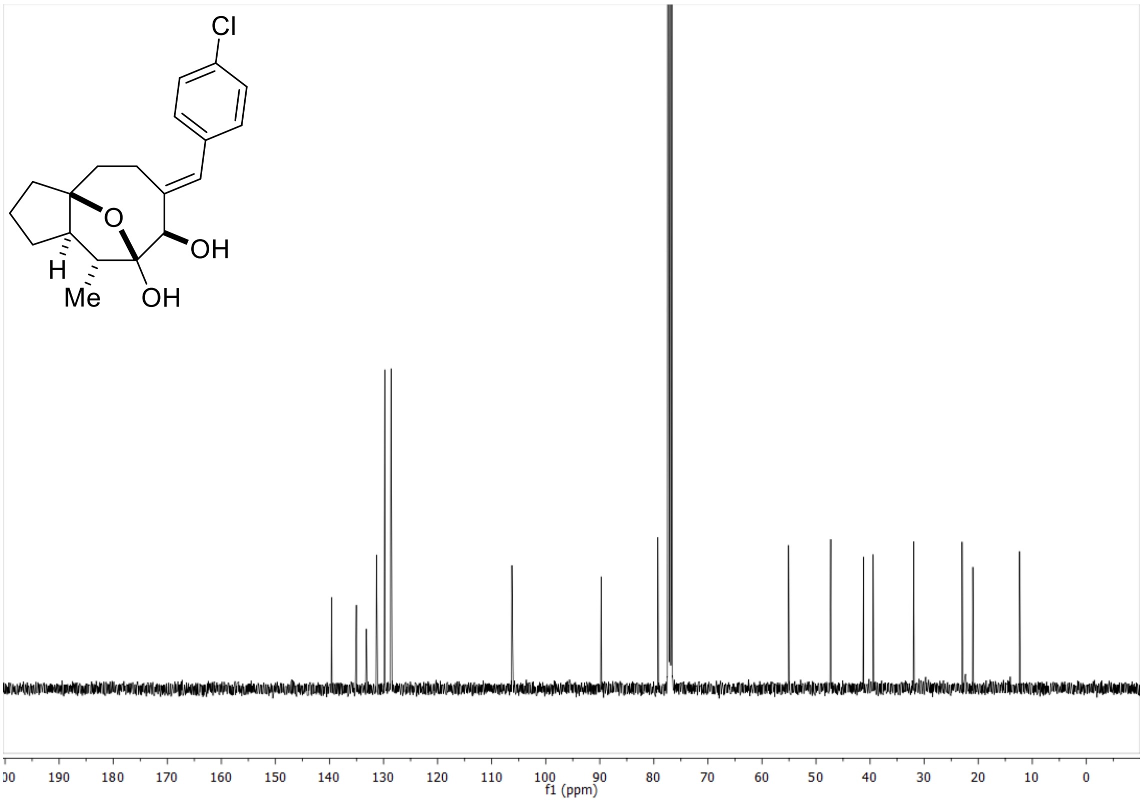

Supplementary Figure 171. ^{1}H NMR (400 MHz, $CDCl_3$) spectrum of **7m**

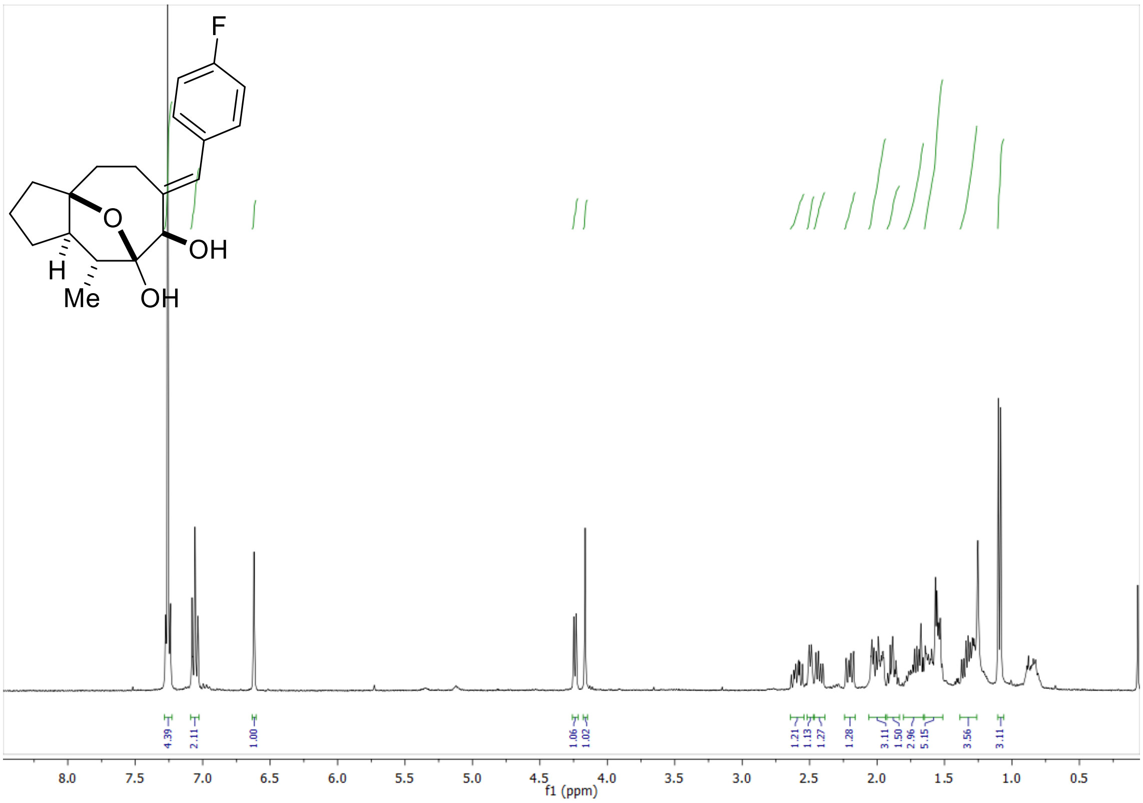

Supplementary Figure 172. ^{13}C NMR (125 MHz, $CDCl_3$) spectrum of **7m**

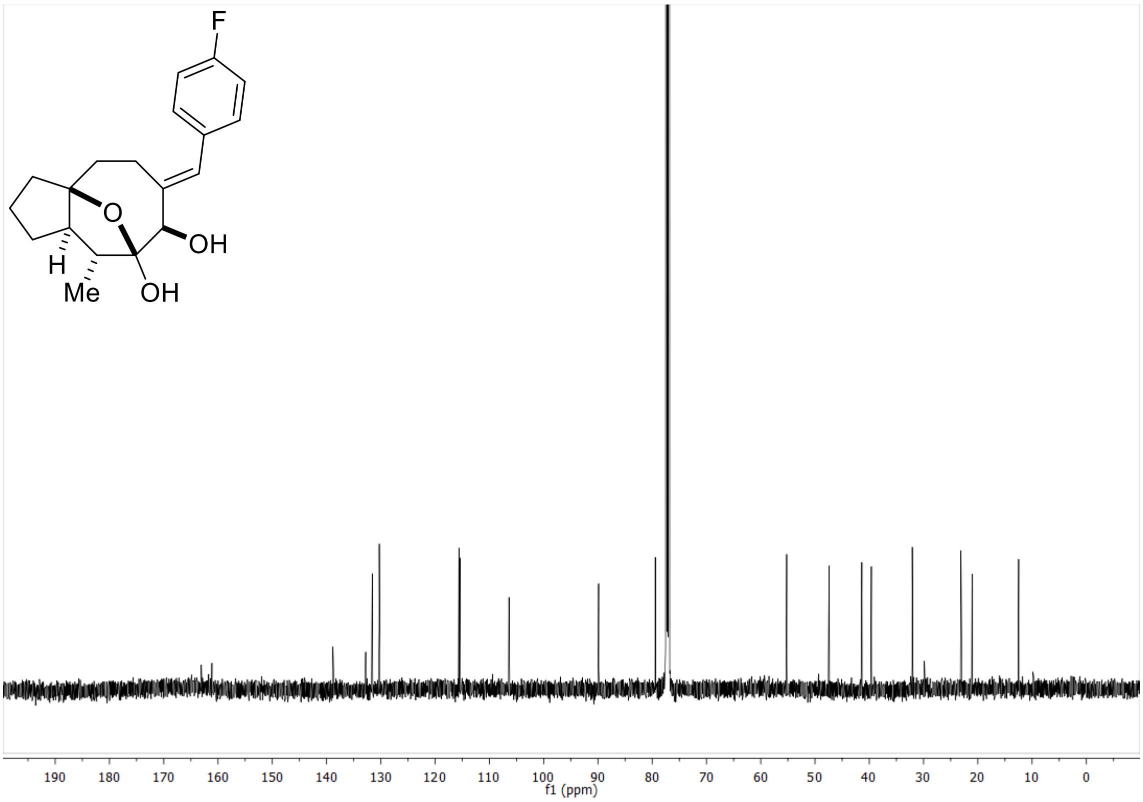

Supplementary Figure 173. ^{1}H NMR (400 MHz, $CDCl_3$) spectrum of **7n**

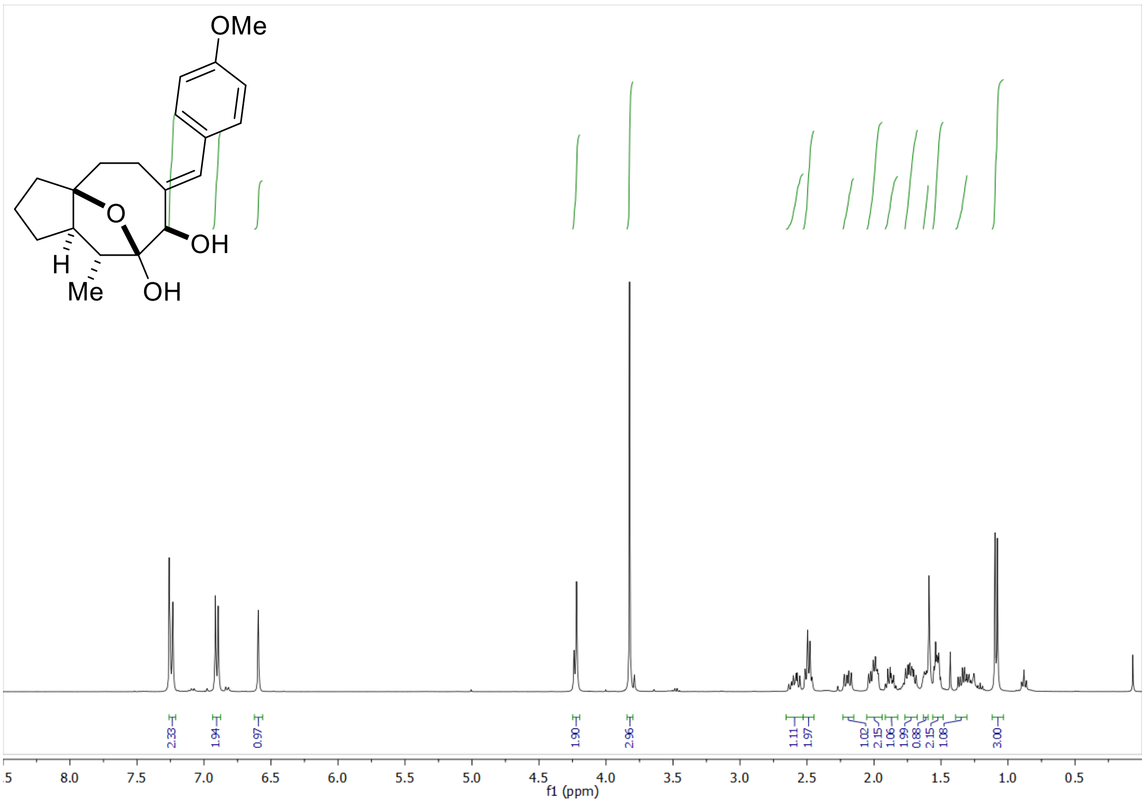

Supplementary Figure 174. ^{13}C NMR (100 MHz, $CDCl_3$) spectrum of **7n**

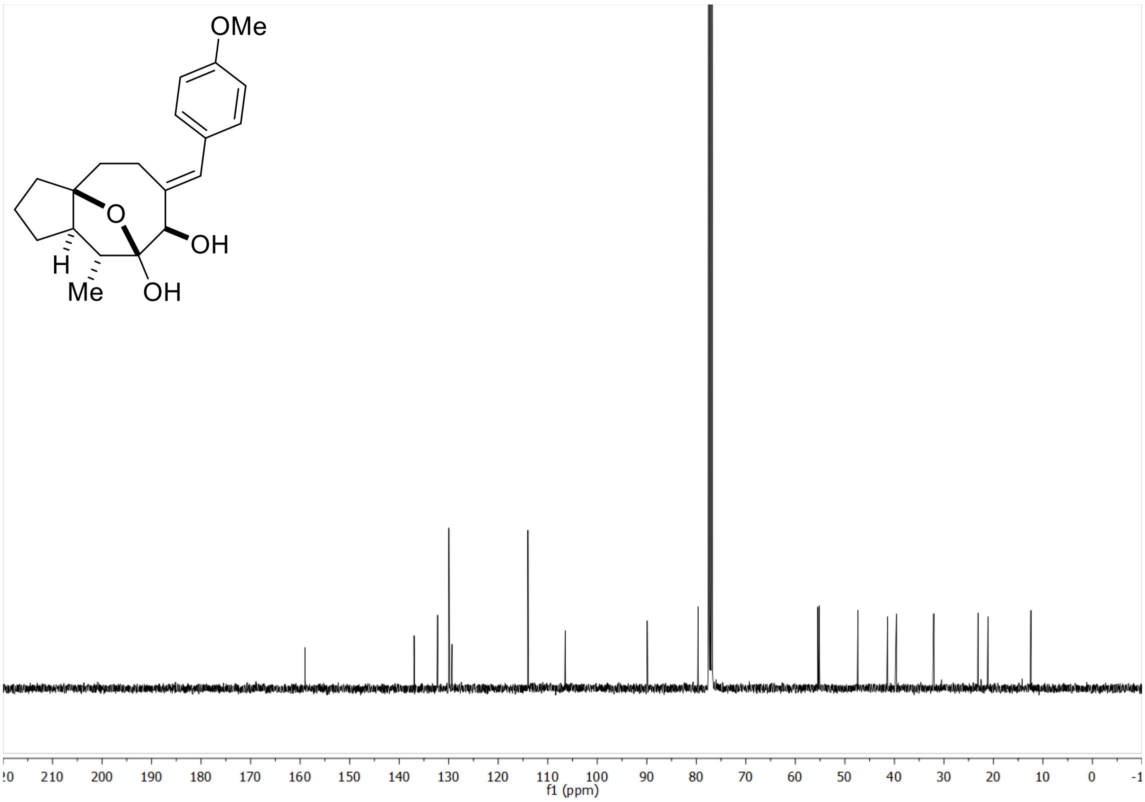

Supplementary Figure 175. ^{1}H NMR (400 MHz, $CDCl_3$) spectrum of **7o**

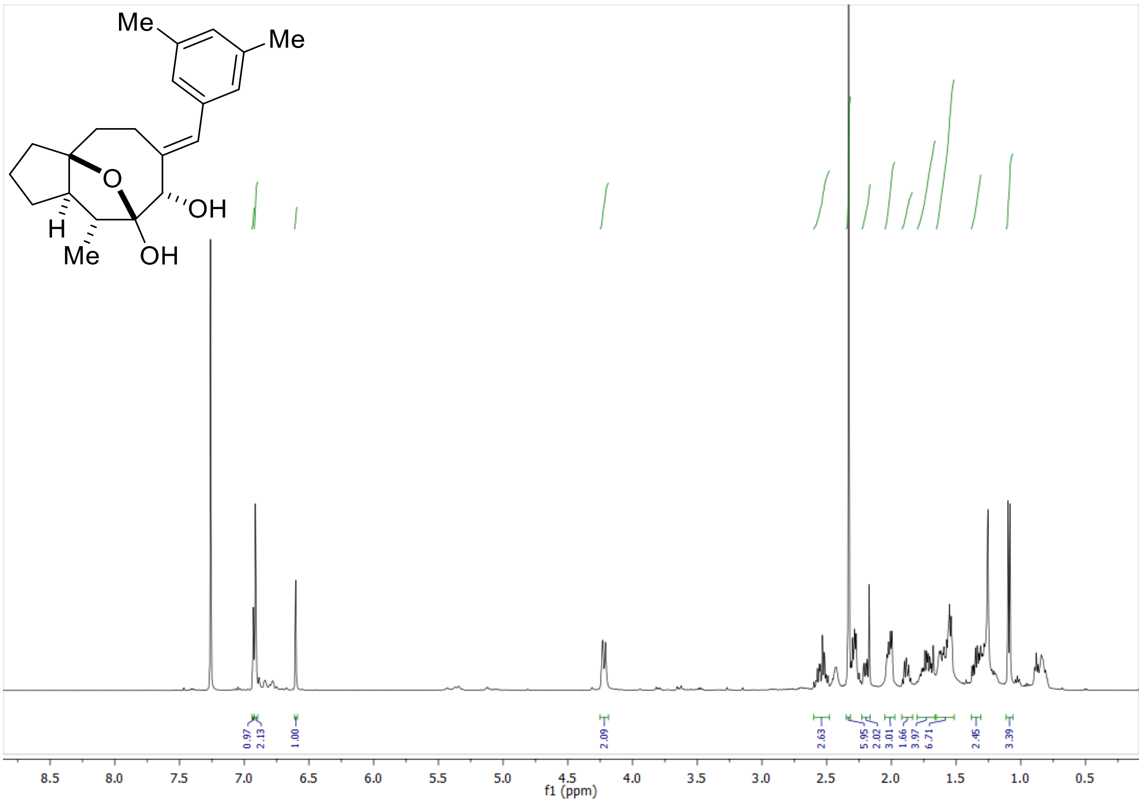

Supplementary Figure 176. ^{13}C NMR (100 MHz, $CDCl_3$) spectrum of **7o**

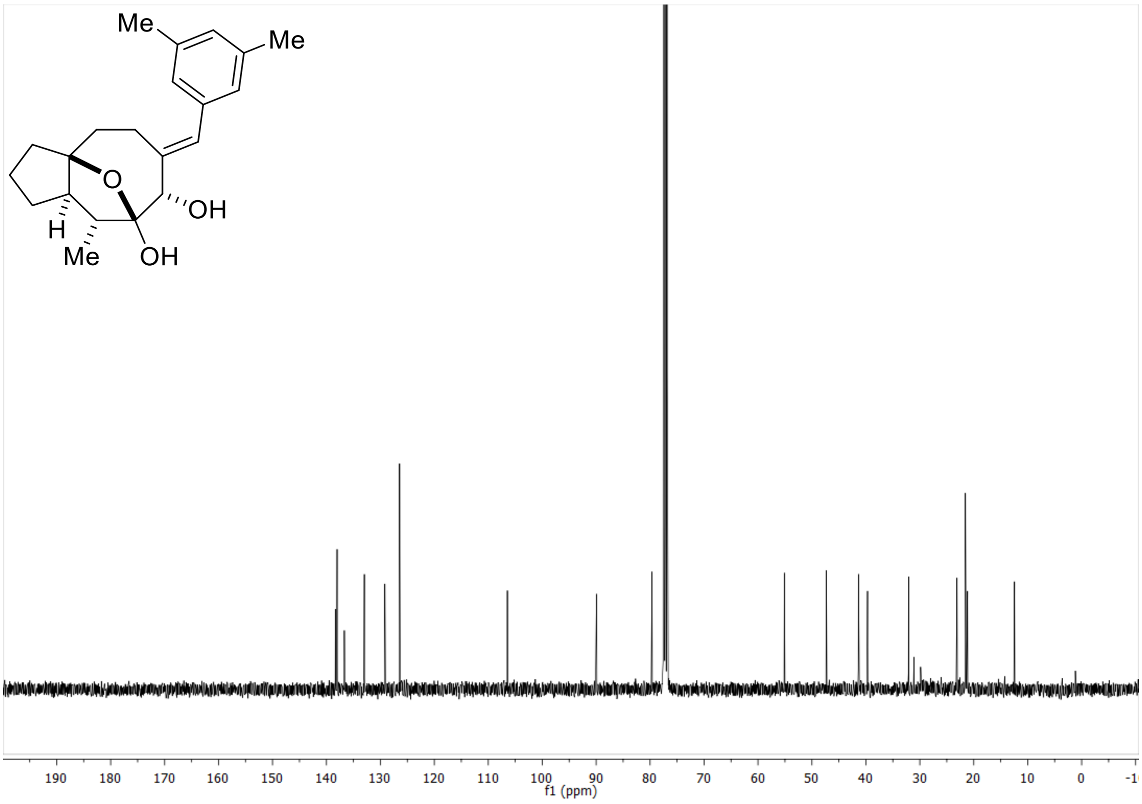

Supplementary Figure 177. ^{1}H NMR (400 MHz, $CDCl_3$) spectrum of **7p**

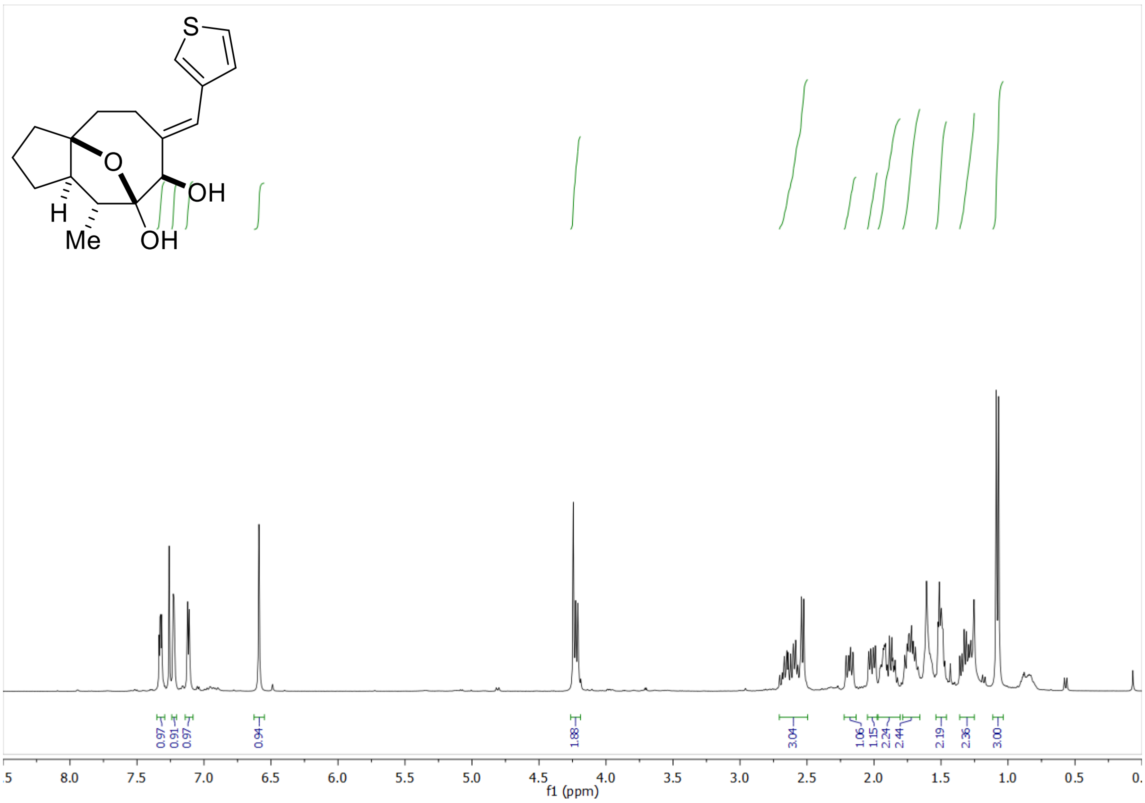

Supplementary Figure 178. ^{13}C NMR (100 MHz, $CDCl_3$) spectrum of **7p**

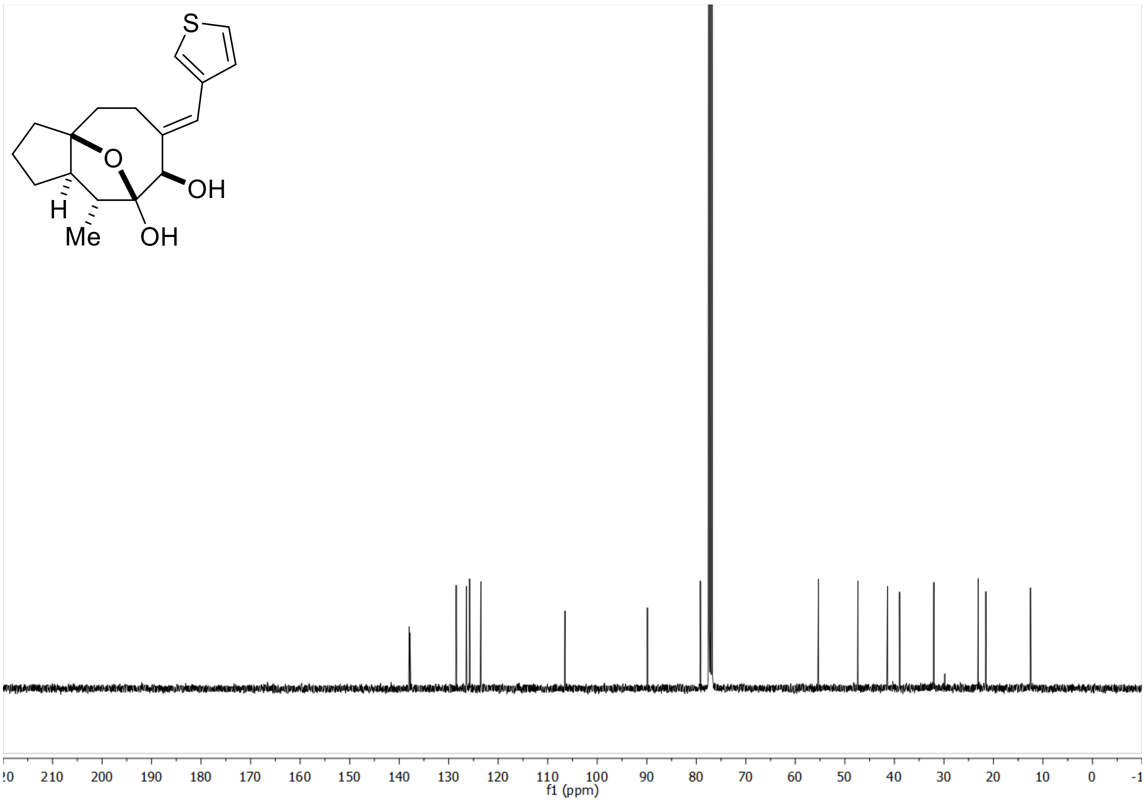

Supplementary Figure 179. ^{1}H NMR (500 MHz, $CDCl_3$) spectrum of **7q**

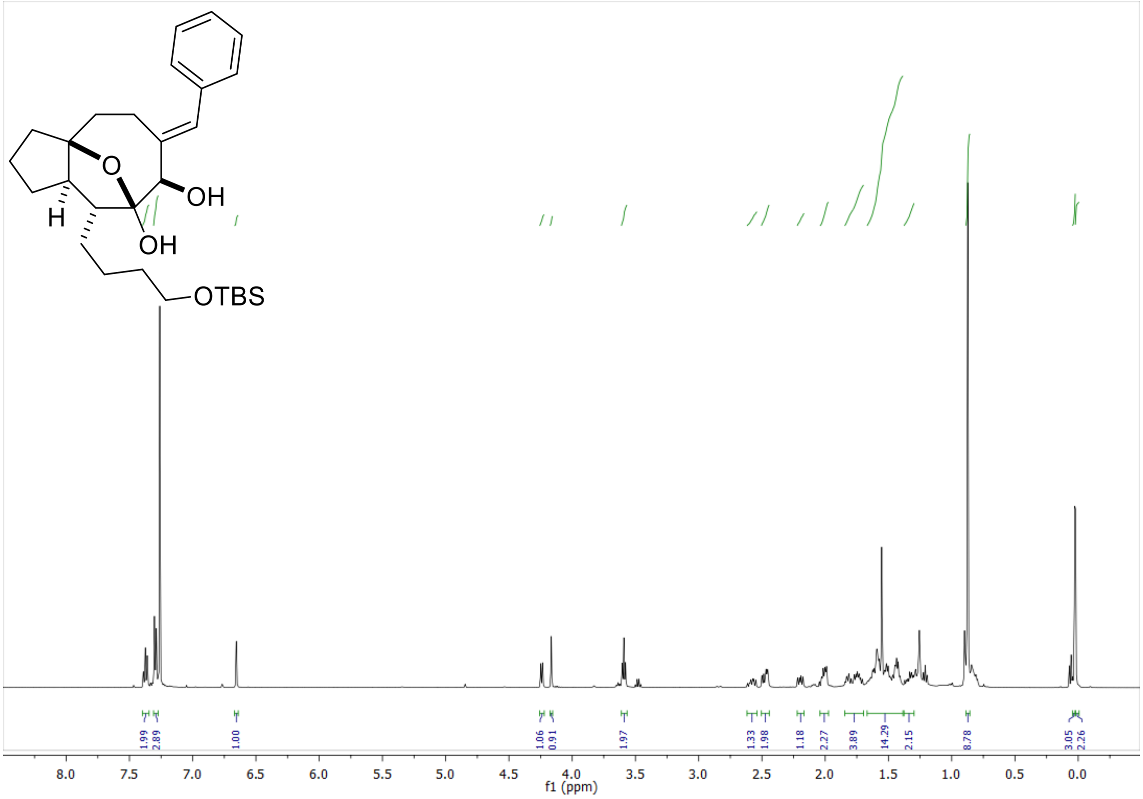

Supplementary Figure 180. ^{13}C NMR (125 MHz, $CDCl_3$) spectrum of **7q**

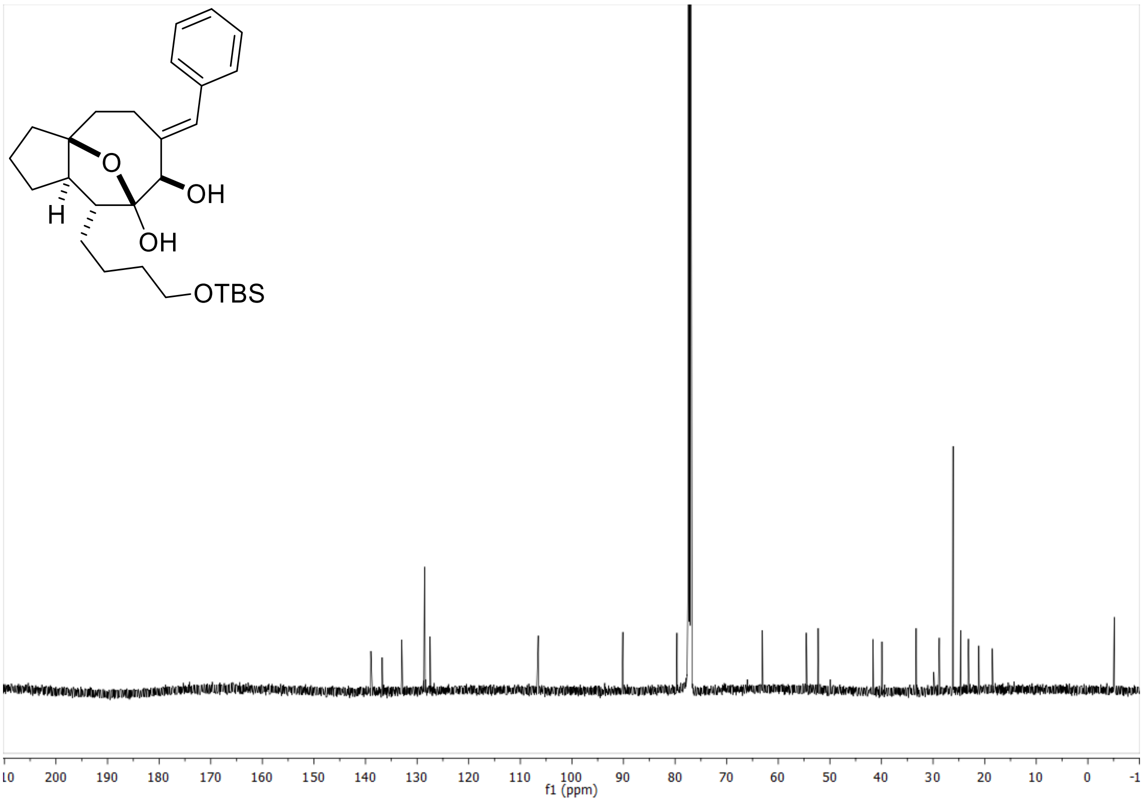

Supplementary Figure 181. ^{1}H NMR (400 MHz, $CDCl_3$) spectrum of **D_2-7a**

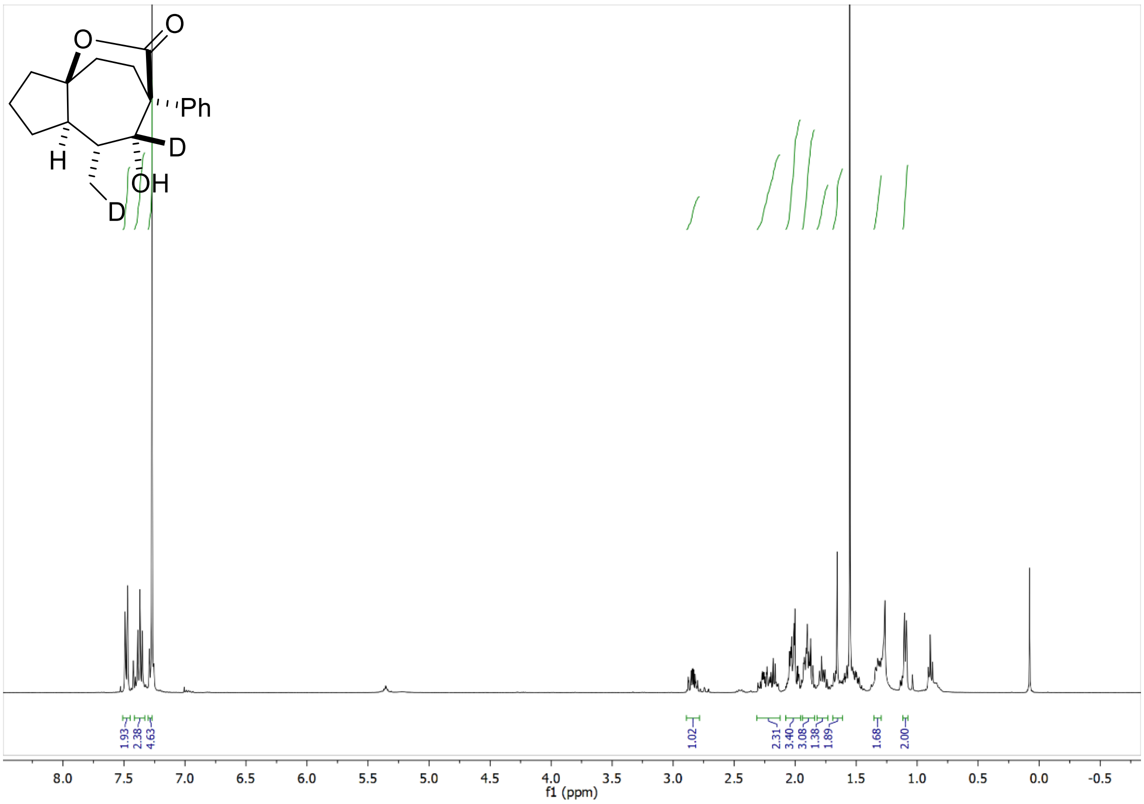

Supplementary Figure 182. ^{13}C NMR (100 MHz, $CDCl_3$) spectrum of **D_2-7a**

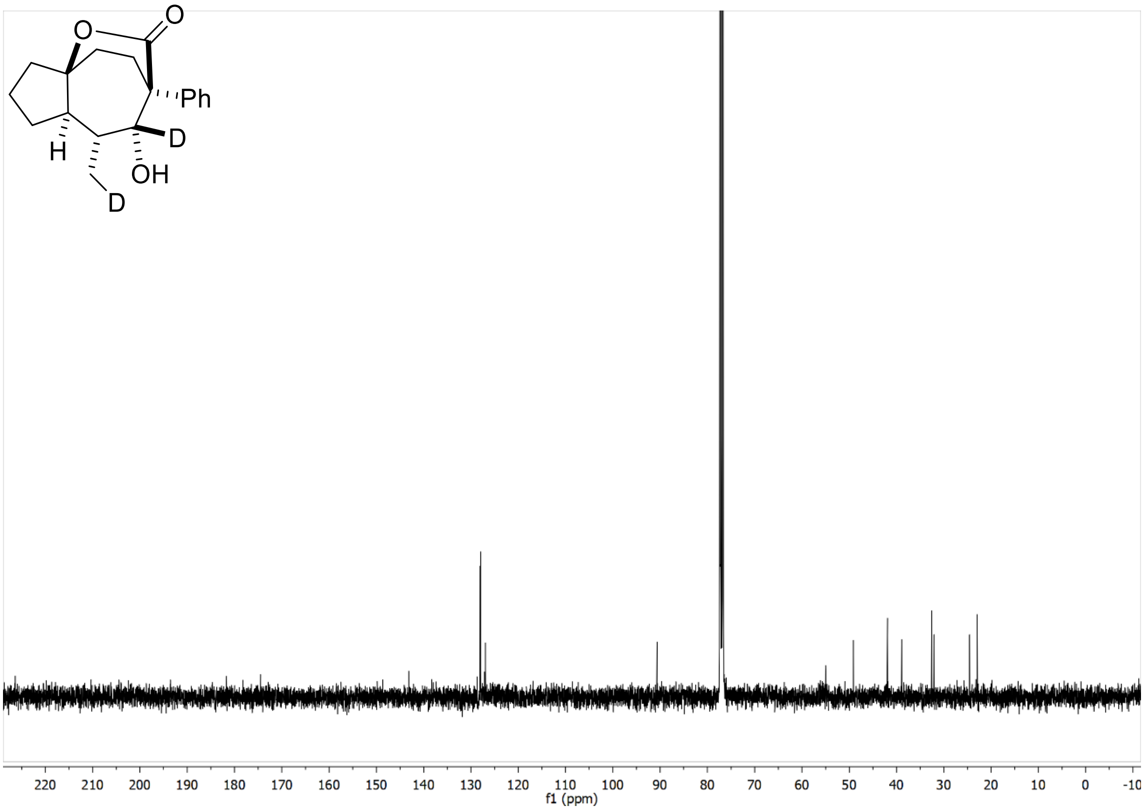

Supplementary Figure 183. ^{1}H NMR (400 MHz, $CDCl_3$) spectrum of **7p**

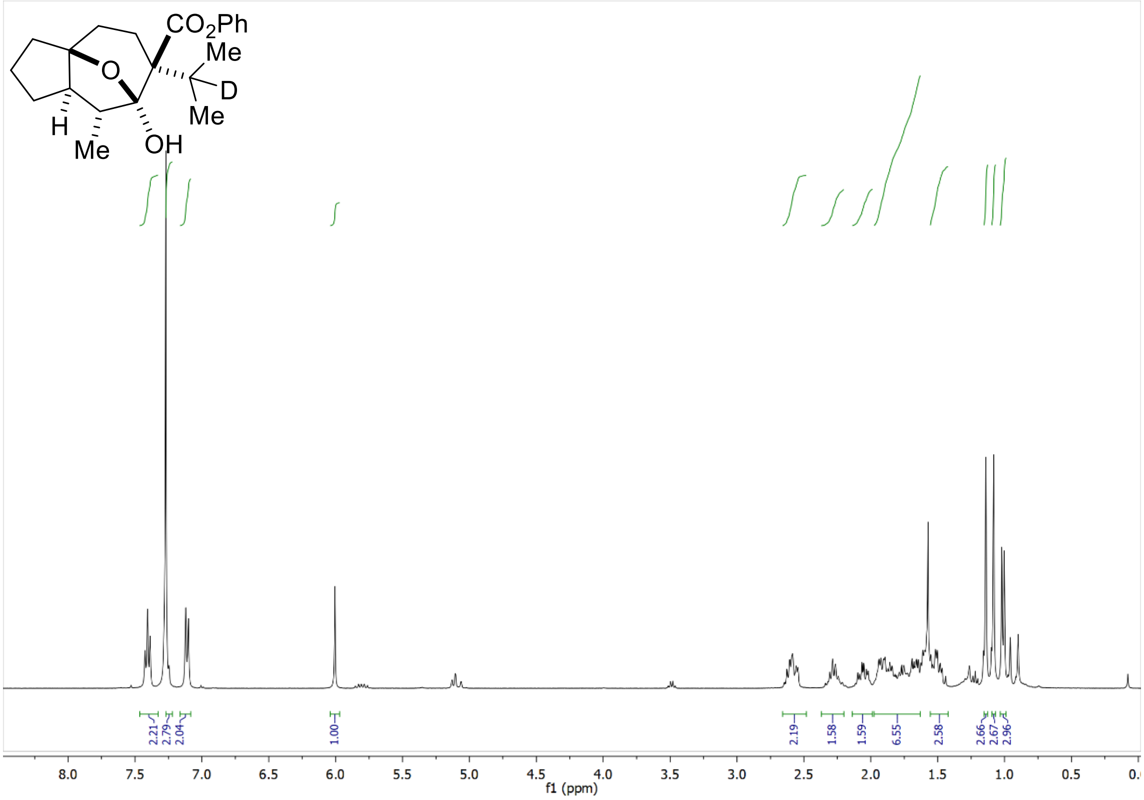

Supplementary Figure 184. ^{13}C NMR (100 MHz, $CDCl_3$) spectrum of **7p**

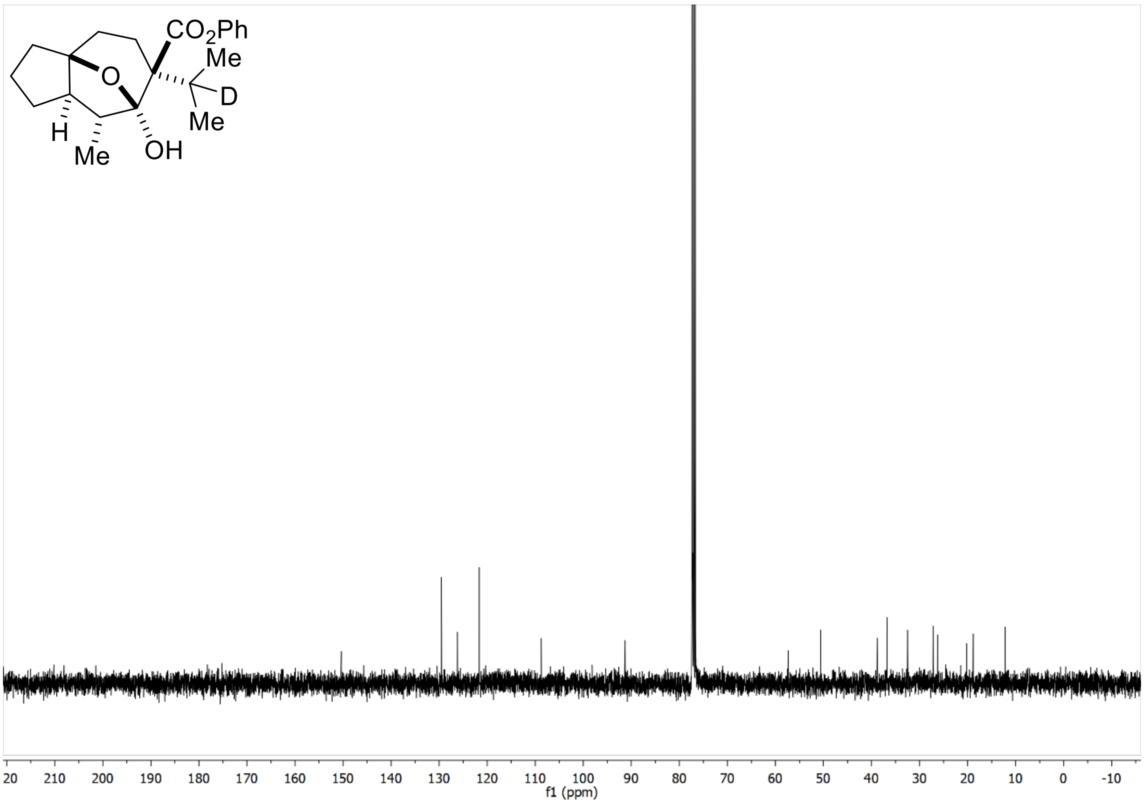

Supplementary Figure 185. ^{1}H NMR (400 MHz, $CDCl_3$) spectrum of **7q**

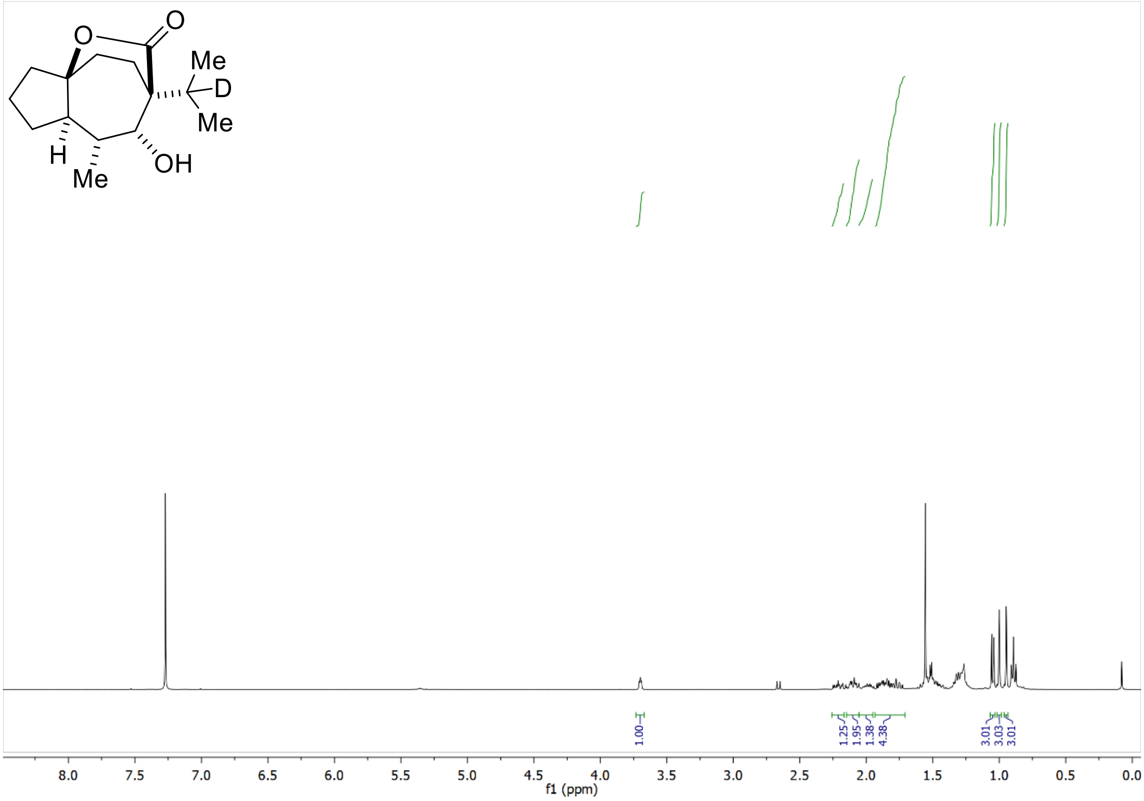

Supplementary Figure 186. ^{13}C NMR (100 MHz, $CDCl_3$) spectrum of **7q**

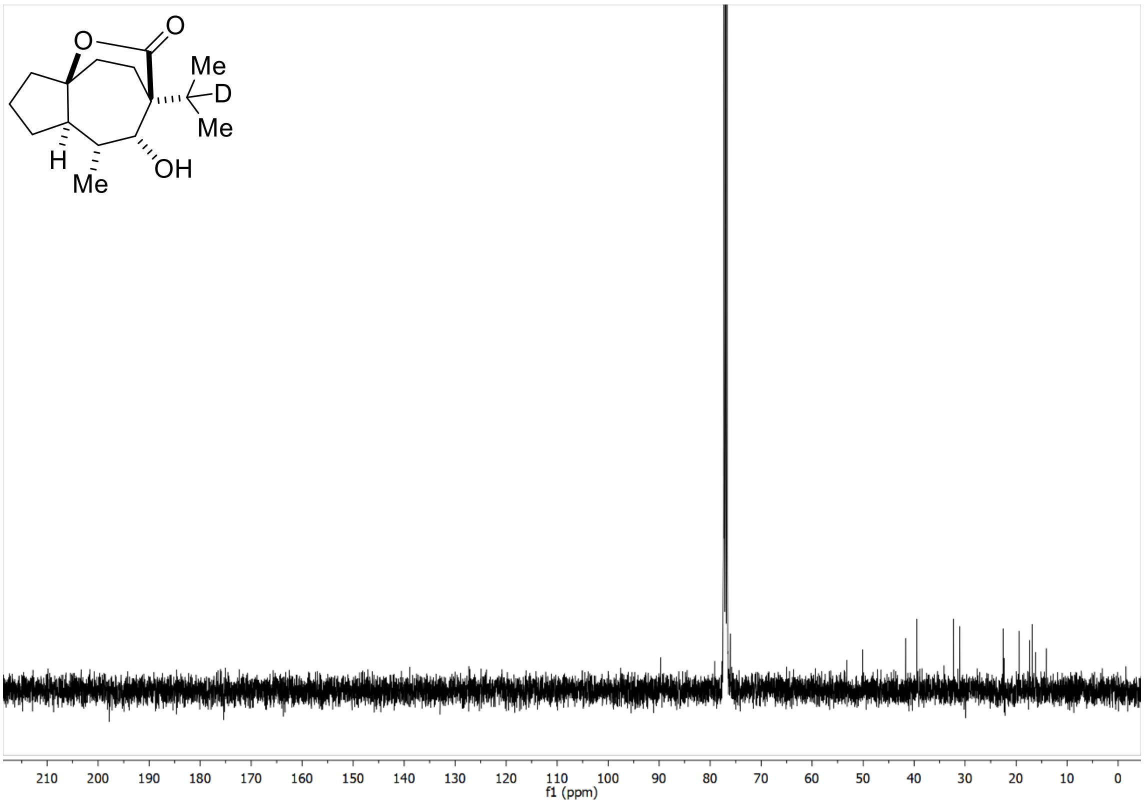

Supplementary Table 1. Crystal data and structure refinement for **7a'**.

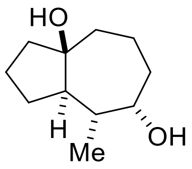

7a'

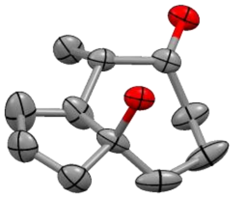

X-Ray of **7a'**

CCDC number: 1825586

Identification code	**7a'**
Empirical formula	$C_{11}H_{20}O_2$
Formula weight	184.28
Temperature/K	150.0
Crystal system	monoclinic
Space group	C2/c
a/Å	19.3670(14)
b/Å	8.1742(5)
c/Å	13.0587(7)
α/°	90
β/°	95.200(6)
γ/°	90
Volume/Å^3	2058.8(2)
Z	8
ρ_{calc}g/cm^3	1.060
μ/mm^{-1}	0.074
F(000)	657.0
Crystal size/mm^3	0.373 × 0.268 × 0.196
Radiation	MoKα (λ = 0.71073)
2Θ range for data collection/°	5.412 to 50.66
Index ranges	$-23 \leq h \leq 22$, $-9 \leq k \leq 9$, $-15 \leq l \leq 15$
Reflections collected	8467
Independent reflections	1882 [R_{int} = 0.0420, R_{sigma} = 0.0363]
Data/restraints/parameters	1882/0/151
Goodness-of-fit on F^2	1.092
Final R indexes [I>=2σ (I)]	R_1 = 0.0668, wR_2 = 0.1559
Final R indexes [all data]	R_1 = 0.0821, wR_2 = 0.1654
Largest diff. peak/hole / e Å^{-3}	0.28/-0.26

Supplementary Table 2. Crystal data and structure refinement for **7d'**.

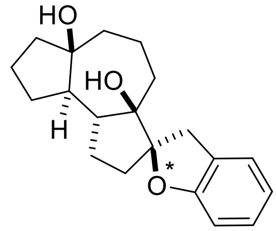

7d'

CCDC number: 1825585

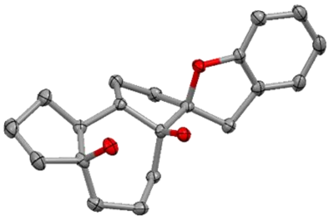

X-Ray of **7d'**

Identification code	**7d'**
Empirical formula	$C_{20}H_{26}O_3$
Formula weight	314.41
Temperature/K	149.9(4)
Crystal system	triclinic
Space group	P-1
a/Å	9.1071(5)
b/Å	9.3085(5)
c/Å	10.4389(5)
α/°	69.348(5)
β/°	82.814(4)
γ/°	76.575(5)
Volume/Å^3	804.54(8)
Z	2
ρ_{calc}g/cm^3	1.298
μ/mm^{-1}	0.085
F(000)	340.0
Crystal size/mm^3	0.536 × 0.278 × 0.118
Radiation	MoKα (λ = 0.71073)
2Θ range for data collection/°	7.324 to 50.694
Index ranges	$-10 \le h \le 10$, $-11 \le k \le 11$, $-12 \le l \le 12$
Reflections collected	23445
Independent reflections	2945 [R_{int} = 0.0546, R_{sigma} = 0.0308]
Data/restraints/parameters	2945/0/210
Goodness-of-fit on F^2	1.035
Final R indexes [I>=2σ (I)]	R_1 = 0.0404, wR_2 = 0.0940
Final R indexes [all data]	R_1 = 0.0486, wR_2 = 0.0991
Largest diff. peak/hole / e Å^{-3}	0.29/-0.19

Supplementary Table 3. Crystal data and structure refinement for **7a**.

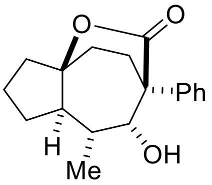

7a

CCDC number: 1825588

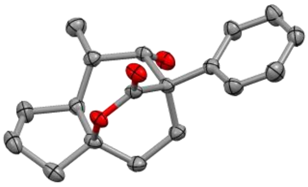

X-Ray of **7a**

Identification code	**7a**
Empirical formula	$C_{18}H_{22}O_3$
Formula weight	286.35
Temperature/K	293(2)
Crystal system	monoclinic
Space group	$P2_1/c$
a/Å	8.3809(16)
b/Å	14.066(2)
c/Å	12.492(2)
α/°	90
β/°	98.533(16)
γ/°	90
Volume/Å^3	1456.3(4)
Z	4
ρ_{calc}g/cm^3	1.306
μ/mm^{-1}	0.087
F(000)	616.0
Crystal size/mm^3	0.321 × 0.212 × 0.121
Radiation	MoKα (λ = 0.71073)
2Θ range for data collection/°	6.666 to 50.694
Index ranges	$-10 \leq h \leq 10$, $-16 \leq k \leq 16$, $-15 \leq l \leq 15$
Reflections collected	17239
Independent reflections	2661 [R_{int} = 0.0502, R_{sigma} = 0.0362]
Data/restraints/parameters	2661/0/278
Goodness-of-fit on F^2	1.026
Final R indexes [I>=2σ (I)]	R_1 = 0.0429, wR_2 = 0.0995
Final R indexes [all data]	R_1 = 0.0603, wR_2 = 0.1100
Largest diff. peak/hole / e Å^{-3}	0.22/-0.30

Supplementary table 4. Crystal data and structure refinement for **7d**.

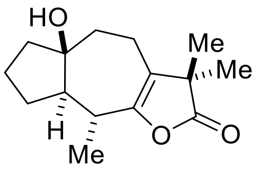

7d
CCDC number: 1825587

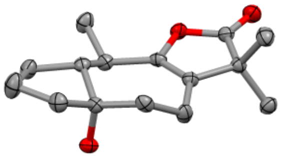

X-Ray of **7d**

Identification code	**7d**
Empirical formula	$C_{15}H_{22}O_3$
Formula weight	250.34
Temperature/K	150
Crystal system	monoclinic
Space group	$P2_1/c$
a/Å	8.3913(15)
b/Å	13.460(3)
c/Å	11.780(2)
α/°	90
β/°	93.941(15)
γ/°	90
Volume/$Å^3$	1327.4(4)
Z	4
ρ_{calc}g/cm^3	1.2526
μ/mm^{-1}	0.085
F(000)	544.3
Crystal size/mm^3	0.321 × 0.212 × 0.121
Radiation	Mo Kα (λ = 0.71073)
2Θ range for data collection/°	6.86 to 50.68
Index ranges	$-11 \leq h \leq 10$, $-18 \leq k \leq 16$, $-16 \leq l \leq 15$
Reflections collected	16851
Independent reflections	2416 [R_{int} = 0.0555, R_{sigma} = 0.0565]
Data/restraints/parameters	2416/0/167
Goodness-of-fit on F^2	1.035
Final R indexes [I>=2σ (I)]	R_1 = 0.0429, wR_2 = 0.0944
Final R indexes [all data]	R_1 = 0.0596, wR_2 = 0.1024
Largest diff. peak/hole / e $Å^{-3}$	0.28/-0.31

Supplementary Table 5. Crystal data and structure refinement for **7f**.

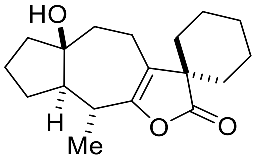

7f

CCDC number: 1825583

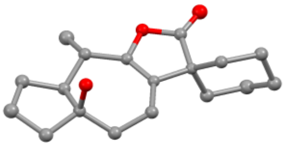

X-Ray of **7f**

Identification code	**7f**
Empirical formula	$C_{18}H_{26}O_3$
Formula weight	290.40
Temperature/K	150
Crystal system	orthorhombic
Space group	Pbca
a/Å	8.1487(4)
b/Å	10.3942(6)
c/Å	36.1900(16)
α/°	90
β/°	90
γ/°	90
Volume/Å^3	3065.3(3)
Z	8
ρ_{calc}g/cm^3	1.145
μ/mm^{-1}	0.079
F(000)	1057.0
Crystal size/mm^3	0.316 × 0.143 × 0.109
Radiation	MoKα (λ = 0.71073)
2Θ range for data collection/°	6.452 to 50.69
Index ranges	$-9 \le h \le 9$, $-12 \le k \le 11$, $-43 \le l \le 43$
Reflections collected	14758
Independent reflections	2801 [R_{int} = 0.0498, R_{sigma} = 0.0397]
Data/restraints/parameters	2801/0/87
Goodness-of-fit on F^2	1.059
Final R indexes [I>=2σ (I)]	R_1 = 0.0727, wR_2 = 0.1542
Final R indexes [all data]	R_1 = 0.0930, wR_2 = 0.1660
Largest diff. peak/hole / e Å^{-3}	0.60/-0.41

Supplementary Table 6. Crystal data and structure refinement for **7k**.

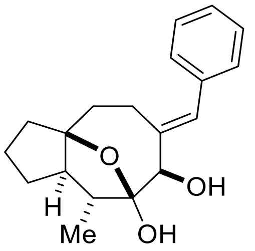

7k
CCDC number: 1825589

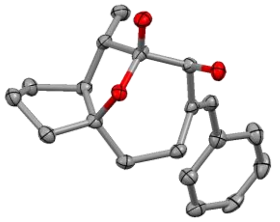

X-Ray of **7k**

Identification code	**7k**
Empirical formula	$C_{19}H_{24}O_3$
Formula weight	300.38
Temperature/K	150
Crystal system	tetragonal
Space group	$P4_2/n$
a/Å	19.6379(8)
b/Å	19.6379(8)
c/Å	8.2729(5)
α/°	90
β/°	90
γ/°	90
Volume/Å^3	3190.4(3)
Z	8
ρ_{calc}g/cm^3	1.251
μ/mm^{-1}	0.083
F(000)	1296.0
Crystal size/mm^3	0.295 × 0.112 × 0.098
Radiation	MoKα (λ = 0.71073)
2Θ range for data collection/°	6.56 to 50.696
Index ranges	$-18 \leq h \leq 21$, $-23 \leq k \leq 23$, $-9 \leq l \leq 9$
Reflections collected	13746
Independent reflections	2906 [R_{int} = 0.0484, R_{sigma} = 0.0412]
Data/restraints/parameters	2906/0/202
Goodness-of-fit on F^2	1.122
Final R indexes [I>=2σ (I)]	R_1 = 0.0923, wR_2 = 0.2580
Final R indexes [all data]	R_1 = 0.1128, wR_2 = 0.2721
Largest diff. peak/hole / e Å^{-3}	0.80/-0.26

Supplementary References

[1] Qian, M.; Covey, D. F. *Adv. Synth. Catal.* **2010**, *352* (11–12), 2057–2061.

[2] Liu W-B., Okamoto., N.; Alexy, E. J.; Hong, A. Y.; Tran, K., Stoltz, B. M. *J. Am. Chem. Soc.*, **2016**, *138* (16), 5234–5237.

[3] Peña-López, M.; Martínez, M. M.; Sarandeses, L. A.; Pérez Sestelo, J. *Org. Lett.*, **2010**, *12* (4), 852–854.

[4] Lafrance, M.; Roggen, M.; Carreira, E. M. *Angew. Chem. Int. Ed.* **2012**, *51* (14), 3470 –3473.

[5] Sibasish, P.; Sankha, P.; Surajit, S. *Tetrahedron Lett*. **2011**, *52* (46), 6166-6169.

[6] Szostak, M.; Spain, M.; Procter, D. J. *Nat. Protoc.* **2012**, *7* (5), 970–977.
